# Burden of Aortic Aneurysm and Its Attributable Risk Factors from 1990 to 2019: An Analysis of the Global Burden of Disease Study 2019

**DOI:** 10.3389/fcvm.2022.901225

**Published:** 2022-05-31

**Authors:** Zhuo Wang, Yayu You, Zhehui Yin, Qinyi Bao, Shuxin Lei, Jiaye Yu, Cuiping Xie, Feiming Ye, Xiaojie Xie

**Affiliations:** ^1^Department of Cardiology, The Second Affiliated Hospital, Zhejiang University School of Medicine, Hangzhou, China; ^2^International Institutes of Medicine, The Fourth Affiliated Hospital, Zhejiang University School of Medicine, Yiwu, China

**Keywords:** aortic aneurysm, Global Burden of Diseases Study, systematic analysis, mortality, disability-adjusted life year (DALY)

## Abstract

**Background:**

Global and national estimates on the epidemiology of aortic aneurysms are prerequisites for disease management and policymaking. Based on the Global Burden of Disease (GBD) 2019, this study aimed to discern the global aortic aneurysm burden by systematically analyzing demographic data on mortality and exploring the attributable risks and relevant factors.

**Methods:**

The data analyzed in this study were available in the Global Health Data Exchange (GHDx) online query tool. The population in our study comprised individuals from 204 countries and territories from 1990 to 2019. The estimated annual percentage changes (EAPCs) were performed to assess the temporal trends of aortic aneurysms and their attributable risks. Spearman correlation analysis was performed to explore the relationship between the burden of aortic aneurysm and covariates.

**Results:**

Although aortic aneurysm-related deaths (82.1%) and disability-adjusted life years (DALYs) (67%) increased from 1990 to 2019, the global trend of age-standardized rate of death (ASRD) (EAPC: −1.34, 95% CI = −1.46 to −1.22, *P* < 0.001) and age-standardized rate of DALY (ASDALYR) (EAPC: −1.06, 95% CI = −1.17 to −0.95, *P* < 0.001) decreased, both of which presented age dependence and gender differences. Smoking and high systolic blood pressure (SBP) were the main attributable risks of disease burden and tend to decease globally (EAPC: −1.89, 95% CI = −2.03 to −1.89, *P* < 0.001; −1.31 95% CI = −1.43 to −1.19, *P* < 0.001, respectively). Alcohol abstinence (male: *R* = −0.71, *P* < 0.001; female: *R* = −0.73, *P* < 0.001), smoking age of initiation (male: *R* = −0.32, *P* < 0.001; female: *R* = −0.50, *P* < 0.001), physical activity (male: *R* = −0.50, *P* < 0.001; female: *R* = −0.55, *P* < 0.001), and mean temperature (*R* = −0.62, *P* < 0.001) had negative correlation with ASRD. However, cholesterol level (male: *R* = 0.62, *P* < 0.001; female: *R* = 0.39, *P* < 0.001), body mass index (BMI) (male: *R* = 0.30, *P* < 0.001; female *R* = −0.01, *P* > 0.05), and alcohol consumption (male: *R* = 0.46, *P* < 0.001; female: *R* = 0.42, *P* < 0.001) had a positive correlation with ASRM. Besides, standard of living and medical resources positively related to burden of aortic aneurysm.

**Conclusion:**

In this study, a decreasing trend of aortic aneurysm burden was found globally, especially in advanced regions. Aged men who smoke and women who have hypertension should pay close attention to, particularly in deprived economic groups, and many approaches can be performed to reduce the burden of aortic aneurysms.

## Introduction

An aortic aneurysm is defined as a permanent localized dilatation of the aorta that is more than 50% of the predicted. It is usually developed in weak locations of the aorta and is classified by its location as a thoracic aortic aneurysm (TAA) and an abdominal aortic aneurysm (AAA). Most aortic aneurysms develop silently without any indications, causing sudden death due to aortic rupture with ~20% chance of survival. Unfortunately, to date, there is no effective medication to prevent or reverse the progression of the disease. Therefore, it is essential to comprehend the epidemiological traits of the disease and take effective interventions.

Unlike some dominant cardiovascular diseases such as coronary heart disease and stroke, less attention has been paid to aortic aneurysms by social economists and government officials. Previous studies suggested prevalence rates of AAA ranged from 1.6 to 7.2% in the general population aged 60 years or older (1). However, aortic aneurysm-related mortality is estimated at 150,000–200,000 deaths per year worldwide, which is equivalent to various types of cancer, e.g., bladder cancer (2), representing a considerable public health burden. Some risk factors, such as being elderly, gender differences, hypertension, smoking, and genetic or metabolic abnormalities, might contribute to the development of aortic aneurysm (3, 4).

The epidemiology of aortic aneurysms based on the global population has been described in previous studies based on the Global Burden of Disease (GBD) study. They focused on the association between healthcare access and quality index (HAQ index) system with aortic aneurysm mortality and YLLs in different incomes and age groups (5). Associations between estimated average percentage change (EAPC) and burden of aortic aneurysm, human development index (HDI) were found respectively (6), and aortic aneurysm-related death and attributable risks in the next decade were projected (7). However, they did extensive efforts on the health epidemiology of aortic aneurysms, and much remains to be performed. Compared with GBD2017 studies, the GBD2019 study used new methods to better measure risk factors by integrating the data of globally multiple high-quality epidemiological studies (8).

This study performed EAPC to quantify the trends of aortic aneurysm death and disability-adjusted life years (DALYs) and its four attributable risk factors based on GBD 2019 data (9). Furthermore, population attributable fractions (PAFs) were calculated to assess the impact of attributable risk factors in populations (10). In addition, we analyzed the association between the burden of aortic aneurysm and various covariates including physical condition, environment, occupation, socio-demographic factors, individual lifestyle, nutrition, and disease states using the Spearman rank-order correlation.

## Methods

### Study Data

All data analyzed, in this study, were available in the Global Health Data Exchange (GHDx) online query tool, which was conducted by the Institute for Health Metrics and Evaluation (IHME). The GBD 2019 is a comprehensive multinational epidemiological collaboration to provide an unbiased perspective estimation of population health over time and offers the opportunity to obtain estimates of incidence, prevalence, mortality, and health risk factors (8, 11). In brief, patients involved are identified from 86,249 sources including published studies, authoritative organization websites, and primary data sources of GBD collaborators (11). In the 2019 GBD database, aortic aneurysms, including both abdominal and thoracic aortic aneurysms, correspond to ICD-9 codes of 441–441.9 and ICD-10 codes of I71–I71.9 (11, 12). We presented data for five socio-demographic index (SDI) regions, four World Bank income (WBI) level groups, 21 GBD regions, and 204 countries and territories from 1990 to 2019, by age and sex. SDI is a comprehensive indicator including education, economics, and fertility rate. Based on SDI, the world was divided into 5 SDI regions, including low (0–0.454743), low-middle (0.4547430.45–0.607679), middle (0.607679–0.689504), high-middle (0.689504–0.805129), and high (0.805129–1) SDI regions (Supplementary Table 1) (13). Income is calculated using the World Bank Atlas method to convert local currency to gross national income (GNI) per capita, in dollars (14). Based on World Bank income (WBI) levels, the world was divided into 4 WBI regions including low (≤1,035 $), lower-middle (1,036–4,045$), upper-middle (4,046–12,535$), and high (> 12,535$) WBI region (Supplementary Table 2) (14, 15).

Mortality data of aortic aneurysms from multiple versions of the International Classification of Diseases and Injuries (ICD) were analyzed and matched to the GBD 2019 cause list (11). Cause of Death Ensemble model (CODEm) and DisMod-MR 2.1 were used to standardize data for global and regional estimates (11). In brief, CODem produces a wide range of submodels with different functional forms on the same data to best reflect all the available input data (16, 17). DisMod-MR 2.1 was a Bayesian meta-regression model used to pool epidemiological outcomes and assessed the age-sex-location-year-specific burden of aortic aneurysms (18). More detail on CODem and DisMod-MR 2.1 could be seen in previous studies (11).

Disability-adjusted life years were the sum of the number of years of life lost (YLLs) and the number of years lived with disability (YLDs). In brief, YLLs are based on cause-specific prevalence and disability weight, which can be calculated by deaths being multiplied by standard life expectancy at each age. YLDs were calculated by disability weights for mutually exclusive sequela multiplying the prevalence of disease (11). The formulas of YLLs and YLDs are as follows:

$$\begin{array}{r} \text{YLL }=\text{ N }\times \text{ L}1\\ \text{YLD}=\text{I}\times \text{ DW}\times \text{ L}2=\text{P }\times \text{ DW} \end{array}$$

where *N* refers to the number of deaths; L1 for standard life expectancy at age of death in years; I for the number of incident cases; DW for disability weight; L2 for the average duration of disability years; and *P* for the number of prevalent cases.

Furthermore, the GBD 2019 study provided 87 risk factors at the global and regional levels using the comparative risk assessment framework (CRA). In brief, CRA can be divided into six key steps: (1) inclusion of risk-outcome pairs; (2) estimation of relative risk; (3) estimation of distributions and exposure; (4) determination of the counterfactual level of exposure and the theoretical minimum risk exposure level (TMREL); (5) computation of PAF and attributable burden; and (6) computation of the burden attributable to combinations of risks (8). Four attributable risks of aortic aneurysm burden were found including smoking, high systolic blood pressure (SBP), diet high in sodium, and lead exposure. PAF, also called the population attributable proportion or attributable proportion among the total population, is the estimated fraction of all cases that would not have occurred if there had been no exposure. Therefore, the computational formula of PAF is as follows:

$$\begin{array}{l} \text{PAF}=\frac{A}{O}\times 100\% \end{array}$$

where *O* and *A* refer to the observed number of cases and the number of cases that can be attributed to exposure, respectively (8, 11, 19). EAPC of the four attributable risks was calculated as well.

In addition, various covariates were downloaded from GBD 2019 covariate dataset (https://cloud.ihme.washington.edu/s/b2tQnbsjAyWgeHm?path=%2FGBD%202019%20Covariates). The dataset contained information of every country and its province. The correlation between the burden of aortic aneurysm and covariates was further analyzed, and the correlation coefficient (*R*) was calculated at the level of groups.

### Statistical Analysis

The age-standardized rates (ASRs), including the age-standardized rate of death (ASRD) and age-standardized DALYs rate (ASDALYR), were calculated to make valid comparisons between different groups. In brief, the sum of the products of age-specific rates (α_i_) and the number of persons (or weight) (*w*_i_) in the same age subgroup *i* of the standard population and then divide the sum of the standard population weights. The formula of ASR (per 100,000 populations) is as follows:

$$\begin{array}{l} \text{ASR}=\frac{\sum_{i=1}^{A}a_{i}w_{i}}{\sum_{i=1}^{A}a_{i}}\times 100,00 \end{array}$$

where *i* denotes the *i*th age class (11). The temporal trend of ASR of the aortic aneurysm was quantified using the estimated annual percentage change (EAPC), calculated using log-linear regression. It is assumed that the natural logarithm of ASR is linear along with time. Thus, *Y* = α + β*X* + ε, where *Y* refers to ln(ASR), *X* represents the calendar year, and ε represents the error term. Based on this formula, β determines the positive or negative trends of ASR. The formula for calculating EAPC is as follows:

$$\begin{array}{l} \text{EAPC }=\;100\;\times \;\left(exp\left(\beta \right)\;-\;1\right). \end{array}$$

In addition, its 95% confidence interval (CI) was computed similarly. When the EAPC was positive, the ASR was deemed to be increasing, while the ASR was decreasing when EAPC was negative (20, 21). In addition, Spearman's correlation coefficient was calculated for the correlation between ASRD, ASDALYR, and covariates. Data analysis was performed using the open-source software R (version 4.1.0) with the package of “ggplot2,” “ggpubr,” “tidyverse,” “data.table,” and “Hmisc.” A 2-tailed *P* < 0.05 was considered statistically significant.

## Results

### Mortality of Aortic Aneurysm

#### Deaths of Aortic Aneurysm Globally

Aortic aneurysm led to 172,427 deaths (95% UI = 157,357–182,899) in 2019, which increased 82.1% from the 94,698 deaths (95% UI = 87,009–102,685) in 1990 (Supplementary Table 3). Aortic aneurysm-related deaths presented the slowest increase by 33.5% in the high SDI region from 1990 (892,344, 95% UI = 860,937–915,453) to 2019 (1,013,966, 95% UI = 930,098–1,063,166), whereas they nearly doubled in the other four SDI regions (Supplementary Table 3). Regionally, the fastest increase of deaths caused by aortic aneurysms was found in the United Arab Emirates (more than 8.3-folds), followed by Taiwan China (4.9-folds) and Qatar (4.87-folds), whereas the fastest decrease found in Niue (−28.17%) from 1990 to 2019. The maximal death numbers caused by an aortic aneurysm in 2019 were found in Japan (20,169, 95% UI = 16,270–22,321) (Supplementary Table 4).

#### ASRD of Aortic Aneurysm

**Figure 1:**
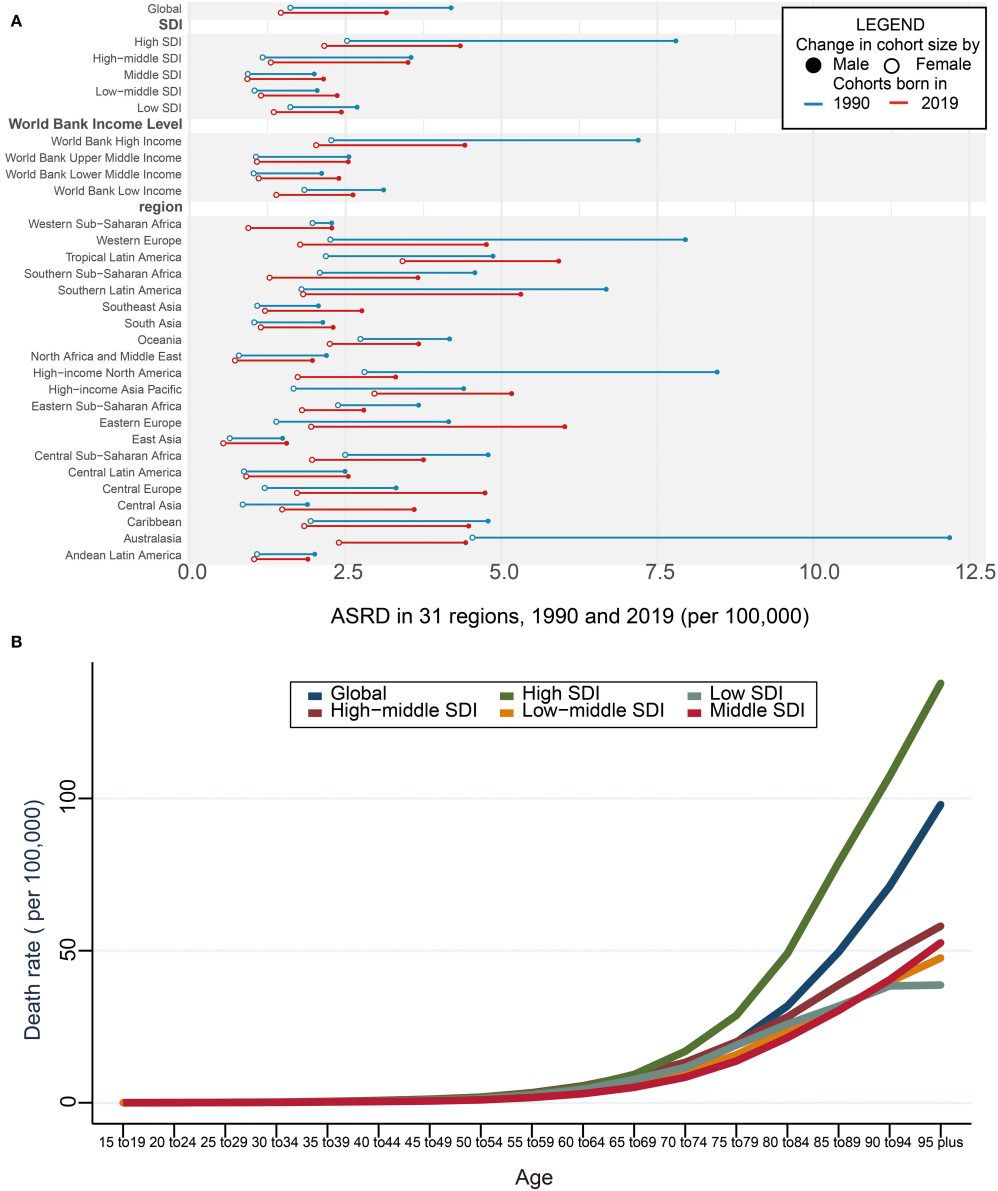
Death and ASRD of aortic aneurysm. **(A)** Global ASRD with 31 regions from 1990 to 2019. The blue line represents differences in ASRD of men and women in 1990 and the red line represents 2019 year. The black solid circle indicates the male group and the hollow circle the female group. **(B)** Global ASRD stratified by age and SDI with 5 SDI regions in 2019. SDI, sociodemographic index; ASRD, age-standardized death rate.

Conversely, the global ASRD of aortic aneurysm decreased by 17.9% from 2.70/100,000 (95% UI = 2.47–2.91/100,000) in 1990 to 2.21/100,000 (95% UI = 2.00–2.35/100,000) in 2019 (Supplementary Table 3), with decreasing by 24.74% in men and 9.45% in women (Figure 1A; Supplementary Table 3). The ASRD of aortic aneurysm dramatically decreased by 44.34% in men in the high SDI region whereas the most increase was found in men in the low-middle SDI region (15.68%) (Figure 1A; Supplementary Table 3). Consistent results were found in the ASRD of aortic aneurysm stratified by WBI Levels (Figure 1A; Supplementary Table 3). Regionally, the highest ASRD of the aortic aneurysm was found in tropical Latin America in 2019 (4.53/100,000, 95% UI = 4.14–4.84/100,000) and the lowest ASRD of the aortic aneurysm was found in East Asia (0.98/100,000, 95% UI = 0.84–1.12/100,000) (Supplementary Table 3). The highest decrease in the ASRD of the aortic aneurysm was found in Australasia (-56.9%), with −63.7% in men and −45.9% in women. However, men in central Asia had the largest increase in ASRD (90.63%) (Figure 1A; Table 1).

**Table 1:** The relevant factors of aortic aneurysm burden in 2019.

**Item**	**ASDALYR**			**ASRD**		
	**Man**	**Female**	**Both**	**Man**	**Female**	**Both**
**Physical condition**						
Population over age 65	0.59	0.49		0.62	0.53	
Cholesterol	0.60	0.40		0.62	0.39	
Bone mineral density	0.74	−0.46		0.78	−0.49	
BMI	0.30	−0.02^*^		0.30	−0.01^*^	
Obesity	0.21	−0.03^*^		0.24	−0.04^*^	
Diabetes fasting plasma glucose (mmol/L)	0.11	−0.05^*^		0.11	−0.04^*^	
**Disease prevalance**						
Alcoholic cirrhosis	0.67	0.54		0.70	0.57	
Diabetes	−0.55	−0.55		−0.60	−0.59	
HepA	−0.67	−0.59		−0.72	−0.63	
HepB	−0.40	−0.43		−0.45	−0.47	
Melanoma	0.64	0.63		0.68	0.69	
Severe anemia	−0.53	−0.33		−0.59	−0.40	
Tuberculosis	0.60	0.39		0.63	0.39	
**Lifestyle**						
Alcohol abstain	−0.68	−0.68		−0.71	−0.73	
Alcohol binge	0.74	0.69		0.76	0.72	
Alcohol g/day	0.45	0.39		0.46	0.42	
Cannabis dependence		0.54			0.61	
Age of smoking initiation	−0.28	−0.48		−0.32	−0.50	
Physical activity MET-min/week	−0.48	−0.54		−0.50	−0.55	
Agricultural activities	−0.64	−0.56		−0.70	−0.51	
**Diet habit**						
Energy kcal/p/day			0.22			0.24
Fruits g/p/d			−0.05^*^			−0.03^*^
Milk g/day			0.50			0.53
Poultry g/day			0.31			0.33
Pufa			0.32			0.33
Red meats			0.47			0.48
Sugar g/p/d			0.20			0.26
Vegetables g/day			0.27			0.30
**Nutrient**						
Calcium g/day			0.54			0.58
Iron mg/day			0.41			0.44
Vitamin A ug/day			0.36			0.41
Zinc			0.31			0.34
**Environment and occupation**						
Asbestos consumption			0.34			0.31
Coal production (per capita)			0.70			0.64
Latitude			0.63			0.68
Mean temperature			−0.57			−0.62
People living at 500–1,500 m elevation			−0.51			−0.55
People living at above 1,500 m elevation			−0.53			−0.55
**Education and health support**						
Education			0.53			0.58
GDP-PPP			0.30			0.35
Fraction of health expenditure			−0.40			−0.45
HAQI			0.58			0.65
Health expenditure (per capita)			0.55			0.50
Health worker density			0.64			0.69
Hospital beds per 1,000			0.52			0.52
Health industry workers			0.71			0.76
Pharmacists per capita			0.53			0.58
Physicians per capita			0.48			0.52
Sanitation			0.53			0.56
UHC			0.59			0.65

*UHC, universal health coverage; BMI, body mass index; PUFA, polyunsaturated fatty acid; asbestos consumption, estimated as production plus imports minus exports (metric tons per year per capita); health industry workers, the proportion of the employed population aged 15–69 years working in health and social work; education, mean level of educational attainment*.

* *p > 0.05*.

#### Age and ASRD of Aortic Aneurysm

It has been demonstrated that the ASRD of the aortic aneurysm was positively related to age, gradually increasing after the age of 65 years. Compared with the patients aged 65-69 years, the ASRD of aortic aneurysm had a 1.7-fold increase in those aged 75–79 years, a 5.7-fold increase in those aged 85–89 years, and a 12.3-fold increase in those aged more than 95 years. Among the 5 SDI regions, the fastest elevation of the ASRD of the aortic aneurysm was found in the high SDI region in the elderly patients (Figure 1B; Supplementary Table 5).

### DALYs and Its ASR of Aortic Aneurysm

#### DALYs of Aortic Aneurysm

Global DALYs caused by aortic aneurysm increased 67.0% from 1990 (1,989,613.52, 95% UI = 1,819,554.20–2,192,796.33) to 2019 (3,322,343.13, 95% UI = 3,107,724.62–3,524,925.22) (Supplementary Table 3) especially in the low-middle SDI region. The high SDI region presented the slowest increase by 13.6% from 1990 to 2019 but the fastest was in the low-middle SDI region (150.55%) (Supplementary Table 3). Regionally, the highest DALYs in 2019 were observed in China, followed by India, Japan, the United States of America, and Brazil (Supplementary Table 4).

#### ASDALYR of Aortic Aneurysm

**Figure 2:**
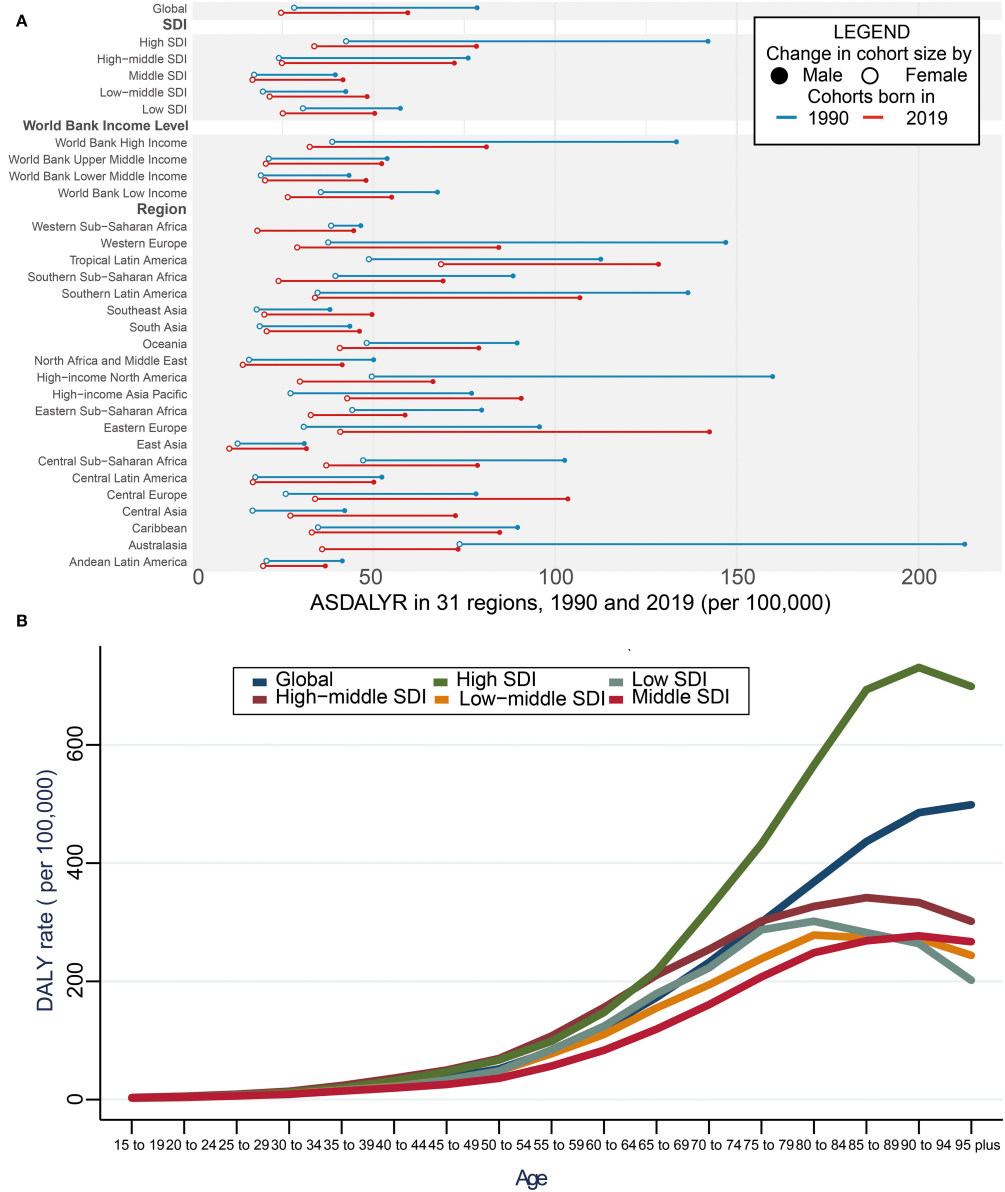
DALYs of aortic aneurysm. **(A)** Global ASDALYR with 31 regions from 1990 to 2019. The blue line represents differences in ASDALYR of men and women in 1990 and the red line represents 2019 year. The black solid circle shows the male group and the hollow circle the female group. **(B)** Global ASDALYR stratified by age and SDI with 5 SDI regions in 2019. SDI, sociodemographic index; DALY, disability-adjusted life year rate; ASDALYR, age-standardized DALY rate.

On the contrary, the ASDALYR caused by aortic aneurysm declined by 24.1% from 50.79/100,000 (95% UI = 46.50–55.66) in 1990 to 40.94/100,000 (95% UI: 38.20–43.43) in 2019 (Supplementary Table 3). A similar trend was noted in both genders, with a 24.3% decrease in men and 12.7% in women. The changes in different WBI levels were consistent with those of SDI regions (Figure 2A; Supplementary Table 3). Australasia showed the greatest decrease of ASDALYR in both genders during the past 30 years, presenting 53.49/100,000 (95% UI = 48.10-57.71/100,000) in 2019, which was almost one-third of that in 1990 (Figure 2A; Supplementary Table 3). Regionally, the highest ASDALYR was observed in Montenegro, followed by Armenia, Brunei Darussalam, Saint Lucia, and Fiji (Supplementary Table 4).

#### Age and ASDALYR of Aortic Aneurysm

In addition, the global ASDALYR increased with age in 2019, which slightly increased in patients aged below 55 years but grew dramatically among patients aged over 55 years. The other four SDI regions remained stable. Compared with the patients aged 65–69 years, the ASDALYR of aortic aneurysm had a 73% increase in those aged 75–79 years, a 1.5-fold increase in those aged 85–89 years, and a 1.9-fold increase in those aged over 95 years, respectively. In high SDI countries, patients aged below 65 years shared a similar increasing rate with those from the other four regions. However, patients aged over 65 years had the fastest elevation (Figure 2B; Supplementary Table 5).

### EAPC of ASRD and ASDALYR

#### EAPC of ASRD

**Figure 3:**
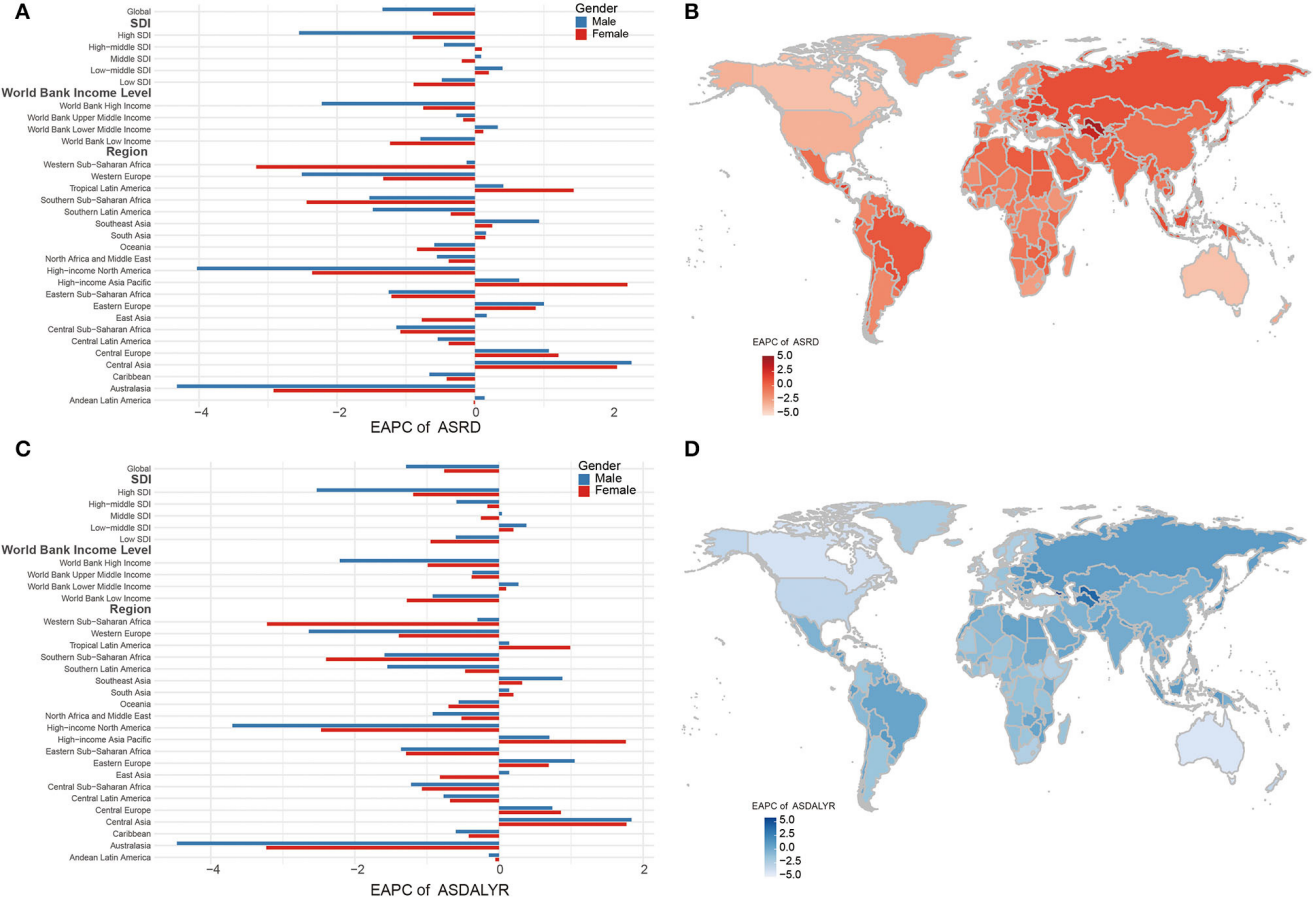
EAPC in ASRD and ASDALYR. **(A)** Global EAPC of ASRD with 31 regions. **(B)** EAPC of ASRD in 204 countries and territories in 2019. **(C)** Global EAPC of ASDALYR in 30 regions. **(D)** EAPC of ASDALYR in 204 countries and territories in 2019. EAPC, estimated annual percentage change; ASRD, age-standardized death rate; ASDALYR, age-standardized DALY rate.

The global trend of ASRD from 1990 to 2019 decreased with EAPC being −1.34 (95% CI = −1.46 to −1.22, *P* < 0.001) in men and −0.61 (95% CI = −0.71 to −0.50, *P* < 0.001) in women (Figure 3A; Supplementary Table 6). The greatest decrease was found in high SDI region for both genders (male: −2.55, female: −0.9), while a rising tendency of ASRD was observed in low-middle SDI regions with EAPC being 0.4 (95% CI = 0.33–0.47, *P* < 0.001) in men and 0.2 (95% CI = −0.14 to 0.27, *P* < 0.001) in women (Figure 3A; Supplementary Table 6). Regionally, the fastest decrease of ASRD was found in Australasia with EAPC being −4.3 (95% CI = −4.62 to −4.02, *P* < 0.001) in men and −2.92 (95% CI = −3.16 to −2.68, *P* < 0.001) in women (Figure 3A; Supplementary Table 6). The trends in male patients of high-income North America and female patients of Western and Southern Sub-Saharan Africa dramatically declined, in contrast to the moderate increase of trends in male patients of Central Asia and female patients of the high-income Asia Pacific and the high-income Asia Pacific and Tropical Latin America (Figure 3A).

The fastest decline of ASRD was found in the Northern Mariana Islands, followed by Guam, Australia, Canada, and the United States of America. In contrast, the top five positive EAPCs of ASRD were found in Georgia, Uzbekistan, Turkmenistan, Taiwan China, and Armenia (Figure 3B; Supplementary Table 7).

#### EAPC of ASDALYR

Consistent trends were discovered in the ASDALYR (EAPC: −1.06, 95% CI = −1.17 to −0.95, *P* < 0.001). The global EAPC of ASDALYR from 1990 to 2019 was −1.29 (95% CI = −1.40 to −1.18, *P* < 0.001) for men and 0.76 (95% CI = −0.86 to −0.65, *P* < 0.001) for women. The greatest decrease was found in the high SDI region for both genders, while a raising trend was found in low-middle SDI in both genders. The consistent trends were observed in the regions by WBI level stratification (Figure 3C; Supplementary Table 6). Regionally, the fastest decreasing of ASDALYR was found in Australasia, with EAPC being −4.47 (95% CI = −4.78 to −4.16, *P* < 0.001) in men and −3.23 (% CI = −3.48 to −2.98, *P* < 0.001) in women. The tendency of ASDALYR in male patients of high-income North America and female patients of Western and Southern Sub-Saharan Africa dramatically declined, in contrast to the moderate increase of ASDALYR in male patients of Central Asia and female patients of high-income Asia Pacific, high-income Asia Pacific, and Tropical Latin America (Figure 3C).

The fastest decline of ASDALYR was found in the Northern Mariana Islands, followed by Australia, Canada, Uzbekistan, Turkmenistan, Philippines, and Taiwan China (Figure 3D; Supplementary Table 7).

### Attributable Risks of Aortic Aneurysm

Four attributable risks of aortic aneurysm were concluded in GBD 2019, including smoking, high SBP, diet high in sodium, and lead exposure.

**Figure 4:**
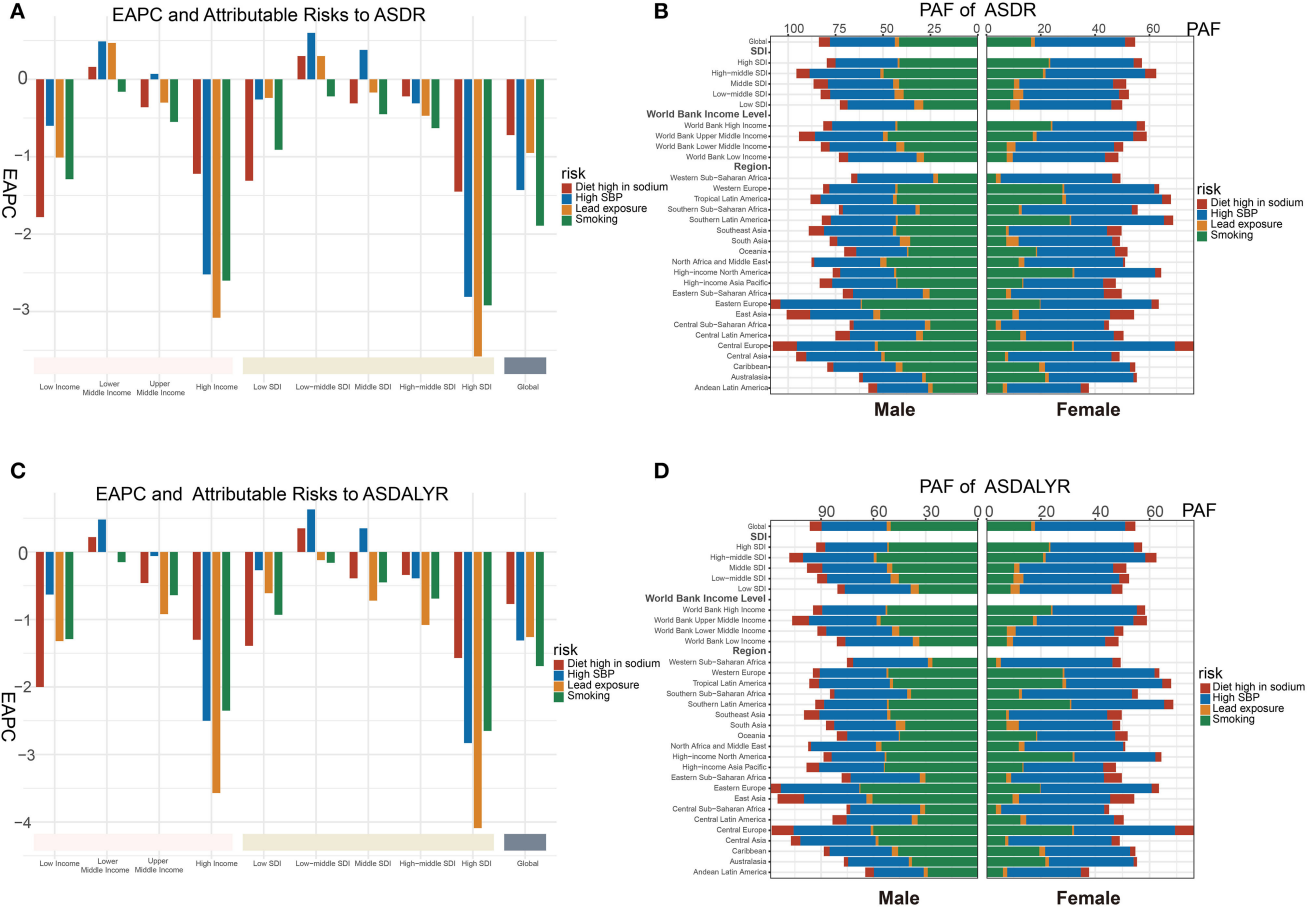
Attributable risks of aortic aneurysm burden. **(A)** PAF of attributable risks of ASRD in 2019. **(B)** PAF of attributable risks of ASDALYR in 2019. **(C)** Global EAPC of attributable risks of ASRD with 30 regions. **(D)** Global EAPC of attributable risks of ASDALYR with 30 regions. PAF, population attributable fraction; ASRD, age-standardized death rate; ASDALYR, age-standardized DALY rate; EAPC, estimated annual percentage change.

The four attributable risks to ASRD showed dramatic decreasing in the world especially in the high SDI region or high WBI region (Figure 4A). Smoking, attributable to ASDR, had a reduced trend around the world (EAPC: −1.89 95% CI = −2.03 to −1.89, *P* < 0.001) (Figure 4A) especially in men in Australasia with EAPC being −5.8 (95% CI = −6.13 to −5.46, *P* < 0.001) but increased in central Asia (EAPC: 1.83 95% CI = 1.68 to 1.97, *P* < 0.001) and eastern Europe (EAPC: 1.88, 95% CI = 1.46 to 2.3, *P* < 0.001) (Supplementary Table 8). ASRD of aortic aneurysm from high SBP had a rising tendency in middle (EAPC: 0.38, 95% CI = 0.28 to 0.47, *P* < 0.001) and low-middle SDI regions (EAPC: 0.6, 95% CI = 0.55 to 0.66, *P* < 0.001) (Figure 4A; Supplementary Table 8). Regionally, ASRD of aortic aneurysm from high SBP had a biggest increase in men in central Asia with EAPC being 2.25 (95% CI = 2.11 to 2.38, *P* < 0.001) and a highest decrease in men in Australasia (EAPC: −5.21, 95% CI = −5.59 to −4.83, *P* < 0.001) (Supplementary Table 8). Diet high in sodium, another attributable risk, had a slight rising trend in low middle SDI region (EAPC: 0.3, 95% CI = 0.26 to 0.35, *P* < 0.001) and in lower middle WBI region (EAPC: 0.16, 95% CI = 0.12 to 0.20, *P* < 0.001) (Figure 4A; Supplementary Table 8). Lead exposure had a biggest drop in high SDI religion (EAPC: −3.58, 95% CI = −3.81 to −3.85, *P* < 0.001) (Figure 4A; Supplementary Table 8).

Smoking was globally considered the major contributor to ASRD of aortic aneurysms in male patients (PAF = 33.02%) while high SBP is the major contributor for female patients (PAF = 34.11%). Regionally, the highest PAF of smoking was found in male patients in Eastern Europe (61.1%), as well as female patients in high-income North America (31.7%). Meanwhile, the highest PAF of high SBP was found in female patients in Western Sub-Saharan Africa (41.05%), as well as male patients of Eastern Europe (42.2%) (Figure 4BA; Supplementary Table 9).

Similar tendency and PAF of the four attributable risks were seen in ASDALYR of aortic aneurysm. The highest PAF of smoking was found in the ASDALYR of male patients in Eastern Europe (67.4%), as well as female patients of Central Europe (41.6%). Meanwhile, the highest PAF of high SBP in the ASDALYR was found in both genders (male: 45.0%; female: 43.3%) of Eastern Europe (Figures 4C,D; Supplementary Tables 8, 9).

### Relevant Factors of ASRD and ASDALYR

To explore the promising intervention strategies for aortic aneurysms, the correlation of population exposure factors with ASRD and ASDALYR was analyzed, including physical condition, disease prevalence, lifestyle, diet habits, nutrient environment, education, economics, and health support.

#### Physical Condition and Burden of Aortic Aneurysm

Population aged over 65 years (male: *R* = 0.62, *P* < 0.001; female: *R* = 0.53, *P* < 0.001) and cholesterol level (male: *R* = 0.62, *P* < 0.001; female: *R* = 0.39, *P* < 0.001) had a positive moderate correlation with ASRD of aortic aneurysm in both genders. However, there are differences between men (*R* = 0.78, *P* < 0.001) and women (*R* = −0.49, *P* < 0.001) in correlation between bone mineral density and ASRD of aortic aneurysm. Body mass index (BMI), obesity, and fasting plasma glucose have a weak relationship with ASRD in men (*R* = 0.30, *P* < 0.001; *R* = 0.24, *P* < 0.001; *R* = 0.11, *P* < 0.01, respectively) but negligible in women (*R* = −0.01, *P* > 0.05; *R* = −0.04, *P* > 0.05, respectively). The relationship between physical condition and ASDALYR of aortic aneurysm was similar (Table 1), and other physical condition could be found in Supplementary Tables 10, 11.

#### Disease Prevalence and Burden of Aortic Aneurysm

In terms of disease prevalence, age-standardized prevalence of diabetes (male: *R* = −0.60, *P* < 0.001; female: *R* = −0.59, *P* < 0.001), hepatitis A (male: *R* = −0.72, *P* < 0.001; female: *R* = −0.63, *P* < 0.001), hepatitis B (male: *R* = −0.45, *P* < 0.001; female: *R* = −0.47, *P* < 0.001), and severe anemia (male: *R* = −0.59, *P* < 0.001; female: *R* = −0.40, *P* < 0.001) was negatively correlated with ASRD. In contrast, age-standardized prevalence of alcoholic cirrhosis (male: *R* = 0.70, *P* < 0.001; female: *R* = 0.57, *P* < 0.001), melanoma (male: *R* = 0.68, *P* < 0.001; female: *R* = 0.69, *P* < 0.001), and tuberculosis (male: *R* = 0.63, *P* < 0.001; female: *R* = 0.39, *P* < 0.001) was positively correlated with ASRD and ASDALYR (*P* < 0.001). Consistent results were found in the relationship between ASDALYR and those diseases (Table 1). The relationship between the other disease and burden of aortic aneurysm is shown in Supplementary Tables 10, 11.

#### Lifestyle and Burden of Aortic Aneurysm

As for the lifestyle, age-standardized proportions of alcohol abstinence were negatively correlated with ASRD using R being −0.71 in the male group and −0.73 in the female group. But alcohol binge (male: *R* = 0.76, *P* < 0.001; female: *R* = 0.72, *P* < 0.001) and alcohol consumption (male: *R* = 0.46, *P* < 0.001; female: *R* = 0.42, *P* < 0.001) had a positive relationship. Taking part in physical activity (male: *R* = −0.50, *P* < 0.001; female: *R* = −0.55, *P* < 0.001) and farm work (male: *R* = −0.70, *P* < 0.001; female: *R* = −0.51, *P* < 0.001) was negatively related to ASRD. Additionally, the later the smoking age, the lower the mortality rate (male: *R* = −0.32, *P* < 0.001; female: *R* = −0.50, *P* < 0.001). Age-standardized prevalence of cannabis dependence in women of reproductive-age was positively correlated with ASRD (female: *R* = 0.61, *P* < 0.001) (Table 1). The relationship between the other life style and burden of aortic aneurysm is shown in Supplementary Tables 10, 11.

#### Diet Habit and Burden of Aortic Aneurysm

As for diet habits, positive correlation was found in daily intake of energy (*R* = 0.24, *P* < 0.001), milk (*R* = 0.53, *P* < 0.001), poultry (*R* = 0.33, *P* < 0.001), polyunsaturated fatty acids (PUFA) (*R* = 0.33, *P* < 0.001), red meats (*R* = 0.48, *P* < 0.001), sugar (*R* = 0.26, *P* < 0.001), and vegetables (*R* = 0.30, *P* < 0.001) with ASRD (Table 1). The relationship between the other diet habit and burden of aortic aneurysm is shown in Supplementary Tables 10, 11.

#### Nutrient and Burden of Aortic Aneurysm

Daily intake of calcium (*R* = 0.58, *P* < 0.001), iron (*R* = 0.44, *P* < 0.001), vitamin A (*R* = 0.41, *P* < 0.001), and zinc (*R* = 0.34, *P* < 0.001) was positively related to ASDR of aortic aneurysm (Table 1). The relationship between the other nutrient and burden of aortic aneurysm is shown in Supplementary Tables 10, 11.

#### Environment and Burden of Aortic Aneurysm

In the aspect of environment, population-weighted mean temperature (*R* = −0.62, *P* < 0.001) and proportion of the population living altitude 500–1,500 m (*R* = −0.55, *P* < 0.001) and 1,500 m plus (*R* = −0.55, *P* < 0.001) had negative correlation with ASRD, whereas social coal production (*R* = 0.64, *P* < 0.001) and proportion of the population living latitude (*R* = 0.68, *P* < 0.001) showed positive correlation with ASRD (Table 1). The relationship between the other environment factors and burden of aortic aneurysm is shown in Supplementary Tables 10, 11.

#### Education, Economics, Health Support, and Burden of Aortic Aneurysm

Considering education, economics, and health support, positive correlation was found age-standardized level of educational attainment (*R* = 0.58, *P* < 0.001), GDP-purchase power parity (GDP-PPP) (*R* = 0.35, *P* < 0.001), healthcare access and quality index (HAQI) (*R* = 0.65, *P* < 0.001), health expenditure (*R* = 0.50, *P* < 0.001), health worker density (*R* = 0.69, *P* < 0.001), hospital beds per 1,000 (*R* = 0.52, *P* < 0.001), health industry workers (*R* = 0.76, *P* < 0.001), pharmacists per capita (*R* = 0.58, *P* < 0.001), physicians per capita (*R* = 0.52, *P* < 0.001), and Universal health coverage (UHC) (*R* = 0.59, *P* < 0.001) with ASRD (Table 1).

## Discussion

In this study, we comprehensively analyzed the current burden and trends in the deaths and DALY of aortic aneurysms at global and regional levels from 1990 to 2019 based on the GBD 2019 study.

Our results revealed that aortic aneurysms remained a public health concern, with progressive effects on deaths and DALYs but declining in ASRD and ASDALYR. The presence of heterogeneities was found in the level of gender, age, social economy, and geography. Smoking and high SBP, major attributable risks to aortic aneurysms in the male group and female group, respectively, were trending downward. Various covariates in the aspect of physical condition, disease prevalence, lifestyle, diet habits, nutrients, environment, education, economics, and health support were found relative to the burden of aortic aneurysm. Our results might serve as an important extension to the previous knowledge and provide intervention targets for clinical scientists and prevention strategies for socio-economists.

Similar to previous global epidemiological reports of aortic aneurysms based on the GBD 2017 study, the decreasing tendency of ASRD and ASDALYR was observed particularly in high SDI and high WBI regions. Stefanos Tyrovolas and his colleagues suggested that a higher HAQI had a relationship with lower mortality and YLLs of aortic aneurysm in multi-level mixed modeling (5). Compared with YLLs, DALY may be more suitable to describe the burden of disease for not all aortic aneurysms being fatal (22). Additionally, the team of Linyan Wei described qualitatively attributable risk factors changes and found high SBP and smoking-caused aortic aneurysm decreased (6). Similar results were found in our study with quantitative analysis including EAPC and PAF. Based on GDB 2019 study, another research predicted a rebounding tendency in death of aortic, and high SBP would be the major risk factor (7). However, according to our result, a decreasing trend in ASRD, ASDALYR, and attributable risks was obtained, which means that the burden of aortic aneurysms would have a continuing decline globally. The difference could be explained that Huang et al. calculated the average annual percentage change every 3 years, not as a general tendency.

Our study generated some novel insights. Besides two attributable risk factors described in previous studies (smoking, high SBP), we calculated PAF and EAPC of two new attributable risks (diet high in sodium and lead exposure) of the aortic aneurysm to quantify the effect and trend. Additionally, we found that generally, smoking remains a major attributable risk factor to aortic aneurysms for men and high SBP for women. All attributable risk factors globally tend to decrease, but high SBP had poor management in underdeveloped areas. Moreover, we explored the relationship between various covariates and ASRD and ASDALYR of aortic aneurysms and found areas having rich medical resources and high-quality life tended to higher burden of aortic aneurysms. This tendency may be explained by the increase in human lifespan and the number of people aging, more advanced diagnosis and treatment techniques, and better health awareness in developed countries (12, 23). The latest deaths and DALY estimates of aortic aneurysms differed widely across regions. In the high SDI region, especially in Australasia and high-income North America, the trend of aortic aneurysm burden was the most rapid decline, whereas a slight upward trend was observed in less developed areas such as some Asia and Tropical Latin America regions. Inversely, the burden of aortic aneurysms is greater in developed regions than developing regions, just as the previous reports indicated (6, 24). This difference suggested that the burden of aortic aneurysms can be under control and under diagnosis of aortic aneurysms occurred in developing regions (24). Therefore, effective means can be performed to improve the level of diagnosis and treatment and ease the burden of aortic aneurysms.

Low-cost screening for aortic aneurysms was effective preventive care and performed more in advanced countries such as Australia and the UK (25–27). Population-wide screening projects in men aged 65 years had shown a prevalence of AAAs as low as 1.0–1.5% in Sweden (28) and the UK (29). A high-quality meta-analysis shows that screening for men 65 years or older could reduce AAA-related mortality by 35–45% but lead to higher rates of surgery (1). Compared with those with no population-based screening, such as Hungary, Austria, and Romania, the mortality rate of AAA in countries with population-based screening, such as the UK, Australia, and Sweden, constantly declines (30). Therefore, population-wide screening projects for aortic aneurysms were necessary for deprived economic groups. Since screening with conventional ultrasound for aortic aneurysms would make a considerable cost to the developing countries, handheld portable echo devices may be a better choice given their cost performance higher and easy availability (31).

Furthermore, it was necessary to improve education attainment and medical quality for aortic aneurysms. Within the past few years, some training programs for endovascular aneurysm repair have been organized by the Australian Vascular Surgery Community to elevate the surgery success and thus improve the prognosis for the patients (26). Additionally, minimally invasive surgery for AAA was a preferred method in the developed world since it lowered mortality and quicker recovery (32).

In addition, investing health system resources in the control of risk factors was important, especially smoking cessation and hypertension management. Our results suggested that attributable risk factors of aortic aneurysm had the most obvious downward trend in high SDI regions which is in accordance with the trend of aortic aneurysm burden. In most regions, smoking was the dominant contributor to aortic aneurysm burden in male patients and high SBP in female patients. The burden of aneurysms associated with smoking was decreasing but hypertension tended to increase in the middle and low SDI regions. Previous studies found that men aged ≥65 years have decreasing prevalence rates of AAA largely owning to smoking cessation in the developed region (33). As Laroche et al. (34) suggested, the reduction of AAA prevalence was parallel to a reduction in cigarettes and tobacco consumption. A significant measure of smoking cessation is that most low-income countries do not follow WHO recommendations for tobacco cessation, resulting in a slow reduction in tobacco consumption (35). Compared with that in high-income countries, hypertensive patients in low-income countries had lower proportions of awareness, treatment, and control (36). As a result, deprived economic groups had an increasing trend of aortic aneurysm burden with poor management of hypertension and slow reduction of tobacco consumption.

Some covariates related to the burden of aortic aneurysm also provided ideas of prevention and control. Our results suggested that temperature, alcohol abstain, age of smoking initiation, physical activity, and altitude had a negative relationship with the burden of aortic aneurysm, whereas aging, cholesterol, BMI, and obesity had a positive relationship. Supporting our results, Chen J and his team performed a time-stratified case-crossover study and found that a rising in AAD risk was associated with lower temperatures when the mean temperature was below 24°C (37). Alcohol consumption and physical activity was found associated with aortic aneurysm with HR = 1.15(95% CI = 1.03 to 1.28) (38) and HR = 0.54 (95% CI = 0.34 to 0.93) (39). Moderate-intensity exercise was beneficial to older adults with AAA by improving vascular function (40). Lowering the cholesterol level was a beneficial method to control aortic aneurysms (41). However, an association between BMI or obesity and aortic aneurysm remains controversial. A Mendelian randomization study showed that BMI did not impact aortic aneurysms (42). But a prospective study showed that BMI was related to a rising risk of incident isolated AAA (43). High BMI or obesity would lead to atherosclerosis and hypertension, which were important incentives for aortic aneurysm (44, 45). Therefore, abstaining from tobacco and drinking, managing hypertension and weight, doing appropriate exercise, dieting in low fat, keeping warm, and living in moderately high altitude may be cost-effective approaches, especially for the aging population.

Some limitations had listed as follows. First, based on GBD 2019, the data about the prevalence, incidence, and subtype of aortic aneurysm were not provided, which partly limits the analysis of the results. Second, the predictions relied largely on the quality of the primitive population-based registry data. A sparsity of data on aortic aneurysms, particularly in low SDI regions, could affect the precision of estimates. However, GBD 2019 study utilized many powerful statistical tools to reduce that impact. Third, as a population epidemiological study, we could not get individual-level data, and our work was inevitably affected by confounding factors when calculating correlation coefficients. However, our results do provide clinical scientists and socio-economists with the latest big data and a more comprehensive analysis of the aortic aneurysm burden.

## Conclusion

Based on GBD 2019, aortic aneurysm, as a public health challenge worldwide, has a decreased ASRD and ASDALYR. Aging men who smoke and women who have hypertension should pay close attention to, particularly in deprived economic groups. A lot of approaches could be performed to reduce the burden of aortic aneurysms. These findings provide valuable insights to formulate increasingly integrated interventions to meet global vascular health challenges.

## Data Availability Statement

The original contributions presented in the study are included in the article/Supplementary Material, further inquiries can be directed to the corresponding author/s.

## Author Contributions

ZW and YY conducted the study, analyzed the data, interpreted the results and drafted the manuscript. ZY, QB, SL, and JY assisted with data analysis. CX and FY critically read the manuscript. XX designed, guided, and revised the manuscript. All authors read and approved the final manuscript.

## Funding

XX was supported by a grant from the National Health Commission, Key Program of Science and Technology of Medical and Health of Zhejiang Province (WKJ-ZJ-2028).

## Conflict of Interest

The authors declare that the research was conducted in the absence of any commercial or financial relationships that could be construed as a potential conflict of interest.

## Publisher's Note

All claims expressed in this article are solely those of the authors and do not necessarily represent those of their affiliated organizations, or those of the publisher, the editors and the reviewers. Any product that may be evaluated in this article, or claim that may be made by its manufacturer, is not guaranteed or endorsed by the publisher.

## Supplementary Material

Supplementary Table 1 Socio-demographic index value for GBD 2019 location. SDI, socio-demographic index; GBD, Global Burden of Disease.

Supplementary Table 2 World bank income level in 2019.

Supplementary Table 3 Global burden of aortic aneurysm in 31 GBD regions in 1990 and 2019. Changes mean increasing times from 1990 to 2019. DALY, disability-adjusted life year rate. SDI, socio-demographic index; GBD, Global Burden of Disease.

Supplementary Table 4 Global burden of aortic aneurysm in 204 countries and territories in 1990 and 2019. Changes mean increasing times from 1990 to 2019. DALY, disability-adjusted life year rate.

Supplementary Table 5 Global burden of aortic aneurysm in different age groups in 2019. DALY, disability-adjusted life year rate. SDI, socio-demographic index.

Supplementary Table 6 Estimated annual percentage changes of aortic aneurism-related age-standardized deaths and DALYs in 31 GBD regions from 1990 to 2019. DALY, disability-adjusted life year rate. SDI, socio-demographic index; EAPC, estimated annual percentage changes; GBD, Global Burden of Disease.

Supplementary Table 7 Estimated annual percentage changes of aortic aneurism-related age-standardized deaths and DALYs in 204 countries and territories from 1990 to 2019. DALY, disability-adjusted life year rate. SDI, socio-demographic index; EAPC, estimated annual percentage changes.

Supplementary Table 8 Estimated annual percentage changes of aortic aneurism-related attributable risks in 31 GBD regions from 1990 to 2019. DALY, disability-adjusted life year rate. SDI, socio-demographic index; EAPC, estimated annual percentage changes; GBD, Global Burden of Disease.

Supplementary Table 9 Population attributable fractions of aortic aneurism-related attributable risks in 31 GBD regions in 2019. DALY, disability-adjusted life year rate. SDI, socio-demographic index; PAF, population attributable fractions; GBD, Global Burden of Disease.

Supplementary Table 10 Correlation analysis of aortic aneurism-related age-standardized deaths and DALYs in 2019, by gender. DALY, disability-adjusted life year rate.

Supplementary Table 11 Correlation analysis of aortic aneurism-related age-standardized deaths and DALYs in 2019. DALY, disability-adjusted life year rate.
