# Supplementary material for: Burden of Aortic Aneurysm and Its Attributable Risk Factors from 1990 to 2019: An Analysis of the Global Burden of Disease Study 2019
Source: Front Cardiovasc Med. 2022 May 31;9:901225. doi: 10.3389/fcvm.2022.901225 (PMC9197430; doi:10.3389/fcvm.2022.901225)
Supplement: Supplementary Table 2 — World bank income level in 2019. [file Data_Sheet_2.PDF]

World Bank Analytical Classifications

(presented in World Development Indicators)

GNI per capita in US$ (Atlas methodology)

Bank's fiscal year:	**FY21**
Data for calendar year :	**2019**
Low income (L)	<= 1, 035
Lower middle income (LM)	036 - 4, 045
Upper middle income (UM)	046 - 12, 535
High income (H)	> 12, 535

Afghanistan	L
Albania	UM
Algeria	LM
American Samoa	UM
Andorra	H
Angola	LM
Antigua and Barbuda	H
Argentina	UM
Armenia	UM
Aruba	H
Australia	H
Austria	H
Azerbaijan	UM
Bahamas, The	H
Bahrain	H
Bangladesh	LM
Barbados	H
Belarus	UM
Belgium	H
Belize	UM
Benin	LM
Bermuda	H
Bhutan	LM
Bolivia	LM
Bosnia and Herzegovina	UM
Botswana	UM
Brazil	UM
British Virgin Islands	H
Brunei Darussalam	H
Bulgaria	UM
Burkina Faso	L
Burundi	L
Cabo Verde	LM
Cambodia	LM
Cameroon	LM
Canada	H
Cayman Islands	H
Central African Republic	L
Chad	L
Channel Islands	H

Chile	H
China	UM
Colombia	UM
Comoros	LM
Congo, Dem. Rep.	L
Congo, Rep.	LM
Costa Rica	UM
Côte d'Ivoire	LM
Croatia	H
Cuba	UM
Curaçao	H
Cyprus	H
Czech Republic	H
Denmark	H
Djibouti	LM
Dominica	UM
Dominican Republic	UM
Ecuador	UM
Egypt, Arab Rep.	LM
El Salvador	LM
Equatorial Guinea	UM
Eritrea	L
Estonia	H
Eswatini	LM
Ethiopia	L
Faeroe Islands	H
Fiji	UM
Finland	H
France	H
French Polynesia	H
Gabon	UM
Gambia, The	L
Georgia	UM
Germany	H
Ghana	LM
Gibraltar	H
Greece	H
Greenland	H
Grenada	UM
Guam	H
Guatemala	UM
Guinea	L
Guinea-Bissau	L
Guyana	UM
Haiti	L
Honduras	LM
Hong Kong SAR, China	H
Hungary	H
Iceland	H
India	LM
Indonesia	UM

Iran, Islamic Rep.	UM
Iraq	UM
Ireland	H
Isle of Man	H
Israel	H
Italy	H
Jamaica	UM
Japan	H
Jordan	UM
Kazakhstan	UM
Kenya	LM
Kiribati	LM
Korea, Dem. Rep.	L
Korea, Rep.	H
Kosovo	UM
Kuwait	H
Kyrgyz Republic	LM
Lao PDR	LM
Latvia	H
Lebanon	UM
Lesotho	LM
Liberia	L
Libya	UM
Liechtenstein	H
Lithuania	H
Luxembourg	H
Macao SAR, China	H
Madagascar	L
Malawi	L
Malaysia	UM
Maldives	UM
Mali	L
Malta	H
Marshall Islands	UM
Mauritania	LM
Mauritius	H
Mexico	UM
Micronesia, Fed. Sts.	LM
Moldova	LM
Monaco	H
Mongolia	LM
Montenegro	UM
Morocco	LM
Mozambique	L
Myanmar	LM
Namibia	UM
Nauru	H
Nepal	LM
Netherlands	H
New Caledonia	H
New Zealand	H

Nicaragua	LM
Niger	L
Nigeria	LM
North Macedonia	UM
Northern Mariana Islands	H
Norway	H
Oman	H
Pakistan	LM
Palau	H
Panama	H
Papua New Guinea	LM
Paraguay	UM
Peru	UM
Philippines	LM
Poland	H
Portugal	H
Puerto Rico	H
Qatar	H
Romania	H
Russian Federation	UM
Rwanda	L
Samoa	UM
San Marino	H
São Tomé and Principe	LM
Saudi Arabia	H
Senegal	LM
Serbia	UM
Seychelles	H
Sierra Leone	L
Singapore	H
Sint Maarten (Dutch part)	H
Slovak Republic	H
Slovenia	H
Solomon Islands	LM
Somalia	L
South Africa	UM
South Sudan	L
Spain	H
Sri Lanka	LM
St. Kitts and Nevis	H
St. Lucia	UM
St. Martin (French part)	H
St. Vincent and the Grenadines	UM
Sudan	L
Suriname	UM
Sweden	H
Switzerland	H
Syrian Arab Republic	L
Taiwan, China	H
Tajikistan	L
Tanzania	LM

Thailand	UM
Timor-Leste	LM
Togo	L
Tonga	UM
Trinidad and Tobago	H
Tunisia	LM
Turkey	UM
Turkmenistan	UM
Turks and Caicos Islands	H
Tuvalu	UM
Uganda	L
Ukraine	LM
United Arab Emirates	H
United Kingdom	H
United States	H
Uruguay	H
Uzbekistan	LM
Vanuatu	LM
Venezuela, RB	UM
Vietnam	LM
Virgin Islands (U.S.)	H
West Bank and Gaza	LM
Yemen, Rep.	L
Zambia	LM
Zimbabwe	LM

* At this time, there were Yemen, PDR (L) and Yemen, Arab Rep. (LM); combined they would have been

Czechoslovakia (former)
Mayotte
Netherlands Antilles (former)
Serbia and Montenegro (former)
USSR (former)
Yugoslavia (former)

Note: Income classifications are set each year on July 1 for all World Bank member economies, and all other economies with populations of more than 30,000. These official analytical classifications are fixed during the World Bank's fiscal year (ending on June 30), thus economies remain in the categories in which they are classified irrespective of any revisions to their per capita income data. The historical classifications shown are as published on July 1 of each fiscal year.

LM.
