# Supplementary material for: Burden of Aortic Aneurysm and Its Attributable Risk Factors from 1990 to 2019: An Analysis of the Global Burden of Disease Study 2019
Source: Front Cardiovasc Med. 2022 May 31;9:901225. doi: 10.3389/fcvm.2022.901225 (PMC9197430; doi:10.3389/fcvm.2022.901225)
Supplement: Supplementary Table 3 — Global burden of aortic aneurysm in 31 GBD regions in 1990 and 2019. Changes mean increasing times from 1990 to 2019. DALY, disability-adjusted life year rate. SDI, socio-demographic index; GBD, Global Burden of Disease. [file Data_Sheet_3.PDF]

measure	location	sex	age	cause	metric	year	val	upper	lower	Changes(1990-2019)
DALYs	Andean Latin America	Both	All Ages	Aortic aneurysm	Number	1990	6686.07772	7775.106215	5581.326899	
DALYs	Andean Latin America	Both	All Ages	Aortic aneurysm	Number	2019	15801.8096	19135.28965	12965.0898	136.34%
DALYs	Andean Latin America	Both	Age-standardized	Aortic aneurysm	Rate	1990	30.7202695	35.60379489	25.78256116	
DALYs	Andean Latin America	Both	Age-standardized	Aortic aneurysm	Rate	2019	27.8703229	33.7091539	22.87872026	-9.28%
Deaths	Andean Latin America	Both	All Ages	Aortic aneurysm	Number	1990	287.56912	330.4975806	244.9768174	
Deaths	Andean Latin America	Both	All Ages	Aortic aneurysm	Number	2019	780.921808	934.6367074	641.5569959	171.56%
Deaths	Andean Latin America	Both	Age-standardized	Aortic aneurysm	Rate	1990	1.52566538	1.740796625	1.311781082	
Deaths	Andean Latin America	Both	Age-standardized	Aortic aneurysm	Rate	2019	1.44106366	1.723011919	1.184711906	-5.55%
DALYs	Andean Latin America	Female	All Ages	Aortic aneurysm	Number	1990	2299.82787	2873.168369	1959.055998	
DALYs	Andean Latin America	Female	All Ages	Aortic aneurysm	Number	2019	5792.60205	7082.594822	4631.44501	151.87%
DALYs	Andean Latin America	Female	Age-standardized	Aortic aneurysm	Rate	1990	20.5976406	25.50873874	17.51960048	
DALYs	Andean Latin America	Female	Age-standardized	Aortic aneurysm	Rate	2019	19.6503917	23.97756371	15.76431164	-4.60%
Deaths	Andean Latin America	Female	All Ages	Aortic aneurysm	Number	1990	103.446877	126.9092869	87.78437979	
Deaths	Andean Latin America	Female	All Ages	Aortic aneurysm	Number	2019	296.40677	357.5175761	242.0771616	186.53%
Deaths	Andean Latin America	Female	Age-standardized	Aortic aneurysm	Rate	1990	1.07990953	1.32316195	0.913760889	
Deaths	Andean Latin America	Female	Age-standardized	Aortic aneurysm	Rate	2019	1.03473676	1.248141993	0.845757875	-4.18%
DALYs	Andean Latin America	Male	All Ages	Aortic aneurysm	Number	1990	4386.24985	5361.161453	3359.526902	
DALYs	Andean Latin America	Male	All Ages	Aortic aneurysm	Number	2019	10009.2075	12179.13306	8092.049963	128.20%
DALYs	Andean Latin America	Male	Age-standardized	Aortic aneurysm	Rate	1990	41.4307978	50.73532566	32.14104319	
DALYs	Andean Latin America	Male	Age-standardized	Aortic aneurysm	Rate	2019	36.7702869	44.71092032	29.61183141	-11.25%
Deaths	Andean Latin America	Male	All Ages	Aortic aneurysm	Number	1990	184.122244	223.4009295	145.4456673	
Deaths	Andean Latin America	Male	All Ages	Aortic aneurysm	Number	2019	484.515037	580.9592284	392.9048338	163.15%
Deaths	Andean Latin America	Male	Age-standardized	Aortic aneurysm	Rate	1990	2.00539156	2.424117157	1.591679654	
Deaths	Andean Latin America	Male	Age-standardized	Aortic aneurysm	Rate	2019	1.89392301	2.269721619	1.537021367	-5.56%
DALYs	Australasia	Both	All Ages	Aortic aneurysm	Number	1990	32379.9786	33841.6044	30841.52967	
DALYs	Australasia	Both	All Ages	Aortic aneurysm	Number	2019	26788.0735	29065.8869	23910.71454	-17.27%
DALYs	Australasia	Both	Age-standardized	Aortic aneurysm	Rate	1990	134.990326	141.1481966	128.5211494	
DALYs	Australasia	Both	Age-standardized	Aortic aneurysm	Rate	2019	53.4938181	57.7073221	48.09956371	-60.37%
Deaths	Australasia	Both	All Ages	Aortic aneurysm	Number	1990	1817.24194	1904.95625	1702.031275	
Deaths	Australasia	Both	All Ages	Aortic aneurysm	Number	2019	1801.88807	1979.248777	1567.548108	-0.84%
Deaths	Australasia	Both	Age-standardized	Aortic aneurysm	Rate	1990	7.69222517	8.065138388	7.19306366	
Deaths	Australasia	Both	Age-standardized	Aortic aneurysm	Rate	2019	3.31292256	3.621200895	2.903548544	-56.93%
DALYs	Australasia	Female	All Ages	Aortic aneurysm	Number	1990	9991.59873	10561.83181	9322.074676	
DALYs	Australasia	Female	All Ages	Aortic aneurysm	Number	2019	10011.6529	11177.79578	8550.842439	0.20%
DALYs	Australasia	Female	Age-standardized	Aortic aneurysm	Rate	1990	73.7055743	77.89196232	69.06424743	
DALYs	Australasia	Female	Age-standardized	Aortic aneurysm	Rate	2019	35.8907001	39.98310591	31.13921399	-51.31%
Deaths	Australasia	Female	All Ages	Aortic aneurysm	Number	1990	630.312327	673.8973702	573.0918781	
Deaths	Australasia	Female	All Ages	Aortic aneurysm	Number	2019	747.737371	845.9466345	611.9574804	18.63%
Deaths	Australasia	Female	Age-standardized	Aortic aneurysm	Rate	1990	4.53385231	4.846457837	4.123920595	
Deaths	Australasia	Female	Age-standardized	Aortic aneurysm	Rate	2019	2.3926134	2.699271366	1.990990305	-47.23%
DALYs	Australasia	Male	All Ages	Aortic aneurysm	Number	1990	22388.3798	23430.99596	21281.36506	
DALYs	Australasia	Male	All Ages	Aortic aneurysm	Number	2019	16776.4205	18238.57807	15105.6269	-25.07%
DALYs	Australasia	Male	Age-standardized	Aortic aneurysm	Rate	1990	212.671531	222.3985817	202.1353883	
DALYs	Australasia	Male	Age-standardized	Aortic aneurysm	Rate	2019	73.2944989	79.56987241	66.26942877	-65.54%
Deaths	Australasia	Male	All Ages	Aortic aneurysm	Number	1990	1186.92961	1247.146578	1120.567911	
Deaths	Australasia	Male	All Ages	Aortic aneurysm	Number	2019	1054.1507	1153.452645	935.1993219	-11.19%
Deaths	Australasia	Male	Age-standardized	Aortic aneurysm	Rate	1990	12.1858066	12.81344204	11.4282176	
Deaths	Australasia	Male	Age-standardized	Aortic aneurysm	Rate	2019	4.42556297	4.832475032	3.928639741	-63.68%
DALYs	Caribbean	Both	All Ages	Aortic aneurysm	Number	1990	15974.7522	17488.68497	14635.81342	
DALYs	Caribbean	Both	All Ages	Aortic aneurysm	Number	2019	29526.2766	34467.71338	24639.95017	84.83%
DALYs	Caribbean	Both	Age-standardized	Aortic aneurysm	Rate	1990	61.0957366	66.86429905	55.87362232	
DALYs	Caribbean	Both	Age-standardized	Aortic aneurysm	Rate	2019	57.2856568	66.7732812	47.83585047	-6.24%
Deaths	Caribbean	Both	All Ages	Aortic aneurysm	Number	1990	812.76475	891.7212868	738.6381672	
Deaths	Caribbean	Both	All Ages	Aortic aneurysm	Number	2019	1571.77667	1824.159567	1333.417826	93.39%
Deaths	Caribbean	Both	Age-standardized	Aortic aneurysm	Rate	1990	3.28469312	3.606182721	2.982153997	
Deaths	Caribbean	Both	Age-standardized	Aortic aneurysm	Rate	2019	3.04430177	3.536385383	2.582054174	-7.32%
DALYs	Caribbean	Female	All Ages	Aortic aneurysm	Number	1990	4699.11563	5533.895845	4269.179777	
DALYs	Caribbean	Female	All Ages	Aortic aneurysm	Number	2019	9101.71038	11096.81703	7665.355687	93.69%
DALYs	Caribbean	Female	Age-standardized	Aortic aneurysm	Rate	1990	34.757184	40.66207991	31.61130748	
DALYs	Caribbean	Female	Age-standardized	Aortic aneurysm	Rate	2019	33.0518077	40.33631482	27.83081517	-4.91%
Deaths	Caribbean	Female	All Ages	Aortic aneurysm	Number	1990	249.248426	282.7930183	226.0101773	
Deaths	Caribbean	Female	All Ages	Aortic aneurysm	Number	2019	521.111065	616.8417457	442.9465127	109.07%
Deaths	Caribbean	Female	Age-standardized	Aortic aneurysm	Rate	1990	1.93969857	2.190372061	1.756386268	
Deaths	Caribbean	Female	Age-standardized	Aortic aneurysm	Rate	2019	1.83690217	2.174989257	1.56223496	-5.30%
DALYs	Caribbean	Male	All Ages	Aortic aneurysm	Number	1990	11275.6365	12764.29841	9919.418807	
DALYs	Caribbean	Male	All Ages	Aortic aneurysm	Number	2019	20424.5662	23959.08349	16802.13119	81.14%
DALYs	Caribbean	Male	Age-standardized	Aortic aneurysm	Rate	1990	89.6559164	101.2314705	78.73293888	
DALYs	Caribbean	Male	Age-standardized	Aortic aneurysm	Rate	2019	84.7277107	99.26536957	69.79858831	-5.50%
Deaths	Caribbean	Male	All Ages	Aortic aneurysm	Number	1990	563.516324	635.0009684	494.7662799	
Deaths	Caribbean	Male	All Ages	Aortic aneurysm	Number	2019	1050.66561	1228.085255	878.0896375	86.45%
Deaths	Caribbean	Male	Age-standardized	Aortic aneurysm	Rate	1990	4.78487156	5.384551714	4.208766469	
Deaths	Caribbean	Male	Age-standardized	Aortic aneurysm	Rate	2019	4.47398702	5.233857809	3.743191554	-6.50%
DALYs	Central Asia	Both	All Ages	Aortic aneurysm	Number	1990	13174.4551	15117.19611	12033.00306	
DALYs	Central Asia	Both	All Ages	Aortic aneurysm	Number	2019	34421.8834	37882.11285	31200.21523	161.28%
DALYs	Central Asia	Both	Age-standardized	Aortic aneurysm	Rate	1990	27.3229748	31.47565906	24.91594291	
DALYs	Central Asia	Both	Age-standardized	Aortic aneurysm	Rate	2019	46.5930544	51.05043777	42.34722981	70.53%
Deaths	Central Asia	Both	All Ages	Aortic aneurysm	Number	1990	547.104828	633.5832983	491.7451867	
Deaths	Central Asia	Both	All Ages	Aortic aneurysm	Number	2019	1437.56694	1577.517341	1308.955056	162.76%
Deaths	Central Asia	Both	Age-standardized	Aortic aneurysm	Rate	1990	1.25195428	1.461185255	1.101703805	
Deaths	Central Asia	Both	Age-standardized	Aortic aneurysm	Rate	2019	2.32721898	2.548277887	2.120523803	85.89%
DALYs	Central Asia	Female	All Ages	Aortic aneurysm	Number	1990	4633.62196	5069.729719	4031.3312	
DALYs	Central Asia	Female	All Ages	Aortic aneurysm	Number	2019	10862.9287	12102.47346	9716.806226	134.44%
DALYs	Central Asia	Female	Age-standardized	Aortic aneurysm	Rate	1990	16.7534429	18.32576584	14.45719506	
DALYs	Central Asia	Female	Age-standardized	Aortic aneurysm	Rate	2019	27.1511786	30.04844591	24.39117122	62.06%
Deaths	Central Asia	Female	All Ages	Aortic aneurysm	Number	1990	224.577333	247.6430949	181.8009169	
Deaths	Central Asia	Female	All Ages	Aortic aneurysm	Number	2019	515.102547	569.9988524	462.1371226	129.37%
Deaths	Central Asia	Female	Age-standardized	Aortic aneurysm	Rate	1990	0.85111116	0.939651894	0.678810666	
Deaths	Central Asia	Female	Age-standardized	Aortic aneurysm	Rate	2019	1.48263638	1.639113287	1.326019655	74.20%
DALYs	Central Asia	Male	All Ages	Aortic aneurysm	Number	1990	8540.83316	10383.36911	7517.661522	
DALYs	Central Asia	Male	All Ages	Aortic aneurysm	Number	2019	23558.9548	25984.69386	21179.64221	175.84%
DALYs	Central Asia	Male	Age-standardized	Aortic aneurysm	Rate	1990	42.0908914	52.14416308	36.79886181	
DALYs	Central Asia	Male	Age-standardized	Aortic aneurysm	Rate	2019	72.5639151	79.80689835	65.64882565	72.40%
Deaths	Central Asia	Male	All Ages	Aortic aneurysm	Number	1990	322.527495	402.5505466	280.1978002	
Deaths	Central Asia	Male	All Ages	Aortic aneurysm	Number	2019	922.464394	1013.294802	833.3671373	186.01%
Deaths	Central Asia	Male	Age-standardized	Aortic aneurysm	Rate	1990	1.88855479	2.417218539	1.612194009	
Deaths	Central Asia	Male	Age-standardized	Aortic aneurysm	Rate	2019	3.60010361	3.94091963	3.267605004	90.63%
DALYs	Central Europe	Both	All Ages	Aortic aneurysm	Number	1990	71673.2306	74482.85397	68710.80617	
DALYs	Central Europe	Both	All Ages	Aortic aneurysm	Number	2019	129958.083	147341.7917	113557.8613	81.32%
DALYs	Central Europe	Both	Age-standardized	Aortic aneurysm	Rate	1990	49.3125853	51.19250286	47.28743876	
DALYs	Central Europe	Both	Age-standardized	Aortic aneurysm	Rate	2019	65.5662864	74.41763429	57.23623637	32.96%

Deaths	Central Europe	Both	All Ages	Aortic aneurysm	Number	1990	2971.62257	3086.515265	2832.643133	
Deaths	Central Europe	Both	All Ages	Aortic aneurysm	Number	2019	6486.78266	7321.79623	5706.900235	118.29%
Deaths	Central Europe	Both	Age-standardized	Aortic aneurysm	Rate	1990	2.09357139	2.174657112	1.987419715	
Deaths	Central Europe	Both	Age-standardized	Aortic aneurysm	Rate	2019	3.00824387	3.403915785	2.638013466	43.69%
DALYs	Central Europe	Female	All Ages	Aortic aneurysm	Number	1990	20925.3384	22051.50555	19970.35804	
DALYs	Central Europe	Female	All Ages	Aortic aneurysm	Number	2019	39104.326	44837.29272	34042.15908	86.88%
DALYs	Central Europe	Female	Age-standardized	Aortic aneurysm	Rate	1990	25.8834563	27.26880097	24.70533371	
DALYs	Central Europe	Female	Age-standardized	Aortic aneurysm	Rate	2019	33.9502013	39.14609569	29.43298346	31.17%
Deaths	Central Europe	Female	All Ages	Aortic aneurysm	Number	1990	982.822891	1035.999123	927.0818219	
Deaths	Central Europe	Female	All Ages	Aortic aneurysm	Number	2019	2274.66122	2603.441699	1961.839087	131.44%
Deaths	Central Europe	Female	Age-standardized	Aortic aneurysm	Rate	1990	1.2040003	1.271302507	1.131078547	
Deaths	Central Europe	Female	Age-standardized	Aortic aneurysm	Rate	2019	1.72084388	1.970925197	1.490982181	42.93%
DALYs	Central Europe	Male	All Ages	Aortic aneurysm	Number	1990	50747.8922	53169.79181	48397.93062	
DALYs	Central Europe	Male	All Ages	Aortic aneurysm	Number	2019	90853.7567	104469.7375	78502.00283	79.03%
DALYs	Central Europe	Male	Age-standardized	Aortic aneurysm	Rate	1990	78.2253907	81.88238827	74.48838448	
DALYs	Central Europe	Male	Age-standardized	Aortic aneurysm	Rate	2019	103.487912	119.0426748	89.68426467	32.29%
Deaths	Central Europe	Male	All Ages	Aortic aneurysm	Number	1990	1988.79968	2080.481286	1886.945117	
Deaths	Central Europe	Male	All Ages	Aortic aneurysm	Number	2019	4212.12144	4820.563621	3657.44828	111.79%
Deaths	Central Europe	Male	Age-standardized	Aortic aneurysm	Rate	1990	3.31069679	3.463623434	3.132461798	
Deaths	Central Europe	Male	Age-standardized	Aortic aneurysm	Rate	2019	4.73617121	5.416401435	4.119449526	43.06%
DALYs	Central Latin America	Both	All Ages	Aortic aneurysm	Number	1990	30369.9608	31637.1617	29254.36766	
DALYs	Central Latin America	Both	All Ages	Aortic aneurysm	Number	2019	75978.3375	91762.30923	62392.00173	150.18%
DALYs	Central Latin America	Both	Age-standardized	Aortic aneurysm	Rate	1990	34.2592456	35.73463993	32.81376689	
DALYs	Central Latin America	Both	Age-standardized	Aortic aneurysm	Rate	2019	32.1922137	38.78517903	26.56822741	-6.03%
Deaths	Central Latin America	Both	All Ages	Aortic aneurysm	Number	1990	1264.93536	1319.296442	1200.295772	
Deaths	Central Latin America	Both	All Ages	Aortic aneurysm	Number	2019	3739.69787	4455.119212	3094.203037	195.64%
Deaths	Central Latin America	Both	Age-standardized	Aortic aneurysm	Rate	1990	1.64555192	1.722467986	1.544483438	
Deaths	Central Latin America	Both	Age-standardized	Aortic aneurysm	Rate	2019	1.64926719	1.961658943	1.360420443	0.23%
DALYs	Central Latin America	Female	All Ages	Aortic aneurysm	Number	1990	8114.11644	8529.153839	7743.491213	
DALYs	Central Latin America	Female	All Ages	Aortic aneurysm	Number	2019	21314.8128	25347.39541	17628.65776	162.69%
DALYs	Central Latin America	Female	Age-standardized	Aortic aneurysm	Rate	1990	17.5079886	18.47709331	16.61378493	
DALYs	Central Latin America	Female	Age-standardized	Aortic aneurysm	Rate	2019	16.830902	20.00630784	13.93195562	-3.87%
Deaths	Central Latin America	Female	All Ages	Aortic aneurysm	Number	1990	345.713084	366.8310546	323.7943534	
Deaths	Central Latin America	Female	All Ages	Aortic aneurysm	Number	2019	1122.38227	1327.879844	914.3695535	224.66%
Deaths	Central Latin America	Female	Age-standardized	Aortic aneurysm	Rate	1990	0.87091438	0.927640016	0.804585747	
Deaths	Central Latin America	Female	Age-standardized	Aortic aneurysm	Rate	2019	0.90748174	1.074367059	0.738170882	4.20%
DALYs	Central Latin America	Male	All Ages	Aortic aneurysm	Number	1990	22255.8444	23260.47325	21349.10573	
DALYs	Central Latin America	Male	All Ages	Aortic aneurysm	Number	2019	54663.5247	66505.74755	44371.00709	145.61%
DALYs	Central Latin America	Male	Age-standardized	Aortic aneurysm	Rate	1990	52.3120463	54.81136188	49.97778799	
DALYs	Central Latin America	Male	Age-standardized	Aortic aneurysm	Rate	2019	50.1026195	60.71948501	40.83107485	-4.22%
Deaths	Central Latin America	Male	All Ages	Aortic aneurysm	Number	1990	919.222275	964.2206548	873.9168665	
Deaths	Central Latin America	Male	All Ages	Aortic aneurysm	Number	2019	2617.3156	3149.665148	2143.844159	184.73%
Deaths	Central Latin America	Male	Age-standardized	Aortic aneurysm	Rate	1990	2.49137781	2.625685361	2.344096422	
Deaths	Central Latin America	Male	Age-standardized	Aortic aneurysm	Rate	2019	2.54230918	3.053470602	2.089538586	2.04%
DALYs	Central Sub-Saharan Africa	Both	All Ages	Aortic aneurysm	Number	1990	16929.5548	23690.12915	11116.89773	
DALYs	Central Sub-Saharan Africa	Both	All Ages	Aortic aneurysm	Number	2019	30728.2398	40888.20062	22736.59817	81.51%
DALYs	Central Sub-Saharan Africa	Both	Age-standardized	Aortic aneurysm	Rate	1990	73.2465383	100.1572157	48.13449349	
DALYs	Central Sub-Saharan Africa	Both	Age-standardized	Aortic aneurysm	Rate	2019	55.7308764	72.71309716	41.33270653	-23.91%
Deaths	Central Sub-Saharan Africa	Both	All Ages	Aortic aneurysm	Number	1990	656.656253	900.0967111	429.8643602	
Deaths	Central Sub-Saharan Africa	Both	All Ages	Aortic aneurysm	Number	2019	1195.96731	1558.312461	885.8079051	82.13%
Deaths	Central Sub-Saharan Africa	Both	Age-standardized	Aortic aneurysm	Rate	1990	3.57155197	4.770604778	2.321225785	
Deaths	Central Sub-Saharan Africa	Both	Age-standardized	Aortic aneurysm	Rate	2019	2.72572842	3.467461585	2.029685584	-23.68%
DALYs	Central Sub-Saharan Africa	Female	All Ages	Aortic aneurysm	Number	1990	5407.38754	7823.06847	3569.974691	
DALYs	Central Sub-Saharan Africa	Female	All Ages	Aortic aneurysm	Number	2019	10512.9429	13851.75276	7414.093953	94.42%
DALYs	Central Sub-Saharan Africa	Female	Age-standardized	Aortic aneurysm	Rate	1990	47.1721157	65.27187064	31.57249658	
DALYs	Central Sub-Saharan Africa	Female	Age-standardized	Aortic aneurysm	Rate	2019	37.0313681	48.5013774	26.09670058	-21.50%
Deaths	Central Sub-Saharan Africa	Female	All Ages	Aortic aneurysm	Number	1990	220.915859	305.8903054	146.6173818	
Deaths	Central Sub-Saharan Africa	Female	All Ages	Aortic aneurysm	Number	2019	458.721238	599.4976019	326.7120354	107.65%
Deaths	Central Sub-Saharan Africa	Female	Age-standardized	Aortic aneurysm	Rate	1990	2.49327418	3.293969086	1.693211023	
Deaths	Central Sub-Saharan Africa	Female	Age-standardized	Aortic aneurysm	Rate	2019	1.9625975	2.56573687	1.381841594	-21.28%
DALYs	Central Sub-Saharan Africa	Male	All Ages	Aortic aneurysm	Number	1990	11522.1673	17727.12578	6785.48697	
DALYs	Central Sub-Saharan Africa	Male	All Ages	Aortic aneurysm	Number	2019	20215.2969	28065.10206	14162.45873	75.45%
DALYs	Central Sub-Saharan Africa	Male	Age-standardized	Aortic aneurysm	Rate	1990	102.602389	155.1738293	59.80222795	
DALYs	Central Sub-Saharan Africa	Male	Age-standardized	Aortic aneurysm	Rate	2019	78.6281243	107.3081725	55.61907964	-23.37%
Deaths	Central Sub-Saharan Africa	Male	All Ages	Aortic aneurysm	Number	1990	435.740394	657.4242688	254.5928828	
Deaths	Central Sub-Saharan Africa	Male	All Ages	Aortic aneurysm	Number	2019	737.24607	1008.021219	521.3692762	69.19%
Deaths	Central Sub-Saharan Africa	Male	Age-standardized	Aortic aneurysm	Rate	1990	4.7853962	6.964393293	2.735648868	
Deaths	Central Sub-Saharan Africa	Male	Age-standardized	Aortic aneurysm	Rate	2019	3.7477824	4.994499054	2.621138777	-21.68%
DALYs	East Asia	Both	All Ages	Aortic aneurysm	Number	1990	196241.706	260617.5132	154791.9069	
DALYs	East Asia	Both	All Ages	Aortic aneurysm	Number	2019	410456.94	482386.9361	349417.1172	109.16%
DALYs	East Asia	Both	Age-standardized	Aortic aneurysm	Rate	1990	21.3438228	28.03183072	17.08224178	
DALYs	East Asia	Both	Age-standardized	Aortic aneurysm	Rate	2019	20.3985917	23.89474392	17.42439113	-4.43%
Deaths	East Asia	Both	All Ages	Aortic aneurysm	Number	1990	7574.68881	9889.410262	6102.781895	
Deaths	East Asia	Both	All Ages	Aortic aneurysm	Number	2019	18515.0674	21435.61044	15850.9202	144.43%
Deaths	East Asia	Both	Age-standardized	Aortic aneurysm	Rate	1990	1.00887051	1.303612683	0.827920084	
Deaths	East Asia	Both	Age-standardized	Aortic aneurysm	Rate	2019	0.97773114	1.121823362	0.841163946	-3.09%
DALYs	East Asia	Female	All Ages	Aortic aneurysm	Number	1990	57387.5706	91444.5526	44994.29609	
DALYs	East Asia	Female	All Ages	Aortic aneurysm	Number	2019	107826.982	130361.5055	87397.48703	87.89%
DALYs	East Asia	Female	Age-standardized	Aortic aneurysm	Rate	1990	12.6357074	19.50796353	10.06231888	
DALYs	East Asia	Female	Age-standardized	Aortic aneurysm	Rate	2019	10.3541216	12.50309949	8.390196196	-18.06%
Deaths	East Asia	Female	All Ages	Aortic aneurysm	Number	1990	2442.53433	3696.755654	1956.080624	
Deaths	East Asia	Female	All Ages	Aortic aneurysm	Number	2019	5451.24696	6464.360715	4505.496742	123.18%
Deaths	East Asia	Female	Age-standardized	Aortic aneurysm	Rate	1990	0.64298876	0.937430607	0.524325424	
Deaths	East Asia	Female	Age-standardized	Aortic aneurysm	Rate	2019	0.53912586	0.637578301	0.444677507	-16.15%
DALYs	East Asia	Male	All Ages	Aortic aneurysm	Number	1990	138854.136	192100.7242	100535.0613	
DALYs	East Asia	Male	All Ages	Aortic aneurysm	Number	2019	302629.958	374727.474	244033.0033	117.95%
DALYs	East Asia	Male	Age-standardized	Aortic aneurysm	Rate	1990	30.9791029	42.09607163	22.6508798	
DALYs	East Asia	Male	Age-standardized	Aortic aneurysm	Rate	2019	31.5544322	38.75809572	25.70740066	1.86%
Deaths	East Asia	Male	All Ages	Aortic aneurysm	Number	1990	5132.15447	7000.225695	3750.565542	
Deaths	East Asia	Male	All Ages	Aortic aneurysm	Number	2019	13063.8204	15937.23885	10688.40926	154.55%
Deaths	East Asia	Male	Age-standardized	Aortic aneurysm	Rate	1990	1.48955651	1.978068723	1.091886992	
Deaths	East Asia	Male	Age-standardized	Aortic aneurysm	Rate	2019	1.55400886	1.858252131	1.286338093	4.33%
DALYs	Eastern Europe	Both	All Ages	Aortic aneurysm	Number	1990	152844.309	167391.018	141103.1757	
DALYs	Eastern Europe	Both	All Ages	Aortic aneurysm	Number	2019	269010.594	304922.7414	238881.2678	76.00%
DALYs	Eastern Europe	Both	Age-standardized	Aortic aneurysm	Rate	1990	55.057791	60.41847168	50.75452674	
DALYs	Eastern Europe	Both	Age-standardized	Aortic aneurysm	Rate	2019	82.8343367	93.98866707	73.45164365	50.45%
Deaths	Eastern Europe	Both	All Ages	Aortic aneurysm	Number	1990	6267.78742	6763.151594	5889.736416	
Deaths	Eastern Europe	Both	All Ages	Aortic aneurysm	Number	2019	11944.7479	13391.84902	10697.68846	90.57%
Deaths	Eastern Europe	Both	Age-standardized	Aortic aneurysm	Rate	1990	2.3011223	2.482965327	2.161353452	
Deaths	Eastern Europe	Both	Age-standardized	Aortic aneurysm	Rate	2019	3.49628516	3.929260475	3.12963079	51.94%
DALYs	Eastern Europe	Female	All Ages	Aortic aneurysm	Number	1990	53371.9582	56401.39213	48423.87747	

DALYs	Eastern Europe	Female	All Ages	Aortic aneurysm	Number	2019	82741.6227	96619.65325	70159.06215	55.03%
DALYs	Eastern Europe	Female	Age-standardized	Aortic aneurysm	Rate	1990	30.8237546	32.67315405	27.57204736	
DALYs	Eastern Europe	Female	Age-standardized	Aortic aneurysm	Rate	2019	40.8936582	48.26991347	34.47340886	32.67%
Deaths	Eastern Europe	Female	All Ages	Aortic aneurysm	Number	1990	2492.49813	2635.738424	2307.077109	
Deaths	Eastern Europe	Female	All Ages	Aortic aneurysm	Number	2019	4411.02925	5090.772202	3757.093284	76.97%
Deaths	Eastern Europe	Female	Age-standardized	Aortic aneurysm	Rate	1990	1.38884741	1.46811878	1.276245841	
Deaths	Eastern Europe	Female	Age-standardized	Aortic aneurysm	Rate	2019	1.94832663	2.255771578	1.662207957	40.28%
DALYs	Eastern Europe	Male	All Ages	Aortic aneurysm	Number	1990	99472.3504	113687.7986	88056.43865	
DALYs	Eastern Europe	Male	All Ages	Aortic aneurysm	Number	2019	186268.971	219720.8256	157731.1949	87.26%
DALYs	Eastern Europe	Male	Age-standardized	Aortic aneurysm	Rate	1990	95.6938352	108.4308947	85.50220618	
DALYs	Eastern Europe	Male	Age-standardized	Aortic aneurysm	Rate	2019	142.456039	167.8843109	121.0214998	48.87%
Deaths	Eastern Europe	Male	All Ages	Aortic aneurysm	Number	1990	3775.28929	4235.583279	3400.917324	
Deaths	Eastern Europe	Male	All Ages	Aortic aneurysm	Number	2019	7533.71869	8795.088584	6434.674317	99.55%
Deaths	Eastern Europe	Male	Age-standardized	Aortic aneurysm	Rate	1990	4.15258092	4.613514302	3.789895758	
Deaths	Eastern Europe	Male	Age-standardized	Aortic aneurysm	Rate	2019	6.01489266	6.990490659	5.142966961	44.85%
DALYs	Eastern Sub-Saharan Africa	Both	All Ages	Aortic aneurysm	Number	1990	48664.5261	70259.48932	27157.6449	
DALYs	Eastern Sub-Saharan Africa	Both	All Ages	Aortic aneurysm	Number	2019	78929.2298	100382.4941	56500.44928	62.19%
DALYs	Eastern Sub-Saharan Africa	Both	Age-standardized	Aortic aneurysm	Rate	1990	61.9459163	86.6405926	34.58635005	
DALYs	Eastern Sub-Saharan Africa	Both	Age-standardized	Aortic aneurysm	Rate	2019	45.2642877	56.50711661	32.28217977	-26.93%
Deaths	Eastern Sub-Saharan Africa	Both	All Ages	Aortic aneurysm	Number	1990	1906.62114	2653.562157	1063.384019	
Deaths	Eastern Sub-Saharan Africa	Both	All Ages	Aortic aneurysm	Number	2019	3131.92695	3893.741203	2226.109509	64.27%
Deaths	Eastern Sub-Saharan Africa	Both	Age-standardized	Aortic aneurysm	Rate	1990	3.02836391	4.081339321	1.704410508	
Deaths	Eastern Sub-Saharan Africa	Both	Age-standardized	Aortic aneurysm	Rate	2019	2.27331083	2.79049982	1.612583185	-24.93%
DALYs	Eastern Sub-Saharan Africa	Female	All Ages	Aortic aneurysm	Number	1990	16520.4867	24713.72332	8642.879932	
DALYs	Eastern Sub-Saharan Africa	Female	All Ages	Aortic aneurysm	Number	2019	28165.2119	35285.65526	19597.2615	70.49%
DALYs	Eastern Sub-Saharan Africa	Female	Age-standardized	Aortic aneurysm	Rate	1990	44.1788607	60.93094793	23.48625953	
DALYs	Eastern Sub-Saharan Africa	Female	Age-standardized	Aortic aneurysm	Rate	2019	32.7620771	41.05646357	22.74554078	-25.84%
Deaths	Eastern Sub-Saharan Africa	Female	All Ages	Aortic aneurysm	Number	1990	704.38007	954.5882458	375.9812266	
Deaths	Eastern Sub-Saharan Africa	Female	All Ages	Aortic aneurysm	Number	2019	1248.56136	1565.083779	866.4770816	77.26%
Deaths	Eastern Sub-Saharan Africa	Female	Age-standardized	Aortic aneurysm	Rate	1990	2.37837097	3.090007777	1.304232451	
Deaths	Eastern Sub-Saharan Africa	Female	Age-standardized	Aortic aneurysm	Rate	2019	1.80131613	2.270181437	1.234591877	-24.26%
DALYs	Eastern Sub-Saharan Africa	Male	All Ages	Aortic aneurysm	Number	1990	32144.0394	53249.16991	17141.97697	
DALYs	Eastern Sub-Saharan Africa	Male	All Ages	Aortic aneurysm	Number	2019	50764.0179	68448.81298	33362.48756	57.93%
DALYs	Eastern Sub-Saharan Africa	Male	Age-standardized	Aortic aneurysm	Rate	1990	79.7627299	128.988743	42.63103781	
DALYs	Eastern Sub-Saharan Africa	Male	Age-standardized	Aortic aneurysm	Rate	2019	58.7031387	79.27529226	38.60214661	-26.40%
Deaths	Eastern Sub-Saharan Africa	Male	All Ages	Aortic aneurysm	Number	1990	1202.24107	1936.968977	640.5349915	
Deaths	Eastern Sub-Saharan Africa	Male	All Ages	Aortic aneurysm	Number	2019	1883.36558	2536.849299	1238.723235	56.65%
Deaths	Eastern Sub-Saharan Africa	Male	Age-standardized	Aortic aneurysm	Rate	1990	3.66999054	5.758551498	1.938847133	
Deaths	Eastern Sub-Saharan Africa	Male	Age-standardized	Aortic aneurysm	Rate	2019	2.7911718	3.814455152	1.823621914	-23.95%
DALYs	Global	Both	All Ages	Aortic aneurysm	Number	1990	1989613.52	2192796.327	1819554.195	
DALYs	Global	Both	All Ages	Aortic aneurysm	Number	2019	3322343.13	3524925.217	3107724.62	66.98%
DALYs	Global	Both	Age-standardized	Aortic aneurysm	Rate	1990	50.7908937	55.65605292	46.5002184	
DALYs	Global	Both	Age-standardized	Aortic aneurysm	Rate	2019	40.9417354	43.43206539	38.19519506	-19.39%
Deaths	Global	Both	All Ages	Aortic aneurysm	Number	1990	94698.3725	102684.7839	87008.88448	
Deaths	Global	Both	All Ages	Aortic aneurysm	Number	2019	172426.748	182899.3603	157357.0767	82.08%
Deaths	Global	Both	Age-standardized	Aortic aneurysm	Rate	1990	2.69711075	2.907018262	2.470325664	
Deaths	Global	Both	Age-standardized	Aortic aneurysm	Rate	2019	2.21410454	2.351478608	2.004032936	-17.91%
DALYs	Global	Female	All Ages	Aortic aneurysm	Number	1990	592045.19	704594.664	552385.2828	
DALYs	Global	Female	All Ages	Aortic aneurysm	Number	2019	1069894.21	1158251.974	969435.5309	80.71%
DALYs	Global	Female	Age-standardized	Aortic aneurysm	Rate	1990	28.178751	33.19785496	26.19555562	
DALYs	Global	Female	Age-standardized	Aortic aneurysm	Rate	2019	24.5914862	26.62951908	22.28876576	-12.73%
Deaths	Global	Female	All Ages	Aortic aneurysm	Number	1990	31547.4003	35782.83284	28946.28151	
Deaths	Global	Female	All Ages	Aortic aneurysm	Number	2019	64085.69	69669.34905	55524.90349	103.14%
Deaths	Global	Female	Age-standardized	Aortic aneurysm	Rate	1990	1.61430471	1.813625191	1.467914284	
Deaths	Global	Female	Age-standardized	Aortic aneurysm	Rate	2019	1.46170428	1.589469777	1.267801919	-9.45%
DALYs	Global	Male	All Ages	Aortic aneurysm	Number	1990	1397568.33	1583994.116	1247431.722	
DALYs	Global	Male	All Ages	Aortic aneurysm	Number	2019	2252448.92	2400190.084	2099537.001	61.17%
DALYs	Global	Male	Age-standardized	Aortic aneurysm	Rate	1990	78.5172571	88.0391079	70.52192271	
DALYs	Global	Male	Age-standardized	Aortic aneurysm	Rate	2019	59.4527505	63.27136405	55.22883541	-24.28%
Deaths	Global	Male	All Ages	Aortic aneurysm	Number	1990	63150.9721	70314.87514	57035.28873	
Deaths	Global	Male	All Ages	Aortic aneurysm	Number	2019	108341.058	114716.0738	100182.0449	71.56%
Deaths	Global	Male	Age-standardized	Aortic aneurysm	Rate	1990	4.19119411	4.620393286	3.762343536	
Deaths	Global	Male	Age-standardized	Aortic aneurysm	Rate	2019	3.15439657	3.346205207	2.892762643	-24.74%
DALYs	High SDI	Both	All Ages	Aortic aneurysm	Number	1990	892344.217	915453.943	860937.3819	
DALYs	High SDI	Both	All Ages	Aortic aneurysm	Number	2019	1013966.19	1063166.425	930098.0512	13.63%
DALYs	High SDI	Both	Age-standardized	Aortic aneurysm	Rate	1990	84.7013285	86.84693268	81.71294943	
DALYs	High SDI	Both	Age-standardized	Aortic aneurysm	Rate	2019	54.7738746	57.17233352	51.06356118	-35.33%
Deaths	High SDI	Both	All Ages	Aortic aneurysm	Number	1990	49334.2106	50870.5001	46820.23972	
Deaths	High SDI	Both	All Ages	Aortic aneurysm	Number	2019	65848.4407	70352.47758	57721.61013	33.47%
Deaths	High SDI	Both	Age-standardized	Aortic aneurysm	Rate	1990	4.60146124	4.749078494	4.358075353	
Deaths	High SDI	Both	Age-standardized	Aortic aneurysm	Rate	2019	3.12636408	3.320598794	2.780543345	-32.06%
DALYs	High SDI	Female	All Ages	Aortic aneurysm	Number	1990	265654.674	274699.6246	250326.3685	
DALYs	High SDI	Female	All Ages	Aortic aneurysm	Number	2019	368092.184	399131.594	319810.7351	38.56%
DALYs	High SDI	Female	Age-standardized	Aortic aneurysm	Rate	1990	42.475563	43.81226071	40.20820235	
DALYs	High SDI	Female	Age-standardized	Aortic aneurysm	Rate	2019	33.7293072	36.15772762	30.14306035	-20.59%
Deaths	High SDI	Female	All Ages	Aortic aneurysm	Number	1990	16821.4961	17562.71705	15441.80303	
Deaths	High SDI	Female	All Ages	Aortic aneurysm	Number	2019	28118.2652	31181.41845	22979.37384	67.16%
Deaths	High SDI	Female	Age-standardized	Aortic aneurysm	Rate	1990	2.5227603	2.630810702	2.325489017	
Deaths	High SDI	Female	Age-standardized	Aortic aneurysm	Rate	2019	2.15823612	2.365283497	1.824509185	-14.45%
DALYs	High SDI	Male	All Ages	Aortic aneurysm	Number	1990	626689.543	643575.1913	609022.4065	
DALYs	High SDI	Male	All Ages	Aortic aneurysm	Number	2019	645874.004	675882.71	606671.7742	3.06%
DALYs	High SDI	Male	Age-standardized	Aortic aneurysm	Rate	1990	142.039723	145.9257321	137.6331375	
DALYs	High SDI	Male	Age-standardized	Aortic aneurysm	Rate	2019	78.3520829	81.87568575	73.8085382	-44.84%
Deaths	High SDI	Male	All Ages	Aortic aneurysm	Number	1990	32512.7146	33484.21907	31332.69264	
Deaths	High SDI	Male	All Ages	Aortic aneurysm	Number	2019	37730.1755	39809.03811	34448.77211	16.05%
Deaths	High SDI	Male	Age-standardized	Aortic aneurysm	Rate	1990	7.79491051	8.046402657	7.47436556	
Deaths	High SDI	Male	Age-standardized	Aortic aneurysm	Rate	2019	4.33848182	4.574188646	3.973020219	-44.34%
DALYs	High-income Asia Pacific	Both	All Ages	Aortic aneurysm	Number	1990	95807.655	99664.8823	91406.85699	
DALYs	High-income Asia Pacific	Both	All Ages	Aortic aneurysm	Number	2019	295709.051	318734.0308	255106.7138	208.65%
DALYs	High-income Asia Pacific	Both	Age-standardized	Aortic aneurysm	Rate	1990	48.2925805	50.37530195	45.85156814	
DALYs	High-income Asia Pacific	Both	Age-standardized	Aortic aneurysm	Rate	2019	65.5339298	69.83367063	58.70860693	35.70%
Deaths	High-income Asia Pacific	Both	All Ages	Aortic aneurysm	Number	1990	5126.36432	5380.720618	4794.239148	
Deaths	High-income Asia Pacific	Both	All Ages	Aortic aneurysm	Number	2019	21789.6332	24065.9683	17748.27978	325.05%
Deaths	High-income Asia Pacific	Both	Age-standardized	Aortic aneurysm	Rate	1990	2.73675695	2.886683373	2.534038182	
Deaths	High-income Asia Pacific	Both	Age-standardized	Aortic aneurysm	Rate	2019	3.94133788	4.294164901	3.320267787	44.01%
DALYs	High-income Asia Pacific	Female	All Ages	Aortic aneurysm	Number	1990	31042.0878	33517.01036	28752.81436	
DALYs	High-income Asia Pacific	Female	All Ages	Aortic aneurysm	Number	2019	124197.442	139910.6376	99652.83996	300.09%
DALYs	High-income Asia Pacific	Female	Age-standardized	Aortic aneurysm	Rate	1990	27.1977633	29.38554267	25.13323433	
DALYs	High-income Asia Pacific	Female	Age-standardized	Aortic aneurysm	Rate	2019	42.7820556	47.05465447	36.10337646	57.30%
Deaths	High-income Asia Pacific	Female	All Ages	Aortic aneurysm	Number	1990	1862.47718	2018.975711	1684.541671	
Deaths	High-income Asia Pacific	Female	All Ages	Aortic aneurysm	Number	2019	10871.3361	12536.62534	8097.078686	483.70%

Deaths	High-income Asia Pacific	Female	Age-standardized	Aortic aneurysm	Rate	1990	1.66579746	1.811857281	1.499747133	
Deaths	High-income Asia Pacific	Female	Age-standardized	Aortic aneurysm	Rate	2019	2.96231856	3.357422838	2.344105311	77.83%
DALYs	High-income Asia Pacific	Male	All Ages	Aortic aneurysm	Number	1990	64765.5672	67191.3268	62144.53543	
DALYs	High-income Asia Pacific	Male	All Ages	Aortic aneurysm	Number	2019	171511.609	185409.5496	155538.8713	164.82%
DALYs	High-income Asia Pacific	Male	Age-standardized	Aortic aneurysm	Rate	1990	77.009777	79.88087355	73.46876384	
DALYs	High-income Asia Pacific	Male	Age-standardized	Aortic aneurysm	Rate	2019	90.6649638	98.1915642	83.27941548	17.73%
Deaths	High-income Asia Pacific	Male	All Ages	Aortic aneurysm	Number	1990	3263.88714	3384.341204	3106.351922	
Deaths	High-income Asia Pacific	Male	All Ages	Aortic aneurysm	Number	2019	10918.2972	11872.57392	9494.945078	234.52%
Deaths	High-income Asia Pacific	Male	Age-standardized	Aortic aneurysm	Rate	1990	4.39383614	4.568103353	4.136455295	
Deaths	High-income Asia Pacific	Male	Age-standardized	Aortic aneurysm	Rate	2019	5.16179785	5.610444681	4.529848801	17.48%
DALYs	High-income North America	Both	All Ages	Aortic aneurysm	Number	1990	345270.121	356100.6191	332277.2054	
DALYs	High-income North America	Both	All Ages	Aortic aneurysm	Number	2019	277594.035	292684.6823	257827.7949	-19.60%
DALYs	High-income North America	Both	Age-standardized	Aortic aneurysm	Rate	1990	97.1475364	100.1624716	93.64443085	
DALYs	High-income North America	Both	Age-standardized	Aortic aneurysm	Rate	2019	47.0899242	49.74150997	43.91904632	-51.53%
Deaths	High-income North America	Both	All Ages	Aortic aneurysm	Number	1990	18810.1064	19498.63009	17747.92687	
Deaths	High-income North America	Both	All Ages	Aortic aneurysm	Number	2019	15972.3435	16972.03545	14439.09534	-15.09%
Deaths	High-income North America	Both	Age-standardized	Aortic aneurysm	Rate	1990	5.08954052	5.275251932	4.806666504	
Deaths	High-income North America	Both	Age-standardized	Aortic aneurysm	Rate	2019	2.44180698	2.587111714	2.232501867	-52.02%
DALYs	High-income North America	Female	All Ages	Aortic aneurysm	Number	1990	103810.023	108203.7622	96471.13765	
DALYs	High-income North America	Female	All Ages	Aortic aneurysm	Number	2019	100256.751	110234.9259	90696.98783	-3.42%
DALYs	High-income North America	Female	Age-standardized	Aortic aneurysm	Rate	1990	49.5392009	51.56277213	46.42042183	
DALYs	High-income North America	Female	Age-standardized	Aortic aneurysm	Rate	2019	29.7795347	32.71369577	27.30745705	-39.89%
Deaths	High-income North America	Female	All Ages	Aortic aneurysm	Number	1990	6410.7987	6748.905198	5794.897567	
Deaths	High-income North America	Female	All Ages	Aortic aneurysm	Number	2019	6736.94889	7459.779535	5860.037221	5.09%
Deaths	High-income North America	Female	Age-standardized	Aortic aneurysm	Rate	1990	2.8030184	2.940504945	2.555249876	
Deaths	High-income North America	Female	Age-standardized	Aortic aneurysm	Rate	2019	1.73247833	1.906649756	1.53329955	-38.19%
DALYs	High-income North America	Male	All Ages	Aortic aneurysm	Number	1990	241460.098	250452.7287	232600.7022	
DALYs	High-income North America	Male	All Ages	Aortic aneurysm	Number	2019	177337.285	186973.0958	164969.4959	-26.56%
DALYs	High-income North America	Male	Age-standardized	Aortic aneurysm	Rate	1990	159.83081	165.9013351	153.7700033	
DALYs	High-income North America	Male	Age-standardized	Aortic aneurysm	Rate	2019	66.3851956	69.96286617	61.55649031	-58.47%
Deaths	High-income North America	Male	All Ages	Aortic aneurysm	Number	1990	12399.3077	12911.51928	11849.61516	
Deaths	High-income North America	Male	All Ages	Aortic aneurysm	Number	2019	9235.39458	9809.750967	8492.911303	-25.52%
Deaths	High-income North America	Male	Age-standardized	Aortic aneurysm	Rate	1990	8.45680322	8.82277959	8.04088874	
Deaths	High-income North America	Male	Age-standardized	Aortic aneurysm	Rate	2019	3.30248184	3.50939622	3.040628507	-60.95%
DALYs	High-middle SDI	Both	All Ages	Aortic aneurysm	Number	1990	507345.716	540632.7161	478335.4431	
DALYs	High-middle SDI	Both	All Ages	Aortic aneurysm	Number	2019	933119.177	994068.3036	869842.7896	83.92%
DALYs	High-middle SDI	Both	Age-standardized	Aortic aneurysm	Rate	1990	46.7093987	49.63426201	44.05644937	
DALYs	High-middle SDI	Both	Age-standardized	Aortic aneurysm	Rate	2019	46.665364	49.72918882	43.50239219	-0.09%
Deaths	High-middle SDI	Both	All Ages	Aortic aneurysm	Number	1990	21460.0085	22630.75586	20268.88434	
Deaths	High-middle SDI	Both	All Ages	Aortic aneurysm	Number	2019	45028.14	47849.57925	41590.38252	109.82%
Deaths	High-middle SDI	Both	Age-standardized	Aortic aneurysm	Rate	1990	2.12816236	2.240154938	2.00331799	
Deaths	High-middle SDI	Both	Age-standardized	Aortic aneurysm	Rate	2019	2.24556311	2.385092435	2.070973114	5.52%
DALYs	High-middle SDI	Female	All Ages	Aortic aneurysm	Number	1990	143630.706	155165.8113	135511.6034	
DALYs	High-middle SDI	Female	All Ages	Aortic aneurysm	Number	2019	272958.505	294800.2151	247646.4644	90.04%
DALYs	High-middle SDI	Female	Age-standardized	Aortic aneurysm	Rate	1990	23.9950452	25.92421768	22.59920885	
DALYs	High-middle SDI	Female	Age-standardized	Aortic aneurysm	Rate	2019	24.8318142	26.85269495	22.57675548	3.49%
Deaths	High-middle SDI	Female	All Ages	Aortic aneurysm	Number	1990	6765.78681	7291.573764	6331.939537	
Deaths	High-middle SDI	Female	All Ages	Aortic aneurysm	Number	2019	15103.7186	16313.94407	13438.17619	123.24%
Deaths	High-middle SDI	Female	Age-standardized	Aortic aneurysm	Rate	1990	1.16906281	1.26014166	1.087098219	
Deaths	High-middle SDI	Female	Age-standardized	Aortic aneurysm	Rate	2019	1.30076011	1.404835499	1.161757977	11.27%
DALYs	High-middle SDI	Male	All Ages	Aortic aneurysm	Number	1990	363715.11	397963.1112	333801.9145	
DALYs	High-middle SDI	Male	All Ages	Aortic aneurysm	Number	2019	660160.672	715495.55	609742.2224	81.50%
DALYs	High-middle SDI	Male	Age-standardized	Aortic aneurysm	Rate	1990	76.0528838	82.71757457	70.16565167	
DALYs	High-middle SDI	Male	Age-standardized	Aortic aneurysm	Rate	2019	72.249099	78.28614464	66.65381373	-5.00%
Deaths	High-middle SDI	Male	All Ages	Aortic aneurysm	Number	1990	14694.2216	15869.90103	13592.90816	
Deaths	High-middle SDI	Male	All Ages	Aortic aneurysm	Number	2019	29924.4214	32119.76916	27571.31377	103.65%
Deaths	High-middle SDI	Male	Age-standardized	Aortic aneurysm	Rate	1990	3.54927227	3.806490799	3.273657255	
Deaths	High-middle SDI	Male	Age-standardized	Aortic aneurysm	Rate	2019	3.50014883	3.755255339	3.219382245	-1.38%
DALYs	Low SDI	Both	All Ages	Aortic aneurysm	Number	1990	107286.315	153847.7814	63144.52019	
DALYs	Low SDI	Both	All Ages	Aortic aneurysm	Number	2019	200999.02	255272.4431	148339.8169	87.35%
DALYs	Low SDI	Both	Age-standardized	Aortic aneurysm	Rate	1990	44.2862768	62.27212789	26.30336237	
DALYs	Low SDI	Both	Age-standardized	Aortic aneurysm	Rate	2019	37.4723337	46.8807259	27.87960444	-15.39%
Deaths	Low SDI	Both	All Ages	Aortic aneurysm	Number	1990	4253.84373	5954.513209	2526.067971	
Deaths	Low SDI	Both	All Ages	Aortic aneurysm	Number	2019	8257.83059	10285.24292	6160.997598	94.13%
Deaths	Low SDI	Both	Age-standardized	Aortic aneurysm	Rate	1990	2.15845694	2.949647591	1.306461378	
Deaths	Low SDI	Both	Age-standardized	Aortic aneurysm	Rate	2019	1.87273694	2.310263961	1.401663285	-13.24%
DALYs	Low SDI	Female	All Ages	Aortic aneurysm	Number	1990	34840.9675	57889.54499	22886.1581	
DALYs	Low SDI	Female	All Ages	Aortic aneurysm	Number	2019	65771.1285	83102.09845	51209.46782	88.78%
DALYs	Low SDI	Female	Age-standardized	Aortic aneurysm	Rate	1990	30.6178229	47.84930201	20.47298606	
DALYs	Low SDI	Female	Age-standardized	Aortic aneurysm	Rate	2019	25.0660469	30.65494189	19.58123629	-18.13%
Deaths	Low SDI	Female	All Ages	Aortic aneurysm	Number	1990	1472.79059	2269.036512	984.6457734	
Deaths	Low SDI	Female	All Ages	Aortic aneurysm	Number	2019	2928.69401	3563.270276	2292.186209	98.85%
Deaths	Low SDI	Female	Age-standardized	Aortic aneurysm	Rate	1990	1.61427179	2.328737177	1.096538391	
Deaths	Low SDI	Female	Age-standardized	Aortic aneurysm	Rate	2019	1.34837469	1.627110323	1.052577326	-16.47%
DALYs	Low SDI	Male	All Ages	Aortic aneurysm	Number	1990	72445.3477	117204.2213	37512.46573	
DALYs	Low SDI	Male	All Ages	Aortic aneurysm	Number	2019	135227.892	183678.0654	91155.41992	86.66%
DALYs	Low SDI	Male	Age-standardized	Aortic aneurysm	Rate	1990	57.4186763	91.70099637	29.43242464	
DALYs	Low SDI	Male	Age-standardized	Aortic aneurysm	Rate	2019	50.3401461	67.90197321	33.84601822	-12.33%
Deaths	Low SDI	Male	All Ages	Aortic aneurysm	Number	1990	2781.05314	4431.363173	1423.257551	
Deaths	Low SDI	Male	All Ages	Aortic aneurysm	Number	2019	5329.13658	7162.890384	3583.864782	91.62%
Deaths	Low SDI	Male	Age-standardized	Aortic aneurysm	Rate	1990	2.6841009	4.206580129	1.35143209	
Deaths	Low SDI	Male	Age-standardized	Aortic aneurysm	Rate	2019	2.43240886	3.252086919	1.625129941	-9.38%
DALYs	Low-middle SDI	Both	All Ages	Aortic aneurysm	Number	1990	188853.32	267481.3825	131484.4937	
DALYs	Low-middle SDI	Both	All Ages	Aortic aneurysm	Number	2019	473180.794	561094.2759	394006.606	150.55%
DALYs	Low-middle SDI	Both	Age-standardized	Aortic aneurysm	Rate	1990	31.0674493	43.08093846	21.87430843	
DALYs	Low-middle SDI	Both	Age-standardized	Aortic aneurysm	Rate	2019	34.3012695	40.47936542	28.66261775	10.41%
Deaths	Low-middle SDI	Both	All Ages	Aortic aneurysm	Number	1990	7618.05599	10540.6435	5368.780828	
Deaths	Low-middle SDI	Both	All Ages	Aortic aneurysm	Number	2019	20810.8858	24502.08237	17478.62217	173.18%
Deaths	Low-middle SDI	Both	Age-standardized	Aortic aneurysm	Rate	1990	1.53711959	2.077924348	1.096890175	
Deaths	Low-middle SDI	Both	Age-standardized	Aortic aneurysm	Rate	2019	1.71385093	2.003257754	1.447753229	11.50%
DALYs	Low-middle SDI	Female	All Ages	Aortic aneurysm	Number	1990	57066.3302	98982.65402	46520.23331	
DALYs	Low-middle SDI	Female	All Ages	Aortic aneurysm	Number	2019	151037.164	188807.0107	127245.8376	164.67%
DALYs	Low-middle SDI	Female	Age-standardized	Aortic aneurysm	Rate	1990	19.5946356	32.11538991	16.06113646	
DALYs	Low-middle SDI	Female	Age-standardized	Aortic aneurysm	Rate	2019	21.4450732	26.5557903	18.05049652	9.44%
Deaths	Low-middle SDI	Female	All Ages	Aortic aneurysm	Number	1990	2444.23388	3953.055514	1997.579345	
Deaths	Low-middle SDI	Female	All Ages	Aortic aneurysm	Number	2019	7175.99141	8729.933117	6030.810313	193.59%
Deaths	Low-middle SDI	Female	Age-standardized	Aortic aneurysm	Rate	1990	1.03999274	1.588145819	0.854531253	
Deaths	Low-middle SDI	Female	Age-standardized	Aortic aneurysm	Rate	2019	1.14339818	1.367897412	0.959624013	9.94%
DALYs	Low-middle SDI	Male	All Ages	Aortic aneurysm	Number	1990	131786.99	204747.6281	80548.35177	
DALYs	Low-middle SDI	Male	All Ages	Aortic aneurysm	Number	2019	322143.629	404380.4966	245842.6418	144.44%
DALYs	Low-middle SDI	Male	Age-standardized	Aortic aneurysm	Rate	1990	42.3980232	65.26850622	25.82890692	

DALYs	Low-middle SDI	Male	Age-standardized	Aortic aneurysm	Rate	2019	48.269941	60.41011611	37.20501485	13.85%
Deaths	Low-middle SDI	Male	All Ages	Aortic aneurysm	Number	1990	5173.82211	7960.879705	3143.271831	
Deaths	Low-middle SDI	Male	All Ages	Aortic aneurysm	Number	2019	13634.8944	17033.68005	10588.78085	163.54%
Deaths	Low-middle SDI	Male	Age-standardized	Aortic aneurysm	Rate	1990	2.0445032	3.100974701	1.236694663	
Deaths	Low-middle SDI	Male	Age-standardized	Aortic aneurysm	Rate	2019	2.3650002	2.923090102	1.851144068	15.68%
DALYs	Middle SDI	Both	All Ages	Aortic aneurysm	Number	1990	292553.76	349336.931	252837.9436	
DALYs	Middle SDI	Both	All Ages	Aortic aneurysm	Number	2019	698992.128	773159.9892	631941.1595	138.93%
DALYs	Middle SDI	Both	Age-standardized	Aortic aneurysm	Rate	1990	27.8413663	32.61157378	24.25288491	
DALYs	Middle SDI	Both	Age-standardized	Aortic aneurysm	Rate	2019	28.528437	31.58121032	25.72815814	2.47%
Deaths	Middle SDI	Both	All Ages	Aortic aneurysm	Number	1990	11970.7885	13993.49806	10456.05272	
Deaths	Middle SDI	Both	All Ages	Aortic aneurysm	Number	2019	32372.3421	35819.20653	29005.76223	170.43%
Deaths	Middle SDI	Both	Age-standardized	Aortic aneurysm	Rate	1990	1.41465878	1.631355417	1.239567682	
Deaths	Middle SDI	Both	Age-standardized	Aortic aneurysm	Rate	2019	1.47723285	1.63800904	1.326232654	4.42%
DALYs	Middle SDI	Female	All Ages	Aortic aneurysm	Number	1990	90495.4035	124947.6172	80366.59198	
DALYs	Middle SDI	Female	All Ages	Aortic aneurysm	Number	2019	211395.754	237059.9499	188066.9644	133.60%
DALYs	Middle SDI	Female	Age-standardized	Aortic aneurysm	Rate	1990	17.1836844	23.01207821	15.26462522	
DALYs	Middle SDI	Female	Age-standardized	Aortic aneurysm	Rate	2019	16.7316941	18.73745361	14.90001811	-2.63%
Deaths	Middle SDI	Female	All Ages	Aortic aneurysm	Number	1990	4023.50784	5279.350856	3568.765293	
Deaths	Middle SDI	Female	All Ages	Aortic aneurysm	Number	2019	10722.0181	11949.34734	9436.05865	166.48%
Deaths	Middle SDI	Female	Age-standardized	Aortic aneurysm	Rate	1990	0.93238821	1.172718009	0.822570445	
Deaths	Middle SDI	Female	Age-standardized	Aortic aneurysm	Rate	2019	0.92498501	1.029011502	0.809078914	-0.79%
DALYs	Middle SDI	Male	All Ages	Aortic aneurysm	Number	1990	202058.356	245865.7746	166951.5572	
DALYs	Middle SDI	Male	All Ages	Aortic aneurysm	Number	2019	487596.375	550138.1618	433141.1437	141.31%
DALYs	Middle SDI	Male	Age-standardized	Aortic aneurysm	Rate	1990	39.5135183	47.50648629	32.79223403	
DALYs	Middle SDI	Male	Age-standardized	Aortic aneurysm	Rate	2019	41.6406799	46.87493915	36.94164402	5.38%
Deaths	Middle SDI	Male	All Ages	Aortic aneurysm	Number	1990	7947.28068	9549.655548	6596.711818	
Deaths	Middle SDI	Male	All Ages	Aortic aneurysm	Number	2019	21650.3239	24380.05123	19193.10463	172.42%
Deaths	Middle SDI	Male	Age-standardized	Aortic aneurysm	Rate	1990	1.99708193	2.366173367	1.66162376	
Deaths	Middle SDI	Male	Age-standardized	Aortic aneurysm	Rate	2019	2.14631391	2.415911868	1.888197279	7.47%
DALYs	North Africa and Middle East	Both	All Ages	Aortic aneurysm	Number	1990	62422.9318	81157.0417	46913.40561	
DALYs	North Africa and Middle East	Both	All Ages	Aortic aneurysm	Number	2019	127698.19	150689.9279	110098.1265	104.57%
DALYs	North Africa and Middle East	Both	Age-standardized	Aortic aneurysm	Rate	1990	33.1256991	42.4206565	24.97144713	
DALYs	North Africa and Middle East	Both	Age-standardized	Aortic aneurysm	Rate	2019	28.0185067	32.8257906	24.27783007	-15.42%
Deaths	North Africa and Middle East	Both	All Ages	Aortic aneurysm	Number	1990	2320.30175	2949.752794	1752.18525	
Deaths	North Africa and Middle East	Both	All Ages	Aortic aneurysm	Number	2019	5232.89685	6096.303468	4554.740277	125.53%
Deaths	North Africa and Middle East	Both	Age-standardized	Aortic aneurysm	Rate	1990	1.48564653	1.861487105	1.134336188	
Deaths	North Africa and Middle East	Both	Age-standardized	Aortic aneurysm	Rate	2019	1.35382851	1.56572858	1.17741355	-8.87%
DALYs	North Africa and Middle East	Female	All Ages	Aortic aneurysm	Number	1990	14020.8925	20854.79881	11734.89499	
DALYs	North Africa and Middle East	Female	All Ages	Aortic aneurysm	Number	2019	30523.3022	38286.33521	25932.7233	117.70%
DALYs	North Africa and Middle East	Female	Age-standardized	Aortic aneurysm	Rate	1990	15.7827431	22.3091816	13.28609491	
DALYs	North Africa and Middle East	Female	Age-standardized	Aortic aneurysm	Rate	2019	14.0655545	17.27909553	12.07979442	-10.88%
Deaths	North Africa and Middle East	Female	All Ages	Aortic aneurysm	Number	1990	576.787727	792.5702377	487.0909016	
Deaths	North Africa and Middle East	Female	All Ages	Aortic aneurysm	Number	2019	1336.71063	1608.458553	1151.53683	131.75%
Deaths	North Africa and Middle East	Female	Age-standardized	Aortic aneurysm	Rate	1990	0.78944747	1.038655496	0.662794359	
Deaths	North Africa and Middle East	Female	Age-standardized	Aortic aneurysm	Rate	2019	0.72710697	0.865341184	0.625754741	-7.90%
DALYs	North Africa and Middle East	Male	All Ages	Aortic aneurysm	Number	1990	48402.0393	65398.20028	33823.46746	
DALYs	North Africa and Middle East	Male	All Ages	Aortic aneurysm	Number	2019	97174.8874	115167.6812	82985.00797	100.77%
DALYs	North Africa and Middle East	Male	Age-standardized	Aortic aneurysm	Rate	1990	50.0394438	66.69057277	34.77405585	
DALYs	North Africa and Middle East	Male	Age-standardized	Aortic aneurysm	Rate	2019	41.4036936	49.02754989	35.65844078	-17.26%
Deaths	North Africa and Middle East	Male	All Ages	Aortic aneurysm	Number	1990	1743.51403	2311.032754	1211.146508	
Deaths	North Africa and Middle East	Male	All Ages	Aortic aneurysm	Number	2019	3896.18622	4600.674561	3362.491655	123.47%
Deaths	North Africa and Middle East	Male	Age-standardized	Aortic aneurysm	Rate	1990	2.19231714	2.863902276	1.50452349	
Deaths	North Africa and Middle East	Male	Age-standardized	Aortic aneurysm	Rate	2019	1.96894244	2.319840302	1.697589503	-10.19%
DALYs	Oceania	Both	All Ages	Aortic aneurysm	Number	1990	2143.69887	3356.090386	1422.07175	
DALYs	Oceania	Both	All Ages	Aortic aneurysm	Number	2019	4472.07767	6774.989844	2988.697118	108.62%
DALYs	Oceania	Both	Age-standardized	Aortic aneurysm	Rate	1990	69.6314322	106.3592753	47.94326036	
DALYs	Oceania	Both	Age-standardized	Aortic aneurysm	Rate	2019	60.4490889	88.10734176	42.30926235	-13.19%
Deaths	Oceania	Both	All Ages	Aortic aneurysm	Number	1990	79.6709436	122.4913966	54.40027604	
Deaths	Oceania	Both	All Ages	Aortic aneurysm	Number	2019	168.103826	243.9754475	117.7726542	111.00%
Deaths	Oceania	Both	Age-standardized	Aortic aneurysm	Rate	1990	3.49549429	5.150208623	2.47139581	
Deaths	Oceania	Both	Age-standardized	Aortic aneurysm	Rate	2019	2.97507132	4.152161853	2.176332891	-14.89%
DALYs	Oceania	Female	All Ages	Aortic aneurysm	Number	1990	647.380788	989.422771	427.590367	
DALYs	Oceania	Female	All Ages	Aortic aneurysm	Number	2019	1340.69743	2024.973006	928.4760802	107.10%
DALYs	Oceania	Female	Age-standardized	Aortic aneurysm	Rate	1990	48.1535637	70.66766982	32.8801765	
DALYs	Oceania	Female	Age-standardized	Aortic aneurysm	Rate	2019	40.7525768	59.38448821	28.98291246	-15.37%
Deaths	Oceania	Female	All Ages	Aortic aneurysm	Number	1990	27.1920747	40.01431612	18.52295098	
Deaths	Oceania	Female	All Ages	Aortic aneurysm	Number	2019	57.3178483	83.25459223	40.95248473	110.79%
Deaths	Oceania	Female	Age-standardized	Aortic aneurysm	Rate	1990	2.73710466	3.822830054	1.901005883	
Deaths	Oceania	Female	Age-standardized	Aortic aneurysm	Rate	2019	2.24635112	3.152516318	1.668482419	-17.93%
DALYs	Oceania	Male	All Ages	Aortic aneurysm	Number	1990	1496.31808	2541.964661	850.3062751	
DALYs	Oceania	Male	All Ages	Aortic aneurysm	Number	2019	3131.38024	4905.385284	1890.1511	109.27%
DALYs	Oceania	Male	Age-standardized	Aortic aneurysm	Rate	1990	89.5196355	149.7247652	52.98333679	
DALYs	Oceania	Male	Age-standardized	Aortic aneurysm	Rate	2019	78.9936919	122.2476131	49.61670779	-11.76%
Deaths	Oceania	Male	All Ages	Aortic aneurysm	Number	1990	52.4788689	88.14101367	30.97567121	
Deaths	Oceania	Male	All Ages	Aortic aneurysm	Number	2019	110.785978	171.5887584	69.79921509	111.11%
Deaths	Oceania	Male	Age-standardized	Aortic aneurysm	Rate	1990	4.16678462	6.846440801	2.591605253	
Deaths	Oceania	Male	Age-standardized	Aortic aneurysm	Rate	2019	3.67118168	5.54165058	2.466605616	-11.89%
DALYs	South Asia	Both	All Ages	Aortic aneurysm	Number	1990	177753.809	271820.0822	110241.0549	
DALYs	South Asia	Both	All Ages	Aortic aneurysm	Number	2019	465928.958	577718.6027	360120.2822	162.12%
DALYs	South Asia	Both	Age-standardized	Aortic aneurysm	Rate	1990	31.6754868	47.65094719	19.96107819	
DALYs	South Asia	Both	Age-standardized	Aortic aneurysm	Rate	2019	33.190981	40.87502483	25.89227932	4.78%
Deaths	South Asia	Both	All Ages	Aortic aneurysm	Number	1990	7156.4342	10766.89662	4515.270017	
Deaths	South Asia	Both	All Ages	Aortic aneurysm	Number	2019	20814.0831	25442.82101	16394.71524	190.84%
Deaths	South Asia	Both	Age-standardized	Aortic aneurysm	Rate	1990	1.60704467	2.358822915	1.037495888	
Deaths	South Asia	Both	Age-standardized	Aortic aneurysm	Rate	2019	1.70099566	2.060991226	1.3530626	5.85%
DALYs	South Asia	Female	All Ages	Aortic aneurysm	Number	1990	47339.6206	89750.62897	35444.05988	
DALYs	South Asia	Female	All Ages	Aortic aneurysm	Number	2019	143389.806	184392.6226	110309.124	202.90%
DALYs	South Asia	Female	Age-standardized	Aortic aneurysm	Rate	1990	18.7034377	33.34474595	13.95308269	
DALYs	South Asia	Female	Age-standardized	Aortic aneurysm	Rate	2019	20.6341024	25.99424607	15.88974797	10.32%
Deaths	South Asia	Female	All Ages	Aortic aneurysm	Number	1990	2025.97037	3601.334064	1508.691103	
Deaths	South Asia	Female	All Ages	Aortic aneurysm	Number	2019	6942.54667	8577.498121	5349.014252	242.68%
Deaths	South Asia	Female	Age-standardized	Aortic aneurysm	Rate	1990	1.03509617	1.716896287	0.76527825	
Deaths	South Asia	Female	Age-standardized	Aortic aneurysm	Rate	2019	1.14146512	1.382962433	0.87745013	10.28%
DALYs	South Asia	Male	All Ages	Aortic aneurysm	Number	1990	130414.188	223370.4874	69062.02558	
DALYs	South Asia	Male	All Ages	Aortic aneurysm	Number	2019	322539.152	431515.4936	215722.1005	147.32%
DALYs	South Asia	Male	Age-standardized	Aortic aneurysm	Rate	1990	43.5166281	74.21304729	23.26766663	
DALYs	South Asia	Male	Age-standardized	Aortic aneurysm	Rate	2019	46.1656275	61.80579185	31.48438653	6.09%
Deaths	South Asia	Male	All Ages	Aortic aneurysm	Number	1990	5130.46383	8757.494601	2743.025117	
Deaths	South Asia	Male	All Ages	Aortic aneurysm	Number	2019	13871.5365	18459.29826	9548.604856	170.38%
Deaths	South Asia	Male	Age-standardized	Aortic aneurysm	Rate	1990	2.13466252	3.602303595	1.134502051	
Deaths	South Asia	Male	Age-standardized	Aortic aneurysm	Rate	2019	2.30062854	3.03884752	1.608027705	7.77%

DALYs	Southeast Asia	Both	All Ages	Aortic aneurysm	Number	1990	67472.0403	82027.32418	56220.30708	
DALYs	Southeast Asia	Both	All Ages	Aortic aneurysm	Number	2019	193135.081	233528.4588	162159.5196	186.24%
DALYs	Southeast Asia	Both	Age-standardized	Aortic aneurysm	Rate	1990	27.1884439	32.52286536	22.89008718	
DALYs	Southeast Asia	Both	Age-standardized	Aortic aneurysm	Rate	2019	33.3099556	40.14086435	28.00658188	22.52%
Deaths	Southeast Asia	Both	All Ages	Aortic aneurysm	Number	1990	3036.06405	3613.373064	2568.550071	
Deaths	Southeast Asia	Both	All Ages	Aortic aneurysm	Number	2019	9360.33411	11199.58779	7845.971489	208.30%
Deaths	Southeast Asia	Both	Age-standardized	Aortic aneurysm	Rate	1990	1.52011461	1.796294382	1.298312553	
Deaths	Southeast Asia	Both	Age-standardized	Aortic aneurysm	Rate	2019	1.87142444	2.235155533	1.562764106	23.11%
DALYs	Southeast Asia	Female	All Ages	Aortic aneurysm	Number	1990	22914.5033	33624.13029	19234.06069	
DALYs	Southeast Asia	Female	All Ages	Aortic aneurysm	Number	2019	61695.3941	75854.82827	52206.02729	169.24%
DALYs	Southeast Asia	Female	Age-standardized	Aortic aneurysm	Rate	1990	17.8916023	24.80677788	15.01370905	
DALYs	Southeast Asia	Female	Age-standardized	Aortic aneurysm	Rate	2019	19.9913698	24.45158513	16.97969586	11.74%
Deaths	Southeast Asia	Female	All Ages	Aortic aneurysm	Number	1990	1140.97994	1529.179932	961.0320916	
Deaths	Southeast Asia	Female	All Ages	Aortic aneurysm	Number	2019	3330.52981	4024.415219	2828.202507	191.90%
Deaths	Southeast Asia	Female	Age-standardized	Aortic aneurysm	Rate	1990	1.08381686	1.390023941	0.909028703	
Deaths	Southeast Asia	Female	Age-standardized	Aortic aneurysm	Rate	2019	1.20758388	1.452800191	1.014276668	11.42%
DALYs	Southeast Asia	Male	All Ages	Aortic aneurysm	Number	1990	44557.537	58231.71531	35183.8349	
DALYs	Southeast Asia	Male	All Ages	Aortic aneurysm	Number	2019	131439.687	163437.6421	105891.8597	194.99%
DALYs	Southeast Asia	Male	Age-standardized	Aortic aneurysm	Rate	1990	38.0448743	49.21426588	30.24519164	
DALYs	Southeast Asia	Male	Age-standardized	Aortic aneurysm	Rate	2019	49.6065857	61.38735192	40.21067233	30.39%
Deaths	Southeast Asia	Male	All Ages	Aortic aneurysm	Number	1990	1895.08411	2446.822726	1506.095761	
Deaths	Southeast Asia	Male	All Ages	Aortic aneurysm	Number	2019	6029.8043	7451.75747	4866.974132	218.18%
Deaths	Southeast Asia	Male	Age-standardized	Aortic aneurysm	Rate	1990	2.06383461	2.653219079	1.644366818	
Deaths	Southeast Asia	Male	Age-standardized	Aortic aneurysm	Rate	2019	2.76107635	3.397625433	2.216161386	33.78%
DALYs	Southern Latin America	Both	All Ages	Aortic aneurysm	Number	1990	37242.1592	40392.85629	34053.41414	
DALYs	Southern Latin America	Both	All Ages	Aortic aneurysm	Number	2019	54879.6675	59036.82368	50705.64741	47.36%
DALYs	Southern Latin America	Both	Age-standardized	Aortic aneurysm	Rate	1990	80.0100552	86.51493203	73.36011097	
DALYs	Southern Latin America	Both	Age-standardized	Aortic aneurysm	Rate	2019	66.7081551	71.62771809	61.71291859	-16.63%
Deaths	Southern Latin America	Both	All Ages	Aortic aneurysm	Number	1990	1741.42963	1900.365208	1577.214602	
Deaths	Southern Latin America	Both	All Ages	Aortic aneurysm	Number	2019	2812.65607	3037.649347	2563.989596	61.51%
Deaths	Southern Latin America	Both	Age-standardized	Aortic aneurysm	Rate	1990	3.88409893	4.234238413	3.519511605	
Deaths	Southern Latin America	Both	Age-standardized	Aortic aneurysm	Rate	2019	3.31956277	3.578881071	3.035456284	-14.53%
DALYs	Southern Latin America	Female	All Ages	Aortic aneurysm	Number	1990	8826.01884	9547.685561	8155.126876	
DALYs	Southern Latin America	Female	All Ages	Aortic aneurysm	Number	2019	15709.6056	16950.29451	14370.03876	77.99%
DALYs	Southern Latin America	Female	Age-standardized	Aortic aneurysm	Rate	1990	34.6277707	37.4672363	31.99265463	
DALYs	Southern Latin America	Female	Age-standardized	Aortic aneurysm	Rate	2019	33.9323723	36.5866801	31.12504627	-2.01%
Deaths	Southern Latin America	Female	All Ages	Aortic aneurysm	Number	1990	450.007034	491.9113051	409.6686704	
Deaths	Southern Latin America	Female	All Ages	Aortic aneurysm	Number	2019	913.619043	1000.884227	813.1319563	103.02%
Deaths	Southern Latin America	Female	Age-standardized	Aortic aneurysm	Rate	1990	1.79364221	1.955169479	1.63005311	
Deaths	Southern Latin America	Female	Age-standardized	Aortic aneurysm	Rate	2019	1.81971252	1.985984905	1.629818277	1.45%
DALYs	Southern Latin America	Male	All Ages	Aortic aneurysm	Number	1990	28416.1404	31081.34877	25766.65415	
DALYs	Southern Latin America	Male	All Ages	Aortic aneurysm	Number	2019	39170.0619	42326.9046	36047.47176	37.84%
DALYs	Southern Latin America	Male	Age-standardized	Aortic aneurysm	Rate	1990	136.527819	149.0970968	123.9273898	
DALYs	Southern Latin America	Male	Age-standardized	Aortic aneurysm	Rate	2019	106.800694	115.4170624	98.26828846	-21.77%
Deaths	Southern Latin America	Male	All Ages	Aortic aneurysm	Number	1990	1291.42259	1421.227917	1158.828765	
Deaths	Southern Latin America	Male	All Ages	Aortic aneurysm	Number	2019	1899.03703	2059.894958	1726.05393	47.05%
Deaths	Southern Latin America	Male	Age-standardized	Aortic aneurysm	Rate	1990	6.67838049	7.335417436	5.997184246	
Deaths	Southern Latin America	Male	Age-standardized	Aortic aneurysm	Rate	2019	5.30971298	5.75428319	4.822223197	-20.49%
DALYs	Southern Sub-Saharan Africa	Both	All Ages	Aortic aneurysm	Number	1990	17678.3935	19528.30228	16223.57434	
DALYs	Southern Sub-Saharan Africa	Both	All Ages	Aortic aneurysm	Number	2019	24510.1958	27272.54307	22105.00258	38.64%
DALYs	Southern Sub-Saharan Africa	Both	Age-standardized	Aortic aneurysm	Rate	1990	60.4207947	66.71266874	55.64581856	
DALYs	Southern Sub-Saharan Africa	Both	Age-standardized	Aortic aneurysm	Rate	2019	42.3703258	46.92820278	38.4028709	-29.87%
Deaths	Southern Sub-Saharan Africa	Both	All Ages	Aortic aneurysm	Number	1990	750.934794	830.9360645	688.3880291	
Deaths	Southern Sub-Saharan Africa	Both	All Ages	Aortic aneurysm	Number	2019	1064.75396	1174.487141	969.5844505	41.79%
Deaths	Southern Sub-Saharan Africa	Both	Age-standardized	Aortic aneurysm	Rate	1990	3.08449464	3.41893125	2.802418567	
Deaths	Southern Sub-Saharan Africa	Both	Age-standardized	Aortic aneurysm	Rate	2019	2.16964445	2.376584661	1.965204432	-29.66%
DALYs	Southern Sub-Saharan Africa	Female	All Ages	Aortic aneurysm	Number	1990	6567.41891	7222.2678	5845.455853	
DALYs	Southern Sub-Saharan Africa	Female	All Ages	Aortic aneurysm	Number	2019	7851.78844	8917.313155	6827.643375	19.56%
DALYs	Southern Sub-Saharan Africa	Female	Age-standardized	Aortic aneurysm	Rate	1990	39.563869	43.48735808	35.06439186	
DALYs	Southern Sub-Saharan Africa	Female	Age-standardized	Aortic aneurysm	Rate	2019	23.9063328	26.97934183	21.03320912	-39.58%
Deaths	Southern Sub-Saharan Africa	Female	All Ages	Aortic aneurysm	Number	1990	295.944495	327.6552184	256.8259398	
Deaths	Southern Sub-Saharan Africa	Female	All Ages	Aortic aneurysm	Number	2019	374.498542	417.3123597	329.3507487	26.54%
Deaths	Southern Sub-Saharan Africa	Female	Age-standardized	Aortic aneurysm	Rate	1990	2.08639989	2.325685248	1.783835568	
Deaths	Southern Sub-Saharan Africa	Female	Age-standardized	Aortic aneurysm	Rate	2019	1.28142736	1.426658823	1.12760792	-38.58%
DALYs	Southern Sub-Saharan Africa	Male	All Ages	Aortic aneurysm	Number	1990	11110.9746	12769.28112	9784.198512	
DALYs	Southern Sub-Saharan Africa	Male	All Ages	Aortic aneurysm	Number	2019	16658.4074	18903.93324	14612.02128	49.93%
DALYs	Southern Sub-Saharan Africa	Male	Age-standardized	Aortic aneurysm	Rate	1990	88.4284076	101.3255147	78.70441091	
DALYs	Southern Sub-Saharan Africa	Male	Age-standardized	Aortic aneurysm	Rate	2019	69.1693729	77.67593989	61.24233177	-21.78%
Deaths	Southern Sub-Saharan Africa	Male	All Ages	Aortic aneurysm	Number	1990	454.990299	520.7693315	404.6763436	
Deaths	Southern Sub-Saharan Africa	Male	All Ages	Aortic aneurysm	Number	2019	690.255422	775.0284178	612.9681569	51.71%
Deaths	Southern Sub-Saharan Africa	Male	Age-standardized	Aortic aneurysm	Rate	1990	4.57293838	5.219585424	4.068054078	
Deaths	Southern Sub-Saharan Africa	Male	Age-standardized	Aortic aneurysm	Rate	2019	3.65919615	4.093471067	3.250243995	-19.98%
DALYs	Tropical Latin America	Both	All Ages	Aortic aneurysm	Number	1990	77743.1101	80821.32414	74881.54735	
DALYs	Tropical Latin America	Both	All Ages	Aortic aneurysm	Number	2019	234827.583	248797.4808	219888.6575	202.06%
DALYs	Tropical Latin America	Both	Age-standardized	Aortic aneurysm	Rate	1990	78.7969046	81.89396504	75.61593025	
DALYs	Tropical Latin America	Both	Age-standardized	Aortic aneurysm	Rate	2019	96.1134045	101.9423722	89.75127635	21.98%
Deaths	Tropical Latin America	Both	All Ages	Aortic aneurysm	Number	1990	2951.5894	3073.848533	2817.972055	
Deaths	Tropical Latin America	Both	All Ages	Aortic aneurysm	Number	2019	10675.9777	11396.80444	9790.049487	261.70%
Deaths	Tropical Latin America	Both	Age-standardized	Aortic aneurysm	Rate	1990	3.41706145	3.572228749	3.23585103	
Deaths	Tropical Latin America	Both	Age-standardized	Aortic aneurysm	Rate	2019	4.53324716	4.843459019	4.138312931	32.67%
DALYs	Tropical Latin America	Female	All Ages	Aortic aneurysm	Number	1990	25100.8204	26411.70533	23885.89927	
DALYs	Tropical Latin America	Female	All Ages	Aortic aneurysm	Number	2019	91114.8334	99366.38367	83144.45136	263.00%
DALYs	Tropical Latin America	Female	Age-standardized	Aortic aneurysm	Rate	1990	48.7539323	51.38378296	46.21362819	
DALYs	Tropical Latin America	Female	Age-standardized	Aortic aneurysm	Rate	2019	68.6074765	74.82876978	62.56509639	40.72%
Deaths	Tropical Latin America	Female	All Ages	Aortic aneurysm	Number	1990	987.551272	1044.156579	929.7029253	
Deaths	Tropical Latin America	Female	All Ages	Aortic aneurysm	Number	2019	4511.9027	4940.384876	4008.650996	356.88%
Deaths	Tropical Latin America	Female	Age-standardized	Aortic aneurysm	Rate	1990	2.18456609	2.316297498	2.03323956	
Deaths	Tropical Latin America	Female	Age-standardized	Aortic aneurysm	Rate	2019	3.41338982	3.738760107	3.031075268	56.25%
DALYs	Tropical Latin America	Male	All Ages	Aortic aneurysm	Number	1990	52642.2898	55025.35478	50511.79733	
DALYs	Tropical Latin America	Male	All Ages	Aortic aneurysm	Number	2019	143712.749	154728.6594	133694.2464	173.00%
DALYs	Tropical Latin America	Male	Age-standardized	Aortic aneurysm	Rate	1990	112.540107	117.7866669	107.7421337	
DALYs	Tropical Latin America	Male	Age-standardized	Aortic aneurysm	Rate	2019	128.416942	138.067686	119.4817122	14.11%
Deaths	Tropical Latin America	Male	All Ages	Aortic aneurysm	Number	1990	1964.03813	2057.081217	1876.294604	
Deaths	Tropical Latin America	Male	All Ages	Aortic aneurysm	Number	2019	6164.07501	6639.396292	5682.483073	213.85%
Deaths	Tropical Latin America	Male	Age-standardized	Aortic aneurysm	Rate	1990	4.86329316	5.098520973	4.613595395	
Deaths	Tropical Latin America	Male	Age-standardized	Aortic aneurysm	Rate	2019	5.91790506	6.389370577	5.436726055	21.69%
DALYs	Western Europe	Both	All Ages	Aortic aneurysm	Number	1990	483276.958	495948.7347	466622.6082	
DALYs	Western Europe	Both	All Ages	Aortic aneurysm	Number	2019	482389.684	503974.8297	448785.0156	-0.18%
DALYs	Western Europe	Both	Age-standardized	Aortic aneurysm	Rate	1990	83.6631945	85.82980013	80.94235046	
DALYs	Western Europe	Both	Age-standardized	Aortic aneurysm	Rate	2019	54.6036454	56.9022206	51.42987409	-34.73%
Deaths	Western Europe	Both	All Ages	Aortic aneurysm	Number	1990	27016.8464	27907.18913	25667.67548	

Deaths	Western Europe	Both	All Ages	Aortic aneurysm	Number	2019	31444.1479	33286.07751	28351.012	16.39%
Deaths	Western Europe	Both	Age-standardized	Aortic aneurysm	Rate	1990	4.48454901	4.626857458	4.261042972	
Deaths	Western Europe	Both	Age-standardized	Aortic aneurysm	Rate	2019	3.06614014	3.224995799	2.804622785	-31.63%
DALYs	Western Europe	Female	All Ages	Aortic aneurysm	Number	1990	131818.37	136846.131	124056.7461	
DALYs	Western Europe	Female	All Ages	Aortic aneurysm	Number	2019	150087.987	162281.8216	133789.0623	13.86%
DALYs	Western Europe	Female	Age-standardized	Aortic aneurysm	Rate	1990	37.561682	38.90807213	35.63624717	
DALYs	Western Europe	Female	Age-standardized	Aortic aneurysm	Rate	2019	29.0300168	31.08527169	26.3410392	-22.71%
Deaths	Western Europe	Female	All Ages	Aortic aneurysm	Number	1990	8631.09589	9033.673322	7949.051137	
Deaths	Western Europe	Female	All Ages	Aortic aneurysm	Number	2019	11195.2651	12293.57529	9638.574385	29.71%
Deaths	Western Europe	Female	Age-standardized	Aortic aneurysm	Rate	1990	2.25558163	2.353801421	2.088729809	
Deaths	Western Europe	Female	Age-standardized	Aortic aneurysm	Rate	2019	1.77154212	1.924120535	1.560897258	-21.46%
DALYs	Western Europe	Male	All Ages	Aortic aneurysm	Number	1990	351458.588	360872.068	341747.3736	
DALYs	Western Europe	Male	All Ages	Aortic aneurysm	Number	2019	332301.698	349462.0354	310846.4099	-5.45%
DALYs	Western Europe	Male	Age-standardized	Aortic aneurysm	Rate	1990	146.913949	150.7419394	142.6654381	
DALYs	Western Europe	Male	Age-standardized	Aortic aneurysm	Rate	2019	84.4862855	88.75937205	79.59466691	-42.49%
Deaths	Western Europe	Male	All Ages	Aortic aneurysm	Number	1990	18385.7505	18938.05568	17751.76022	
Deaths	Western Europe	Male	All Ages	Aortic aneurysm	Number	2019	20248.8828	21327.74742	18617.00161	10.13%
Deaths	Western Europe	Male	Age-standardized	Aortic aneurysm	Rate	1990	7.94810903	8.189212131	7.623475916	
Deaths	Western Europe	Male	Age-standardized	Aortic aneurysm	Rate	2019	4.75681708	5.010319743	4.388459415	-40.15%
DALYs	Western Sub-Saharan Africa	Both	All Ages	Aortic aneurysm	Number	1990	37864.093	51755.17784	25936.98545	
DALYs	Western Sub-Saharan Africa	Both	All Ages	Aortic aneurysm	Number	2019	59599.1378	74649.26161	46140.49494	57.40%
DALYs	Western Sub-Saharan Africa	Both	Age-standardized	Aortic aneurysm	Rate	1990	42.5226238	57.8647913	29.49976463	
DALYs	Western Sub-Saharan Africa	Both	Age-standardized	Aortic aneurysm	Rate	2019	30.6959468	38.14889358	24.22214635	-27.81%
Deaths	Western Sub-Saharan Africa	Both	All Ages	Aortic aneurysm	Number	1990	1601.63838	2168.047352	1120.124382	
Deaths	Western Sub-Saharan Africa	Both	All Ages	Aortic aneurysm	Number	2019	2485.4741	3074.21355	1977.199794	55.18%
Deaths	Western Sub-Saharan Africa	Both	Age-standardized	Aortic aneurysm	Rate	1990	2.12942342	2.8806416	1.511885486	
Deaths	Western Sub-Saharan Africa	Both	Age-standardized	Aortic aneurysm	Rate	2019	1.5735473	1.919433598	1.26789814	-26.10%
DALYs	Western Sub-Saharan Africa	Female	All Ages	Aortic aneurysm	Number	1990	16607.0321	24531.94531	11408.86008	
DALYs	Western Sub-Saharan Africa	Female	All Ages	Aortic aneurysm	Number	2019	18291.8131	23945.41396	13857.61561	10.14%
DALYs	Western Sub-Saharan Africa	Female	Age-standardized	Aortic aneurysm	Rate	1990	38.3674358	56.13161978	26.45706273	
DALYs	Western Sub-Saharan Africa	Female	Age-standardized	Aortic aneurysm	Rate	2019	18.0653868	23.38606203	14.0292827	-52.91%
Deaths	Western Sub-Saharan Africa	Female	All Ages	Aortic aneurysm	Number	1990	742.146331	1073.120583	510.6960513	
Deaths	Western Sub-Saharan Africa	Female	All Ages	Aortic aneurysm	Number	2019	768.054682	982.5462193	602.5427979	3.49%
Deaths	Western Sub-Saharan Africa	Female	Age-standardized	Aortic aneurysm	Rate	1990	1.97039185	2.816740241	1.370175346	
Deaths	Western Sub-Saharan Africa	Female	Age-standardized	Aortic aneurysm	Rate	2019	0.94089226	1.180069104	0.741856357	-52.25%
DALYs	Western Sub-Saharan Africa	Male	All Ages	Aortic aneurysm	Number	1990	21257.0609	31435.23922	12190.29192	
DALYs	Western Sub-Saharan Africa	Male	All Ages	Aortic aneurysm	Number	2019	41307.3247	53820.63517	31045.80103	94.32%
DALYs	Western Sub-Saharan Africa	Male	Age-standardized	Aortic aneurysm	Rate	1990	46.532448	67.89379499	27.14939552	
DALYs	Western Sub-Saharan Africa	Male	Age-standardized	Aortic aneurysm	Rate	2019	44.5775135	57.56913461	34.21329152	-4.20%
Deaths	Western Sub-Saharan Africa	Male	All Ages	Aortic aneurysm	Number	1990	859.492051	1249.228843	501.432253	
Deaths	Western Sub-Saharan Africa	Male	All Ages	Aortic aneurysm	Number	2019	1717.41942	2200.144845	1329.374169	99.82%
Deaths	Western Sub-Saharan Africa	Male	Age-standardized	Aortic aneurysm	Rate	1990	2.27597584	3.260442658	1.355992923	
Deaths	Western Sub-Saharan Africa	Male	Age-standardized	Aortic aneurysm	Rate	2019	2.27880401	2.874699764	1.77642253	0.12%
DALYs	World Bank High Income	Both	All Ages	Aortic aneurysm	Number	1990	1019823.97	1043949.601	985536.7845	
DALYs	World Bank High Income	Both	All Ages	Aortic aneurysm	Number	2019	1216868.75	1275573.34	1121282.475	19.32%
DALYs	World Bank High Income	Both	Age-standardized	Aortic aneurysm	Rate	1990	79.0444176	80.87934986	76.39923765	
DALYs	World Bank High Income	Both	Age-standardized	Aortic aneurysm	Rate	2019	55.2636746	57.7201168	51.69387765	-30.09%
Deaths	World Bank High Income	Both	All Ages	Aortic aneurysm	Number	1990	55469.2537	57094.79741	52735.17467	
Deaths	World Bank High Income	Both	All Ages	Aortic aneurysm	Number	2019	77591.9429	82650.23438	68317.47534	39.88%
Deaths	World Bank High Income	Both	Age-standardized	Aortic aneurysm	Rate	1990	4.22590684	4.352036169	4.005993574	
Deaths	World Bank High Income	Both	Age-standardized	Aortic aneurysm	Rate	2019	3.080838	3.261778883	2.76356236	-27.10%
DALYs	World Bank High Income	Female	All Ages	Aortic aneurysm	Number	1990	294868.359	304665.4154	278364.2561	
DALYs	World Bank High Income	Female	All Ages	Aortic aneurysm	Number	2019	422863.065	457322.3568	371636.9967	43.41%
DALYs	World Bank High Income	Female	Age-standardized	Aortic aneurysm	Rate	1990	38.6790138	39.89579826	36.67550755	
DALYs	World Bank High Income	Female	Age-standardized	Aortic aneurysm	Rate	2019	32.4850311	34.69115342	29.13398535	-16.01%
Deaths	World Bank High Income	Female	All Ages	Aortic aneurysm	Number	1990	18416.2574	19205.43347	16933.17886	
Deaths	World Bank High Income	Female	All Ages	Aortic aneurysm	Number	2019	31788.2012	35186.77867	26044.64513	72.61%
Deaths	World Bank High Income	Female	Age-standardized	Aortic aneurysm	Rate	1990	2.27105684	2.365286765	2.094596279	
Deaths	World Bank High Income	Female	Age-standardized	Aortic aneurysm	Rate	2019	2.02903283	2.216486426	1.736066409	-10.66%
DALYs	World Bank High Income	Male	All Ages	Aortic aneurysm	Number	1990	724955.613	742311.6715	706373.1596	
DALYs	World Bank High Income	Male	All Ages	Aortic aneurysm	Number	2019	794005.686	831719.2438	748089.4696	9.52%
DALYs	World Bank High Income	Male	Age-standardized	Aortic aneurysm	Rate	1990	133.358123	136.5879098	129.4810162	
DALYs	World Bank High Income	Male	Age-standardized	Aortic aneurysm	Rate	2019	81.0797297	84.96062505	76.50415721	-39.20%
Deaths	World Bank High Income	Male	All Ages	Aortic aneurysm	Number	1990	37052.9963	38038.29223	35763.96896	
Deaths	World Bank High Income	Male	All Ages	Aortic aneurysm	Number	2019	45803.7417	48368.71845	41791.57683	23.62%
Deaths	World Bank High Income	Male	Age-standardized	Aortic aneurysm	Rate	1990	7.1925874	7.398195322	6.905485957	
Deaths	World Bank High Income	Male	Age-standardized	Aortic aneurysm	Rate	2019	4.41194818	4.655005756	4.038989865	-38.66%
DALYs	World Bank Low Income	Both	All Ages	Aortic aneurysm	Number	1990	78679.4229	110459.4916	47608.91463	
DALYs	World Bank Low Income	Both	All Ages	Aortic aneurysm	Number	2019	131777.593	170816.9847	95244.63087	67.49%
DALYs	World Bank Low Income	Both	Age-standardized	Aortic aneurysm	Rate	1990	51.0545026	69.83111251	30.98109313	
DALYs	World Bank Low Income	Both	Age-standardized	Aortic aneurysm	Rate	2019	39.8046227	50.66164056	28.81825446	-22.04%
Deaths	World Bank Low Income	Both	All Ages	Aortic aneurysm	Number	1990	3096.15724	4224.214536	1874.977455	
Deaths	World Bank Low Income	Both	All Ages	Aortic aneurysm	Number	2019	5281.03197	6668.938448	3844.244613	70.57%
Deaths	World Bank Low Income	Both	Age-standardized	Aortic aneurysm	Rate	1990	2.45006486	3.252790249	1.495995841	
Deaths	World Bank Low Income	Both	Age-standardized	Aortic aneurysm	Rate	2019	1.94660267	2.421435732	1.426906172	-20.55%
DALYs	World Bank Low Income	Female	All Ages	Aortic aneurysm	Number	1990	27106.505	41484.7204	16263.53077	
DALYs	World Bank Low Income	Female	All Ages	Aortic aneurysm	Number	2019	44779.3185	55460.58666	32135.1546	65.20%
DALYs	World Bank Low Income	Female	Age-standardized	Aortic aneurysm	Rate	1990	35.5159711	50.77766136	21.7550242	
DALYs	World Bank Low Income	Female	Age-standardized	Aortic aneurysm	Rate	2019	26.4391425	32.46525127	19.1133629	-25.56%
Deaths	World Bank Low Income	Female	All Ages	Aortic aneurysm	Number	1990	1142.75308	1597.786279	703.6356239	
Deaths	World Bank Low Income	Female	All Ages	Aortic aneurysm	Number	2019	1970.86971	2400.852109	1425.678491	72.47%
Deaths	World Bank Low Income	Female	Age-standardized	Aortic aneurysm	Rate	1990	1.83912797	2.406747428	1.156259152	
Deaths	World Bank Low Income	Female	Age-standardized	Aortic aneurysm	Rate	2019	1.38970004	1.687995683	1.007237505	-24.44%
DALYs	World Bank Low Income	Male	All Ages	Aortic aneurysm	Number	1990	51572.918	81280.2302	28716.59522	
DALYs	World Bank Low Income	Male	All Ages	Aortic aneurysm	Number	2019	86998.2745	118908.4672	58102.08963	68.69%
DALYs	World Bank Low Income	Male	Age-standardized	Aortic aneurysm	Rate	1990	67.6202231	104.7218069	37.43847021	
DALYs	World Bank Low Income	Male	Age-standardized	Aortic aneurysm	Rate	2019	55.0030151	74.10007249	36.7383983	-18.66%
Deaths	World Bank Low Income	Male	All Ages	Aortic aneurysm	Number	1990	1953.40416	3010.566024	1077.024908	
Deaths	World Bank Low Income	Male	All Ages	Aortic aneurysm	Number	2019	3310.16226	4454.619473	2207.324064	69.46%
Deaths	World Bank Low Income	Male	Age-standardized	Aortic aneurysm	Rate	1990	3.10968393	4.682515706	1.693460889	
Deaths	World Bank Low Income	Male	Age-standardized	Aortic aneurysm	Rate	2019	2.61797768	3.44969059	1.744736041	-15.81%
DALYs	World Bank Lower Middle Income	Both	All Ages	Aortic aneurysm	Number	1990	322234.64	437708.5891	233412.3946	
DALYs	World Bank Lower Middle Income	Both	All Ages	Aortic aneurysm	Number	2019	794568.682	915193.1483	665807.4343	146.58%
DALYs	World Bank Lower Middle Income	Both	Age-standardized	Aortic aneurysm	Rate	1990	30.9335965	41.40253784	22.65533798	
DALYs	World Bank Lower Middle Income	Both	Age-standardized	Aortic aneurysm	Rate	2019	33.5012971	38.34734748	28.19755205	8.30%
Deaths	World Bank Lower Middle Income	Both	All Ages	Aortic aneurysm	Number	1990	13227.1514	17607.91153	9715.116529	
Deaths	World Bank Lower Middle Income	Both	All Ages	Aortic aneurysm	Number	2019	34943.7744	39864.44068	29578.64632	164.18%
Deaths	World Bank Lower Middle Income	Both	Age-standardized	Aortic aneurysm	Rate	1990	1.54623556	2.022307554	1.148182124	
Deaths	World Bank Lower Middle Income	Both	Age-standardized	Aortic aneurysm	Rate	2019	1.70660411	1.933303275	1.452258553	10.37%
DALYs	World Bank Lower Middle Income	Female	All Ages	Aortic aneurysm	Number	1990	96804.2731	159913.2185	79501.7837	
DALYs	World Bank Lower Middle Income	Female	All Ages	Aortic aneurysm	Number	2019	242438.997	294478.8718	201887.843	150.44%

DALYs	World Bank Lower Middle Income	Female	Age-standardized	Aortic aneurysm	Rate	1990	19.0262314	29.97444963	15.64868926	
DALYs	World Bank Lower Middle Income	Female	Age-standardized	Aortic aneurysm	Rate	2019	20.1859542	24.30718443	16.79485375	6.10%
Deaths	World Bank Lower Middle Income	Female	All Ages	Aortic aneurysm	Number	1990	4298.19003	6610.228969	3534.898103	
Deaths	World Bank Lower Middle Income	Female	All Ages	Aortic aneurysm	Number	2019	11601.3198	13788.84201	9585.371141	169.91%
Deaths	World Bank Lower Middle Income	Female	Age-standardized	Aortic aneurysm	Rate	1990	1.02584452	1.49172392	0.84085242	
Deaths	World Bank Lower Middle Income	Female	Age-standardized	Aortic aneurysm	Rate	2019	1.10620468	1.297977544	0.915634775	7.83%
DALYs	World Bank Lower Middle Income	Male	All Ages	Aortic aneurysm	Number	1990	225430.367	339328.7984	146212.6599	
DALYs	World Bank Lower Middle Income	Male	All Ages	Aortic aneurysm	Number	2019	552129.685	667926.2284	429141.3819	144.92%
DALYs	World Bank Lower Middle Income	Male	Age-standardized	Aortic aneurysm	Rate	1990	43.2891277	64.47459037	27.87534393	
DALYs	World Bank Lower Middle Income	Male	Age-standardized	Aortic aneurysm	Rate	2019	47.9581612	57.76437111	37.23712231	10.79%
Deaths	World Bank Lower Middle Income	Male	All Ages	Aortic aneurysm	Number	1990	8928.96139	13294.09321	5726.757874	
Deaths	World Bank Lower Middle Income	Male	All Ages	Aortic aneurysm	Number	2019	23342.4546	27995.99068	18186.41664	161.42%
Deaths	World Bank Lower Middle Income	Male	Age-standardized	Aortic aneurysm	Rate	1990	2.11606665	3.106580841	1.353663138	
Deaths	World Bank Lower Middle Income	Male	Age-standardized	Aortic aneurysm	Rate	2019	2.39265678	2.868053911	1.881530569	13.07%
DALYs	World Bank Upper Middle Income	Both	All Ages	Aortic aneurysm	Number	1990	567630.812	633966.4148	514595.5177	
DALYs	World Bank Upper Middle Income	Both	All Ages	Aortic aneurysm	Number	2019	1177024.62	1280491.799	1077689.619	107.36%
DALYs	World Bank Upper Middle Income	Both	Age-standardized	Aortic aneurysm	Rate	1990	36.1919047	40.12002399	32.9100844	
DALYs	World Bank Upper Middle Income	Both	Age-standardized	Aortic aneurysm	Rate	2019	35.379196	38.46747384	32.38135513	-2.25%
Deaths	World Bank Upper Middle Income	Both	All Ages	Aortic aneurysm	Number	1990	22843.7111	25237.24481	20866.30368	
Deaths	World Bank Upper Middle Income	Both	All Ages	Aortic aneurysm	Number	2019	54500.0204	59052.40044	49818.43141	138.58%
Deaths	World Bank Upper Middle Income	Both	Age-standardized	Aortic aneurysm	Rate	1990	1.69937589	1.870024172	1.55196618	
Deaths	World Bank Upper Middle Income	Both	Age-standardized	Aortic aneurysm	Rate	2019	1.72673386	1.867251038	1.571724553	1.61%
DALYs	World Bank Upper Middle Income	Female	All Ages	Aortic aneurysm	Number	1990	172904.606	207056.3845	158922.5699	
DALYs	World Bank Upper Middle Income	Female	All Ages	Aortic aneurysm	Number	2019	359167.761	390261.6901	326329.5123	107.73%
DALYs	World Bank Upper Middle Income	Female	Age-standardized	Aortic aneurysm	Rate	1990	21.210063	25.10146027	19.52357544	
DALYs	World Bank Upper Middle Income	Female	Age-standardized	Aortic aneurysm	Rate	2019	20.4050301	22.17398823	18.48616112	-3.80%
Deaths	World Bank Upper Middle Income	Female	All Ages	Aortic aneurysm	Number	1990	7670.39377	8941.52051	7033.501349	
Deaths	World Bank Upper Middle Income	Female	All Ages	Aortic aneurysm	Number	2019	18687.9795	20315.64169	16656.13677	143.64%
Deaths	World Bank Upper Middle Income	Female	Age-standardized	Aortic aneurysm	Rate	1990	1.06267385	1.217930506	0.961794431	
Deaths	World Bank Upper Middle Income	Female	Age-standardized	Aortic aneurysm	Rate	2019	1.07865819	1.173242707	0.961861626	1.50%
DALYs	World Bank Upper Middle Income	Male	All Ages	Aortic aneurysm	Number	1990	394726.206	457019.0423	343393.6283	
DALYs	World Bank Upper Middle Income	Male	All Ages	Aortic aneurysm	Number	2019	817856.862	906695.6322	736558.1729	107.20%
DALYs	World Bank Upper Middle Income	Male	Age-standardized	Aortic aneurysm	Rate	1990	53.7769727	61.77736524	47.05681748	
DALYs	World Bank Upper Middle Income	Male	Age-standardized	Aortic aneurysm	Rate	2019	52.2724239	57.7482832	47.11992281	-2.80%
Deaths	World Bank Upper Middle Income	Male	All Ages	Aortic aneurysm	Number	1990	15173.3173	17385.36347	13288.61537	
Deaths	World Bank Upper Middle Income	Male	All Ages	Aortic aneurysm	Number	2019	35812.0409	39420.63391	32262.93577	136.02%
Deaths	World Bank Upper Middle Income	Male	Age-standardized	Aortic aneurysm	Rate	1990	2.55308278	2.891396485	2.235726825	
Deaths	World Bank Upper Middle Income	Male	Age-standardized	Aortic aneurysm	Rate	2019	2.54295492	2.794729922	2.290525251	-0.40%
