# Supplementary material for: Burden of Aortic Aneurysm and Its Attributable Risk Factors from 1990 to 2019: An Analysis of the Global Burden of Disease Study 2019
Source: Front Cardiovasc Med. 2022 May 31;9:901225. doi: 10.3389/fcvm.2022.901225 (PMC9197430; doi:10.3389/fcvm.2022.901225)
Supplement: Supplementary Table 4 — Global burden of aortic aneurysm in 204 countries and territories in 1990 and 2019. Changes mean increasing times from 1990 to 2019. DALY, disability-adjusted life year rate. [file Data_Sheet_4.PDF]

measure	location	sex	age	cause	metric	year	val	upper	lower	change(1990-2019)
DALYs	Afghanistan	Both	Age-standardized	Aortic aneurysm	Rate	1990	22.95420099	42.53166728	11.92319893	
DALYs	Afghanistan	Both	Age-standardized	Aortic aneurysm	Rate	2019	21.21082143	35.22023842	12.88084407	-7.60%
DALYs	Afghanistan	Both	All Ages	Aortic aneurysm	Number	1990	1666.977132	3134.126639	855.2911212	
DALYs	Afghanistan	Both	All Ages	Aortic aneurysm	Number	2019	3148.024099	5394.377596	1799.165263	88.85%
Deaths	Afghanistan	Both	Age-standardized	Aortic aneurysm	Rate	1990	1.049052939	1.856861325	0.570691088	
Deaths	Afghanistan	Both	Age-standardized	Aortic aneurysm	Rate	2019	0.998461997	1.581810408	0.638365746	-4.82%
Deaths	Afghanistan	Both	All Ages	Aortic aneurysm	Number	1990	66.28991317	120.2147945	34.80447123	
Deaths	Afghanistan	Both	All Ages	Aortic aneurysm	Number	2019	109.0298778	180.8421812	66.44636002	64.47%
DALYs	Albania	Both	Age-standardized	Aortic aneurysm	Rate	1990	24.59100411	26.80254567	22.46400597	
DALYs	Albania	Both	Age-standardized	Aortic aneurysm	Rate	2019	23.00299479	30.80715224	17.23853215	-6.46%
DALYs	Albania	Both	All Ages	Aortic aneurysm	Number	1990	543.6421845	593.6874312	496.1380035	
DALYs	Albania	Both	All Ages	Aortic aneurysm	Number	2019	926.4748583	1240.4121	688.3837351	70.42%
Deaths	Albania	Both	Age-standardized	Aortic aneurysm	Rate	1990	1.187680398	1.30648985	1.079018892	
Deaths	Albania	Both	Age-standardized	Aortic aneurysm	Rate	2019	1.148209339	1.506951768	0.874791574	-3.32%
Deaths	Albania	Both	All Ages	Aortic aneurysm	Number	1990	22.52173202	24.67003027	20.5281886	
Deaths	Albania	Both	All Ages	Aortic aneurysm	Number	2019	48.05344714	63.01327678	36.57509534	113.36%
DALYs	Algeria	Both	Age-standardized	Aortic aneurysm	Rate	1990	25.25322343	34.95498301	17.26944253	
DALYs	Algeria	Both	Age-standardized	Aortic aneurysm	Rate	2019	21.121957	29.11153769	15.32665404	-16.36%
DALYs	Algeria	Both	All Ages	Aortic aneurysm	Number	1990	3110.098837	4323.13357	2084.964481	
DALYs	Algeria	Both	All Ages	Aortic aneurysm	Number	2019	7037.720118	9855.366811	5046.121765	126.29%
Deaths	Algeria	Both	Age-standardized	Aortic aneurysm	Rate	1990	1.360866382	1.85873414	0.944872954	
Deaths	Algeria	Both	Age-standardized	Aortic aneurysm	Rate	2019	1.158847146	1.5325795	0.876199288	-14.84%
Deaths	Algeria	Both	All Ages	Aortic aneurysm	Number	1990	123.9544635	170.8948311	84.89478498	
Deaths	Algeria	Both	All Ages	Aortic aneurysm	Number	2019	318.4268936	430.8635189	235.0342542	156.89%
DALYs	American Samoa	Both	Age-standardized	Aortic aneurysm	Rate	1990	87.07523498	100.5625049	74.35781334	
DALYs	American Samoa	Both	Age-standardized	Aortic aneurysm	Rate	2019	48.7637121	57.71836534	40.27053026	-44.00%
DALYs	American Samoa	Both	All Ages	Aortic aneurysm	Number	1990	20.00183533	23.37663254	16.99248082	
DALYs	American Samoa	Both	All Ages	Aortic aneurysm	Number	2019	22.85360959	27.28643837	18.73638095	14.26%
Deaths	American Samoa	Both	Age-standardized	Aortic aneurysm	Rate	1990	4.606296623	5.297119655	3.938500973	
Deaths	American Samoa	Both	Age-standardized	Aortic aneurysm	Rate	2019	2.514045325	2.939426917	2.1245555	-45.42%
Deaths	American Samoa	Both	All Ages	Aortic aneurysm	Number	1990	0.806213764	0.928844583	0.687835792	
Deaths	American Samoa	Both	All Ages	Aortic aneurysm	Number	2019	1.034722597	1.210581972	0.874044361	28.34%
DALYs	Andorra	Both	Age-standardized	Aortic aneurysm	Rate	1990	118.0707243	183.3380344	81.73246127	
DALYs	Andorra	Both	Age-standardized	Aortic aneurysm	Rate	2019	94.15593812	134.0524238	64.97099345	-20.25%
DALYs	Andorra	Both	All Ages	Aortic aneurysm	Number	1990	64.85169918	102.391856	44.33734975	
DALYs	Andorra	Both	All Ages	Aortic aneurysm	Number	2019	130.7097097	185.1593033	90.63083154	101.55%
Deaths	Andorra	Both	Age-standardized	Aortic aneurysm	Rate	1990	6.633136837	9.91247229	4.685935011	
Deaths	Andorra	Both	Age-standardized	Aortic aneurysm	Rate	2019	5.543449312	7.633035867	3.881449961	-16.43%
Deaths	Andorra	Both	All Ages	Aortic aneurysm	Number	1990	3.228806631	4.970648158	2.25344205	
Deaths	Andorra	Both	All Ages	Aortic aneurysm	Number	2019	8.15882232	11.1734285	5.736860946	152.69%
DALYs	Angola	Both	Age-standardized	Aortic aneurysm	Rate	1990	74.2481952	109.4391645	42.97214853	
DALYs	Angola	Both	Age-standardized	Aortic aneurysm	Rate	2019	61.21498307	78.51981116	45.86136468	-17.55%
DALYs	Angola	Both	All Ages	Aortic aneurysm	Number	1990	3031.135309	4689.057091	1694.898709	
DALYs	Angola	Both	All Ages	Aortic aneurysm	Number	2019	7143.454348	9341.431252	5161.611171	135.67%
Deaths	Angola	Both	Age-standardized	Aortic aneurysm	Rate	1990	3.483098075	4.967344363	2.075600461	
Deaths	Angola	Both	Age-standardized	Aortic aneurysm	Rate	2019	3.005925942	3.772670154	2.289646251	-13.70%
Deaths	Angola	Both	All Ages	Aortic aneurysm	Number	1990	112.0794692	166.27291	64.53948156	
Deaths	Angola	Both	All Ages	Aortic aneurysm	Number	2019	270.6808703	348.3123122	202.2042734	141.51%
DALYs	Antigua and Barbuda	Both	Age-standardized	Aortic aneurysm	Rate	1990	50.44831971	56.48450664	44.94444744	
DALYs	Antigua and Barbuda	Both	Age-standardized	Aortic aneurysm	Rate	2019	44.17297228	51.984371	36.81852363	-12.44%
DALYs	Antigua and Barbuda	Both	All Ages	Aortic aneurysm	Number	1990	27.45912114	30.82245594	24.38689204	
DALYs	Antigua and Barbuda	Both	All Ages	Aortic aneurysm	Number	2019	43.1740757	50.92557987	35.85665185	57.23%
Deaths	Antigua and Barbuda	Both	Age-standardized	Aortic aneurysm	Rate	1990	2.695851126	3.042436183	2.373540021	
Deaths	Antigua and Barbuda	Both	Age-standardized	Aortic aneurysm	Rate	2019	2.560000643	2.973300731	2.149245079	-5.04%
Deaths	Antigua and Barbuda	Both	All Ages	Aortic aneurysm	Number	1990	1.536239039	1.73921004	1.342014621	
Deaths	Antigua and Barbuda	Both	All Ages	Aortic aneurysm	Number	2019	2.308790654	2.704736169	1.933900742	50.29%
DALYs	Argentina	Both	Age-standardized	Aortic aneurysm	Rate	1990	86.20806327	95.25618525	76.69960188	
DALYs	Argentina	Both	Age-standardized	Aortic aneurysm	Rate	2019	69.48317209	75.95964921	63.23541643	-19.40%
DALYs	Argentina	Both	All Ages	Aortic aneurysm	Number	1990	28052.10281	31093.7171	24878.19501	
DALYs	Argentina	Both	All Ages	Aortic aneurysm	Number	2019	37128.97717	40599.21415	33757.05576	32.36%
Deaths	Argentina	Both	Age-standardized	Aortic aneurysm	Rate	1990	4.213014854	4.706141467	3.717322729	
Deaths	Argentina	Both	Age-standardized	Aortic aneurysm	Rate	2019	3.469615247	3.811192524	3.122873638	-17.65%
Deaths	Argentina	Both	All Ages	Aortic aneurysm	Number	1990	1327.142763	1481.331541	1171.166128	
Deaths	Argentina	Both	All Ages	Aortic aneurysm	Number	2019	1908.767211	2105.392254	1715.650039	43.83%
DALYs	Armenia	Both	Age-standardized	Aortic aneurysm	Rate	1990	100.0723324	112.6446456	89.20020704	
DALYs	Armenia	Both	Age-standardized	Aortic aneurysm	Rate	2019	170.5875985	203.0042315	141.9371586	70.46%
DALYs	Armenia	Both	All Ages	Aortic aneurysm	Number	1990	2733.769647	3084.651156	2427.767709	
DALYs	Armenia	Both	All Ages	Aortic aneurysm	Number	2019	7032.625218	8381.697852	5854.78903	157.25%
Deaths	Armenia	Both	Age-standardized	Aortic aneurysm	Rate	1990	4.76581274	5.390633239	4.257174456	
Deaths	Armenia	Both	Age-standardized	Aortic aneurysm	Rate	2019	8.745465994	10.28982925	7.336752262	83.50%
Deaths	Armenia	Both	All Ages	Aortic aneurysm	Number	1990	115.8380122	130.5637204	103.2051895	
Deaths	Armenia	Both	All Ages	Aortic aneurysm	Number	2019	356.4604492	421.5047887	299.4013909	207.72%
DALYs	Australia	Both	Age-standardized	Aortic aneurysm	Rate	1990	127.1721251	133.7097007	120.2342324	
DALYs	Australia	Both	Age-standardized	Aortic aneurysm	Rate	2019	47.06678626	51.63836796	41.59157915	-62.99%
DALYs	Australia	Both	All Ages	Aortic aneurysm	Number	1990	25403.67091	26732.17214	24002.94296	
DALYs	Australia	Both	All Ages	Aortic aneurysm	Number	2019	19872.91454	21872.91738	17413.40567	-21.77%
Deaths	Australia	Both	Age-standardized	Aortic aneurysm	Rate	1990	7.260671909	7.66141774	6.736237699	
Deaths	Australia	Both	Age-standardized	Aortic aneurysm	Rate	2019	2.940806805	3.264328171	2.536698846	-59.50%
Deaths	Australia	Both	All Ages	Aortic aneurysm	Number	1990	1424.717878	1504.045456	1327.254443	
Deaths	Australia	Both	All Ages	Aortic aneurysm	Number	2019	1354.120199	1508.777711	1159.930308	-4.96%
DALYs	Austria	Both	Age-standardized	Aortic aneurysm	Rate	1990	65.73532822	69.91410274	62.38889348	
DALYs	Austria	Both	Age-standardized	Aortic aneurysm	Rate	2019	44.07501565	48.13452736	40.40744027	-32.95%
DALYs	Austria	Both	All Ages	Aortic aneurysm	Number	1990	7391.770065	7894.862811	7000.189513	
DALYs	Austria	Both	All Ages	Aortic aneurysm	Number	2019	7229.710948	7908.113847	6562.402542	-2.19%
Deaths	Austria	Both	Age-standardized	Aortic aneurysm	Rate	1990	3.100830864	3.349794069	2.900076387	
Deaths	Austria	Both	Age-standardized	Aortic aneurysm	Rate	2019	2.215213989	2.439955911	1.989121252	-28.56%
Deaths	Austria	Both	All Ages	Aortic aneurysm	Number	1990	376.2414995	406.819695	350.4637388	
Deaths	Austria	Both	All Ages	Aortic aneurysm	Number	2019	420.493867	467.2618877	373.1368638	11.76%
DALYs	Azerbaijan	Both	Age-standardized	Aortic aneurysm	Rate	1990	13.67408863	15.49879304	11.79466991	
DALYs	Azerbaijan	Both	Age-standardized	Aortic aneurysm	Rate	2019	17.08812776	20.5403791	14.22168985	24.97%
DALYs	Azerbaijan	Both	All Ages	Aortic aneurysm	Number	1990	702.9533843	801.9898072	613.5462817	
DALYs	Azerbaijan	Both	All Ages	Aortic aneurysm	Number	2019	1529.075951	1876.809491	1241.74614	117.52%
Deaths	Azerbaijan	Both	Age-standardized	Aortic aneurysm	Rate	1990	0.660007873	0.751269384	0.532876053	
Deaths	Azerbaijan	Both	Age-standardized	Aortic aneurysm	Rate	2019	0.919722592	1.072233466	0.782173383	39.35%
Deaths	Azerbaijan	Both	All Ages	Aortic aneurysm	Number	1990	30.51629302	34.70050434	25.02157925	
Deaths	Azerbaijan	Both	All Ages	Aortic aneurysm	Number	2019	64.5457124	77.55232951	53.99267925	111.51%
DALYs	Bahamas	Both	Age-standardized	Aortic aneurysm	Rate	1990	75.14997653	85.01868253	66.32025922	
DALYs	Bahamas	Both	Age-standardized	Aortic aneurysm	Rate	2019	75.86987539	94.91000794	61.42645399	0.96%
DALYs	Bahamas	Both	All Ages	Aortic aneurysm	Number	1990	118.1411672	133.3260744	105.0422062	
DALYs	Bahamas	Both	All Ages	Aortic aneurysm	Number	2019	292.8905262	368.8154134	236.1750467	147.92%

Deaths	Bahamas	Both	Age-standardized	Aortic aneurysm	Rate	1990	3.79995927	4.311765193	3.329998292	
Deaths	Bahamas	Both	Age-standardized	Aortic aneurysm	Rate	2019	3.874072501	4.755610205	3.148469081	1.95%
Deaths	Bahamas	Both	All Ages	Aortic aneurysm	Number	1990	5.313781001	6.050276423	4.679336406	
Deaths	Bahamas	Both	All Ages	Aortic aneurysm	Number	2019	13.57180343	16.78137417	11.05558202	155.41%
DALYs	Bahrain	Both	Age-standardized	Aortic aneurysm	Rate	1990	31.61246253	38.42162978	25.90392881	
DALYs	Bahrain	Both	Age-standardized	Aortic aneurysm	Rate	2019	24.92443061	31.79345281	19.2032825	-21.16%
DALYs	Bahrain	Both	All Ages	Aortic aneurysm	Number	1990	63.4881215	77.11690354	52.07226111	
DALYs	Bahrain	Both	All Ages	Aortic aneurysm	Number	2019	241.2591965	318.4584395	180.8636985	280.01%
Deaths	Bahrain	Both	Age-standardized	Aortic aneurysm	Rate	1990	1.636067907	1.957714887	1.342919481	
Deaths	Bahrain	Both	Age-standardized	Aortic aneurysm	Rate	2019	1.460404226	1.833179826	1.138588935	-10.74%
Deaths	Bahrain	Both	All Ages	Aortic aneurysm	Number	1990	2.244585148	2.738272616	1.840489635	
Deaths	Bahrain	Both	All Ages	Aortic aneurysm	Number	2019	8.941659394	11.60320893	6.824052419	298.37%
DALYs	Bangladesh	Both	Age-standardized	Aortic aneurysm	Rate	1990	29.21949091	45.44086884	17.59190804	
DALYs	Bangladesh	Both	Age-standardized	Aortic aneurysm	Rate	2019	30.0604649	39.92998225	21.80201505	2.88%
DALYs	Bangladesh	Both	All Ages	Aortic aneurysm	Number	1990	14064.32443	22252.61213	8360.373247	
DALYs	Bangladesh	Both	All Ages	Aortic aneurysm	Number	2019	38761.59444	51457.90006	28149.30431	175.60%
Deaths	Bangladesh	Both	Age-standardized	Aortic aneurysm	Rate	1990	1.480207967	2.243229581	0.910230971	
Deaths	Bangladesh	Both	Age-standardized	Aortic aneurysm	Rate	2019	1.641894801	2.173277401	1.173342715	10.92%
Deaths	Bangladesh	Both	All Ages	Aortic aneurysm	Number	1990	595.6648042	915.6487869	361.1023258	
Deaths	Bangladesh	Both	All Ages	Aortic aneurysm	Number	2019	1877.677923	2497.388153	1346.299192	215.22%
DALYs	Barbados	Both	Age-standardized	Aortic aneurysm	Rate	1990	47.06378515	52.61580764	41.96408403	
DALYs	Barbados	Both	Age-standardized	Aortic aneurysm	Rate	2019	49.24972382	59.84756173	39.69723636	4.64%
DALYs	Barbados	Both	All Ages	Aortic aneurysm	Number	1990	139.6336588	157.0776227	122.9660443	
DALYs	Barbados	Both	All Ages	Aortic aneurysm	Number	2019	240.9174728	292.7750981	193.9358733	72.54%
Deaths	Barbados	Both	Age-standardized	Aortic aneurysm	Rate	1990	2.538982588	2.853032572	2.242336546	
Deaths	Barbados	Both	Age-standardized	Aortic aneurysm	Rate	2019	2.761285346	3.303008758	2.256261903	8.76%
Deaths	Barbados	Both	All Ages	Aortic aneurysm	Number	1990	7.998209848	9.082661766	7.0014285	
Deaths	Barbados	Both	All Ages	Aortic aneurysm	Number	2019	13.72037817	16.42155916	11.19619305	71.54%
DALYs	Belarus	Both	Age-standardized	Aortic aneurysm	Rate	1990	34.18391654	43.51569604	29.02856925	
DALYs	Belarus	Both	Age-standardized	Aortic aneurysm	Rate	2019	54.46473926	71.55243873	41.46119141	59.33%
DALYs	Belarus	Both	All Ages	Aortic aneurysm	Number	1990	4397.052149	5618.634089	3724.919124	
DALYs	Belarus	Both	All Ages	Aortic aneurysm	Number	2019	8287.927622	10845.14757	6300.532561	88.49%
Deaths	Belarus	Both	Age-standardized	Aortic aneurysm	Rate	1990	1.488566801	1.840805442	1.276324132	
Deaths	Belarus	Both	Age-standardized	Aortic aneurysm	Rate	2019	2.395226156	3.110000637	1.854566749	60.91%
Deaths	Belarus	Both	All Ages	Aortic aneurysm	Number	1990	190.2852238	235.6242491	162.7892943	
Deaths	Belarus	Both	All Ages	Aortic aneurysm	Number	2019	381.5499142	491.4105517	295.0368065	100.51%
DALYs	Belgium	Both	Age-standardized	Aortic aneurysm	Rate	1990	80.59258465	86.29732858	75.15353513	
DALYs	Belgium	Both	Age-standardized	Aortic aneurysm	Rate	2019	47.29254507	51.8847288	42.82197425	-41.32%
DALYs	Belgium	Both	All Ages	Aortic aneurysm	Number	1990	12370.43921	13286.66105	11511.05129	
DALYs	Belgium	Both	All Ages	Aortic aneurysm	Number	2019	10780.88804	11877.33101	9668.162444	-12.85%
Deaths	Belgium	Both	Age-standardized	Aortic aneurysm	Rate	1990	4.381734473	4.696446812	4.06317769	
Deaths	Belgium	Both	Age-standardized	Aortic aneurysm	Rate	2019	2.793957664	3.079755738	2.494018686	-36.24%
Deaths	Belgium	Both	All Ages	Aortic aneurysm	Number	1990	699.7253295	752.3243934	646.7757999	
Deaths	Belgium	Both	All Ages	Aortic aneurysm	Number	2019	734.9279956	817.6079061	645.1537146	5.03%
DALYs	Belize	Both	Age-standardized	Aortic aneurysm	Rate	1990	27.05982592	34.92770296	22.28193739	
DALYs	Belize	Both	Age-standardized	Aortic aneurysm	Rate	2019	31.88101745	37.58368641	26.65595786	17.82%
DALYs	Belize	Both	All Ages	Aortic aneurysm	Number	1990	25.5528994	32.95968403	20.94259675	
DALYs	Belize	Both	All Ages	Aortic aneurysm	Number	2019	87.50717073	103.4319203	73.25339768	242.45%
Deaths	Belize	Both	Age-standardized	Aortic aneurysm	Rate	1990	1.434590022	1.848866238	1.177751371	
Deaths	Belize	Both	Age-standardized	Aortic aneurysm	Rate	2019	1.683667043	1.98045798	1.401672354	17.36%
Deaths	Belize	Both	All Ages	Aortic aneurysm	Number	1990	1.287833921	1.661364805	1.058415859	
Deaths	Belize	Both	All Ages	Aortic aneurysm	Number	2019	4.130201376	4.84491188	3.460748048	220.71%
DALYs	Benin	Both	Age-standardized	Aortic aneurysm	Rate	1990	41.22364686	53.99869343	27.05686242	
DALYs	Benin	Both	Age-standardized	Aortic aneurysm	Rate	2019	31.36323722	41.19661774	22.22676068	-23.92%
DALYs	Benin	Both	All Ages	Aortic aneurysm	Number	1990	853.3154173	1118.637429	555.4845405	
DALYs	Benin	Both	All Ages	Aortic aneurysm	Number	2019	1622.060495	2173.887478	1122.619394	90.09%
Deaths	Benin	Both	Age-standardized	Aortic aneurysm	Rate	1990	1.998234914	2.573494881	1.315973179	
Deaths	Benin	Both	Age-standardized	Aortic aneurysm	Rate	2019	1.548456	2.003465796	1.113491875	-22.51%
Deaths	Benin	Both	All Ages	Aortic aneurysm	Number	1990	36.93508925	48.17931277	24.45155187	
Deaths	Benin	Both	All Ages	Aortic aneurysm	Number	2019	66.05867065	86.08433589	47.51553683	78.85%
DALYs	Bermuda	Both	Age-standardized	Aortic aneurysm	Rate	1990	161.1259517	185.5550135	128.2163859	
DALYs	Bermuda	Both	Age-standardized	Aortic aneurysm	Rate	2019	95.78972021	116.6053817	79.29420841	-40.55%
DALYs	Bermuda	Both	All Ages	Aortic aneurysm	Number	1990	99.68841294	115.0008764	78.99479494	
DALYs	Bermuda	Both	All Ages	Aortic aneurysm	Number	2019	125.3828845	152.5417081	103.3814362	25.77%
Deaths	Bermuda	Both	Age-standardized	Aortic aneurysm	Rate	1990	8.887080278	10.25577337	7.114782966	
Deaths	Bermuda	Both	Age-standardized	Aortic aneurysm	Rate	2019	5.700850536	6.936494799	4.705164441	-35.85%
Deaths	Bermuda	Both	All Ages	Aortic aneurysm	Number	1990	5.200655219	6.025309234	4.135990814	
Deaths	Bermuda	Both	All Ages	Aortic aneurysm	Number	2019	7.934637988	9.681497572	6.532244642	52.57%
DALYs	Bhutan	Both	Age-standardized	Aortic aneurysm	Rate	1990	28.87188626	48.8802224	15.67332362	
DALYs	Bhutan	Both	Age-standardized	Aortic aneurysm	Rate	2019	38.54302671	52.68481781	25.82065358	33.50%
DALYs	Bhutan	Both	All Ages	Aortic aneurysm	Number	1990	71.38652996	126.2410364	36.33787606	
DALYs	Bhutan	Both	All Ages	Aortic aneurysm	Number	2019	212.7211486	295.1419368	140.3421555	197.98%
Deaths	Bhutan	Both	Age-standardized	Aortic aneurysm	Rate	1990	1.4571169	2.356068976	0.843274404	
Deaths	Bhutan	Both	Age-standardized	Aortic aneurysm	Rate	2019	2.146072025	2.896420924	1.457063711	47.28%
Deaths	Bhutan	Both	All Ages	Aortic aneurysm	Number	1990	2.849940656	4.814955243	1.54627301	
Deaths	Bhutan	Both	All Ages	Aortic aneurysm	Number	2019	10.56312659	14.23524666	7.111668888	270.64%
DALYs	Bolivia (Plurinational State of)	Both	Age-standardized	Aortic aneurysm	Rate	1990	38.19819611	53.50668886	25.90907236	
DALYs	Bolivia (Plurinational State of)	Both	Age-standardized	Aortic aneurysm	Rate	2019	40.66281989	55.26876976	27.29936701	6.45%
DALYs	Bolivia (Plurinational State of)	Both	All Ages	Aortic aneurysm	Number	1990	1272.695336	1791.38625	857.2650296	
DALYs	Bolivia (Plurinational State of)	Both	All Ages	Aortic aneurysm	Number	2019	3556.993819	4869.814326	2378.168086	179.49%
Deaths	Bolivia (Plurinational State of)	Both	Age-standardized	Aortic aneurysm	Rate	1990	1.895746626	2.581926092	1.337973886	
Deaths	Bolivia (Plurinational State of)	Both	Age-standardized	Aortic aneurysm	Rate	2019	2.212714846	2.909561632	1.514502863	16.72%
Deaths	Bolivia (Plurinational State of)	Both	All Ages	Aortic aneurysm	Number	1990	52.99959342	73.42389597	36.05266574	
Deaths	Bolivia (Plurinational State of)	Both	All Ages	Aortic aneurysm	Number	2019	169.8638693	226.2491354	114.5867088	220.50%
DALYs	Bosnia and Herzegovina	Both	Age-standardized	Aortic aneurysm	Rate	1990	58.65093401	70.58588509	48.79850354	
DALYs	Bosnia and Herzegovina	Both	Age-standardized	Aortic aneurysm	Rate	2019	72.94736371	90.39135921	58.10660384	24.38%
DALYs	Bosnia and Herzegovina	Both	All Ages	Aortic aneurysm	Number	1990	2442.644684	2983.278713	2007.343127	
DALYs	Bosnia and Herzegovina	Both	All Ages	Aortic aneurysm	Number	2019	4111.455624	5122.49484	3274.992613	68.32%
Deaths	Bosnia and Herzegovina	Both	Age-standardized	Aortic aneurysm	Rate	1990	2.783123378	3.268200307	2.338167742	
Deaths	Bosnia and Herzegovina	Both	Age-standardized	Aortic aneurysm	Rate	2019	3.75613131	4.596661966	3.04025027	34.96%
Deaths	Bosnia and Herzegovina	Both	All Ages	Aortic aneurysm	Number	1990	98.33252314	117.3947033	82.03769525	
Deaths	Bosnia and Herzegovina	Both	All Ages	Aortic aneurysm	Number	2019	212.1835041	258.8448334	170.4620401	115.78%
DALYs	Botswana	Both	Age-standardized	Aortic aneurysm	Rate	1990	58.57605845	77.60470223	43.21785941	
DALYs	Botswana	Both	Age-standardized	Aortic aneurysm	Rate	2019	47.00568867	64.25373945	33.38822145	-19.75%
DALYs	Botswana	Both	All Ages	Aortic aneurysm	Number	1990	347.5980678	470.4928934	254.4041713	
DALYs	Botswana	Both	All Ages	Aortic aneurysm	Number	2019	675.4008233	964.3455256	460.7225509	94.31%
Deaths	Botswana	Both	Age-standardized	Aortic aneurysm	Rate	1990	2.948625315	3.796332235	2.265726501	
Deaths	Botswana	Both	Age-standardized	Aortic aneurysm	Rate	2019	2.380803884	3.17457473	1.765797436	-19.26%
Deaths	Botswana	Both	All Ages	Aortic aneurysm	Number	1990	13.8507964	18.08875875	10.31051724	
Deaths	Botswana	Both	All Ages	Aortic aneurysm	Number	2019	26.51622377	36.12889982	19.02978202	91.44%
DALYs	Brazil	Both	Age-standardized	Aortic aneurysm	Rate	1990	79.66915076	82.83802482	76.36077572	

DALYs	Brazil	Both	Age-standardized	Aortic aneurysm	Rate	2019	97.01477674	102.9287454	90.6136429	21.77%
DALYs	Brazil	Both	All Ages	Aortic aneurysm	Number	1990	76726.00267	79785.07708	73846.32915	
DALYs	Brazil	Both	All Ages	Aortic aneurysm	Number	2019	231484.2932	245471.0612	216550.5088	201.70%
Deaths	Brazil	Both	Age-standardized	Aortic aneurysm	Rate	1990	3.45566906	3.613620781	3.273020624	
Deaths	Brazil	Both	Age-standardized	Aortic aneurysm	Rate	2019	4.576853239	4.891789598	4.183027226	32.44%
Deaths	Brazil	Both	All Ages	Aortic aneurysm	Number	1990	2909.313681	3032.983932	2777.458043	
Deaths	Brazil	Both	All Ages	Aortic aneurysm	Number	2019	10532.84888	11249.03913	9669.777022	262.04%
DALYs	Brunei Darussalam	Both	Age-standardized	Aortic aneurysm	Rate	1990	121.6945554	153.7768876	96.63900573	
DALYs	Brunei Darussalam	Both	Age-standardized	Aortic aneurysm	Rate	2019	115.8386379	131.6138511	101.8254081	-4.81%
DALYs	Brunei Darussalam	Both	All Ages	Aortic aneurysm	Number	1990	124.7516904	158.6524664	96.44477066	
DALYs	Brunei Darussalam	Both	All Ages	Aortic aneurysm	Number	2019	319.4120632	366.3474114	277.0220857	156.04%
Deaths	Brunei Darussalam	Both	Age-standardized	Aortic aneurysm	Rate	1990	6.541406824	8.292631634	5.286753151	
Deaths	Brunei Darussalam	Both	Age-standardized	Aortic aneurysm	Rate	2019	6.810178428	7.720227064	5.95430534	4.11%
Deaths	Brunei Darussalam	Both	All Ages	Aortic aneurysm	Number	1990	4.853055956	6.153866303	3.834531267	
Deaths	Brunei Darussalam	Both	All Ages	Aortic aneurysm	Number	2019	13.44114399	15.36552718	11.80580672	176.96%
DALYs	Bulgaria	Both	Age-standardized	Aortic aneurysm	Rate	1990	47.63258612	52.38103514	43.45020452	
DALYs	Bulgaria	Both	Age-standardized	Aortic aneurysm	Rate	2019	73.95320708	93.74038311	56.97341635	55.26%
DALYs	Bulgaria	Both	All Ages	Aortic aneurysm	Number	1990	5464.19275	6032.687469	4946.00786	
DALYs	Bulgaria	Both	All Ages	Aortic aneurysm	Number	2019	8619.269351	10925.81177	6675.18487	57.74%
Deaths	Bulgaria	Both	Age-standardized	Aortic aneurysm	Rate	1990	1.732299148	1.898319851	1.583953608	
Deaths	Bulgaria	Both	Age-standardized	Aortic aneurysm	Rate	2019	2.725214468	3.400937769	2.141562455	57.32%
Deaths	Bulgaria	Both	All Ages	Aortic aneurysm	Number	1990	197.8911235	218.4663842	179.1596941	
Deaths	Bulgaria	Both	All Ages	Aortic aneurysm	Number	2019	363.7549172	454.783658	286.7299665	83.82%
DALYs	Burkina Faso	Both	Age-standardized	Aortic aneurysm	Rate	1990	38.48127474	56.90627327	20.41796301	
DALYs	Burkina Faso	Both	Age-standardized	Aortic aneurysm	Rate	2019	36.56757944	53.43428253	22.64604299	-4.97%
DALYs	Burkina Faso	Both	All Ages	Aortic aneurysm	Number	1990	1702.7204	2546.237645	895.9184162	
DALYs	Burkina Faso	Both	All Ages	Aortic aneurysm	Number	2019	3370.871024	4985.356396	2065.659923	97.97%
Deaths	Burkina Faso	Both	Age-standardized	Aortic aneurysm	Rate	1990	1.883770541	2.775719559	1.00656396	
Deaths	Burkina Faso	Both	Age-standardized	Aortic aneurysm	Rate	2019	1.824622464	2.610934111	1.159569194	-3.14%
Deaths	Burkina Faso	Both	All Ages	Aortic aneurysm	Number	1990	71.2542296	105.9993471	37.56893414	
Deaths	Burkina Faso	Both	All Ages	Aortic aneurysm	Number	2019	141.6634406	205.3225044	88.85835593	98.81%
DALYs	Burundi	Both	Age-standardized	Aortic aneurysm	Rate	1990	78.32290944	132.2369602	41.66429597	
DALYs	Burundi	Both	Age-standardized	Aortic aneurysm	Rate	2019	41.13976599	60.66339349	22.96203737	-47.47%
DALYs	Burundi	Both	All Ages	Aortic aneurysm	Number	1990	1957.188036	3402.390963	1030.455311	
DALYs	Burundi	Both	All Ages	Aortic aneurysm	Number	2019	2015.420196	3031.948404	1110.642775	2.98%
Deaths	Burundi	Both	Age-standardized	Aortic aneurysm	Rate	1990	3.675005991	5.885534028	1.996698744	
Deaths	Burundi	Both	Age-standardized	Aortic aneurysm	Rate	2019	2.024851623	2.894787767	1.155704955	-44.90%
Deaths	Burundi	Both	All Ages	Aortic aneurysm	Number	1990	78.04802446	129.7882506	41.91437007	
Deaths	Burundi	Both	All Ages	Aortic aneurysm	Number	2019	76.03720757	111.5679995	42.63179216	-2.58%
DALYs	Cabo Verde	Both	Age-standardized	Aortic aneurysm	Rate	1990	21.83917606	27.10580474	16.78209045	
DALYs	Cabo Verde	Both	Age-standardized	Aortic aneurysm	Rate	2019	39.47060725	46.56634213	33.47761401	80.73%
DALYs	Cabo Verde	Both	All Ages	Aortic aneurysm	Number	1990	51.82691382	64.50360402	39.70769221	
DALYs	Cabo Verde	Both	All Ages	Aortic aneurysm	Number	2019	168.9882395	201.7766913	141.4560709	226.06%
Deaths	Cabo Verde	Both	Age-standardized	Aortic aneurysm	Rate	1990	1.048526558	1.297402682	0.795125842	
Deaths	Cabo Verde	Both	Age-standardized	Aortic aneurysm	Rate	2019	2.027081285	2.355412821	1.744488858	93.33%
Deaths	Cabo Verde	Both	All Ages	Aortic aneurysm	Number	1990	2.54794401	3.162352717	1.916905684	
Deaths	Cabo Verde	Both	All Ages	Aortic aneurysm	Number	2019	8.265616918	9.621253934	7.110009402	224.40%
DALYs	Cambodia	Both	Age-standardized	Aortic aneurysm	Rate	1990	19.88696283	29.39866004	13.39162372	
DALYs	Cambodia	Both	Age-standardized	Aortic aneurysm	Rate	2019	23.53854569	31.72551384	17.89303993	18.36%
DALYs	Cambodia	Both	All Ages	Aortic aneurysm	Number	1990	896.9600606	1372.299484	587.4403415	
DALYs	Cambodia	Both	All Ages	Aortic aneurysm	Number	2019	2738.043093	3725.269491	2067.032817	205.26%
Deaths	Cambodia	Both	Age-standardized	Aortic aneurysm	Rate	1990	1.018595941	1.466532353	0.70807167	
Deaths	Cambodia	Both	Age-standardized	Aortic aneurysm	Rate	2019	1.261524081	1.706158253	0.974722103	23.85%
Deaths	Cambodia	Both	All Ages	Aortic aneurysm	Number	1990	36.81960125	53.87520046	24.8874747	
Deaths	Cambodia	Both	All Ages	Aortic aneurysm	Number	2019	123.6435991	167.1427496	95.1475604	235.81%
DALYs	Cameroon	Both	Age-standardized	Aortic aneurysm	Rate	1990	40.82058675	56.55391455	27.44808859	
DALYs	Cameroon	Both	Age-standardized	Aortic aneurysm	Rate	2019	34.86320409	50.97818704	24.20887671	-14.59%
DALYs	Cameroon	Both	All Ages	Aortic aneurysm	Number	1990	1849.411967	2623.122559	1242.639504	
DALYs	Cameroon	Both	All Ages	Aortic aneurysm	Number	2019	4461.160461	6597.981121	2981.404824	141.22%
Deaths	Cameroon	Both	Age-standardized	Aortic aneurysm	Rate	1990	2.034762168	2.80606553	1.387756151	
Deaths	Cameroon	Both	Age-standardized	Aortic aneurysm	Rate	2019	1.713958939	2.451503639	1.226481801	-15.77%
Deaths	Cameroon	Both	All Ages	Aortic aneurysm	Number	1990	75.86153629	104.8171934	51.13341542	
Deaths	Cameroon	Both	All Ages	Aortic aneurysm	Number	2019	174.5091168	253.7861064	122.0338377	130.04%
DALYs	Canada	Both	Age-standardized	Aortic aneurysm	Rate	1990	109.6157678	115.4193335	103.7099935	
DALYs	Canada	Both	Age-standardized	Aortic aneurysm	Rate	2019	45.64832165	50.23081938	40.84538794	-58.36%
DALYs	Canada	Both	All Ages	Aortic aneurysm	Number	1990	36121.98776	38058.80022	34153.79294	
DALYs	Canada	Both	All Ages	Aortic aneurysm	Number	2019	31003.42434	34245.92453	27525.38507	-14.17%
Deaths	Canada	Both	Age-standardized	Aortic aneurysm	Rate	1990	6.22862173	6.598949112	5.788741398	
Deaths	Canada	Both	Age-standardized	Aortic aneurysm	Rate	2019	2.735896328	3.053569507	2.38723521	-56.08%
Deaths	Canada	Both	All Ages	Aortic aneurysm	Number	1990	2038.254483	2162.197915	1899.975567	
Deaths	Canada	Both	All Ages	Aortic aneurysm	Number	2019	2030.993524	2273.397883	1763.507034	-0.36%
DALYs	Central African Republic	Both	Age-standardized	Aortic aneurysm	Rate	1990	85.2237469	132.5186151	46.44466284	
DALYs	Central African Republic	Both	Age-standardized	Aortic aneurysm	Rate	2019	60.80773946	92.22470258	36.41855113	-28.65%
DALYs	Central African Republic	Both	All Ages	Aortic aneurysm	Number	1990	1033.265306	1678.219427	558.7834425	
DALYs	Central African Republic	Both	All Ages	Aortic aneurysm	Number	2019	1408.524193	2227.19733	809.1913067	36.32%
Deaths	Central African Republic	Both	Age-standardized	Aortic aneurysm	Rate	1990	3.931849627	5.8427661	2.177661279	
Deaths	Central African Republic	Both	Age-standardized	Aortic aneurysm	Rate	2019	2.785795762	3.989237915	1.7184769	-29.15%
Deaths	Central African Republic	Both	All Ages	Aortic aneurysm	Number	1990	38.33421933	59.83233478	20.8199399	
Deaths	Central African Republic	Both	All Ages	Aortic aneurysm	Number	2019	49.93580736	76.33991652	29.66669835	30.26%
DALYs	Chad	Both	Age-standardized	Aortic aneurysm	Rate	1990	34.62072596	48.66074443	17.81765368	
DALYs	Chad	Both	Age-standardized	Aortic aneurysm	Rate	2019	29.7872386	43.43359999	18.7609005	-13.96%
DALYs	Chad	Both	All Ages	Aortic aneurysm	Number	1990	995.397381	1405.24238	513.2461815	
DALYs	Chad	Both	All Ages	Aortic aneurysm	Number	2019	1740.225861	2592.536947	1078.399895	74.83%
Deaths	Chad	Both	Age-standardized	Aortic aneurysm	Rate	1990	1.713179134	2.393221154	0.88899294	
Deaths	Chad	Both	Age-standardized	Aortic aneurysm	Rate	2019	1.471119441	2.096182269	0.942982118	-14.13%
Deaths	Chad	Both	All Ages	Aortic aneurysm	Number	1990	44.11208403	61.70584502	22.90873818	
Deaths	Chad	Both	All Ages	Aortic aneurysm	Number	2019	71.19724051	103.529092	44.98560631	61.40%
DALYs	Chile	Both	Age-standardized	Aortic aneurysm	Rate	1990	48.66024562	52.44273797	44.55266063	
DALYs	Chile	Both	Age-standardized	Aortic aneurysm	Rate	2019	53.72937044	58.67618259	48.84606867	10.42%
DALYs	Chile	Both	All Ages	Aortic aneurysm	Number	1990	5003.429578	5389.807971	4591.314122	
DALYs	Chile	Both	All Ages	Aortic aneurysm	Number	2019	12648.34554	13846.16545	11484.90504	152.79%
Deaths	Chile	Both	Age-standardized	Aortic aneurysm	Rate	1990	2.323815384	2.521688761	2.130660961	
Deaths	Chile	Both	Age-standardized	Aortic aneurysm	Rate	2019	2.667999107	2.938596469	2.369184287	14.81%
Deaths	Chile	Both	All Ages	Aortic aneurysm	Number	1990	217.3095106	235.7410792	199.8749924	
Deaths	Chile	Both	All Ages	Aortic aneurysm	Number	2019	636.5693975	701.7773317	564.8631093	192.93%
DALYs	China	Both	Age-standardized	Aortic aneurysm	Rate	1990	21.09178193	27.89145132	16.77441952	
DALYs	China	Both	Age-standardized	Aortic aneurysm	Rate	2019	19.47960674	23.07156018	16.35914997	-7.64%
DALYs	China	Both	All Ages	Aortic aneurysm	Number	1990	186884.8132	250034.4425	146111.3967	
DALYs	China	Both	All Ages	Aortic aneurysm	Number	2019	378578.2355	450478.7506	315979.665	102.57%
Deaths	China	Both	Age-standardized	Aortic aneurysm	Rate	1990	0.998679259	1.297000817	0.811051361	
Deaths	China	Both	Age-standardized	Aortic aneurysm	Rate	2019	0.930869413	1.084619028	0.787436232	-6.79%

Deaths	China	Both	All Ages	Aortic aneurysm	Number	1990	7215.475773	9514.581863	5766.980199	
Deaths	China	Both	All Ages	Aortic aneurysm	Number	2019	17038.44293	19979.52252	14391.68145	136.14%
DALYs	Colombia	Both	Age-standardized	Aortic aneurysm	Rate	1990	72.0285717	76.26181238	68.04537806	
DALYs	Colombia	Both	Age-standardized	Aortic aneurysm	Rate	2019	57.61602677	74.94260092	43.61334414	-20.01%
DALYs	Colombia	Both	All Ages	Aortic aneurysm	Number	1990	13514.60121	14297.91542	12799.43242	
DALYs	Colombia	Both	All Ages	Aortic aneurysm	Number	2019	30176.62173	39223.89939	22814.90349	123.29%
Deaths	Colombia	Both	Age-standardized	Aortic aneurysm	Rate	1990	3.315672274	3.533768052	3.095858777	
Deaths	Colombia	Both	Age-standardized	Aortic aneurysm	Rate	2019	2.849291425	3.655700357	2.181362873	-14.07%
Deaths	Colombia	Both	All Ages	Aortic aneurysm	Number	1990	544.0295185	577.418216	511.5890897	
Deaths	Colombia	Both	All Ages	Aortic aneurysm	Number	2019	1515.453018	1939.164532	1158.545286	178.56%
DALYs	Comoros	Both	Age-standardized	Aortic aneurysm	Rate	1990	66.9237699	96.9459114	36.27324825	
DALYs	Comoros	Both	Age-standardized	Aortic aneurysm	Rate	2019	43.91131156	57.01357524	31.68206381	-34.39%
DALYs	Comoros	Both	All Ages	Aortic aneurysm	Number	1990	152.6739626	225.4383289	76.30187708	
DALYs	Comoros	Both	All Ages	Aortic aneurysm	Number	2019	217.6749598	285.5657629	154.9719947	42.58%
Deaths	Comoros	Both	Age-standardized	Aortic aneurysm	Rate	1990	3.343731405	4.604895482	2.075007522	
Deaths	Comoros	Both	Age-standardized	Aortic aneurysm	Rate	2019	2.248495427	2.908034841	1.644669282	-32.75%
Deaths	Comoros	Both	All Ages	Aortic aneurysm	Number	1990	6.649352882	9.339259996	3.895237177	
Deaths	Comoros	Both	All Ages	Aortic aneurysm	Number	2019	9.870671926	12.78855025	7.204176448	48.45%
DALYs	Congo	Both	Age-standardized	Aortic aneurysm	Rate	1990	98.49955591	128.1821437	72.82349486	
DALYs	Congo	Both	Age-standardized	Aortic aneurysm	Rate	2019	66.8777288	84.84030565	51.07468413	-32.10%
DALYs	Congo	Both	All Ages	Aortic aneurysm	Number	1990	1082.972992	1445.149281	782.2564703	
DALYs	Congo	Both	All Ages	Aortic aneurysm	Number	2019	1789.922515	2361.327725	1321.198785	65.28%
Deaths	Congo	Both	Age-standardized	Aortic aneurysm	Rate	1990	4.708545199	5.914975087	3.561207957	
Deaths	Congo	Both	Age-standardized	Aortic aneurysm	Rate	2019	3.342265045	4.141279174	2.651518905	-29.02%
Deaths	Congo	Both	All Ages	Aortic aneurysm	Number	1990	42.4733967	54.73296298	31.34818874	
Deaths	Congo	Both	All Ages	Aortic aneurysm	Number	2019	70.94503002	89.61905626	54.27708004	67.03%
DALYs	Cook Islands	Both	Age-standardized	Aortic aneurysm	Rate	1990	90.85810983	109.3565907	70.69792362	
DALYs	Cook Islands	Both	Age-standardized	Aortic aneurysm	Rate	2019	65.31862895	84.0911744	51.43586066	-28.11%
DALYs	Cook Islands	Both	All Ages	Aortic aneurysm	Number	1990	11.79849757	14.42002287	9.109055912	
DALYs	Cook Islands	Both	All Ages	Aortic aneurysm	Number	2019	15.46024389	19.60968199	12.28746419	31.04%
Deaths	Cook Islands	Both	Age-standardized	Aortic aneurysm	Rate	1990	4.363633081	5.157466303	3.48840352	
Deaths	Cook Islands	Both	Age-standardized	Aortic aneurysm	Rate	2019	3.224325833	3.97792224	2.597297969	-26.11%
Deaths	Cook Islands	Both	All Ages	Aortic aneurysm	Number	1990	0.491709796	0.582519719	0.386423062	
Deaths	Cook Islands	Both	All Ages	Aortic aneurysm	Number	2019	0.763336281	0.945154305	0.616334274	55.24%
DALYs	Costa Rica	Both	Age-standardized	Aortic aneurysm	Rate	1990	39.03675669	43.52544602	34.91778983	
DALYs	Costa Rica	Both	Age-standardized	Aortic aneurysm	Rate	2019	54.58452078	70.8801478	41.10931101	39.83%
DALYs	Costa Rica	Both	All Ages	Aortic aneurysm	Number	1990	729.6905476	808.4373382	659.283471	
DALYs	Costa Rica	Both	All Ages	Aortic aneurysm	Number	2019	2786.031871	3624.315005	2098.907852	281.81%
Deaths	Costa Rica	Both	Age-standardized	Aortic aneurysm	Rate	1990	1.926022768	2.179310383	1.68616242	
Deaths	Costa Rica	Both	Age-standardized	Aortic aneurysm	Rate	2019	2.683136778	3.46845365	2.046318081	39.31%
Deaths	Costa Rica	Both	All Ages	Aortic aneurysm	Number	1990	32.52230948	36.65686697	28.58927476	
Deaths	Costa Rica	Both	All Ages	Aortic aneurysm	Number	2019	135.5397304	174.421202	103.41144	316.76%
DALYs	Croatia	Both	Age-standardized	Aortic aneurysm	Rate	1990	47.22102289	51.43780178	43.20544916	
DALYs	Croatia	Both	Age-standardized	Aortic aneurysm	Rate	2019	79.64332769	100.6239344	62.01778321	68.66%
DALYs	Croatia	Both	All Ages	Aortic aneurysm	Number	1990	2990.852084	3263.173165	2728.39332	
DALYs	Croatia	Both	All Ages	Aortic aneurysm	Number	2019	6471.889853	8121.895827	5042.209896	116.39%
Deaths	Croatia	Both	Age-standardized	Aortic aneurysm	Rate	1990	2.01312335	2.196722018	1.846037399	
Deaths	Croatia	Both	Age-standardized	Aortic aneurysm	Rate	2019	3.815974274	4.713274995	3.002720579	89.55%
Deaths	Croatia	Both	All Ages	Aortic aneurysm	Number	1990	124.2536691	135.7385815	113.3209565	
Deaths	Croatia	Both	All Ages	Aortic aneurysm	Number	2019	344.2001744	423.7874072	272.1606714	177.01%
DALYs	Cuba	Both	Age-standardized	Aortic aneurysm	Rate	1990	79.98364328	90.49852057	68.78777572	
DALYs	Cuba	Both	Age-standardized	Aortic aneurysm	Rate	2019	71.33764744	88.15671109	57.0294403	-10.81%
DALYs	Cuba	Both	All Ages	Aortic aneurysm	Number	1990	8325.272321	9447.823811	7152.654729	
DALYs	Cuba	Both	All Ages	Aortic aneurysm	Number	2019	13535.37428	16727.74704	10817.72589	62.58%
Deaths	Cuba	Both	Age-standardized	Aortic aneurysm	Rate	1990	4.438243579	5.087028444	3.776788895	
Deaths	Cuba	Both	Age-standardized	Aortic aneurysm	Rate	2019	4.019064927	4.952230059	3.243292615	-9.44%
Deaths	Cuba	Both	All Ages	Aortic aneurysm	Number	1990	454.1196234	522.4271246	385.3801874	
Deaths	Cuba	Both	All Ages	Aortic aneurysm	Number	2019	796.1392412	980.1411236	642.3097656	75.31%
DALYs	Cyprus	Both	Age-standardized	Aortic aneurysm	Rate	1990	131.8605334	161.9374531	106.0879962	
DALYs	Cyprus	Both	Age-standardized	Aortic aneurysm	Rate	2019	78.82613529	90.74718641	67.78468637	-40.22%
DALYs	Cyprus	Both	All Ages	Aortic aneurysm	Number	1990	1074.624123	1312.788882	864.9946148	
DALYs	Cyprus	Both	All Ages	Aortic aneurysm	Number	2019	1510.195674	1745.366401	1292.007022	40.53%
Deaths	Cyprus	Both	Age-standardized	Aortic aneurysm	Rate	1990	6.788383598	8.460291635	5.456501404	
Deaths	Cyprus	Both	Age-standardized	Aortic aneurysm	Rate	2019	4.066264963	4.729232401	3.495349002	-40.10%
Deaths	Cyprus	Both	All Ages	Aortic aneurysm	Number	1990	51.00938671	63.23989295	41.0406384	
Deaths	Cyprus	Both	All Ages	Aortic aneurysm	Number	2019	77.69206513	90.45488496	66.33133956	52.31%
DALYs	Czechia	Both	Age-standardized	Aortic aneurysm	Rate	1990	50.59384597	54.12244226	46.91922195	
DALYs	Czechia	Both	Age-standardized	Aortic aneurysm	Rate	2019	59.04774244	71.8757592	48.14095349	16.71%
DALYs	Czechia	Both	All Ages	Aortic aneurysm	Number	1990	6744.056668	7216.219794	6243.39705	
DALYs	Czechia	Both	All Ages	Aortic aneurysm	Number	2019	11578.71975	14124.34878	9437.95881	71.69%
Deaths	Czechia	Both	Age-standardized	Aortic aneurysm	Rate	1990	2.176208722	2.331509967	2.027206844	
Deaths	Czechia	Both	Age-standardized	Aortic aneurysm	Rate	2019	2.856068665	3.452031252	2.338366708	31.24%
Deaths	Czechia	Both	All Ages	Aortic aneurysm	Number	1990	296.686145	317.7048943	275.2772022	
Deaths	Czechia	Both	All Ages	Aortic aneurysm	Number	2019	613.2010302	740.9628357	501.4538818	106.68%
DALYs	Côte d’Ivoire	Both	Age-standardized	Aortic aneurysm	Rate	1990	43.01996288	58.77399103	28.30507078	
DALYs	Côte d’Ivoire	Both	Age-standardized	Aortic aneurysm	Rate	2019	32.70307821	43.95780041	23.46839787	-23.98%
DALYs	Côte d’Ivoire	Both	All Ages	Aortic aneurysm	Number	1990	1820.117354	2564.749068	1162.087211	
DALYs	Côte d’Ivoire	Both	All Ages	Aortic aneurysm	Number	2019	3674.833975	5023.451549	2520.202714	101.90%
Deaths	Côte d’Ivoire	Both	Age-standardized	Aortic aneurysm	Rate	1990	2.13845203	2.882095366	1.433244897	
Deaths	Côte d’Ivoire	Both	Age-standardized	Aortic aneurysm	Rate	2019	1.641108302	2.141190491	1.218496807	-23.26%
Deaths	Côte d’Ivoire	Both	All Ages	Aortic aneurysm	Number	1990	67.57075663	92.63698139	44.16245707	
Deaths	Côte d’Ivoire	Both	All Ages	Aortic aneurysm	Number	2019	142.301669	190.4438525	102.4746211	110.60%
DALYs	Democratic People's Republic of Korea	Both	Age-standardized	Aortic aneurysm	Rate	1990	28.05123288	39.91307741	19.09637017	
DALYs	Democratic People's Republic of Korea	Both	Age-standardized	Aortic aneurysm	Rate	2019	29.58980522	38.31410415	23.49540102	5.48%
DALYs	Democratic People's Republic of Korea	Both	All Ages	Aortic aneurysm	Number	1990	4781.633766	7015.572768	3147.946082	
DALYs	Democratic People's Republic of Korea	Both	All Ages	Aortic aneurysm	Number	2019	9514.992213	12365.32546	7501.379365	98.99%
Deaths	Democratic People's Republic of Korea	Both	Age-standardized	Aortic aneurysm	Rate	1990	1.224725085	1.645337546	0.877915435	
Deaths	Democratic People's Republic of Korea	Both	Age-standardized	Aortic aneurysm	Rate	2019	1.229243191	1.480680148	1.033400045	0.37%
Deaths	Democratic People's Republic of Korea	Both	All Ages	Aortic aneurysm	Number	1990	173.0959445	239.0325761	119.5580036	
Deaths	Democratic People's Republic of Korea	Both	All Ages	Aortic aneurysm	Number	2019	377.8768836	461.9759442	314.1567564	118.30%
DALYs	Democratic Republic of the Congo	Both	Age-standardized	Aortic aneurysm	Rate	1990	69.58341569	96.44240672	44.38939347	
DALYs	Democratic Republic of the Congo	Both	Age-standardized	Aortic aneurysm	Rate	2019	52.27194155	70.28961273	37.00943034	-24.88%
DALYs	Democratic Republic of the Congo	Both	All Ages	Aortic aneurysm	Number	1990	11093.85172	15756.36015	7153.218396	
DALYs	Democratic Republic of the Congo	Both	All Ages	Aortic aneurysm	Number	2019	19294.89892	26506.6384	13587.35489	73.92%
Deaths	Democratic Republic of the Congo	Both	Age-standardized	Aortic aneurysm	Rate	1990	3.448664414	4.691342751	2.186595451	
Deaths	Democratic Republic of the Congo	Both	Age-standardized	Aortic aneurysm	Rate	2019	2.558000642	3.385372998	1.805942285	-25.83%
Deaths	Democratic Republic of the Congo	Both	All Ages	Aortic aneurysm	Number	1990	434.9942042	603.6013352	277.5316081	
Deaths	Democratic Republic of the Congo	Both	All Ages	Aortic aneurysm	Number	2019	758.8311634	1015.304341	538.6634873	74.45%
DALYs	Denmark	Both	Age-standardized	Aortic aneurysm	Rate	1990	124.494988	136.1348559	112.3620674	
DALYs	Denmark	Both	Age-standardized	Aortic aneurysm	Rate	2019	88.1340846	97.83620834	79.20374166	-29.21%
DALYs	Denmark	Both	All Ages	Aortic aneurysm	Number	1990	10125.55917	11156.90021	9093.280385	

DALYs	Denmark	Both	All Ages	Aortic aneurysm	Number	2019	10380.87021	11587.60928	9267.089627	2.52%
Deaths	Denmark	Both	Age-standardized	Aortic aneurysm	Rate	1990	6.400369507	7.048943773	5.745505761	
Deaths	Denmark	Both	Age-standardized	Aortic aneurysm	Rate	2019	5.322914292	5.955829409	4.720657526	-16.83%
Deaths	Denmark	Both	All Ages	Aortic aneurysm	Number	1990	552.7731252	612.5331358	493.0171457	
Deaths	Denmark	Both	All Ages	Aortic aneurysm	Number	2019	671.2519512	752.4031244	591.8962897	21.43%
DALYs	Djibouti	Both	Age-standardized	Aortic aneurysm	Rate	1990	63.67818505	91.15925211	37.75950054	
DALYs	Djibouti	Both	Age-standardized	Aortic aneurysm	Rate	2019	46.51435269	65.60810133	31.85867519	-26.95%
DALYs	Djibouti	Both	All Ages	Aortic aneurysm	Number	1990	100.0949518	148.0345537	58.67960663	
DALYs	Djibouti	Both	All Ages	Aortic aneurysm	Number	2019	292.5528501	433.1733316	189.7135132	192.28%
Deaths	Djibouti	Both	Age-standardized	Aortic aneurysm	Rate	1990	3.130725564	4.367532331	1.85758141	
Deaths	Djibouti	Both	Age-standardized	Aortic aneurysm	Rate	2019	2.345963784	3.239463789	1.653690893	-25.07%
Deaths	Djibouti	Both	All Ages	Aortic aneurysm	Number	1990	3.465601674	5.030989075	2.036686761	
Deaths	Djibouti	Both	All Ages	Aortic aneurysm	Number	2019	10.97650909	15.57277442	7.48817234	216.73%
DALYs	Dominica	Both	Age-standardized	Aortic aneurysm	Rate	1990	88.46910975	103.4332161	73.41400867	
DALYs	Dominica	Both	Age-standardized	Aortic aneurysm	Rate	2019	85.56197937	104.0923679	70.25314706	-3.29%
DALYs	Dominica	Both	All Ages	Aortic aneurysm	Number	1990	65.40004864	76.20172808	54.26048495	
DALYs	Dominica	Both	All Ages	Aortic aneurysm	Number	2019	76.99291717	94.0478532	63.17877128	17.73%
Deaths	Dominica	Both	Age-standardized	Aortic aneurysm	Rate	1990	4.978296601	5.731937573	4.164052762	
Deaths	Dominica	Both	Age-standardized	Aortic aneurysm	Rate	2019	4.914872103	5.924029322	4.068046753	-1.27%
Deaths	Dominica	Both	All Ages	Aortic aneurysm	Number	1990	3.798572487	4.392386837	3.173104939	
Deaths	Dominica	Both	All Ages	Aortic aneurysm	Number	2019	4.501781673	5.448710407	3.727217617	18.51%
DALYs	Dominican Republic	Both	Age-standardized	Aortic aneurysm	Rate	1990	29.03974064	33.2303967	25.1122831	
DALYs	Dominican Republic	Both	Age-standardized	Aortic aneurysm	Rate	2019	42.2101867	55.01565781	31.77274393	45.35%
DALYs	Dominican Republic	Both	All Ages	Aortic aneurysm	Number	1990	1148.199589	1323.115629	993.9398767	
DALYs	Dominican Republic	Both	All Ages	Aortic aneurysm	Number	2019	3988.161689	5212.0528	2986.131542	247.34%
Deaths	Dominican Republic	Both	Age-standardized	Aortic aneurysm	Rate	1990	1.437604526	1.642798901	1.24235121	
Deaths	Dominican Republic	Both	Age-standardized	Aortic aneurysm	Rate	2019	2.072948585	2.64007044	1.611817507	44.19%
Deaths	Dominican Republic	Both	All Ages	Aortic aneurysm	Number	1990	48.05653776	54.72272587	41.60415801	
Deaths	Dominican Republic	Both	All Ages	Aortic aneurysm	Number	2019	183.0340163	234.2266922	141.4715318	280.87%
DALYs	Ecuador	Both	Age-standardized	Aortic aneurysm	Rate	1990	35.46859608	39.57027069	31.66323689	
DALYs	Ecuador	Both	Age-standardized	Aortic aneurysm	Rate	2019	37.29701717	48.21880262	29.36370054	5.16%
DALYs	Ecuador	Both	All Ages	Aortic aneurysm	Number	1990	2020.446246	2240.281848	1809.932204	
DALYs	Ecuador	Both	All Ages	Aortic aneurysm	Number	2019	5655.779523	7321.134954	4446.935655	179.93%
Deaths	Ecuador	Both	Age-standardized	Aortic aneurysm	Rate	1990	1.785181774	2.004660406	1.582945053	
Deaths	Ecuador	Both	Age-standardized	Aortic aneurysm	Rate	2019	2.001723482	2.539235115	1.593135793	12.13%
Deaths	Ecuador	Both	All Ages	Aortic aneurysm	Number	1990	87.00152168	97.5196771	77.31352648	
Deaths	Ecuador	Both	All Ages	Aortic aneurysm	Number	2019	275.9095916	351.1389439	218.2666733	217.13%
DALYs	Egypt	Both	Age-standardized	Aortic aneurysm	Rate	1990	24.98997372	35.90177856	16.97724903	
DALYs	Egypt	Both	Age-standardized	Aortic aneurysm	Rate	2019	29.4736525	42.5472655	20.90321963	17.94%
DALYs	Egypt	Both	All Ages	Aortic aneurysm	Number	1990	8005.408765	11470.98979	5402.49417	
DALYs	Egypt	Both	All Ages	Aortic aneurysm	Number	2019	19751.54679	28485.60487	13927.41901	146.73%
Deaths	Egypt	Both	Age-standardized	Aortic aneurysm	Rate	1990	1.185391505	1.671339785	0.809718866	
Deaths	Egypt	Both	Age-standardized	Aortic aneurysm	Rate	2019	1.440090874	2.08270103	1.034320064	21.49%
Deaths	Egypt	Both	All Ages	Aortic aneurysm	Number	1990	297.4598669	423.6898662	202.6542041	
Deaths	Egypt	Both	All Ages	Aortic aneurysm	Number	2019	756.7502832	1097.932918	535.9719289	154.40%
DALYs	El Salvador	Both	Age-standardized	Aortic aneurysm	Rate	1990	18.24896323	20.88131184	16.05531334	
DALYs	El Salvador	Both	Age-standardized	Aortic aneurysm	Rate	2019	18.83620282	24.40563302	14.15034243	3.22%
DALYs	El Salvador	Both	All Ages	Aortic aneurysm	Number	1990	578.6544851	659.8442238	510.4863535	
DALYs	El Salvador	Both	All Ages	Aortic aneurysm	Number	2019	1129.008696	1462.154391	847.4292094	95.11%
Deaths	El Salvador	Both	Age-standardized	Aortic aneurysm	Rate	1990	0.863380009	0.993901615	0.74975182	
Deaths	El Salvador	Both	Age-standardized	Aortic aneurysm	Rate	2019	0.919600217	1.175053829	0.698940124	6.51%
Deaths	El Salvador	Both	All Ages	Aortic aneurysm	Number	1990	24.86505436	28.55953111	21.69020437	
Deaths	El Salvador	Both	All Ages	Aortic aneurysm	Number	2019	56.80527965	72.52503882	43.15314605	128.45%
DALYs	Equatorial Guinea	Both	Age-standardized	Aortic aneurysm	Rate	1990	74.03327618	113.9825794	38.7673275	
DALYs	Equatorial Guinea	Both	Age-standardized	Aortic aneurysm	Rate	2019	57.27932649	84.2023549	39.03126517	-22.63%
DALYs	Equatorial Guinea	Both	All Ages	Aortic aneurysm	Number	1990	150.4003337	239.9919803	76.47688605	
DALYs	Equatorial Guinea	Both	All Ages	Aortic aneurysm	Number	2019	287.2245472	434.1012154	186.2655248	90.97%
Deaths	Equatorial Guinea	Both	Age-standardized	Aortic aneurysm	Rate	1990	3.445428578	5.094384402	1.85812262	
Deaths	Equatorial Guinea	Both	Age-standardized	Aortic aneurysm	Rate	2019	3.020519056	4.31335352	2.169292346	-12.33%
Deaths	Equatorial Guinea	Both	All Ages	Aortic aneurysm	Number	1990	5.882184565	8.986621909	3.072654129	
Deaths	Equatorial Guinea	Both	All Ages	Aortic aneurysm	Number	2019	11.94022164	17.4121483	8.319373512	102.99%
DALYs	Eritrea	Both	Age-standardized	Aortic aneurysm	Rate	1990	69.79660166	111.4720246	40.23593688	
DALYs	Eritrea	Both	Age-standardized	Aortic aneurysm	Rate	2019	52.54002086	76.59965698	34.67232174	-24.72%
DALYs	Eritrea	Both	All Ages	Aortic aneurysm	Number	1990	776.2821958	1340.907117	418.7856064	
DALYs	Eritrea	Both	All Ages	Aortic aneurysm	Number	2019	1561.43271	2379.615227	995.5406185	101.14%
Deaths	Eritrea	Both	Age-standardized	Aortic aneurysm	Rate	1990	3.166638745	4.88783764	1.914058803	
Deaths	Eritrea	Both	Age-standardized	Aortic aneurysm	Rate	2019	2.528366109	3.557384566	1.700683436	-20.16%
Deaths	Eritrea	Both	All Ages	Aortic aneurysm	Number	1990	25.8977346	42.21419853	14.63215271	
Deaths	Eritrea	Both	All Ages	Aortic aneurysm	Number	2019	54.7723552	80.06530449	35.90493262	111.49%
DALYs	Estonia	Both	Age-standardized	Aortic aneurysm	Rate	1990	58.29581989	66.69881204	51.33627505	
DALYs	Estonia	Both	Age-standardized	Aortic aneurysm	Rate	2019	62.99773295	82.89291243	47.68512598	8.07%
DALYs	Estonia	Both	All Ages	Aortic aneurysm	Number	1990	1176.721372	1350.214066	1031.882121	
DALYs	Estonia	Both	All Ages	Aortic aneurysm	Number	2019	1505.386879	1988.320857	1141.289458	27.93%
Deaths	Estonia	Both	Age-standardized	Aortic aneurysm	Rate	1990	2.493376178	2.849880219	2.202691949	
Deaths	Estonia	Both	Age-standardized	Aortic aneurysm	Rate	2019	2.8940726	3.815959719	2.208563087	16.07%
Deaths	Estonia	Both	All Ages	Aortic aneurysm	Number	1990	50.81874727	58.07073352	44.70941979	
Deaths	Estonia	Both	All Ages	Aortic aneurysm	Number	2019	78.83801472	103.5853845	60.2140112	55.14%
DALYs	Eswatini	Both	Age-standardized	Aortic aneurysm	Rate	1990	53.33066675	68.90719642	40.0299767	
DALYs	Eswatini	Both	Age-standardized	Aortic aneurysm	Rate	2019	45.35776787	61.70226314	32.21083003	-14.95%
DALYs	Eswatini	Both	All Ages	Aortic aneurysm	Number	1990	163.3455499	212.7077653	121.2630881	
DALYs	Eswatini	Both	All Ages	Aortic aneurysm	Number	2019	282.3154561	403.2968899	190.7720994	72.83%
Deaths	Eswatini	Both	Age-standardized	Aortic aneurysm	Rate	1990	2.701179374	3.42253864	2.075070557	
Deaths	Eswatini	Both	Age-standardized	Aortic aneurysm	Rate	2019	2.231872425	2.973486472	1.692516768	-17.37%
Deaths	Eswatini	Both	All Ages	Aortic aneurysm	Number	1990	6.565138001	8.414971867	4.953902774	
Deaths	Eswatini	Both	All Ages	Aortic aneurysm	Number	2019	10.78268958	14.53316093	7.816928261	64.24%
DALYs	Ethiopia	Both	Age-standardized	Aortic aneurysm	Rate	1990	57.94589395	100.3767066	27.28016529	
DALYs	Ethiopia	Both	Age-standardized	Aortic aneurysm	Rate	2019	32.07423664	42.37843072	20.65594444	-44.65%
DALYs	Ethiopia	Both	All Ages	Aortic aneurysm	Number	1990	12116.69005	21908.84961	5650.995133	
DALYs	Ethiopia	Both	All Ages	Aortic aneurysm	Number	2019	13919.86618	18634.17989	9007.366131	14.88%
Deaths	Ethiopia	Both	Age-standardized	Aortic aneurysm	Rate	1990	2.751176379	4.472041448	1.31965048	
Deaths	Ethiopia	Both	Age-standardized	Aortic aneurysm	Rate	2019	1.6951066	2.224303662	1.078708987	-38.39%
Deaths	Ethiopia	Both	All Ages	Aortic aneurysm	Number	1990	450.415297	788.7655721	213.1039068	
Deaths	Ethiopia	Both	All Ages	Aortic aneurysm	Number	2019	597.3327555	786.0051666	386.6046102	32.62%
DALYs	Fiji	Both	Age-standardized	Aortic aneurysm	Rate	1990	114.3788649	141.8584608	90.65068437	
DALYs	Fiji	Both	Age-standardized	Aortic aneurysm	Rate	2019	105.9074603	131.9573638	82.79611944	-7.41%
DALYs	Fiji	Both	All Ages	Aortic aneurysm	Number	1990	441.1500115	554.1921594	345.3862448	
DALYs	Fiji	Both	All Ages	Aortic aneurysm	Number	2019	781.9129992	993.2770715	594.5125152	77.24%
Deaths	Fiji	Both	Age-standardized	Aortic aneurysm	Rate	1990	5.648825606	6.932437299	4.532521307	
Deaths	Fiji	Both	Age-standardized	Aortic aneurysm	Rate	2019	5.397848961	6.555921285	4.357203179	-4.44%
Deaths	Fiji	Both	All Ages	Aortic aneurysm	Number	1990	16.16897772	20.05892091	12.7926944	
Deaths	Fiji	Both	All Ages	Aortic aneurysm	Number	2019	31.44479847	39.15154172	24.45827256	94.48%

DALYs	Finland	Both	Age-standardized	Aortic aneurysm	Rate	1990	137.5157185	156.0352425	117.5549705	
DALYs	Finland	Both	Age-standardized	Aortic aneurysm	Rate	2019	86.10243208	95.18825063	77.58201629	-37.39%
DALYs	Finland	Both	All Ages	Aortic aneurysm	Number	1990	9787.801824	11141.67466	8363.394468	
DALYs	Finland	Both	All Ages	Aortic aneurysm	Number	2019	10319.37444	11510.0688	9220.595453	5.43%
Deaths	Finland	Both	Age-standardized	Aortic aneurysm	Rate	1990	6.95081764	7.901444076	6.006584325	
Deaths	Finland	Both	Age-standardized	Aortic aneurysm	Rate	2019	4.837897423	5.408457096	4.298875395	-30.40%
Deaths	Finland	Both	All Ages	Aortic aneurysm	Number	1990	509.0652425	580.5648458	439.4061287	
Deaths	Finland	Both	All Ages	Aortic aneurysm	Number	2019	654.4895152	735.8543517	575.212023	28.57%
DALYs	France	Both	Age-standardized	Aortic aneurysm	Rate	1990	62.99269244	65.83877694	59.76395575	
DALYs	France	Both	Age-standardized	Aortic aneurysm	Rate	2019	38.73942653	42.69627095	34.87933118	-38.50%
DALYs	France	Both	All Ages	Aortic aneurysm	Number	1990	51369.13784	53861.91924	48576.90708	
DALYs	France	Both	All Ages	Aortic aneurysm	Number	2019	50759.73415	55988.83456	45380.26285	-1.19%
Deaths	France	Both	Age-standardized	Aortic aneurysm	Rate	1990	3.373062494	3.562383174	3.149737376	
Deaths	France	Both	Age-standardized	Aortic aneurysm	Rate	2019	2.144219506	2.377527543	1.901024797	-36.43%
Deaths	France	Both	All Ages	Aortic aneurysm	Number	1990	2946.313729	3126.129189	2741.109774	
Deaths	France	Both	All Ages	Aortic aneurysm	Number	2019	3400.857926	3809.280536	2956.132008	15.43%
DALYs	Gabon	Both	Age-standardized	Aortic aneurysm	Rate	1990	94.65645702	130.0438845	66.55320804	
DALYs	Gabon	Both	Age-standardized	Aortic aneurysm	Rate	2019	75.42325886	96.60745829	57.51548051	-20.32%
DALYs	Gabon	Both	All Ages	Aortic aneurysm	Number	1990	537.9291392	745.5672909	372.740741	
DALYs	Gabon	Both	All Ages	Aortic aneurysm	Number	2019	804.2152796	1045.65132	597.6277385	49.50%
Deaths	Gabon	Both	Age-standardized	Aortic aneurysm	Rate	1990	4.561035964	6.084549341	3.222489984	
Deaths	Gabon	Both	Age-standardized	Aortic aneurysm	Rate	2019	3.71057064	4.621370732	2.926484213	-18.65%
Deaths	Gabon	Both	All Ages	Aortic aneurysm	Number	1990	22.89277929	30.82089059	16.17013989	
Deaths	Gabon	Both	All Ages	Aortic aneurysm	Number	2019	33.63421493	42.72928586	26.05106945	46.92%
DALYs	Gambia	Both	Age-standardized	Aortic aneurysm	Rate	1990	35.85373424	50.59880863	21.16102565	
DALYs	Gambia	Both	Age-standardized	Aortic aneurysm	Rate	2019	34.08089775	44.13595174	25.68660969	-4.94%
DALYs	Gambia	Both	All Ages	Aortic aneurysm	Number	1990	130.5354571	187.7774898	76.07862126	
DALYs	Gambia	Both	All Ages	Aortic aneurysm	Number	2019	344.5924465	454.9525057	255.1282539	163.98%
Deaths	Gambia	Both	Age-standardized	Aortic aneurysm	Rate	1990	1.825602264	2.487963205	1.09398896	
Deaths	Gambia	Both	Age-standardized	Aortic aneurysm	Rate	2019	1.719369346	2.170671496	1.330330919	-5.82%
Deaths	Gambia	Both	All Ages	Aortic aneurysm	Number	1990	5.368353879	7.539031497	3.15368096	
Deaths	Gambia	Both	All Ages	Aortic aneurysm	Number	2019	14.80716832	18.99211378	11.30640793	175.82%
DALYs	Georgia	Both	Age-standardized	Aortic aneurysm	Rate	1990	15.64509839	18.74139984	11.39216268	
DALYs	Georgia	Both	Age-standardized	Aortic aneurysm	Rate	2019	53.79183655	64.65086378	43.47897889	243.83%
DALYs	Georgia	Both	All Ages	Aortic aneurysm	Number	1990	947.4750895	1145.067708	688.286459	
DALYs	Georgia	Both	All Ages	Aortic aneurysm	Number	2019	2989.926844	3577.794328	2413.573323	215.57%
Deaths	Georgia	Both	Age-standardized	Aortic aneurysm	Rate	1990	0.75582324	0.907558281	0.555320539	
Deaths	Georgia	Both	Age-standardized	Aortic aneurysm	Rate	2019	2.302574659	2.736025119	1.864983985	204.64%
Deaths	Georgia	Both	All Ages	Aortic aneurysm	Number	1990	42.20920666	50.69085168	30.93473674	
Deaths	Georgia	Both	All Ages	Aortic aneurysm	Number	2019	139.3870282	165.6945823	112.4837714	230.23%
DALYs	Germany	Both	Age-standardized	Aortic aneurysm	Rate	1990	60.29510028	66.39485739	54.42840953	
DALYs	Germany	Both	Age-standardized	Aortic aneurysm	Rate	2019	43.77802017	47.41416584	40.21116986	-27.39%
DALYs	Germany	Both	All Ages	Aortic aneurysm	Number	1990	74585.69586	82485.82044	66919.8743	
DALYs	Germany	Both	All Ages	Aortic aneurysm	Number	2019	75708.55172	82687.52934	68844.0825	1.51%
Deaths	Germany	Both	Age-standardized	Aortic aneurysm	Rate	1990	3.077035233	3.394573412	2.752442896	
Deaths	Germany	Both	Age-standardized	Aortic aneurysm	Rate	2019	2.217573122	2.426687401	1.999073968	-27.93%
Deaths	Germany	Both	All Ages	Aortic aneurysm	Number	1990	4061.258936	4530.449826	3613.19958	
Deaths	Germany	Both	All Ages	Aortic aneurysm	Number	2019	4557.83087	5040.559536	4040.375936	12.23%
DALYs	Ghana	Both	Age-standardized	Aortic aneurysm	Rate	1990	44.87183478	55.18145592	36.25782172	
DALYs	Ghana	Both	Age-standardized	Aortic aneurysm	Rate	2019	28.84408937	36.91472473	22.14127328	-35.72%
DALYs	Ghana	Both	All Ages	Aortic aneurysm	Number	1990	2933.261914	3653.690109	2332.510138	
DALYs	Ghana	Both	All Ages	Aortic aneurysm	Number	2019	4901.280066	6383.268197	3602.317338	67.09%
Deaths	Ghana	Both	Age-standardized	Aortic aneurysm	Rate	1990	2.210005053	2.682856375	1.808405361	
Deaths	Ghana	Both	Age-standardized	Aortic aneurysm	Rate	2019	1.429061029	1.811788135	1.136197218	-35.34%
Deaths	Ghana	Both	All Ages	Aortic aneurysm	Number	1990	115.388132	141.8438656	93.28684492	
Deaths	Ghana	Both	All Ages	Aortic aneurysm	Number	2019	196.4912808	250.8887452	151.1749873	70.29%
DALYs	Greece	Both	Age-standardized	Aortic aneurysm	Rate	1990	72.77173432	76.68927465	69.06511863	
DALYs	Greece	Both	Age-standardized	Aortic aneurysm	Rate	2019	93.89884197	103.5481935	84.73656971	29.03%
DALYs	Greece	Both	All Ages	Aortic aneurysm	Number	1990	10585.88192	11168.14363	10044.06029	
DALYs	Greece	Both	All Ages	Aortic aneurysm	Number	2019	19123.6089	21053.12773	17107.16101	80.65%
Deaths	Greece	Both	Age-standardized	Aortic aneurysm	Rate	1990	3.443652025	3.650282866	3.247668897	
Deaths	Greece	Both	Age-standardized	Aortic aneurysm	Rate	2019	4.399446264	4.848000504	3.907265614	27.76%
Deaths	Greece	Both	All Ages	Aortic aneurysm	Number	1990	515.1774124	546.6933908	485.0894194	
Deaths	Greece	Both	All Ages	Aortic aneurysm	Number	2019	1104.63338	1223.01718	970.489493	114.42%
DALYs	Greenland	Both	Age-standardized	Aortic aneurysm	Rate	1990	65.23852953	76.16248257	56.58330435	
DALYs	Greenland	Both	Age-standardized	Aortic aneurysm	Rate	2019	39.99903326	48.17416034	32.34172848	-38.69%
DALYs	Greenland	Both	All Ages	Aortic aneurysm	Number	1990	21.26556932	24.96083771	18.15196286	
DALYs	Greenland	Both	All Ages	Aortic aneurysm	Number	2019	26.21673091	31.73895044	20.89500789	23.28%
Deaths	Greenland	Both	Age-standardized	Aortic aneurysm	Rate	1990	3.558839122	4.110107553	3.097522347	
Deaths	Greenland	Both	Age-standardized	Aortic aneurysm	Rate	2019	2.254340641	2.692305552	1.843703653	-36.66%
Deaths	Greenland	Both	All Ages	Aortic aneurysm	Number	1990	0.910762124	1.06024033	0.791234082	
Deaths	Greenland	Both	All Ages	Aortic aneurysm	Number	2019	1.296310624	1.547697365	1.051626952	42.33%
DALYs	Grenada	Both	Age-standardized	Aortic aneurysm	Rate	1990	87.72781132	106.3840222	73.70487706	
DALYs	Grenada	Both	Age-standardized	Aortic aneurysm	Rate	2019	89.72774157	101.6586511	78.52571127	2.28%
DALYs	Grenada	Both	All Ages	Aortic aneurysm	Number	1990	64.27762594	78.79958764	53.82027617	
DALYs	Grenada	Both	All Ages	Aortic aneurysm	Number	2019	99.33909609	112.7271383	86.36139544	54.55%
Deaths	Grenada	Both	Age-standardized	Aortic aneurysm	Rate	1990	4.580459269	5.750885642	3.818122259	
Deaths	Grenada	Both	Age-standardized	Aortic aneurysm	Rate	2019	4.649811824	5.257268467	4.046167384	1.51%
Deaths	Grenada	Both	All Ages	Aortic aneurysm	Number	1990	3.621173592	4.63862182	2.989286418	
Deaths	Grenada	Both	All Ages	Aortic aneurysm	Number	2019	4.765714814	5.406579641	4.140028812	31.61%
DALYs	Guam	Both	Age-standardized	Aortic aneurysm	Rate	1990	139.3141218	165.0748487	119.1357875	
DALYs	Guam	Both	Age-standardized	Aortic aneurysm	Rate	2019	53.21260954	63.99803835	43.78548124	-61.80%
DALYs	Guam	Both	All Ages	Aortic aneurysm	Number	1990	110.9469116	130.8952447	94.39121717	
DALYs	Guam	Both	All Ages	Aortic aneurysm	Number	2019	99.24521492	119.6408155	81.13587072	-10.55%
Deaths	Guam	Both	Age-standardized	Aortic aneurysm	Rate	1990	7.124698827	8.379328346	6.062299126	
Deaths	Guam	Both	Age-standardized	Aortic aneurysm	Rate	2019	2.337584745	2.782093447	1.939935945	-67.19%
Deaths	Guam	Both	All Ages	Aortic aneurysm	Number	1990	4.230082808	5.007290077	3.602670865	
Deaths	Guam	Both	All Ages	Aortic aneurysm	Number	2019	4.337418177	5.175904563	3.573468249	2.54%
DALYs	Guatemala	Both	Age-standardized	Aortic aneurysm	Rate	1990	21.23215544	25.45706833	17.70726488	
DALYs	Guatemala	Both	Age-standardized	Aortic aneurysm	Rate	2019	19.62423125	25.05899086	15.42713137	-7.57%
DALYs	Guatemala	Both	All Ages	Aortic aneurysm	Number	1990	797.2547217	968.6071098	660.7078617	
DALYs	Guatemala	Both	All Ages	Aortic aneurysm	Number	2019	2283.256289	2933.879823	1781.99128	186.39%
Deaths	Guatemala	Both	Age-standardized	Aortic aneurysm	Rate	1990	1.121687862	1.367531234	0.914927511	
Deaths	Guatemala	Both	Age-standardized	Aortic aneurysm	Rate	2019	1.044130737	1.297675243	0.842217026	-6.91%
Deaths	Guatemala	Both	All Ages	Aortic aneurysm	Number	1990	31.89503844	38.364908	26.71093188	
Deaths	Guatemala	Both	All Ages	Aortic aneurysm	Number	2019	106.9267248	134.4243247	84.88510612	235.25%
DALYs	Guinea	Both	Age-standardized	Aortic aneurysm	Rate	1990	39.09913084	53.60383328	23.69533249	
DALYs	Guinea	Both	Age-standardized	Aortic aneurysm	Rate	2019	34.61635414	48.8106918	24.02257543	-11.47%
DALYs	Guinea	Both	All Ages	Aortic aneurysm	Number	1990	1314.643731	1821.538301	792.3356531	
DALYs	Guinea	Both	All Ages	Aortic aneurysm	Number	2019	1983.603955	2804.693931	1368.193736	50.89%
Deaths	Guinea	Both	Age-standardized	Aortic aneurysm	Rate	1990	1.932572465	2.598984821	1.190027847	

Deaths	Guinea	Both	Age-standardized	Aortic aneurysm	Rate	2019	1.694830964	2.357680434	1.202987431	-12.30%
Deaths	Guinea	Both	All Ages	Aortic aneurysm	Number	1990	57.76688773	78.43078502	35.40326399	
Deaths	Guinea	Both	All Ages	Aortic aneurysm	Number	2019	84.40670403	118.7844739	59.36010745	46.12%
DALYs	Guinea-Bissau	Both	Age-standardized	Aortic aneurysm	Rate	1990	49.53210911	75.57462478	29.14658318	
DALYs	Guinea-Bissau	Both	Age-standardized	Aortic aneurysm	Rate	2019	39.29117895	54.8525735	27.60961278	-20.68%
DALYs	Guinea-Bissau	Both	All Ages	Aortic aneurysm	Number	1990	210.8161319	329.6375114	121.8985234	
DALYs	Guinea-Bissau	Both	All Ages	Aortic aneurysm	Number	2019	312.682867	445.9869159	214.5337595	48.32%
Deaths	Guinea-Bissau	Both	Age-standardized	Aortic aneurysm	Rate	1990	2.321182591	3.4646938	1.39191725	
Deaths	Guinea-Bissau	Both	Age-standardized	Aortic aneurysm	Rate	2019	1.838742044	2.50426591	1.342333557	-20.78%
Deaths	Guinea-Bissau	Both	All Ages	Aortic aneurysm	Number	1990	8.224664206	12.66508882	4.872655115	
Deaths	Guinea-Bissau	Both	All Ages	Aortic aneurysm	Number	2019	11.4770923	15.9780205	8.084067095	39.54%
DALYs	Guyana	Both	Age-standardized	Aortic aneurysm	Rate	1990	59.88438682	68.8005694	51.82210025	
DALYs	Guyana	Both	Age-standardized	Aortic aneurysm	Rate	2019	91.68445258	115.274122	70.41952115	53.10%
DALYs	Guyana	Both	All Ages	Aortic aneurysm	Number	1990	234.0997992	267.9662955	201.9472548	
DALYs	Guyana	Both	All Ages	Aortic aneurysm	Number	2019	577.1490409	733.0321851	440.5668509	146.54%
Deaths	Guyana	Both	Age-standardized	Aortic aneurysm	Rate	1990	2.969163193	3.384072489	2.588102288	
Deaths	Guyana	Both	Age-standardized	Aortic aneurysm	Rate	2019	4.517434996	5.57475797	3.541997416	52.15%
Deaths	Guyana	Both	All Ages	Aortic aneurysm	Number	1990	9.983915875	11.41857662	8.651717723	
Deaths	Guyana	Both	All Ages	Aortic aneurysm	Number	2019	24.72936185	30.89176426	19.20506665	147.69%
DALYs	Haiti	Both	Age-standardized	Aortic aneurysm	Rate	1990	71.68112236	104.2256146	46.32305243	
DALYs	Haiti	Both	Age-standardized	Aortic aneurysm	Rate	2019	62.5710699	94.80723447	37.6489576	-12.71%
DALYs	Haiti	Both	All Ages	Aortic aneurysm	Number	1990	2280.448317	3400.561076	1429.71588	
DALYs	Haiti	Both	All Ages	Aortic aneurysm	Number	2019	4288.477936	6530.414398	2523.25676	88.05%
Deaths	Haiti	Both	Age-standardized	Aortic aneurysm	Rate	1990	3.708895396	5.244311559	2.480223657	
Deaths	Haiti	Both	Age-standardized	Aortic aneurysm	Rate	2019	3.285733638	4.83754095	2.045942065	-11.41%
Deaths	Haiti	Both	All Ages	Aortic aneurysm	Number	1990	97.34620558	141.2794943	62.8585825	
Deaths	Haiti	Both	All Ages	Aortic aneurysm	Number	2019	187.3747203	283.0095014	114.4440457	92.48%
DALYs	Honduras	Both	Age-standardized	Aortic aneurysm	Rate	1990	19.01189305	27.73442369	12.9991083	
DALYs	Honduras	Both	Age-standardized	Aortic aneurysm	Rate	2019	26.10263834	33.68256428	19.78037222	37.30%
DALYs	Honduras	Both	All Ages	Aortic aneurysm	Number	1990	399.6409725	567.3197587	272.7351989	
DALYs	Honduras	Both	All Ages	Aortic aneurysm	Number	2019	1525.390913	1996.883428	1143.607268	281.69%
Deaths	Honduras	Both	Age-standardized	Aortic aneurysm	Rate	1990	0.980751279	1.533754152	0.669009627	
Deaths	Honduras	Both	Age-standardized	Aortic aneurysm	Rate	2019	1.470679613	1.900781586	1.141969558	49.95%
Deaths	Honduras	Both	All Ages	Aortic aneurysm	Number	1990	17.86494535	27.16282843	12.26928292	
Deaths	Honduras	Both	All Ages	Aortic aneurysm	Number	2019	76.19094267	97.99221952	58.81327494	326.48%
DALYs	Hungary	Both	Age-standardized	Aortic aneurysm	Rate	1990	60.81599897	64.54138126	57.39852235	
DALYs	Hungary	Both	Age-standardized	Aortic aneurysm	Rate	2019	60.27095327	73.84476782	49.30740671	-0.90%
DALYs	Hungary	Both	All Ages	Aortic aneurysm	Number	1990	8594.680358	9151.319195	8096.598242	
DALYs	Hungary	Both	All Ages	Aortic aneurysm	Number	2019	10773.20524	13110.22345	8796.902105	25.35%
Deaths	Hungary	Both	Age-standardized	Aortic aneurysm	Rate	1990	2.692605674	2.852204532	2.527543279	
Deaths	Hungary	Both	Age-standardized	Aortic aneurysm	Rate	2019	2.859689076	3.42468088	2.354699516	6.21%
Deaths	Hungary	Both	All Ages	Aortic aneurysm	Number	1990	383.3012955	407.5400467	359.3641184	
Deaths	Hungary	Both	All Ages	Aortic aneurysm	Number	2019	562.2588775	672.4963956	462.2269414	46.69%
DALYs	Iceland	Both	Age-standardized	Aortic aneurysm	Rate	1990	79.14700071	87.18215078	71.96999712	
DALYs	Iceland	Both	Age-standardized	Aortic aneurysm	Rate	2019	54.05945548	61.02045684	47.07420964	-31.70%
DALYs	Iceland	Both	All Ages	Aortic aneurysm	Number	1990	225.3752464	247.8664261	205.3884662	
DALYs	Iceland	Both	All Ages	Aortic aneurysm	Number	2019	291.8984948	330.07796	253.4736424	29.52%
Deaths	Iceland	Both	Age-standardized	Aortic aneurysm	Rate	1990	4.140108443	4.569657152	3.721326676	
Deaths	Iceland	Both	Age-standardized	Aortic aneurysm	Rate	2019	3.014518675	3.450435684	2.581454485	-27.19%
Deaths	Iceland	Both	All Ages	Aortic aneurysm	Number	1990	12.28765172	13.55952432	11.00828395	
Deaths	Iceland	Both	All Ages	Aortic aneurysm	Number	2019	18.06692643	20.76307768	15.41889873	47.03%
DALYs	India	Both	Age-standardized	Aortic aneurysm	Rate	1990	32.28759528	49.14402283	20.26139366	
DALYs	India	Both	Age-standardized	Aortic aneurysm	Rate	2019	32.87026317	40.92824737	24.75630305	1.80%
DALYs	India	Both	All Ages	Aortic aneurysm	Number	1990	143696.5523	222255.5082	88354.05355	
DALYs	India	Both	All Ages	Aortic aneurysm	Number	2019	374226.0017	469642.1295	277989.0085	160.43%
Deaths	India	Both	Age-standardized	Aortic aneurysm	Rate	1990	1.643012982	2.451174661	1.054555812	
Deaths	India	Both	Age-standardized	Aortic aneurysm	Rate	2019	1.677821216	2.043510772	1.302049901	2.12%
Deaths	India	Both	All Ages	Aortic aneurysm	Number	1990	5663.875809	8652.23702	3538.96068	
Deaths	India	Both	All Ages	Aortic aneurysm	Number	2019	16765.40419	20686.54921	12738.93969	196.01%
DALYs	Indonesia	Both	Age-standardized	Aortic aneurysm	Rate	1990	21.95154248	28.87205772	17.70128094	
DALYs	Indonesia	Both	Age-standardized	Aortic aneurysm	Rate	2019	29.99688264	42.0295697	21.56101751	36.65%
DALYs	Indonesia	Both	All Ages	Aortic aneurysm	Number	1990	21520.76088	28907.15046	17239.53651	
DALYs	Indonesia	Both	All Ages	Aortic aneurysm	Number	2019	61165.79016	86268.27563	43050.56299	184.22%
Deaths	Indonesia	Both	Age-standardized	Aortic aneurysm	Rate	1990	1.161701818	1.50095216	0.941495463	
Deaths	Indonesia	Both	Age-standardized	Aortic aneurysm	Rate	2019	1.669988681	2.321551783	1.226209307	43.75%
Deaths	Indonesia	Both	All Ages	Aortic aneurysm	Number	1990	887.7846198	1161.104538	715.3824176	
Deaths	Indonesia	Both	All Ages	Aortic aneurysm	Number	2019	2773.429862	3880.09705	2012.861023	212.40%
DALYs	Iran (Islamic Republic of)	Both	Age-standardized	Aortic aneurysm	Rate	1990	22.20355518	26.86402023	17.45855793	
DALYs	Iran (Islamic Republic of)	Both	Age-standardized	Aortic aneurysm	Rate	2019	19.96572611	21.98285843	17.97818532	-10.08%
DALYs	Iran (Islamic Republic of)	Both	All Ages	Aortic aneurysm	Number	1990	6096.257819	7559.356267	4724.672587	
DALYs	Iran (Islamic Republic of)	Both	All Ages	Aortic aneurysm	Number	2019	14815.23504	16363.43652	13371.16623	143.02%
Deaths	Iran (Islamic Republic of)	Both	Age-standardized	Aortic aneurysm	Rate	1990	1.107666876	1.32998432	0.879012833	
Deaths	Iran (Islamic Republic of)	Both	Age-standardized	Aortic aneurysm	Rate	2019	1.017139395	1.121849582	0.900744221	-8.17%
Deaths	Iran (Islamic Republic of)	Both	All Ages	Aortic aneurysm	Number	1990	232.9640975	282.45141	182.7930064	
Deaths	Iran (Islamic Republic of)	Both	All Ages	Aortic aneurysm	Number	2019	671.2142665	740.4145945	598.128786	188.12%
DALYs	Iraq	Both	Age-standardized	Aortic aneurysm	Rate	1990	17.23486566	22.83817402	12.68539313	
DALYs	Iraq	Both	Age-standardized	Aortic aneurysm	Rate	2019	17.33433633	21.62432451	13.45187427	0.58%
DALYs	Iraq	Both	All Ages	Aortic aneurysm	Number	1990	1516.986526	2028.065378	1108.684216	
DALYs	Iraq	Both	All Ages	Aortic aneurysm	Number	2019	4243.772387	5434.04471	3189.726167	179.75%
Deaths	Iraq	Both	Age-standardized	Aortic aneurysm	Rate	1990	0.790140071	1.02774131	0.586521885	
Deaths	Iraq	Both	Age-standardized	Aortic aneurysm	Rate	2019	0.871075297	1.040343344	0.707763531	10.24%
Deaths	Iraq	Both	All Ages	Aortic aneurysm	Number	1990	58.05442464	76.16364801	42.99704177	
Deaths	Iraq	Both	All Ages	Aortic aneurysm	Number	2019	169.9704029	210.3056596	133.9220863	192.78%
DALYs	Ireland	Both	Age-standardized	Aortic aneurysm	Rate	1990	102.4843303	108.7603507	96.61294886	
DALYs	Ireland	Both	Age-standardized	Aortic aneurysm	Rate	2019	68.56085988	76.86905294	60.63143885	-33.10%
DALYs	Ireland	Both	All Ages	Aortic aneurysm	Number	1990	4336.996561	4611.775029	4079.865948	
DALYs	Ireland	Both	All Ages	Aortic aneurysm	Number	2019	5153.893415	5782.723999	4545.446908	18.84%
Deaths	Ireland	Both	Age-standardized	Aortic aneurysm	Rate	1990	5.604777424	5.996176981	5.231798569	
Deaths	Ireland	Both	Age-standardized	Aortic aneurysm	Rate	2019	4.240873597	4.791496499	3.691595828	-24.33%
Deaths	Ireland	Both	All Ages	Aortic aneurysm	Number	1990	237.0817503	253.9724323	221.5138227	
Deaths	Ireland	Both	All Ages	Aortic aneurysm	Number	2019	330.9843331	374.3081907	287.0812139	39.61%
DALYs	Israel	Both	Age-standardized	Aortic aneurysm	Rate	1990	39.25704103	43.72490884	35.72952626	
DALYs	Israel	Both	Age-standardized	Aortic aneurysm	Rate	2019	28.9318351	31.69020136	25.92758941	-26.30%
DALYs	Israel	Both	All Ages	Aortic aneurysm	Number	1990	1907.474805	2139.530808	1732.567194	
DALYs	Israel	Both	All Ages	Aortic aneurysm	Number	2019	3311.962981	3633.742869	2964.387232	73.63%
Deaths	Israel	Both	Age-standardized	Aortic aneurysm	Rate	1990	2.218743225	2.495983273	1.980706847	
Deaths	Israel	Both	Age-standardized	Aortic aneurysm	Rate	2019	1.664057424	1.842281025	1.467180847	-25.00%
Deaths	Israel	Both	All Ages	Aortic aneurysm	Number	1990	104.8255949	117.9144522	93.18351895	
Deaths	Israel	Both	All Ages	Aortic aneurysm	Number	2019	203.9127162	226.1334553	178.8270623	94.53%
DALYs	Italy	Both	Age-standardized	Aortic aneurysm	Rate	1990	58.13068207	60.15540495	56.43327662	
DALYs	Italy	Both	Age-standardized	Aortic aneurysm	Rate	2019	46.10920484	49.31036483	42.67811077	-20.68%

DALYs	Italy	Both	All Ages	Aortic aneurysm	Number	1990	50823.7204	52607.69263	49288.64289	
DALYs	Italy	Both	All Ages	Aortic aneurysm	Number	2019	62217.59926	66624.46666	57177.86318	22.42%
Deaths	Italy	Both	Age-standardized	Aortic aneurysm	Rate	1990	2.854994644	2.995608082	2.72617409	
Deaths	Italy	Both	Age-standardized	Aortic aneurysm	Rate	2019	2.49028699	2.66686167	2.246682224	-12.77%
Deaths	Italy	Both	All Ages	Aortic aneurysm	Number	1990	2594.053441	2720.028476	2488.537407	
Deaths	Italy	Both	All Ages	Aortic aneurysm	Number	2019	4031.577376	4329.351637	3548.448856	55.42%
DALYs	Jamaica	Both	Age-standardized	Aortic aneurysm	Rate	1990	29.59707507	33.33184529	26.60874655	
DALYs	Jamaica	Both	Age-standardized	Aortic aneurysm	Rate	2019	46.79204708	58.24274611	36.83510609	58.10%
DALYs	Jamaica	Both	All Ages	Aortic aneurysm	Number	1990	531.488955	600.5623927	477.9377083	
DALYs	Jamaica	Both	All Ages	Aortic aneurysm	Number	2019	1393.674983	1734.946942	1101.659736	162.22%
Deaths	Jamaica	Both	Age-standardized	Aortic aneurysm	Rate	1990	1.49150533	1.697033323	1.326882654	
Deaths	Jamaica	Both	Age-standardized	Aortic aneurysm	Rate	2019	2.27993445	2.790192643	1.816584128	52.86%
Deaths	Jamaica	Both	All Ages	Aortic aneurysm	Number	1990	27.21741852	30.99015418	24.13805087	
Deaths	Jamaica	Both	All Ages	Aortic aneurysm	Number	2019	70.24719562	85.72122834	56.17297024	158.10%
DALYs	Japan	Both	Age-standardized	Aortic aneurysm	Rate	1990	49.78321871	51.39088716	47.43843882	
DALYs	Japan	Both	Age-standardized	Aortic aneurysm	Rate	2019	78.67401913	83.96194855	70.802082	58.03%
DALYs	Japan	Both	All Ages	Aortic aneurysm	Number	1990	83780.53574	86455.84862	79960.51776	
DALYs	Japan	Both	All Ages	Aortic aneurysm	Number	2019	268760.1388	290468.0226	230931.6602	220.79%
Deaths	Japan	Both	Age-standardized	Aortic aneurysm	Rate	1990	2.830382683	2.949957631	2.641427993	
Deaths	Japan	Both	Age-standardized	Aortic aneurysm	Rate	2019	4.513351085	4.907097107	3.834628498	59.46%
Deaths	Japan	Both	All Ages	Aortic aneurysm	Number	1990	4606.781519	4790.932816	4328.490763	
Deaths	Japan	Both	All Ages	Aortic aneurysm	Number	2019	20168.88885	22321.83294	16270.80103	337.81%
DALYs	Jordan	Both	Age-standardized	Aortic aneurysm	Rate	1990	38.67284927	48.74266703	30.52608693	
DALYs	Jordan	Both	Age-standardized	Aortic aneurysm	Rate	2019	27.73542737	34.91262604	22.39133199	-28.28%
DALYs	Jordan	Both	All Ages	Aortic aneurysm	Number	1990	637.5220005	803.2818455	494.4804923	
DALYs	Jordan	Both	All Ages	Aortic aneurysm	Number	2019	2097.408817	2649.046799	1676.777773	228.99%
Deaths	Jordan	Both	Age-standardized	Aortic aneurysm	Rate	1990	1.746921822	2.185373336	1.398961769	
Deaths	Jordan	Both	Age-standardized	Aortic aneurysm	Rate	2019	1.305232775	1.620518825	1.059365284	-25.28%
Deaths	Jordan	Both	All Ages	Aortic aneurysm	Number	1990	21.4154909	26.94640148	16.90623534	
Deaths	Jordan	Both	All Ages	Aortic aneurysm	Number	2019	75.45579462	94.94264213	60.95784898	252.34%
DALYs	Kazakhstan	Both	Age-standardized	Aortic aneurysm	Rate	1990	37.97794571	48.33452304	32.65204765	
DALYs	Kazakhstan	Both	Age-standardized	Aortic aneurysm	Rate	2019	57.38023587	67.90094075	48.29612489	51.09%
DALYs	Kazakhstan	Both	All Ages	Aortic aneurysm	Number	1990	5144.342131	6522.103258	4420.385849	
DALYs	Kazakhstan	Both	All Ages	Aortic aneurysm	Number	2019	10628.17336	12685.91937	8925.118895	106.60%
Deaths	Kazakhstan	Both	Age-standardized	Aortic aneurysm	Rate	1990	1.481266493	1.882159828	1.26310173	
Deaths	Kazakhstan	Both	Age-standardized	Aortic aneurysm	Rate	2019	2.305286183	2.695227145	1.958266831	55.63%
Deaths	Kazakhstan	Both	All Ages	Aortic aneurysm	Number	1990	184.681688	233.4439045	157.8211009	
Deaths	Kazakhstan	Both	All Ages	Aortic aneurysm	Number	2019	391.3385258	459.9387915	330.7886087	111.90%
DALYs	Kenya	Both	Age-standardized	Aortic aneurysm	Rate	1990	43.89079185	57.70181314	29.94770108	
DALYs	Kenya	Both	Age-standardized	Aortic aneurysm	Rate	2019	43.85164598	53.7266217	36.65802592	-0.09%
DALYs	Kenya	Both	All Ages	Aortic aneurysm	Number	1990	3792.245909	5036.285624	2588.908229	
DALYs	Kenya	Both	All Ages	Aortic aneurysm	Number	2019	10076.83967	12472.21502	8431.070653	165.72%
Deaths	Kenya	Both	Age-standardized	Aortic aneurysm	Rate	1990	2.272437412	2.958496573	1.531921296	
Deaths	Kenya	Both	Age-standardized	Aortic aneurysm	Rate	2019	2.280064095	2.740032482	1.864303856	0.34%
Deaths	Kenya	Both	All Ages	Aortic aneurysm	Number	1990	159.8718072	209.2736817	109.0024265	
Deaths	Kenya	Both	All Ages	Aortic aneurysm	Number	2019	406.5113131	495.5734008	340.0071241	154.27%
DALYs	Kiribati	Both	Age-standardized	Aortic aneurysm	Rate	1990	36.08086444	43.1021207	29.55645489	
DALYs	Kiribati	Both	Age-standardized	Aortic aneurysm	Rate	2019	30.65845322	37.78609207	24.41674921	-15.03%
DALYs	Kiribati	Both	All Ages	Aortic aneurysm	Number	1990	14.95134841	18.16919647	12.05121317	
DALYs	Kiribati	Both	All Ages	Aortic aneurysm	Number	2019	23.58337646	30.43646438	18.0273548	57.73%
Deaths	Kiribati	Both	Age-standardized	Aortic aneurysm	Rate	1990	1.54708492	1.814197891	1.286103094	
Deaths	Kiribati	Both	Age-standardized	Aortic aneurysm	Rate	2019	1.373239851	1.651263056	1.139709706	-11.24%
Deaths	Kiribati	Both	All Ages	Aortic aneurysm	Number	1990	0.500433573	0.601219076	0.408918643	
Deaths	Kiribati	Both	All Ages	Aortic aneurysm	Number	2019	0.774099358	0.958902433	0.612443809	54.69%
DALYs	Kuwait	Both	Age-standardized	Aortic aneurysm	Rate	1990	24.46129405	28.22128164	20.84399637	
DALYs	Kuwait	Both	Age-standardized	Aortic aneurysm	Rate	2019	26.49765503	32.66347094	20.92243957	8.32%
DALYs	Kuwait	Both	All Ages	Aortic aneurysm	Number	1990	192.2198824	217.5889784	167.2057649	
DALYs	Kuwait	Both	All Ages	Aortic aneurysm	Number	2019	724.183846	881.4095583	585.4106428	276.75%
Deaths	Kuwait	Both	Age-standardized	Aortic aneurysm	Rate	1990	1.161247366	1.364815352	0.960901168	
Deaths	Kuwait	Both	Age-standardized	Aortic aneurysm	Rate	2019	1.432622525	1.77179289	1.11115435	23.37%
Deaths	Kuwait	Both	All Ages	Aortic aneurysm	Number	1990	6.384739432	7.294744445	5.492516744	
Deaths	Kuwait	Both	All Ages	Aortic aneurysm	Number	2019	30.06560201	36.75784207	23.83175879	370.90%
DALYs	Kyrgyzstan	Both	Age-standardized	Aortic aneurysm	Rate	1990	17.50664199	20.17921821	15.36748638	
DALYs	Kyrgyzstan	Both	Age-standardized	Aortic aneurysm	Rate	2019	18.78951741	21.75665007	16.21734367	7.33%
DALYs	Kyrgyzstan	Both	All Ages	Aortic aneurysm	Number	1990	555.1291859	641.7117965	486.1704638	
DALYs	Kyrgyzstan	Both	All Ages	Aortic aneurysm	Number	2019	889.244644	1033.554537	762.0731082	60.19%
Deaths	Kyrgyzstan	Both	Age-standardized	Aortic aneurysm	Rate	1990	0.742877603	0.855777207	0.650401107	
Deaths	Kyrgyzstan	Both	Age-standardized	Aortic aneurysm	Rate	2019	0.933785897	1.073457442	0.802670117	25.70%
Deaths	Kyrgyzstan	Both	All Ages	Aortic aneurysm	Number	1990	22.1991439	25.5336843	19.47032099	
Deaths	Kyrgyzstan	Both	All Ages	Aortic aneurysm	Number	2019	37.66112316	43.6074417	32.39256359	69.65%
DALYs	Lao People's Democratic Republic	Both	Age-standardized	Aortic aneurysm	Rate	1990	23.6173519	36.95611449	14.65900497	
DALYs	Lao People's Democratic Republic	Both	Age-standardized	Aortic aneurysm	Rate	2019	28.96483544	37.01084792	21.61422023	22.64%
DALYs	Lao People's Democratic Republic	Both	All Ages	Aortic aneurysm	Number	1990	483.0576864	785.0291965	291.7004253	
DALYs	Lao People's Democratic Republic	Both	All Ages	Aortic aneurysm	Number	2019	1265.461954	1656.117656	922.9847747	161.97%
Deaths	Lao People's Democratic Republic	Both	Age-standardized	Aortic aneurysm	Rate	1990	1.222811343	1.819492397	0.786669428	
Deaths	Lao People's Democratic Republic	Both	Age-standardized	Aortic aneurysm	Rate	2019	1.537815655	1.917876814	1.198624903	25.76%
Deaths	Lao People's Democratic Republic	Both	All Ages	Aortic aneurysm	Number	1990	20.11879853	31.17007451	12.50711435	
Deaths	Lao People's Democratic Republic	Both	All Ages	Aortic aneurysm	Number	2019	54.08459526	68.35150974	40.9423273	168.83%
DALYs	Latvia	Both	Age-standardized	Aortic aneurysm	Rate	1990	46.45083591	52.58004606	41.25719027	
DALYs	Latvia	Both	Age-standardized	Aortic aneurysm	Rate	2019	47.00392564	58.481671	38.3255652	1.19%
DALYs	Latvia	Both	All Ages	Aortic aneurysm	Number	1990	1621.21873	1841.195693	1433.064944	
DALYs	Latvia	Both	All Ages	Aortic aneurysm	Number	2019	1695.018544	2087.803774	1388.782355	4.55%
Deaths	Latvia	Both	Age-standardized	Aortic aneurysm	Rate	1990	2.0583275	2.319908273	1.82848763	
Deaths	Latvia	Both	Age-standardized	Aortic aneurysm	Rate	2019	2.155793246	2.616066383	1.783558082	4.74%
Deaths	Latvia	Both	All Ages	Aortic aneurysm	Number	1990	73.18015517	82.41290488	64.9787248	
Deaths	Latvia	Both	All Ages	Aortic aneurysm	Number	2019	88.98559144	107.7044702	74.00382552	21.60%
DALYs	Lebanon	Both	Age-standardized	Aortic aneurysm	Rate	1990	30.7443694	41.2742634	22.46266846	
DALYs	Lebanon	Both	Age-standardized	Aortic aneurysm	Rate	2019	27.70629042	35.17279813	20.60326166	-9.88%
DALYs	Lebanon	Both	All Ages	Aortic aneurysm	Number	1990	710.6022676	965.1611985	513.0788474	
DALYs	Lebanon	Both	All Ages	Aortic aneurysm	Number	2019	1455.009357	1847.047233	1082.068237	104.76%
Deaths	Lebanon	Both	Age-standardized	Aortic aneurysm	Rate	1990	1.4904896	1.971140621	1.09287012	
Deaths	Lebanon	Both	Age-standardized	Aortic aneurysm	Rate	2019	1.37296889	1.699358635	1.02532318	-7.88%
Deaths	Lebanon	Both	All Ages	Aortic aneurysm	Number	1990	29.53775727	39.45172374	21.68180256	
Deaths	Lebanon	Both	All Ages	Aortic aneurysm	Number	2019	71.22905652	88.0950514	53.32027581	141.15%
DALYs	Lesotho	Both	Age-standardized	Aortic aneurysm	Rate	1990	44.91753558	61.3883486	29.98562074	
DALYs	Lesotho	Both	Age-standardized	Aortic aneurysm	Rate	2019	47.15535416	65.93610612	32.30142468	4.98%
DALYs	Lesotho	Both	All Ages	Aortic aneurysm	Number	1990	441.4874537	609.6533121	290.5078036	
DALYs	Lesotho	Both	All Ages	Aortic aneurysm	Number	2019	614.6094361	875.5514116	402.0933865	39.21%
Deaths	Lesotho	Both	Age-standardized	Aortic aneurysm	Rate	1990	2.289153516	3.084797113	1.572122481	
Deaths	Lesotho	Both	Age-standardized	Aortic aneurysm	Rate	2019	2.329237826	3.151066322	1.684907189	1.75%
Deaths	Lesotho	Both	All Ages	Aortic aneurysm	Number	1990	19.37145274	26.4790805	12.95015706	

Deaths	Lesotho	Both	All Ages	Aortic aneurysm	Number	2019	24.56854258	34.10957295	17.12737257	26.83%
DALYs	Liberia	Both	Age-standardized	Aortic aneurysm	Rate	1990	37.58261984	52.48310776	24.25664993	
DALYs	Liberia	Both	Age-standardized	Aortic aneurysm	Rate	2019	27.58706348	38.82555227	18.90013612	-26.60%
DALYs	Liberia	Both	All Ages	Aortic aneurysm	Number	1990	419.9214634	592.0370863	268.4776051	
DALYs	Liberia	Both	All Ages	Aortic aneurysm	Number	2019	590.3733387	847.6769125	391.1974215	40.59%
Deaths	Liberia	Both	Age-standardized	Aortic aneurysm	Rate	1990	1.898918925	2.622264182	1.245608298	
Deaths	Liberia	Both	Age-standardized	Aortic aneurysm	Rate	2019	1.425761166	1.967594358	0.999128151	-24.92%
Deaths	Liberia	Both	All Ages	Aortic aneurysm	Number	1990	18.67680277	26.08267694	12.0368311	
Deaths	Liberia	Both	All Ages	Aortic aneurysm	Number	2019	24.26533202	33.99875379	16.63589517	29.92%
DALYs	Libya	Both	Age-standardized	Aortic aneurysm	Rate	1990	16.3313737	23.60472489	11.76297193	
DALYs	Libya	Both	Age-standardized	Aortic aneurysm	Rate	2019	18.72142918	28.74167525	11.61325361	14.63%
DALYs	Libya	Both	All Ages	Aortic aneurysm	Number	1990	350.1554516	510.8493794	249.9451345	
DALYs	Libya	Both	All Ages	Aortic aneurysm	Number	2019	1034.246381	1597.565629	634.9399917	195.37%
Deaths	Libya	Both	Age-standardized	Aortic aneurysm	Rate	1990	0.780008583	1.11372606	0.560587885	
Deaths	Libya	Both	Age-standardized	Aortic aneurysm	Rate	2019	0.897019615	1.361975717	0.565559969	15.00%
Deaths	Libya	Both	All Ages	Aortic aneurysm	Number	1990	13.6396373	19.5959662	9.779049396	
Deaths	Libya	Both	All Ages	Aortic aneurysm	Number	2019	41.77762735	62.95036466	25.90238305	206.30%
DALYs	Lithuania	Both	Age-standardized	Aortic aneurysm	Rate	1990	46.450822	54.40930009	41.17863905	
DALYs	Lithuania	Both	Age-standardized	Aortic aneurysm	Rate	2019	64.21808078	78.17112939	51.70052865	38.25%
DALYs	Lithuania	Both	All Ages	Aortic aneurysm	Number	1990	2057.417294	2409.422224	1821.083896	
DALYs	Lithuania	Both	All Ages	Aortic aneurysm	Number	2019	3218.225364	3911.862731	2588.561207	56.42%
Deaths	Lithuania	Both	Age-standardized	Aortic aneurysm	Rate	1990	1.892700585	2.196430407	1.677446841	
Deaths	Lithuania	Both	Age-standardized	Aortic aneurysm	Rate	2019	2.725656276	3.298528136	2.201282932	44.01%
Deaths	Lithuania	Both	All Ages	Aortic aneurysm	Number	1990	85.03424246	98.73904076	75.28345242	
Deaths	Lithuania	Both	All Ages	Aortic aneurysm	Number	2019	156.446734	189.0134688	127.1251222	83.98%
DALYs	Luxembourg	Both	Age-standardized	Aortic aneurysm	Rate	1990	77.76944501	84.55457837	71.47365503	
DALYs	Luxembourg	Both	Age-standardized	Aortic aneurysm	Rate	2019	46.73262516	54.54573573	40.12126509	-39.91%
DALYs	Luxembourg	Both	All Ages	Aortic aneurysm	Number	1990	418.3421021	456.9521018	383.0966817	
DALYs	Luxembourg	Both	All Ages	Aortic aneurysm	Number	2019	458.3308639	533.2322941	394.5703093	9.56%
Deaths	Luxembourg	Both	Age-standardized	Aortic aneurysm	Rate	1990	3.924900343	4.277782384	3.589529106	
Deaths	Luxembourg	Both	Age-standardized	Aortic aneurysm	Rate	2019	2.613004161	3.069689835	2.229337451	-33.42%
Deaths	Luxembourg	Both	All Ages	Aortic aneurysm	Number	1990	21.68057393	23.71740408	19.73507697	
Deaths	Luxembourg	Both	All Ages	Aortic aneurysm	Number	2019	27.86916685	32.63035945	23.70209989	28.54%
DALYs	Madagascar	Both	Age-standardized	Aortic aneurysm	Rate	1990	92.56423901	125.4575083	60.1452693	
DALYs	Madagascar	Both	Age-standardized	Aortic aneurysm	Rate	2019	67.52513989	93.91956124	46.66357544	-27.05%
DALYs	Madagascar	Both	All Ages	Aortic aneurysm	Number	1990	5350.63921	7368.255665	3457.780891	
DALYs	Madagascar	Both	All Ages	Aortic aneurysm	Number	2019	8596.175713	12155.17423	5909.770299	60.66%
Deaths	Madagascar	Both	Age-standardized	Aortic aneurysm	Rate	1990	4.220022647	5.588371955	2.728000046	
Deaths	Madagascar	Both	Age-standardized	Aortic aneurysm	Rate	2019	3.095703741	4.2445757	2.167303223	-26.64%
Deaths	Madagascar	Both	All Ages	Aortic aneurysm	Number	1990	197.0322252	264.729095	127.3269991	
Deaths	Madagascar	Both	All Ages	Aortic aneurysm	Number	2019	291.8009116	406.8582217	200.2774324	48.10%
DALYs	Malawi	Both	Age-standardized	Aortic aneurysm	Rate	1990	54.76081293	77.33557123	28.05347615	
DALYs	Malawi	Both	Age-standardized	Aortic aneurysm	Rate	2019	46.58366077	63.42878094	30.74285614	-14.93%
DALYs	Malawi	Both	All Ages	Aortic aneurysm	Number	1990	2187.268254	3128.167144	1123.036425	
DALYs	Malawi	Both	All Ages	Aortic aneurysm	Number	2019	3665.118154	5095.307126	2387.150041	67.57%
Deaths	Malawi	Both	Age-standardized	Aortic aneurysm	Rate	1990	2.748638741	3.810470064	1.407538996	
Deaths	Malawi	Both	Age-standardized	Aortic aneurysm	Rate	2019	2.312104894	3.052004312	1.534614108	-15.88%
Deaths	Malawi	Both	All Ages	Aortic aneurysm	Number	1990	86.79238362	123.2932031	44.46934817	
Deaths	Malawi	Both	All Ages	Aortic aneurysm	Number	2019	145.8219343	196.3695196	95.98666487	68.01%
DALYs	Malaysia	Both	Age-standardized	Aortic aneurysm	Rate	1990	77.33511015	91.55267793	65.62356347	
DALYs	Malaysia	Both	Age-standardized	Aortic aneurysm	Rate	2019	74.92277767	94.09277548	59.3415273	-3.12%
DALYs	Malaysia	Both	All Ages	Aortic aneurysm	Number	1990	6814.423137	8069.808431	5782.542508	
DALYs	Malaysia	Both	All Ages	Aortic aneurysm	Number	2019	18892.48838	23725.33398	14910.04497	177.24%
Deaths	Malaysia	Both	Age-standardized	Aortic aneurysm	Rate	1990	4.229670685	4.97803506	3.570672271	
Deaths	Malaysia	Both	Age-standardized	Aortic aneurysm	Rate	2019	4.39753203	5.451635361	3.508050558	3.97%
Deaths	Malaysia	Both	All Ages	Aortic aneurysm	Number	1990	325.5519151	384.1462225	275.8081077	
Deaths	Malaysia	Both	All Ages	Aortic aneurysm	Number	2019	970.5655998	1212.983419	770.0088278	198.13%
DALYs	Maldives	Both	Age-standardized	Aortic aneurysm	Rate	1990	44.65954863	62.74856907	30.57938543	
DALYs	Maldives	Both	Age-standardized	Aortic aneurysm	Rate	2019	34.9145966	41.8772382	28.9371281	-21.82%
DALYs	Maldives	Both	All Ages	Aortic aneurysm	Number	1990	34.63731332	49.29850412	22.65741147	
DALYs	Maldives	Both	All Ages	Aortic aneurysm	Number	2019	99.8973058	120.0315263	82.81766716	188.41%
Deaths	Maldives	Both	Age-standardized	Aortic aneurysm	Rate	1990	2.724143137	3.78111954	1.899874497	
Deaths	Maldives	Both	Age-standardized	Aortic aneurysm	Rate	2019	2.336240158	2.820579464	1.935534182	-14.24%
Deaths	Maldives	Both	All Ages	Aortic aneurysm	Number	1990	1.50535406	2.113327184	1.01884943	
Deaths	Maldives	Both	All Ages	Aortic aneurysm	Number	2019	5.627257847	6.748795991	4.689902184	273.82%
DALYs	Mali	Both	Age-standardized	Aortic aneurysm	Rate	1990	36.07161865	51.46523764	20.32626352	
DALYs	Mali	Both	Age-standardized	Aortic aneurysm	Rate	2019	28.00383483	39.93434497	18.45924553	-22.37%
DALYs	Mali	Both	All Ages	Aortic aneurysm	Number	1990	1493.676571	2196.044724	827.6701269	
DALYs	Mali	Both	All Ages	Aortic aneurysm	Number	2019	2445.921116	3532.618023	1578.122425	63.75%
Deaths	Mali	Both	Age-standardized	Aortic aneurysm	Rate	1990	1.825552509	2.531355531	1.048293141	
Deaths	Mali	Both	Age-standardized	Aortic aneurysm	Rate	2019	1.479591251	2.070593011	0.998703287	-18.95%
Deaths	Mali	Both	All Ages	Aortic aneurysm	Number	1990	62.27927109	88.21045046	35.39761646	
Deaths	Mali	Both	All Ages	Aortic aneurysm	Number	2019	105.4212617	149.1757908	70.08395785	69.27%
DALYs	Malta	Both	Age-standardized	Aortic aneurysm	Rate	1990	44.49513719	48.88478151	40.30694739	
DALYs	Malta	Both	Age-standardized	Aortic aneurysm	Rate	2019	33.1805621	38.9135419	28.15270593	-25.43%
DALYs	Malta	Both	All Ages	Aortic aneurysm	Number	1990	190.518083	210.0590501	172.2785268	
DALYs	Malta	Both	All Ages	Aortic aneurysm	Number	2019	294.6431584	346.0951625	248.4969145	54.65%
Deaths	Malta	Both	Age-standardized	Aortic aneurysm	Rate	1990	2.344317576	2.58148037	2.113303441	
Deaths	Malta	Both	Age-standardized	Aortic aneurysm	Rate	2019	1.759077903	2.066049892	1.485116622	-24.96%
Deaths	Malta	Both	All Ages	Aortic aneurysm	Number	1990	9.8152853	10.84725903	8.846054277	
Deaths	Malta	Both	All Ages	Aortic aneurysm	Number	2019	17.26064734	20.25419686	14.50743599	75.85%
DALYs	Marshall Islands	Both	Age-standardized	Aortic aneurysm	Rate	1990	100.3100238	151.9976624	70.18959189	
DALYs	Marshall Islands	Both	Age-standardized	Aortic aneurysm	Rate	2019	88.95710409	124.5713085	62.22024881	-11.32%
DALYs	Marshall Islands	Both	All Ages	Aortic aneurysm	Number	1990	17.5726515	27.57562932	11.64325194	
DALYs	Marshall Islands	Both	All Ages	Aortic aneurysm	Number	2019	33.09980588	47.87995828	22.15264859	88.36%
Deaths	Marshall Islands	Both	Age-standardized	Aortic aneurysm	Rate	1990	4.856474255	7.208092377	3.484371941	
Deaths	Marshall Islands	Both	Age-standardized	Aortic aneurysm	Rate	2019	4.194401882	5.67830032	3.031394171	-13.63%
Deaths	Marshall Islands	Both	All Ages	Aortic aneurysm	Number	1990	0.664301735	1.002401658	0.464763587	
Deaths	Marshall Islands	Both	All Ages	Aortic aneurysm	Number	2019	1.140312943	1.611543923	0.794215907	71.66%
DALYs	Mauritania	Both	Age-standardized	Aortic aneurysm	Rate	1990	45.68877628	57.47756224	33.8863353	
DALYs	Mauritania	Both	Age-standardized	Aortic aneurysm	Rate	2019	27.01676373	36.3852972	19.81752236	-40.87%
DALYs	Mauritania	Both	All Ages	Aortic aneurysm	Number	1990	468.3314523	596.7157976	344.9141189	
DALYs	Mauritania	Both	All Ages	Aortic aneurysm	Number	2019	565.2266451	780.0470257	403.6724768	20.69%
Deaths	Mauritania	Both	Age-standardized	Aortic aneurysm	Rate	1990	2.254395877	2.799156883	1.681405208	
Deaths	Mauritania	Both	Age-standardized	Aortic aneurysm	Rate	2019	1.445662857	1.865773205	1.132079804	-35.87%
Deaths	Mauritania	Both	All Ages	Aortic aneurysm	Number	1990	20.2959802	25.28081472	15.18247827	
Deaths	Mauritania	Both	All Ages	Aortic aneurysm	Number	2019	26.38030581	34.73678008	20.129851	29.98%
DALYs	Mauritius	Both	Age-standardized	Aortic aneurysm	Rate	1990	24.14554159	26.49888992	21.95513546	
DALYs	Mauritius	Both	Age-standardized	Aortic aneurysm	Rate	2019	22.00082722	27.035925	17.54631047	-8.88%
DALYs	Mauritius	Both	All Ages	Aortic aneurysm	Number	1990	192.3241302	210.4150502	175.1837056	
DALYs	Mauritius	Both	All Ages	Aortic aneurysm	Number	2019	364.7197163	450.1290039	290.4920695	89.64%

Deaths	Mauritius	Both	Age-standardized	Aortic aneurysm	Rate	1990	1.130046313	1.245482179	1.022195627	
Deaths	Mauritius	Both	Age-standardized	Aortic aneurysm	Rate	2019	1.073764606	1.29812299	0.861360899	-4.98%
Deaths	Mauritius	Both	All Ages	Aortic aneurysm	Number	1990	7.448605543	8.182009476	6.748511189	
Deaths	Mauritius	Both	All Ages	Aortic aneurysm	Number	2019	17.15000527	20.82188992	13.65154588	130.24%
DALYs	Mexico	Both	Age-standardized	Aortic aneurysm	Rate	1990	20.32528358	21.04993214	19.56321183	
DALYs	Mexico	Both	Age-standardized	Aortic aneurysm	Rate	2019	19.37059024	22.91630758	16.02464742	-4.70%
DALYs	Mexico	Both	All Ages	Aortic aneurysm	Number	1990	9255.495846	9558.651005	8969.138944	
DALYs	Mexico	Both	All Ages	Aortic aneurysm	Number	2019	22667.72307	26914.56917	18721.5923	144.91%
Deaths	Mexico	Both	Age-standardized	Aortic aneurysm	Rate	1990	1.024409652	1.06816676	0.961727011	
Deaths	Mexico	Both	Age-standardized	Aortic aneurysm	Rate	2019	0.998814477	1.165355745	0.831317205	-2.50%
Deaths	Mexico	Both	All Ages	Aortic aneurysm	Number	1990	391.2495764	405.7975994	372.9357868	
Deaths	Mexico	Both	All Ages	Aortic aneurysm	Number	2019	1098.517625	1286.359052	912.3665905	180.77%
DALYs	Micronesia (Federated States of)	Both	Age-standardized	Aortic aneurysm	Rate	1990	106.8228514	164.6438371	71.1867054	
DALYs	Micronesia (Federated States of)	Both	Age-standardized	Aortic aneurysm	Rate	2019	93.97192198	134.4256792	58.81930041	-12.03%
DALYs	Micronesia (Federated States of)	Both	All Ages	Aortic aneurysm	Number	1990	52.59818144	83.16652752	32.6120779	
DALYs	Micronesia (Federated States of)	Both	All Ages	Aortic aneurysm	Number	2019	68.72406476	101.7002383	39.87376265	30.66%
Deaths	Micronesia (Federated States of)	Both	Age-standardized	Aortic aneurysm	Rate	1990	5.08602569	7.438782308	3.593795834	
Deaths	Micronesia (Federated States of)	Both	Age-standardized	Aortic aneurysm	Rate	2019	4.457176895	6.10198313	3.05459775	-12.36%
Deaths	Micronesia (Federated States of)	Both	All Ages	Aortic aneurysm	Number	1990	1.955850652	2.966500862	1.323619637	
Deaths	Micronesia (Federated States of)	Both	All Ages	Aortic aneurysm	Number	2019	2.503791683	3.56358565	1.584989343	28.02%
DALYs	Monaco	Both	Age-standardized	Aortic aneurysm	Rate	1990	93.04390843	114.8818381	72.12909009	
DALYs	Monaco	Both	Age-standardized	Aortic aneurysm	Rate	2019	76.3479355	92.92657645	58.67570033	-17.94%
DALYs	Monaco	Both	All Ages	Aortic aneurysm	Number	1990	63.20501769	77.39610838	48.65499229	
DALYs	Monaco	Both	All Ages	Aortic aneurysm	Number	2019	69.61477331	83.39333037	54.52048146	10.14%
Deaths	Monaco	Both	Age-standardized	Aortic aneurysm	Rate	1990	5.055134462	6.147752156	3.955302573	
Deaths	Monaco	Both	Age-standardized	Aortic aneurysm	Rate	2019	4.335064239	5.144442744	3.4348122	-14.24%
Deaths	Monaco	Both	All Ages	Aortic aneurysm	Number	1990	3.975624889	4.815496693	3.115293896	
Deaths	Monaco	Both	All Ages	Aortic aneurysm	Number	2019	4.70664051	5.554504809	3.738734766	18.39%
DALYs	Mongolia	Both	Age-standardized	Aortic aneurysm	Rate	1990	23.52491961	28.10494579	19.25673353	
DALYs	Mongolia	Both	Age-standardized	Aortic aneurysm	Rate	2019	24.2470745	30.94809299	19.24635837	3.07%
DALYs	Mongolia	Both	All Ages	Aortic aneurysm	Number	1990	255.5296946	310.092305	207.8197711	
DALYs	Mongolia	Both	All Ages	Aortic aneurysm	Number	2019	603.2750389	786.3694056	467.3916897	136.09%
Deaths	Mongolia	Both	Age-standardized	Aortic aneurysm	Rate	1990	1.136522873	1.336000583	0.941390238	
Deaths	Mongolia	Both	Age-standardized	Aortic aneurysm	Rate	2019	1.181039384	1.44515864	0.966946356	3.92%
Deaths	Mongolia	Both	All Ages	Aortic aneurysm	Number	1990	10.41649767	12.38839669	8.616658248	
Deaths	Mongolia	Both	All Ages	Aortic aneurysm	Number	2019	22.09216142	28.22339917	17.42612594	112.09%
DALYs	Montenegro	Both	Age-standardized	Aortic aneurysm	Rate	1990	155.7131732	182.6588546	137.5509141	
DALYs	Montenegro	Both	Age-standardized	Aortic aneurysm	Rate	2019	178.3364421	219.6558811	144.8590753	14.53%
DALYs	Montenegro	Both	All Ages	Aortic aneurysm	Number	1990	990.5494347	1161.081591	874.525247	
DALYs	Montenegro	Both	All Ages	Aortic aneurysm	Number	2019	1680.19841	2075.060871	1360.264373	69.62%
Deaths	Montenegro	Both	Age-standardized	Aortic aneurysm	Rate	1990	6.519300107	7.638437685	5.780441456	
Deaths	Montenegro	Both	Age-standardized	Aortic aneurysm	Rate	2019	8.261407181	10.15245399	6.786419312	26.72%
Deaths	Montenegro	Both	All Ages	Aortic aneurysm	Number	1990	39.31416061	46.03999961	34.84686653	
Deaths	Montenegro	Both	All Ages	Aortic aneurysm	Number	2019	79.22771471	97.23347384	64.69280711	101.52%
DALYs	Morocco	Both	Age-standardized	Aortic aneurysm	Rate	1990	20.62578391	29.56031351	13.46657781	
DALYs	Morocco	Both	Age-standardized	Aortic aneurysm	Rate	2019	24.16011574	31.54725366	17.24021709	17.14%
DALYs	Morocco	Both	All Ages	Aortic aneurysm	Number	1990	2924.662191	4292.883467	1887.738154	
DALYs	Morocco	Both	All Ages	Aortic aneurysm	Number	2019	7361.052042	9795.594125	5140.207983	151.69%
Deaths	Morocco	Both	Age-standardized	Aortic aneurysm	Rate	1990	1.026515743	1.421547043	0.69757839	
Deaths	Morocco	Both	Age-standardized	Aortic aneurysm	Rate	2019	1.28597424	1.621067117	0.948508603	25.28%
Deaths	Morocco	Both	All Ages	Aortic aneurysm	Number	1990	120.7809227	171.9494322	79.76139263	
Deaths	Morocco	Both	All Ages	Aortic aneurysm	Number	2019	337.9617071	434.923961	243.9669521	179.81%
DALYs	Mozambique	Both	Age-standardized	Aortic aneurysm	Rate	1990	55.96988348	83.13991072	27.27238735	
DALYs	Mozambique	Both	Age-standardized	Aortic aneurysm	Rate	2019	60.04981081	89.66265718	35.74835715	7.29%
DALYs	Mozambique	Both	All Ages	Aortic aneurysm	Number	1990	3331.258835	5014.705231	1610.912256	
DALYs	Mozambique	Both	All Ages	Aortic aneurysm	Number	2019	7050.701892	10936.61786	4210.60036	111.65%
Deaths	Mozambique	Both	Age-standardized	Aortic aneurysm	Rate	1990	2.858336022	4.168426799	1.402422996	
Deaths	Mozambique	Both	Age-standardized	Aortic aneurysm	Rate	2019	2.917349254	4.222226046	1.760974421	2.06%
Deaths	Mozambique	Both	All Ages	Aortic aneurysm	Number	1990	138.3688195	205.7962202	67.44133048	
Deaths	Mozambique	Both	All Ages	Aortic aneurysm	Number	2019	271.9591415	403.6895877	162.1343926	96.55%
DALYs	Myanmar	Both	Age-standardized	Aortic aneurysm	Rate	1990	25.46578575	37.54712226	16.00629199	
DALYs	Myanmar	Both	Age-standardized	Aortic aneurysm	Rate	2019	27.60217259	34.50038588	21.70285829	8.39%
DALYs	Myanmar	Both	All Ages	Aortic aneurysm	Number	1990	6100.129974	9216.766425	3740.180278	
DALYs	Myanmar	Both	All Ages	Aortic aneurysm	Number	2019	12528.63035	15838.09275	9750.889551	105.38%
Deaths	Myanmar	Both	Age-standardized	Aortic aneurysm	Rate	1990	1.250245069	1.78258177	0.835523636	
Deaths	Myanmar	Both	Age-standardized	Aortic aneurysm	Rate	2019	1.440618247	1.756185077	1.16610762	15.23%
Deaths	Myanmar	Both	All Ages	Aortic aneurysm	Number	1990	242.1031078	352.703026	153.8182762	
Deaths	Myanmar	Both	All Ages	Aortic aneurysm	Number	2019	566.426632	698.7333935	453.7510694	133.96%
DALYs	Namibia	Both	Age-standardized	Aortic aneurysm	Rate	1990	59.70368817	72.53337985	47.43729069	
DALYs	Namibia	Both	Age-standardized	Aortic aneurysm	Rate	2019	49.0884676	64.85155351	36.74120095	-17.78%
DALYs	Namibia	Both	All Ages	Aortic aneurysm	Number	1990	433.0848792	529.9644965	334.9129765	
DALYs	Namibia	Both	All Ages	Aortic aneurysm	Number	2019	698.4046363	951.3055629	510.989722	61.26%
Deaths	Namibia	Both	Age-standardized	Aortic aneurysm	Rate	1990	3.083813064	3.714833652	2.510084401	
Deaths	Namibia	Both	Age-standardized	Aortic aneurysm	Rate	2019	2.620367948	3.320708866	2.014025067	-15.03%
Deaths	Namibia	Both	All Ages	Aortic aneurysm	Number	1990	19.00952862	22.92596164	15.20445342	
Deaths	Namibia	Both	All Ages	Aortic aneurysm	Number	2019	32.21563212	41.51694426	24.49926927	69.47%
DALYs	Nauru	Both	Age-standardized	Aortic aneurysm	Rate	1990	119.3388135	167.4658968	78.39304265	
DALYs	Nauru	Both	Age-standardized	Aortic aneurysm	Rate	2019	97.61621167	131.5529855	65.24767575	-18.20%
DALYs	Nauru	Both	All Ages	Aortic aneurysm	Number	1990	5.348586982	7.903517803	3.140993235	
DALYs	Nauru	Both	All Ages	Aortic aneurysm	Number	2019	5.112979131	7.282947172	3.084571708	-4.41%
Deaths	Nauru	Both	Age-standardized	Aortic aneurysm	Rate	1990	5.575909365	7.474978561	3.935214231	
Deaths	Nauru	Both	Age-standardized	Aortic aneurysm	Rate	2019	4.509452777	5.869168503	3.232023743	-19.13%
Deaths	Nauru	Both	All Ages	Aortic aneurysm	Number	1990	0.169326449	0.241174514	0.108037739	
Deaths	Nauru	Both	All Ages	Aortic aneurysm	Number	2019	0.148101745	0.204249126	0.094558088	-12.53%
DALYs	Nepal	Both	Age-standardized	Aortic aneurysm	Rate	1990	23.58643815	40.05429832	11.99394734	
DALYs	Nepal	Both	Age-standardized	Aortic aneurysm	Rate	2019	31.66072962	43.73640004	19.86767014	34.23%
DALYs	Nepal	Both	All Ages	Aortic aneurysm	Number	1990	2208.180711	3844.311804	1093.684884	
DALYs	Nepal	Both	All Ages	Aortic aneurysm	Number	2019	6876.452757	9563.941616	4272.159477	211.41%
Deaths	Nepal	Both	Age-standardized	Aortic aneurysm	Rate	1990	1.174780241	1.943792181	0.61180611	
Deaths	Nepal	Both	Age-standardized	Aortic aneurysm	Rate	2019	1.716616086	2.320331355	1.10047985	46.12%
Deaths	Nepal	Both	All Ages	Aortic aneurysm	Number	1990	89.71039295	151.2922732	45.66541727	
Deaths	Nepal	Both	All Ages	Aortic aneurysm	Number	2019	325.7671013	446.3299015	206.1319442	263.13%
DALYs	Netherlands	Both	Age-standardized	Aortic aneurysm	Rate	1990	127.4738462	134.1492532	120.5175083	
DALYs	Netherlands	Both	Age-standardized	Aortic aneurysm	Rate	2019	69.74567496	76.39199378	62.83137304	-45.29%
DALYs	Netherlands	Both	All Ages	Aortic aneurysm	Number	1990	25877.28205	27258.00938	24438.84764	
DALYs	Netherlands	Both	All Ages	Aortic aneurysm	Number	2019	24246.23158	26680.19598	21593.98583	-6.30%
Deaths	Netherlands	Both	Age-standardized	Aortic aneurysm	Rate	1990	7.103185843	7.506473568	6.624780377	
Deaths	Netherlands	Both	Age-standardized	Aortic aneurysm	Rate	2019	4.339721851	4.81458507	3.827408038	-38.90%
Deaths	Netherlands	Both	All Ages	Aortic aneurysm	Number	1990	1475.318065	1563.623289	1375.449805	
Deaths	Netherlands	Both	All Ages	Aortic aneurysm	Number	2019	1612.304598	1792.450744	1413.543034	9.29%
DALYs	New Zealand	Both	Age-standardized	Aortic aneurysm	Rate	1990	173.8929271	183.124264	164.2795141	

DALYs	New Zealand	Both	Age-standardized	Aortic aneurysm	Rate	2019	87.81224806	96.25892933	79.62226935	-49.50%
DALYs	New Zealand	Both	All Ages	Aortic aneurysm	Number	1990	6976.307648	7357.083218	6592.676703	
DALYs	New Zealand	Both	All Ages	Aortic aneurysm	Number	2019	6915.158911	7617.343511	6230.722435	-0.88%
Deaths	New Zealand	Both	Age-standardized	Aortic aneurysm	Rate	1990	9.812421891	10.42877374	9.136745088	
Deaths	New Zealand	Both	Age-standardized	Aortic aneurysm	Rate	2019	5.316638948	5.919224382	4.698963187	-45.82%
Deaths	New Zealand	Both	All Ages	Aortic aneurysm	Number	1990	392.5240574	416.9550499	366.6316242	
Deaths	New Zealand	Both	All Ages	Aortic aneurysm	Number	2019	447.7678689	499.5969821	392.621592	14.07%
DALYs	Nicaragua	Both	Age-standardized	Aortic aneurysm	Rate	1990	16.73109245	19.38013064	14.14801023	
DALYs	Nicaragua	Both	Age-standardized	Aortic aneurysm	Rate	2019	19.6793799	23.7974331	15.89926678	17.62%
DALYs	Nicaragua	Both	All Ages	Aortic aneurysm	Number	1990	278.601271	321.2603175	238.1086998	
DALYs	Nicaragua	Both	All Ages	Aortic aneurysm	Number	2019	860.3664142	1057.109917	686.7670185	208.82%
Deaths	Nicaragua	Both	Age-standardized	Aortic aneurysm	Rate	1990	0.808480026	0.939835122	0.680974897	
Deaths	Nicaragua	Both	Age-standardized	Aortic aneurysm	Rate	2019	1.098829678	1.296919409	0.905022518	35.91%
Deaths	Nicaragua	Both	All Ages	Aortic aneurysm	Number	1990	11.39267142	13.1448198	9.670940443	
Deaths	Nicaragua	Both	All Ages	Aortic aneurysm	Number	2019	41.27593872	49.29600862	33.91915899	262.30%
DALYs	Niger	Both	Age-standardized	Aortic aneurysm	Rate	1990	35.53407919	51.4235623	18.42959291	
DALYs	Niger	Both	Age-standardized	Aortic aneurysm	Rate	2019	25.27193691	38.53771564	13.36686251	-28.88%
DALYs	Niger	Both	All Ages	Aortic aneurysm	Number	1990	1019.326729	1507.296537	522.4675709	
DALYs	Niger	Both	All Ages	Aortic aneurysm	Number	2019	2041.009609	3177.313113	1061.024623	100.23%
Deaths	Niger	Both	Age-standardized	Aortic aneurysm	Rate	1990	1.744107313	2.491016045	0.913039481	
Deaths	Niger	Both	Age-standardized	Aortic aneurysm	Rate	2019	1.275720513	1.881378901	0.688578826	-26.86%
Deaths	Niger	Both	All Ages	Aortic aneurysm	Number	1990	39.72492616	57.47103544	20.6784277	
Deaths	Niger	Both	All Ages	Aortic aneurysm	Number	2019	81.47961897	123.7235633	43.38871078	105.11%
DALYs	Nigeria	Both	Age-standardized	Aortic aneurysm	Rate	1990	45.70172996	67.86654301	30.67917099	
DALYs	Nigeria	Both	Age-standardized	Aortic aneurysm	Rate	2019	30.367566	40.62467486	22.64847222	-33.55%
DALYs	Nigeria	Both	All Ages	Aortic aneurysm	Number	1990	20237.91379	30422.01525	13291.19063	
DALYs	Nigeria	Both	All Ages	Aortic aneurysm	Number	2019	26789.76558	36733.02856	19367.99978	32.37%
Deaths	Nigeria	Both	Age-standardized	Aortic aneurysm	Rate	1990	2.310391513	3.382660586	1.620255676	
Deaths	Nigeria	Both	Age-standardized	Aortic aneurysm	Rate	2019	1.600460555	2.087382165	1.222336463	-30.73%
Deaths	Nigeria	Both	All Ages	Aortic aneurysm	Number	1990	874.743368	1290.33268	598.6480185	
Deaths	Nigeria	Both	All Ages	Aortic aneurysm	Number	2019	1148.619684	1520.389169	868.3158886	31.31%
DALYs	Niue	Both	Age-standardized	Aortic aneurysm	Rate	1990	85.96570234	114.3141373	64.48026329	
DALYs	Niue	Both	Age-standardized	Aortic aneurysm	Rate	2019	67.61830376	88.12569704	50.75005891	-21.34%
DALYs	Niue	Both	All Ages	Aortic aneurysm	Number	1990	1.858145973	2.451403419	1.404460244	
DALYs	Niue	Both	All Ages	Aortic aneurysm	Number	2019	1.426352984	1.840875692	1.076306457	-23.24%
Deaths	Niue	Both	Age-standardized	Aortic aneurysm	Rate	1990	4.145539197	5.398716139	3.200379623	
Deaths	Niue	Both	Age-standardized	Aortic aneurysm	Rate	2019	3.272654854	4.124398249	2.514347574	-21.06%
Deaths	Niue	Both	All Ages	Aortic aneurysm	Number	1990	0.096618444	0.125339371	0.075090948	
Deaths	Niue	Both	All Ages	Aortic aneurysm	Number	2019	0.069404536	0.087324976	0.053198262	-28.17%
DALYs	North Macedonia	Both	Age-standardized	Aortic aneurysm	Rate	1990	30.83823652	34.41528004	27.91700556	
DALYs	North Macedonia	Both	Age-standardized	Aortic aneurysm	Rate	2019	49.55886972	62.57786602	38.22006059	60.71%
DALYs	North Macedonia	Both	All Ages	Aortic aneurysm	Number	1990	573.43442	641.6465416	519.0036195	
DALYs	North Macedonia	Both	All Ages	Aortic aneurysm	Number	2019	1543.256181	1969.553965	1169.57718	169.13%
Deaths	North Macedonia	Both	Age-standardized	Aortic aneurysm	Rate	1990	1.531753576	1.700395103	1.386500954	
Deaths	North Macedonia	Both	Age-standardized	Aortic aneurysm	Rate	2019	2.476256845	3.061837371	1.968256505	61.66%
Deaths	North Macedonia	Both	All Ages	Aortic aneurysm	Number	1990	25.07706537	27.88134838	22.68293344	
Deaths	North Macedonia	Both	All Ages	Aortic aneurysm	Number	2019	71.70681461	89.74714473	55.61778124	185.95%
DALYs	Northern Mariana Islands	Both	Age-standardized	Aortic aneurysm	Rate	1990	146.6399764	182.4843899	121.7303356	
DALYs	Northern Mariana Islands	Both	Age-standardized	Aortic aneurysm	Rate	2019	45.03940415	52.7577662	37.52446641	-69.29%
DALYs	Northern Mariana Islands	Both	All Ages	Aortic aneurysm	Number	1990	30.21314996	38.93250594	23.52998218	
DALYs	Northern Mariana Islands	Both	All Ages	Aortic aneurysm	Number	2019	22.86771929	27.04690497	18.75959289	-24.31%
Deaths	Northern Mariana Islands	Both	Age-standardized	Aortic aneurysm	Rate	1990	7.67273003	9.361866231	6.435099552	
Deaths	Northern Mariana Islands	Both	Age-standardized	Aortic aneurysm	Rate	2019	2.28690044	2.627268198	1.960343973	-70.19%
Deaths	Northern Mariana Islands	Both	All Ages	Aortic aneurysm	Number	1990	0.986860291	1.249023457	0.800907389	
Deaths	Northern Mariana Islands	Both	All Ages	Aortic aneurysm	Number	2019	0.929188028	1.075898312	0.77988668	-5.84%
DALYs	Norway	Both	Age-standardized	Aortic aneurysm	Rate	1990	135.7280105	140.8861254	129.8906936	
DALYs	Norway	Both	Age-standardized	Aortic aneurysm	Rate	2019	83.85629543	89.94758318	77.34035225	-38.22%
DALYs	Norway	Both	All Ages	Aortic aneurysm	Number	1990	9446.308802	9833.812462	8995.832647	
DALYs	Norway	Both	All Ages	Aortic aneurysm	Number	2019	8201.952282	8808.227497	7511.932764	-13.17%
Deaths	Norway	Both	Age-standardized	Aortic aneurysm	Rate	1990	7.340886442	7.650865925	6.903797856	
Deaths	Norway	Both	Age-standardized	Aortic aneurysm	Rate	2019	5.209923407	5.603725952	4.693416602	-29.03%
Deaths	Norway	Both	All Ages	Aortic aneurysm	Number	1990	548.1448102	572.2425693	514.1797662	
Deaths	Norway	Both	All Ages	Aortic aneurysm	Number	2019	551.5768156	593.9836232	491.28321	0.63%
DALYs	Oman	Both	Age-standardized	Aortic aneurysm	Rate	1990	41.64638617	62.44408802	25.86871757	
DALYs	Oman	Both	Age-standardized	Aortic aneurysm	Rate	2019	41.02042745	55.16296095	28.825126	-1.50%
DALYs	Oman	Both	All Ages	Aortic aneurysm	Number	1990	323.9435482	501.9402895	199.4542897	
DALYs	Oman	Both	All Ages	Aortic aneurysm	Number	2019	836.7660881	1243.11748	556.3212085	158.31%
Deaths	Oman	Both	Age-standardized	Aortic aneurysm	Rate	1990	1.961447181	2.896888765	1.260921582	
Deaths	Oman	Both	Age-standardized	Aortic aneurysm	Rate	2019	2.246440656	3.032087674	1.548888823	14.53%
Deaths	Oman	Both	All Ages	Aortic aneurysm	Number	1990	11.2346751	16.87452934	6.955181262	
Deaths	Oman	Both	All Ages	Aortic aneurysm	Number	2019	28.68481881	39.0531773	19.9003556	155.32%
DALYs	Pakistan	Both	Age-standardized	Aortic aneurysm	Rate	1990	30.83430233	45.03197847	19.11611134	
DALYs	Pakistan	Both	Age-standardized	Aortic aneurysm	Rate	2019	40.10510373	52.87764947	29.55387507	30.07%
DALYs	Pakistan	Both	All Ages	Aortic aneurysm	Number	1990	17713.36498	26149.87161	10906.81256	
DALYs	Pakistan	Both	All Ages	Aortic aneurysm	Number	2019	45852.1879	61911.05709	33008.30397	158.86%
Deaths	Pakistan	Both	Age-standardized	Aortic aneurysm	Rate	1990	1.568639661	2.2406555	0.975228561	
Deaths	Pakistan	Both	Age-standardized	Aortic aneurysm	Rate	2019	2.01206489	2.589989246	1.519668959	28.27%
Deaths	Pakistan	Both	All Ages	Aortic aneurysm	Number	1990	804.333249	1161.719721	500.9717733	
Deaths	Pakistan	Both	All Ages	Aortic aneurysm	Number	2019	1834.670794	2404.513182	1354.221456	128.10%
DALYs	Palau	Both	Age-standardized	Aortic aneurysm	Rate	1990	64.84968391	88.76245642	39.32052118	
DALYs	Palau	Both	Age-standardized	Aortic aneurysm	Rate	2019	57.90855957	79.05011462	39.75639226	-10.70%
DALYs	Palau	Both	All Ages	Aortic aneurysm	Number	1990	7.011912011	9.777687986	4.06876287	
DALYs	Palau	Both	All Ages	Aortic aneurysm	Number	2019	12.67257028	17.72257447	8.477990905	80.73%
Deaths	Palau	Both	Age-standardized	Aortic aneurysm	Rate	1990	2.822722852	3.721154472	1.832772138	
Deaths	Palau	Both	Age-standardized	Aortic aneurysm	Rate	2019	2.470297722	3.278044273	1.808197308	-12.49%
Deaths	Palau	Both	All Ages	Aortic aneurysm	Number	1990	0.257350205	0.346563847	0.15958908	
Deaths	Palau	Both	All Ages	Aortic aneurysm	Number	2019	0.459013587	0.630296823	0.322170338	78.36%
DALYs	Palestine	Both	Age-standardized	Aortic aneurysm	Rate	1990	20.78325841	29.78653065	13.08306695	
DALYs	Palestine	Both	Age-standardized	Aortic aneurysm	Rate	2019	21.26426829	25.11794275	17.92781742	2.31%
DALYs	Palestine	Both	All Ages	Aortic aneurysm	Number	1990	192.782516	282.2649453	120.7727819	
DALYs	Palestine	Both	All Ages	Aortic aneurysm	Number	2019	541.008873	646.5895193	452.1722745	180.63%
Deaths	Palestine	Both	Age-standardized	Aortic aneurysm	Rate	1990	1.023167745	1.404731876	0.65547759	
Deaths	Palestine	Both	Age-standardized	Aortic aneurysm	Rate	2019	1.075083342	1.251473795	0.912216367	5.07%
Deaths	Palestine	Both	All Ages	Aortic aneurysm	Number	1990	8.111308458	11.34908239	5.14813307	
Deaths	Palestine	Both	All Ages	Aortic aneurysm	Number	2019	21.37485576	25.15178106	18.08006189	163.52%
DALYs	Panama	Both	Age-standardized	Aortic aneurysm	Rate	1990	45.36222425	50.37640417	40.74078516	
DALYs	Panama	Both	Age-standardized	Aortic aneurysm	Rate	2019	40.07414396	51.67444025	30.24463863	-11.66%
DALYs	Panama	Both	All Ages	Aortic aneurysm	Number	1990	717.1843136	792.5829483	646.8574909	
DALYs	Panama	Both	All Ages	Aortic aneurysm	Number	2019	1668.906005	2153.376398	1257.324807	132.70%
Deaths	Panama	Both	Age-standardized	Aortic aneurysm	Rate	1990	2.141395345	2.409111896	1.88719568	
Deaths	Panama	Both	Age-standardized	Aortic aneurysm	Rate	2019	1.862006048	2.370285687	1.425071196	-13.05%

Deaths	Panama	Both	All Ages	Aortic aneurysm	Number	1990	31.11378695	34.84812201	27.65133358	
Deaths	Panama	Both	All Ages	Aortic aneurysm	Number	2019	77.74398955	98.82117913	59.52779605	149.87%
DALYs	Papua New Guinea	Both	Age-standardized	Aortic aneurysm	Rate	1990	54.86725239	100.0797662	30.05916294	
DALYs	Papua New Guinea	Both	Age-standardized	Aortic aneurysm	Rate	2019	52.93110212	87.12129029	31.39144781	-3.53%
DALYs	Papua New Guinea	Both	All Ages	Aortic aneurysm	Number	1990	1038.548247	1958.735855	546.1544754	
DALYs	Papua New Guinea	Both	All Ages	Aortic aneurysm	Number	2019	2654.920154	4575.149964	1498.056643	155.64%
Deaths	Papua New Guinea	Both	Age-standardized	Aortic aneurysm	Rate	1990	2.78718437	4.812299668	1.579275388	
Deaths	Papua New Guinea	Both	Age-standardized	Aortic aneurysm	Rate	2019	2.617684462	4.16724603	1.620586793	-6.08%
Deaths	Papua New Guinea	Both	All Ages	Aortic aneurysm	Number	1990	38.62854357	71.49653079	21.17630555	
Deaths	Papua New Guinea	Both	All Ages	Aortic aneurysm	Number	2019	97.27356074	160.4245376	57.73028107	151.82%
DALYs	Paraguay	Both	Age-standardized	Aortic aneurysm	Rate	1990	43.74113644	50.73415847	37.39298825	
DALYs	Paraguay	Both	Age-standardized	Aortic aneurysm	Rate	2019	58.4888524	76.53810161	43.52486709	33.72%
DALYs	Paraguay	Both	All Ages	Aortic aneurysm	Number	1990	1017.107457	1181.157148	868.8894387	
DALYs	Paraguay	Both	All Ages	Aortic aneurysm	Number	2019	3343.289545	4409.146701	2476.848104	228.71%
Deaths	Paraguay	Both	Age-standardized	Aortic aneurysm	Rate	1990	1.974372361	2.279116746	1.68585834	
Deaths	Paraguay	Both	Age-standardized	Aortic aneurysm	Rate	2019	2.65555215	3.42318261	2.028679527	34.50%
Deaths	Paraguay	Both	All Ages	Aortic aneurysm	Number	1990	42.27571649	48.91376088	36.29088281	
Deaths	Paraguay	Both	All Ages	Aortic aneurysm	Number	2019	143.1288237	184.3841622	108.6811326	238.56%
DALYs	Peru	Both	Age-standardized	Aortic aneurysm	Rate	1990	26.63348151	31.73688644	21.18694005	
DALYs	Peru	Both	Age-standardized	Aortic aneurysm	Rate	2019	20.27058811	26.99356304	14.8878723	-23.89%
DALYs	Peru	Both	All Ages	Aortic aneurysm	Number	1990	3392.936142	4068.318213	2628.093226	
DALYs	Peru	Both	All Ages	Aortic aneurysm	Number	2019	6589.036244	8762.124876	4837.119728	94.20%
Deaths	Peru	Both	Age-standardized	Aortic aneurysm	Rate	1990	1.323104414	1.553872392	1.091285956	
Deaths	Peru	Both	Age-standardized	Aortic aneurysm	Rate	2019	1.036882456	1.349265438	0.770297696	-21.63%
Deaths	Peru	Both	All Ages	Aortic aneurysm	Number	1990	147.5680051	173.9774712	121.2318848	
Deaths	Peru	Both	All Ages	Aortic aneurysm	Number	2019	335.1483469	436.6907919	248.4261048	127.11%
DALYs	Philippines	Both	Age-standardized	Aortic aneurysm	Rate	1990	17.16166448	19.8018106	15.25115135	
DALYs	Philippines	Both	Age-standardized	Aortic aneurysm	Rate	2019	33.90597704	41.0621587	26.97690883	97.57%
DALYs	Philippines	Both	All Ages	Aortic aneurysm	Number	1990	4790.789221	5945.041064	4231.523536	
DALYs	Philippines	Both	All Ages	Aortic aneurysm	Number	2019	27040.44048	32935.36048	21055.33984	464.43%
Deaths	Philippines	Both	Age-standardized	Aortic aneurysm	Rate	1990	1.032014428	1.15166067	0.914529467	
Deaths	Philippines	Both	Age-standardized	Aortic aneurysm	Rate	2019	1.685757536	2.014050958	1.380311968	63.35%
Deaths	Philippines	Both	All Ages	Aortic aneurysm	Number	1990	223.9883929	257.5546362	198.986499	
Deaths	Philippines	Both	All Ages	Aortic aneurysm	Number	2019	1118.711821	1348.946822	903.0397206	399.45%
DALYs	Poland	Both	Age-standardized	Aortic aneurysm	Rate	1990	48.46731716	50.09712919	46.73117519	
DALYs	Poland	Both	Age-standardized	Aortic aneurysm	Rate	2019	70.48758706	84.18757708	57.74826198	45.43%
DALYs	Poland	Both	All Ages	Aortic aneurysm	Number	1990	21066.50974	21784.47397	20295.01813	
DALYs	Poland	Both	All Ages	Aortic aneurysm	Number	2019	46220.82681	55075.14775	38008.38861	119.40%
Deaths	Poland	Both	Age-standardized	Aortic aneurysm	Rate	1990	2.021389498	2.096955601	1.932643609	
Deaths	Poland	Both	Age-standardized	Aortic aneurysm	Rate	2019	3.232282047	3.805598851	2.693013219	59.90%
Deaths	Poland	Both	All Ages	Aortic aneurysm	Number	1990	869.548951	902.6786061	833.394413	
Deaths	Poland	Both	All Ages	Aortic aneurysm	Number	2019	2282.595242	2685.18105	1895.330705	162.50%
DALYs	Portugal	Both	Age-standardized	Aortic aneurysm	Rate	1990	33.37999158	35.28273027	31.55821855	
DALYs	Portugal	Both	Age-standardized	Aortic aneurysm	Rate	2019	36.88652133	40.62565917	33.3312146	10.50%
DALYs	Portugal	Both	All Ages	Aortic aneurysm	Number	1990	4418.024477	4680.313301	4167.91583	
DALYs	Portugal	Both	All Ages	Aortic aneurysm	Number	2019	7704.032951	8534.585038	6932.689985	74.38%
Deaths	Portugal	Both	Age-standardized	Aortic aneurysm	Rate	1990	1.462297232	1.551210414	1.372074357	
Deaths	Portugal	Both	Age-standardized	Aortic aneurysm	Rate	2019	1.720197724	1.905010895	1.542480671	17.64%
Deaths	Portugal	Both	All Ages	Aortic aneurysm	Number	1990	198.0248527	210.9629721	185.4065992	
Deaths	Portugal	Both	All Ages	Aortic aneurysm	Number	2019	427.9237995	475.8935934	381.128003	116.10%
DALYs	Puerto Rico	Both	Age-standardized	Aortic aneurysm	Rate	1990	34.19966433	37.61564777	31.05634145	
DALYs	Puerto Rico	Both	Age-standardized	Aortic aneurysm	Rate	2019	20.91555118	26.81702532	16.03448875	-38.84%
DALYs	Puerto Rico	Both	All Ages	Aortic aneurysm	Number	1990	1246.002186	1376.9837	1128.601944	
DALYs	Puerto Rico	Both	All Ages	Aortic aneurysm	Number	2019	1431.980162	1825.397078	1105.005205	14.93%
Deaths	Puerto Rico	Both	Age-standardized	Aortic aneurysm	Rate	1990	1.878168907	2.080790367	1.669709786	
Deaths	Puerto Rico	Both	Age-standardized	Aortic aneurysm	Rate	2019	1.133987564	1.41932768	0.886331585	-39.62%
Deaths	Puerto Rico	Both	All Ages	Aortic aneurysm	Number	1990	66.69412421	73.97370183	59.28213463	
Deaths	Puerto Rico	Both	All Ages	Aortic aneurysm	Number	2019	90.18806649	112.4871105	70.03769241	35.23%
DALYs	Qatar	Both	Age-standardized	Aortic aneurysm	Rate	1990	38.38261704	51.593768	28.50845489	
DALYs	Qatar	Both	Age-standardized	Aortic aneurysm	Rate	2019	34.57712262	47.68766464	24.07326942	-9.91%
DALYs	Qatar	Both	All Ages	Aortic aneurysm	Number	1990	55.32593105	74.90732211	38.69686966	
DALYs	Qatar	Both	All Ages	Aortic aneurysm	Number	2019	325.2971922	469.8538946	212.4235165	487.97%
Deaths	Qatar	Both	Age-standardized	Aortic aneurysm	Rate	1990	2.014334758	2.5960185	1.540409526	
Deaths	Qatar	Both	Age-standardized	Aortic aneurysm	Rate	2019	2.326855129	3.136213447	1.646651956	15.51%
Deaths	Qatar	Both	All Ages	Aortic aneurysm	Number	1990	1.711645192	2.29637906	1.231293351	
Deaths	Qatar	Both	All Ages	Aortic aneurysm	Number	2019	10.04881344	14.27690039	6.626305984	487.09%
DALYs	Republic of Korea	Both	Age-standardized	Aortic aneurysm	Rate	1990	35.3999854	43.3070097	29.24182859	
DALYs	Republic of Korea	Both	Age-standardized	Aortic aneurysm	Rate	2019	27.8525363	31.63285711	24.31462258	-21.32%
DALYs	Republic of Korea	Both	All Ages	Aortic aneurysm	Number	1990	10417.19051	12647.4716	8446.007032	
DALYs	Republic of Korea	Both	All Ages	Aortic aneurysm	Number	2019	24014.18591	27308.06067	20926.96279	130.52%
Deaths	Republic of Korea	Both	Age-standardized	Aortic aneurysm	Rate	1990	1.922593778	2.372936131	1.600880532	
Deaths	Republic of Korea	Both	Age-standardized	Aortic aneurysm	Rate	2019	1.722666298	1.99590075	1.469847418	-10.40%
Deaths	Republic of Korea	Both	All Ages	Aortic aneurysm	Number	1990	449.0988129	549.9789669	370.5185434	
Deaths	Republic of Korea	Both	All Ages	Aortic aneurysm	Number	2019	1461.085832	1691.326056	1251.43533	225.34%
DALYs	Republic of Moldova	Both	Age-standardized	Aortic aneurysm	Rate	1990	23.1510386	25.98837516	20.29222608	
DALYs	Republic of Moldova	Both	Age-standardized	Aortic aneurysm	Rate	2019	35.97957301	42.31682773	30.76541581	55.41%
DALYs	Republic of Moldova	Both	All Ages	Aortic aneurysm	Number	1990	1027.200636	1158.873807	895.7185996	
DALYs	Republic of Moldova	Both	All Ages	Aortic aneurysm	Number	2019	1952.550903	2287.809483	1668.610508	90.08%
Deaths	Republic of Moldova	Both	Age-standardized	Aortic aneurysm	Rate	1990	0.948095071	1.070334908	0.831259578	
Deaths	Republic of Moldova	Both	Age-standardized	Aortic aneurysm	Rate	2019	1.422126951	1.643645291	1.227044127	50.00%
Deaths	Republic of Moldova	Both	All Ages	Aortic aneurysm	Number	1990	38.19007683	43.24110366	33.27839899	
Deaths	Republic of Moldova	Both	All Ages	Aortic aneurysm	Number	2019	80.68942685	93.47603178	69.58466824	111.28%
DALYs	Romania	Both	Age-standardized	Aortic aneurysm	Rate	1990	38.14287947	42.46245816	34.57052722	
DALYs	Romania	Both	Age-standardized	Aortic aneurysm	Rate	2019	54.20915502	65.73922855	44.10507528	42.12%
DALYs	Romania	Both	All Ages	Aortic aneurysm	Number	1990	10330.70606	11509.0893	9349.483934	
DALYs	Romania	Both	All Ages	Aortic aneurysm	Number	2019	17756.53678	21561.58486	14415.25441	71.88%
Deaths	Romania	Both	Age-standardized	Aortic aneurysm	Rate	1990	1.626570791	1.816504505	1.465333059	
Deaths	Romania	Both	Age-standardized	Aortic aneurysm	Rate	2019	2.400832693	2.892311797	1.974018221	47.60%
Deaths	Romania	Both	All Ages	Aortic aneurysm	Number	1990	420.4965251	471.404503	377.9541116	
Deaths	Romania	Both	All Ages	Aortic aneurysm	Number	2019	889.2860064	1072.309262	729.2063779	111.48%
DALYs	Russian Federation	Both	Age-standardized	Aortic aneurysm	Rate	1990	62.30118976	67.47377454	56.69825161	
DALYs	Russian Federation	Both	Age-standardized	Aortic aneurysm	Rate	2019	90.184239	104.7950623	77.2295111	44.76%
DALYs	Russian Federation	Both	All Ages	Aortic aneurysm	Number	1990	112019.279	121166.8455	102021.6705	
DALYs	Russian Federation	Both	All Ages	Aortic aneurysm	Number	2019	201659.1842	233723.0124	173107.1698	80.02%
Deaths	Russian Federation	Both	Age-standardized	Aortic aneurysm	Rate	1990	2.612742613	2.779260455	2.439821318	
Deaths	Russian Federation	Both	Age-standardized	Aortic aneurysm	Rate	2019	3.918047746	4.504508339	3.398131593	49.96%
Deaths	Russian Federation	Both	All Ages	Aortic aneurysm	Number	1990	4560.886853	4857.602778	4250.33889	
Deaths	Russian Federation	Both	All Ages	Aortic aneurysm	Number	2019	9129.909723	10483.17768	7920.458206	100.18%
DALYs	Rwanda	Both	Age-standardized	Aortic aneurysm	Rate	1990	82.00463833	123.9131794	47.03017911	
DALYs	Rwanda	Both	Age-standardized	Aortic aneurysm	Rate	2019	43.68180443	59.23381571	30.33628046	-46.73%
DALYs	Rwanda	Both	All Ages	Aortic aneurysm	Number	1990	2534.277436	3930.324524	1406.010695	

DALYs	Rwanda	Both	All Ages	Aortic aneurysm	Number	2019	2758.681211	3766.81582	1897.176535	8.85%
Deaths	Rwanda	Both	Age-standardized	Aortic aneurysm	Rate	1990	3.848578191	5.600765324	2.285740698	
Deaths	Rwanda	Both	Age-standardized	Aortic aneurysm	Rate	2019	2.219564838	2.930698077	1.548577506	-42.33%
Deaths	Rwanda	Both	All Ages	Aortic aneurysm	Number	1990	96.49704663	144.3655969	55.21788228	
Deaths	Rwanda	Both	All Ages	Aortic aneurysm	Number	2019	109.7496558	147.0205604	75.97440724	13.73%
DALYs	Saint Kitts and Nevis	Both	Age-standardized	Aortic aneurysm	Rate	1990	64.57292157	73.93427549	55.27442877	
DALYs	Saint Kitts and Nevis	Both	Age-standardized	Aortic aneurysm	Rate	2019	60.01268396	71.10073225	49.49100911	-7.06%
DALYs	Saint Kitts and Nevis	Both	All Ages	Aortic aneurysm	Number	1990	24.80776375	28.66123398	21.12397833	
DALYs	Saint Kitts and Nevis	Both	All Ages	Aortic aneurysm	Number	2019	37.69748659	45.06588059	30.44601199	51.96%
Deaths	Saint Kitts and Nevis	Both	Age-standardized	Aortic aneurysm	Rate	1990	3.482710114	3.964830999	3.03811468	
Deaths	Saint Kitts and Nevis	Both	Age-standardized	Aortic aneurysm	Rate	2019	3.30848239	3.826559141	2.845371286	-5.00%
Deaths	Saint Kitts and Nevis	Both	All Ages	Aortic aneurysm	Number	1990	1.314689321	1.521398518	1.123251805	
Deaths	Saint Kitts and Nevis	Both	All Ages	Aortic aneurysm	Number	2019	1.800105082	2.099836868	1.526443541	36.92%
DALYs	Saint Lucia	Both	Age-standardized	Aortic aneurysm	Rate	1990	120.0419514	135.1974863	106.5507333	
DALYs	Saint Lucia	Both	Age-standardized	Aortic aneurysm	Rate	2019	108.8623623	129.3879488	89.22270569	-9.31%
DALYs	Saint Lucia	Both	All Ages	Aortic aneurysm	Number	1990	104.1277008	117.3948776	92.4338248	
DALYs	Saint Lucia	Both	All Ages	Aortic aneurysm	Number	2019	229.9587942	273.7545202	188.4630999	120.84%
Deaths	Saint Lucia	Both	Age-standardized	Aortic aneurysm	Rate	1990	6.904365153	7.766753166	6.0999769	
Deaths	Saint Lucia	Both	Age-standardized	Aortic aneurysm	Rate	2019	6.244021809	7.407848144	5.179247973	-9.56%
Deaths	Saint Lucia	Both	All Ages	Aortic aneurysm	Number	1990	5.470384842	6.213261657	4.809622469	
Deaths	Saint Lucia	Both	All Ages	Aortic aneurysm	Number	2019	12.71974806	15.13012443	10.538845	132.52%
DALYs	Saint Vincent and the Grenadines	Both	Age-standardized	Aortic aneurysm	Rate	1990	52.22918807	59.08372141	46.42053428	
DALYs	Saint Vincent and the Grenadines	Both	Age-standardized	Aortic aneurysm	Rate	2019	59.18292492	68.34624361	50.19633544	13.31%
DALYs	Saint Vincent and the Grenadines	Both	All Ages	Aortic aneurysm	Number	1990	38.4376893	43.59836797	34.07144547	
DALYs	Saint Vincent and the Grenadines	Both	All Ages	Aortic aneurysm	Number	2019	78.616039	90.89130835	66.61103726	104.53%
Deaths	Saint Vincent and the Grenadines	Both	Age-standardized	Aortic aneurysm	Rate	1990	2.830921924	3.207774324	2.491311433	
Deaths	Saint Vincent and the Grenadines	Both	Age-standardized	Aortic aneurysm	Rate	2019	3.259280853	3.760215712	2.798281679	15.13%
Deaths	Saint Vincent and the Grenadines	Both	All Ages	Aortic aneurysm	Number	1990	1.98668146	2.260441896	1.741762487	
Deaths	Saint Vincent and the Grenadines	Both	All Ages	Aortic aneurysm	Number	2019	4.126329283	4.769694386	3.528306099	107.70%
DALYs	Samoa	Both	Age-standardized	Aortic aneurysm	Rate	1990	80.16704847	110.0485575	60.40091579	
DALYs	Samoa	Both	Age-standardized	Aortic aneurysm	Rate	2019	60.74086383	80.10589074	43.21304404	-24.23%
DALYs	Samoa	Both	All Ages	Aortic aneurysm	Number	1990	70.46198596	98.85165607	52.01844748	
DALYs	Samoa	Both	All Ages	Aortic aneurysm	Number	2019	90.15794624	119.9225034	62.37057491	27.95%
Deaths	Samoa	Both	Age-standardized	Aortic aneurysm	Rate	1990	4.063469723	5.414191621	3.197371934	
Deaths	Samoa	Both	Age-standardized	Aortic aneurysm	Rate	2019	3.002740927	3.855594147	2.257931827	-26.10%
Deaths	Samoa	Both	All Ages	Aortic aneurysm	Number	1990	3.039336704	4.077009402	2.323953443	
Deaths	Samoa	Both	All Ages	Aortic aneurysm	Number	2019	3.925269119	5.073303142	2.896874663	29.15%
DALYs	San Marino	Both	Age-standardized	Aortic aneurysm	Rate	1990	53.01783612	63.18119536	43.8305697	
DALYs	San Marino	Both	Age-standardized	Aortic aneurysm	Rate	2019	47.39893962	67.9657339	30.13815908	-10.60%
DALYs	San Marino	Both	All Ages	Aortic aneurysm	Number	1990	17.61571917	21.00817634	14.50667253	
DALYs	San Marino	Both	All Ages	Aortic aneurysm	Number	2019	29.73342227	41.50134981	19.47005544	68.79%
Deaths	San Marino	Both	Age-standardized	Aortic aneurysm	Rate	1990	2.893310903	3.41845658	2.402871432	
Deaths	San Marino	Both	Age-standardized	Aortic aneurysm	Rate	2019	2.672117544	3.692670601	1.770378002	-7.64%
Deaths	San Marino	Both	All Ages	Aortic aneurysm	Number	1990	0.976704868	1.150786673	0.805946915	
Deaths	San Marino	Both	All Ages	Aortic aneurysm	Number	2019	1.922302677	2.625767372	1.291946295	96.82%
DALYs	Sao Tome and Principe	Both	Age-standardized	Aortic aneurysm	Rate	1990	32.65544504	42.66167166	22.91287824	
DALYs	Sao Tome and Principe	Both	Age-standardized	Aortic aneurysm	Rate	2019	33.36527227	41.81982687	25.59851291	2.17%
DALYs	Sao Tome and Principe	Both	All Ages	Aortic aneurysm	Number	1990	21.1296113	27.79356912	14.63982399	
DALYs	Sao Tome and Principe	Both	All Ages	Aortic aneurysm	Number	2019	36.5295875	47.31131673	27.46390436	72.88%
Deaths	Sao Tome and Principe	Both	Age-standardized	Aortic aneurysm	Rate	1990	1.648795856	2.100230992	1.167672933	
Deaths	Sao Tome and Principe	Both	Age-standardized	Aortic aneurysm	Rate	2019	1.67686162	2.062700006	1.328478375	1.70%
Deaths	Sao Tome and Principe	Both	All Ages	Aortic aneurysm	Number	1990	0.946488175	1.219558769	0.663594536	
Deaths	Sao Tome and Principe	Both	All Ages	Aortic aneurysm	Number	2019	1.524641409	1.888978229	1.186038488	61.08%
DALYs	Saudi Arabia	Both	Age-standardized	Aortic aneurysm	Rate	1990	17.75024946	26.11597813	10.74986825	
DALYs	Saudi Arabia	Both	Age-standardized	Aortic aneurysm	Rate	2019	19.66961584	25.40321421	15.00253416	10.81%
DALYs	Saudi Arabia	Both	All Ages	Aortic aneurysm	Number	1990	1166.337083	1771.470406	700.5032996	
DALYs	Saudi Arabia	Both	All Ages	Aortic aneurysm	Number	2019	4137.999861	5621.043911	3001.292487	254.79%
Deaths	Saudi Arabia	Both	Age-standardized	Aortic aneurysm	Rate	1990	0.877553741	1.2566535	0.535672493	
Deaths	Saudi Arabia	Both	Age-standardized	Aortic aneurysm	Rate	2019	1.001876557	1.260594984	0.784755843	14.17%
Deaths	Saudi Arabia	Both	All Ages	Aortic aneurysm	Number	1990	44.4180515	65.21010477	27.04518171	
Deaths	Saudi Arabia	Both	All Ages	Aortic aneurysm	Number	2019	139.612655	183.9302566	105.0019482	214.32%
DALYs	Senegal	Both	Age-standardized	Aortic aneurysm	Rate	1990	34.34698398	46.88877646	22.12834101	
DALYs	Senegal	Both	Age-standardized	Aortic aneurysm	Rate	2019	27.86056952	36.47214825	20.10976681	-18.88%
DALYs	Senegal	Both	All Ages	Aortic aneurysm	Number	1990	1132.736425	1557.460854	728.0522819	
DALYs	Senegal	Both	All Ages	Aortic aneurysm	Number	2019	2158.997481	2865.495257	1513.021387	90.60%
Deaths	Senegal	Both	Age-standardized	Aortic aneurysm	Rate	1990	1.722781967	2.323581458	1.109091297	
Deaths	Senegal	Both	Age-standardized	Aortic aneurysm	Rate	2019	1.408943451	1.823660272	1.044092092	-18.22%
Deaths	Senegal	Both	All Ages	Aortic aneurysm	Number	1990	48.44806585	65.84789171	31.10067378	
Deaths	Senegal	Both	All Ages	Aortic aneurysm	Number	2019	92.68712833	121.1925776	68.72119648	91.31%
DALYs	Serbia	Both	Age-standardized	Aortic aneurysm	Rate	1990	71.78783264	82.91586884	63.64846536	
DALYs	Serbia	Both	Age-standardized	Aortic aneurysm	Rate	2019	90.98117694	113.0178851	70.61062084	26.74%
DALYs	Serbia	Both	All Ages	Aortic aneurysm	Number	1990	8232.605698	9500.368767	7253.923949	
DALYs	Serbia	Both	All Ages	Aortic aneurysm	Number	2019	13844.5106	17188.29405	10737.57364	68.17%
Deaths	Serbia	Both	Age-standardized	Aortic aneurysm	Rate	1990	3.144106356	3.608478358	2.787825147	
Deaths	Serbia	Both	Age-standardized	Aortic aneurysm	Rate	2019	4.508721023	5.508405332	3.605520524	43.40%
Deaths	Serbia	Both	All Ages	Aortic aneurysm	Number	1990	339.184485	391.8156936	298.8882536	
Deaths	Serbia	Both	All Ages	Aortic aneurysm	Number	2019	708.7820235	872.6167676	559.6731761	108.97%
DALYs	Seychelles	Both	Age-standardized	Aortic aneurysm	Rate	1990	38.7344955	45.6523177	30.51029123	
DALYs	Seychelles	Both	Age-standardized	Aortic aneurysm	Rate	2019	38.73257989	50.46579679	29.78456702	0.00%
DALYs	Seychelles	Both	All Ages	Aortic aneurysm	Number	1990	22.4235113	26.39889544	17.79845245	
DALYs	Seychelles	Both	All Ages	Aortic aneurysm	Number	2019	41.89205792	54.74625895	32.04484755	86.82%
Deaths	Seychelles	Both	Age-standardized	Aortic aneurysm	Rate	1990	1.91962141	2.286473856	1.526303487	
Deaths	Seychelles	Both	Age-standardized	Aortic aneurysm	Rate	2019	1.920845197	2.50241097	1.484997951	0.06%
Deaths	Seychelles	Both	All Ages	Aortic aneurysm	Number	1990	1.083180985	1.28589305	0.863099825	
Deaths	Seychelles	Both	All Ages	Aortic aneurysm	Number	2019	1.888655427	2.456635335	1.455437463	74.36%
DALYs	Sierra Leone	Both	Age-standardized	Aortic aneurysm	Rate	1990	33.86275497	50.54105016	20.17702756	
DALYs	Sierra Leone	Both	Age-standardized	Aortic aneurysm	Rate	2019	27.49703819	40.10886809	18.04960291	-18.80%
DALYs	Sierra Leone	Both	All Ages	Aortic aneurysm	Number	1990	660.0620823	994.6761684	387.272994	
DALYs	Sierra Leone	Both	All Ages	Aortic aneurysm	Number	2019	1052.95603	1562.345273	684.4778994	59.52%
Deaths	Sierra Leone	Both	Age-standardized	Aortic aneurysm	Rate	1990	1.704915468	2.492905267	1.027249404	
Deaths	Sierra Leone	Both	Age-standardized	Aortic aneurysm	Rate	2019	1.367618322	1.940801927	0.932032729	-19.78%
Deaths	Sierra Leone	Both	All Ages	Aortic aneurysm	Number	1990	29.7938734	44.13524651	17.85955225	
Deaths	Sierra Leone	Both	All Ages	Aortic aneurysm	Number	2019	43.19849423	62.42660039	28.7210391	44.99%
DALYs	Singapore	Both	Age-standardized	Aortic aneurysm	Rate	1990	65.64290256	70.56848541	60.6028194	
DALYs	Singapore	Both	Age-standardized	Aortic aneurysm	Rate	2019	34.53077995	38.16379405	30.40977942	-47.40%
DALYs	Singapore	Both	All Ages	Aortic aneurysm	Number	1990	1485.177051	1600.134164	1377.969469	
DALYs	Singapore	Both	All Ages	Aortic aneurysm	Number	2019	2615.314454	2882.684995	2316.358946	76.09%
Deaths	Singapore	Both	Age-standardized	Aortic aneurysm	Rate	1990	3.406086264	3.682719006	3.109527983	
Deaths	Singapore	Both	Age-standardized	Aortic aneurysm	Rate	2019	2.025290946	2.271077014	1.717560197	-40.54%
Deaths	Singapore	Both	All Ages	Aortic aneurysm	Number	1990	65.63093338	70.64744129	60.37234708	
Deaths	Singapore	Both	All Ages	Aortic aneurysm	Number	2019	146.2174105	163.0591915	125.1801193	122.79%

DALYs	Slovakia	Both	Age-standardized	Aortic aneurysm	Rate	1990	41.83523239	46.9782182	37.41818534	
DALYs	Slovakia	Both	Age-standardized	Aortic aneurysm	Rate	2019	48.91359811	62.31316431	37.80291755	16.92%
DALYs	Slovakia	Both	All Ages	Aortic aneurysm	Number	1990	2448.9393	2757.416312	2181.507099	
DALYs	Slovakia	Both	All Ages	Aortic aneurysm	Number	2019	4210.601188	5317.160377	3254.012296	71.94%
Deaths	Slovakia	Both	Age-standardized	Aortic aneurysm	Rate	1990	1.718364095	1.923810063	1.550729659	
Deaths	Slovakia	Both	Age-standardized	Aortic aneurysm	Rate	2019	2.107415303	2.617341143	1.657991968	22.64%
Deaths	Slovakia	Both	All Ages	Aortic aneurysm	Number	1990	100.2156163	112.5423565	90.25397458	
Deaths	Slovakia	Both	All Ages	Aortic aneurysm	Number	2019	190.0025887	235.6584615	149.474148	89.59%
DALYs	Slovenia	Both	Age-standardized	Aortic aneurysm	Rate	1990	51.90213664	67.47539828	39.1664211	
DALYs	Slovenia	Both	Age-standardized	Aortic aneurysm	Rate	2019	55.45886993	73.26762547	42.46313585	6.85%
DALYs	Slovenia	Both	All Ages	Aortic aneurysm	Number	1990	1250.417257	1624.954309	941.7311831	
DALYs	Slovenia	Both	All Ages	Aortic aneurysm	Number	2019	2221.138009	2944.964419	1699.553841	77.63%
Deaths	Slovenia	Both	Age-standardized	Aortic aneurysm	Rate	1990	2.278173012	2.948950491	1.735561602	
Deaths	Slovenia	Both	Age-standardized	Aortic aneurysm	Rate	2019	2.691167962	3.505562849	2.070176989	18.13%
Deaths	Slovenia	Both	All Ages	Aortic aneurysm	Number	1990	54.79927657	70.79259543	41.77982882	
Deaths	Slovenia	Both	All Ages	Aortic aneurysm	Number	2019	121.5303171	157.5162771	93.65948736	121.77%
DALYs	Solomon Islands	Both	Age-standardized	Aortic aneurysm	Rate	1990	80.98881672	144.3751004	43.66708899	
DALYs	Solomon Islands	Both	Age-standardized	Aortic aneurysm	Rate	2019	72.71659258	118.1248844	42.48540646	-10.21%
DALYs	Solomon Islands	Both	All Ages	Aortic aneurysm	Number	1990	120.6672941	226.3706221	60.62297424	
DALYs	Solomon Islands	Both	All Ages	Aortic aneurysm	Number	2019	250.7422089	426.0527739	132.7730755	107.80%
Deaths	Solomon Islands	Both	Age-standardized	Aortic aneurysm	Rate	1990	3.587533902	5.987987617	2.089833282	
Deaths	Solomon Islands	Both	Age-standardized	Aortic aneurysm	Rate	2019	3.281431465	5.0299612	2.063900841	-8.53%
Deaths	Solomon Islands	Both	All Ages	Aortic aneurysm	Number	1990	4.080184105	7.315375967	2.18188118	
Deaths	Solomon Islands	Both	All Ages	Aortic aneurysm	Number	2019	8.40324228	13.74586285	4.838351982	105.95%
DALYs	Somalia	Both	Age-standardized	Aortic aneurysm	Rate	1990	65.71983475	109.0004553	27.52077939	
DALYs	Somalia	Both	Age-standardized	Aortic aneurysm	Rate	2019	38.92132165	63.78967542	15.95178923	-40.78%
DALYs	Somalia	Both	All Ages	Aortic aneurysm	Number	1990	1799.652017	3129.038992	746.3772604	
DALYs	Somalia	Both	All Ages	Aortic aneurysm	Number	2019	2884.827519	4941.844943	1160.514628	60.30%
Deaths	Somalia	Both	Age-standardized	Aortic aneurysm	Rate	1990	3.072774963	4.800330115	1.300147947	
Deaths	Somalia	Both	Age-standardized	Aortic aneurysm	Rate	2019	1.857170007	2.907436267	0.766122177	-39.56%
Deaths	Somalia	Both	All Ages	Aortic aneurysm	Number	1990	64.01739309	106.3932329	26.88112441	
Deaths	Somalia	Both	All Ages	Aortic aneurysm	Number	2019	101.0713601	167.347822	41.47147885	57.88%
DALYs	South Africa	Both	Age-standardized	Aortic aneurysm	Rate	1990	63.39401329	70.72247148	58.03707675	
DALYs	South Africa	Both	Age-standardized	Aortic aneurysm	Rate	2019	39.54544309	43.62251201	35.4203814	-37.62%
DALYs	South Africa	Both	All Ages	Aortic aneurysm	Number	1990	14226.20191	15763.49383	12996.08905	
DALYs	South Africa	Both	All Ages	Aortic aneurysm	Number	2019	17843.88423	19803.71081	15896.94969	25.43%
Deaths	South Africa	Both	Age-standardized	Aortic aneurysm	Rate	1990	3.244964658	3.629684292	2.931796066	
Deaths	South Africa	Both	Age-standardized	Aortic aneurysm	Rate	2019	2.091322457	2.292070285	1.871912484	-35.55%
Deaths	South Africa	Both	All Ages	Aortic aneurysm	Number	1990	614.4265886	685.2632238	559.8251795	
Deaths	South Africa	Both	All Ages	Aortic aneurysm	Number	2019	816.4778932	896.9296213	732.5443995	32.88%
DALYs	South Sudan	Both	Age-standardized	Aortic aneurysm	Rate	1990	61.13702173	89.79803161	36.16447215	
DALYs	South Sudan	Both	Age-standardized	Aortic aneurysm	Rate	2019	35.08574614	52.56373744	22.15796863	-42.61%
DALYs	South Sudan	Both	All Ages	Aortic aneurysm	Number	1990	1523.157489	2301.217638	888.2550446	
DALYs	South Sudan	Both	All Ages	Aortic aneurysm	Number	2019	1379.363384	2085.333253	861.3397295	-9.44%
Deaths	South Sudan	Both	Age-standardized	Aortic aneurysm	Rate	1990	2.996397624	4.310609352	1.809123361	
Deaths	South Sudan	Both	Age-standardized	Aortic aneurysm	Rate	2019	1.799168485	2.618669727	1.147575149	-39.96%
Deaths	South Sudan	Both	All Ages	Aortic aneurysm	Number	1990	63.18758595	92.32654948	37.65513871	
Deaths	South Sudan	Both	All Ages	Aortic aneurysm	Number	2019	56.5558076	84.62389846	35.66996003	-10.50%
DALYs	Spain	Both	Age-standardized	Aortic aneurysm	Rate	1990	44.85109948	47.3550606	42.39109442	
DALYs	Spain	Both	Age-standardized	Aortic aneurysm	Rate	2019	44.57571338	49.02701878	40.33776127	-0.61%
DALYs	Spain	Both	All Ages	Aortic aneurysm	Number	1990	23815.69124	25191.79836	22468.46058	
DALYs	Spain	Both	All Ages	Aortic aneurysm	Number	2019	39717.74735	43869.49495	35415.97486	66.77%
Deaths	Spain	Both	Age-standardized	Aortic aneurysm	Rate	1990	2.091568614	2.215926387	1.956713625	
Deaths	Spain	Both	Age-standardized	Aortic aneurysm	Rate	2019	2.295172516	2.548810454	2.041598201	9.73%
Deaths	Spain	Both	All Ages	Aortic aneurysm	Number	1990	1149.781289	1222.877401	1073.53922	
Deaths	Spain	Both	All Ages	Aortic aneurysm	Number	2019	2401.509847	2683.571592	2107.227642	108.87%
DALYs	Sri Lanka	Both	Age-standardized	Aortic aneurysm	Rate	1990	31.79901421	36.63860725	27.25484614	
DALYs	Sri Lanka	Both	Age-standardized	Aortic aneurysm	Rate	2019	29.1519274	38.32443113	21.92245308	-8.32%
DALYs	Sri Lanka	Both	All Ages	Aortic aneurysm	Number	1990	3378.509443	3942.903033	2885.86889	
DALYs	Sri Lanka	Both	All Ages	Aortic aneurysm	Number	2019	7038.981703	9257.393368	5258.683188	108.35%
Deaths	Sri Lanka	Both	Age-standardized	Aortic aneurysm	Rate	1990	1.79855535	2.059444063	1.520500084	
Deaths	Sri Lanka	Both	Age-standardized	Aortic aneurysm	Rate	2019	1.709833799	2.209083423	1.287480748	-4.93%
Deaths	Sri Lanka	Both	All Ages	Aortic aneurysm	Number	1990	153.6442399	176.4725943	131.0060823	
Deaths	Sri Lanka	Both	All Ages	Aortic aneurysm	Number	2019	373.9375737	488.8997258	280.0072149	143.38%
DALYs	Sudan	Both	Age-standardized	Aortic aneurysm	Rate	1990	22.1031295	36.7785526	11.20435122	
DALYs	Sudan	Both	Age-standardized	Aortic aneurysm	Rate	2019	22.60516338	36.55970695	14.10161956	2.27%
DALYs	Sudan	Both	All Ages	Aortic aneurysm	Number	1990	2213.903418	3750.346574	1098.416522	
DALYs	Sudan	Both	All Ages	Aortic aneurysm	Number	2019	4620.686502	7617.315681	2765.886602	108.71%
Deaths	Sudan	Both	Age-standardized	Aortic aneurysm	Rate	1990	1.019271867	1.649265845	0.531096309	
Deaths	Sudan	Both	Age-standardized	Aortic aneurysm	Rate	2019	1.113282118	1.792688974	0.727420901	9.22%
Deaths	Sudan	Both	All Ages	Aortic aneurysm	Number	1990	86.27971936	142.0947206	44.40629732	
Deaths	Sudan	Both	All Ages	Aortic aneurysm	Number	2019	184.616866	296.5866161	116.73771	113.97%
DALYs	Suriname	Both	Age-standardized	Aortic aneurysm	Rate	1990	47.7939385	52.96180465	42.24719768	
DALYs	Suriname	Both	Age-standardized	Aortic aneurysm	Rate	2019	45.33072254	54.82596438	37.34357259	-5.15%
DALYs	Suriname	Both	All Ages	Aortic aneurysm	Number	1990	124.5004253	138.2683457	109.2474647	
DALYs	Suriname	Both	All Ages	Aortic aneurysm	Number	2019	268.4145545	326.0247023	220.3186598	115.59%
Deaths	Suriname	Both	Age-standardized	Aortic aneurysm	Rate	1990	2.430749022	2.686340903	2.150278313	
Deaths	Suriname	Both	Age-standardized	Aortic aneurysm	Rate	2019	2.29928409	2.754801506	1.912314946	-5.41%
Deaths	Suriname	Both	All Ages	Aortic aneurysm	Number	1990	5.868949231	6.484834121	5.194390966	
Deaths	Suriname	Both	All Ages	Aortic aneurysm	Number	2019	12.9051627	15.46338836	10.70097062	119.89%
DALYs	Sweden	Both	Age-standardized	Aortic aneurysm	Rate	1990	128.180042	133.7412917	122.2615351	
DALYs	Sweden	Both	Age-standardized	Aortic aneurysm	Rate	2019	80.57221283	87.74677991	73.81566813	-37.14%
DALYs	Sweden	Both	All Ages	Aortic aneurysm	Number	1990	19209.20802	20064.14873	18281.82443	
DALYs	Sweden	Both	All Ages	Aortic aneurysm	Number	2019	16884.21857	18480.50033	15278.95495	-12.10%
Deaths	Sweden	Both	Age-standardized	Aortic aneurysm	Rate	1990	6.794860456	7.101208795	6.394753226	
Deaths	Sweden	Both	Age-standardized	Aortic aneurysm	Rate	2019	4.753893013	5.223034813	4.25707125	-30.04%
Deaths	Sweden	Both	All Ages	Aortic aneurysm	Number	1990	1110.908615	1165.410064	1042.778376	
Deaths	Sweden	Both	All Ages	Aortic aneurysm	Number	2019	1133.505819	1256.391809	998.2465554	2.03%
DALYs	Switzerland	Both	Age-standardized	Aortic aneurysm	Rate	1990	85.23987169	99.26080679	72.32715761	
DALYs	Switzerland	Both	Age-standardized	Aortic aneurysm	Rate	2019	50.36503636	56.21097662	44.65761489	-40.91%
DALYs	Switzerland	Both	All Ages	Aortic aneurysm	Number	1990	8779.89609	10231.79985	7448.957649	
DALYs	Switzerland	Both	All Ages	Aortic aneurysm	Number	2019	8753.034144	9793.967314	7672.539328	-0.31%
Deaths	Switzerland	Both	Age-standardized	Aortic aneurysm	Rate	1990	4.462566728	5.21170728	3.810606643	
Deaths	Switzerland	Both	Age-standardized	Aortic aneurysm	Rate	2019	2.992717628	3.390221989	2.593750741	-32.94%
Deaths	Switzerland	Both	All Ages	Aortic aneurysm	Number	1990	493.5844294	579.1966375	420.3844198	
Deaths	Switzerland	Both	All Ages	Aortic aneurysm	Number	2019	592.0271713	678.5385732	508.8823218	19.94%
DALYs	Syrian Arab Republic	Both	Age-standardized	Aortic aneurysm	Rate	1990	22.01217185	30.43552106	15.29230178	
DALYs	Syrian Arab Republic	Both	Age-standardized	Aortic aneurysm	Rate	2019	21.79261681	28.82431732	16.66230695	-1.00%
DALYs	Syrian Arab Republic	Both	All Ages	Aortic aneurysm	Number	1990	1263.3951	1798.660737	859.0572178	
DALYs	Syrian Arab Republic	Both	All Ages	Aortic aneurysm	Number	2019	2595.117969	3488.99637	1944.827646	105.41%
Deaths	Syrian Arab Republic	Both	Age-standardized	Aortic aneurysm	Rate	1990	1.090515165	1.444775502	0.781146656	

Deaths	Syrian Arab Republic	Both	Age-standardized	Aortic aneurysm	Rate	2019	1.178558386	1.526130296	0.923223484	8.07%
Deaths	Syrian Arab Republic	Both	All Ages	Aortic aneurysm	Number	1990	50.78384257	68.84293038	35.95768068	
Deaths	Syrian Arab Republic	Both	All Ages	Aortic aneurysm	Number	2019	113.1384133	148.0648264	86.9181035	122.78%
DALYs	Taiwan (Province of China)	Both	Age-standardized	Aortic aneurysm	Rate	1990	27.83469492	29.62093941	26.06568885	
DALYs	Taiwan (Province of China)	Both	Age-standardized	Aortic aneurysm	Rate	2019	59.2365261	75.83923471	46.35980722	112.82%
DALYs	Taiwan (Province of China)	Both	All Ages	Aortic aneurysm	Number	1990	4575.259337	4866.95952	4278.182639	
DALYs	Taiwan (Province of China)	Both	All Ages	Aortic aneurysm	Number	2019	22363.71206	28649.044	17394.54287	388.80%
Deaths	Taiwan (Province of China)	Both	Age-standardized	Aortic aneurysm	Rate	1990	1.324919826	1.430486637	1.22928402	
Deaths	Taiwan (Province of China)	Both	Age-standardized	Aortic aneurysm	Rate	2019	2.793662023	3.538158069	2.220410082	110.86%
Deaths	Taiwan (Province of China)	Both	All Ages	Aortic aneurysm	Number	1990	186.1170881	199.26219	173.8887672	
Deaths	Taiwan (Province of China)	Both	All Ages	Aortic aneurysm	Number	2019	1098.747556	1389.201285	872.1005331	490.35%
DALYs	Tajikistan	Both	Age-standardized	Aortic aneurysm	Rate	1990	41.39005756	48.06066337	33.8142742	
DALYs	Tajikistan	Both	Age-standardized	Aortic aneurysm	Rate	2019	56.43806983	68.46455893	46.11623333	36.36%
DALYs	Tajikistan	Both	All Ages	Aortic aneurysm	Number	1990	1136.547674	1315.04026	938.0048456	
DALYs	Tajikistan	Both	All Ages	Aortic aneurysm	Number	2019	2389.436768	2958.038926	1920.813065	110.24%
Deaths	Tajikistan	Both	Age-standardized	Aortic aneurysm	Rate	1990	2.319489078	2.807847673	1.697124775	
Deaths	Tajikistan	Both	Age-standardized	Aortic aneurysm	Rate	2019	3.370615363	4.056209547	2.809178252	45.32%
Deaths	Tajikistan	Both	All Ages	Aortic aneurysm	Number	1990	61.6000372	74.4974009	45.72637512	
Deaths	Tajikistan	Both	All Ages	Aortic aneurysm	Number	2019	105.9555893	128.8712949	86.41848856	72.01%
DALYs	Thailand	Both	Age-standardized	Aortic aneurysm	Rate	1990	35.74043245	43.01129387	29.63148797	
DALYs	Thailand	Both	Age-standardized	Aortic aneurysm	Rate	2019	32.62179578	41.85912406	24.51792626	-8.73%
DALYs	Thailand	Both	All Ages	Aortic aneurysm	Number	1990	11804.8192	14289.43658	9832.707602	
DALYs	Thailand	Both	All Ages	Aortic aneurysm	Number	2019	31717.31805	40808.99048	23759.23055	168.68%
Deaths	Thailand	Both	Age-standardized	Aortic aneurysm	Rate	1990	2.211453947	2.628408658	1.821501124	
Deaths	Thailand	Both	Age-standardized	Aortic aneurysm	Rate	2019	1.957380348	2.483446756	1.468690634	-11.49%
Deaths	Thailand	Both	All Ages	Aortic aneurysm	Number	1990	585.1752386	700.8080469	484.3960465	
Deaths	Thailand	Both	All Ages	Aortic aneurysm	Number	2019	1893.030721	2403.165806	1418.851044	223.50%
DALYs	Timor-Leste	Both	Age-standardized	Aortic aneurysm	Rate	1990	15.48624709	22.823998	9.434145732	
DALYs	Timor-Leste	Both	Age-standardized	Aortic aneurysm	Rate	2019	25.81362264	36.20514061	17.68396026	66.69%
DALYs	Timor-Leste	Both	All Ages	Aortic aneurysm	Number	1990	41.1378866	64.04152576	24.44704648	
DALYs	Timor-Leste	Both	All Ages	Aortic aneurysm	Number	2019	203.7608511	289.8824963	136.1352649	395.31%
Deaths	Timor-Leste	Both	Age-standardized	Aortic aneurysm	Rate	1990	0.873760075	1.24925521	0.550226708	
Deaths	Timor-Leste	Both	Age-standardized	Aortic aneurysm	Rate	2019	1.454548377	1.981401339	1.032540629	66.47%
Deaths	Timor-Leste	Both	All Ages	Aortic aneurysm	Number	1990	1.706628175	2.519585292	1.038665386	
Deaths	Timor-Leste	Both	All Ages	Aortic aneurysm	Number	2019	9.587956178	13.18504718	6.679787378	461.81%
DALYs	Togo	Both	Age-standardized	Aortic aneurysm	Rate	1990	41.73328026	54.30751279	29.46624274	
DALYs	Togo	Both	Age-standardized	Aortic aneurysm	Rate	2019	34.20468558	45.05364238	26.253647	-18.04%
DALYs	Togo	Both	All Ages	Aortic aneurysm	Number	1990	547.6796355	715.4245051	381.0884567	
DALYs	Togo	Both	All Ages	Aortic aneurysm	Number	2019	1337.225989	1788.373196	998.6716881	144.16%
Deaths	Togo	Both	Age-standardized	Aortic aneurysm	Rate	1990	2.05739957	2.641802089	1.459912251	
Deaths	Togo	Both	Age-standardized	Aortic aneurysm	Rate	2019	1.645848469	2.115693011	1.315308153	-20.00%
Deaths	Togo	Both	All Ages	Aortic aneurysm	Number	1990	21.64626839	28.0756908	15.32370931	
Deaths	Togo	Both	All Ages	Aortic aneurysm	Number	2019	50.68489139	66.54975341	39.15688123	134.15%
DALYs	Tokelau	Both	Age-standardized	Aortic aneurysm	Rate	1990	70.51279542	104.8660455	52.06182701	
DALYs	Tokelau	Both	Age-standardized	Aortic aneurysm	Rate	2019	59.16175351	76.98158926	45.95345799	-16.10%
DALYs	Tokelau	Both	All Ages	Aortic aneurysm	Number	1990	0.924505471	1.365717376	0.686275602	
DALYs	Tokelau	Both	All Ages	Aortic aneurysm	Number	2019	0.771767491	1.012891683	0.593481201	-16.52%
Deaths	Tokelau	Both	Age-standardized	Aortic aneurysm	Rate	1990	3.563723261	5.057478207	2.699474233	
Deaths	Tokelau	Both	Age-standardized	Aortic aneurysm	Rate	2019	2.985562483	3.74245623	2.411087763	-16.22%
Deaths	Tokelau	Both	All Ages	Aortic aneurysm	Number	1990	0.045577402	0.065455613	0.034732565	
Deaths	Tokelau	Both	All Ages	Aortic aneurysm	Number	2019	0.036713264	0.046396861	0.029547131	-19.45%
DALYs	Tonga	Both	Age-standardized	Aortic aneurysm	Rate	1990	40.45101107	51.21869664	31.76846712	
DALYs	Tonga	Both	Age-standardized	Aortic aneurysm	Rate	2019	44.36853403	56.35640437	35.03613993	9.68%
DALYs	Tonga	Both	All Ages	Aortic aneurysm	Number	1990	22.9623899	29.09097513	17.76274739	
DALYs	Tonga	Both	All Ages	Aortic aneurysm	Number	2019	35.39354035	45.19881767	27.77323286	54.14%
Deaths	Tonga	Both	Age-standardized	Aortic aneurysm	Rate	1990	1.923583151	2.384055097	1.544410131	
Deaths	Tonga	Both	Age-standardized	Aortic aneurysm	Rate	2019	2.062391269	2.571822169	1.668344072	7.22%
Deaths	Tonga	Both	All Ages	Aortic aneurysm	Number	1990	0.961276099	1.205958297	0.763136206	
Deaths	Tonga	Both	All Ages	Aortic aneurysm	Number	2019	1.590676949	1.985664269	1.281191333	65.48%
DALYs	Trinidad and Tobago	Both	Age-standardized	Aortic aneurysm	Rate	1990	92.11579955	104.4139269	81.75796585	
DALYs	Trinidad and Tobago	Both	Age-standardized	Aortic aneurysm	Rate	2019	87.57652911	115.77638	64.29684611	-4.93%
DALYs	Trinidad and Tobago	Both	All Ages	Aortic aneurysm	Number	1990	791.3892896	895.4796296	703.6997579	
DALYs	Trinidad and Tobago	Both	All Ages	Aortic aneurysm	Number	2019	1605.591817	2124.997377	1173.417586	102.88%
Deaths	Trinidad and Tobago	Both	Age-standardized	Aortic aneurysm	Rate	1990	4.582183389	5.255293092	4.053185708	
Deaths	Trinidad and Tobago	Both	Age-standardized	Aortic aneurysm	Rate	2019	4.362870316	5.639747597	3.258298088	-4.79%
Deaths	Trinidad and Tobago	Both	All Ages	Aortic aneurysm	Number	1990	36.41898324	41.59202828	32.09312588	
Deaths	Trinidad and Tobago	Both	All Ages	Aortic aneurysm	Number	2019	77.83088749	101.8553147	57.5828936	113.71%
DALYs	Tunisia	Both	Age-standardized	Aortic aneurysm	Rate	1990	20.43237855	26.18660445	15.49587166	
DALYs	Tunisia	Both	Age-standardized	Aortic aneurysm	Rate	2019	21.55972745	29.22782726	15.47124673	5.52%
DALYs	Tunisia	Both	All Ages	Aortic aneurysm	Number	1990	1043.877783	1345.972064	788.0751722	
DALYs	Tunisia	Both	All Ages	Aortic aneurysm	Number	2019	2673.780537	3629.575102	1918.080871	156.14%
Deaths	Tunisia	Both	Age-standardized	Aortic aneurysm	Rate	1990	1.054091175	1.341948056	0.806531727	
Deaths	Tunisia	Both	Age-standardized	Aortic aneurysm	Rate	2019	1.12058088	1.491748568	0.829246248	6.31%
Deaths	Tunisia	Both	All Ages	Aortic aneurysm	Number	1990	44.97571975	57.83801398	34.16023908	
Deaths	Tunisia	Both	All Ages	Aortic aneurysm	Number	2019	129.1237749	172.7596586	95.00619527	187.10%
DALYs	Turkey	Both	Age-standardized	Aortic aneurysm	Rate	1990	73.75245896	98.91345153	55.55396779	
DALYs	Turkey	Both	Age-standardized	Aortic aneurysm	Rate	2019	48.05822868	60.30743015	37.91654577	-34.84%
DALYs	Turkey	Both	All Ages	Aortic aneurysm	Number	1990	29384.70861	40016.44821	21924.2876	
DALYs	Turkey	Both	All Ages	Aortic aneurysm	Number	2019	43589.25761	54969.26866	34188.88981	48.34%
Deaths	Turkey	Both	Age-standardized	Aortic aneurysm	Rate	1990	2.952426232	3.818841895	2.273512309	
Deaths	Turkey	Both	Age-standardized	Aortic aneurysm	Rate	2019	2.081342177	2.573307993	1.664019124	-29.50%
Deaths	Turkey	Both	All Ages	Aortic aneurysm	Number	1990	1047.516494	1381.203777	795.0644921	
Deaths	Turkey	Both	All Ages	Aortic aneurysm	Number	2019	1803.035335	2235.546432	1438.570524	72.12%
DALYs	Turkmenistan	Both	Age-standardized	Aortic aneurysm	Rate	1990	21.91980811	25.82715358	19.41922379	
DALYs	Turkmenistan	Both	Age-standardized	Aortic aneurysm	Rate	2019	51.97050544	65.69667464	41.48328313	137.09%
DALYs	Turkmenistan	Both	All Ages	Aortic aneurysm	Number	1990	434.077124	510.0854583	382.9526435	
DALYs	Turkmenistan	Both	All Ages	Aortic aneurysm	Number	2019	2136.659242	2723.668901	1688.526168	392.23%
Deaths	Turkmenistan	Both	Age-standardized	Aortic aneurysm	Rate	1990	1.041314307	1.227549588	0.920069019	
Deaths	Turkmenistan	Both	Age-standardized	Aortic aneurysm	Rate	2019	2.344665571	2.904398878	1.904235536	125.16%
Deaths	Turkmenistan	Both	All Ages	Aortic aneurysm	Number	1990	17.69924941	20.70348731	15.71074119	
Deaths	Turkmenistan	Both	All Ages	Aortic aneurysm	Number	2019	83.02917612	103.7764589	66.75923503	369.11%
DALYs	Tuvalu	Both	Age-standardized	Aortic aneurysm	Rate	1990	90.41598604	132.4436912	61.93022769	
DALYs	Tuvalu	Both	Age-standardized	Aortic aneurysm	Rate	2019	74.0676954	100.2148675	55.18818466	-18.08%
DALYs	Tuvalu	Both	All Ages	Aortic aneurysm	Number	1990	6.180921063	9.261752919	4.106703522	
DALYs	Tuvalu	Both	All Ages	Aortic aneurysm	Number	2019	7.571047815	10.28772008	5.521081947	22.49%
Deaths	Tuvalu	Both	Age-standardized	Aortic aneurysm	Rate	1990	4.369202706	6.175678287	3.144677796	
Deaths	Tuvalu	Both	Age-standardized	Aortic aneurysm	Rate	2019	3.519648963	4.600890587	2.704275575	-19.44%
Deaths	Tuvalu	Both	All Ages	Aortic aneurysm	Number	1990	0.244981591	0.357495256	0.169137724	
Deaths	Tuvalu	Both	All Ages	Aortic aneurysm	Number	2019	0.314002364	0.416018431	0.238798324	28.17%
DALYs	Uganda	Both	Age-standardized	Aortic aneurysm	Rate	1990	51.55178188	79.2273698	23.61401247	
DALYs	Uganda	Both	Age-standardized	Aortic aneurysm	Rate	2019	41.47732306	57.48176841	25.6163708	-19.54%

DALYs	Uganda	Both	All Ages	Aortic aneurysm	Number	1990	3478.573881	5472.103664	1590.30421	
DALYs	Uganda	Both	All Ages	Aortic aneurysm	Number	2019	6440.908198	9165.775173	3929.16571	85.16%
Deaths	Uganda	Both	Age-standardized	Aortic aneurysm	Rate	1990	2.558686483	3.801988815	1.170152287	
Deaths	Uganda	Both	Age-standardized	Aortic aneurysm	Rate	2019	2.067329582	2.765114472	1.263057039	-19.20%
Deaths	Uganda	Both	All Ages	Aortic aneurysm	Number	1990	142.9313036	218.0073126	65.45752881	
Deaths	Uganda	Both	All Ages	Aortic aneurysm	Number	2019	250.0085224	341.5997173	155.1238717	74.92%
DALYs	Ukraine	Both	Age-standardized	Aortic aneurysm	Rate	1990	43.03240434	50.17229094	37.59718443	
DALYs	Ukraine	Both	Age-standardized	Aortic aneurysm	Rate	2019	73.43012759	88.84335696	61.06967958	70.64%
DALYs	Ukraine	Both	All Ages	Aortic aneurysm	Number	1990	30545.41942	35480.79756	26670.38421	
DALYs	Ukraine	Both	All Ages	Aortic aneurysm	Number	2019	50692.30043	60938.46556	42092.03618	65.96%
Deaths	Ukraine	Both	Age-standardized	Aortic aneurysm	Rate	1990	1.793431339	2.066576052	1.582373023	
Deaths	Ukraine	Both	Age-standardized	Aortic aneurysm	Rate	2019	2.749617104	3.268896227	2.306936691	53.32%
Deaths	Ukraine	Both	All Ages	Aortic aneurysm	Number	1990	1269.392123	1465.815019	1117.83534	
Deaths	Ukraine	Both	All Ages	Aortic aneurysm	Number	2019	2028.32854	2407.328561	1700.563084	59.79%
DALYs	United Arab Emirates	Both	Age-standardized	Aortic aneurysm	Rate	1990	42.61224582	60.95499296	28.07739678	
DALYs	United Arab Emirates	Both	Age-standardized	Aortic aneurysm	Rate	2019	43.78042112	71.61547701	22.64476532	2.74%
DALYs	United Arab Emirates	Both	All Ages	Aortic aneurysm	Number	1990	301.9403164	445.7044888	204.5611058	
DALYs	United Arab Emirates	Both	All Ages	Aortic aneurysm	Number	2019	2933.490319	5243.37861	1392.103757	871.55%
Deaths	United Arab Emirates	Both	Age-standardized	Aortic aneurysm	Rate	1990	1.986327197	2.817301319	1.273593145	
Deaths	United Arab Emirates	Both	Age-standardized	Aortic aneurysm	Rate	2019	2.034293875	3.320789003	1.076227383	2.41%
Deaths	United Arab Emirates	Both	All Ages	Aortic aneurysm	Number	1990	8.154915567	11.88586549	5.514752878	
Deaths	United Arab Emirates	Both	All Ages	Aortic aneurysm	Number	2019	76.41540123	133.4087451	37.0566811	837.05%
DALYs	United Kingdom	Both	Age-standardized	Aortic aneurysm	Rate	1990	165.590958	169.3149563	159.8782178	
DALYs	United Kingdom	Both	Age-standardized	Aortic aneurysm	Rate	2019	91.14458349	95.20597177	85.11627592	-44.96%
DALYs	United Kingdom	Both	All Ages	Aortic aneurysm	Number	1990	155990.281	159660.2432	150289.6531	
DALYs	United Kingdom	Both	All Ages	Aortic aneurysm	Number	2019	118690.6138	124391.9958	109779.0784	-23.91%
Deaths	United Kingdom	Both	Age-standardized	Aortic aneurysm	Rate	1990	9.571488351	9.852872387	9.075858433	
Deaths	United Kingdom	Both	Age-standardized	Aortic aneurysm	Rate	2019	5.896238628	6.222279954	5.33148781	-38.40%
Deaths	United Kingdom	Both	All Ages	Aortic aneurysm	Number	1990	9319.162603	9588.461724	8847.952889	
Deaths	United Kingdom	Both	All Ages	Aortic aneurysm	Number	2019	8431.251244	8920.59886	7552.003395	-9.53%
DALYs	United Republic of Tanzania	Both	Age-standardized	Aortic aneurysm	Rate	1990	69.98467177	96.10669112	40.9066486	
DALYs	United Republic of Tanzania	Both	Age-standardized	Aortic aneurysm	Rate	2019	52.28504243	74.70120287	34.08124903	-25.29%
DALYs	United Republic of Tanzania	Both	All Ages	Aortic aneurysm	Number	1990	7859.897536	10910.06544	4589.301916	
DALYs	United Republic of Tanzania	Both	All Ages	Aortic aneurysm	Number	2019	13359.95127	19112.22823	8732.905718	69.98%
Deaths	United Republic of Tanzania	Both	Age-standardized	Aortic aneurysm	Rate	1990	3.512759647	4.77307647	2.045426323	
Deaths	United Republic of Tanzania	Both	Age-standardized	Aortic aneurysm	Rate	2019	2.671379643	3.764840986	1.716367792	-23.95%
Deaths	United Republic of Tanzania	Both	All Ages	Aortic aneurysm	Number	1990	324.6073914	442.8310908	189.0712639	
Deaths	United Republic of Tanzania	Both	All Ages	Aortic aneurysm	Number	2019	565.6960623	803.4382696	368.4832935	74.27%
DALYs	United States of America	Both	Age-standardized	Aortic aneurysm	Rate	1990	96.04036772	99.30148726	92.40482316	
DALYs	United States of America	Both	Age-standardized	Aortic aneurysm	Rate	2019	47.20258301	49.99875473	43.94631684	-50.85%
DALYs	United States of America	Both	All Ages	Aortic aneurysm	Number	1990	309118.9746	319630.5918	296848.3269	
DALYs	United States of America	Both	All Ages	Aortic aneurysm	Number	2019	246559.9842	260469.2506	229399.2498	-20.24%
Deaths	United States of America	Both	Age-standardized	Aortic aneurysm	Rate	1990	4.987355603	5.179589064	4.710520953	
Deaths	United States of America	Both	Age-standardized	Aortic aneurysm	Rate	2019	2.40291012	2.550337959	2.200703307	-51.82%
Deaths	United States of America	Both	All Ages	Aortic aneurysm	Number	1990	16770.51118	17426.50009	15817.25849	
Deaths	United States of America	Both	All Ages	Aortic aneurysm	Number	2019	13939.79989	14827.86153	12628.15779	-16.88%
DALYs	United States Virgin Islands	Both	Age-standardized	Aortic aneurysm	Rate	1990	64.06447781	74.74492392	53.80443164	
DALYs	United States Virgin Islands	Both	Age-standardized	Aortic aneurysm	Rate	2019	66.56237675	79.01392256	55.61912083	3.90%
DALYs	United States Virgin Islands	Both	All Ages	Aortic aneurysm	Number	1990	53.66615967	63.02839315	44.83283554	
DALYs	United States Virgin Islands	Both	All Ages	Aortic aneurysm	Number	2019	124.7752242	148.1277628	104.350802	132.50%
Deaths	United States Virgin Islands	Both	Age-standardized	Aortic aneurysm	Rate	1990	3.35850309	3.889634926	2.831549573	
Deaths	United States Virgin Islands	Both	Age-standardized	Aortic aneurysm	Rate	2019	3.559804614	4.161263081	3.03949981	5.99%
Deaths	United States Virgin Islands	Both	All Ages	Aortic aneurysm	Number	1990	2.455540192	2.865138488	2.068168976	
Deaths	United States Virgin Islands	Both	All Ages	Aortic aneurysm	Number	2019	6.50471362	7.642174872	5.505005251	164.90%
DALYs	Uruguay	Both	Age-standardized	Aortic aneurysm	Rate	1990	109.1012428	119.4128983	99.18018812	
DALYs	Uruguay	Both	Age-standardized	Aortic aneurysm	Rate	2019	100.9130446	110.23342	92.0790916	-7.51%
DALYs	Uruguay	Both	All Ages	Aortic aneurysm	Number	1990	4185.120539	4593.933897	3796.978119	
DALYs	Uruguay	Both	All Ages	Aortic aneurysm	Number	2019	5099.586822	5576.437385	4633.428913	21.85%
Deaths	Uruguay	Both	Age-standardized	Aortic aneurysm	Rate	1990	5.000805887	5.504347113	4.549785294	
Deaths	Uruguay	Both	Age-standardized	Aortic aneurysm	Rate	2019	4.75543298	5.20843606	4.296824653	-4.91%
Deaths	Uruguay	Both	All Ages	Aortic aneurysm	Number	1990	196.9069198	216.9118157	178.4211913	
Deaths	Uruguay	Both	All Ages	Aortic aneurysm	Number	2019	267.1781155	292.4588652	241.7601674	35.69%
DALYs	Uzbekistan	Both	Age-standardized	Aortic aneurysm	Rate	1990	11.11844903	15.37118942	8.078689674	
DALYs	Uzbekistan	Both	Age-standardized	Aortic aneurysm	Rate	2019	32.64824304	38.33695375	27.52440628	193.64%
DALYs	Uzbekistan	Both	All Ages	Aortic aneurysm	Number	1990	1264.631182	1724.462929	951.9489259	
DALYs	Uzbekistan	Both	All Ages	Aortic aneurysm	Number	2019	6223.466336	7478.343313	5116.354491	392.12%
Deaths	Uzbekistan	Both	Age-standardized	Aortic aneurysm	Rate	1990	0.605512211	0.862302748	0.39937313	
Deaths	Uzbekistan	Both	Age-standardized	Aortic aneurysm	Rate	2019	1.823038026	2.092491063	1.561331562	201.07%
Deaths	Uzbekistan	Both	All Ages	Aortic aneurysm	Number	1990	61.94470033	87.74265235	41.96993474	
Deaths	Uzbekistan	Both	All Ages	Aortic aneurysm	Number	2019	237.0971752	282.3940236	196.877326	282.76%
DALYs	Vanuatu	Both	Age-standardized	Aortic aneurysm	Rate	1990	74.04669242	115.8893593	43.93695893	
DALYs	Vanuatu	Both	Age-standardized	Aortic aneurysm	Rate	2019	72.97981779	105.0737508	46.8808588	-1.44%
DALYs	Vanuatu	Both	All Ages	Aortic aneurysm	Number	1990	51.87438983	83.39650012	29.26051528	
DALYs	Vanuatu	Both	All Ages	Aortic aneurysm	Number	2019	134.3410425	197.499601	82.88995638	158.97%
Deaths	Vanuatu	Both	Age-standardized	Aortic aneurysm	Rate	1990	3.571633337	5.331290051	2.250994605	
Deaths	Vanuatu	Both	Age-standardized	Aortic aneurysm	Rate	2019	3.375329643	4.677991642	2.280346136	-5.50%
Deaths	Vanuatu	Both	All Ages	Aortic aneurysm	Number	1990	1.934492015	3.004776713	1.1563188	
Deaths	Vanuatu	Both	All Ages	Aortic aneurysm	Number	2019	5.016450312	7.125859055	3.283384931	159.32%
DALYs	Venezuela (Bolivarian Republic of)	Both	Age-standardized	Aortic aneurysm	Rate	1990	41.14898006	46.10651281	36.91414454	
DALYs	Venezuela (Bolivarian Republic of)	Both	Age-standardized	Aortic aneurysm	Rate	2019	44.92567658	58.77966543	33.75975229	9.18%
DALYs	Venezuela (Bolivarian Republic of)	Both	All Ages	Aortic aneurysm	Number	1990	4098.837439	4578.99667	3687.128727	
DALYs	Venezuela (Bolivarian Republic of)	Both	All Ages	Aortic aneurysm	Number	2019	12881.03251	17019.9431	9658.381541	214.26%
Deaths	Venezuela (Bolivarian Republic of)	Both	Age-standardized	Aortic aneurysm	Rate	1990	2.024454391	2.274916352	1.788560176	
Deaths	Venezuela (Bolivarian Republic of)	Both	Age-standardized	Aortic aneurysm	Rate	2019	2.3139409	2.978692858	1.756356711	14.30%
Deaths	Venezuela (Bolivarian Republic of)	Both	All Ages	Aortic aneurysm	Number	1990	180.0024578	201.5309028	160.4339031	
Deaths	Venezuela (Bolivarian Republic of)	Both	All Ages	Aortic aneurysm	Number	2019	631.2446223	818.227542	477.694165	250.69%
DALYs	Viet Nam	Both	Age-standardized	Aortic aneurysm	Rate	1990	28.12806146	36.11137552	21.81271466	
DALYs	Viet Nam	Both	Age-standardized	Aortic aneurysm	Rate	2019	33.11217155	42.30107514	24.57331834	17.72%
DALYs	Viet Nam	Both	All Ages	Aortic aneurysm	Number	1990	11302.38239	14635.0363	8683.580395	
DALYs	Viet Nam	Both	All Ages	Aortic aneurysm	Number	2019	29784.63021	38394.37748	21747.04084	163.53%
Deaths	Viet Nam	Both	Age-standardized	Aortic aneurysm	Rate	1990	1.521310625	1.905149983	1.218969045	
Deaths	Viet Nam	Both	Age-standardized	Aortic aneurysm	Rate	2019	1.803663955	2.245148682	1.38361551	18.56%
Deaths	Viet Nam	Both	All Ages	Aortic aneurysm	Number	1990	545.0987577	690.6786373	431.5595627	
Deaths	Viet Nam	Both	All Ages	Aortic aneurysm	Number	2019	1439.986847	1813.724667	1087.949644	164.17%
DALYs	Yemen	Both	Age-standardized	Aortic aneurysm	Rate	1990	21.80335659	38.2597015	11.59685858	
DALYs	Yemen	Both	Age-standardized	Aortic aneurysm	Rate	2019	23.31004633	35.18178153	15.65652379	6.91%
DALYs	Yemen	Both	All Ages	Aortic aneurysm	Number	1990	1160.353887	2111.664022	589.2410655	
DALYs	Yemen	Both	All Ages	Aortic aneurysm	Number	2019	3405.587233	5347.991791	2203.479187	193.50%
Deaths	Yemen	Both	Age-standardized	Aortic aneurysm	Rate	1990	0.992309857	1.676813888	0.562238497	
Deaths	Yemen	Both	Age-standardized	Aortic aneurysm	Rate	2019	1.13063586	1.641272537	0.79206013	13.94%
Deaths	Yemen	Both	All Ages	Aortic aneurysm	Number	1990	42.82888652	74.44823134	23.00214791	

Deaths	Yemen	Both	All Ages	Aortic aneurysm	Number	2019	130.7062026	194.8106943	89.08804072	205.18%
DALYs	Zambia	Both	Age-standardized	Aortic aneurysm	Rate	1990	56.86090594	81.65646188	36.21087785	
DALYs	Zambia	Both	Age-standardized	Aortic aneurysm	Rate	2019	64.99527597	86.66285695	48.73913957	14.31%
DALYs	Zambia	Both	All Ages	Aortic aneurysm	Number	1990	1668.914905	2435.528373	1048.752045	
DALYs	Zambia	Both	All Ages	Aortic aneurysm	Number	2019	4646.616701	6338.767256	3387.761559	178.42%
Deaths	Zambia	Both	Age-standardized	Aortic aneurysm	Rate	1990	2.90318921	4.074424262	1.852489115	
Deaths	Zambia	Both	Age-standardized	Aortic aneurysm	Rate	2019	3.237975641	4.227967587	2.464436888	11.53%
Deaths	Zambia	Both	All Ages	Aortic aneurysm	Number	1990	67.44003432	96.63190038	42.7903084	
Deaths	Zambia	Both	All Ages	Aortic aneurysm	Number	2019	181.2589483	241.6619618	137.3245736	168.77%
DALYs	Zimbabwe	Both	Age-standardized	Aortic aneurysm	Rate	1990	46.99605989	56.98268294	39.56475039	
DALYs	Zimbabwe	Both	Age-standardized	Aortic aneurysm	Rate	2019	55.1336222	71.65446531	42.33207984	17.32%
DALYs	Zimbabwe	Both	All Ages	Aortic aneurysm	Number	1990	2066.675652	2531.125301	1728.814491	
DALYs	Zimbabwe	Both	All Ages	Aortic aneurysm	Number	2019	4395.581226	5807.249031	3304.270745	112.69%
Deaths	Zimbabwe	Both	Age-standardized	Aortic aneurysm	Rate	1990	2.134418607	2.558059782	1.824721695	
Deaths	Zimbabwe	Both	Age-standardized	Aortic aneurysm	Rate	2019	2.394334949	3.049619854	1.894568585	12.18%
Deaths	Zimbabwe	Both	All Ages	Aortic aneurysm	Number	1990	77.71129004	93.65054233	66.00699322	
Deaths	Zimbabwe	Both	All Ages	Aortic aneurysm	Number	2019	154.1929831	198.6213686	119.0553321	98.42%
DALYs	Afghanistan	Female	Age-standardized	Aortic aneurysm	Rate	1990	10.95667377	27.18414345	7.11326597	
DALYs	Afghanistan	Female	Age-standardized	Aortic aneurysm	Rate	2019	12.09828896	26.78296323	7.782973528	10.42%
DALYs	Afghanistan	Female	All Ages	Aortic aneurysm	Number	1990	379.4099059	1026.136255	238.7670931	
DALYs	Afghanistan	Female	All Ages	Aortic aneurysm	Number	2019	876.5713803	2325.998458	530.2608066	131.04%
Deaths	Afghanistan	Female	Age-standardized	Aortic aneurysm	Rate	1990	0.53238692	1.083610515	0.364401852	
Deaths	Afghanistan	Female	Age-standardized	Aortic aneurysm	Rate	2019	0.607014739	1.122554567	0.411853731	14.02%
Deaths	Afghanistan	Female	All Ages	Aortic aneurysm	Number	1990	15.11325254	34.6551241	10.04555515	
Deaths	Afghanistan	Female	All Ages	Aortic aneurysm	Number	2019	32.15658278	71.59130837	20.73137158	112.77%
DALYs	Albania	Female	Age-standardized	Aortic aneurysm	Rate	1990	13.33509781	15.03968207	11.77828598	
DALYs	Albania	Female	Age-standardized	Aortic aneurysm	Rate	2019	13.15109064	17.52818558	9.525454711	-1.38%
DALYs	Albania	Female	All Ages	Aortic aneurysm	Number	1990	152.7192899	172.8056797	135.3777572	
DALYs	Albania	Female	All Ages	Aortic aneurysm	Number	2019	281.4860731	372.3249499	203.3331276	84.32%
Deaths	Albania	Female	Age-standardized	Aortic aneurysm	Rate	1990	0.699286282	0.804418325	0.604445365	
Deaths	Albania	Female	Age-standardized	Aortic aneurysm	Rate	2019	0.724475072	0.95355425	0.530714504	3.60%
Deaths	Albania	Female	All Ages	Aortic aneurysm	Number	1990	7.28804974	8.347286795	6.342116317	
Deaths	Albania	Female	All Ages	Aortic aneurysm	Number	2019	16.6101202	21.77976208	12.13119076	127.91%
DALYs	Algeria	Female	Age-standardized	Aortic aneurysm	Rate	1990	13.65190616	21.33627494	9.878519541	
DALYs	Algeria	Female	Age-standardized	Aortic aneurysm	Rate	2019	12.59976556	17.5410794	9.445796184	-7.71%
DALYs	Algeria	Female	All Ages	Aortic aneurysm	Number	1990	849.1589148	1360.247563	599.2035466	
DALYs	Algeria	Female	All Ages	Aortic aneurysm	Number	2019	2057.652087	2928.214983	1520.565234	142.32%
Deaths	Algeria	Female	Age-standardized	Aortic aneurysm	Rate	1990	0.729426972	1.120794217	0.536887666	
Deaths	Algeria	Female	Age-standardized	Aortic aneurysm	Rate	2019	0.692345399	0.955572467	0.518489142	-5.08%
Deaths	Algeria	Female	All Ages	Aortic aneurysm	Number	1990	33.34616506	49.0472643	24.05292784	
Deaths	Algeria	Female	All Ages	Aortic aneurysm	Number	2019	91.14300004	126.7157697	68.55601984	173.32%
DALYs	American Samoa	Female	Age-standardized	Aortic aneurysm	Rate	1990	83.60316887	99.0858274	68.88738489	
DALYs	American Samoa	Female	Age-standardized	Aortic aneurysm	Rate	2019	35.86654503	44.51154871	28.79717366	-57.10%
DALYs	American Samoa	Female	All Ages	Aortic aneurysm	Number	1990	8.709356399	10.45958268	7.117501406	
DALYs	American Samoa	Female	All Ages	Aortic aneurysm	Number	2019	8.578575517	10.67704335	6.836519127	-1.50%
Deaths	American Samoa	Female	Age-standardized	Aortic aneurysm	Rate	1990	4.891997925	5.81139884	3.978601667	
Deaths	American Samoa	Female	Age-standardized	Aortic aneurysm	Rate	2019	2.053709869	2.546471092	1.632910823	-58.02%
Deaths	American Samoa	Female	All Ages	Aortic aneurysm	Number	1990	0.40097629	0.472815213	0.329463093	
Deaths	American Samoa	Female	All Ages	Aortic aneurysm	Number	2019	0.439667132	0.541299706	0.351046358	9.65%
DALYs	Andorra	Female	Age-standardized	Aortic aneurysm	Rate	1990	57.83569997	85.39372129	36.24745854	
DALYs	Andorra	Female	Age-standardized	Aortic aneurysm	Rate	2019	63.08136129	89.36359279	33.05006033	9.07%
DALYs	Andorra	Female	All Ages	Aortic aneurysm	Number	1990	15.02819649	22.34153459	9.198625706	
DALYs	Andorra	Female	All Ages	Aortic aneurysm	Number	2019	45.18173412	63.6736669	24.14631251	200.65%
Deaths	Andorra	Female	Age-standardized	Aortic aneurysm	Rate	1990	3.520640728	5.121873812	2.327895278	
Deaths	Andorra	Female	Age-standardized	Aortic aneurysm	Rate	2019	3.979812909	5.563002273	2.129366353	13.04%
Deaths	Andorra	Female	All Ages	Aortic aneurysm	Number	1990	0.808059744	1.21039629	0.515068665	
Deaths	Andorra	Female	All Ages	Aortic aneurysm	Number	2019	3.20781976	4.422895577	1.739324485	296.98%
DALYs	Angola	Female	Age-standardized	Aortic aneurysm	Rate	1990	42.75875952	65.52790809	27.93948869	
DALYs	Angola	Female	Age-standardized	Aortic aneurysm	Rate	2019	38.14430927	50.38673158	28.30025485	-10.79%
DALYs	Angola	Female	All Ages	Aortic aneurysm	Number	1990	812.3856127	1347.213069	517.8963149	
DALYs	Angola	Female	All Ages	Aortic aneurysm	Number	2019	2296.188433	3116.988058	1672.867583	182.65%
Deaths	Angola	Female	Age-standardized	Aortic aneurysm	Rate	1990	2.211284763	3.165460198	1.480316934	
Deaths	Angola	Female	Age-standardized	Aortic aneurysm	Rate	2019	2.023981844	2.606515693	1.470021866	-8.47%
Deaths	Angola	Female	All Ages	Aortic aneurysm	Number	1990	32.75478866	50.2418145	21.32919769	
Deaths	Angola	Female	All Ages	Aortic aneurysm	Number	2019	94.44858378	124.4965434	70.02182056	188.35%
DALYs	Antigua and Barbuda	Female	Age-standardized	Aortic aneurysm	Rate	1990	29.99471728	34.33975635	25.8375028	
DALYs	Antigua and Barbuda	Female	Age-standardized	Aortic aneurysm	Rate	2019	32.11308126	37.66266085	27.13663792	7.06%
DALYs	Antigua and Barbuda	Female	All Ages	Aortic aneurysm	Number	1990	9.615652195	10.95034564	8.249490514	
DALYs	Antigua and Barbuda	Female	All Ages	Aortic aneurysm	Number	2019	16.56907308	19.45844398	13.91726337	72.31%
Deaths	Antigua and Barbuda	Female	Age-standardized	Aortic aneurysm	Rate	1990	1.713090529	1.958284636	1.453977373	
Deaths	Antigua and Barbuda	Female	Age-standardized	Aortic aneurysm	Rate	2019	1.947455517	2.25410316	1.651235745	13.68%
Deaths	Antigua and Barbuda	Female	All Ages	Aortic aneurysm	Number	1990	0.611888884	0.701200318	0.512496446	
Deaths	Antigua and Barbuda	Female	All Ages	Aortic aneurysm	Number	2019	0.955264952	1.106823012	0.81513405	56.12%
DALYs	Argentina	Female	Age-standardized	Aortic aneurysm	Rate	1990	32.64163491	36.21583173	29.35240389	
DALYs	Argentina	Female	Age-standardized	Aortic aneurysm	Rate	2019	30.9147129	33.91441281	27.79620841	-5.29%
DALYs	Argentina	Female	All Ages	Aortic aneurysm	Number	1990	5803.729887	6472.811101	5199.614631	
DALYs	Argentina	Female	All Ages	Aortic aneurysm	Number	2019	9301.465209	10229.33736	8333.860666	60.27%
Deaths	Argentina	Female	Age-standardized	Aortic aneurysm	Rate	1990	1.68945898	1.911294976	1.492980918	
Deaths	Argentina	Female	Age-standardized	Aortic aneurysm	Rate	2019	1.628209484	1.796449774	1.440033318	-3.63%
Deaths	Argentina	Female	All Ages	Aortic aneurysm	Number	1990	298.359365	338.1802215	262.260546	
Deaths	Argentina	Female	All Ages	Aortic aneurysm	Number	2019	532.7611997	591.1747663	467.3733404	78.56%
DALYs	Armenia	Female	Age-standardized	Aortic aneurysm	Rate	1990	59.61700824	69.33865613	53.51621851	
DALYs	Armenia	Female	Age-standardized	Aortic aneurysm	Rate	2019	98.14327819	117.5898629	81.53346716	64.62%
DALYs	Armenia	Female	All Ages	Aortic aneurysm	Number	1990	882.5460746	1028.711463	788.4970318	
DALYs	Armenia	Female	All Ages	Aortic aneurysm	Number	2019	2375.132955	2838.5656	1966.700052	169.12%
Deaths	Armenia	Female	Age-standardized	Aortic aneurysm	Rate	1990	3.310401223	3.860766439	2.937100011	
Deaths	Armenia	Female	Age-standardized	Aortic aneurysm	Rate	2019	5.885603433	6.914282655	4.890098384	77.79%
Deaths	Armenia	Female	All Ages	Aortic aneurysm	Number	1990	45.02702711	52.4275657	40.0839951	
Deaths	Armenia	Female	All Ages	Aortic aneurysm	Number	2019	143.5614101	169.8173033	119.3052419	218.83%
DALYs	Australia	Female	Age-standardized	Aortic aneurysm	Rate	1990	67.0656928	71.51680922	61.77445652	
DALYs	Australia	Female	Age-standardized	Aortic aneurysm	Rate	2019	30.17390755	34.10044228	25.72235083	-55.01%
DALYs	Australia	Female	All Ages	Aortic aneurysm	Number	1990	7573.931116	8079.976997	6973.356204	
DALYs	Australia	Female	All Ages	Aortic aneurysm	Number	2019	7153.994112	8103.710809	5971.798032	-5.54%
Deaths	Australia	Female	Age-standardized	Aortic aneurysm	Rate	1990	4.172397733	4.492818711	3.751965692	
Deaths	Australia	Female	Age-standardized	Aortic aneurysm	Rate	2019	2.056616855	2.356249104	1.697655656	-50.71%
Deaths	Australia	Female	All Ages	Aortic aneurysm	Number	1990	481.662424	518.8135802	432.8465549	
Deaths	Australia	Female	All Ages	Aortic aneurysm	Number	2019	549.0329701	631.690023	441.3409316	13.99%
DALYs	Austria	Female	Age-standardized	Aortic aneurysm	Rate	1990	36.26045365	39.07838155	33.73815188	
DALYs	Austria	Female	Age-standardized	Aortic aneurysm	Rate	2019	28.91935945	32.06628465	25.66370789	-20.25%
DALYs	Austria	Female	All Ages	Aortic aneurysm	Number	1990	2484.265685	2702.069816	2305.319642	
DALYs	Austria	Female	All Ages	Aortic aneurysm	Number	2019	2667.615458	2985.039061	2335.88245	7.38%

Deaths	Austria	Female	Age-standardized	Aortic aneurysm	Rate	1990	1.796502967	1.977916044	1.651325315	
Deaths	Austria	Female	Age-standardized	Aortic aneurysm	Rate	2019	1.513373221	1.703383588	1.309132154	-15.76%
Deaths	Austria	Female	All Ages	Aortic aneurysm	Number	1990	142.4188914	158.9702093	129.5994053	
Deaths	Austria	Female	All Ages	Aortic aneurysm	Number	2019	172.6895933	196.4271385	146.1943577	21.25%
DALYs	Azerbaijan	Female	Age-standardized	Aortic aneurysm	Rate	1990	7.987717365	9.430879677	6.101619933	
DALYs	Azerbaijan	Female	Age-standardized	Aortic aneurysm	Rate	2019	9.750726101	12.27469305	7.641607712	22.07%
DALYs	Azerbaijan	Female	All Ages	Aortic aneurysm	Number	1990	234.3603249	277.7112494	181.3789848	
DALYs	Azerbaijan	Female	All Ages	Aortic aneurysm	Number	2019	457.2148355	586.8442577	347.2111381	95.09%
Deaths	Azerbaijan	Female	Age-standardized	Aortic aneurysm	Rate	1990	0.444656931	0.537667165	0.292796337	
Deaths	Azerbaijan	Female	Age-standardized	Aortic aneurysm	Rate	2019	0.565732364	0.694894784	0.446339943	27.23%
Deaths	Azerbaijan	Female	All Ages	Aortic aneurysm	Number	1990	12.77204006	15.46459297	8.41486572	
Deaths	Azerbaijan	Female	All Ages	Aortic aneurysm	Number	2019	21.08483516	26.49777459	16.46050453	65.09%
DALYs	Bahamas	Female	Age-standardized	Aortic aneurysm	Rate	1990	55.26141992	62.8845455	48.26971259	
DALYs	Bahamas	Female	Age-standardized	Aortic aneurysm	Rate	2019	58.89426284	73.04732558	47.64089977	6.57%
DALYs	Bahamas	Female	All Ages	Aortic aneurysm	Number	1990	48.6124265	55.15881712	42.50678734	
DALYs	Bahamas	Female	All Ages	Aortic aneurysm	Number	2019	122.9832785	153.4116763	99.14015602	152.99%
Deaths	Bahamas	Female	Age-standardized	Aortic aneurysm	Rate	1990	2.901232248	3.310918293	2.504081495	
Deaths	Bahamas	Female	Age-standardized	Aortic aneurysm	Rate	2019	3.156126815	3.910106424	2.549319705	8.79%
Deaths	Bahamas	Female	All Ages	Aortic aneurysm	Number	1990	2.36109998	2.696631339	2.047449773	
Deaths	Bahamas	Female	All Ages	Aortic aneurysm	Number	2019	6.173159058	7.619344101	4.987829552	161.45%
DALYs	Bahrain	Female	Age-standardized	Aortic aneurysm	Rate	1990	13.94673772	16.76151391	11.67469326	
DALYs	Bahrain	Female	Age-standardized	Aortic aneurysm	Rate	2019	15.36109509	18.91275781	12.35352496	10.14%
DALYs	Bahrain	Female	All Ages	Aortic aneurysm	Number	1990	11.31933308	13.66969236	9.380985366	
DALYs	Bahrain	Female	All Ages	Aortic aneurysm	Number	2019	49.29432103	61.8229859	39.03373846	335.49%
Deaths	Bahrain	Female	Age-standardized	Aortic aneurysm	Rate	1990	0.792581921	0.963396473	0.658315893	
Deaths	Bahrain	Female	Age-standardized	Aortic aneurysm	Rate	2019	0.986194681	1.208584981	0.803529714	24.43%
Deaths	Bahrain	Female	All Ages	Aortic aneurysm	Number	1990	0.442690017	0.531504239	0.368126751	
Deaths	Bahrain	Female	All Ages	Aortic aneurysm	Number	2019	2.139671136	2.657502443	1.71546807	383.33%
DALYs	Bangladesh	Female	Age-standardized	Aortic aneurysm	Rate	1990	19.60317595	33.68776231	13.82317595	
DALYs	Bangladesh	Female	Age-standardized	Aortic aneurysm	Rate	2019	22.81269399	29.90326514	15.09752067	16.37%
DALYs	Bangladesh	Female	All Ages	Aortic aneurysm	Number	1990	4202.621574	8017.175203	2884.11247	
DALYs	Bangladesh	Female	All Ages	Aortic aneurysm	Number	2019	14156.89933	18369.6449	9473.560657	236.86%
Deaths	Bangladesh	Female	Age-standardized	Aortic aneurysm	Rate	1990	1.061737275	1.611869459	0.747955585	
Deaths	Bangladesh	Female	Age-standardized	Aortic aneurysm	Rate	2019	1.273551325	1.694424571	0.836628899	19.95%
Deaths	Bangladesh	Female	All Ages	Aortic aneurysm	Number	1990	183.0874221	302.0434746	128.8499373	
Deaths	Bangladesh	Female	All Ages	Aortic aneurysm	Number	2019	685.5668487	905.0626102	449.0557175	274.45%
DALYs	Barbados	Female	Age-standardized	Aortic aneurysm	Rate	1990	25.17856533	28.4284697	22.17859536	
DALYs	Barbados	Female	Age-standardized	Aortic aneurysm	Rate	2019	29.81827774	36.3517863	24.20114217	18.43%
DALYs	Barbados	Female	All Ages	Aortic aneurysm	Number	1990	44.51989172	50.86087349	38.83083867	
DALYs	Barbados	Female	All Ages	Aortic aneurysm	Number	2019	81.0419072	98.61143707	65.63351064	82.04%
Deaths	Barbados	Female	Age-standardized	Aortic aneurysm	Rate	1990	1.471596835	1.676339761	1.285025318	
Deaths	Barbados	Female	Age-standardized	Aortic aneurysm	Rate	2019	1.787881355	2.143742316	1.478000226	21.49%
Deaths	Barbados	Female	All Ages	Aortic aneurysm	Number	1990	2.831574317	3.280879367	2.435950688	
Deaths	Barbados	Female	All Ages	Aortic aneurysm	Number	2019	5.042000697	6.053679758	4.164878913	78.06%
DALYs	Belarus	Female	Age-standardized	Aortic aneurysm	Rate	1990	22.01741382	26.23646598	17.69203719	
DALYs	Belarus	Female	Age-standardized	Aortic aneurysm	Rate	2019	30.50618386	40.56119841	23.23085272	38.55%
DALYs	Belarus	Female	All Ages	Aortic aneurysm	Number	1990	1750.852235	2086.236077	1420.48528	
DALYs	Belarus	Female	All Ages	Aortic aneurysm	Number	2019	2857.660255	3719.462265	2191.906838	63.22%
Deaths	Belarus	Female	Age-standardized	Aortic aneurysm	Rate	1990	1.008202255	1.199663543	0.843762826	
Deaths	Belarus	Female	Age-standardized	Aortic aneurysm	Rate	2019	1.446330063	1.877334864	1.12569097	43.46%
Deaths	Belarus	Female	All Ages	Aortic aneurysm	Number	1990	84.14260929	100.2252016	70.89693859	
Deaths	Belarus	Female	All Ages	Aortic aneurysm	Number	2019	153.0718399	197.8212785	119.7270417	81.92%
DALYs	Belgium	Female	Age-standardized	Aortic aneurysm	Rate	1990	30.746442	33.11099258	28.2460815	
DALYs	Belgium	Female	Age-standardized	Aortic aneurysm	Rate	2019	23.03105948	25.74350813	20.4276124	-25.09%
DALYs	Belgium	Female	All Ages	Aortic aneurysm	Number	1990	2815.493887	3053.555259	2567.056375	
DALYs	Belgium	Female	All Ages	Aortic aneurysm	Number	2019	3020.027055	3413.257116	2605.76588	7.26%
Deaths	Belgium	Female	Age-standardized	Aortic aneurysm	Rate	1990	1.860355076	2.015197114	1.678877657	
Deaths	Belgium	Female	Age-standardized	Aortic aneurysm	Rate	2019	1.422710301	1.616725932	1.21247549	-23.52%
Deaths	Belgium	Female	All Ages	Aortic aneurysm	Number	1990	189.1024555	206.4329236	169.2732674	
Deaths	Belgium	Female	All Ages	Aortic aneurysm	Number	2019	233.7699539	268.5062337	194.5508521	23.62%
DALYs	Belize	Female	Age-standardized	Aortic aneurysm	Rate	1990	26.77762981	31.75292142	22.5366794	
DALYs	Belize	Female	Age-standardized	Aortic aneurysm	Rate	2019	27.94797204	32.75944825	23.60115937	4.37%
DALYs	Belize	Female	All Ages	Aortic aneurysm	Number	1990	12.65712227	14.93686555	10.65076105	
DALYs	Belize	Female	All Ages	Aortic aneurysm	Number	2019	37.53196004	44.05146997	31.59729751	196.53%
Deaths	Belize	Female	Age-standardized	Aortic aneurysm	Rate	1990	1.424713985	1.701522557	1.214703756	
Deaths	Belize	Female	Age-standardized	Aortic aneurysm	Rate	2019	1.528572811	1.804645741	1.274343831	7.29%
Deaths	Belize	Female	All Ages	Aortic aneurysm	Number	1990	0.660913629	0.789143182	0.563163449	
Deaths	Belize	Female	All Ages	Aortic aneurysm	Number	2019	1.851708042	2.159588948	1.54866145	180.17%
DALYs	Benin	Female	Age-standardized	Aortic aneurysm	Rate	1990	34.23449225	45.29179138	21.86930724	
DALYs	Benin	Female	Age-standardized	Aortic aneurysm	Rate	2019	17.00749573	24.76533954	12.11177271	-50.32%
DALYs	Benin	Female	All Ages	Aortic aneurysm	Number	1990	358.3758059	480.8408832	227.1172078	
DALYs	Benin	Female	All Ages	Aortic aneurysm	Number	2019	459.8170078	669.380366	319.029669	28.31%
Deaths	Benin	Female	Age-standardized	Aortic aneurysm	Rate	1990	1.729948574	2.301689699	1.101688636	
Deaths	Benin	Female	Age-standardized	Aortic aneurysm	Rate	2019	0.884069488	1.254479073	0.642988753	-48.90%
Deaths	Benin	Female	All Ages	Aortic aneurysm	Number	1990	16.12789452	21.47690341	10.21901455	
Deaths	Benin	Female	All Ages	Aortic aneurysm	Number	2019	19.98371091	28.61503718	14.4108073	23.91%
DALYs	Bermuda	Female	Age-standardized	Aortic aneurysm	Rate	1990	70.87185631	83.66503956	61.86098528	
DALYs	Bermuda	Female	Age-standardized	Aortic aneurysm	Rate	2019	39.31503499	49.72576038	31.34073514	-44.53%
DALYs	Bermuda	Female	All Ages	Aortic aneurysm	Number	1990	24.98522695	29.57795983	21.73417558	
DALYs	Bermuda	Female	All Ages	Aortic aneurysm	Number	2019	30.42685516	38.50349888	24.08310182	21.78%
Deaths	Bermuda	Female	Age-standardized	Aortic aneurysm	Rate	1990	4.425284874	5.133313823	3.821277199	
Deaths	Bermuda	Female	Age-standardized	Aortic aneurysm	Rate	2019	2.674906973	3.374342446	2.106883169	-39.55%
Deaths	Bermuda	Female	All Ages	Aortic aneurysm	Number	1990	1.520394964	1.775340818	1.317954506	
Deaths	Bermuda	Female	All Ages	Aortic aneurysm	Number	2019	2.326531567	2.939486754	1.813753734	53.02%
DALYs	Bhutan	Female	Age-standardized	Aortic aneurysm	Rate	1990	18.85939377	35.84780858	12.19430606	
DALYs	Bhutan	Female	Age-standardized	Aortic aneurysm	Rate	2019	27.03150945	36.23811942	16.73749533	43.33%
DALYs	Bhutan	Female	All Ages	Aortic aneurysm	Number	1990	22.63136245	45.37093439	14.17473599	
DALYs	Bhutan	Female	All Ages	Aortic aneurysm	Number	2019	71.34849689	97.25350254	44.26966737	215.26%
Deaths	Bhutan	Female	Age-standardized	Aortic aneurysm	Rate	1990	1.018478863	1.792768367	0.682089887	
Deaths	Bhutan	Female	Age-standardized	Aortic aneurysm	Rate	2019	1.587771662	2.100259685	0.992171857	55.90%
Deaths	Bhutan	Female	All Ages	Aortic aneurysm	Number	1990	1.011567556	1.873275819	0.662747614	
Deaths	Bhutan	Female	All Ages	Aortic aneurysm	Number	2019	3.751065178	4.999129334	2.355988689	270.82%
DALYs	Bolivia (Plurinational State of)	Female	Age-standardized	Aortic aneurysm	Rate	1990	24.59778464	39.29801754	16.31848039	
DALYs	Bolivia (Plurinational State of)	Female	Age-standardized	Aortic aneurysm	Rate	2019	29.3111981	41.70735549	17.88825643	19.16%
DALYs	Bolivia (Plurinational State of)	Female	All Ages	Aortic aneurysm	Number	1990	434.7790289	716.8225051	283.3390639	
DALYs	Bolivia (Plurinational State of)	Female	All Ages	Aortic aneurysm	Number	2019	1331.396099	1914.813477	793.8812141	206.22%
Deaths	Bolivia (Plurinational State of)	Female	Age-standardized	Aortic aneurysm	Rate	1990	1.264551877	1.89221921	0.866098798	
Deaths	Bolivia (Plurinational State of)	Female	Age-standardized	Aortic aneurysm	Rate	2019	1.640138139	2.261718911	1.056322553	29.70%
Deaths	Bolivia (Plurinational State of)	Female	All Ages	Aortic aneurysm	Number	1990	18.70356296	28.9531825	12.53976296	
Deaths	Bolivia (Plurinational State of)	Female	All Ages	Aortic aneurysm	Number	2019	65.49039052	91.96219072	41.26945456	250.15%
DALYs	Bosnia and Herzegovina	Female	Age-standardized	Aortic aneurysm	Rate	1990	36.16392588	44.62593193	31.35438453	

DALYs	Bosnia and Herzegovina	Female	Age-standardized	Aortic aneurysm	Rate	2019	44.35642916	56.08730169	34.84901946	22.65%
DALYs	Bosnia and Herzegovina	Female	All Ages	Aortic aneurysm	Number	1990	798.5867269	1011.35433	689.3707126	
DALYs	Bosnia and Herzegovina	Female	All Ages	Aortic aneurysm	Number	2019	1366.478691	1723.035314	1076.046779	71.11%
Deaths	Bosnia and Herzegovina	Female	Age-standardized	Aortic aneurysm	Rate	1990	1.875869392	2.196022026	1.616784584	
Deaths	Bosnia and Herzegovina	Female	Age-standardized	Aortic aneurysm	Rate	2019	2.426671315	3.001192789	1.918710648	29.36%
Deaths	Bosnia and Herzegovina	Female	All Ages	Aortic aneurysm	Number	1990	35.63416977	42.59778627	30.75607517	
Deaths	Bosnia and Herzegovina	Female	All Ages	Aortic aneurysm	Number	2019	77.9405592	96.96298196	61.42174796	118.72%
DALYs	Botswana	Female	Age-standardized	Aortic aneurysm	Rate	1990	38.21518645	54.22726381	26.89934099	
DALYs	Botswana	Female	Age-standardized	Aortic aneurysm	Rate	2019	23.15414459	34.08419201	15.58189964	-39.41%
DALYs	Botswana	Female	All Ages	Aortic aneurysm	Number	1990	126.715705	180.1937421	87.18883728	
DALYs	Botswana	Female	All Ages	Aortic aneurysm	Number	2019	189.1499538	284.52246	120.7209659	49.27%
Deaths	Botswana	Female	Age-standardized	Aortic aneurysm	Rate	1990	1.986376999	2.759403416	1.421525104	
Deaths	Botswana	Female	Age-standardized	Aortic aneurysm	Rate	2019	1.216936999	1.727982565	0.858864612	-38.74%
Deaths	Botswana	Female	All Ages	Aortic aneurysm	Number	1990	5.178689162	7.268313916	3.654501367	
Deaths	Botswana	Female	All Ages	Aortic aneurysm	Number	2019	7.774415648	11.19785528	5.340570349	50.12%
DALYs	Brazil	Female	Age-standardized	Aortic aneurysm	Rate	1990	49.25541543	51.95030863	46.67562923	
DALYs	Brazil	Female	Age-standardized	Aortic aneurysm	Rate	2019	69.44756864	75.73337468	63.19112368	40.99%
DALYs	Brazil	Female	All Ages	Aortic aneurysm	Number	1990	24759.9744	26086.26057	23550.21004	
DALYs	Brazil	Female	All Ages	Aortic aneurysm	Number	2019	90191.60718	98327.77326	82121.77476	264.26%
Deaths	Brazil	Female	Age-standardized	Aortic aneurysm	Rate	1990	2.208029018	2.344346366	2.054441777	
Deaths	Brazil	Female	Age-standardized	Aortic aneurysm	Rate	2019	3.455424489	3.787884011	3.068246552	56.49%
Deaths	Brazil	Female	All Ages	Aortic aneurysm	Number	1990	972.8346713	1030.016085	915.7828792	
Deaths	Brazil	Female	All Ages	Aortic aneurysm	Number	2019	4468.471608	4896.670324	3970.051314	359.32%
DALYs	Brunei Darussalam	Female	Age-standardized	Aortic aneurysm	Rate	1990	80.15507315	117.6713747	63.88461207	
DALYs	Brunei Darussalam	Female	Age-standardized	Aortic aneurysm	Rate	2019	89.38949786	105.5240781	75.98472118	11.52%
DALYs	Brunei Darussalam	Female	All Ages	Aortic aneurysm	Number	1990	37.40919491	56.12588024	29.72607535	
DALYs	Brunei Darussalam	Female	All Ages	Aortic aneurysm	Number	2019	123.0619215	147.0252617	103.1977261	228.96%
Deaths	Brunei Darussalam	Female	Age-standardized	Aortic aneurysm	Rate	1990	4.59687067	6.599883414	3.684156073	
Deaths	Brunei Darussalam	Female	Age-standardized	Aortic aneurysm	Rate	2019	5.456369769	6.387288786	4.65267419	18.70%
Deaths	Brunei Darussalam	Female	All Ages	Aortic aneurysm	Number	1990	1.702874515	2.498506964	1.361119553	
Deaths	Brunei Darussalam	Female	All Ages	Aortic aneurysm	Number	2019	5.848944127	6.932743098	4.964237907	243.47%
DALYs	Bulgaria	Female	Age-standardized	Aortic aneurysm	Rate	1990	23.14496113	26.0672174	20.82037651	
DALYs	Bulgaria	Female	Age-standardized	Aortic aneurysm	Rate	2019	33.65616135	43.86059893	25.4531256	45.41%
DALYs	Bulgaria	Female	All Ages	Aortic aneurysm	Number	1990	1406.786305	1590.109049	1259.040808	
DALYs	Bulgaria	Female	All Ages	Aortic aneurysm	Number	2019	2172.281661	2766.889301	1678.419851	54.41%
Deaths	Bulgaria	Female	Age-standardized	Aortic aneurysm	Rate	1990	0.916487897	1.029068145	0.826455949	
Deaths	Bulgaria	Female	Age-standardized	Aortic aneurysm	Rate	2019	1.3256234	1.674852341	1.03500389	44.64%
Deaths	Bulgaria	Female	All Ages	Aortic aneurysm	Number	1990	55.92700019	63.30697427	49.98024223	
Deaths	Bulgaria	Female	All Ages	Aortic aneurysm	Number	2019	104.1760178	130.7736155	82.22160925	86.27%
DALYs	Burkina Faso	Female	Age-standardized	Aortic aneurysm	Rate	1990	33.88994321	54.45665842	16.55640582	
DALYs	Burkina Faso	Female	Age-standardized	Aortic aneurysm	Rate	2019	21.58803198	34.13795751	12.77955902	-36.30%
DALYs	Burkina Faso	Female	All Ages	Aortic aneurysm	Number	1990	781.7287709	1257.796623	378.1665861	
DALYs	Burkina Faso	Female	All Ages	Aortic aneurysm	Number	2019	1074.023607	1726.853578	636.3680522	37.39%
Deaths	Burkina Faso	Female	Age-standardized	Aortic aneurysm	Rate	1990	1.68013831	2.638799299	0.82267728	
Deaths	Burkina Faso	Female	Age-standardized	Aortic aneurysm	Rate	2019	1.119626108	1.737494992	0.662476571	-33.36%
Deaths	Burkina Faso	Female	All Ages	Aortic aneurysm	Number	1990	32.65340987	52.02610267	15.98243575	
Deaths	Burkina Faso	Female	All Ages	Aortic aneurysm	Number	2019	46.82304857	73.71050479	27.84863525	43.39%
DALYs	Burundi	Female	Age-standardized	Aortic aneurysm	Rate	1990	46.74596043	81.9777271	23.42051532	
DALYs	Burundi	Female	Age-standardized	Aortic aneurysm	Rate	2019	27.15334548	38.2557518	15.09379551	-41.91%
DALYs	Burundi	Female	All Ages	Aortic aneurysm	Number	1990	598.2742105	1106.307896	293.2302888	
DALYs	Burundi	Female	All Ages	Aortic aneurysm	Number	2019	598.7676997	859.9311407	329.6276094	0.08%
Deaths	Burundi	Female	Age-standardized	Aortic aneurysm	Rate	1990	2.479290916	3.810133815	1.282515068	
Deaths	Burundi	Female	Age-standardized	Aortic aneurysm	Rate	2019	1.474232198	2.054396307	0.832453973	-40.54%
Deaths	Burundi	Female	All Ages	Aortic aneurysm	Number	1990	26.63501171	44.61570102	13.4535763	
Deaths	Burundi	Female	All Ages	Aortic aneurysm	Number	2019	25.06893518	35.06609893	14.00080631	-5.88%
DALYs	Cabo Verde	Female	Age-standardized	Aortic aneurysm	Rate	1990	11.34771506	14.27326737	9.256796566	
DALYs	Cabo Verde	Female	Age-standardized	Aortic aneurysm	Rate	2019	12.64392221	15.49877701	9.981106901	11.42%
DALYs	Cabo Verde	Female	All Ages	Aortic aneurysm	Number	1990	15.20840386	19.14467026	12.41944732	
DALYs	Cabo Verde	Female	All Ages	Aortic aneurysm	Number	2019	31.29890159	38.21038119	24.85330541	105.80%
Deaths	Cabo Verde	Female	Age-standardized	Aortic aneurysm	Rate	1990	0.555675036	0.681913812	0.450188482	
Deaths	Cabo Verde	Female	Age-standardized	Aortic aneurysm	Rate	2019	0.679135425	0.835837409	0.538611294	22.22%
Deaths	Cabo Verde	Female	All Ages	Aortic aneurysm	Number	1990	0.767444605	0.940961535	0.617093816	
Deaths	Cabo Verde	Female	All Ages	Aortic aneurysm	Number	2019	1.694000702	2.085957651	1.338276667	120.73%
DALYs	Cambodia	Female	Age-standardized	Aortic aneurysm	Rate	1990	13.03889644	25.38760648	9.207432024	
DALYs	Cambodia	Female	Age-standardized	Aortic aneurysm	Rate	2019	16.06902869	22.92150372	11.88542784	23.24%
DALYs	Cambodia	Female	All Ages	Aortic aneurysm	Number	1990	326.5646338	679.1864571	226.9979536	
DALYs	Cambodia	Female	All Ages	Aortic aneurysm	Number	2019	1073.20309	1564.103079	786.2769136	228.63%
Deaths	Cambodia	Female	Age-standardized	Aortic aneurysm	Rate	1990	0.699802936	1.219905723	0.506649804	
Deaths	Cambodia	Female	Age-standardized	Aortic aneurysm	Rate	2019	0.88147329	1.2469063	0.667279764	25.96%
Deaths	Cambodia	Female	All Ages	Aortic aneurysm	Number	1990	14.02205478	26.47261048	9.982645165	
Deaths	Cambodia	Female	All Ages	Aortic aneurysm	Number	2019	50.99322709	71.6669538	38.11664104	263.66%
DALYs	Cameroon	Female	Age-standardized	Aortic aneurysm	Rate	1990	37.15093132	59.77427238	23.85032574	
DALYs	Cameroon	Female	Age-standardized	Aortic aneurysm	Rate	2019	17.89883443	28.65697003	11.70346254	-51.82%
DALYs	Cameroon	Female	All Ages	Aortic aneurysm	Number	1990	855.1148717	1405.10291	545.1010873	
DALYs	Cameroon	Female	All Ages	Aortic aneurysm	Number	2019	1172.932645	1921.518859	737.6350423	37.17%
Deaths	Cameroon	Female	Age-standardized	Aortic aneurysm	Rate	1990	1.866698881	2.917626805	1.233046858	
Deaths	Cameroon	Female	Age-standardized	Aortic aneurysm	Rate	2019	0.912240195	1.422570132	0.623869026	-51.13%
Deaths	Cameroon	Female	All Ages	Aortic aneurysm	Number	1990	35.53912373	56.54789706	22.96393793	
Deaths	Cameroon	Female	All Ages	Aortic aneurysm	Number	2019	47.72984576	76.31010161	31.8101213	34.30%
DALYs	Canada	Female	Age-standardized	Aortic aneurysm	Rate	1990	57.09048007	60.95880653	52.49436784	
DALYs	Canada	Female	Age-standardized	Aortic aneurysm	Rate	2019	27.31977828	30.99772973	23.76934627	-52.15%
DALYs	Canada	Female	All Ages	Aortic aneurysm	Number	1990	10822.28559	11568.38064	9965.450489	
DALYs	Canada	Female	All Ages	Aortic aneurysm	Number	2019	10591.01009	12086.36885	9093.547355	-2.14%
Deaths	Canada	Female	Age-standardized	Aortic aneurysm	Rate	1990	3.572550378	3.834607979	3.216309613	
Deaths	Canada	Female	Age-standardized	Aortic aneurysm	Rate	2019	1.822739778	2.09661926	1.524873087	-48.98%
Deaths	Canada	Female	All Ages	Aortic aneurysm	Number	1990	699.0265488	750.6952536	629.7090555	
Deaths	Canada	Female	All Ages	Aortic aneurysm	Number	2019	799.8550632	925.2669038	661.9203683	14.42%
DALYs	Central African Republic	Female	Age-standardized	Aortic aneurysm	Rate	1990	49.0112941	81.73212402	25.84190612	
DALYs	Central African Republic	Female	Age-standardized	Aortic aneurysm	Rate	2019	34.84476736	56.00724952	21.26814851	-28.90%
DALYs	Central African Republic	Female	All Ages	Aortic aneurysm	Number	1990	289.3184466	526.3732106	148.1663362	
DALYs	Central African Republic	Female	All Ages	Aortic aneurysm	Number	2019	388.7828855	676.3613689	230.2905839	34.38%
Deaths	Central African Republic	Female	Age-standardized	Aortic aneurysm	Rate	1990	2.575366188	3.93074748	1.429260711	
Deaths	Central African Republic	Female	Age-standardized	Aortic aneurysm	Rate	2019	1.838296432	2.791631456	1.162655071	-28.62%
Deaths	Central African Republic	Female	All Ages	Aortic aneurysm	Number	1990	12.06407893	19.98842054	6.39897444	
Deaths	Central African Republic	Female	All Ages	Aortic aneurysm	Number	2019	16.16359582	25.8647201	9.845005948	33.98%
DALYs	Chad	Female	Age-standardized	Aortic aneurysm	Rate	1990	31.94803147	47.54564987	15.26187376	
DALYs	Chad	Female	Age-standardized	Aortic aneurysm	Rate	2019	17.00837244	26.05306537	10.09794189	-46.76%
DALYs	Chad	Female	All Ages	Aortic aneurysm	Number	1990	469.272478	702.9134473	222.599701	
DALYs	Chad	Female	All Ages	Aortic aneurysm	Number	2019	462.2735532	737.0073865	268.8519975	-1.49%
Deaths	Chad	Female	Age-standardized	Aortic aneurysm	Rate	1990	1.602839082	2.307585566	0.776403286	
Deaths	Chad	Female	Age-standardized	Aortic aneurysm	Rate	2019	0.852186367	1.258594646	0.526889819	-46.83%

Deaths	Chad	Female	All Ages	Aortic aneurysm	Number	1990	21.03215912	30.87250077	10.19353765	
Deaths	Chad	Female	All Ages	Aortic aneurysm	Number	2019	18.7941782	28.49852161	11.31577517	-10.64%
DALYs	Chile	Female	Age-standardized	Aortic aneurysm	Rate	1990	31.00524182	33.98137671	28.40220537	
DALYs	Chile	Female	Age-standardized	Aortic aneurysm	Rate	2019	36.32384281	40.20892353	32.20349425	17.15%
DALYs	Chile	Female	All Ages	Aortic aneurysm	Number	1990	1694.568248	1855.238657	1552.679389	
DALYs	Chile	Female	All Ages	Aortic aneurysm	Number	2019	4744.989291	5257.489296	4198.723197	180.01%
Deaths	Chile	Female	Age-standardized	Aortic aneurysm	Rate	1990	1.678101543	1.851055741	1.514250804	
Deaths	Chile	Female	Age-standardized	Aortic aneurysm	Rate	2019	2.079928447	2.336148196	1.793026322	23.95%
Deaths	Chile	Female	All Ages	Aortic aneurysm	Number	1990	83.9121118	92.59632277	76.0875822	
Deaths	Chile	Female	All Ages	Aortic aneurysm	Number	2019	284.5707699	320.3035361	244.3166486	239.13%
DALYs	China	Female	Age-standardized	Aortic aneurysm	Rate	1990	12.48185004	19.49076977	9.769862033	
DALYs	China	Female	Age-standardized	Aortic aneurysm	Rate	2019	9.850342617	12.07348714	7.81355258	-21.08%
DALYs	China	Female	All Ages	Aortic aneurysm	Number	1990	54553.7295	87732.00438	42385.24581	
DALYs	China	Female	All Ages	Aortic aneurysm	Number	2019	98815.3355	121354.5434	78341.85462	81.13%
Deaths	China	Female	Age-standardized	Aortic aneurysm	Rate	1990	0.635164303	0.935969124	0.511219799	
Deaths	China	Female	Age-standardized	Aortic aneurysm	Rate	2019	0.50898302	0.609943425	0.413239728	-19.87%
Deaths	China	Female	All Ages	Aortic aneurysm	Number	1990	2318.177742	3549.180536	1833.135133	
Deaths	China	Female	All Ages	Aortic aneurysm	Number	2019	4949.400433	5967.442793	4021.957316	113.50%
DALYs	Colombia	Female	Age-standardized	Aortic aneurysm	Rate	1990	39.93480792	42.67238478	37.32227208	
DALYs	Colombia	Female	Age-standardized	Aortic aneurysm	Rate	2019	32.72154314	41.75960763	25.08004872	-18.06%
DALYs	Colombia	Female	All Ages	Aortic aneurysm	Number	1990	3833.136034	4088.164032	3573.335266	
DALYs	Colombia	Female	All Ages	Aortic aneurysm	Number	2019	9345.29498	11885.06895	7173.389938	143.80%
Deaths	Colombia	Female	Age-standardized	Aortic aneurysm	Rate	1990	1.895434021	2.042523538	1.741511848	
Deaths	Colombia	Female	Age-standardized	Aortic aneurysm	Rate	2019	1.691279505	2.12152834	1.286013882	-10.77%
Deaths	Colombia	Female	All Ages	Aortic aneurysm	Number	1990	158.9091675	170.6227428	147.348526	
Deaths	Colombia	Female	All Ages	Aortic aneurysm	Number	2019	501.9599437	625.4371231	383.2593712	215.88%
DALYs	Comoros	Female	Age-standardized	Aortic aneurysm	Rate	1990	49.59471857	69.97963906	30.57989276	
DALYs	Comoros	Female	Age-standardized	Aortic aneurysm	Rate	2019	33.88926667	45.98041679	23.89548721	-31.67%
DALYs	Comoros	Female	All Ages	Aortic aneurysm	Number	1990	57.13966861	83.30806355	32.92732659	
DALYs	Comoros	Female	All Ages	Aortic aneurysm	Number	2019	89.47405066	123.3576391	62.92162175	56.59%
Deaths	Comoros	Female	Age-standardized	Aortic aneurysm	Rate	1990	2.642241742	3.613378424	1.769895712	
Deaths	Comoros	Female	Age-standardized	Aortic aneurysm	Rate	2019	1.824084653	2.469890904	1.285546932	-30.96%
Deaths	Comoros	Female	All Ages	Aortic aneurysm	Number	1990	2.641332538	3.676807582	1.724086518	
Deaths	Comoros	Female	All Ages	Aortic aneurysm	Number	2019	4.365837977	5.874402969	3.072326582	65.29%
DALYs	Congo	Female	Age-standardized	Aortic aneurysm	Rate	1990	61.16049594	82.32752078	43.80509524	
DALYs	Congo	Female	Age-standardized	Aortic aneurysm	Rate	2019	42.4161748	58.38091908	30.80894415	-30.65%
DALYs	Congo	Female	All Ages	Aortic aneurysm	Number	1990	344.5592564	499.4073614	245.9042513	
DALYs	Congo	Female	All Ages	Aortic aneurysm	Number	2019	558.0705344	796.0915759	391.7462551	61.97%
Deaths	Congo	Female	Age-standardized	Aortic aneurysm	Rate	1990	3.29279934	4.349103066	2.376651688	
Deaths	Congo	Female	Age-standardized	Aortic aneurysm	Rate	2019	2.298912943	3.082698346	1.685654409	-30.18%
Deaths	Congo	Female	All Ages	Aortic aneurysm	Number	1990	15.18113523	20.33833296	10.86527584	
Deaths	Congo	Female	All Ages	Aortic aneurysm	Number	2019	24.2972799	33.23202496	17.75453036	60.05%
DALYs	Cook Islands	Female	Age-standardized	Aortic aneurysm	Rate	1990	53.11609543	70.66210689	40.29737853	
DALYs	Cook Islands	Female	Age-standardized	Aortic aneurysm	Rate	2019	39.17154205	50.57784024	28.73950179	-26.25%
DALYs	Cook Islands	Female	All Ages	Aortic aneurysm	Number	1990	3.179262762	4.277918562	2.392583434	
DALYs	Cook Islands	Female	All Ages	Aortic aneurysm	Number	2019	4.811879021	6.214306518	3.540872174	51.35%
Deaths	Cook Islands	Female	Age-standardized	Aortic aneurysm	Rate	1990	2.850555203	3.78691166	2.190246681	
Deaths	Cook Islands	Female	Age-standardized	Aortic aneurysm	Rate	2019	2.237885736	2.862092428	1.680863965	-21.49%
Deaths	Cook Islands	Female	All Ages	Aortic aneurysm	Number	1990	0.150739949	0.199569695	0.114233242	
Deaths	Cook Islands	Female	All Ages	Aortic aneurysm	Number	2019	0.274235733	0.350804537	0.20488209	81.93%
DALYs	Costa Rica	Female	Age-standardized	Aortic aneurysm	Rate	1990	17.61678136	19.87230114	15.57886133	
DALYs	Costa Rica	Female	Age-standardized	Aortic aneurysm	Rate	2019	24.9864044	31.84822567	19.09843355	41.83%
DALYs	Costa Rica	Female	All Ages	Aortic aneurysm	Number	1990	172.0155842	193.7546869	153.3583381	
DALYs	Costa Rica	Female	All Ages	Aortic aneurysm	Number	2019	689.197195	877.131303	528.6058272	300.66%
Deaths	Costa Rica	Female	Age-standardized	Aortic aneurysm	Rate	1990	0.890360862	1.017568487	0.762693995	
Deaths	Costa Rica	Female	Age-standardized	Aortic aneurysm	Rate	2019	1.302326184	1.677380632	1.010719436	46.27%
Deaths	Costa Rica	Female	All Ages	Aortic aneurysm	Number	1990	7.808896632	8.904727878	6.729977763	
Deaths	Costa Rica	Female	All Ages	Aortic aneurysm	Number	2019	36.60285548	46.89115798	28.48684047	368.73%
DALYs	Croatia	Female	Age-standardized	Aortic aneurysm	Rate	1990	23.54237698	26.14126505	21.12471619	
DALYs	Croatia	Female	Age-standardized	Aortic aneurysm	Rate	2019	37.83638382	47.96557051	29.07677186	60.72%
DALYs	Croatia	Female	All Ages	Aortic aneurysm	Number	1990	861.1703912	956.7030813	768.5497438	
DALYs	Croatia	Female	All Ages	Aortic aneurysm	Number	2019	1825.161296	2288.427325	1407.059489	111.94%
Deaths	Croatia	Female	Age-standardized	Aortic aneurysm	Rate	1990	1.103823307	1.222291176	0.98180589	
Deaths	Croatia	Female	Age-standardized	Aortic aneurysm	Rate	2019	2.005382951	2.491163007	1.565593857	81.68%
Deaths	Croatia	Female	All Ages	Aortic aneurysm	Number	1990	40.83763629	45.43140729	36.17711513	
Deaths	Croatia	Female	All Ages	Aortic aneurysm	Number	2019	113.0648143	140.1991916	87.98012473	176.86%
DALYs	Cuba	Female	Age-standardized	Aortic aneurysm	Rate	1990	42.21924449	47.50178055	37.84531579	
DALYs	Cuba	Female	Age-standardized	Aortic aneurysm	Rate	2019	39.667527	49.07876956	32.02111798	-6.04%
DALYs	Cuba	Female	All Ages	Aortic aneurysm	Number	1990	2230.398314	2514.054611	1994.327985	
DALYs	Cuba	Female	All Ages	Aortic aneurysm	Number	2019	4042.827006	4999.142069	3282.201101	81.26%
Deaths	Cuba	Female	Age-standardized	Aortic aneurysm	Rate	1990	2.396135403	2.690587831	2.115928239	
Deaths	Cuba	Female	Age-standardized	Aortic aneurysm	Rate	2019	2.311810939	2.832783785	1.865951651	-3.52%
Deaths	Cuba	Female	All Ages	Aortic aneurysm	Number	1990	124.5254861	140.8180927	109.6662848	
Deaths	Cuba	Female	All Ages	Aortic aneurysm	Number	2019	252.0564494	309.5221722	202.5223659	102.41%
DALYs	Cyprus	Female	Age-standardized	Aortic aneurysm	Rate	1990	66.05426281	87.50996253	54.52534422	
DALYs	Cyprus	Female	Age-standardized	Aortic aneurysm	Rate	2019	35.17604065	41.42370801	29.52320226	-46.75%
DALYs	Cyprus	Female	All Ages	Aortic aneurysm	Number	1990	280.3577252	369.7599131	227.2724116	
DALYs	Cyprus	Female	All Ages	Aortic aneurysm	Number	2019	351.8824708	417.0689913	294.1017588	25.51%
Deaths	Cyprus	Female	Age-standardized	Aortic aneurysm	Rate	1990	3.644861997	5.114850624	2.981514048	
Deaths	Cyprus	Female	Age-standardized	Aortic aneurysm	Rate	2019	1.973118747	2.329918493	1.644320141	-45.87%
Deaths	Cyprus	Female	All Ages	Aortic aneurysm	Number	1990	14.03813123	19.31684154	11.37875334	
Deaths	Cyprus	Female	All Ages	Aortic aneurysm	Number	2019	19.85870122	23.76353401	16.40942229	41.46%
DALYs	Czechia	Female	Age-standardized	Aortic aneurysm	Rate	1990	22.2289203	23.91947405	20.58728897	
DALYs	Czechia	Female	Age-standardized	Aortic aneurysm	Rate	2019	35.32552775	43.24151619	28.35627123	58.92%
DALYs	Czechia	Female	All Ages	Aortic aneurysm	Number	1990	1723.792876	1854.070149	1588.730002	
DALYs	Czechia	Female	All Ages	Aortic aneurysm	Number	2019	3982.105856	4855.458914	3219.227221	131.01%
Deaths	Czechia	Female	Age-standardized	Aortic aneurysm	Rate	1990	1.042227186	1.124426853	0.957553094	
Deaths	Czechia	Female	Age-standardized	Aortic aneurysm	Rate	2019	1.844728369	2.22260974	1.48834864	77.00%
Deaths	Czechia	Female	All Ages	Aortic aneurysm	Number	1990	86.70724261	93.79470054	79.32672528	
Deaths	Czechia	Female	All Ages	Aortic aneurysm	Number	2019	238.7123536	287.0757053	193.2958106	175.31%
DALYs	C么te d'Ivoire	Female	Age-standardized	Aortic aneurysm	Rate	1990	31.66395596	43.81802925	21.18024644	
DALYs	C么te d'Ivoire	Female	Age-standardized	Aortic aneurysm	Rate	2019	15.21572909	23.62755034	10.75098798	-51.95%
DALYs	C么te d'Ivoire	Female	All Ages	Aortic aneurysm	Number	1990	600.592363	856.6856397	398.7074941	
DALYs	C么te d'Ivoire	Female	All Ages	Aortic aneurysm	Number	2019	812.0020072	1294.523608	552.2120665	35.20%
Deaths	C么te d'Ivoire	Female	Age-standardized	Aortic aneurysm	Rate	1990	1.629202611	2.174987023	1.101149856	
Deaths	C么te d'Ivoire	Female	Age-standardized	Aortic aneurysm	Rate	2019	0.79720018	1.191705398	0.588438972	-51.07%
Deaths	C么te d'Ivoire	Female	All Ages	Aortic aneurysm	Number	1990	23.12364149	32.16003198	15.51252762	
Deaths	C么te d'Ivoire	Female	All Ages	Aortic aneurysm	Number	2019	32.85264613	50.52858934	23.54389339	42.07%
DALYs	Democratic People's Republic of Korea	Female	Age-standardized	Aortic aneurysm	Rate	1990	16.953582	25.94238791	11.55388633	
DALYs	Democratic People's Republic of Korea	Female	Age-standardized	Aortic aneurysm	Rate	2019	16.14783341	21.68794759	12.15336821	-4.75%
DALYs	Democratic People's Republic of Korea	Female	All Ages	Aortic aneurysm	Number	1990	1630.322418	2597.056857	1104.852266	

DALYs	Democratic People's Republic of Korea	Female	All Ages	Aortic aneurysm	Number	2019	2941.080665	3940.485494	2223.68787	80.40%
Deaths	Democratic People's Republic of Korea	Female	Age-standardized	Aortic aneurysm	Rate	1990	0.834034139	1.155114183	0.581422821	
Deaths	Democratic People's Republic of Korea	Female	Age-standardized	Aortic aneurysm	Rate	2019	0.753372927	0.965331882	0.588328532	-9.67%
Deaths	Democratic People's Republic of Korea	Female	All Ages	Aortic aneurysm	Number	1990	69.57380328	100.5514433	48.04772972	
Deaths	Democratic People's Republic of Korea	Female	All Ages	Aortic aneurysm	Number	2019	141.3377434	181.7519072	109.9960512	103.15%
DALYs	Democratic Republic of the Congo	Female	Age-standardized	Aortic aneurysm	Rate	1990	46.54410721	65.28640588	29.49111545	
DALYs	Democratic Republic of the Congo	Female	Age-standardized	Aortic aneurysm	Rate	2019	36.28830218	49.6794104	23.71350769	-22.03%
DALYs	Democratic Republic of the Congo	Female	All Ages	Aortic aneurysm	Number	1990	3743.143122	5394.686608	2374.189958	
DALYs	Democratic Republic of the Congo	Female	All Ages	Aortic aneurysm	Number	2019	6935.66756	9484.969176	4552.138342	85.29%
Deaths	Democratic Republic of the Congo	Female	Age-standardized	Aortic aneurysm	Rate	1990	2.457736956	3.308991088	1.545800704	
Deaths	Democratic Republic of the Congo	Female	Age-standardized	Aortic aneurysm	Rate	2019	1.9174127	2.620128819	1.21075826	-21.98%
Deaths	Democratic Republic of the Congo	Female	All Ages	Aortic aneurysm	Number	1990	150.4408034	213.1070798	94.53117884	
Deaths	Democratic Republic of the Congo	Female	All Ages	Aortic aneurysm	Number	2019	307.8755216	419.8505392	199.5449346	104.65%
DALYs	Denmark	Female	Age-standardized	Aortic aneurysm	Rate	1990	57.37890358	63.25975824	51.43514114	
DALYs	Denmark	Female	Age-standardized	Aortic aneurysm	Rate	2019	52.19902089	58.91272908	45.8482717	-9.03%
DALYs	Denmark	Female	All Ages	Aortic aneurysm	Number	1990	2734.014456	3037.916	2422.679607	
DALYs	Denmark	Female	All Ages	Aortic aneurysm	Number	2019	3380.453924	3849.00817	2939.089609	23.64%
Deaths	Denmark	Female	Age-standardized	Aortic aneurysm	Rate	1990	3.164104801	3.515666001	2.790643262	
Deaths	Denmark	Female	Age-standardized	Aortic aneurysm	Rate	2019	3.258457626	3.726829501	2.793389064	2.98%
Deaths	Denmark	Female	All Ages	Aortic aneurysm	Number	1990	166.5811694	186.7679637	145.7404074	
Deaths	Denmark	Female	All Ages	Aortic aneurysm	Number	2019	234.2623512	269.07005	198.6692413	40.63%
DALYs	Djibouti	Female	Age-standardized	Aortic aneurysm	Rate	1990	49.85179643	69.09709218	27.89038495	
DALYs	Djibouti	Female	Age-standardized	Aortic aneurysm	Rate	2019	32.15064705	44.98326001	19.9778713	-35.51%
DALYs	Djibouti	Female	All Ages	Aortic aneurysm	Number	1990	34.76971247	50.6876396	19.12662695	
DALYs	Djibouti	Female	All Ages	Aortic aneurysm	Number	2019	87.33019131	129.4918977	51.88380735	151.17%
Deaths	Djibouti	Female	Age-standardized	Aortic aneurysm	Rate	1990	2.635296656	3.601616323	1.479132012	
Deaths	Djibouti	Female	Age-standardized	Aortic aneurysm	Rate	2019	1.766262592	2.417516862	1.118368872	-32.98%
Deaths	Djibouti	Female	All Ages	Aortic aneurysm	Number	1990	1.333127224	1.851623491	0.746621108	
Deaths	Djibouti	Female	All Ages	Aortic aneurysm	Number	2019	3.584771504	5.017900962	2.220739737	168.90%
DALYs	Dominica	Female	Age-standardized	Aortic aneurysm	Rate	1990	76.7708185	90.83166293	62.22206201	
DALYs	Dominica	Female	Age-standardized	Aortic aneurysm	Rate	2019	72.56834004	89.58012952	58.7104563	-5.47%
DALYs	Dominica	Female	All Ages	Aortic aneurysm	Number	1990	33.73084837	40.09224962	27.5421309	
DALYs	Dominica	Female	All Ages	Aortic aneurysm	Number	2019	34.38191205	42.57904366	27.95915748	1.93%
Deaths	Dominica	Female	Age-standardized	Aortic aneurysm	Rate	1990	4.455362607	5.23311253	3.635278606	
Deaths	Dominica	Female	Age-standardized	Aortic aneurysm	Rate	2019	4.410637896	5.445595055	3.600886693	-1.00%
Deaths	Dominica	Female	All Ages	Aortic aneurysm	Number	1990	2.101783726	2.491796118	1.711560949	
Deaths	Dominica	Female	All Ages	Aortic aneurysm	Number	2019	2.250832195	2.791491382	1.829337331	7.09%
DALYs	Dominican Republic	Female	Age-standardized	Aortic aneurysm	Rate	1990	17.36580728	20.54172398	14.60608516	
DALYs	Dominican Republic	Female	Age-standardized	Aortic aneurysm	Rate	2019	22.59597877	29.74774428	17.04880401	30.12%
DALYs	Dominican Republic	Female	All Ages	Aortic aneurysm	Number	1990	344.1820867	406.5008636	288.9080779	
DALYs	Dominican Republic	Female	All Ages	Aortic aneurysm	Number	2019	1095.128126	1444.262546	819.4305695	218.18%
Deaths	Dominican Republic	Female	Age-standardized	Aortic aneurysm	Rate	1990	0.909964952	1.07872684	0.76261681	
Deaths	Dominican Republic	Female	Age-standardized	Aortic aneurysm	Rate	2019	1.242081646	1.580550243	0.958883494	36.50%
Deaths	Dominican Republic	Female	All Ages	Aortic aneurysm	Number	1990	14.89001145	17.55330966	12.5715295	
Deaths	Dominican Republic	Female	All Ages	Aortic aneurysm	Number	2019	57.1924107	72.93571334	44.09400217	284.10%
DALYs	Ecuador	Female	Age-standardized	Aortic aneurysm	Rate	1990	26.14793628	32.52370111	22.75957818	
DALYs	Ecuador	Female	Age-standardized	Aortic aneurysm	Rate	2019	26.31897638	33.37633866	21.05848063	0.65%
DALYs	Ecuador	Female	All Ages	Aortic aneurysm	Number	1990	759.4205095	942.7657278	660.0523218	
DALYs	Ecuador	Female	All Ages	Aortic aneurysm	Number	2019	2073.474603	2651.625386	1649.795155	173.03%
Deaths	Ecuador	Female	Age-standardized	Aortic aneurysm	Rate	1990	1.36422952	1.713178843	1.158471607	
Deaths	Ecuador	Female	Age-standardized	Aortic aneurysm	Rate	2019	1.45462366	1.805909615	1.177615175	6.63%
Deaths	Ecuador	Female	All Ages	Aortic aneurysm	Number	1990	33.12248697	41.54218607	28.27722481	
Deaths	Ecuador	Female	All Ages	Aortic aneurysm	Number	2019	105.5069075	132.1580283	85.25136973	218.54%
DALYs	Egypt	Female	Age-standardized	Aortic aneurysm	Rate	1990	12.73540946	24.37332851	8.766337345	
DALYs	Egypt	Female	Age-standardized	Aortic aneurysm	Rate	2019	13.54866777	24.12728526	9.329860474	6.39%
DALYs	Egypt	Female	All Ages	Aortic aneurysm	Number	1990	2064.098902	3983.673348	1405.007475	
DALYs	Egypt	Female	All Ages	Aortic aneurysm	Number	2019	4221.194032	7346.415532	2828.033303	104.51%
Deaths	Egypt	Female	Age-standardized	Aortic aneurysm	Rate	1990	0.6095748	1.132902068	0.426146356	
Deaths	Egypt	Female	Age-standardized	Aortic aneurysm	Rate	2019	0.672864033	1.220987257	0.470617917	10.38%
Deaths	Egypt	Female	All Ages	Aortic aneurysm	Number	1990	76.54001114	144.3912333	53.14837539	
Deaths	Egypt	Female	All Ages	Aortic aneurysm	Number	2019	148.6809092	271.161689	101.6935535	94.25%
DALYs	El Salvador	Female	Age-standardized	Aortic aneurysm	Rate	1990	9.449377269	11.28531813	8.192672572	
DALYs	El Salvador	Female	Age-standardized	Aortic aneurysm	Rate	2019	9.095714852	11.94183837	6.640727688	-3.74%
DALYs	El Salvador	Female	All Ages	Aortic aneurysm	Number	1990	161.867953	193.5378746	140.9566681	
DALYs	El Salvador	Female	All Ages	Aortic aneurysm	Number	2019	315.7524507	413.8896578	231.5327288	95.07%
Deaths	El Salvador	Female	Age-standardized	Aortic aneurysm	Rate	1990	0.45794472	0.547558359	0.39138864	
Deaths	El Salvador	Female	Age-standardized	Aortic aneurysm	Rate	2019	0.469233364	0.6055898	0.348740948	2.47%
Deaths	El Salvador	Female	All Ages	Aortic aneurysm	Number	1990	7.20117106	8.576345627	6.170860904	
Deaths	El Salvador	Female	All Ages	Aortic aneurysm	Number	2019	17.22891172	22.09448194	12.85668075	139.25%
DALYs	Equatorial Guinea	Female	Age-standardized	Aortic aneurysm	Rate	1990	42.1313633	71.76433075	24.96744542	
DALYs	Equatorial Guinea	Female	Age-standardized	Aortic aneurysm	Rate	2019	40.57979371	62.00664037	24.45193888	-3.68%
DALYs	Equatorial Guinea	Female	All Ages	Aortic aneurysm	Number	1990	44.95207432	82.50428056	26.37017816	
DALYs	Equatorial Guinea	Female	All Ages	Aortic aneurysm	Number	2019	110.5081296	177.2782889	64.33344898	145.84%
Deaths	Equatorial Guinea	Female	Age-standardized	Aortic aneurysm	Rate	1990	2.19133584	3.492461174	1.334319756	
Deaths	Equatorial Guinea	Female	Age-standardized	Aortic aneurysm	Rate	2019	2.265947676	3.304623422	1.435617308	3.40%
Deaths	Equatorial Guinea	Female	All Ages	Aortic aneurysm	Number	1990	1.949334542	3.244659259	1.155711117	
Deaths	Equatorial Guinea	Female	All Ages	Aortic aneurysm	Number	2019	5.032360637	7.508108187	3.09015763	158.16%
DALYs	Eritrea	Female	Age-standardized	Aortic aneurysm	Rate	1990	45.30179948	80.54600763	24.6910524	
DALYs	Eritrea	Female	Age-standardized	Aortic aneurysm	Rate	2019	36.58534734	51.60774692	22.26190396	-19.24%
DALYs	Eritrea	Female	All Ages	Aortic aneurysm	Number	1990	251.3599197	475.9173672	131.1655161	
DALYs	Eritrea	Female	All Ages	Aortic aneurysm	Number	2019	538.0908568	790.2586573	319.9984166	114.07%
Deaths	Eritrea	Female	Age-standardized	Aortic aneurysm	Rate	1990	2.344450692	3.979917526	1.289066346	
Deaths	Eritrea	Female	Age-standardized	Aortic aneurysm	Rate	2019	1.992573089	2.771533528	1.234812107	-15.01%
Deaths	Eritrea	Female	All Ages	Aortic aneurysm	Number	1990	9.805409561	17.64122724	5.310094251	
Deaths	Eritrea	Female	All Ages	Aortic aneurysm	Number	2019	22.65657278	31.758888	13.9729342	131.06%
DALYs	Estonia	Female	Age-standardized	Aortic aneurysm	Rate	1990	31.78236834	36.01761011	28.21089928	
DALYs	Estonia	Female	Age-standardized	Aortic aneurysm	Rate	2019	31.41273754	42.17923941	23.56104346	-1.16%
DALYs	Estonia	Female	All Ages	Aortic aneurysm	Number	1990	399.3615127	454.4886589	354.9570141	
DALYs	Estonia	Female	All Ages	Aortic aneurysm	Number	2019	477.6800418	646.1261868	358.4232956	19.61%
Deaths	Estonia	Female	Age-standardized	Aortic aneurysm	Rate	1990	1.438456894	1.628879348	1.275266551	
Deaths	Estonia	Female	Age-standardized	Aortic aneurysm	Rate	2019	1.574692595	2.138847213	1.186552771	9.47%
Deaths	Estonia	Female	All Ages	Aortic aneurysm	Number	1990	19.19120973	21.91870721	16.95252229	
Deaths	Estonia	Female	All Ages	Aortic aneurysm	Number	2019	29.66568845	40.74577296	22.42807625	54.58%
DALYs	Eswatini	Female	Age-standardized	Aortic aneurysm	Rate	1990	34.61396267	47.83463198	26.08098734	
DALYs	Eswatini	Female	Age-standardized	Aortic aneurysm	Rate	2019	21.47649057	33.16325373	13.04808287	-37.95%
DALYs	Eswatini	Female	All Ages	Aortic aneurysm	Number	1990	62.3270632	85.48585692	46.21750897	
DALYs	Eswatini	Female	All Ages	Aortic aneurysm	Number	2019	76.78808338	122.8336268	44.42458722	23.20%
Deaths	Eswatini	Female	Age-standardized	Aortic aneurysm	Rate	1990	1.809501981	2.427668542	1.374151036	
Deaths	Eswatini	Female	Age-standardized	Aortic aneurysm	Rate	2019	1.161854022	1.719592433	0.750458008	-35.79%
Deaths	Eswatini	Female	All Ages	Aortic aneurysm	Number	1990	2.597950458	3.531921418	1.99543071	
Deaths	Eswatini	Female	All Ages	Aortic aneurysm	Number	2019	3.236035203	4.905131352	2.025488738	24.56%

DALYs	Ethiopia	Female	Age-standardized	Aortic aneurysm	Rate	1990	41.69626388	69.35483757	16.86827265	
DALYs	Ethiopia	Female	Age-standardized	Aortic aneurysm	Rate	2019	26.8162656	36.78995649	15.63740453	-35.69%
DALYs	Ethiopia	Female	All Ages	Aortic aneurysm	Number	1990	3884.992897	7373.523019	1475.782615	
DALYs	Ethiopia	Female	All Ages	Aortic aneurysm	Number	2019	5508.931069	7573.275531	3209.065621	41.80%
Deaths	Ethiopia	Female	Age-standardized	Aortic aneurysm	Rate	1990	2.194434369	3.421907304	0.918752843	
Deaths	Ethiopia	Female	Age-standardized	Aortic aneurysm	Rate	2019	1.500275162	2.071228131	0.864089652	-31.63%
Deaths	Ethiopia	Female	All Ages	Aortic aneurysm	Number	1990	154.5303493	257.3324322	62.55026522	
Deaths	Ethiopia	Female	All Ages	Aortic aneurysm	Number	2019	247.7771335	340.2222957	144.6131608	60.34%
DALYs	Fiji	Female	Age-standardized	Aortic aneurysm	Rate	1990	91.6696688	116.7621423	69.93734661	
DALYs	Fiji	Female	Age-standardized	Aortic aneurysm	Rate	2019	79.76476242	101.7623381	61.03301858	-12.99%
DALYs	Fiji	Female	All Ages	Aortic aneurysm	Number	1990	167.0769576	216.1648035	125.8779659	
DALYs	Fiji	Female	All Ages	Aortic aneurysm	Number	2019	301.1039077	387.0318561	226.3273191	80.22%
Deaths	Fiji	Female	Age-standardized	Aortic aneurysm	Rate	1990	4.832379171	6.079853383	3.696683516	
Deaths	Fiji	Female	Age-standardized	Aortic aneurysm	Rate	2019	4.299388679	5.471717193	3.38222223	-11.03%
Deaths	Fiji	Female	All Ages	Aortic aneurysm	Number	1990	6.685593624	8.51701693	5.090374736	
Deaths	Fiji	Female	All Ages	Aortic aneurysm	Number	2019	13.20235022	16.87285184	10.0980724	97.47%
DALYs	Finland	Female	Age-standardized	Aortic aneurysm	Rate	1990	53.36716398	64.29027673	46.73810718	
DALYs	Finland	Female	Age-standardized	Aortic aneurysm	Rate	2019	45.07383967	51.27590376	38.96284027	-15.54%
DALYs	Finland	Female	All Ages	Aortic aneurysm	Number	1990	2424.088287	2941.689441	2101.277023	
DALYs	Finland	Female	All Ages	Aortic aneurysm	Number	2019	3190.652038	3629.346533	2722.420851	31.62%
Deaths	Finland	Female	Age-standardized	Aortic aneurysm	Rate	1990	3.123656779	3.783606322	2.704782329	
Deaths	Finland	Female	Age-standardized	Aortic aneurysm	Rate	2019	2.81142157	3.198567312	2.383102112	-10.00%
Deaths	Finland	Female	All Ages	Aortic aneurysm	Number	1990	152.1666977	185.6391266	131.0940445	
Deaths	Finland	Female	All Ages	Aortic aneurysm	Number	2019	235.6573096	270.7049242	196.3410193	54.87%
DALYs	France	Female	Age-standardized	Aortic aneurysm	Rate	1990	23.99355043	25.52463406	22.17880165	
DALYs	France	Female	Age-standardized	Aortic aneurysm	Rate	2019	17.80161072	20.05737366	15.40608633	-25.81%
DALYs	France	Female	All Ages	Aortic aneurysm	Number	1990	12395.01067	13310.59894	11251.30966	
DALYs	France	Female	All Ages	Aortic aneurysm	Number	2019	14183.62217	16048.11076	11877.4309	14.43%
Deaths	France	Female	Age-standardized	Aortic aneurysm	Rate	1990	1.534680161	1.658249685	1.363520833	
Deaths	France	Female	Age-standardized	Aortic aneurysm	Rate	2019	1.087940743	1.238642294	0.899886123	-29.11%
Deaths	France	Female	All Ages	Aortic aneurysm	Number	1990	904.8483063	988.0942613	796.9555099	
Deaths	France	Female	All Ages	Aortic aneurysm	Number	2019	1143.59628	1339.803921	923.3758711	26.39%
DALYs	Gabon	Female	Age-standardized	Aortic aneurysm	Rate	1990	57.27394098	76.0428663	35.24624672	
DALYs	Gabon	Female	Age-standardized	Aortic aneurysm	Rate	2019	40.67841997	55.24121703	29.4777634	-28.98%
DALYs	Gabon	Female	All Ages	Aortic aneurysm	Number	1990	173.0290312	230.8893377	106.4742634	
DALYs	Gabon	Female	All Ages	Aortic aneurysm	Number	2019	223.7253713	310.7402096	160.5917768	29.30%
Deaths	Gabon	Female	Age-standardized	Aortic aneurysm	Rate	1990	3.0922159	3.986747856	1.924162032	
Deaths	Gabon	Female	Age-standardized	Aortic aneurysm	Rate	2019	2.232410515	2.911750125	1.684618661	-27.81%
Deaths	Gabon	Female	All Ages	Aortic aneurysm	Number	1990	8.525718537	10.9944731	5.23028075	
Deaths	Gabon	Female	All Ages	Aortic aneurysm	Number	2019	10.90389621	14.35001275	8.182471091	27.89%
DALYs	Gambia	Female	Age-standardized	Aortic aneurysm	Rate	1990	29.97905266	40.88032232	16.47949536	
DALYs	Gambia	Female	Age-standardized	Aortic aneurysm	Rate	2019	18.39795577	24.6882939	13.86261423	-38.63%
DALYs	Gambia	Female	All Ages	Aortic aneurysm	Number	1990	50.69629076	70.41798453	27.75596556	
DALYs	Gambia	Female	All Ages	Aortic aneurysm	Number	2019	95.07882142	128.8624231	70.74290682	87.55%
Deaths	Gambia	Female	Age-standardized	Aortic aneurysm	Rate	1990	1.573083909	2.085451637	0.880121263	
Deaths	Gambia	Female	Age-standardized	Aortic aneurysm	Rate	2019	0.969131223	1.269335656	0.749882662	-38.39%
Deaths	Gambia	Female	All Ages	Aortic aneurysm	Number	1990	2.235883189	3.023356863	1.245029237	
Deaths	Gambia	Female	All Ages	Aortic aneurysm	Number	2019	4.339730309	5.712907828	3.370476919	94.09%
DALYs	Georgia	Female	Age-standardized	Aortic aneurysm	Rate	1990	7.713473754	10.37979703	6.169604727	
DALYs	Georgia	Female	Age-standardized	Aortic aneurysm	Rate	2019	17.85073178	21.78989198	14.40803877	131.42%
DALYs	Georgia	Female	All Ages	Aortic aneurysm	Number	1990	277.931763	372.152678	221.1402992	
DALYs	Georgia	Female	All Ages	Aortic aneurysm	Number	2019	629.5242462	764.5808779	505.9167764	126.50%
Deaths	Georgia	Female	Age-standardized	Aortic aneurysm	Rate	1990	0.407731929	0.547375122	0.327381612	
Deaths	Georgia	Female	Age-standardized	Aortic aneurysm	Rate	2019	0.920120911	1.116202399	0.735157584	125.67%
Deaths	Georgia	Female	All Ages	Aortic aneurysm	Number	1990	14.14630912	18.92864553	11.27085775	
Deaths	Georgia	Female	All Ages	Aortic aneurysm	Number	2019	37.56938628	45.74416668	29.87952701	165.58%
DALYs	Germany	Female	Age-standardized	Aortic aneurysm	Rate	1990	28.87642863	32.10249545	26.30325384	
DALYs	Germany	Female	Age-standardized	Aortic aneurysm	Rate	2019	24.9627852	27.59524324	22.49524983	-13.55%
DALYs	Germany	Female	All Ages	Aortic aneurysm	Number	1990	21971.35231	24622.93655	19792.76721	
DALYs	Germany	Female	All Ages	Aortic aneurysm	Number	2019	24597.56578	27321.86102	21773.92355	11.95%
Deaths	Germany	Female	Age-standardized	Aortic aneurysm	Rate	1990	1.538398866	1.725076971	1.370100575	
Deaths	Germany	Female	Age-standardized	Aortic aneurysm	Rate	2019	1.348689159	1.506422746	1.180853448	-12.33%
Deaths	Germany	Female	All Ages	Aortic aneurysm	Number	1990	1340.499843	1515.15553	1175.826444	
Deaths	Germany	Female	All Ages	Aortic aneurysm	Number	2019	1666.87406	1877.15437	1426.777735	24.35%
DALYs	Ghana	Female	Age-standardized	Aortic aneurysm	Rate	1990	50.44084951	63.88264302	38.79384182	
DALYs	Ghana	Female	Age-standardized	Aortic aneurysm	Rate	2019	23.74112274	31.22945441	17.1187732	-52.93%
DALYs	Ghana	Female	All Ages	Aortic aneurysm	Number	1990	1711.306393	2197.573422	1284.764185	
DALYs	Ghana	Female	All Ages	Aortic aneurysm	Number	2019	2289.867293	3078.034181	1628.753991	33.81%
Deaths	Ghana	Female	Age-standardized	Aortic aneurysm	Rate	1990	2.4681664	3.052006535	1.958073899	
Deaths	Ghana	Female	Age-standardized	Aortic aneurysm	Rate	2019	1.134989931	1.456464017	0.875200138	-54.01%
Deaths	Ghana	Female	All Ages	Aortic aneurysm	Number	1990	67.98349123	85.36017144	52.77190869	
Deaths	Ghana	Female	All Ages	Aortic aneurysm	Number	2019	90.75341147	117.5273183	67.32794256	33.49%
DALYs	Greece	Female	Age-standardized	Aortic aneurysm	Rate	1990	31.66207731	33.83138759	29.45332935	
DALYs	Greece	Female	Age-standardized	Aortic aneurysm	Rate	2019	39.91668688	44.03365256	35.66554012	26.07%
DALYs	Greece	Female	All Ages	Aortic aneurysm	Number	1990	2458.190283	2636.959303	2277.584505	
DALYs	Greece	Female	All Ages	Aortic aneurysm	Number	2019	4604.12242	5130.083642	4058.829338	87.30%
Deaths	Greece	Female	Age-standardized	Aortic aneurysm	Rate	1990	1.572365248	1.692603462	1.440701034	
Deaths	Greece	Female	Age-standardized	Aortic aneurysm	Rate	2019	2.029809976	2.264835873	1.780166604	29.09%
Deaths	Greece	Female	All Ages	Aortic aneurysm	Number	1990	128.3231457	138.7507723	117.5003061	
Deaths	Greece	Female	All Ages	Aortic aneurysm	Number	2019	303.4833132	343.3622847	262.5764395	136.50%
DALYs	Greenland	Female	Age-standardized	Aortic aneurysm	Rate	1990	50.1179463	60.99125272	41.46497241	
DALYs	Greenland	Female	Age-standardized	Aortic aneurysm	Rate	2019	30.81619057	38.70006002	24.12401265	-38.51%
DALYs	Greenland	Female	All Ages	Aortic aneurysm	Number	1990	7.631428902	9.278848981	6.287919343	
DALYs	Greenland	Female	All Ages	Aortic aneurysm	Number	2019	9.12084502	11.42928467	7.167665566	19.52%
Deaths	Greenland	Female	Age-standardized	Aortic aneurysm	Rate	1990	3.097853955	3.749649808	2.561694415	
Deaths	Greenland	Female	Age-standardized	Aortic aneurysm	Rate	2019	1.94265269	2.436885953	1.526907719	-37.29%
Deaths	Greenland	Female	All Ages	Aortic aneurysm	Number	1990	0.393743773	0.478991882	0.325280729	
Deaths	Greenland	Female	All Ages	Aortic aneurysm	Number	2019	0.529279249	0.6661303	0.414167844	34.42%
DALYs	Grenada	Female	Age-standardized	Aortic aneurysm	Rate	1990	43.52985862	52.88859224	37.06662184	
DALYs	Grenada	Female	Age-standardized	Aortic aneurysm	Rate	2019	41.58531139	47.24720374	35.95788508	-4.47%
DALYs	Grenada	Female	All Ages	Aortic aneurysm	Number	1990	18.83700146	22.86623946	16.04201989	
DALYs	Grenada	Female	All Ages	Aortic aneurysm	Number	2019	23.89605889	27.32206609	20.57019899	26.86%
Deaths	Grenada	Female	Age-standardized	Aortic aneurysm	Rate	1990	2.36991753	2.899399078	2.021182157	
Deaths	Grenada	Female	Age-standardized	Aortic aneurysm	Rate	2019	2.350718526	2.682320318	2.022242225	-0.81%
Deaths	Grenada	Female	All Ages	Aortic aneurysm	Number	1990	1.159868306	1.417587378	0.988065577	
Deaths	Grenada	Female	All Ages	Aortic aneurysm	Number	2019	1.321819236	1.513723313	1.136311963	13.96%
DALYs	Guam	Female	Age-standardized	Aortic aneurysm	Rate	1990	42.95402771	54.07318754	35.76435794	
DALYs	Guam	Female	Age-standardized	Aortic aneurysm	Rate	2019	19.60927371	24.30880965	16.01188914	-54.35%
DALYs	Guam	Female	All Ages	Aortic aneurysm	Number	1990	14.27979876	17.65884072	11.76821022	
DALYs	Guam	Female	All Ages	Aortic aneurysm	Number	2019	19.01716581	23.49547886	15.52246491	33.18%
Deaths	Guam	Female	Age-standardized	Aortic aneurysm	Rate	1990	2.673635358	3.439509815	2.184531217	

Deaths	Guam	Female	Age-standardized	Aortic aneurysm	Rate	2019	1.026991513	1.270866191	0.835942285	-61.59%
Deaths	Guam	Female	All Ages	Aortic aneurysm	Number	1990	0.649327742	0.814962789	0.539043046	
Deaths	Guam	Female	All Ages	Aortic aneurysm	Number	2019	1.04039446	1.284607371	0.844847893	60.23%
DALYs	Guatemala	Female	Age-standardized	Aortic aneurysm	Rate	1990	9.567802815	11.32826605	7.968880881	
DALYs	Guatemala	Female	Age-standardized	Aortic aneurysm	Rate	2019	10.85147426	13.83622817	8.464264673	13.42%
DALYs	Guatemala	Female	All Ages	Aortic aneurysm	Number	1990	187.1500378	225.1589224	153.887881	
DALYs	Guatemala	Female	All Ages	Aortic aneurysm	Number	2019	706.2380338	903.6586539	546.371455	277.36%
Deaths	Guatemala	Female	Age-standardized	Aortic aneurysm	Rate	1990	0.493162214	0.577058266	0.415170131	
Deaths	Guatemala	Female	Age-standardized	Aortic aneurysm	Rate	2019	0.580634282	0.72574752	0.457834369	17.74%
Deaths	Guatemala	Female	All Ages	Aortic aneurysm	Number	1990	7.099250228	8.400403671	5.905056613	
Deaths	Guatemala	Female	All Ages	Aortic aneurysm	Number	2019	33.22798893	41.87832496	26.05367705	368.05%
DALYs	Guinea	Female	Age-standardized	Aortic aneurysm	Rate	1990	38.20886217	57.42333678	23.34955299	
DALYs	Guinea	Female	Age-standardized	Aortic aneurysm	Rate	2019	19.18280121	29.45722146	13.92947673	-49.79%
DALYs	Guinea	Female	All Ages	Aortic aneurysm	Number	1990	638.2682842	964.5895317	388.1371055	
DALYs	Guinea	Female	All Ages	Aortic aneurysm	Number	2019	548.0630693	857.8236803	392.731023	-14.13%
Deaths	Guinea	Female	Age-standardized	Aortic aneurysm	Rate	1990	1.903452586	2.801357351	1.189422957	
Deaths	Guinea	Female	Age-standardized	Aortic aneurysm	Rate	2019	0.963580625	1.427508388	0.714800788	-49.38%
Deaths	Guinea	Female	All Ages	Aortic aneurysm	Number	1990	28.10969974	41.59284152	17.52171759	
Deaths	Guinea	Female	All Ages	Aortic aneurysm	Number	2019	23.82312692	35.82179401	17.53645357	-15.25%
DALYs	Guinea-Bissau	Female	Age-standardized	Aortic aneurysm	Rate	1990	34.44455297	61.39546471	19.93736101	
DALYs	Guinea-Bissau	Female	Age-standardized	Aortic aneurysm	Rate	2019	18.80476738	30.03086506	13.4627231	-45.41%
DALYs	Guinea-Bissau	Female	All Ages	Aortic aneurysm	Number	1990	73.98922777	136.7728489	42.20076283	
DALYs	Guinea-Bissau	Female	All Ages	Aortic aneurysm	Number	2019	79.10630347	134.6092436	55.34206031	6.92%
Deaths	Guinea-Bissau	Female	Age-standardized	Aortic aneurysm	Rate	1990	1.690413042	2.794250921	1.024754499	
Deaths	Guinea-Bissau	Female	Age-standardized	Aortic aneurysm	Rate	2019	0.941204984	1.421139352	0.688885512	-44.32%
Deaths	Guinea-Bissau	Female	All Ages	Aortic aneurysm	Number	1990	2.931274884	5.15711809	1.726164498	
Deaths	Guinea-Bissau	Female	All Ages	Aortic aneurysm	Number	2019	3.085826891	4.89075847	2.217557919	5.27%
DALYs	Guyana	Female	Age-standardized	Aortic aneurysm	Rate	1990	47.31296924	54.89850397	40.43205653	
DALYs	Guyana	Female	Age-standardized	Aortic aneurysm	Rate	2019	69.95614915	88.12090181	53.85270418	47.86%
DALYs	Guyana	Female	All Ages	Aortic aneurysm	Number	1990	95.33787082	111.0351679	81.01619597	
DALYs	Guyana	Female	All Ages	Aortic aneurysm	Number	2019	229.8681712	292.8653542	175.654687	141.11%
Deaths	Guyana	Female	Age-standardized	Aortic aneurysm	Rate	1990	2.419554359	2.788673023	2.095435738	
Deaths	Guyana	Female	Age-standardized	Aortic aneurysm	Rate	2019	3.612419352	4.457034621	2.846186531	49.30%
Deaths	Guyana	Female	All Ages	Aortic aneurysm	Number	1990	4.232714652	4.894986783	3.663828588	
Deaths	Guyana	Female	All Ages	Aortic aneurysm	Number	2019	10.49555738	13.07879143	8.190839832	147.96%
DALYs	Haiti	Female	Age-standardized	Aortic aneurysm	Rate	1990	46.48821737	73.10106137	32.68066607	
DALYs	Haiti	Female	Age-standardized	Aortic aneurysm	Rate	2019	39.70426138	70.32850683	24.62207655	-14.59%
DALYs	Haiti	Female	All Ages	Aortic aneurysm	Number	1990	731.5711374	1286.366977	507.132361	
DALYs	Haiti	Female	All Ages	Aortic aneurysm	Number	2019	1419.122605	2651.739218	850.6014291	93.98%
Deaths	Haiti	Female	Age-standardized	Aortic aneurysm	Rate	1990	2.640844347	3.872619279	1.824746642	
Deaths	Haiti	Female	Age-standardized	Aortic aneurysm	Rate	2019	2.163388731	3.539488044	1.368474428	-18.08%
Deaths	Haiti	Female	All Ages	Aortic aneurysm	Number	1990	34.3069249	51.70888864	24.3401578	
Deaths	Haiti	Female	All Ages	Aortic aneurysm	Number	2019	63.33703026	110.2199702	39.5555679	84.62%
DALYs	Honduras	Female	Age-standardized	Aortic aneurysm	Rate	1990	10.73870069	14.11348936	8.357813254	
DALYs	Honduras	Female	Age-standardized	Aortic aneurysm	Rate	2019	14.32712345	18.37545802	11.32984756	33.42%
DALYs	Honduras	Female	All Ages	Aortic aneurysm	Number	1990	116.2479076	154.7118404	89.74524239	
DALYs	Honduras	Female	All Ages	Aortic aneurysm	Number	2019	434.3983116	578.6617483	332.2548322	273.68%
Deaths	Honduras	Female	Age-standardized	Aortic aneurysm	Rate	1990	0.578069907	0.770660339	0.440380848	
Deaths	Honduras	Female	Age-standardized	Aortic aneurysm	Rate	2019	0.883174468	1.137635643	0.705045989	52.78%
Deaths	Honduras	Female	All Ages	Aortic aneurysm	Number	1990	5.33655394	7.016062385	4.131242549	
Deaths	Honduras	Female	All Ages	Aortic aneurysm	Number	2019	23.04803183	29.41766924	18.43553842	331.89%
DALYs	Hungary	Female	Age-standardized	Aortic aneurysm	Rate	1990	36.65785424	39.30824943	34.31063742	
DALYs	Hungary	Female	Age-standardized	Aortic aneurysm	Rate	2019	37.34141071	45.51399335	30.10921967	1.86%
DALYs	Hungary	Female	All Ages	Aortic aneurysm	Number	1990	3019.929907	3234.126341	2826.642578	
DALYs	Hungary	Female	All Ages	Aortic aneurysm	Number	2019	4058.093152	4913.809306	3250.354412	34.38%
Deaths	Hungary	Female	Age-standardized	Aortic aneurysm	Rate	1990	1.769508591	1.892147378	1.639150672	
Deaths	Hungary	Female	Age-standardized	Aortic aneurysm	Rate	2019	1.923654023	2.309385785	1.551820978	8.71%
Deaths	Hungary	Female	All Ages	Aortic aneurysm	Number	1990	151.9659163	162.841974	141.1039906	
Deaths	Hungary	Female	All Ages	Aortic aneurysm	Number	2019	243.7810082	293.3121147	196.4559474	60.42%
DALYs	Iceland	Female	Age-standardized	Aortic aneurysm	Rate	1990	46.40286068	51.8755108	41.58993136	
DALYs	Iceland	Female	Age-standardized	Aortic aneurysm	Rate	2019	30.17807414	34.44014922	25.5725832	-34.97%
DALYs	Iceland	Female	All Ages	Aortic aneurysm	Number	1990	72.18412722	80.89150124	64.33820882	
DALYs	Iceland	Female	All Ages	Aortic aneurysm	Number	2019	87.8264305	100.9759273	73.59384319	21.67%
Deaths	Iceland	Female	Age-standardized	Aortic aneurysm	Rate	1990	2.537318318	2.878337423	2.227207062	
Deaths	Iceland	Female	Age-standardized	Aortic aneurysm	Rate	2019	1.794804157	2.097736161	1.473065203	-29.26%
Deaths	Iceland	Female	All Ages	Aortic aneurysm	Number	1990	4.319498472	4.91538902	3.762878802	
Deaths	Iceland	Female	All Ages	Aortic aneurysm	Number	2019	6.309168854	7.439877102	5.013444463	46.06%
DALYs	India	Female	Age-standardized	Aortic aneurysm	Rate	1990	18.47548985	33.44980385	13.60082945	
DALYs	India	Female	Age-standardized	Aortic aneurysm	Rate	2019	20.1395765	26.07922498	14.94564959	9.01%
DALYs	India	Female	All Ages	Aortic aneurysm	Number	1990	37316.71272	71645.07149	27620.45273	
DALYs	India	Female	All Ages	Aortic aneurysm	Number	2019	114607.957	151034.0245	84319.85528	207.12%
Deaths	India	Female	Age-standardized	Aortic aneurysm	Rate	1990	1.025043159	1.730673984	0.749599305	
Deaths	India	Female	Age-standardized	Aortic aneurysm	Rate	2019	1.112776185	1.395870228	0.825455123	8.56%
Deaths	India	Female	All Ages	Aortic aneurysm	Number	1990	1565.909845	2846.703979	1150.469396	
Deaths	India	Female	All Ages	Aortic aneurysm	Number	2019	5595.454098	7090.445779	4170.248463	257.33%
DALYs	Indonesia	Female	Age-standardized	Aortic aneurysm	Rate	1990	16.11837508	26.41660159	12.039465	
DALYs	Indonesia	Female	Age-standardized	Aortic aneurysm	Rate	2019	18.90704621	30.41212031	13.70616321	17.30%
DALYs	Indonesia	Female	All Ages	Aortic aneurysm	Number	1990	8055.431853	14172.80645	5904.193907	
DALYs	Indonesia	Female	All Ages	Aortic aneurysm	Number	2019	19900.71389	32297.849	14270.89022	147.05%
Deaths	Indonesia	Female	Age-standardized	Aortic aneurysm	Rate	1990	0.886864637	1.36113667	0.66254765	
Deaths	Indonesia	Female	Age-standardized	Aortic aneurysm	Rate	2019	1.104743444	1.731347995	0.815860512	24.57%
Deaths	Indonesia	Female	All Ages	Aortic aneurysm	Number	1990	348.2021065	567.3438462	260.4919777	
Deaths	Indonesia	Female	All Ages	Aortic aneurysm	Number	2019	966.4594207	1530.879723	705.6741212	177.56%
DALYs	Iran (Islamic Republic of)	Female	Age-standardized	Aortic aneurysm	Rate	1990	12.04444482	14.10681589	10.64364557	
DALYs	Iran (Islamic Republic of)	Female	Age-standardized	Aortic aneurysm	Rate	2019	10.15521108	11.04684528	9.259536941	-15.69%
DALYs	Iran (Islamic Republic of)	Female	All Ages	Aortic aneurysm	Number	1990	1484.538753	1763.654614	1314.097803	
DALYs	Iran (Islamic Republic of)	Female	All Ages	Aortic aneurysm	Number	2019	3773.370158	4119.624726	3446.676072	154.18%
Deaths	Iran (Islamic Republic of)	Female	Age-standardized	Aortic aneurysm	Rate	1990	0.659669827	0.78331535	0.562596974	
Deaths	Iran (Islamic Republic of)	Female	Age-standardized	Aortic aneurysm	Rate	2019	0.522434331	0.571980406	0.46896987	-20.80%
Deaths	Iran (Islamic Republic of)	Female	All Ages	Aortic aneurysm	Number	1990	61.60418274	72.19816661	54.35613823	
Deaths	Iran (Islamic Republic of)	Female	All Ages	Aortic aneurysm	Number	2019	169.8964994	185.8598409	153.8765364	175.79%
DALYs	Iraq	Female	Age-standardized	Aortic aneurysm	Rate	1990	9.928045875	12.8265057	7.428937794	
DALYs	Iraq	Female	Age-standardized	Aortic aneurysm	Rate	2019	8.694817841	10.98612946	6.850073338	-12.42%
DALYs	Iraq	Female	All Ages	Aortic aneurysm	Number	1990	449.1674711	583.8925444	331.0981105	
DALYs	Iraq	Female	All Ages	Aortic aneurysm	Number	2019	1092.805434	1421.873245	842.9544766	143.30%
Deaths	Iraq	Female	Age-standardized	Aortic aneurysm	Rate	1990	0.482871226	0.60399936	0.364348154	
Deaths	Iraq	Female	Age-standardized	Aortic aneurysm	Rate	2019	0.449075849	0.548564256	0.364359475	-7.00%
Deaths	Iraq	Female	All Ages	Aortic aneurysm	Number	1990	18.1095324	22.95701551	13.63581219	
Deaths	Iraq	Female	All Ages	Aortic aneurysm	Number	2019	44.9014115	56.3469824	35.85959439	147.94%
DALYs	Ireland	Female	Age-standardized	Aortic aneurysm	Rate	1990	54.16911212	58.52199038	49.84030989	
DALYs	Ireland	Female	Age-standardized	Aortic aneurysm	Rate	2019	43.98926781	51.05684627	37.70152731	-18.79%

DALYs	Ireland	Female	All Ages	Aortic aneurysm	Number	1990	1306.403527	1421.500115	1200.764164	
DALYs	Ireland	Female	All Ages	Aortic aneurysm	Number	2019	1823.281364	2127.278453	1558.055986	39.56%
Deaths	Ireland	Female	Age-standardized	Aortic aneurysm	Rate	1990	3.299057075	3.577388303	2.999868984	
Deaths	Ireland	Female	Age-standardized	Aortic aneurysm	Rate	2019	3.016350292	3.544623831	2.545810177	-8.57%
Deaths	Ireland	Female	All Ages	Aortic aneurysm	Number	1990	80.98500068	88.08598781	73.88837595	
Deaths	Ireland	Female	All Ages	Aortic aneurysm	Number	2019	134.0874638	157.7624758	112.1992288	65.57%
DALYs	Israel	Female	Age-standardized	Aortic aneurysm	Rate	1990	21.34924728	23.63003302	19.08415758	
DALYs	Israel	Female	Age-standardized	Aortic aneurysm	Rate	2019	14.37832653	16.15859785	12.44670927	-32.65%
DALYs	Israel	Female	All Ages	Aortic aneurysm	Number	1990	563.451138	629.9264779	499.3091048	
DALYs	Israel	Female	All Ages	Aortic aneurysm	Number	2019	946.8955029	1073.877148	809.2613484	68.05%
Deaths	Israel	Female	Age-standardized	Aortic aneurysm	Rate	1990	1.373663613	1.544940216	1.201889831	
Deaths	Israel	Female	Age-standardized	Aortic aneurysm	Rate	2019	0.969197752	1.113964157	0.812881172	-29.44%
Deaths	Israel	Female	All Ages	Aortic aneurysm	Number	1990	34.56334301	39.19479776	30.24969767	
Deaths	Israel	Female	All Ages	Aortic aneurysm	Number	2019	70.50536717	81.23755753	59.08476529	103.99%
DALYs	Italy	Female	Age-standardized	Aortic aneurysm	Rate	1990	21.33911326	22.74423749	20.2557137	
DALYs	Italy	Female	Age-standardized	Aortic aneurysm	Rate	2019	21.62794645	23.34318449	19.36453566	1.35%
DALYs	Italy	Female	All Ages	Aortic aneurysm	Number	1990	10818.40931	11591.85999	10232.89538	
DALYs	Italy	Female	All Ages	Aortic aneurysm	Number	2019	17517.36762	19014.74709	15148.35786	61.92%
Deaths	Italy	Female	Age-standardized	Aortic aneurysm	Rate	1990	1.181510783	1.285857115	1.098324765	
Deaths	Italy	Female	Age-standardized	Aortic aneurysm	Rate	2019	1.283528164	1.395633402	1.095115063	8.63%
Deaths	Italy	Female	All Ages	Aortic aneurysm	Number	1990	647.8108165	704.6516339	602.4803952	
Deaths	Italy	Female	All Ages	Aortic aneurysm	Number	2019	1318.713025	1455.067462	1103.5497	103.56%
DALYs	Jamaica	Female	Age-standardized	Aortic aneurysm	Rate	1990	25.64631707	29.25716654	22.71731345	
DALYs	Jamaica	Female	Age-standardized	Aortic aneurysm	Rate	2019	29.26791236	36.43943496	22.71351471	14.12%
DALYs	Jamaica	Female	All Ages	Aortic aneurysm	Number	1990	246.7326124	281.8385716	217.9717848	
DALYs	Jamaica	Female	All Ages	Aortic aneurysm	Number	2019	465.0559767	576.7953805	361.9213514	88.49%
Deaths	Jamaica	Female	Age-standardized	Aortic aneurysm	Rate	1990	1.340893329	1.546719307	1.165139978	
Deaths	Jamaica	Female	Age-standardized	Aortic aneurysm	Rate	2019	1.531908121	1.89807467	1.207155952	14.25%
Deaths	Jamaica	Female	All Ages	Aortic aneurysm	Number	1990	13.62859646	15.77561168	11.81994764	
Deaths	Jamaica	Female	All Ages	Aortic aneurysm	Number	2019	27.1528045	33.34087896	21.46272968	99.23%
DALYs	Japan	Female	Age-standardized	Aortic aneurysm	Rate	1990	27.20912642	28.42112634	25.29333556	
DALYs	Japan	Female	Age-standardized	Aortic aneurysm	Rate	2019	49.66017556	54.63300698	41.95814965	82.51%
DALYs	Japan	Female	All Ages	Aortic aneurysm	Number	1990	26468.16701	27677.61033	24581.50079	
DALYs	Japan	Female	All Ages	Aortic aneurysm	Number	2019	113135.7206	127806.7358	89734.36081	327.44%
Deaths	Japan	Female	Age-standardized	Aortic aneurysm	Rate	1990	1.67647285	1.776918774	1.512973156	
Deaths	Japan	Female	Age-standardized	Aortic aneurysm	Rate	2019	3.322946485	3.756784979	2.63406753	98.21%
Deaths	Japan	Female	All Ages	Aortic aneurysm	Number	1990	1628.038482	1721.405246	1474.363238	
Deaths	Japan	Female	All Ages	Aortic aneurysm	Number	2019	10078.925	11647.61468	7466.262973	519.08%
DALYs	Jordan	Female	Age-standardized	Aortic aneurysm	Rate	1990	19.75558287	24.57051963	15.95007714	
DALYs	Jordan	Female	Age-standardized	Aortic aneurysm	Rate	2019	13.93195076	17.36564639	10.99391541	-29.48%
DALYs	Jordan	Female	All Ages	Aortic aneurysm	Number	1990	154.0979804	202.656997	121.1919094	
DALYs	Jordan	Female	All Ages	Aortic aneurysm	Number	2019	471.822162	597.4148521	367.0186768	206.18%
Deaths	Jordan	Female	Age-standardized	Aortic aneurysm	Rate	1990	0.980869283	1.204564875	0.800215486	
Deaths	Jordan	Female	Age-standardized	Aortic aneurysm	Rate	2019	0.712511074	0.869919055	0.564957025	-27.36%
Deaths	Jordan	Female	All Ages	Aortic aneurysm	Number	1990	5.478064387	6.802126899	4.441732515	
Deaths	Jordan	Female	All Ages	Aortic aneurysm	Number	2019	17.84303539	22.19053144	14.15541313	225.72%
DALYs	Kazakhstan	Female	Age-standardized	Aortic aneurysm	Rate	1990	20.66574437	23.74110507	16.854	
DALYs	Kazakhstan	Female	Age-standardized	Aortic aneurysm	Rate	2019	30.08430425	36.18460275	24.71156284	45.58%
DALYs	Kazakhstan	Female	All Ages	Aortic aneurysm	Number	1990	1641.375167	1892.134349	1342.379068	
DALYs	Kazakhstan	Female	All Ages	Aortic aneurysm	Number	2019	3135.052573	3774.322716	2565.051587	91.00%
Deaths	Kazakhstan	Female	Age-standardized	Aortic aneurysm	Rate	1990	0.867747856	1.002435	0.684931651	
Deaths	Kazakhstan	Female	Age-standardized	Aortic aneurysm	Rate	2019	1.274112724	1.500632406	1.059910621	46.83%
Deaths	Kazakhstan	Female	All Ages	Aortic aneurysm	Number	1990	66.8240463	77.26730237	53.03267424	
Deaths	Kazakhstan	Female	All Ages	Aortic aneurysm	Number	2019	126.5269021	150.0828152	105.0475461	89.34%
DALYs	Kenya	Female	Age-standardized	Aortic aneurysm	Rate	1990	36.83713202	48.59528355	23.48739085	
DALYs	Kenya	Female	Age-standardized	Aortic aneurysm	Rate	2019	33.08976328	44.58292701	22.97374054	-10.17%
DALYs	Kenya	Female	All Ages	Aortic aneurysm	Number	1990	1557.55098	2072.00651	988.4057738	
DALYs	Kenya	Female	All Ages	Aortic aneurysm	Number	2019	3787.980329	5182.987264	2683.198824	143.20%
Deaths	Kenya	Female	Age-standardized	Aortic aneurysm	Rate	1990	2.025774323	2.645968155	1.302545147	
Deaths	Kenya	Female	Age-standardized	Aortic aneurysm	Rate	2019	1.871812927	2.491050956	1.225379044	-7.60%
Deaths	Kenya	Female	All Ages	Aortic aneurysm	Number	1990	69.9503767	91.55327796	44.70746986	
Deaths	Kenya	Female	All Ages	Aortic aneurysm	Number	2019	173.4538534	231.5499554	118.1537286	147.97%
DALYs	Kiribati	Female	Age-standardized	Aortic aneurysm	Rate	1990	18.06801422	22.11144372	13.96036415	
DALYs	Kiribati	Female	Age-standardized	Aortic aneurysm	Rate	2019	16.63747396	21.17411311	12.51819646	-7.92%
DALYs	Kiribati	Female	All Ages	Aortic aneurysm	Number	1990	3.718431967	4.57808425	2.857627	
DALYs	Kiribati	Female	All Ages	Aortic aneurysm	Number	2019	6.499894829	8.403346703	4.80671249	74.80%
Deaths	Kiribati	Female	Age-standardized	Aortic aneurysm	Rate	1990	0.87337657	1.086039053	0.666558778	
Deaths	Kiribati	Female	Age-standardized	Aortic aneurysm	Rate	2019	0.835002185	1.052511861	0.64710503	-4.39%
Deaths	Kiribati	Female	All Ages	Aortic aneurysm	Number	1990	0.140689761	0.172349606	0.107650698	
Deaths	Kiribati	Female	All Ages	Aortic aneurysm	Number	2019	0.242223752	0.309796988	0.18323436	72.17%
DALYs	Kuwait	Female	Age-standardized	Aortic aneurysm	Rate	1990	12.32322467	14.98457716	10.64042289	
DALYs	Kuwait	Female	Age-standardized	Aortic aneurysm	Rate	2019	12.15134412	15.41095149	9.302862331	-1.39%
DALYs	Kuwait	Female	All Ages	Aortic aneurysm	Number	1990	37.77110075	43.40615507	33.4608833	
DALYs	Kuwait	Female	All Ages	Aortic aneurysm	Number	2019	149.2271448	191.9629931	110.8906912	295.08%
Deaths	Kuwait	Female	Age-standardized	Aortic aneurysm	Rate	1990	0.615294609	0.757495783	0.52239419	
Deaths	Kuwait	Female	Age-standardized	Aortic aneurysm	Rate	2019	0.667124492	0.845393513	0.510426486	8.42%
Deaths	Kuwait	Female	All Ages	Aortic aneurysm	Number	1990	1.365766408	1.613373961	1.187771655	
Deaths	Kuwait	Female	All Ages	Aortic aneurysm	Number	2019	6.017456274	7.651522457	4.643416536	340.59%
DALYs	Kyrgyzstan	Female	Age-standardized	Aortic aneurysm	Rate	1990	11.06399434	13.05276304	9.697515306	
DALYs	Kyrgyzstan	Female	Age-standardized	Aortic aneurysm	Rate	2019	11.7135265	13.70883859	9.980358633	5.87%
DALYs	Kyrgyzstan	Female	All Ages	Aortic aneurysm	Number	1990	202.00423	238.653167	177.0054899	
DALYs	Kyrgyzstan	Female	All Ages	Aortic aneurysm	Number	2019	306.6416395	359.967609	259.3410051	51.80%
Deaths	Kyrgyzstan	Female	Age-standardized	Aortic aneurysm	Rate	1990	0.481393598	0.56932905	0.420616093	
Deaths	Kyrgyzstan	Female	Age-standardized	Aortic aneurysm	Rate	2019	0.615869926	0.713356704	0.520111748	27.93%
Deaths	Kyrgyzstan	Female	All Ages	Aortic aneurysm	Number	1990	8.919582232	10.55190679	7.777620254	
Deaths	Kyrgyzstan	Female	All Ages	Aortic aneurysm	Number	2019	14.43500676	16.76296444	12.23235394	61.84%
DALYs	Lao People's Democratic Republic	Female	Age-standardized	Aortic aneurysm	Rate	1990	13.0492403	28.72829406	8.434232791	
DALYs	Lao People's Democratic Republic	Female	Age-standardized	Aortic aneurysm	Rate	2019	18.00006408	25.92033298	13.32115639	37.94%
DALYs	Lao People's Democratic Republic	Female	All Ages	Aortic aneurysm	Number	1990	131.5510295	313.843663	84.6783598	
DALYs	Lao People's Democratic Republic	Female	All Ages	Aortic aneurysm	Number	2019	400.5318209	589.166808	286.9325547	204.47%
Deaths	Lao People's Democratic Republic	Female	Age-standardized	Aortic aneurysm	Rate	1990	0.751357386	1.455942204	0.483380669	
Deaths	Lao People's Democratic Republic	Female	Age-standardized	Aortic aneurysm	Rate	2019	1.003693284	1.404993788	0.763873649	33.58%
Deaths	Lao People's Democratic Republic	Female	All Ages	Aortic aneurysm	Number	1990	6.070758235	12.86201348	3.921100826	
Deaths	Lao People's Democratic Republic	Female	All Ages	Aortic aneurysm	Number	2019	18.25352008	25.83750977	13.7234423	200.68%
DALYs	Latvia	Female	Age-standardized	Aortic aneurysm	Rate	1990	22.51022528	25.56446673	20.09651634	
DALYs	Latvia	Female	Age-standardized	Aortic aneurysm	Rate	2019	22.57135308	29.82854328	16.85134255	0.27%
DALYs	Latvia	Female	All Ages	Aortic aneurysm	Number	1990	491.6786052	559.5218407	437.3488835	
DALYs	Latvia	Female	All Ages	Aortic aneurysm	Number	2019	530.1430161	682.5615693	404.0556182	7.82%
Deaths	Latvia	Female	Age-standardized	Aortic aneurysm	Rate	1990	1.053385766	1.199056673	0.940410647	
Deaths	Latvia	Female	Age-standardized	Aortic aneurysm	Rate	2019	1.132407998	1.456751507	0.870274531	7.50%
Deaths	Latvia	Female	All Ages	Aortic aneurysm	Number	1990	24.75670404	28.20920478	21.91367564	

Deaths	Latvia	Female	All Ages	Aortic aneurysm	Number	2019	32.90207613	42.34031203	25.3534361	32.90%
DALYs	Lebanon	Female	Age-standardized	Aortic aneurysm	Rate	1990	14.50695476	19.51756445	11.10143255	
DALYs	Lebanon	Female	Age-standardized	Aortic aneurysm	Rate	2019	10.88695789	14.08080926	7.076326446	-24.95%
DALYs	Lebanon	Female	All Ages	Aortic aneurysm	Number	1990	165.3727072	223.9410019	125.7283034	
DALYs	Lebanon	Female	All Ages	Aortic aneurysm	Number	2019	310.282382	401.020521	201.8335129	87.63%
Deaths	Lebanon	Female	Age-standardized	Aortic aneurysm	Rate	1990	0.758548559	1.001283571	0.581393965	
Deaths	Lebanon	Female	Age-standardized	Aortic aneurysm	Rate	2019	0.572899405	0.726934195	0.37763214	-24.47%
Deaths	Lebanon	Female	All Ages	Aortic aneurysm	Number	1990	7.436975905	9.895048361	5.718389493	
Deaths	Lebanon	Female	All Ages	Aortic aneurysm	Number	2019	16.31251576	20.66477396	10.69850444	119.34%
DALYs	Lesotho	Female	Age-standardized	Aortic aneurysm	Rate	1990	29.74935381	41.6347241	20.45769639	
DALYs	Lesotho	Female	Age-standardized	Aortic aneurysm	Rate	2019	23.87562266	38.52499027	14.80399438	-19.74%
DALYs	Lesotho	Female	All Ages	Aortic aneurysm	Number	1990	166.4994829	233.3679155	112.5891687	
DALYs	Lesotho	Female	All Ages	Aortic aneurysm	Number	2019	173.6195887	288.1914376	103.7935003	4.28%
Deaths	Lesotho	Female	Age-standardized	Aortic aneurysm	Rate	1990	1.576811162	2.18395041	1.075608095	
Deaths	Lesotho	Female	Age-standardized	Aortic aneurysm	Rate	2019	1.311981379	2.0172604	0.85915158	-16.80%
Deaths	Lesotho	Female	All Ages	Aortic aneurysm	Number	1990	7.973393793	11.00035147	5.459138349	
Deaths	Lesotho	Female	All Ages	Aortic aneurysm	Number	2019	7.925513891	12.57970117	5.07035142	-0.60%
DALYs	Liberia	Female	Age-standardized	Aortic aneurysm	Rate	1990	32.00953678	48.33144274	22.60420738	
DALYs	Liberia	Female	Age-standardized	Aortic aneurysm	Rate	2019	15.69486092	23.87912809	11.24169886	-50.97%
DALYs	Liberia	Female	All Ages	Aortic aneurysm	Number	1990	164.1544637	252.2326563	115.2461973	
DALYs	Liberia	Female	All Ages	Aortic aneurysm	Number	2019	166.480605	259.8459791	115.8557855	1.42%
Deaths	Liberia	Female	Age-standardized	Aortic aneurysm	Rate	1990	1.633134307	2.396123082	1.181414966	
Deaths	Liberia	Female	Age-standardized	Aortic aneurysm	Rate	2019	0.818878569	1.219323114	0.612339229	-49.86%
Deaths	Liberia	Female	All Ages	Aortic aneurysm	Number	1990	7.261754675	10.82744045	5.182358299	
Deaths	Liberia	Female	All Ages	Aortic aneurysm	Number	2019	6.886604685	10.45327221	5.013644821	-5.17%
DALYs	Libya	Female	Age-standardized	Aortic aneurysm	Rate	1990	9.513982467	13.01739931	7.0529958	
DALYs	Libya	Female	Age-standardized	Aortic aneurysm	Rate	2019	9.527058361	16.77624797	6.469758191	0.14%
DALYs	Libya	Female	All Ages	Aortic aneurysm	Number	1990	95.12238799	130.7238143	69.62908354	
DALYs	Libya	Female	All Ages	Aortic aneurysm	Number	2019	259.9930341	458.1752976	171.3834877	173.32%
Deaths	Libya	Female	Age-standardized	Aortic aneurysm	Rate	1990	0.459719806	0.628741295	0.344119086	
Deaths	Libya	Female	Age-standardized	Aortic aneurysm	Rate	2019	0.461634821	0.82533882	0.3067767	0.42%
Deaths	Libya	Female	All Ages	Aortic aneurysm	Number	1990	3.833219029	5.208750379	2.847436274	
Deaths	Libya	Female	All Ages	Aortic aneurysm	Number	2019	10.63537833	18.90466425	7.048884503	177.45%
DALYs	Lithuania	Female	Age-standardized	Aortic aneurysm	Rate	1990	24.37173321	28.01862925	21.28324051	
DALYs	Lithuania	Female	Age-standardized	Aortic aneurysm	Rate	2019	30.59265607	38.19663371	24.23425586	25.53%
DALYs	Lithuania	Female	All Ages	Aortic aneurysm	Number	1990	644.9867843	743.8034178	563.6909886	
DALYs	Lithuania	Female	All Ages	Aortic aneurysm	Number	2019	986.361401	1223.283357	791.9369817	52.93%
Deaths	Lithuania	Female	Age-standardized	Aortic aneurysm	Rate	1990	1.065275999	1.228021253	0.928733479	
Deaths	Lithuania	Female	Age-standardized	Aortic aneurysm	Rate	2019	1.434174812	1.763600097	1.15851905	34.63%
Deaths	Lithuania	Female	All Ages	Aortic aneurysm	Number	1990	29.78937627	34.44078998	25.87774116	
Deaths	Lithuania	Female	All Ages	Aortic aneurysm	Number	2019	57.08256188	69.10344248	46.07738058	91.62%
DALYs	Luxembourg	Female	Age-standardized	Aortic aneurysm	Rate	1990	30.43421442	33.73414122	27.34099702	
DALYs	Luxembourg	Female	Age-standardized	Aortic aneurysm	Rate	2019	22.57737101	27.22514715	18.32270887	-25.82%
DALYs	Luxembourg	Female	All Ages	Aortic aneurysm	Number	1990	97.36930327	108.9935448	86.58415676	
DALYs	Luxembourg	Female	All Ages	Aortic aneurysm	Number	2019	123.9263662	150.9483816	99.86549154	27.27%
Deaths	Luxembourg	Female	Age-standardized	Aortic aneurysm	Rate	1990	1.692742772	1.886697157	1.496017401	
Deaths	Luxembourg	Female	Age-standardized	Aortic aneurysm	Rate	2019	1.359050409	1.65267731	1.08835461	-19.71%
Deaths	Luxembourg	Female	All Ages	Aortic aneurysm	Number	1990	5.845525124	6.568623247	5.129301092	
Deaths	Luxembourg	Female	All Ages	Aortic aneurysm	Number	2019	8.787193482	10.69913307	6.898239108	50.32%
DALYs	Madagascar	Female	Age-standardized	Aortic aneurysm	Rate	1990	68.99813696	91.37351935	43.63420647	
DALYs	Madagascar	Female	Age-standardized	Aortic aneurysm	Rate	2019	48.97904408	67.13083833	33.46302386	-29.01%
DALYs	Madagascar	Female	All Ages	Aortic aneurysm	Number	1990	1851.658608	2605.799365	1150.394632	
DALYs	Madagascar	Female	All Ages	Aortic aneurysm	Number	2019	2989.022214	4150.194922	1995.114592	61.42%
Deaths	Madagascar	Female	Age-standardized	Aortic aneurysm	Rate	1990	3.460220026	4.491607488	2.235058963	
Deaths	Madagascar	Female	Age-standardized	Aortic aneurysm	Rate	2019	2.43701546	3.328231879	1.662273485	-29.57%
Deaths	Madagascar	Female	All Ages	Aortic aneurysm	Number	1990	73.45058016	95.50476178	46.58921854	
Deaths	Madagascar	Female	All Ages	Aortic aneurysm	Number	2019	110.1843876	150.6192638	75.40516823	50.01%
DALYs	Malawi	Female	Age-standardized	Aortic aneurysm	Rate	1990	37.59836735	51.20026947	18.30288653	
DALYs	Malawi	Female	Age-standardized	Aortic aneurysm	Rate	2019	29.02834611	38.51390554	18.88165007	-22.79%
DALYs	Malawi	Female	All Ages	Aortic aneurysm	Number	1990	754.8384734	1081.469644	362.6480484	
DALYs	Malawi	Female	All Ages	Aortic aneurysm	Number	2019	1189.38896	1594.183839	769.6903943	57.57%
Deaths	Malawi	Female	Age-standardized	Aortic aneurysm	Rate	1990	2.051640264	2.690014135	1.006077766	
Deaths	Malawi	Female	Age-standardized	Aortic aneurysm	Rate	2019	1.606868526	2.13730161	1.045138281	-21.68%
Deaths	Malawi	Female	All Ages	Aortic aneurysm	Number	1990	32.23201068	43.72545546	15.81169573	
Deaths	Malawi	Female	All Ages	Aortic aneurysm	Number	2019	55.49986525	73.47560122	36.16751417	72.19%
DALYs	Malaysia	Female	Age-standardized	Aortic aneurysm	Rate	1990	42.6128668	50.85752871	36.12308563	
DALYs	Malaysia	Female	Age-standardized	Aortic aneurysm	Rate	2019	35.61590756	45.40663383	27.32436087	-16.42%
DALYs	Malaysia	Female	All Ages	Aortic aneurysm	Number	1990	1890.460012	2286.987238	1610.330264	
DALYs	Malaysia	Female	All Ages	Aortic aneurysm	Number	2019	4357.730892	5564.354618	3306.082822	130.51%
Deaths	Malaysia	Female	Age-standardized	Aortic aneurysm	Rate	1990	2.54143905	2.972327802	2.125193903	
Deaths	Malaysia	Female	Age-standardized	Aortic aneurysm	Rate	2019	2.246255869	2.822342824	1.73556106	-11.61%
Deaths	Malaysia	Female	All Ages	Aortic aneurysm	Number	1990	97.7930766	114.6782275	82.70639113	
Deaths	Malaysia	Female	All Ages	Aortic aneurysm	Number	2019	240.8094334	306.3052463	184.5398564	146.24%
DALYs	Maldives	Female	Age-standardized	Aortic aneurysm	Rate	1990	20.99906844	44.58797149	15.27315812	
DALYs	Maldives	Female	Age-standardized	Aortic aneurysm	Rate	2019	21.30790784	26.25156516	17.11493087	1.47%
DALYs	Maldives	Female	All Ages	Aortic aneurysm	Number	1990	6.200043999	14.74565775	4.364238036	
DALYs	Maldives	Female	All Ages	Aortic aneurysm	Number	2019	25.84131653	31.86732978	20.8005389	316.79%
Deaths	Maldives	Female	Age-standardized	Aortic aneurysm	Rate	1990	1.37144804	2.678455312	1.002373112	
Deaths	Maldives	Female	Age-standardized	Aortic aneurysm	Rate	2019	1.532685063	1.907096948	1.211586948	11.76%
Deaths	Maldives	Female	All Ages	Aortic aneurysm	Number	1990	0.265398397	0.581048535	0.191415119	
Deaths	Maldives	Female	All Ages	Aortic aneurysm	Number	2019	1.662366042	2.062643007	1.326835797	526.37%
DALYs	Mali	Female	Age-standardized	Aortic aneurysm	Rate	1990	36.04295633	59.52210937	18.66522601	
DALYs	Mali	Female	Age-standardized	Aortic aneurysm	Rate	2019	19.16270698	29.49581405	11.90211179	-46.83%
DALYs	Mali	Female	All Ages	Aortic aneurysm	Number	1990	765.882286	1303.329687	392.7630861	
DALYs	Mali	Female	All Ages	Aortic aneurysm	Number	2019	857.0378931	1347.471215	519.0211996	11.90%
Deaths	Mali	Female	Age-standardized	Aortic aneurysm	Rate	1990	1.778333121	2.759147124	0.953263623	
Deaths	Mali	Female	Age-standardized	Aortic aneurysm	Rate	2019	0.954362803	1.427040995	0.600391489	-46.33%
Deaths	Mali	Female	All Ages	Aortic aneurysm	Number	1990	31.14772719	50.41966476	16.29269152	
Deaths	Mali	Female	All Ages	Aortic aneurysm	Number	2019	34.19191885	52.20381288	21.31873101	9.77%
DALYs	Malta	Female	Age-standardized	Aortic aneurysm	Rate	1990	14.19294898	15.7350997	12.68280835	
DALYs	Malta	Female	Age-standardized	Aortic aneurysm	Rate	2019	10.99092687	12.95084215	9.011156797	-22.56%
DALYs	Malta	Female	All Ages	Aortic aneurysm	Number	1990	33.6884891	37.45221539	30.00708727	
DALYs	Malta	Female	All Ages	Aortic aneurysm	Number	2019	53.3494038	63.44256238	42.91239804	58.36%
Deaths	Malta	Female	Age-standardized	Aortic aneurysm	Rate	1990	0.778020609	0.876921553	0.688099575	
Deaths	Malta	Female	Age-standardized	Aortic aneurysm	Rate	2019	0.620239781	0.736808037	0.503162829	-20.28%
Deaths	Malta	Female	All Ages	Aortic aneurysm	Number	1990	1.854391818	2.10427096	1.633822755	
Deaths	Malta	Female	All Ages	Aortic aneurysm	Number	2019	3.543873381	4.223648867	2.879197551	91.11%
DALYs	Marshall Islands	Female	Age-standardized	Aortic aneurysm	Rate	1990	64.86042283	101.6138418	42.89434133	
DALYs	Marshall Islands	Female	Age-standardized	Aortic aneurysm	Rate	2019	59.82939917	95.8471257	39.74562052	-7.76%
DALYs	Marshall Islands	Female	All Ages	Aortic aneurysm	Number	1990	5.085488525	8.17932349	3.353497368	
DALYs	Marshall Islands	Female	All Ages	Aortic aneurysm	Number	2019	9.535755101	16.07878581	5.979131734	87.51%

Deaths	Marshall Islands	Female	Age-standardized	Aortic aneurysm	Rate	1990	3.578280594	5.337449603	2.377834412	
Deaths	Marshall Islands	Female	Age-standardized	Aortic aneurysm	Rate	2019	3.255446181	4.890461848	2.230049251	-9.02%
Deaths	Marshall Islands	Female	All Ages	Aortic aneurysm	Number	1990	0.226959417	0.355213806	0.14986133	
Deaths	Marshall Islands	Female	All Ages	Aortic aneurysm	Number	2019	0.364689708	0.588473467	0.237817187	60.68%
DALYs	Mauritania	Female	Age-standardized	Aortic aneurysm	Rate	1990	38.7758105	53.34866505	28.92272919	
DALYs	Mauritania	Female	Age-standardized	Aortic aneurysm	Rate	2019	16.82834702	22.42834491	12.78439846	-56.60%
DALYs	Mauritania	Female	All Ages	Aortic aneurysm	Number	1990	206.6510431	288.806586	154.35321	
DALYs	Mauritania	Female	All Ages	Aortic aneurysm	Number	2019	178.0911571	239.8092451	131.3184447	-13.82%
Deaths	Mauritania	Female	Age-standardized	Aortic aneurysm	Rate	1990	1.965016584	2.591528061	1.486978458	
Deaths	Mauritania	Female	Age-standardized	Aortic aneurysm	Rate	2019	0.883585797	1.140984424	0.689046495	-55.03%
Deaths	Mauritania	Female	All Ages	Aortic aneurysm	Number	1990	9.384810081	12.4986503	7.024566945	
Deaths	Mauritania	Female	All Ages	Aortic aneurysm	Number	2019	7.985039737	10.49699298	6.173745787	-14.92%
DALYs	Mauritius	Female	Age-standardized	Aortic aneurysm	Rate	1990	14.66621234	16.29475769	13.17963865	
DALYs	Mauritius	Female	Age-standardized	Aortic aneurysm	Rate	2019	12.33051736	15.22314859	9.753920283	-15.93%
DALYs	Mauritius	Female	All Ages	Aortic aneurysm	Number	1990	63.24123202	70.72489482	56.56149384	
DALYs	Mauritius	Female	All Ages	Aortic aneurysm	Number	2019	108.8522581	134.1237292	85.82119912	72.12%
Deaths	Mauritius	Female	Age-standardized	Aortic aneurysm	Rate	1990	0.711666103	0.798331658	0.632772844	
Deaths	Mauritius	Female	Age-standardized	Aortic aneurysm	Rate	2019	0.620894692	0.754267329	0.491317727	-12.75%
Deaths	Mauritius	Female	All Ages	Aortic aneurysm	Number	1990	2.612299968	2.909966898	2.330353453	
Deaths	Mauritius	Female	All Ages	Aortic aneurysm	Number	2019	5.59269575	6.778006123	4.434362393	114.09%
DALYs	Mexico	Female	Age-standardized	Aortic aneurysm	Rate	1990	9.422155925	9.774149995	8.945206675	
DALYs	Mexico	Female	Age-standardized	Aortic aneurysm	Rate	2019	8.985249549	10.82586439	7.395071346	-4.64%
DALYs	Mexico	Female	All Ages	Aortic aneurysm	Number	1990	2292.12014	2367.092234	2190.022026	
DALYs	Mexico	Female	All Ages	Aortic aneurysm	Number	2019	5596.375801	6761.561327	4586.83852	144.16%
Deaths	Mexico	Female	Age-standardized	Aortic aneurysm	Rate	1990	0.490021966	0.515910987	0.454290698	
Deaths	Mexico	Female	Age-standardized	Aortic aneurysm	Rate	2019	0.484393672	0.576437325	0.397151808	-1.15%
Deaths	Mexico	Female	All Ages	Aortic aneurysm	Number	1990	96.94377575	101.4088823	91.10061582	
Deaths	Mexico	Female	All Ages	Aortic aneurysm	Number	2019	285.5558458	340.2676269	234.7397375	194.56%
DALYs	Micronesia (Federated States of)	Female	Age-standardized	Aortic aneurysm	Rate	1990	73.43099634	119.8798944	49.11349802	
DALYs	Micronesia (Federated States of)	Female	Age-standardized	Aortic aneurysm	Rate	2019	60.31062369	102.5075629	37.62075145	-17.87%
DALYs	Micronesia (Federated States of)	Female	All Ages	Aortic aneurysm	Number	1990	15.83518755	27.09723888	10.44802386	
DALYs	Micronesia (Federated States of)	Female	All Ages	Aortic aneurysm	Number	2019	20.63267954	36.59631866	12.10312493	30.30%
Deaths	Micronesia (Federated States of)	Female	Age-standardized	Aortic aneurysm	Rate	1990	4.137488566	6.264514278	2.798344705	
Deaths	Micronesia (Federated States of)	Female	Age-standardized	Aortic aneurysm	Rate	2019	3.382746163	5.277809941	2.208594924	-18.24%
Deaths	Micronesia (Federated States of)	Female	All Ages	Aortic aneurysm	Number	1990	0.7198278	1.16006014	0.481736657	
Deaths	Micronesia (Federated States of)	Female	All Ages	Aortic aneurysm	Number	2019	0.904290081	1.504133424	0.576652112	25.63%
DALYs	Monaco	Female	Age-standardized	Aortic aneurysm	Rate	1990	78.36101998	99.88194311	57.03419511	
DALYs	Monaco	Female	Age-standardized	Aortic aneurysm	Rate	2019	66.15897135	84.42204596	48.07818578	-15.57%
DALYs	Monaco	Female	All Ages	Aortic aneurysm	Number	1990	31.4771861	40.25659569	22.92322446	
DALYs	Monaco	Female	All Ages	Aortic aneurysm	Number	2019	33.74609196	42.89160135	24.3565642	7.21%
Deaths	Monaco	Female	Age-standardized	Aortic aneurysm	Rate	1990	4.392864922	5.535567761	3.232681375	
Deaths	Monaco	Female	Age-standardized	Aortic aneurysm	Rate	2019	3.928243233	4.970221366	2.866121582	-10.58%
Deaths	Monaco	Female	All Ages	Aortic aneurysm	Number	1990	2.169691584	2.738737535	1.588727529	
Deaths	Monaco	Female	All Ages	Aortic aneurysm	Number	2019	2.499753891	3.144792099	1.859882405	15.21%
DALYs	Mongolia	Female	Age-standardized	Aortic aneurysm	Rate	1990	19.14713183	24.2149584	14.92974566	
DALYs	Mongolia	Female	Age-standardized	Aortic aneurysm	Rate	2019	17.51294329	22.35210124	13.34361995	-8.53%
DALYs	Mongolia	Female	All Ages	Aortic aneurysm	Number	1990	110.5292233	141.3889399	85.96697132	
DALYs	Mongolia	Female	All Ages	Aortic aneurysm	Number	2019	239.6110448	317.7407444	178.2752226	116.79%
Deaths	Mongolia	Female	Age-standardized	Aortic aneurysm	Rate	1990	0.905528651	1.113582767	0.722384925	
Deaths	Mongolia	Female	Age-standardized	Aortic aneurysm	Rate	2019	0.866188756	1.077751444	0.68727381	-4.34%
Deaths	Mongolia	Female	All Ages	Aortic aneurysm	Number	1990	4.647920127	5.839387272	3.678048842	
Deaths	Mongolia	Female	All Ages	Aortic aneurysm	Number	2019	9.062885995	11.50639388	6.936917037	94.99%
DALYs	Montenegro	Female	Age-standardized	Aortic aneurysm	Rate	1990	78.76227078	91.55508128	66.96582141	
DALYs	Montenegro	Female	Age-standardized	Aortic aneurysm	Rate	2019	94.07925141	113.4969175	76.05308457	19.45%
DALYs	Montenegro	Female	All Ages	Aortic aneurysm	Number	1990	269.1631732	315.4066741	228.7330542	
DALYs	Montenegro	Female	All Ages	Aortic aneurysm	Number	2019	475.5309702	572.4507878	382.6133044	76.67%
Deaths	Montenegro	Female	Age-standardized	Aortic aneurysm	Rate	1990	3.586883905	4.225961858	3.02086808	
Deaths	Montenegro	Female	Age-standardized	Aortic aneurysm	Rate	2019	4.477384939	5.338695002	3.62770975	24.83%
Deaths	Montenegro	Female	All Ages	Aortic aneurysm	Number	1990	12.09349176	14.23209625	10.18861907	
Deaths	Montenegro	Female	All Ages	Aortic aneurysm	Number	2019	24.04777941	28.66118407	19.42053541	98.85%
DALYs	Morocco	Female	Age-standardized	Aortic aneurysm	Rate	1990	10.73850638	16.34345385	8.114557959	
DALYs	Morocco	Female	Age-standardized	Aortic aneurysm	Rate	2019	12.21000832	18.0946806	8.981568782	13.70%
DALYs	Morocco	Female	All Ages	Aortic aneurysm	Number	1990	751.9629638	1260.300167	554.1415663	
DALYs	Morocco	Female	All Ages	Aortic aneurysm	Number	2019	1857.470464	2890.474007	1330.762913	147.02%
Deaths	Morocco	Female	Age-standardized	Aortic aneurysm	Rate	1990	0.57270406	0.779303007	0.439139019	
Deaths	Morocco	Female	Age-standardized	Aortic aneurysm	Rate	2019	0.685952075	0.918300391	0.526111752	19.77%
Deaths	Morocco	Female	All Ages	Aortic aneurysm	Number	1990	32.92378434	47.62951212	25.0537614	
Deaths	Morocco	Female	All Ages	Aortic aneurysm	Number	2019	88.46687763	122.6613207	66.94175705	168.70%
DALYs	Mozambique	Female	Age-standardized	Aortic aneurysm	Rate	1990	36.86681454	49.53158485	18.01781189	
DALYs	Mozambique	Female	Age-standardized	Aortic aneurysm	Rate	2019	32.3773201	46.61060024	17.65370843	-12.18%
DALYs	Mozambique	Female	All Ages	Aortic aneurysm	Number	1990	1095.65407	1519.980802	522.9431657	
DALYs	Mozambique	Female	All Ages	Aortic aneurysm	Number	2019	1920.980443	2780.252962	1013.859747	75.33%
Deaths	Mozambique	Female	Age-standardized	Aortic aneurysm	Rate	1990	2.087799616	2.790447649	1.045646266	
Deaths	Mozambique	Female	Age-standardized	Aortic aneurysm	Rate	2019	1.851053725	2.629865436	1.00848252	-11.34%
Deaths	Mozambique	Female	All Ages	Aortic aneurysm	Number	1990	49.83066327	66.87220143	24.51699423	
Deaths	Mozambique	Female	All Ages	Aortic aneurysm	Number	2019	90.10252078	128.3534118	49.17384466	80.82%
DALYs	Myanmar	Female	Age-standardized	Aortic aneurysm	Rate	1990	14.16197437	27.87894383	9.805872299	
DALYs	Myanmar	Female	Age-standardized	Aortic aneurysm	Rate	2019	16.7847467	21.9812419	12.86348753	18.52%
DALYs	Myanmar	Female	All Ages	Aortic aneurysm	Number	1990	1727.182849	3671.326615	1152.539641	
DALYs	Myanmar	Female	All Ages	Aortic aneurysm	Number	2019	4202.469822	5559.82218	3187.915391	143.31%
Deaths	Myanmar	Female	Age-standardized	Aortic aneurysm	Rate	1990	0.760119877	1.314258661	0.546092449	
Deaths	Myanmar	Female	Age-standardized	Aortic aneurysm	Rate	2019	0.93440888	1.226402626	0.731598967	22.93%
Deaths	Myanmar	Female	All Ages	Aortic aneurysm	Number	1990	75.28014348	140.8300579	52.67407526	
Deaths	Myanmar	Female	All Ages	Aortic aneurysm	Number	2019	208.4927859	273.6902348	162.6312363	176.96%
DALYs	Namibia	Female	Age-standardized	Aortic aneurysm	Rate	1990	38.37847422	49.34687837	29.1585658	
DALYs	Namibia	Female	Age-standardized	Aortic aneurysm	Rate	2019	24.39562669	33.44207207	17.33912428	-36.43%
DALYs	Namibia	Female	All Ages	Aortic aneurysm	Number	1990	150.9141927	194.1566283	113.3515908	
DALYs	Namibia	Female	All Ages	Aortic aneurysm	Number	2019	202.1618681	284.3676515	139.9561714	33.96%
Deaths	Namibia	Female	Age-standardized	Aortic aneurysm	Rate	1990	2.021794439	2.573467398	1.561067634	
Deaths	Namibia	Female	Age-standardized	Aortic aneurysm	Rate	2019	1.358478049	1.784597677	1.00603076	-32.81%
Deaths	Namibia	Female	All Ages	Aortic aneurysm	Number	1990	6.7100247	8.5464133	5.163313224	
Deaths	Namibia	Female	All Ages	Aortic aneurysm	Number	2019	9.948480445	13.21393974	7.272991321	48.26%
DALYs	Nauru	Female	Age-standardized	Aortic aneurysm	Rate	1990	71.82552226	107.4415952	53.84504429	
DALYs	Nauru	Female	Age-standardized	Aortic aneurysm	Rate	2019	51.9208555	79.59901866	37.54076465	-27.71%
DALYs	Nauru	Female	All Ages	Aortic aneurysm	Number	1990	1.209656655	1.948496926	0.848781921	
DALYs	Nauru	Female	All Ages	Aortic aneurysm	Number	2019	1.135968832	1.915775737	0.761360962	-6.09%
Deaths	Nauru	Female	Age-standardized	Aortic aneurysm	Rate	1990	4.055545677	5.590070386	3.10464485	
Deaths	Nauru	Female	Age-standardized	Aortic aneurysm	Rate	2019	2.862253177	4.065831764	2.145469928	-29.42%
Deaths	Nauru	Female	All Ages	Aortic aneurysm	Number	1990	0.046094753	0.070438812	0.034001017	
Deaths	Nauru	Female	All Ages	Aortic aneurysm	Number	2019	0.038358956	0.061690706	0.026530735	-16.78%
DALYs	Nepal	Female	Age-standardized	Aortic aneurysm	Rate	1990	14.16533028	26.28410878	9.32882828	

DALYs	Nepal	Female	Age-standardized	Aortic aneurysm	Rate	2019	19.83804258	27.48439893	13.13561222	40.05%
DALYs	Nepal	Female	All Ages	Aortic aneurysm	Number	1990	626.1260278	1221.091605	411.8062997	
DALYs	Nepal	Female	All Ages	Aortic aneurysm	Number	2019	2215.267678	3107.314382	1454.255508	253.81%
Deaths	Nepal	Female	Age-standardized	Aortic aneurysm	Rate	1990	0.745039257	1.30088717	0.48875026	
Deaths	Nepal	Female	Age-standardized	Aortic aneurysm	Rate	2019	1.132222169	1.565542206	0.749095307	51.97%
Deaths	Nepal	Female	All Ages	Aortic aneurysm	Number	1990	26.72515208	48.54618999	17.5324306	
Deaths	Nepal	Female	All Ages	Aortic aneurysm	Number	2019	109.0353197	150.6134462	71.94269077	307.99%
DALYs	Netherlands	Female	Age-standardized	Aortic aneurysm	Rate	1990	48.87966665	52.3082052	44.93867797	
DALYs	Netherlands	Female	Age-standardized	Aortic aneurysm	Rate	2019	42.54771535	48.05854789	37.32507161	-12.95%
DALYs	Netherlands	Female	All Ages	Aortic aneurysm	Number	1990	5964.470929	6420.712877	5451.546643	
DALYs	Netherlands	Female	All Ages	Aortic aneurysm	Number	2019	8145.456469	9284.665309	7058.373997	36.57%
Deaths	Netherlands	Female	Age-standardized	Aortic aneurysm	Rate	1990	3.07779958	3.316786194	2.755830075	
Deaths	Netherlands	Female	Age-standardized	Aortic aneurysm	Rate	2019	2.742108908	3.142489787	2.345062543	-10.91%
Deaths	Netherlands	Female	All Ages	Aortic aneurysm	Number	1990	405.1906933	439.12682	360.9384003	
Deaths	Netherlands	Female	All Ages	Aortic aneurysm	Number	2019	592.6916387	682.2961429	501.104811	46.27%
DALYs	New Zealand	Female	Age-standardized	Aortic aneurysm	Rate	1990	106.6652258	114.1816284	98.93759034	
DALYs	New Zealand	Female	Age-standardized	Aortic aneurysm	Rate	2019	66.21407319	74.02008752	58.38134032	-37.92%
DALYs	New Zealand	Female	All Ages	Aortic aneurysm	Number	1990	2417.667616	2590.657262	2237.484999	
DALYs	New Zealand	Female	All Ages	Aortic aneurysm	Number	2019	2857.658819	3200.478013	2497.550773	18.20%
Deaths	New Zealand	Female	Age-standardized	Aortic aneurysm	Rate	1990	6.307276134	6.805406101	5.768646611	
Deaths	New Zealand	Female	Age-standardized	Aortic aneurysm	Rate	2019	4.200391173	4.743248386	3.623804844	-33.40%
Deaths	New Zealand	Female	All Ages	Aortic aneurysm	Number	1990	148.649903	160.7858863	135.51888	
Deaths	New Zealand	Female	All Ages	Aortic aneurysm	Number	2019	198.704401	224.8293899	169.389771	33.67%
DALYs	Nicaragua	Female	Age-standardized	Aortic aneurysm	Rate	1990	8.298782229	9.673697032	7.116027135	
DALYs	Nicaragua	Female	Age-standardized	Aortic aneurysm	Rate	2019	10.79595765	12.84231071	8.980067077	30.09%
DALYs	Nicaragua	Female	All Ages	Aortic aneurysm	Number	1990	76.5671062	90.20896425	66.04677181	
DALYs	Nicaragua	Female	All Ages	Aortic aneurysm	Number	2019	258.0545682	310.948544	213.2457783	237.03%
Deaths	Nicaragua	Female	Age-standardized	Aortic aneurysm	Rate	1990	0.410480851	0.48130192	0.343297897	
Deaths	Nicaragua	Female	Age-standardized	Aortic aneurysm	Rate	2019	0.630442052	0.74291297	0.523929964	53.59%
Deaths	Nicaragua	Female	All Ages	Aortic aneurysm	Number	1990	3.254636349	3.824036053	2.762136005	
Deaths	Nicaragua	Female	All Ages	Aortic aneurysm	Number	2019	13.12447313	15.52016599	10.9741078	303.25%
DALYs	Niger	Female	Age-standardized	Aortic aneurysm	Rate	1990	31.48943163	49.84934867	15.91502671	
DALYs	Niger	Female	Age-standardized	Aortic aneurysm	Rate	2019	16.82610737	26.36649203	7.65933328	-46.57%
DALYs	Niger	Female	All Ages	Aortic aneurysm	Number	1990	424.6698216	685.3694681	211.5313402	
DALYs	Niger	Female	All Ages	Aortic aneurysm	Number	2019	693.8024509	1103.42585	309.5477883	63.37%
Deaths	Niger	Female	Age-standardized	Aortic aneurysm	Rate	1990	1.569439668	2.473749029	0.802692048	
Deaths	Niger	Female	Age-standardized	Aortic aneurysm	Rate	2019	0.853204875	1.281627821	0.397410494	-45.64%
Deaths	Niger	Female	All Ages	Aortic aneurysm	Number	1990	16.8557724	26.78141713	8.473911013	
Deaths	Niger	Female	All Ages	Aortic aneurysm	Number	2019	27.61311405	43.28432324	12.63568964	63.82%
DALYs	Nigeria	Female	Age-standardized	Aortic aneurysm	Rate	1990	40.33977673	64.73733412	27.36122909	
DALYs	Nigeria	Female	Age-standardized	Aortic aneurysm	Rate	2019	17.48219286	24.35162357	12.04827895	-56.66%
DALYs	Nigeria	Female	All Ages	Aortic aneurysm	Number	1990	8521.7679	13722.32742	5757.146135	
DALYs	Nigeria	Female	All Ages	Aortic aneurysm	Number	2019	8053.167032	11684.86242	5369.431529	-5.50%
Deaths	Nigeria	Female	Age-standardized	Aortic aneurysm	Rate	1990	2.113256981	3.251069872	1.469698317	
Deaths	Nigeria	Female	Age-standardized	Aortic aneurysm	Rate	2019	0.943461892	1.249339377	0.664089624	-55.36%
Deaths	Nigeria	Female	All Ages	Aortic aneurysm	Number	1990	404.8502209	635.0284466	281.3482057	
Deaths	Nigeria	Female	All Ages	Aortic aneurysm	Number	2019	345.1867487	475.8190922	239.2316	-14.74%
DALYs	Niue	Female	Age-standardized	Aortic aneurysm	Rate	1990	53.15652991	75.02599371	39.676798	
DALYs	Niue	Female	Age-standardized	Aortic aneurysm	Rate	2019	38.07498482	52.58865189	27.42067848	-28.37%
DALYs	Niue	Female	All Ages	Aortic aneurysm	Number	1990	0.704181465	0.98314139	0.529662768	
DALYs	Niue	Female	All Ages	Aortic aneurysm	Number	2019	0.44642818	0.614551243	0.321515985	-36.60%
Deaths	Niue	Female	Age-standardized	Aortic aneurysm	Rate	1990	2.979936732	4.10021923	2.256420186	
Deaths	Niue	Female	Age-standardized	Aortic aneurysm	Rate	2019	2.075228739	2.878095222	1.503839916	-30.36%
Deaths	Niue	Female	All Ages	Aortic aneurysm	Number	1990	0.046422017	0.062346013	0.035219967	
Deaths	Niue	Female	All Ages	Aortic aneurysm	Number	2019	0.025734656	0.035599205	0.01855482	-44.56%
DALYs	North Macedonia	Female	Age-standardized	Aortic aneurysm	Rate	1990	16.35144828	18.54398383	14.49300277	
DALYs	North Macedonia	Female	Age-standardized	Aortic aneurysm	Rate	2019	25.28759922	32.22441525	19.46917039	54.65%
DALYs	North Macedonia	Female	All Ages	Aortic aneurysm	Number	1990	150.9863581	171.1146098	134.1626111	
DALYs	North Macedonia	Female	All Ages	Aortic aneurysm	Number	2019	398.9476621	508.9259716	306.1576023	164.23%
Deaths	North Macedonia	Female	Age-standardized	Aortic aneurysm	Rate	1990	0.911457976	1.048167594	0.784451756	
Deaths	North Macedonia	Female	Age-standardized	Aortic aneurysm	Rate	2019	1.384149231	1.717731274	1.097743344	51.86%
Deaths	North Macedonia	Female	All Ages	Aortic aneurysm	Number	1990	7.375878471	8.437587921	6.445644688	
Deaths	North Macedonia	Female	All Ages	Aortic aneurysm	Number	2019	20.71876862	26.06330695	16.25681936	180.90%
DALYs	Northern Mariana Islands	Female	Age-standardized	Aortic aneurysm	Rate	1990	165.4589695	213.5617597	128.25361	
DALYs	Northern Mariana Islands	Female	Age-standardized	Aortic aneurysm	Rate	2019	29.30808288	36.81148041	23.46592153	-82.29%
DALYs	Northern Mariana Islands	Female	All Ages	Aortic aneurysm	Number	1990	11.91941621	15.7247067	8.923594015	
DALYs	Northern Mariana Islands	Female	All Ages	Aortic aneurysm	Number	2019	6.903679163	8.830271082	5.384454997	-42.08%
Deaths	Northern Mariana Islands	Female	Age-standardized	Aortic aneurysm	Rate	1990	9.40876077	12.12325782	7.293536627	
Deaths	Northern Mariana Islands	Female	Age-standardized	Aortic aneurysm	Rate	2019	1.650686487	2.060348825	1.326653813	-82.46%
Deaths	Northern Mariana Islands	Female	All Ages	Aortic aneurysm	Number	1990	0.447993774	0.580238967	0.344537404	
Deaths	Northern Mariana Islands	Female	All Ages	Aortic aneurysm	Number	2019	0.305299739	0.384889398	0.244573445	-31.85%
DALYs	Norway	Female	Age-standardized	Aortic aneurysm	Rate	1990	62.98113696	66.12417321	58.75204411	
DALYs	Norway	Female	Age-standardized	Aortic aneurysm	Rate	2019	51.98501473	57.29576487	45.69082261	-17.46%
DALYs	Norway	Female	All Ages	Aortic aneurysm	Number	1990	2659.755619	2803.66734	2454.580801	
DALYs	Norway	Female	All Ages	Aortic aneurysm	Number	2019	2853.726653	3147.575953	2477.233485	7.29%
Deaths	Norway	Female	Age-standardized	Aortic aneurysm	Rate	1990	3.806402763	4.024891299	3.46050672	
Deaths	Norway	Female	Age-standardized	Aortic aneurysm	Rate	2019	3.464828379	3.835570114	2.974780812	-8.97%
Deaths	Norway	Female	All Ages	Aortic aneurysm	Number	1990	177.1823315	187.9984796	160.0459833	
Deaths	Norway	Female	All Ages	Aortic aneurysm	Number	2019	215.5278426	240.437798	181.507939	21.64%
DALYs	Oman	Female	Age-standardized	Aortic aneurysm	Rate	1990	17.56944541	32.05171751	12.54343017	
DALYs	Oman	Female	Age-standardized	Aortic aneurysm	Rate	2019	15.68980949	22.9392288	11.77187968	-10.70%
DALYs	Oman	Female	All Ages	Aortic aneurysm	Number	1990	56.7406045	104.1105418	39.34116616	
DALYs	Oman	Female	All Ages	Aortic aneurysm	Number	2019	135.4695759	198.7375587	100.2245025	138.75%
Deaths	Oman	Female	Age-standardized	Aortic aneurysm	Rate	1990	0.883050268	1.535530597	0.647706683	
Deaths	Oman	Female	Age-standardized	Aortic aneurysm	Rate	2019	0.822764334	1.21036918	0.611111777	-6.83%
Deaths	Oman	Female	All Ages	Aortic aneurysm	Number	1990	2.200648438	3.988864258	1.586248978	
Deaths	Oman	Female	All Ages	Aortic aneurysm	Number	2019	4.699932099	6.879013791	3.501137683	113.57%
DALYs	Pakistan	Female	Age-standardized	Aortic aneurysm	Rate	1990	20.57959211	37.18024985	14.93157504	
DALYs	Pakistan	Female	Age-standardized	Aortic aneurysm	Rate	2019	23.8697729	39.69609549	17.75117248	15.99%
DALYs	Pakistan	Female	All Ages	Aortic aneurysm	Number	1990	5171.52895	9828.439604	3770.554977	
DALYs	Pakistan	Female	All Ages	Aortic aneurysm	Number	2019	12338.33346	22109.53281	8828.790411	138.58%
Deaths	Pakistan	Female	Age-standardized	Aortic aneurysm	Rate	1990	1.129710801	1.890659969	0.811560527	
Deaths	Pakistan	Female	Age-standardized	Aortic aneurysm	Rate	2019	1.334569792	1.951020681	1.008954243	18.13%
Deaths	Pakistan	Female	All Ages	Aortic aneurysm	Number	1990	249.2363811	432.3973167	179.6985539	
Deaths	Pakistan	Female	All Ages	Aortic aneurysm	Number	2019	548.7393411	886.8457926	409.1478453	120.17%
DALYs	Palau	Female	Age-standardized	Aortic aneurysm	Rate	1990	11.06500117	15.54050719	7.695640818	
DALYs	Palau	Female	Age-standardized	Aortic aneurysm	Rate	2019	9.487544885	12.10646265	7.111285117	-14.26%
DALYs	Palau	Female	All Ages	Aortic aneurysm	Number	1990	0.527882663	0.748710299	0.358872815	
DALYs	Palau	Female	All Ages	Aortic aneurysm	Number	2019	0.938297869	1.224237387	0.691354677	77.75%
Deaths	Palau	Female	Age-standardized	Aortic aneurysm	Rate	1990	0.615938139	0.846068491	0.433208062	
Deaths	Palau	Female	Age-standardized	Aortic aneurysm	Rate	2019	0.527957384	0.665591139	0.392502379	-14.28%

Deaths	Palau	Female	All Ages	Aortic aneurysm	Number	1990	0.024391033	0.033618341	0.016946404	
Deaths	Palau	Female	All Ages	Aortic aneurysm	Number	2019	0.042743901	0.054614743	0.031825574	75.24%
DALYs	Palestine	Female	Age-standardized	Aortic aneurysm	Rate	1990	11.62117663	16.84379023	7.023609478	
DALYs	Palestine	Female	Age-standardized	Aortic aneurysm	Rate	2019	12.00037264	14.17425826	9.971065462	3.26%
DALYs	Palestine	Female	All Ages	Aortic aneurysm	Number	1990	55.11678901	81.83537903	33.21768056	
DALYs	Palestine	Female	All Ages	Aortic aneurysm	Number	2019	149.259815	176.4475182	124.1052022	170.81%
Deaths	Palestine	Female	Age-standardized	Aortic aneurysm	Rate	1990	0.630591591	0.884569636	0.393191711	
Deaths	Palestine	Female	Age-standardized	Aortic aneurysm	Rate	2019	0.662657153	0.78766367	0.551956766	5.08%
Deaths	Palestine	Female	All Ages	Aortic aneurysm	Number	1990	2.633002122	3.724646167	1.618951503	
Deaths	Palestine	Female	All Ages	Aortic aneurysm	Number	2019	6.750347047	7.961261384	5.597251937	156.37%
DALYs	Panama	Female	Age-standardized	Aortic aneurysm	Rate	1990	22.06243264	25.61815993	19.40058908	
DALYs	Panama	Female	Age-standardized	Aortic aneurysm	Rate	2019	17.76817603	22.49510714	13.37128938	-19.46%
DALYs	Panama	Female	All Ages	Aortic aneurysm	Number	1990	174.2138657	201.8400189	154.0702541	
DALYs	Panama	Female	All Ages	Aortic aneurysm	Number	2019	380.4432473	481.3770373	286.802269	118.38%
Deaths	Panama	Female	Age-standardized	Aortic aneurysm	Rate	1990	1.112325148	1.293679735	0.960479828	
Deaths	Panama	Female	Age-standardized	Aortic aneurysm	Rate	2019	0.907224082	1.13669108	0.688117194	-18.44%
Deaths	Panama	Female	All Ages	Aortic aneurysm	Number	1990	8.079785154	9.378318195	7.032340594	
Deaths	Panama	Female	All Ages	Aortic aneurysm	Number	2019	20.16091551	25.21266559	15.27374484	149.52%
DALYs	Papua New Guinea	Female	Age-standardized	Aortic aneurysm	Rate	1990	37.76956104	64.9789575	19.60699351	
DALYs	Papua New Guinea	Female	Age-standardized	Aortic aneurysm	Rate	2019	35.44631611	58.28727106	20.42609314	-6.15%
DALYs	Papua New Guinea	Female	All Ages	Aortic aneurysm	Number	1990	304.574214	548.9720559	152.2394047	
DALYs	Papua New Guinea	Female	All Ages	Aortic aneurysm	Number	2019	754.2585222	1273.534257	413.4320243	147.64%
Deaths	Papua New Guinea	Female	Age-standardized	Aortic aneurysm	Rate	1990	2.21146546	3.602056538	1.195818316	
Deaths	Papua New Guinea	Female	Age-standardized	Aortic aneurysm	Rate	2019	1.997282678	3.208079321	1.191467108	-9.69%
Deaths	Papua New Guinea	Female	All Ages	Aortic aneurysm	Number	1990	12.90141488	22.1724484	6.721847299	
Deaths	Papua New Guinea	Female	All Ages	Aortic aneurysm	Number	2019	31.57925067	51.94707331	18.13566274	144.77%
DALYs	Paraguay	Female	Age-standardized	Aortic aneurysm	Rate	1990	28.3024336	33.08752426	23.89741393	
DALYs	Paraguay	Female	Age-standardized	Aortic aneurysm	Rate	2019	31.46157723	40.6390052	23.46986875	11.16%
DALYs	Paraguay	Female	All Ages	Aortic aneurysm	Number	1990	340.8459547	398.7182105	289.1137871	
DALYs	Paraguay	Female	All Ages	Aortic aneurysm	Number	2019	923.2261948	1194.512536	686.1019532	170.86%
Deaths	Paraguay	Female	Age-standardized	Aortic aneurysm	Rate	1990	1.29512451	1.509492923	1.086043272	
Deaths	Paraguay	Female	Age-standardized	Aortic aneurysm	Rate	2019	1.511344189	1.938053542	1.141284625	16.69%
Deaths	Paraguay	Female	All Ages	Aortic aneurysm	Number	1990	14.71660023	17.11993867	12.41021712	
Deaths	Paraguay	Female	All Ages	Aortic aneurysm	Number	2019	43.43108816	55.43326821	32.84928575	195.12%
DALYs	Peru	Female	Age-standardized	Aortic aneurysm	Rate	1990	17.0745288	21.84596294	13.96634657	
DALYs	Peru	Female	Age-standardized	Aortic aneurysm	Rate	2019	14.13932599	18.859558	10.21045834	-17.19%
DALYs	Peru	Female	All Ages	Aortic aneurysm	Number	1990	1105.628334	1406.000195	896.9102975	
DALYs	Peru	Female	All Ages	Aortic aneurysm	Number	2019	2387.73135	3190.664922	1725.136937	115.96%
Deaths	Peru	Female	Age-standardized	Aortic aneurysm	Rate	1990	0.913393146	1.153804674	0.744175539	
Deaths	Peru	Female	Age-standardized	Aortic aneurysm	Rate	2019	0.732722278	0.966731134	0.53213375	-19.78%
Deaths	Peru	Female	All Ages	Aortic aneurysm	Number	1990	51.62082665	65.61285704	41.99234114	
Deaths	Peru	Female	All Ages	Aortic aneurysm	Number	2019	125.4094724	165.3856793	90.98156569	142.94%
DALYs	Philippines	Female	Age-standardized	Aortic aneurysm	Rate	1990	11.53159522	13.8067442	10.03924751	
DALYs	Philippines	Female	Age-standardized	Aortic aneurysm	Rate	2019	22.36912095	28.58270663	17.2122453	93.98%
DALYs	Philippines	Female	All Ages	Aortic aneurysm	Number	1990	1576.281583	1887.439002	1361.160672	
DALYs	Philippines	Female	All Ages	Aortic aneurysm	Number	2019	9320.014499	12078.6776	7089.941451	491.27%
Deaths	Philippines	Female	Age-standardized	Aortic aneurysm	Rate	1990	0.763350434	0.895615515	0.660351172	
Deaths	Philippines	Female	Age-standardized	Aortic aneurysm	Rate	2019	1.19433429	1.488520366	0.943652974	56.46%
Deaths	Philippines	Female	All Ages	Aortic aneurysm	Number	1990	79.25069222	95.19863004	68.81620671	
Deaths	Philippines	Female	All Ages	Aortic aneurysm	Number	2019	422.1937396	530.068291	330.4396075	432.73%
DALYs	Poland	Female	Age-standardized	Aortic aneurysm	Rate	1990	21.75445981	22.60684936	20.8550321	
DALYs	Poland	Female	Age-standardized	Aortic aneurysm	Rate	2019	29.77173847	37.52150314	23.4109266	36.85%
DALYs	Poland	Female	All Ages	Aortic aneurysm	Number	1990	5398.405987	5617.63805	5159.265676	
DALYs	Poland	Female	All Ages	Aortic aneurysm	Number	2019	11495.62476	14371.6131	9113.364641	112.94%
Deaths	Poland	Female	Age-standardized	Aortic aneurysm	Rate	1990	0.990049034	1.03489544	0.931399714	
Deaths	Poland	Female	Age-standardized	Aortic aneurysm	Rate	2019	1.51868311	1.886031231	1.20752412	53.39%
Deaths	Poland	Female	All Ages	Aortic aneurysm	Number	1990	253.5384818	265.3728959	239.1088872	
Deaths	Poland	Female	All Ages	Aortic aneurysm	Number	2019	672.125459	831.1594288	535.050928	165.10%
DALYs	Portugal	Female	Age-standardized	Aortic aneurysm	Rate	1990	14.08536343	15.08907448	13.06707966	
DALYs	Portugal	Female	Age-standardized	Aortic aneurysm	Rate	2019	15.85960669	17.68620789	13.89307433	12.60%
DALYs	Portugal	Female	All Ages	Aortic aneurysm	Number	1990	1024.261909	1102.593787	949.1572903	
DALYs	Portugal	Female	All Ages	Aortic aneurysm	Number	2019	1954.725764	2192.481688	1693.652723	90.84%
Deaths	Portugal	Female	Age-standardized	Aortic aneurysm	Rate	1990	0.675545497	0.727031724	0.620018916	
Deaths	Portugal	Female	Age-standardized	Aortic aneurysm	Rate	2019	0.815706617	0.91710511	0.702799681	20.75%
Deaths	Portugal	Female	All Ages	Aortic aneurysm	Number	1990	52.06458835	56.36980289	47.48120246	
Deaths	Portugal	Female	All Ages	Aortic aneurysm	Number	2019	129.7286455	147.7971667	109.7667163	149.17%
DALYs	Puerto Rico	Female	Age-standardized	Aortic aneurysm	Rate	1990	16.93496229	18.83040636	15.13857902	
DALYs	Puerto Rico	Female	Age-standardized	Aortic aneurysm	Rate	2019	11.44856211	14.49954081	8.808186092	-32.40%
DALYs	Puerto Rico	Female	All Ages	Aortic aneurysm	Number	1990	333.2478148	371.5311247	296.8388036	
DALYs	Puerto Rico	Female	All Ages	Aortic aneurysm	Number	2019	457.0438578	576.8844598	353.583977	37.15%
Deaths	Puerto Rico	Female	Age-standardized	Aortic aneurysm	Rate	1990	1.002474364	1.126562721	0.873039391	
Deaths	Puerto Rico	Female	Age-standardized	Aortic aneurysm	Rate	2019	0.677816943	0.85159231	0.52426806	-32.39%
Deaths	Puerto Rico	Female	All Ages	Aortic aneurysm	Number	1990	19.31653728	21.66115317	16.80915584	
Deaths	Puerto Rico	Female	All Ages	Aortic aneurysm	Number	2019	32.99298935	41.49088263	25.44503338	70.80%
DALYs	Qatar	Female	Age-standardized	Aortic aneurysm	Rate	1990	18.26790611	24.22373245	13.66540609	
DALYs	Qatar	Female	Age-standardized	Aortic aneurysm	Rate	2019	21.89622473	27.75872704	16.59661715	19.86%
DALYs	Qatar	Female	All Ages	Aortic aneurysm	Number	1990	6.548324837	8.468508282	5.047984766	
DALYs	Qatar	Female	All Ages	Aortic aneurysm	Number	2019	35.15892674	47.01794056	24.56556832	436.91%
Deaths	Qatar	Female	Age-standardized	Aortic aneurysm	Rate	1990	1.121252761	1.492617373	0.828956557	
Deaths	Qatar	Female	Age-standardized	Aortic aneurysm	Rate	2019	1.543053691	1.945541698	1.191656568	37.62%
Deaths	Qatar	Female	All Ages	Aortic aneurysm	Number	1990	0.27416077	0.357542087	0.210812755	
Deaths	Qatar	Female	All Ages	Aortic aneurysm	Number	2019	1.259648156	1.650226141	0.905558819	359.46%
DALYs	Republic of Korea	Female	Age-standardized	Aortic aneurysm	Rate	1990	25.57456861	35.56300466	21.0320363	
DALYs	Republic of Korea	Female	Age-standardized	Aortic aneurysm	Rate	2019	20.13709677	23.83867534	16.8089702	-21.26%
DALYs	Republic of Korea	Female	All Ages	Aortic aneurysm	Number	1990	4123.627094	5798.165881	3405.616931	
DALYs	Republic of Korea	Female	All Ages	Aortic aneurysm	Number	2019	10096.7682	12012.09809	8393.11917	144.85%
Deaths	Republic of Korea	Female	Age-standardized	Aortic aneurysm	Rate	1990	1.534088465	2.093396463	1.245975472	
Deaths	Republic of Korea	Female	Age-standardized	Aortic aneurysm	Rate	2019	1.396809454	1.678302914	1.123235369	-8.95%
Deaths	Republic of Korea	Female	All Ages	Aortic aneurysm	Number	1990	211.478699	289.7273451	172.4591339	
Deaths	Republic of Korea	Female	All Ages	Aortic aneurysm	Number	2019	730.4483211	877.6684918	587.3601656	245.40%
DALYs	Republic of Moldova	Female	Age-standardized	Aortic aneurysm	Rate	1990	12.19167819	14.12030426	10.67909039	
DALYs	Republic of Moldova	Female	Age-standardized	Aortic aneurysm	Rate	2019	18.99255343	22.50703789	15.62656239	55.78%
DALYs	Republic of Moldova	Female	All Ages	Aortic aneurysm	Number	1990	310.1271336	360.358884	270.0523685	
DALYs	Republic of Moldova	Female	All Ages	Aortic aneurysm	Number	2019	608.319343	717.4364463	502.1577201	96.15%
Deaths	Republic of Moldova	Female	Age-standardized	Aortic aneurysm	Rate	1990	0.546690747	0.63139037	0.480177933	
Deaths	Republic of Moldova	Female	Age-standardized	Aortic aneurysm	Rate	2019	0.837489232	0.982615701	0.706569039	53.19%
Deaths	Republic of Moldova	Female	All Ages	Aortic aneurysm	Number	1990	12.95260775	15.07536928	11.34360915	
Deaths	Republic of Moldova	Female	All Ages	Aortic aneurysm	Number	2019	29.14656184	34.08673623	24.54653583	125.02%
DALYs	Romania	Female	Age-standardized	Aortic aneurysm	Rate	1990	26.28386871	30.13339778	23.29416819	
DALYs	Romania	Female	Age-standardized	Aortic aneurysm	Rate	2019	35.37160214	42.79412176	28.52658893	34.58%
DALYs	Romania	Female	All Ages	Aortic aneurysm	Number	1990	3864.525165	4461.94836	3418.77661	

DALYs	Romania	Female	All Ages	Aortic aneurysm	Number	2019	6867.088116	8294.181536	5578.297834	77.70%
Deaths	Romania	Female	Age-standardized	Aortic aneurysm	Rate	1990	1.234947222	1.423705039	1.088113741	
Deaths	Romania	Female	Age-standardized	Aortic aneurysm	Rate	2019	1.756593051	2.121452021	1.437409056	42.24%
Deaths	Romania	Female	All Ages	Aortic aneurysm	Number	1990	176.4758467	205.3485252	154.3051823	
Deaths	Romania	Female	All Ages	Aortic aneurysm	Number	2019	405.4206233	488.3903568	330.7044242	129.73%
DALYs	Russian Federation	Female	Age-standardized	Aortic aneurysm	Rate	1990	36.14456988	37.80260563	31.97039217	
DALYs	Russian Federation	Female	Age-standardized	Aortic aneurysm	Rate	2019	46.45983616	56.56005019	37.45760132	28.54%
DALYs	Russian Federation	Female	All Ages	Aortic aneurysm	Number	1990	40795.43119	42524.09859	36561.95062	
DALYs	Russian Federation	Female	All Ages	Aortic aneurysm	Number	2019	64854.64662	78173.51121	52942.56485	58.98%
Deaths	Russian Federation	Female	Age-standardized	Aortic aneurysm	Rate	1990	1.638366288	1.705496256	1.497495814	
Deaths	Russian Federation	Female	Age-standardized	Aortic aneurysm	Rate	2019	2.280139872	2.712411784	1.874565165	39.17%
Deaths	Russian Federation	Female	All Ages	Aortic aneurysm	Number	1990	1906.523135	1980.361902	1757.448024	
Deaths	Russian Federation	Female	All Ages	Aortic aneurysm	Number	2019	3533.046839	4183.102406	2911.210301	85.31%
DALYs	Rwanda	Female	Age-standardized	Aortic aneurysm	Rate	1990	54.82062225	96.04547765	33.40413225	
DALYs	Rwanda	Female	Age-standardized	Aortic aneurysm	Rate	2019	33.84758135	47.897088	22.68267391	-38.26%
DALYs	Rwanda	Female	All Ages	Aortic aneurysm	Number	1990	864.7314088	1681.420777	506.9805021	
DALYs	Rwanda	Female	All Ages	Aortic aneurysm	Number	2019	1163.544789	1674.582953	773.761788	34.56%
Deaths	Rwanda	Female	Age-standardized	Aortic aneurysm	Rate	1990	2.881839856	4.617917437	1.81466142	
Deaths	Rwanda	Female	Age-standardized	Aortic aneurysm	Rate	2019	1.84588414	2.563358504	1.234116498	-35.95%
Deaths	Rwanda	Female	All Ages	Aortic aneurysm	Number	1990	36.55522178	62.5673052	22.1382769	
Deaths	Rwanda	Female	All Ages	Aortic aneurysm	Number	2019	51.60330444	72.83625225	34.62309174	41.17%
DALYs	Saint Kitts and Nevis	Female	Age-standardized	Aortic aneurysm	Rate	1990	39.99574299	50.83254199	34.40125321	
DALYs	Saint Kitts and Nevis	Female	Age-standardized	Aortic aneurysm	Rate	2019	36.57009853	43.8989048	29.46717377	-8.57%
DALYs	Saint Kitts and Nevis	Female	All Ages	Aortic aneurysm	Number	1990	8.662904331	10.95416669	7.385288187	
DALYs	Saint Kitts and Nevis	Female	All Ages	Aortic aneurysm	Number	2019	11.50427459	13.97318191	9.197559781	32.80%
Deaths	Saint Kitts and Nevis	Female	Age-standardized	Aortic aneurysm	Rate	1990	2.283876487	2.770315086	1.980412185	
Deaths	Saint Kitts and Nevis	Female	Age-standardized	Aortic aneurysm	Rate	2019	2.213645681	2.630962798	1.834664412	-3.08%
Deaths	Saint Kitts and Nevis	Female	All Ages	Aortic aneurysm	Number	1990	0.489373173	0.601144189	0.418566448	
Deaths	Saint Kitts and Nevis	Female	All Ages	Aortic aneurysm	Number	2019	0.633830483	0.753367911	0.524493978	29.52%
DALYs	Saint Lucia	Female	Age-standardized	Aortic aneurysm	Rate	1990	105.2245341	125.294437	92.36666951	
DALYs	Saint Lucia	Female	Age-standardized	Aortic aneurysm	Rate	2019	92.90412048	110.8072815	77.21865577	-11.71%
DALYs	Saint Lucia	Female	All Ages	Aortic aneurysm	Number	1990	51.3501164	61.54257727	45.04276257	
DALYs	Saint Lucia	Female	All Ages	Aortic aneurysm	Number	2019	105.2686202	125.3729618	87.68289974	105.00%
Deaths	Saint Lucia	Female	Age-standardized	Aortic aneurysm	Rate	1990	6.219300093	7.265465649	5.452591351	
Deaths	Saint Lucia	Female	Age-standardized	Aortic aneurysm	Rate	2019	5.561956053	6.602501227	4.597496749	-10.57%
Deaths	Saint Lucia	Female	All Ages	Aortic aneurysm	Number	1990	2.888050406	3.422027043	2.519359149	
Deaths	Saint Lucia	Female	All Ages	Aortic aneurysm	Number	2019	6.399688846	7.600843425	5.242942879	121.59%
DALYs	Saint Vincent and the Grenadines	Female	Age-standardized	Aortic aneurysm	Rate	1990	34.84601388	39.18015249	30.67746453	
DALYs	Saint Vincent and the Grenadines	Female	Age-standardized	Aortic aneurysm	Rate	2019	39.77147725	46.40768927	34.14554404	14.13%
DALYs	Saint Vincent and the Grenadines	Female	All Ages	Aortic aneurysm	Number	1990	14.2508277	16.12307426	12.48093444	
DALYs	Saint Vincent and the Grenadines	Female	All Ages	Aortic aneurysm	Number	2019	25.70406564	29.9490604	22.07522216	80.37%
Deaths	Saint Vincent and the Grenadines	Female	Age-standardized	Aortic aneurysm	Rate	1990	1.976327584	2.233470633	1.724043012	
Deaths	Saint Vincent and the Grenadines	Female	Age-standardized	Aortic aneurysm	Rate	2019	2.317302972	2.691685489	1.988624586	17.25%
Deaths	Saint Vincent and the Grenadines	Female	All Ages	Aortic aneurysm	Number	1990	0.796931529	0.905824935	0.692385386	
Deaths	Saint Vincent and the Grenadines	Female	All Ages	Aortic aneurysm	Number	2019	1.46504431	1.699223182	1.255335609	83.84%
DALYs	Samoa	Female	Age-standardized	Aortic aneurysm	Rate	1990	56.29252828	81.23602074	42.47050661	
DALYs	Samoa	Female	Age-standardized	Aortic aneurysm	Rate	2019	46.81392468	66.85411933	32.83941983	-16.84%
DALYs	Samoa	Female	All Ages	Aortic aneurysm	Number	1990	24.30968246	35.89069482	18.18434771	
DALYs	Samoa	Female	All Ages	Aortic aneurysm	Number	2019	34.32956485	49.2210685	23.67016764	41.22%
Deaths	Samoa	Female	Age-standardized	Aortic aneurysm	Rate	1990	3.196201197	4.436102621	2.461245337	
Deaths	Samoa	Female	Age-standardized	Aortic aneurysm	Rate	2019	2.527206662	3.541241436	1.808733118	-20.93%
Deaths	Samoa	Female	All Ages	Aortic aneurysm	Number	1990	1.243222856	1.735443135	0.960412004	
Deaths	Samoa	Female	All Ages	Aortic aneurysm	Number	2019	1.705556223	2.401609739	1.222010155	37.19%
DALYs	San Marino	Female	Age-standardized	Aortic aneurysm	Rate	1990	8.404007384	10.15289277	6.850824803	
DALYs	San Marino	Female	Age-standardized	Aortic aneurysm	Rate	2019	8.923889394	13.87244409	5.646132864	6.19%
DALYs	San Marino	Female	All Ages	Aortic aneurysm	Number	1990	1.498617441	1.800090586	1.220283352	
DALYs	San Marino	Female	All Ages	Aortic aneurysm	Number	2019	3.063132795	4.633018273	1.999454938	104.40%
Deaths	San Marino	Female	Age-standardized	Aortic aneurysm	Rate	1990	0.483760453	0.585594539	0.391496873	
Deaths	San Marino	Female	Age-standardized	Aortic aneurysm	Rate	2019	0.519207645	0.775933586	0.341696809	7.33%
Deaths	San Marino	Female	All Ages	Aortic aneurysm	Number	1990	0.091779288	0.111819279	0.073675107	
Deaths	San Marino	Female	All Ages	Aortic aneurysm	Number	2019	0.222435887	0.32307419	0.146014808	142.36%
DALYs	Sao Tome and Principe	Female	Age-standardized	Aortic aneurysm	Rate	1990	30.66677521	38.5886689	21.78142285	
DALYs	Sao Tome and Principe	Female	Age-standardized	Aortic aneurysm	Rate	2019	18.48026976	24.59641307	13.57538982	-39.74%
DALYs	Sao Tome and Principe	Female	All Ages	Aortic aneurysm	Number	1990	10.36468102	13.13933743	7.368106508	
DALYs	Sao Tome and Principe	Female	All Ages	Aortic aneurysm	Number	2019	10.67738771	14.25725889	7.644034401	3.02%
Deaths	Sao Tome and Principe	Female	Age-standardized	Aortic aneurysm	Rate	1990	1.534584471	1.903848651	1.09715223	
Deaths	Sao Tome and Principe	Female	Age-standardized	Aortic aneurysm	Rate	2019	0.928656735	1.21941327	0.704842337	-39.48%
Deaths	Sao Tome and Principe	Female	All Ages	Aortic aneurysm	Number	1990	0.473054619	0.585853352	0.340013384	
Deaths	Sao Tome and Principe	Female	All Ages	Aortic aneurysm	Number	2019	0.446096757	0.586933566	0.332571112	-5.70%
DALYs	Saudi Arabia	Female	Age-standardized	Aortic aneurysm	Rate	1990	11.09572779	15.00481125	6.49671219	
DALYs	Saudi Arabia	Female	Age-standardized	Aortic aneurysm	Rate	2019	8.79204242	11.39520182	6.771354677	-20.76%
DALYs	Saudi Arabia	Female	All Ages	Aortic aneurysm	Number	1990	293.3876067	399.6408956	175.4404504	
DALYs	Saudi Arabia	Female	All Ages	Aortic aneurysm	Number	2019	667.8470513	879.9891641	501.9076989	127.63%
Deaths	Saudi Arabia	Female	Age-standardized	Aortic aneurysm	Rate	1990	0.577816599	0.767578051	0.346345782	
Deaths	Saudi Arabia	Female	Age-standardized	Aortic aneurysm	Rate	2019	0.477406904	0.607205393	0.368964731	-17.38%
Deaths	Saudi Arabia	Female	All Ages	Aortic aneurysm	Number	1990	12.24884918	16.33078339	7.278165169	
Deaths	Saudi Arabia	Female	All Ages	Aortic aneurysm	Number	2019	24.58123808	31.8407554	18.9294329	100.68%
DALYs	Senegal	Female	Age-standardized	Aortic aneurysm	Rate	1990	29.19861778	38.90447789	18.98779369	
DALYs	Senegal	Female	Age-standardized	Aortic aneurysm	Rate	2019	15.93280363	21.38911376	11.54063676	-45.43%
DALYs	Senegal	Female	All Ages	Aortic aneurysm	Number	1990	467.4381155	628.2019162	302.7864571	
DALYs	Senegal	Female	All Ages	Aortic aneurysm	Number	2019	632.9510802	858.4753191	448.8839734	35.41%
Deaths	Senegal	Female	Age-standardized	Aortic aneurysm	Rate	1990	1.50083464	1.995730426	0.973620995	
Deaths	Senegal	Female	Age-standardized	Aortic aneurysm	Rate	2019	0.827371286	1.084605147	0.622284985	-44.87%
Deaths	Senegal	Female	All Ages	Aortic aneurysm	Number	1990	20.46531055	27.17277026	13.44622009	
Deaths	Senegal	Female	All Ages	Aortic aneurysm	Number	2019	28.04305465	37.15401233	20.68464402	37.03%
DALYs	Serbia	Female	Age-standardized	Aortic aneurysm	Rate	1990	35.3356408	40.80315346	30.72287152	
DALYs	Serbia	Female	Age-standardized	Aortic aneurysm	Rate	2019	50.43911937	63.49019652	39.64643017	42.74%
DALYs	Serbia	Female	All Ages	Aortic aneurysm	Number	1990	2064.904041	2404.271866	1783.236796	
DALYs	Serbia	Female	All Ages	Aortic aneurysm	Number	2019	4247.403659	5362.620511	3348.367617	105.69%
Deaths	Serbia	Female	Age-standardized	Aortic aneurysm	Rate	1990	1.761699519	2.045673304	1.522675342	
Deaths	Serbia	Female	Age-standardized	Aortic aneurysm	Rate	2019	2.858589948	3.5283377	2.298368237	62.26%
Deaths	Serbia	Female	All Ages	Aortic aneurysm	Number	1990	97.42254567	112.7113491	84.65986186	
Deaths	Serbia	Female	All Ages	Aortic aneurysm	Number	2019	248.5316458	312.2317301	198.1645994	155.11%
DALYs	Seychelles	Female	Age-standardized	Aortic aneurysm	Rate	1990	19.62598464	25.7179573	16.26156571	
DALYs	Seychelles	Female	Age-standardized	Aortic aneurysm	Rate	2019	19.62215723	25.61692932	14.96167694	-0.02%
DALYs	Seychelles	Female	All Ages	Aortic aneurysm	Number	1990	6.492915246	8.468186044	5.398952445	
DALYs	Seychelles	Female	All Ages	Aortic aneurysm	Number	2019	10.80547537	14.11742377	8.207642852	66.42%
Deaths	Seychelles	Female	Age-standardized	Aortic aneurysm	Rate	1990	1.070083968	1.407793018	0.877871695	
Deaths	Seychelles	Female	Age-standardized	Aortic aneurysm	Rate	2019	1.090939992	1.440670139	0.830544747	1.95%
Deaths	Seychelles	Female	All Ages	Aortic aneurysm	Number	1990	0.368912812	0.483553884	0.302053843	
Deaths	Seychelles	Female	All Ages	Aortic aneurysm	Number	2019	0.593205759	0.78135235	0.451243495	60.80%

DALYs	Sierra Leone	Female	Age-standardized	Aortic aneurysm	Rate	1990	26.738647	40.52733086	17.16155584	
DALYs	Sierra Leone	Female	Age-standardized	Aortic aneurysm	Rate	2019	15.08739076	24.91330635	10.34700871	-43.57%
DALYs	Sierra Leone	Female	All Ages	Aortic aneurysm	Number	1990	250.0148637	383.9935666	157.2278817	
DALYs	Sierra Leone	Female	All Ages	Aortic aneurysm	Number	2019	288.1692343	483.7551035	193.3433289	15.26%
Deaths	Sierra Leone	Female	Age-standardized	Aortic aneurysm	Rate	1990	1.383052448	2.029831884	0.90507251	
Deaths	Sierra Leone	Female	Age-standardized	Aortic aneurysm	Rate	2019	0.763381931	1.226463659	0.536674883	-44.80%
Deaths	Sierra Leone	Female	All Ages	Aortic aneurysm	Number	1990	11.43596813	17.08915457	7.469451867	
Deaths	Sierra Leone	Female	All Ages	Aortic aneurysm	Number	2019	12.07755222	19.87178963	8.455443645	5.61%
DALYs	Singapore	Female	Age-standardized	Aortic aneurysm	Rate	1990	35.21289546	38.45657932	32.04475593	
DALYs	Singapore	Female	Age-standardized	Aortic aneurysm	Rate	2019	21.18209239	24.26500182	17.65456113	-39.85%
DALYs	Singapore	Female	All Ages	Aortic aneurysm	Number	1990	412.8844821	449.505766	374.8627965	
DALYs	Singapore	Female	All Ages	Aortic aneurysm	Number	2019	841.89168	962.4916424	701.7468494	103.90%
Deaths	Singapore	Female	Age-standardized	Aortic aneurysm	Rate	1990	2.048046594	2.260204875	1.826023288	
Deaths	Singapore	Female	Age-standardized	Aortic aneurysm	Rate	2019	1.392705987	1.621095915	1.112171291	-32.00%
Deaths	Singapore	Female	All Ages	Aortic aneurysm	Number	1990	21.25712882	23.38602016	19.06964859	
Deaths	Singapore	Female	All Ages	Aortic aneurysm	Number	2019	56.11381666	65.26026585	44.67818025	163.98%
DALYs	Slovakia	Female	Age-standardized	Aortic aneurysm	Rate	1990	24.71158628	28.47037839	21.57466985	
DALYs	Slovakia	Female	Age-standardized	Aortic aneurysm	Rate	2019	26.0020134	33.42551507	19.53219223	5.22%
DALYs	Slovakia	Female	All Ages	Aortic aneurysm	Number	1990	813.7202845	932.0428444	710.7388344	
DALYs	Slovakia	Female	All Ages	Aortic aneurysm	Number	2019	1277.314535	1635.128445	977.4063847	56.97%
Deaths	Slovakia	Female	Age-standardized	Aortic aneurysm	Rate	1990	1.09934471	1.251688823	0.961977602	
Deaths	Slovakia	Female	Age-standardized	Aortic aneurysm	Rate	2019	1.229887105	1.55031489	0.950494721	11.87%
Deaths	Slovakia	Female	All Ages	Aortic aneurysm	Number	1990	37.64785292	43.1363626	32.75997163	
Deaths	Slovakia	Female	All Ages	Aortic aneurysm	Number	2019	66.94391005	84.00025548	51.85379533	77.82%
DALYs	Slovenia	Female	Age-standardized	Aortic aneurysm	Rate	1990	28.00042672	36.97954703	21.04245536	
DALYs	Slovenia	Female	Age-standardized	Aortic aneurysm	Rate	2019	28.40621514	37.59326808	21.67279811	1.45%
DALYs	Slovenia	Female	All Ages	Aortic aneurysm	Number	1990	400.6479238	526.603129	301.880838	
DALYs	Slovenia	Female	All Ages	Aortic aneurysm	Number	2019	656.8095217	865.1185637	497.6312608	63.94%
Deaths	Slovenia	Female	Age-standardized	Aortic aneurysm	Rate	1990	1.331140984	1.735900186	1.010983472	
Deaths	Slovenia	Female	Age-standardized	Aortic aneurysm	Rate	2019	1.503776349	1.967951312	1.132257397	12.97%
Deaths	Slovenia	Female	All Ages	Aortic aneurysm	Number	1990	19.90877852	26.22673894	15.00314385	
Deaths	Slovenia	Female	All Ages	Aortic aneurysm	Number	2019	42.58815647	55.81423333	31.960781	113.92%
DALYs	Solomon Islands	Female	Age-standardized	Aortic aneurysm	Rate	1990	58.55250393	113.014062	34.25084485	
DALYs	Solomon Islands	Female	Age-standardized	Aortic aneurysm	Rate	2019	49.68409014	90.38336499	30.03524984	-15.15%
DALYs	Solomon Islands	Female	All Ages	Aortic aneurysm	Number	1990	33.38404164	73.49457814	18.38838347	
DALYs	Solomon Islands	Female	All Ages	Aortic aneurysm	Number	2019	70.69640627	146.2734323	40.5562535	111.77%
Deaths	Solomon Islands	Female	Age-standardized	Aortic aneurysm	Rate	1990	3.024218403	5.206366181	1.882729669	
Deaths	Solomon Islands	Female	Age-standardized	Aortic aneurysm	Rate	2019	2.659176492	4.305756882	1.683729435	-12.07%
Deaths	Solomon Islands	Female	All Ages	Aortic aneurysm	Number	1990	1.254382716	2.473537855	0.717742757	
Deaths	Solomon Islands	Female	All Ages	Aortic aneurysm	Number	2019	2.756972167	5.126072271	1.655075829	119.79%
DALYs	Somalia	Female	Age-standardized	Aortic aneurysm	Rate	1990	43.86476185	74.19097466	18.29761103	
DALYs	Somalia	Female	Age-standardized	Aortic aneurysm	Rate	2019	26.83992799	43.51966003	11.12391171	-38.81%
DALYs	Somalia	Female	All Ages	Aortic aneurysm	Number	1990	580.038424	1102.456242	228.9971616	
DALYs	Somalia	Female	All Ages	Aortic aneurysm	Number	2019	992.8826039	1717.932343	403.454478	71.18%
Deaths	Somalia	Female	Age-standardized	Aortic aneurysm	Rate	1990	2.284552684	3.52746181	0.988731211	
Deaths	Somalia	Female	Age-standardized	Aortic aneurysm	Rate	2019	1.431236084	2.195878884	0.601710661	-37.35%
Deaths	Somalia	Female	All Ages	Aortic aneurysm	Number	1990	22.81737922	38.77955066	9.517416172	
Deaths	Somalia	Female	All Ages	Aortic aneurysm	Number	2019	40.35017886	65.51291916	16.73414864	76.84%
DALYs	South Africa	Female	Age-standardized	Aortic aneurysm	Rate	1990	41.75699608	46.4419845	36.53865908	
DALYs	South Africa	Female	Age-standardized	Aortic aneurysm	Rate	2019	22.55338622	25.53341489	18.6557371	-45.99%
DALYs	South Africa	Female	All Ages	Aortic aneurysm	Number	1990	5378.678328	5971.237642	4763.139289	
DALYs	South Africa	Female	All Ages	Aortic aneurysm	Number	2019	5802.775575	6608.985507	4798.237199	7.88%
Deaths	South Africa	Female	Age-standardized	Aortic aneurysm	Rate	1990	2.211048485	2.489665657	1.840572547	
Deaths	South Africa	Female	Age-standardized	Aortic aneurysm	Rate	2019	1.251111601	1.39975143	1.053006732	-43.42%
Deaths	South Africa	Female	All Ages	Aortic aneurysm	Number	1990	247.5677693	277.4742772	207.9348438	
Deaths	South Africa	Female	All Ages	Aortic aneurysm	Number	2019	294.6754366	330.4026398	248.1282198	19.03%
DALYs	South Sudan	Female	Age-standardized	Aortic aneurysm	Rate	1990	46.28019049	66.09683355	26.85876233	
DALYs	South Sudan	Female	Age-standardized	Aortic aneurysm	Rate	2019	27.39759038	39.13401888	18.01937981	-40.80%
DALYs	South Sudan	Female	All Ages	Aortic aneurysm	Number	1990	505.6094724	729.0197681	293.0229598	
DALYs	South Sudan	Female	All Ages	Aortic aneurysm	Number	2019	499.7363669	738.9160267	322.7684232	-1.16%
Deaths	South Sudan	Female	Age-standardized	Aortic aneurysm	Rate	1990	2.446338648	3.353654504	1.426620436	
Deaths	South Sudan	Female	Age-standardized	Aortic aneurysm	Rate	2019	1.489624269	2.097716218	0.991600131	-39.11%
Deaths	South Sudan	Female	All Ages	Aortic aneurysm	Number	1990	23.09191036	32.24210798	13.40275011	
Deaths	South Sudan	Female	All Ages	Aortic aneurysm	Number	2019	21.23575526	30.35442766	14.13885927	-8.04%
DALYs	Spain	Female	Age-standardized	Aortic aneurysm	Rate	1990	12.71380167	13.63128944	11.7661243	
DALYs	Spain	Female	Age-standardized	Aortic aneurysm	Rate	2019	16.42276528	18.24784202	14.48695902	29.17%
DALYs	Spain	Female	All Ages	Aortic aneurysm	Number	1990	3725.777676	4006.392292	3429.240199	
DALYs	Spain	Female	All Ages	Aortic aneurysm	Number	2019	8320.210258	9400.731478	7194.124151	123.31%
Deaths	Spain	Female	Age-standardized	Aortic aneurysm	Rate	1990	0.645039082	0.697760454	0.582363224	
Deaths	Spain	Female	Age-standardized	Aortic aneurysm	Rate	2019	0.887042559	1.013348445	0.75923603	37.52%
Deaths	Spain	Female	All Ages	Aortic aneurysm	Number	1990	206.2195212	224.2164499	184.820702	
Deaths	Spain	Female	All Ages	Aortic aneurysm	Number	2019	581.4427707	671.8725768	479.3007034	181.95%
DALYs	Sri Lanka	Female	Age-standardized	Aortic aneurysm	Rate	1990	19.14444877	24.06869008	15.99986963	
DALYs	Sri Lanka	Female	Age-standardized	Aortic aneurysm	Rate	2019	19.1206475	25.36187435	13.86006724	-0.12%
DALYs	Sri Lanka	Female	All Ages	Aortic aneurysm	Number	1990	988.5046551	1270.6583	824.6270863	
DALYs	Sri Lanka	Female	All Ages	Aortic aneurysm	Number	2019	2549.41806	3414.277823	1847.933029	157.91%
Deaths	Sri Lanka	Female	Age-standardized	Aortic aneurysm	Rate	1990	1.155593416	1.42079762	0.959711919	
Deaths	Sri Lanka	Female	Age-standardized	Aortic aneurysm	Rate	2019	1.185995842	1.562374836	0.858904646	2.63%
Deaths	Sri Lanka	Female	All Ages	Aortic aneurysm	Number	1990	47.37711329	59.38739017	39.39115761	
Deaths	Sri Lanka	Female	All Ages	Aortic aneurysm	Number	2019	148.194038	196.2640882	107.5195365	212.80%
DALYs	Sudan	Female	Age-standardized	Aortic aneurysm	Rate	1990	9.939745634	18.17260792	6.618005952	
DALYs	Sudan	Female	Age-standardized	Aortic aneurysm	Rate	2019	10.5531511	17.52321631	6.649116461	6.17%
DALYs	Sudan	Female	All Ages	Aortic aneurysm	Number	1990	479.4767708	958.8366143	305.3040761	
DALYs	Sudan	Female	All Ages	Aortic aneurysm	Number	2019	1037.54883	1805.357867	610.1605734	116.39%
Deaths	Sudan	Female	Age-standardized	Aortic aneurysm	Rate	1990	0.481895387	0.785123112	0.331433148	
Deaths	Sudan	Female	Age-standardized	Aortic aneurysm	Rate	2019	0.534291443	0.867161928	0.35585909	10.87%
Deaths	Sudan	Female	All Ages	Aortic aneurysm	Number	1990	18.98242709	33.20916235	12.86122677	
Deaths	Sudan	Female	All Ages	Aortic aneurysm	Number	2019	40.63464722	66.37035109	25.99306281	114.06%
DALYs	Suriname	Female	Age-standardized	Aortic aneurysm	Rate	1990	22.67449402	25.98613983	19.71468283	
DALYs	Suriname	Female	Age-standardized	Aortic aneurysm	Rate	2019	19.95445736	23.95850185	16.27456351	-12.00%
DALYs	Suriname	Female	All Ages	Aortic aneurysm	Number	1990	30.67339736	35.06575601	26.59545914	
DALYs	Suriname	Female	All Ages	Aortic aneurysm	Number	2019	63.41765513	76.04691858	51.71400461	106.75%
Deaths	Suriname	Female	Age-standardized	Aortic aneurysm	Rate	1990	1.214182748	1.391300078	1.052330935	
Deaths	Suriname	Female	Age-standardized	Aortic aneurysm	Rate	2019	1.08172851	1.301992855	0.885125078	-10.91%
Deaths	Suriname	Female	All Ages	Aortic aneurysm	Number	1990	1.605184396	1.836235148	1.395020733	
Deaths	Suriname	Female	All Ages	Aortic aneurysm	Number	2019	3.375764771	4.049722054	2.772174738	110.30%
DALYs	Sweden	Female	Age-standardized	Aortic aneurysm	Rate	1990	63.02357597	66.79125248	58.57195731	
DALYs	Sweden	Female	Age-standardized	Aortic aneurysm	Rate	2019	52.67796298	59.18344632	46.85274795	-16.42%
DALYs	Sweden	Female	All Ages	Aortic aneurysm	Number	1990	5550.891084	5888.451915	5117.947393	
DALYs	Sweden	Female	All Ages	Aortic aneurysm	Number	2019	6132.848085	6959.228163	5353.920954	10.48%
Deaths	Sweden	Female	Age-standardized	Aortic aneurysm	Rate	1990	3.637213877	3.875689781	3.29413969	

Deaths	Sweden	Female	Age-standardized	Aortic aneurysm	Rate	2019	3.261942511	3.738170205	2.824897512	-10.32%
Deaths	Sweden	Female	All Ages	Aortic aneurysm	Number	1990	363.4456403	388.4596254	327.3046749	
Deaths	Sweden	Female	All Ages	Aortic aneurysm	Number	2019	446.9714076	518.7550502	380.0456498	22.98%
DALYs	Switzerland	Female	Age-standardized	Aortic aneurysm	Rate	1990	31.73934201	36.52700534	28.06152806	
DALYs	Switzerland	Female	Age-standardized	Aortic aneurysm	Rate	2019	28.19850068	32.24610246	24.21062901	-11.16%
DALYs	Switzerland	Female	All Ages	Aortic aneurysm	Number	1990	2019.232302	2342.341791	1769.595185	
DALYs	Switzerland	Female	All Ages	Aortic aneurysm	Number	2019	2861.414829	3326.251207	2399.672664	41.71%
Deaths	Switzerland	Female	Age-standardized	Aortic aneurysm	Rate	1990	1.887520135	2.201080797	1.633576339	
Deaths	Switzerland	Female	Age-standardized	Aortic aneurysm	Rate	2019	1.837414907	2.152885665	1.512777009	-2.65%
Deaths	Switzerland	Female	All Ages	Aortic aneurysm	Number	1990	135.2449885	158.5011582	115.3928337	
Deaths	Switzerland	Female	All Ages	Aortic aneurysm	Number	2019	222.5794701	263.1953526	177.6423248	64.58%
DALYs	Syrian Arab Republic	Female	Age-standardized	Aortic aneurysm	Rate	1990	12.37477955	16.25966477	9.540072925	
DALYs	Syrian Arab Republic	Female	Age-standardized	Aortic aneurysm	Rate	2019	11.95413757	15.61340364	9.185674063	-3.40%
DALYs	Syrian Arab Republic	Female	All Ages	Aortic aneurysm	Number	1990	302.9526408	432.5052419	229.6798304	
DALYs	Syrian Arab Republic	Female	All Ages	Aortic aneurysm	Number	2019	652.9268196	874.1695475	488.5362208	115.52%
Deaths	Syrian Arab Republic	Female	Age-standardized	Aortic aneurysm	Rate	1990	0.71969771	0.888316038	0.579878384	
Deaths	Syrian Arab Republic	Female	Age-standardized	Aortic aneurysm	Rate	2019	0.71375709	0.891643448	0.565154126	-0.83%
Deaths	Syrian Arab Republic	Female	All Ages	Aortic aneurysm	Number	1990	14.55450026	18.39984366	11.51086872	
Deaths	Syrian Arab Republic	Female	All Ages	Aortic aneurysm	Number	2019	29.73796894	38.66639954	23.0325693	104.32%
DALYs	Taiwan (Province of China)	Female	Age-standardized	Aortic aneurysm	Rate	1990	16.15206534	17.57064719	14.81523944	
DALYs	Taiwan (Province of China)	Female	Age-standardized	Aortic aneurysm	Rate	2019	29.48120183	37.80505953	22.88687882	82.52%
DALYs	Taiwan (Province of China)	Female	All Ages	Aortic aneurysm	Number	1990	1203.51871	1304.886776	1104.013756	
DALYs	Taiwan (Province of China)	Female	All Ages	Aortic aneurysm	Number	2019	6070.565297	7774.62767	4700.139609	404.40%
Deaths	Taiwan (Province of China)	Female	Age-standardized	Aortic aneurysm	Rate	1990	0.854599489	0.939396968	0.774680015	
Deaths	Taiwan (Province of China)	Female	Age-standardized	Aortic aneurysm	Rate	2019	1.653974773	2.10466043	1.289000473	93.54%
Deaths	Taiwan (Province of China)	Female	All Ages	Aortic aneurysm	Number	1990	54.78278806	59.91122648	50.03284661	
Deaths	Taiwan (Province of China)	Female	All Ages	Aortic aneurysm	Number	2019	360.5087865	459.380241	280.5828072	558.07%
DALYs	Tajikistan	Female	Age-standardized	Aortic aneurysm	Rate	1990	42.03883544	49.36515941	33.54676442	
DALYs	Tajikistan	Female	Age-standardized	Aortic aneurysm	Rate	2019	52.29053218	64.76576563	42.59179367	24.39%
DALYs	Tajikistan	Female	All Ages	Aortic aneurysm	Number	1990	653.5902092	769.3072567	524.6319963	
DALYs	Tajikistan	Female	All Ages	Aortic aneurysm	Number	2019	1192.500111	1507.995705	947.322497	82.45%
Deaths	Tajikistan	Female	Age-standardized	Aortic aneurysm	Rate	1990	2.428436353	2.925339971	1.612764405	
Deaths	Tajikistan	Female	Age-standardized	Aortic aneurysm	Rate	2019	3.073922224	3.718600339	2.508000958	26.58%
Deaths	Tajikistan	Female	All Ages	Aortic aneurysm	Number	1990	38.25813502	46.37757271	25.22728255	
Deaths	Tajikistan	Female	All Ages	Aortic aneurysm	Number	2019	53.35448538	65.84127835	43.42814562	39.46%
DALYs	Thailand	Female	Age-standardized	Aortic aneurysm	Rate	1990	23.42948012	29.45053274	18.66455583	
DALYs	Thailand	Female	Age-standardized	Aortic aneurysm	Rate	2019	19.09184491	24.98662693	14.23417737	-18.51%
DALYs	Thailand	Female	All Ages	Aortic aneurysm	Number	1990	3944.318929	4948.023093	3167.323806	
DALYs	Thailand	Female	All Ages	Aortic aneurysm	Number	2019	10408.7572	13675.36363	7771.1082	163.89%
Deaths	Thailand	Female	Age-standardized	Aortic aneurysm	Rate	1990	1.633606802	2.038485429	1.279312955	
Deaths	Thailand	Female	Age-standardized	Aortic aneurysm	Rate	2019	1.329818079	1.74103425	0.969110034	-18.60%
Deaths	Thailand	Female	All Ages	Aortic aneurysm	Number	1990	234.3918177	289.9269314	186.8278006	
Deaths	Thailand	Female	All Ages	Aortic aneurysm	Number	2019	741.8527233	971.4424424	538.9242703	216.50%
DALYs	Timor-Leste	Female	Age-standardized	Aortic aneurysm	Rate	1990	12.04709341	20.57144668	8.716257631	
DALYs	Timor-Leste	Female	Age-standardized	Aortic aneurysm	Rate	2019	18.45074238	25.3936881	13.70226458	53.16%
DALYs	Timor-Leste	Female	All Ages	Aortic aneurysm	Number	1990	14.97384011	28.68711059	10.34534578	
DALYs	Timor-Leste	Female	All Ages	Aortic aneurysm	Number	2019	71.88341666	98.06830127	52.14857011	380.06%
Deaths	Timor-Leste	Female	Age-standardized	Aortic aneurysm	Rate	1990	0.709208267	1.123196497	0.51830259	
Deaths	Timor-Leste	Female	Age-standardized	Aortic aneurysm	Rate	2019	1.067649983	1.486377073	0.81691672	50.54%
Deaths	Timor-Leste	Female	All Ages	Aortic aneurysm	Number	1990	0.67148724	1.140464169	0.484733605	
Deaths	Timor-Leste	Female	All Ages	Aortic aneurysm	Number	2019	3.465652954	4.792941404	2.624704628	416.12%
DALYs	Togo	Female	Age-standardized	Aortic aneurysm	Rate	1990	35.3759315	46.76543598	24.50229008	
DALYs	Togo	Female	Age-standardized	Aortic aneurysm	Rate	2019	17.90510399	24.60327262	13.24379759	-49.39%
DALYs	Togo	Female	All Ages	Aortic aneurysm	Number	1990	240.9796516	325.7050923	162.6397315	
DALYs	Togo	Female	All Ages	Aortic aneurysm	Number	2019	386.7173953	539.2002604	277.6519609	60.48%
Deaths	Togo	Female	Age-standardized	Aortic aneurysm	Rate	1990	1.777152226	2.324059189	1.228039389	
Deaths	Togo	Female	Age-standardized	Aortic aneurysm	Rate	2019	0.910426228	1.20851281	0.691560499	-48.77%
Deaths	Togo	Female	All Ages	Aortic aneurysm	Number	1990	9.742825685	12.92406535	6.793631862	
Deaths	Togo	Female	All Ages	Aortic aneurysm	Number	2019	15.73429111	21.43713781	11.73942612	61.50%
DALYs	Tokelau	Female	Age-standardized	Aortic aneurysm	Rate	1990	63.65095585	101.5539545	45.57012803	
DALYs	Tokelau	Female	Age-standardized	Aortic aneurysm	Rate	2019	52.52264136	71.22188381	38.46696173	-17.48%
DALYs	Tokelau	Female	All Ages	Aortic aneurysm	Number	1990	0.454586451	0.717586364	0.326864271	
DALYs	Tokelau	Female	All Ages	Aortic aneurysm	Number	2019	0.335325734	0.464272767	0.242412747	-26.23%
Deaths	Tokelau	Female	Age-standardized	Aortic aneurysm	Rate	1990	3.421494477	5.193280564	2.495603249	
Deaths	Tokelau	Female	Age-standardized	Aortic aneurysm	Rate	2019	2.838338276	3.728676396	2.131050987	-17.04%
Deaths	Tokelau	Female	All Ages	Aortic aneurysm	Number	1990	0.023716943	0.036479092	0.017178483	
Deaths	Tokelau	Female	All Ages	Aortic aneurysm	Number	2019	0.017150071	0.022624931	0.012774832	-27.69%
DALYs	Tonga	Female	Age-standardized	Aortic aneurysm	Rate	1990	8.12904311	10.14962694	6.514491764	
DALYs	Tonga	Female	Age-standardized	Aortic aneurysm	Rate	2019	7.846658114	10.57236717	5.888657504	-3.47%
DALYs	Tonga	Female	All Ages	Aortic aneurysm	Number	1990	2.315555645	2.912316884	1.840766339	
DALYs	Tonga	Female	All Ages	Aortic aneurysm	Number	2019	3.37766654	4.553262827	2.547421747	45.87%
Deaths	Tonga	Female	Age-standardized	Aortic aneurysm	Rate	1990	0.436924483	0.540562231	0.348906106	
Deaths	Tonga	Female	Age-standardized	Aortic aneurysm	Rate	2019	0.420389557	0.550346353	0.322324575	-3.78%
Deaths	Tonga	Female	All Ages	Aortic aneurysm	Number	1990	0.108440248	0.133998206	0.087449696	
Deaths	Tonga	Female	All Ages	Aortic aneurysm	Number	2019	0.185634954	0.243093313	0.142545506	71.19%
DALYs	Trinidad and Tobago	Female	Age-standardized	Aortic aneurysm	Rate	1990	54.84406112	62.85628876	48.89221924	
DALYs	Trinidad and Tobago	Female	Age-standardized	Aortic aneurysm	Rate	2019	52.27207562	69.37018069	38.91992498	-4.69%
DALYs	Trinidad and Tobago	Female	All Ages	Aortic aneurysm	Number	1990	244.7747829	279.5918401	218.5821365	
DALYs	Trinidad and Tobago	Female	All Ages	Aortic aneurysm	Number	2019	497.8407552	657.3662783	370.9831547	103.39%
Deaths	Trinidad and Tobago	Female	Age-standardized	Aortic aneurysm	Rate	1990	2.837561789	3.292844282	2.488801738	
Deaths	Trinidad and Tobago	Female	Age-standardized	Aortic aneurysm	Rate	2019	2.759146353	3.592680914	2.086638928	-2.76%
Deaths	Trinidad and Tobago	Female	All Ages	Aortic aneurysm	Number	1990	12.09992856	13.97644554	10.60477692	
Deaths	Trinidad and Tobago	Female	All Ages	Aortic aneurysm	Number	2019	26.44473781	34.4840021	20.02028315	118.55%
DALYs	Tunisia	Female	Age-standardized	Aortic aneurysm	Rate	1990	12.3454128	15.35152181	9.92286634	
DALYs	Tunisia	Female	Age-standardized	Aortic aneurysm	Rate	2019	11.65363508	15.77454814	8.356163186	-5.60%
DALYs	Tunisia	Female	All Ages	Aortic aneurysm	Number	1990	309.2695525	386.1558228	247.5895325	
DALYs	Tunisia	Female	All Ages	Aortic aneurysm	Number	2019	743.576587	1005.793856	531.1821552	140.43%
Deaths	Tunisia	Female	Age-standardized	Aortic aneurysm	Rate	1990	0.632312586	0.775871718	0.508845654	
Deaths	Tunisia	Female	Age-standardized	Aortic aneurysm	Rate	2019	0.596411216	0.792543829	0.429272852	-5.68%
Deaths	Tunisia	Female	All Ages	Aortic aneurysm	Number	1990	12.87906895	15.97406917	10.38142578	
Deaths	Tunisia	Female	All Ages	Aortic aneurysm	Number	2019	36.01805762	47.90514131	25.82068056	179.66%
DALYs	Turkey	Female	Age-standardized	Aortic aneurysm	Rate	1990	29.86275304	40.53167022	23.71944253	
DALYs	Turkey	Female	Age-standardized	Aortic aneurysm	Rate	2019	23.44769102	29.17697108	18.47061638	-21.48%
DALYs	Turkey	Female	All Ages	Aortic aneurysm	Number	1990	5760.823285	7898.011062	4561.971958	
DALYs	Turkey	Female	All Ages	Aortic aneurysm	Number	2019	10946.68122	13670.34961	8613.296213	90.02%
Deaths	Turkey	Female	Age-standardized	Aortic aneurysm	Rate	1990	1.395903121	1.850467824	1.120380606	
Deaths	Turkey	Female	Age-standardized	Aortic aneurysm	Rate	2019	1.148419705	1.434656568	0.911119331	-17.73%
Deaths	Turkey	Female	All Ages	Aortic aneurysm	Number	1990	244.1641507	324.8319154	195.6742159	
Deaths	Turkey	Female	All Ages	Aortic aneurysm	Number	2019	526.1794957	656.9125716	416.901434	115.50%
DALYs	Turkmenistan	Female	Age-standardized	Aortic aneurysm	Rate	1990	13.14015465	14.33228269	12.0676599	
DALYs	Turkmenistan	Female	Age-standardized	Aortic aneurysm	Rate	2019	26.88380728	34.28500626	20.43060471	104.59%

DALYs	Turkmenistan	Female	All Ages	Aortic aneurysm	Number	1990	146.282743	159.4068202	134.835331	
DALYs	Turkmenistan	Female	All Ages	Aortic aneurysm	Number	2019	581.6223743	749.5547245	436.962156	297.60%
Deaths	Turkmenistan	Female	Age-standardized	Aortic aneurysm	Rate	1990	0.662402109	0.729246462	0.597030452	
Deaths	Turkmenistan	Female	Age-standardized	Aortic aneurysm	Rate	2019	1.331746607	1.664502612	1.05107987	101.05%
Deaths	Turkmenistan	Female	All Ages	Aortic aneurysm	Number	1990	6.631905302	7.277446041	6.046318756	
Deaths	Turkmenistan	Female	All Ages	Aortic aneurysm	Number	2019	26.063573	32.64407567	20.40926005	293.00%
DALYs	Tuvalu	Female	Age-standardized	Aortic aneurysm	Rate	1990	63.14168682	98.36353979	45.78210939	
DALYs	Tuvalu	Female	Age-standardized	Aortic aneurysm	Rate	2019	49.29531561	69.16638722	35.53827111	-21.93%
DALYs	Tuvalu	Female	All Ages	Aortic aneurysm	Number	1990	2.300951966	3.7245597	1.627650663	
DALYs	Tuvalu	Female	All Ages	Aortic aneurysm	Number	2019	2.548531292	3.583185079	1.821191146	10.76%
Deaths	Tuvalu	Female	Age-standardized	Aortic aneurysm	Rate	1990	3.443921244	5.01746464	2.519729485	
Deaths	Tuvalu	Female	Age-standardized	Aortic aneurysm	Rate	2019	2.705334966	3.727099471	1.996181965	-21.45%
Deaths	Tuvalu	Female	All Ages	Aortic aneurysm	Number	1990	0.103065799	0.158415973	0.074102696	
Deaths	Tuvalu	Female	All Ages	Aortic aneurysm	Number	2019	0.123165645	0.170264898	0.090047516	19.50%
DALYs	Uganda	Female	Age-standardized	Aortic aneurysm	Rate	1990	34.56044651	49.80578373	14.97350969	
DALYs	Uganda	Female	Age-standardized	Aortic aneurysm	Rate	2019	28.56642316	38.7432883	14.82027258	-17.34%
DALYs	Uganda	Female	All Ages	Aortic aneurysm	Number	1990	1140.232977	1642.182249	487.812952	
DALYs	Uganda	Female	All Ages	Aortic aneurysm	Number	2019	2316.159354	3188.36955	1185.399199	103.13%
Deaths	Uganda	Female	Age-standardized	Aortic aneurysm	Rate	1990	1.919579994	2.778961487	0.842953317	
Deaths	Uganda	Female	Age-standardized	Aortic aneurysm	Rate	2019	1.574609389	2.129453388	0.838725768	-17.97%
Deaths	Uganda	Female	All Ages	Aortic aneurysm	Number	1990	52.19659302	75.2080497	22.77361563	
Deaths	Uganda	Female	All Ages	Aortic aneurysm	Number	2019	103.5878348	139.4696124	54.66184535	98.46%
DALYs	Ukraine	Female	Age-standardized	Aortic aneurysm	Rate	1990	20.53254451	24.12909099	17.77006983	
DALYs	Ukraine	Female	Age-standardized	Aortic aneurysm	Rate	2019	29.47873512	37.47962884	22.8613376	43.57%
DALYs	Ukraine	Female	All Ages	Aortic aneurysm	Number	1990	8979.520701	10563.19706	7775.652141	
DALYs	Ukraine	Female	All Ages	Aortic aneurysm	Number	2019	12426.81206	15642.69442	9703.869406	38.39%
Deaths	Ukraine	Female	Age-standardized	Aortic aneurysm	Rate	1990	0.908756065	1.065896408	0.7896399	
Deaths	Ukraine	Female	Age-standardized	Aortic aneurysm	Rate	2019	1.212026823	1.501905893	0.956913053	33.37%
Deaths	Ukraine	Female	All Ages	Aortic aneurysm	Number	1990	415.1424896	489.1994751	360.4295572	
Deaths	Ukraine	Female	All Ages	Aortic aneurysm	Number	2019	576.1136861	714.1630367	459.5350361	38.77%
DALYs	United Arab Emirates	Female	Age-standardized	Aortic aneurysm	Rate	1990	13.24619591	27.16107429	8.471972396	
DALYs	United Arab Emirates	Female	Age-standardized	Aortic aneurysm	Rate	2019	10.57368477	22.2006435	6.671000042	-20.18%
DALYs	United Arab Emirates	Female	All Ages	Aortic aneurysm	Number	1990	28.28269292	51.65066969	17.63737211	
DALYs	United Arab Emirates	Female	All Ages	Aortic aneurysm	Number	2019	173.7685413	371.0883866	103.9064961	514.40%
Deaths	United Arab Emirates	Female	Age-standardized	Aortic aneurysm	Rate	1990	0.656776991	1.390757928	0.421187645	
Deaths	United Arab Emirates	Female	Age-standardized	Aortic aneurysm	Rate	2019	0.488592916	1.031918233	0.307009934	-25.61%
Deaths	United Arab Emirates	Female	All Ages	Aortic aneurysm	Number	1990	0.796693368	1.523109124	0.508335186	
Deaths	United Arab Emirates	Female	All Ages	Aortic aneurysm	Number	2019	4.419616967	9.411240779	2.636579075	454.75%
DALYs	United Kingdom	Female	Age-standardized	Aortic aneurysm	Rate	1990	86.66056136	89.51142471	81.76606578	
DALYs	United Kingdom	Female	Age-standardized	Aortic aneurysm	Rate	2019	57.36842182	61.93288929	51.77506535	-33.80%
DALYs	United Kingdom	Female	All Ages	Aortic aneurysm	Number	1990	50262.25062	52007.39673	47039.43239	
DALYs	United Kingdom	Female	All Ages	Aortic aneurysm	Number	2019	43058.18317	46533.49112	38225.0552	-14.33%
Deaths	United Kingdom	Female	Age-standardized	Aortic aneurysm	Rate	1990	5.578580738	5.812700928	5.121071654	
Deaths	United Kingdom	Female	Age-standardized	Aortic aneurysm	Rate	2019	4.049344111	4.396072542	3.54092327	-27.41%
Deaths	United Kingdom	Female	All Ages	Aortic aneurysm	Number	1990	3468.155122	3620.014258	3169.677053	
Deaths	United Kingdom	Female	All Ages	Aortic aneurysm	Number	2019	3438.495958	3754.062483	2966.916487	-0.86%
DALYs	United Republic of Tanzania	Female	Age-standardized	Aortic aneurysm	Rate	1990	50.35573603	67.19172225	27.3910026	
DALYs	United Republic of Tanzania	Female	Age-standardized	Aortic aneurysm	Rate	2019	39.09852174	52.1246072	25.27736151	-22.36%
DALYs	United Republic of Tanzania	Female	All Ages	Aortic aneurysm	Number	1990	2754.329597	3744.32946	1456.627914	
DALYs	United Republic of Tanzania	Female	All Ages	Aortic aneurysm	Number	2019	5045.032893	6794.397327	3260.25525	83.17%
Deaths	United Republic of Tanzania	Female	Age-standardized	Aortic aneurysm	Rate	1990	2.784859844	3.666869461	1.52726135	
Deaths	United Republic of Tanzania	Female	Age-standardized	Aortic aneurysm	Rate	2019	2.148023678	2.841142917	1.384181102	-22.87%
Deaths	United Republic of Tanzania	Female	All Ages	Aortic aneurysm	Number	1990	124.8262591	165.1801157	68.26718888	
Deaths	United Republic of Tanzania	Female	All Ages	Aortic aneurysm	Number	2019	236.196767	312.1549014	152.8599197	89.22%
DALYs	United States of America	Female	Age-standardized	Aortic aneurysm	Rate	1990	48.90501095	50.93249424	45.83431979	
DALYs	United States of America	Female	Age-standardized	Aortic aneurysm	Rate	2019	30.05092481	32.94210714	27.68477561	-38.55%
DALYs	United States of America	Female	All Ages	Aortic aneurysm	Number	1990	92977.73254	96995.98935	86447.18899	
DALYs	United States of America	Female	All Ages	Aortic aneurysm	Number	2019	89655.02701	98459.83706	81538.78148	-3.57%
Deaths	United States of America	Female	Age-standardized	Aortic aneurysm	Rate	1990	2.737027427	2.87820812	2.486231587	
Deaths	United States of America	Female	Age-standardized	Aortic aneurysm	Rate	2019	1.720456669	1.895159351	1.53132322	-37.14%
Deaths	United States of America	Female	All Ages	Aortic aneurysm	Number	1990	5711.231863	6014.954363	5157.074864	
Deaths	United States of America	Female	All Ages	Aortic aneurysm	Number	2019	5936.457519	6542.602359	5170.927267	3.94%
DALYs	United States Virgin Islands	Female	Age-standardized	Aortic aneurysm	Rate	1990	41.65043109	49.35082591	34.48289703	
DALYs	United States Virgin Islands	Female	Age-standardized	Aortic aneurysm	Rate	2019	32.44569063	39.81737618	26.82562146	-22.10%
DALYs	United States Virgin Islands	Female	All Ages	Aortic aneurysm	Number	1990	18.43627553	21.90054957	15.17457658	
DALYs	United States Virgin Islands	Female	All Ages	Aortic aneurysm	Number	2019	33.77846014	41.9141535	27.80588914	83.22%
Deaths	United States Virgin Islands	Female	Age-standardized	Aortic aneurysm	Rate	1990	2.316897739	2.717016749	1.928801871	
Deaths	United States Virgin Islands	Female	Age-standardized	Aortic aneurysm	Rate	2019	1.953826941	2.409553824	1.612612199	-15.67%
Deaths	United States Virgin Islands	Female	All Ages	Aortic aneurysm	Number	1990	0.918073337	1.078245541	0.757809412	
Deaths	United States Virgin Islands	Female	All Ages	Aortic aneurysm	Number	2019	1.990842667	2.444530059	1.64140979	116.85%
DALYs	Uruguay	Female	Age-standardized	Aortic aneurysm	Rate	1990	62.4925527	68.88189523	56.83601287	
DALYs	Uruguay	Female	Age-standardized	Aortic aneurysm	Rate	2019	56.93685553	63.1518911	50.99332705	-8.89%
DALYs	Uruguay	Female	All Ages	Aortic aneurysm	Number	1990	1327.363733	1471.043782	1203.782883	
DALYs	Uruguay	Female	All Ages	Aortic aneurysm	Number	2019	1662.361585	1850.089537	1471.864622	25.24%
Deaths	Uruguay	Female	Age-standardized	Aortic aneurysm	Rate	1990	2.9804267	3.309346383	2.68475061	
Deaths	Uruguay	Female	Age-standardized	Aortic aneurysm	Rate	2019	2.778528897	3.110342467	2.445911026	-6.77%
Deaths	Uruguay	Female	All Ages	Aortic aneurysm	Number	1990	67.71735642	75.41681714	60.54113243	
Deaths	Uruguay	Female	All Ages	Aortic aneurysm	Number	2019	96.24115966	108.7462159	83.92018664	42.12%
DALYs	Uzbekistan	Female	Age-standardized	Aortic aneurysm	Rate	1990	7.424876287	8.764422227	3.87734494	
DALYs	Uzbekistan	Female	Age-standardized	Aortic aneurysm	Rate	2019	20.96927466	24.5860639	17.72991092	182.42%
DALYs	Uzbekistan	Female	All Ages	Aortic aneurysm	Number	1990	485.0022223	571.1245326	259.5389388	
DALYs	Uzbekistan	Female	All Ages	Aortic aneurysm	Number	2019	1945.628872	2348.375285	1587.736295	301.16%
Deaths	Uzbekistan	Female	Age-standardized	Aortic aneurysm	Rate	1990	0.443610024	0.532343942	0.204100024	
Deaths	Uzbekistan	Female	Age-standardized	Aortic aneurysm	Rate	2019	1.308411019	1.5234972	1.116592686	194.95%
Deaths	Uzbekistan	Female	All Ages	Aortic aneurysm	Number	1990	27.35036796	32.73497984	12.78661932	
Deaths	Uzbekistan	Female	All Ages	Aortic aneurysm	Number	2019	83.44406175	99.366819	69.35312219	205.09%
DALYs	Vanuatu	Female	Age-standardized	Aortic aneurysm	Rate	1990	44.16229095	74.04448769	28.11199567	
DALYs	Vanuatu	Female	Age-standardized	Aortic aneurysm	Rate	2019	40.36930909	65.8436667	26.49193592	-8.59%
DALYs	Vanuatu	Female	All Ages	Aortic aneurysm	Number	1990	11.97140984	21.5109407	7.341184231	
DALYs	Vanuatu	Female	All Ages	Aortic aneurysm	Number	2019	32.22460308	53.21038562	20.63149878	169.18%
Deaths	Vanuatu	Female	Age-standardized	Aortic aneurysm	Rate	1990	2.517988242	3.948264498	1.647531731	
Deaths	Vanuatu	Female	Age-standardized	Aortic aneurysm	Rate	2019	2.169228692	3.302876813	1.445258781	-13.85%
Deaths	Vanuatu	Female	All Ages	Aortic aneurysm	Number	1990	0.514061472	0.846860296	0.328032616	
Deaths	Vanuatu	Female	All Ages	Aortic aneurysm	Number	2019	1.36294677	2.18994989	0.896409723	165.13%
DALYs	Venezuela (Bolivarian Republic of)	Female	Age-standardized	Aortic aneurysm	Rate	1990	20.71720542	23.46041566	18.38109421	
DALYs	Venezuela (Bolivarian Republic of)	Female	Age-standardized	Aortic aneurysm	Rate	2019	23.70624126	31.03887137	17.99294741	14.43%
DALYs	Venezuela (Bolivarian Republic of)	Female	All Ages	Aortic aneurysm	Number	1990	1100.797808	1242.030184	981.7291306	
DALYs	Venezuela (Bolivarian Republic of)	Female	All Ages	Aortic aneurysm	Number	2019	3589.05819	4712.812782	2724.875708	226.04%
Deaths	Venezuela (Bolivarian Republic of)	Female	Age-standardized	Aortic aneurysm	Rate	1990	1.075691883	1.236254071	0.940880528	
Deaths	Venezuela (Bolivarian Republic of)	Female	Age-standardized	Aortic aneurysm	Rate	2019	1.28434139	1.658852455	0.980646832	19.40%
Deaths	Venezuela (Bolivarian Republic of)	Female	All Ages	Aortic aneurysm	Number	1990	51.07984711	58.38311678	44.95141615	

Deaths	Venezuela (Bolivarian Republic of)	Female	All Ages	Aortic aneurysm	Number	2019	191.4733007	246.5676062	147.0629169	274.85%
DALYs	Viet Nam	Female	Age-standardized	Aortic aneurysm	Rate	1990	18.01532528	22.57765832	13.93397889	
DALYs	Viet Nam	Female	Age-standardized	Aortic aneurysm	Rate	2019	18.02421311	23.27814227	12.9551416	0.05%
DALYs	Viet Nam	Female	All Ages	Aortic aneurysm	Number	1990	4152.841207	5207.465427	3210.361051	
DALYs	Viet Nam	Female	All Ages	Aortic aneurysm	Number	2019	9184.345099	11934.58955	6608.009291	121.16%
Deaths	Viet Nam	Female	Age-standardized	Aortic aneurysm	Rate	1990	1.070936609	1.344754875	0.833868854	
Deaths	Viet Nam	Female	Age-standardized	Aortic aneurysm	Rate	2019	1.071729521	1.360034958	0.787064309	0.07%
Deaths	Viet Nam	Female	All Ages	Aortic aneurysm	Number	1990	233.1574576	291.3088453	183.4072137	
Deaths	Viet Nam	Female	All Ages	Aortic aneurysm	Number	2019	517.6036695	656.5308574	379.4585094	122.00%
DALYs	Yemen	Female	Age-standardized	Aortic aneurysm	Rate	1990	10.64204054	20.3307747	7.133212658	
DALYs	Yemen	Female	Age-standardized	Aortic aneurysm	Rate	2019	11.38646745	18.07727336	7.924201568	7.00%
DALYs	Yemen	Female	All Ages	Aortic aneurysm	Number	1990	276.8435667	573.6173475	179.2289536	
DALYs	Yemen	Female	All Ages	Aortic aneurysm	Number	2019	830.3710739	1429.198012	549.4863224	199.94%
Deaths	Yemen	Female	Age-standardized	Aortic aneurysm	Rate	1990	0.53246614	0.895297633	0.375170961	
Deaths	Yemen	Female	Age-standardized	Aortic aneurysm	Rate	2019	0.579637761	0.842146677	0.420877738	8.86%
Deaths	Yemen	Female	All Ages	Aortic aneurysm	Number	1990	11.47264415	20.91589212	7.844184867	
Deaths	Yemen	Female	All Ages	Aortic aneurysm	Number	2019	32.87825954	50.85949666	23.29856311	186.58%
DALYs	Zambia	Female	Age-standardized	Aortic aneurysm	Rate	1990	42.52651758	61.8460228	26.28181183	
DALYs	Zambia	Female	Age-standardized	Aortic aneurysm	Rate	2019	40.24196149	53.1966925	28.68618788	-5.37%
DALYs	Zambia	Female	All Ages	Aortic aneurysm	Number	1990	577.1830856	914.236554	349.2977342	
DALYs	Zambia	Female	All Ages	Aortic aneurysm	Number	2019	1415.373715	1881.919864	1002.746318	145.22%
Deaths	Zambia	Female	Age-standardized	Aortic aneurysm	Rate	1990	2.340799358	3.186937211	1.490420748	
Deaths	Zambia	Female	Age-standardized	Aortic aneurysm	Rate	2019	2.237264844	2.930416439	1.600302341	-4.42%
Deaths	Zambia	Female	All Ages	Aortic aneurysm	Number	1990	23.96695047	34.57570945	14.90603889	
Deaths	Zambia	Female	All Ages	Aortic aneurysm	Number	2019	61.89549523	81.57808982	44.27853616	158.25%
DALYs	Zimbabwe	Female	Age-standardized	Aortic aneurysm	Rate	1990	30.07077264	41.17153779	22.98914637	
DALYs	Zimbabwe	Female	Age-standardized	Aortic aneurysm	Rate	2019	31.56617545	46.89553781	21.79441425	4.97%
DALYs	Zimbabwe	Female	All Ages	Aortic aneurysm	Number	1990	682.2841418	938.4234798	514.6347008	
DALYs	Zimbabwe	Female	All Ages	Aortic aneurysm	Number	2019	1407.293365	2141.717797	947.2745211	106.26%
Deaths	Zimbabwe	Female	Age-standardized	Aortic aneurysm	Rate	1990	1.373725522	1.820563778	1.071860087	
Deaths	Zimbabwe	Female	Age-standardized	Aortic aneurysm	Rate	2019	1.40615251	1.972790087	1.018193392	2.36%
Deaths	Zimbabwe	Female	All Ages	Aortic aneurysm	Number	1990	25.91666795	34.93611408	20.1299979	
Deaths	Zimbabwe	Female	All Ages	Aortic aneurysm	Number	2019	50.93866042	73.91693911	35.72575199	96.55%
DALYs	Afghanistan	Male	Age-standardized	Aortic aneurysm	Rate	1990	34.44717247	62.77917834	14.90413449	
DALYs	Afghanistan	Male	Age-standardized	Aortic aneurysm	Rate	2019	31.07820731	51.08490937	16.34009584	-9.78%
DALYs	Afghanistan	Male	All Ages	Aortic aneurysm	Number	1990	1287.567226	2371.750109	557.567232	
DALYs	Afghanistan	Male	All Ages	Aortic aneurysm	Number	2019	2271.452719	3922.082873	1125.637334	76.41%
Deaths	Afghanistan	Male	Age-standardized	Aortic aneurysm	Rate	1990	1.522067694	2.697836176	0.656690996	
Deaths	Afghanistan	Male	Age-standardized	Aortic aneurysm	Rate	2019	1.422603254	2.221776687	0.770074238	-6.53%
Deaths	Afghanistan	Male	All Ages	Aortic aneurysm	Number	1990	51.17666064	91.65638857	22.14127113	
Deaths	Afghanistan	Male	All Ages	Aortic aneurysm	Number	2019	76.87329501	126.4828226	40.38097009	50.21%
DALYs	Albania	Male	Age-standardized	Aortic aneurysm	Rate	1990	37.01614839	41.06701522	33.08991253	
DALYs	Albania	Male	Age-standardized	Aortic aneurysm	Rate	2019	33.57027737	46.21414029	24.16871907	-9.31%
DALYs	Albania	Male	All Ages	Aortic aneurysm	Number	1990	390.9228946	432.6185454	349.616107	
DALYs	Albania	Male	All Ages	Aortic aneurysm	Number	2019	644.9887852	893.7022865	464.4109718	64.99%
Deaths	Albania	Male	Age-standardized	Aortic aneurysm	Rate	1990	1.811712268	2.039491707	1.601870143	
Deaths	Albania	Male	Age-standardized	Aortic aneurysm	Rate	2019	1.639682583	2.216418759	1.19697213	-9.50%
Deaths	Albania	Male	All Ages	Aortic aneurysm	Number	1990	15.23368228	17.01694566	13.54023942	
Deaths	Albania	Male	All Ages	Aortic aneurysm	Number	2019	31.44332694	42.72685632	22.885172	106.41%
DALYs	Algeria	Male	Age-standardized	Aortic aneurysm	Rate	1990	36.39794941	53.88706745	22.41809804	
DALYs	Algeria	Male	Age-standardized	Aortic aneurysm	Rate	2019	29.11374342	43.79765307	20.24906427	-20.01%
DALYs	Algeria	Male	All Ages	Aortic aneurysm	Number	1990	2260.939922	3393.690679	1348.201831	
DALYs	Algeria	Male	All Ages	Aortic aneurysm	Number	2019	4980.068031	7599.584193	3403.511513	120.27%
Deaths	Algeria	Male	Age-standardized	Aortic aneurysm	Rate	1990	1.906038686	2.747119778	1.201342589	
Deaths	Algeria	Male	Age-standardized	Aortic aneurysm	Rate	2019	1.564282673	2.216243803	1.123359051	-17.93%
Deaths	Algeria	Male	All Ages	Aortic aneurysm	Number	1990	90.60829841	133.2621627	55.36099148	
Deaths	Algeria	Male	All Ages	Aortic aneurysm	Number	2019	227.2838935	334.1499715	160.1287828	150.84%
DALYs	American Samoa	Male	Age-standardized	Aortic aneurysm	Rate	1990	88.73816348	108.303677	72.47762539	
DALYs	American Samoa	Male	Age-standardized	Aortic aneurysm	Rate	2019	62.1344601	73.47881183	51.10157048	-29.98%
DALYs	American Samoa	Male	All Ages	Aortic aneurysm	Number	1990	11.29247893	13.90127302	9.091622634	
DALYs	American Samoa	Male	All Ages	Aortic aneurysm	Number	2019	14.27503407	17.06864878	11.64278136	26.41%
Deaths	American Samoa	Male	Age-standardized	Aortic aneurysm	Rate	1990	4.124328169	4.97881134	3.396640859	
Deaths	American Samoa	Male	Age-standardized	Aortic aneurysm	Rate	2019	2.983413496	3.480757818	2.522863695	-27.66%
Deaths	American Samoa	Male	All Ages	Aortic aneurysm	Number	1990	0.405237473	0.493424193	0.3313175	
Deaths	American Samoa	Male	All Ages	Aortic aneurysm	Number	2019	0.595055464	0.694206145	0.497551093	46.84%
DALYs	Andorra	Male	Age-standardized	Aortic aneurysm	Rate	1990	176.7487781	285.3835729	117.6737077	
DALYs	Andorra	Male	Age-standardized	Aortic aneurysm	Rate	2019	124.9800281	194.168114	83.57178015	-29.29%
DALYs	Andorra	Male	All Ages	Aortic aneurysm	Number	1990	49.8235027	81.59441194	32.67657022	
DALYs	Andorra	Male	All Ages	Aortic aneurysm	Number	2019	85.52797553	133.2587499	57.06834586	71.66%
Deaths	Andorra	Male	Age-standardized	Aortic aneurysm	Rate	1990	9.918392838	15.29232769	6.768614533	
Deaths	Andorra	Male	Age-standardized	Aortic aneurysm	Rate	2019	7.241695025	10.82468247	4.973294109	-26.99%
Deaths	Andorra	Male	All Ages	Aortic aneurysm	Number	1990	2.420746887	3.870806937	1.633026471	
Deaths	Andorra	Male	All Ages	Aortic aneurysm	Number	2019	4.95100256	7.394584689	3.391714732	104.52%
DALYs	Angola	Male	Age-standardized	Aortic aneurysm	Rate	1990	106.1332819	177.2271853	49.16078374	
DALYs	Angola	Male	Age-standardized	Aortic aneurysm	Rate	2019	89.7274633	118.3346809	61.79545085	-15.46%
DALYs	Angola	Male	All Ages	Aortic aneurysm	Number	1990	2218.749696	3828.583316	1044.260491	
DALYs	Angola	Male	All Ages	Aortic aneurysm	Number	2019	4847.265914	6541.242051	3312.263526	118.47%
Deaths	Angola	Male	Age-standardized	Aortic aneurysm	Rate	1990	4.826144212	7.769655635	2.159002891	
Deaths	Angola	Male	Age-standardized	Aortic aneurysm	Rate	2019	4.293323983	5.550788057	2.971099259	-11.04%
Deaths	Angola	Male	All Ages	Aortic aneurysm	Number	1990	79.32468053	133.3289206	36.92843861	
Deaths	Angola	Male	All Ages	Aortic aneurysm	Number	2019	176.2322866	233.3050789	120.6010424	122.17%
DALYs	Antigua and Barbuda	Male	Age-standardized	Aortic aneurysm	Rate	1990	76.88481252	87.33068353	68.2729559	
DALYs	Antigua and Barbuda	Male	Age-standardized	Aortic aneurysm	Rate	2019	57.9077171	68.54227061	47.92677449	-24.68%
DALYs	Antigua and Barbuda	Male	All Ages	Aortic aneurysm	Number	1990	17.84346895	20.24733525	15.77680002	
DALYs	Antigua and Barbuda	Male	All Ages	Aortic aneurysm	Number	2019	26.60500262	31.84325919	21.8902307	49.10%
Deaths	Antigua and Barbuda	Male	Age-standardized	Aortic aneurysm	Rate	1990	4.10717139	4.649384563	3.624489894	
Deaths	Antigua and Barbuda	Male	Age-standardized	Aortic aneurysm	Rate	2019	3.30374316	3.891140848	2.741330762	-19.56%
Deaths	Antigua and Barbuda	Male	All Ages	Aortic aneurysm	Number	1990	0.924350156	1.057014445	0.808738625	
Deaths	Antigua and Barbuda	Male	All Ages	Aortic aneurysm	Number	2019	1.353525703	1.600953162	1.116230136	46.43%
DALYs	Argentina	Male	Age-standardized	Aortic aneurysm	Rate	1990	153.7976369	171.1782241	136.1659464	
DALYs	Argentina	Male	Age-standardized	Aortic aneurysm	Rate	2019	117.8220158	128.8122802	106.3225087	-23.39%
DALYs	Argentina	Male	All Ages	Aortic aneurysm	Number	1990	22248.37292	24817.04648	19633.74434	
DALYs	Argentina	Male	All Ages	Aortic aneurysm	Number	2019	27827.51196	30436.37943	25087.23796	25.08%
Deaths	Argentina	Male	Age-standardized	Aortic aneurysm	Rate	1990	7.656298331	8.608982522	6.700512086	
Deaths	Argentina	Male	Age-standardized	Aortic aneurysm	Rate	2019	6.008201674	6.634338436	5.374500853	-21.53%
Deaths	Argentina	Male	All Ages	Aortic aneurysm	Number	1990	1028.783398	1157.279208	901.0889989	
Deaths	Argentina	Male	All Ages	Aortic aneurysm	Number	2019	1376.006011	1519.914465	1232.443633	33.75%
DALYs	Armenia	Male	Age-standardized	Aortic aneurysm	Rate	1990	151.3747657	173.7537212	130.6562338	
DALYs	Armenia	Male	Age-standardized	Aortic aneurysm	Rate	2019	263.5230922	313.1129158	215.5560335	74.09%
DALYs	Armenia	Male	All Ages	Aortic aneurysm	Number	1990	1851.223573	2136.392597	1601.89928	
DALYs	Armenia	Male	All Ages	Aortic aneurysm	Number	2019	4657.492263	5554.004348	3797.911118	151.59%

Deaths	Armenia	Male	Age-standardized	Aortic aneurysm	Rate	1990	6.766744029	7.843086648	5.834456035	
Deaths	Armenia	Male	Age-standardized	Aortic aneurysm	Rate	2019	12.62785956	14.8878921	10.48468406	86.62%
Deaths	Armenia	Male	All Ages	Aortic aneurysm	Number	1990	70.81098511	81.81885774	61.22712227	
Deaths	Armenia	Male	All Ages	Aortic aneurysm	Number	2019	212.8990391	252.075556	175.6017009	200.66%
DALYs	Australia	Male	Age-standardized	Aortic aneurysm	Rate	1990	203.1024892	214.3831984	191.4262186	
DALYs	Australia	Male	Age-standardized	Aortic aneurysm	Rate	2019	66.03680866	72.66610256	58.75703489	-67.49%
DALYs	Australia	Male	All Ages	Aortic aneurysm	Number	1990	17829.73979	18812.65275	16834.89659	
DALYs	Australia	Male	All Ages	Aortic aneurysm	Number	2019	12718.92043	13997.10829	11301.88089	-28.66%
Deaths	Australia	Male	Age-standardized	Aortic aneurysm	Rate	1990	11.6515513	12.35366825	10.84103932	
Deaths	Australia	Male	Age-standardized	Aortic aneurysm	Rate	2019	4.011881067	4.451061296	3.515488115	-65.57%
Deaths	Australia	Male	All Ages	Aortic aneurysm	Number	1990	943.0554539	998.6360236	881.8906002	
Deaths	Australia	Male	All Ages	Aortic aneurysm	Number	2019	805.0872285	894.8750607	702.5601267	-14.63%
DALYs	Austria	Male	Age-standardized	Aortic aneurysm	Rate	1990	109.4059149	117.5531821	102.7277014	
DALYs	Austria	Male	Age-standardized	Aortic aneurysm	Rate	2019	61.62412759	68.29446979	55.76252033	-43.67%
DALYs	Austria	Male	All Ages	Aortic aneurysm	Number	1990	4907.504379	5285.860046	4604.83982	
DALYs	Austria	Male	All Ages	Aortic aneurysm	Number	2019	4562.09549	5063.600886	4102.059141	-7.04%
Deaths	Austria	Male	Age-standardized	Aortic aneurysm	Rate	1990	5.35297667	5.825503573	4.984571516	
Deaths	Austria	Male	Age-standardized	Aortic aneurysm	Rate	2019	3.124443039	3.459894447	2.807253685	-41.63%
Deaths	Austria	Male	All Ages	Aortic aneurysm	Number	1990	233.8226081	253.3039693	218.1143865	
Deaths	Austria	Male	All Ages	Aortic aneurysm	Number	2019	247.8042737	275.2191252	222.1935572	5.98%
DALYs	Azerbaijan	Male	Age-standardized	Aortic aneurysm	Rate	1990	21.6887796	25.44963404	18.35471761	
DALYs	Azerbaijan	Male	Age-standardized	Aortic aneurysm	Rate	2019	26.45677701	33.18052749	21.13398259	21.98%
DALYs	Azerbaijan	Male	All Ages	Aortic aneurysm	Number	1990	468.5930594	548.989059	395.9533116	
DALYs	Azerbaijan	Male	All Ages	Aortic aneurysm	Number	2019	1071.861115	1387.489856	833.5654247	128.74%
Deaths	Azerbaijan	Male	Age-standardized	Aortic aneurysm	Rate	1990	1.002983401	1.233459119	0.835598612	
Deaths	Azerbaijan	Male	Age-standardized	Aortic aneurysm	Rate	2019	1.41014652	1.738497501	1.13533744	40.60%
Deaths	Azerbaijan	Male	All Ages	Aortic aneurysm	Number	1990	17.74425296	20.96319917	14.91706753	
Deaths	Azerbaijan	Male	All Ages	Aortic aneurysm	Number	2019	43.46087724	54.99766993	34.73285884	144.93%
DALYs	Bahamas	Male	Age-standardized	Aortic aneurysm	Rate	1990	101.0549722	116.9243964	87.44503806	
DALYs	Bahamas	Male	Age-standardized	Aortic aneurysm	Rate	2019	96.21717333	119.2647445	77.6271541	-4.79%
DALYs	Bahamas	Male	All Ages	Aortic aneurysm	Number	1990	69.52874073	80.20532168	60.39776962	
DALYs	Bahamas	Male	All Ages	Aortic aneurysm	Number	2019	169.9072477	213.1709896	135.9122777	144.37%
Deaths	Bahamas	Male	Age-standardized	Aortic aneurysm	Rate	1990	5.110913184	5.898404927	4.400150028	
Deaths	Bahamas	Male	Age-standardized	Aortic aneurysm	Rate	2019	4.793269397	5.87576677	3.901819325	-6.22%
Deaths	Bahamas	Male	All Ages	Aortic aneurysm	Number	1990	2.952681021	3.421872379	2.548723906	
Deaths	Bahamas	Male	All Ages	Aortic aneurysm	Number	2019	7.398644376	9.175959897	6.002793183	150.57%
DALYs	Bahrain	Male	Age-standardized	Aortic aneurysm	Rate	1990	47.19570726	59.31072622	37.48359522	
DALYs	Bahrain	Male	Age-standardized	Aortic aneurysm	Rate	2019	31.94743732	42.47076579	23.09366909	-32.31%
DALYs	Bahrain	Male	All Ages	Aortic aneurysm	Number	1990	52.16878843	65.27489266	41.71774336	
DALYs	Bahrain	Male	All Ages	Aortic aneurysm	Number	2019	191.9648754	262.9970713	137.8512378	267.97%
Deaths	Bahrain	Male	Age-standardized	Aortic aneurysm	Rate	1990	2.500682832	3.145579055	1.987164408	
Deaths	Bahrain	Male	Age-standardized	Aortic aneurysm	Rate	2019	1.868118936	2.455582419	1.368180864	-25.30%
Deaths	Bahrain	Male	All Ages	Aortic aneurysm	Number	1990	1.80189513	2.267095133	1.43479554	
Deaths	Bahrain	Male	All Ages	Aortic aneurysm	Number	2019	6.801988258	9.266946772	4.864262824	277.49%
DALYs	Bangladesh	Male	Age-standardized	Aortic aneurysm	Rate	1990	37.0937444	66.41769057	18.07429182	
DALYs	Bangladesh	Male	Age-standardized	Aortic aneurysm	Rate	2019	36.80104933	52.83778829	24.45239364	-0.79%
DALYs	Bangladesh	Male	All Ages	Aortic aneurysm	Number	1990	9861.702858	17783.42	4726.427234	
DALYs	Bangladesh	Male	All Ages	Aortic aneurysm	Number	2019	24604.69511	35267.46395	16044.69984	149.50%
Deaths	Bangladesh	Male	Age-standardized	Aortic aneurysm	Rate	1990	1.817260183	3.187746432	0.887816918	
Deaths	Bangladesh	Male	Age-standardized	Aortic aneurysm	Rate	2019	1.974097926	2.840107321	1.293346064	8.63%
Deaths	Bangladesh	Male	All Ages	Aortic aneurysm	Number	1990	412.5773821	733.7973732	200.8561912	
Deaths	Bangladesh	Male	All Ages	Aortic aneurysm	Number	2019	1192.111074	1716.272946	785.2491147	188.94%
DALYs	Barbados	Male	Age-standardized	Aortic aneurysm	Rate	1990	76.8603754	87.00942781	67.63704669	
DALYs	Barbados	Male	Age-standardized	Aortic aneurysm	Rate	2019	72.71040894	89.60331219	58.24501452	-5.40%
DALYs	Barbados	Male	All Ages	Aortic aneurysm	Number	1990	95.11376713	108.0628698	83.29508342	
DALYs	Barbados	Male	All Ages	Aortic aneurysm	Number	2019	159.8755656	197.8712201	127.5888102	68.09%
Deaths	Barbados	Male	Age-standardized	Aortic aneurysm	Rate	1990	4.129442239	4.688173216	3.628081054	
Deaths	Barbados	Male	Age-standardized	Aortic aneurysm	Rate	2019	4.001035536	4.854421371	3.234121809	-3.11%
Deaths	Barbados	Male	All Ages	Aortic aneurysm	Number	1990	5.16663553	5.919397495	4.490744397	
Deaths	Barbados	Male	All Ages	Aortic aneurysm	Number	2019	8.678377474	10.54500615	6.984256599	67.97%
DALYs	Belarus	Male	Age-standardized	Aortic aneurysm	Rate	1990	53.89155827	75.97234172	42.75100416	
DALYs	Belarus	Male	Age-standardized	Aortic aneurysm	Rate	2019	90.09941964	118.8224186	66.83921668	67.19%
DALYs	Belarus	Male	All Ages	Aortic aneurysm	Number	1990	2646.199914	3777.629683	2095.346037	
DALYs	Belarus	Male	All Ages	Aortic aneurysm	Number	2019	5430.267366	7211.562491	3980.030312	105.21%
Deaths	Belarus	Male	Age-standardized	Aortic aneurysm	Rate	1990	2.394966123	3.278580278	1.919987623	
Deaths	Belarus	Male	Age-standardized	Aortic aneurysm	Rate	2019	4.03120489	5.197037643	3.057454	68.32%
Deaths	Belarus	Male	All Ages	Aortic aneurysm	Number	1990	106.1426145	146.5357887	84.78306349	
Deaths	Belarus	Male	All Ages	Aortic aneurysm	Number	2019	228.4780744	299.0803542	170.5642783	115.26%
DALYs	Belgium	Male	Age-standardized	Aortic aneurysm	Rate	1990	150.031207	160.7585364	139.8185022	
DALYs	Belgium	Male	Age-standardized	Aortic aneurysm	Rate	2019	76.83073324	85.01357423	69.40986394	-48.79%
DALYs	Belgium	Male	All Ages	Aortic aneurysm	Number	1990	9554.945319	10296.58812	8864.94818	
DALYs	Belgium	Male	All Ages	Aortic aneurysm	Number	2019	7760.860986	8602.452508	6973.226256	-18.78%
Deaths	Belgium	Male	Age-standardized	Aortic aneurysm	Rate	1990	8.424801366	9.030104848	7.830129945	
Deaths	Belgium	Male	Age-standardized	Aortic aneurysm	Rate	2019	4.684863085	5.174818213	4.182806793	-44.39%
Deaths	Belgium	Male	All Ages	Aortic aneurysm	Number	1990	510.622874	548.791432	474.306362	
Deaths	Belgium	Male	All Ages	Aortic aneurysm	Number	2019	501.1580417	554.5132139	446.0294721	-1.85%
DALYs	Belize	Male	Age-standardized	Aortic aneurysm	Rate	1990	27.32589812	40.16001014	20.81286783	
DALYs	Belize	Male	Age-standardized	Aortic aneurysm	Rate	2019	35.65438997	42.49037961	29.43914905	30.48%
DALYs	Belize	Male	All Ages	Aortic aneurysm	Number	1990	12.89577713	19.15982788	9.750622137	
DALYs	Belize	Male	All Ages	Aortic aneurysm	Number	2019	49.97521068	59.60716351	41.15191003	287.53%
Deaths	Belize	Male	Age-standardized	Aortic aneurysm	Rate	1990	1.439905398	2.075344001	1.092331614	
Deaths	Belize	Male	Age-standardized	Aortic aneurysm	Rate	2019	1.827946433	2.160765211	1.517906449	26.95%
Deaths	Belize	Male	All Ages	Aortic aneurysm	Number	1990	0.626920292	0.906811707	0.477280569	
Deaths	Belize	Male	All Ages	Aortic aneurysm	Number	2019	2.278493334	2.703653988	1.89266809	263.44%
DALYs	Benin	Male	Age-standardized	Aortic aneurysm	Rate	1990	48.80103775	71.67054818	28.60807323	
DALYs	Benin	Male	Age-standardized	Aortic aneurysm	Rate	2019	48.0045936	65.55094935	32.72446198	-1.63%
DALYs	Benin	Male	All Ages	Aortic aneurysm	Number	1990	494.9396114	731.8982085	292.9605322	
DALYs	Benin	Male	All Ages	Aortic aneurysm	Number	2019	1162.243487	1605.748621	774.4502368	134.83%
Deaths	Benin	Male	Age-standardized	Aortic aneurysm	Rate	1990	2.276453799	3.351580261	1.32526069	
Deaths	Benin	Male	Age-standardized	Aortic aneurysm	Rate	2019	2.374649754	3.170224529	1.642516393	4.31%
Deaths	Benin	Male	All Ages	Aortic aneurysm	Number	1990	20.80719473	30.69364843	12.13125577	
Deaths	Benin	Male	All Ages	Aortic aneurysm	Number	2019	46.07495974	62.68635688	31.55536365	121.44%
DALYs	Bermuda	Male	Age-standardized	Aortic aneurysm	Rate	1990	282.5511514	334.4671904	209.7364229	
DALYs	Bermuda	Male	Age-standardized	Aortic aneurysm	Rate	2019	166.5473968	200.3649328	137.8421264	-41.06%
DALYs	Bermuda	Male	All Ages	Aortic aneurysm	Number	1990	74.70318599	88.51159212	55.30397603	
DALYs	Bermuda	Male	All Ages	Aortic aneurysm	Number	2019	94.95602938	114.800036	78.36993741	27.11%
Deaths	Bermuda	Male	Age-standardized	Aortic aneurysm	Rate	1990	15.56345308	18.37857091	11.62663024	
Deaths	Bermuda	Male	Age-standardized	Aortic aneurysm	Rate	2019	9.884236489	11.78566542	8.19135779	-36.49%
Deaths	Bermuda	Male	All Ages	Aortic aneurysm	Number	1990	3.680260255	4.363845605	2.728597492	
Deaths	Bermuda	Male	All Ages	Aortic aneurysm	Number	2019	5.608106421	6.694126846	4.634816542	52.38%
DALYs	Bhutan	Male	Age-standardized	Aortic aneurysm	Rate	1990	39.86777878	73.30482622	16.73360862	

DALYs	Bhutan	Male	Age-standardized	Aortic aneurysm	Rate	2019	49.49886112	70.14959273	32.57747431	24.16%
DALYs	Bhutan	Male	All Ages	Aortic aneurysm	Number	1990	48.75516751	92.492831	20.29795069	
DALYs	Bhutan	Male	All Ages	Aortic aneurysm	Number	2019	141.3726517	202.7950088	92.25073981	189.96%
Deaths	Bhutan	Male	Age-standardized	Aortic aneurysm	Rate	1990	2.006766684	3.568791439	0.839686347	
Deaths	Bhutan	Male	Age-standardized	Aortic aneurysm	Rate	2019	2.685720362	3.73150809	1.78495008	33.83%
Deaths	Bhutan	Male	All Ages	Aortic aneurysm	Number	1990	1.8383731	3.422976573	0.774895546	
Deaths	Bhutan	Male	All Ages	Aortic aneurysm	Number	2019	6.812061409	9.535303702	4.517604407	270.55%
DALYs	Bolivia (Plurinational State of)	Male	Age-standardized	Aortic aneurysm	Rate	1990	53.85215592	82.86609216	29.31604892	
DALYs	Bolivia (Plurinational State of)	Male	Age-standardized	Aortic aneurysm	Rate	2019	53.25420939	75.82146813	31.93857816	-1.11%
DALYs	Bolivia (Plurinational State of)	Male	All Ages	Aortic aneurysm	Number	1990	837.9163068	1301.323003	455.5772882	
DALYs	Bolivia (Plurinational State of)	Male	All Ages	Aortic aneurysm	Number	2019	2225.59772	3194.480897	1325.726312	165.61%
Deaths	Bolivia (Plurinational State of)	Male	Age-standardized	Aortic aneurysm	Rate	1990	2.663292599	4.017919467	1.495503728	
Deaths	Bolivia (Plurinational State of)	Male	Age-standardized	Aortic aneurysm	Rate	2019	2.864864224	3.964000581	1.736860571	7.57%
Deaths	Bolivia (Plurinational State of)	Male	All Ages	Aortic aneurysm	Number	1990	34.29603046	52.28520774	18.62576395	
Deaths	Bolivia (Plurinational State of)	Male	All Ages	Aortic aneurysm	Number	2019	104.3734787	146.9042531	62.4969989	204.33%
DALYs	Bosnia and Herzegovina	Male	Age-standardized	Aortic aneurysm	Rate	1990	87.39713537	112.3800106	67.19642994	
DALYs	Bosnia and Herzegovina	Male	Age-standardized	Aortic aneurysm	Rate	2019	108.011967	135.2760171	84.27591756	23.59%
DALYs	Bosnia and Herzegovina	Male	All Ages	Aortic aneurysm	Number	1990	1644.057957	2138.42461	1244.190875	
DALYs	Bosnia and Herzegovina	Male	All Ages	Aortic aneurysm	Number	2019	2744.976933	3458.673924	2132.957023	66.96%
Deaths	Bosnia and Herzegovina	Male	Age-standardized	Aortic aneurysm	Rate	1990	4.02625206	5.118498129	3.082010565	
Deaths	Bosnia and Herzegovina	Male	Age-standardized	Aortic aneurysm	Rate	2019	5.545554427	6.853130773	4.417547511	37.73%
Deaths	Bosnia and Herzegovina	Male	All Ages	Aortic aneurysm	Number	1990	62.69835337	80.28382693	48.15676322	
Deaths	Bosnia and Herzegovina	Male	All Ages	Aortic aneurysm	Number	2019	134.2429449	167.0825309	105.8001495	114.11%
DALYs	Botswana	Male	Age-standardized	Aortic aneurysm	Rate	1990	85.80364886	115.708952	59.06719589	
DALYs	Botswana	Male	Age-standardized	Aortic aneurysm	Rate	2019	82.03082223	115.8294357	54.54553249	-4.40%
DALYs	Botswana	Male	All Ages	Aortic aneurysm	Number	1990	220.8823628	302.3030637	149.2497739	
DALYs	Botswana	Male	All Ages	Aortic aneurysm	Number	2019	486.2508695	720.9673648	313.7620062	120.14%
Deaths	Botswana	Male	Age-standardized	Aortic aneurysm	Rate	1990	4.372174814	5.82255853	3.041834395	
Deaths	Botswana	Male	Age-standardized	Aortic aneurysm	Rate	2019	4.385571526	6.076231646	2.956666231	0.31%
Deaths	Botswana	Male	All Ages	Aortic aneurysm	Number	1990	8.672107233	11.68296688	5.958493597	
Deaths	Botswana	Male	All Ages	Aortic aneurysm	Number	2019	18.74180812	26.59255825	12.43126934	116.12%
DALYs	Brazil	Male	Age-standardized	Aortic aneurysm	Rate	1990	113.8708465	119.4526245	108.9248579	
DALYs	Brazil	Male	Age-standardized	Aortic aneurysm	Rate	2019	129.4647184	139.2050926	120.1619532	13.69%
DALYs	Brazil	Male	All Ages	Aortic aneurysm	Number	1990	51966.02828	54371.18727	49852.8228	
DALYs	Brazil	Male	All Ages	Aortic aneurysm	Number	2019	141292.686	152057.275	131118.9806	171.89%
Deaths	Brazil	Male	Age-standardized	Aortic aneurysm	Rate	1990	4.922259471	5.162623777	4.666183213	
Deaths	Brazil	Male	Age-standardized	Aortic aneurysm	Rate	2019	5.966536372	6.442643705	5.479774651	21.22%
Deaths	Brazil	Male	All Ages	Aortic aneurysm	Number	1990	1936.479009	2029.662266	1849.424365	
Deaths	Brazil	Male	All Ages	Aortic aneurysm	Number	2019	6064.377274	6533.801002	5592.752005	213.17%
DALYs	Brunei Darussalam	Male	Age-standardized	Aortic aneurysm	Rate	1990	174.1139681	233.2472958	123.4717103	
DALYs	Brunei Darussalam	Male	Age-standardized	Aortic aneurysm	Rate	2019	155.2805702	181.0572291	132.0092055	-10.82%
DALYs	Brunei Darussalam	Male	All Ages	Aortic aneurysm	Number	1990	87.34249553	118.5441864	60.48946532	
DALYs	Brunei Darussalam	Male	All Ages	Aortic aneurysm	Number	2019	196.3501417	233.7171629	163.2189032	124.80%
Deaths	Brunei Darussalam	Male	Age-standardized	Aortic aneurysm	Rate	1990	9.663533985	12.87788179	7.024869895	
Deaths	Brunei Darussalam	Male	Age-standardized	Aortic aneurysm	Rate	2019	9.519962576	11.12024505	8.08982133	-1.49%
Deaths	Brunei Darussalam	Male	All Ages	Aortic aneurysm	Number	1990	3.150181441	4.246305307	2.199582966	
Deaths	Brunei Darussalam	Male	All Ages	Aortic aneurysm	Number	2019	7.592199865	8.934642136	6.351266558	141.01%
DALYs	Bulgaria	Male	Age-standardized	Aortic aneurysm	Rate	1990	74.29745398	82.31902353	67.18123122	
DALYs	Bulgaria	Male	Age-standardized	Aortic aneurysm	Rate	2019	119.3402419	151.4994946	92.07287585	60.62%
DALYs	Bulgaria	Male	All Ages	Aortic aneurysm	Number	1990	4057.406445	4517.701251	3647.530946	
DALYs	Bulgaria	Male	All Ages	Aortic aneurysm	Number	2019	6446.98769	8172.820944	4940.589991	58.89%
Deaths	Bulgaria	Male	Age-standardized	Aortic aneurysm	Rate	1990	2.67762104	2.955669289	2.414714934	
Deaths	Bulgaria	Male	Age-standardized	Aortic aneurysm	Rate	2019	4.460562583	5.55963637	3.502036521	66.59%
Deaths	Bulgaria	Male	All Ages	Aortic aneurysm	Number	1990	141.9641233	157.6626197	127.6650014	
Deaths	Bulgaria	Male	All Ages	Aortic aneurysm	Number	2019	259.5788994	324.959526	201.8417719	82.85%
DALYs	Burkina Faso	Male	Age-standardized	Aortic aneurysm	Rate	1990	43.62127115	73.46973483	19.09231118	
DALYs	Burkina Faso	Male	Age-standardized	Aortic aneurysm	Rate	2019	54.80755039	84.71994479	29.72317818	25.64%
DALYs	Burkina Faso	Male	All Ages	Aortic aneurysm	Number	1990	920.9916296	1562.880225	399.9610406	
DALYs	Burkina Faso	Male	All Ages	Aortic aneurysm	Number	2019	2296.847417	3619.212329	1213.454146	149.39%
Deaths	Burkina Faso	Male	Age-standardized	Aortic aneurysm	Rate	1990	2.113629642	3.548989217	0.927018354	
Deaths	Burkina Faso	Male	Age-standardized	Aortic aneurysm	Rate	2019	2.710450005	4.149967893	1.486368454	28.24%
Deaths	Burkina Faso	Male	All Ages	Aortic aneurysm	Number	1990	38.60081973	64.56153536	16.8798704	
Deaths	Burkina Faso	Male	All Ages	Aortic aneurysm	Number	2019	94.84039207	147.1491916	51.59020744	145.70%
DALYs	Burundi	Male	Age-standardized	Aortic aneurysm	Rate	1990	115.7560236	220.714741	50.97428041	
DALYs	Burundi	Male	Age-standardized	Aortic aneurysm	Rate	2019	54.01221494	85.86234645	25.4296868	-53.34%
DALYs	Burundi	Male	All Ages	Aortic aneurysm	Number	1990	1358.913825	2595.452427	588.3961568	
DALYs	Burundi	Male	All Ages	Aortic aneurysm	Number	2019	1416.652496	2301.742236	655.5281904	4.25%
Deaths	Burundi	Male	Age-standardized	Aortic aneurysm	Rate	1990	5.103207776	9.583501138	2.287562883	
Deaths	Burundi	Male	Age-standardized	Aortic aneurysm	Rate	2019	2.542802846	3.967931294	1.209499813	-50.17%
Deaths	Burundi	Male	All Ages	Aortic aneurysm	Number	1990	51.41301274	97.14033382	22.73339212	
Deaths	Burundi	Male	All Ages	Aortic aneurysm	Number	2019	50.96827239	80.93564052	24.0554867	-0.87%
DALYs	Cabo Verde	Male	Age-standardized	Aortic aneurysm	Rate	1990	35.95807767	47.80848326	25.0558587	
DALYs	Cabo Verde	Male	Age-standardized	Aortic aneurysm	Rate	2019	76.52646198	91.89040472	64.13696008	112.82%
DALYs	Cabo Verde	Male	All Ages	Aortic aneurysm	Number	1990	36.61850995	48.55591657	25.48494064	
DALYs	Cabo Verde	Male	All Ages	Aortic aneurysm	Number	2019	137.6893379	167.4045791	113.6308573	276.01%
Deaths	Cabo Verde	Male	Age-standardized	Aortic aneurysm	Rate	1990	1.718392047	2.287605976	1.189749296	
Deaths	Cabo Verde	Male	Age-standardized	Aortic aneurysm	Rate	2019	4.052469488	4.830447689	3.425267495	135.83%
Deaths	Cabo Verde	Male	All Ages	Aortic aneurysm	Number	1990	1.780499405	2.384937858	1.226263658	
Deaths	Cabo Verde	Male	All Ages	Aortic aneurysm	Number	2019	6.571616217	7.831130206	5.526986803	269.09%
DALYs	Cambodia	Male	Age-standardized	Aortic aneurysm	Rate	1990	29.02232666	46.04835805	16.95091457	
DALYs	Cambodia	Male	Age-standardized	Aortic aneurysm	Rate	2019	34.57271217	49.25394893	24.73509853	19.12%
DALYs	Cambodia	Male	All Ages	Aortic aneurysm	Number	1990	570.3954269	927.7159422	330.8961157	
DALYs	Cambodia	Male	All Ages	Aortic aneurysm	Number	2019	1664.840004	2394.3709	1173.512854	191.87%
Deaths	Cambodia	Male	Age-standardized	Aortic aneurysm	Rate	1990	1.462526287	2.274196407	0.860200998	
Deaths	Cambodia	Male	Age-standardized	Aortic aneurysm	Rate	2019	1.868270335	2.653241163	1.344225652	27.74%
Deaths	Cambodia	Male	All Ages	Aortic aneurysm	Number	1990	22.79754647	36.06663746	13.33803405	
Deaths	Cambodia	Male	All Ages	Aortic aneurysm	Number	2019	72.65037202	103.4490213	52.18302534	218.68%
DALYs	Cameroon	Male	Age-standardized	Aortic aneurysm	Rate	1990	44.67481591	66.59357709	27.55106614	
DALYs	Cameroon	Male	Age-standardized	Aortic aneurysm	Rate	2019	53.52736947	80.65629193	34.97309048	19.82%
DALYs	Cameroon	Male	All Ages	Aortic aneurysm	Number	1990	994.2970953	1486.539324	606.9753049	
DALYs	Cameroon	Male	All Ages	Aortic aneurysm	Number	2019	3288.227815	5010.290394	2082.124025	230.71%
Deaths	Cameroon	Male	Age-standardized	Aortic aneurysm	Rate	1990	2.209008126	3.266435255	1.359534539	
Deaths	Cameroon	Male	Age-standardized	Aortic aneurysm	Rate	2019	2.66248132	3.957575206	1.782538392	20.53%
Deaths	Cameroon	Male	All Ages	Aortic aneurysm	Number	1990	40.32241255	60.04997348	24.90403756	
Deaths	Cameroon	Male	All Ages	Aortic aneurysm	Number	2019	126.779271	191.1430763	83.20464969	214.41%
DALYs	Canada	Male	Age-standardized	Aortic aneurysm	Rate	1990	176.8105597	186.6082506	167.1027135	
DALYs	Canada	Male	Age-standardized	Aortic aneurysm	Rate	2019	66.43028031	73.02410226	59.78282256	-62.43%
DALYs	Canada	Male	All Ages	Aortic aneurysm	Number	1990	25299.70216	26778.59976	23916.82712	
DALYs	Canada	Male	All Ages	Aortic aneurysm	Number	2019	20412.41424	22488.15844	18297.08692	-19.32%
Deaths	Canada	Male	Age-standardized	Aortic aneurysm	Rate	1990	9.995808665	10.62060939	9.357058251	
Deaths	Canada	Male	Age-standardized	Aortic aneurysm	Rate	2019	3.868968247	4.268046313	3.4292802	-61.29%

Deaths	Canada	Male	All Ages	Aortic aneurysm	Number	1990	1339.227934	1422.367063	1256.413426	
Deaths	Canada	Male	All Ages	Aortic aneurysm	Number	2019	1231.13846	1362.587605	1086.863195	-8.07%
DALYs	Central African Republic	Male	Age-standardized	Aortic aneurysm	Rate	1990	126.8479643	220.6908984	63.58255575	
DALYs	Central African Republic	Male	Age-standardized	Aortic aneurysm	Rate	2019	92.2988444	155.7502201	50.85234327	-27.24%
DALYs	Central African Republic	Male	All Ages	Aortic aneurysm	Number	1990	743.9468598	1326.855042	364.0343547	
DALYs	Central African Republic	Male	All Ages	Aortic aneurysm	Number	2019	1019.741307	1828.492081	541.9482704	37.07%
Deaths	Central African Republic	Male	Age-standardized	Aortic aneurysm	Rate	1990	5.538460918	9.393882847	2.725370574	
Deaths	Central African Republic	Male	Age-standardized	Aortic aneurysm	Rate	2019	4.03478938	6.49893238	2.238343504	-27.15%
Deaths	Central African Republic	Male	All Ages	Aortic aneurysm	Number	1990	26.2701404	45.85989327	13.16178476	
Deaths	Central African Republic	Male	All Ages	Aortic aneurysm	Number	2019	33.77221154	57.9417986	18.4499131	28.56%
DALYs	Chad	Male	Age-standardized	Aortic aneurysm	Rate	1990	37.39528322	63.89002905	18.26695737	
DALYs	Chad	Male	Age-standardized	Aortic aneurysm	Rate	2019	40.65717645	63.54300185	24.44432412	8.72%
DALYs	Chad	Male	All Ages	Aortic aneurysm	Number	1990	526.124903	896.1670315	256.4581393	
DALYs	Chad	Male	All Ages	Aortic aneurysm	Number	2019	1277.952308	2024.867404	764.4984805	142.90%
Deaths	Chad	Male	Age-standardized	Aortic aneurysm	Rate	1990	1.816665783	3.060581722	0.886808578	
Deaths	Chad	Male	Age-standardized	Aortic aneurysm	Rate	2019	2.00396261	3.095704591	1.222308633	10.31%
Deaths	Chad	Male	All Ages	Aortic aneurysm	Number	1990	23.07992492	39.3239858	11.19520147	
Deaths	Chad	Male	All Ages	Aortic aneurysm	Number	2019	52.40306231	81.65466732	31.61598349	127.05%
DALYs	Chile	Male	Age-standardized	Aortic aneurysm	Rate	1990	69.24295062	74.99555705	62.4499713	
DALYs	Chile	Male	Age-standardized	Aortic aneurysm	Rate	2019	73.1322337	80.07469641	66.53469344	5.62%
DALYs	Chile	Male	All Ages	Aortic aneurysm	Number	1990	3308.86133	3579.696238	2986.793031	
DALYs	Chile	Male	All Ages	Aortic aneurysm	Number	2019	7903.356249	8671.991814	7170.927903	138.85%
Deaths	Chile	Male	Age-standardized	Aortic aneurysm	Rate	1990	3.10971722	3.376834538	2.820641791	
Deaths	Chile	Male	Age-standardized	Aortic aneurysm	Rate	2019	3.329669011	3.661127293	2.993413696	7.07%
Deaths	Chile	Male	All Ages	Aortic aneurysm	Number	1990	133.3973988	144.89114	120.8086352	
Deaths	Chile	Male	All Ages	Aortic aneurysm	Number	2019	351.9986276	387.1973372	317.0848716	163.87%
DALYs	China	Male	Age-standardized	Aortic aneurysm	Rate	1990	30.62096484	42.00804641	22.11487833	
DALYs	China	Male	Age-standardized	Aortic aneurysm	Rate	2019	30.13958668	37.58921511	24.02920586	-1.57%
DALYs	China	Male	All Ages	Aortic aneurysm	Number	1990	132331.0837	184451.8285	95032.3466	
DALYs	China	Male	All Ages	Aortic aneurysm	Number	2019	279762.9	352084.8072	220488.9022	111.41%
Deaths	China	Male	Age-standardized	Aortic aneurysm	Rate	1990	1.478126351	1.977656088	1.070570468	
Deaths	China	Male	Age-standardized	Aortic aneurysm	Rate	2019	1.480737009	1.803433675	1.202105069	0.18%
Deaths	China	Male	All Ages	Aortic aneurysm	Number	1990	4897.298031	6746.23203	3535.15633	
Deaths	China	Male	All Ages	Aortic aneurysm	Number	2019	12089.0425	14990.94835	9620.270198	146.85%
DALYs	Colombia	Male	Age-standardized	Aortic aneurysm	Rate	1990	105.9844047	113.0138057	99.55881935	
DALYs	Colombia	Male	Age-standardized	Aortic aneurysm	Rate	2019	87.10297408	114.0762874	64.67655264	-17.82%
DALYs	Colombia	Male	All Ages	Aortic aneurysm	Number	1990	9681.465172	10283.96437	9107.732625	
DALYs	Colombia	Male	All Ages	Aortic aneurysm	Number	2019	20831.32675	27267.25254	15495.36601	115.17%
Deaths	Colombia	Male	Age-standardized	Aortic aneurysm	Rate	1990	4.849665427	5.21676036	4.506024562	
Deaths	Colombia	Male	Age-standardized	Aortic aneurysm	Rate	2019	4.267161008	5.501651957	3.218126505	-12.01%
Deaths	Colombia	Male	All Ages	Aortic aneurysm	Number	1990	385.120351	411.1002709	360.7025855	
Deaths	Colombia	Male	All Ages	Aortic aneurysm	Number	2019	1013.493074	1308.340943	765.1215035	163.16%
DALYs	Comoros	Male	Age-standardized	Aortic aneurysm	Rate	1990	85.13798783	137.9169033	38.24399445	
DALYs	Comoros	Male	Age-standardized	Aortic aneurysm	Rate	2019	55.7336505	83.74520107	36.38061734	-34.54%
DALYs	Comoros	Male	All Ages	Aortic aneurysm	Number	1990	95.53429399	157.2423038	39.56727829	
DALYs	Comoros	Male	All Ages	Aortic aneurysm	Number	2019	128.2009091	193.6864382	82.73814972	34.19%
Deaths	Comoros	Male	Age-standardized	Aortic aneurysm	Rate	1990	4.08506324	6.451059715	2.018142768	
Deaths	Comoros	Male	Age-standardized	Aortic aneurysm	Rate	2019	2.773818498	4.230051616	1.833455237	-32.10%
Deaths	Comoros	Male	All Ages	Aortic aneurysm	Number	1990	4.008020344	6.377201178	1.900519952	
Deaths	Comoros	Male	All Ages	Aortic aneurysm	Number	2019	5.504833949	8.374107052	3.626798142	37.35%
DALYs	Congo	Male	Age-standardized	Aortic aneurysm	Rate	1990	145.1436704	200.2093779	96.24617383	
DALYs	Congo	Male	Age-standardized	Aortic aneurysm	Rate	2019	93.64793745	124.3102948	70.35330708	-35.48%
DALYs	Congo	Male	All Ages	Aortic aneurysm	Number	1990	738.4137358	1057.858497	493.0867943	
DALYs	Congo	Male	All Ages	Aortic aneurysm	Number	2019	1231.851981	1676.513443	898.1390539	66.82%
Deaths	Congo	Male	Age-standardized	Aortic aneurysm	Rate	1990	6.552333971	8.736162636	4.262018868	
Deaths	Congo	Male	Age-standardized	Aortic aneurysm	Rate	2019	4.553581009	6.05684174	3.492960552	-30.50%
Deaths	Congo	Male	All Ages	Aortic aneurysm	Number	1990	27.29226147	37.65895833	18.06700887	
Deaths	Congo	Male	All Ages	Aortic aneurysm	Number	2019	46.64775012	61.86426425	35.0440396	70.92%
DALYs	Cook Islands	Male	Age-standardized	Aortic aneurysm	Rate	1990	124.7001627	152.4933404	92.14861144	
DALYs	Cook Islands	Male	Age-standardized	Aortic aneurysm	Rate	2019	93.20345153	121.8404623	69.85445188	-25.26%
DALYs	Cook Islands	Male	All Ages	Aortic aneurysm	Number	1990	8.619234804	10.66244264	6.290703687	
DALYs	Cook Islands	Male	All Ages	Aortic aneurysm	Number	2019	10.64836486	13.72032053	8.059077611	23.54%
Deaths	Cook Islands	Male	Age-standardized	Aortic aneurysm	Rate	1990	5.76480706	6.936339662	4.346071442	
Deaths	Cook Islands	Male	Age-standardized	Aortic aneurysm	Rate	2019	4.294048085	5.447206446	3.305987284	-25.51%
Deaths	Cook Islands	Male	All Ages	Aortic aneurysm	Number	1990	0.340969847	0.413038205	0.254352964	
Deaths	Cook Islands	Male	All Ages	Aortic aneurysm	Number	2019	0.489100548	0.612828882	0.376703247	43.44%
DALYs	Costa Rica	Male	Age-standardized	Aortic aneurysm	Rate	1990	61.75430126	69.25842741	55.09088562	
DALYs	Costa Rica	Male	Age-standardized	Aortic aneurysm	Rate	2019	88.73397502	115.7653375	66.10644662	43.69%
DALYs	Costa Rica	Male	All Ages	Aortic aneurysm	Number	1990	557.6749634	622.0422645	502.0345795	
DALYs	Costa Rica	Male	All Ages	Aortic aneurysm	Number	2019	2096.834676	2734.947522	1558.773561	276.00%
Deaths	Costa Rica	Male	Age-standardized	Aortic aneurysm	Rate	1990	3.046445695	3.461696183	2.662763853	
Deaths	Costa Rica	Male	Age-standardized	Aortic aneurysm	Rate	2019	4.330930081	5.685958944	3.264163903	42.16%
Deaths	Costa Rica	Male	All Ages	Aortic aneurysm	Number	1990	24.71341285	27.95029539	21.73298869	
Deaths	Costa Rica	Male	All Ages	Aortic aneurysm	Number	2019	98.9368749	128.8646847	74.63815993	300.34%
DALYs	Croatia	Male	Age-standardized	Aortic aneurysm	Rate	1990	78.83243463	86.7746025	71.3470565	
DALYs	Croatia	Male	Age-standardized	Aortic aneurysm	Rate	2019	130.6425282	166.4365612	100.8704132	65.72%
DALYs	Croatia	Male	All Ages	Aortic aneurysm	Number	1990	2129.681693	2351.418102	1914.426126	
DALYs	Croatia	Male	All Ages	Aortic aneurysm	Number	2019	4646.728558	5930.026365	3583.595945	118.19%
Deaths	Croatia	Male	Age-standardized	Aortic aneurysm	Rate	1990	3.403211173	3.745598825	3.086895198	
Deaths	Croatia	Male	Age-standardized	Aortic aneurysm	Rate	2019	6.322788177	7.868998029	4.978069298	85.79%
Deaths	Croatia	Male	All Ages	Aortic aneurysm	Number	1990	83.41603282	91.98484549	75.45396022	
Deaths	Croatia	Male	All Ages	Aortic aneurysm	Number	2019	231.1353601	288.9435168	180.850071	177.09%
DALYs	Cuba	Male	Age-standardized	Aortic aneurysm	Rate	1990	119.207132	137.5966044	98.31846567	
DALYs	Cuba	Male	Age-standardized	Aortic aneurysm	Rate	2019	107.2726971	133.706716	84.59443171	-10.01%
DALYs	Cuba	Male	All Ages	Aortic aneurysm	Number	1990	6094.874007	7047.17522	5025.717908	
DALYs	Cuba	Male	All Ages	Aortic aneurysm	Number	2019	9492.547272	11837.28061	7470.178329	55.75%
Deaths	Cuba	Male	Age-standardized	Aortic aneurysm	Rate	1990	6.588703801	7.694079644	5.364371742	
Deaths	Cuba	Male	Age-standardized	Aortic aneurysm	Rate	2019	6.038333392	7.498842046	4.758729963	-8.35%
Deaths	Cuba	Male	All Ages	Aortic aneurysm	Number	1990	329.5941372	386.3513365	267.4664165	
Deaths	Cuba	Male	All Ages	Aortic aneurysm	Number	2019	544.0827919	674.5222531	428.9361644	65.08%
DALYs	Cyprus	Male	Age-standardized	Aortic aneurysm	Rate	1990	206.0525817	269.4511442	155.3223615	
DALYs	Cyprus	Male	Age-standardized	Aortic aneurysm	Rate	2019	127.0525518	148.2341424	107.3392802	-38.34%
DALYs	Cyprus	Male	All Ages	Aortic aneurysm	Number	1990	794.2663982	1029.732941	597.1084111	
DALYs	Cyprus	Male	All Ages	Aortic aneurysm	Number	2019	1158.313203	1351.885977	979.2194076	45.83%
Deaths	Cyprus	Male	Age-standardized	Aortic aneurysm	Rate	1990	10.32283435	13.72600705	7.696297489	
Deaths	Cyprus	Male	Age-standardized	Aortic aneurysm	Rate	2019	6.526392284	7.717403149	5.482070302	-36.78%
Deaths	Cyprus	Male	All Ages	Aortic aneurysm	Number	1990	36.97125548	48.89292926	27.71126562	
Deaths	Cyprus	Male	All Ages	Aortic aneurysm	Number	2019	57.83336391	68.66159087	48.25863685	56.43%
DALYs	Czechia	Male	Age-standardized	Aortic aneurysm	Rate	1990	88.01299721	94.66671731	80.50914907	
DALYs	Czechia	Male	Age-standardized	Aortic aneurysm	Rate	2019	86.65400036	106.1264803	70.44343079	-1.54%
DALYs	Czechia	Male	All Ages	Aortic aneurysm	Number	1990	5020.263792	5412.222936	4582.866153	

DALYs	Czechia	Male	All Ages	Aortic aneurysm	Number	2019	7596.61389	9341.928639	6158.412068	51.32%
Deaths	Czechia	Male	Age-standardized	Aortic aneurysm	Rate	1990	3.856115665	4.158067805	3.546359999	
Deaths	Czechia	Male	Age-standardized	Aortic aneurysm	Rate	2019	4.173363102	5.047077216	3.434672085	8.23%
Deaths	Czechia	Male	All Ages	Aortic aneurysm	Number	1990	209.9789024	226.8039261	192.4642916	
Deaths	Czechia	Male	All Ages	Aortic aneurysm	Number	2019	374.4886767	454.0076922	307.3096579	78.35%
DALYs	C么te d'Ivoire	Male	Age-standardized	Aortic aneurysm	Rate	1990	52.99328212	80.37947169	30.77265747	
DALYs	C么te d'Ivoire	Male	Age-standardized	Aortic aneurysm	Rate	2019	49.3719883	67.35901595	34.01478895	-6.83%
DALYs	C么te d'Ivoire	Male	All Ages	Aortic aneurysm	Number	1990	1219.524991	1880.00773	698.8278819	
DALYs	C么te d'Ivoire	Male	All Ages	Aortic aneurysm	Number	2019	2862.831968	3997.263144	1902.30072	134.75%
Deaths	C么te d'Ivoire	Male	Age-standardized	Aortic aneurysm	Rate	1990	2.597012964	3.871082693	1.511598391	
Deaths	C么te d'Ivoire	Male	Age-standardized	Aortic aneurysm	Rate	2019	2.508689093	3.359333927	1.770306497	-3.40%
Deaths	C么te d'Ivoire	Male	All Ages	Aortic aneurysm	Number	1990	44.44711513	67.77235063	25.80193083	
Deaths	C么te d'Ivoire	Male	All Ages	Aortic aneurysm	Number	2019	109.4490229	149.4334579	75.34892454	146.25%
DALYs	Democratic People's Republic of Korea	Male	Age-standardized	Aortic aneurysm	Rate	1990	44.69238284	67.05491719	28.52184848	
DALYs	Democratic People's Republic of Korea	Male	Age-standardized	Aortic aneurysm	Rate	2019	46.44935541	60.91480276	36.55832885	3.93%
DALYs	Democratic People's Republic of Korea	Male	All Ages	Aortic aneurysm	Number	1990	3151.311348	4930.855982	1916.747991	
DALYs	Democratic People's Republic of Korea	Male	All Ages	Aortic aneurysm	Number	2019	6573.911548	8957.108748	4975.67674	108.61%
Deaths	Democratic People's Republic of Korea	Male	Age-standardized	Aortic aneurysm	Rate	1990	1.926236656	2.734168267	1.245409718	
Deaths	Democratic People's Republic of Korea	Male	Age-standardized	Aortic aneurysm	Rate	2019	1.955542468	2.388710233	1.61280196	1.52%
Deaths	Democratic People's Republic of Korea	Male	All Ages	Aortic aneurysm	Number	1990	103.5221412	156.8858296	65.69930146	
Deaths	Democratic People's Republic of Korea	Male	All Ages	Aortic aneurysm	Number	2019	236.5391402	301.6337881	189.3980959	128.49%
DALYs	Democratic Republic of the Congo	Male	Age-standardized	Aortic aneurysm	Rate	1990	95.60526167	145.4386761	55.37804973	
DALYs	Democratic Republic of the Congo	Male	Age-standardized	Aortic aneurysm	Rate	2019	71.84042686	104.5905407	47.46323925	-24.86%
DALYs	Democratic Republic of the Congo	Male	All Ages	Aortic aneurysm	Number	1990	7350.708596	11484.33729	4281.011464	
DALYs	Democratic Republic of the Congo	Male	All Ages	Aortic aneurysm	Number	2019	12359.23136	18096.88103	8144.135585	68.14%
Deaths	Democratic Republic of the Congo	Male	Age-standardized	Aortic aneurysm	Rate	1990	4.538262605	6.775151537	2.588313391	
Deaths	Democratic Republic of the Congo	Male	Age-standardized	Aortic aneurysm	Rate	2019	3.422832121	4.931929575	2.243977276	-24.58%
Deaths	Democratic Republic of the Congo	Male	All Ages	Aortic aneurysm	Number	1990	284.5534008	431.9124101	164.9763177	
Deaths	Democratic Republic of the Congo	Male	All Ages	Aortic aneurysm	Number	2019	450.9556418	658.6714535	298.2961096	58.48%
DALYs	Denmark	Male	Age-standardized	Aortic aneurysm	Rate	1990	209.0430607	230.2622441	187.9671931	
DALYs	Denmark	Male	Age-standardized	Aortic aneurysm	Rate	2019	129.826424	145.1807683	115.9998304	-37.89%
DALYs	Denmark	Male	All Ages	Aortic aneurysm	Number	1990	7391.544709	8190.565984	6614.683599	
DALYs	Denmark	Male	All Ages	Aortic aneurysm	Number	2019	7000.416282	7868.735093	6228.001162	-5.29%
Deaths	Denmark	Male	Age-standardized	Aortic aneurysm	Rate	1990	10.90020432	12.05804342	9.803389845	
Deaths	Denmark	Male	Age-standardized	Aortic aneurysm	Rate	2019	7.973077998	8.966293951	7.06804237	-26.85%
Deaths	Denmark	Male	All Ages	Aortic aneurysm	Number	1990	386.1919558	429.7025532	345.5273039	
Deaths	Denmark	Male	All Ages	Aortic aneurysm	Number	2019	436.9895999	492.9889599	385.3825613	13.15%
DALYs	Djibouti	Male	Age-standardized	Aortic aneurysm	Rate	1990	76.3700116	119.69424	43.15336438	
DALYs	Djibouti	Male	Age-standardized	Aortic aneurysm	Rate	2019	58.75585667	88.01029014	38.17157064	-23.06%
DALYs	Djibouti	Male	All Ages	Aortic aneurysm	Number	1990	65.32523929	104.961039	36.48671573	
DALYs	Djibouti	Male	All Ages	Aortic aneurysm	Number	2019	205.2226588	314.6855761	127.4754504	214.16%
Deaths	Djibouti	Male	Age-standardized	Aortic aneurysm	Rate	1990	3.595947145	5.539470081	2.040487051	
Deaths	Djibouti	Male	Age-standardized	Aortic aneurysm	Rate	2019	2.859343125	4.264533484	1.93146212	-20.48%
Deaths	Djibouti	Male	All Ages	Aortic aneurysm	Number	1990	2.13247445	3.363703574	1.195847503	
Deaths	Djibouti	Male	All Ages	Aortic aneurysm	Number	2019	7.391737584	11.10815342	4.765246743	246.63%
DALYs	Dominica	Male	Age-standardized	Aortic aneurysm	Rate	1990	104.8379783	130.6677383	83.48514187	
DALYs	Dominica	Male	Age-standardized	Aortic aneurysm	Rate	2019	97.76234313	121.574584	77.61057636	-6.75%
DALYs	Dominica	Male	All Ages	Aortic aneurysm	Number	1990	31.66920027	39.48504375	25.27894165	
DALYs	Dominica	Male	All Ages	Aortic aneurysm	Number	2019	42.61100512	53.39227051	33.78257136	34.55%
Deaths	Dominica	Male	Age-standardized	Aortic aneurysm	Rate	1990	5.733702417	7.048736646	4.596268115	
Deaths	Dominica	Male	Age-standardized	Aortic aneurysm	Rate	2019	5.36877295	6.652461419	4.299121016	-6.36%
Deaths	Dominica	Male	All Ages	Aortic aneurysm	Number	1990	1.696788761	2.099277622	1.362550966	
Deaths	Dominica	Male	All Ages	Aortic aneurysm	Number	2019	2.250949478	2.79013592	1.793464826	32.66%
DALYs	Dominican Republic	Male	Age-standardized	Aortic aneurysm	Rate	1990	41.03623142	48.50078235	34.31774538	
DALYs	Dominican Republic	Male	Age-standardized	Aortic aneurysm	Rate	2019	63.01028751	84.2351437	46.41635589	53.55%
DALYs	Dominican Republic	Male	All Ages	Aortic aneurysm	Number	1990	804.0175027	953.5552604	672.6683307	
DALYs	Dominican Republic	Male	All Ages	Aortic aneurysm	Number	2019	2893.033562	3886.151034	2109.039685	259.82%
Deaths	Dominican Republic	Male	Age-standardized	Aortic aneurysm	Rate	1990	1.979835995	2.340582485	1.661570108	
Deaths	Dominican Republic	Male	Age-standardized	Aortic aneurysm	Rate	2019	2.987649769	3.908697228	2.270425446	50.90%
Deaths	Dominican Republic	Male	All Ages	Aortic aneurysm	Number	1990	33.1665263	39.1582712	27.84179495	
Deaths	Dominican Republic	Male	All Ages	Aortic aneurysm	Number	2019	125.8416056	165.8930116	94.5873931	279.42%
DALYs	Ecuador	Male	Age-standardized	Aortic aneurysm	Rate	1990	45.12351527	51.87523657	38.99754342	
DALYs	Ecuador	Male	Age-standardized	Aortic aneurysm	Rate	2019	49.34342221	65.09193436	37.26502697	9.35%
DALYs	Ecuador	Male	All Ages	Aortic aneurysm	Number	1990	1261.025737	1452.012995	1100.561394	
DALYs	Ecuador	Male	All Ages	Aortic aneurysm	Number	2019	3582.304919	4732.635579	2703.729	184.08%
Deaths	Ecuador	Male	Age-standardized	Aortic aneurysm	Rate	1990	2.218734897	2.596784862	1.896396801	
Deaths	Ecuador	Male	Age-standardized	Aortic aneurysm	Rate	2019	2.628443245	3.370886399	2.028412792	18.47%
Deaths	Ecuador	Male	All Ages	Aortic aneurysm	Number	1990	53.87903471	62.9253025	46.40811608	
Deaths	Ecuador	Male	All Ages	Aortic aneurysm	Number	2019	170.4026841	220.7162846	131.0678742	216.27%
DALYs	Egypt	Male	Age-standardized	Aortic aneurysm	Rate	1990	37.22985532	53.93837349	23.91819451	
DALYs	Egypt	Male	Age-standardized	Aortic aneurysm	Rate	2019	42.52286238	64.50793377	29.15696566	14.22%
DALYs	Egypt	Male	All Ages	Aortic aneurysm	Number	1990	5941.309863	8587.583549	3795.661039	
DALYs	Egypt	Male	All Ages	Aortic aneurysm	Number	2019	15530.35276	23618.70563	10567.1333	161.40%
Deaths	Egypt	Male	Age-standardized	Aortic aneurysm	Rate	1990	1.76389784	2.582531226	1.124303242	
Deaths	Egypt	Male	Age-standardized	Aortic aneurysm	Rate	2019	2.003705108	3.048768814	1.402026279	13.60%
Deaths	Egypt	Male	All Ages	Aortic aneurysm	Number	1990	220.9198558	321.5437128	142.1261663	
Deaths	Egypt	Male	All Ages	Aortic aneurysm	Number	2019	608.0693739	922.0135967	419.0614658	175.24%
DALYs	El Salvador	Male	Age-standardized	Aortic aneurysm	Rate	1990	28.37450914	33.75320177	23.97121906	
DALYs	El Salvador	Male	Age-standardized	Aortic aneurysm	Rate	2019	31.86200134	41.98939886	23.33687022	12.29%
DALYs	El Salvador	Male	All Ages	Aortic aneurysm	Number	1990	416.7865321	496.4046503	352.7614732	
DALYs	El Salvador	Male	All Ages	Aortic aneurysm	Number	2019	813.2562448	1068.281512	599.8936916	95.13%
Deaths	El Salvador	Male	Age-standardized	Aortic aneurysm	Rate	1990	1.3448745	1.629337551	1.119532132	
Deaths	El Salvador	Male	Age-standardized	Aortic aneurysm	Rate	2019	1.535884279	2.030009664	1.140213861	14.20%
Deaths	El Salvador	Male	All Ages	Aortic aneurysm	Number	1990	17.6638833	21.30837253	14.80132341	
Deaths	El Salvador	Male	All Ages	Aortic aneurysm	Number	2019	39.57636793	52.14278838	29.36652712	124.05%
DALYs	Equatorial Guinea	Male	Age-standardized	Aortic aneurysm	Rate	1990	114.4194582	203.4202587	44.53076795	
DALYs	Equatorial Guinea	Male	Age-standardized	Aortic aneurysm	Rate	2019	80.41691825	128.5155401	51.83590344	-29.72%
DALYs	Equatorial Guinea	Male	All Ages	Aortic aneurysm	Number	1990	105.4482594	192.4095113	41.51107325	
DALYs	Equatorial Guinea	Male	All Ages	Aortic aneurysm	Number	2019	176.7164176	287.6354602	111.0398528	67.59%
Deaths	Equatorial Guinea	Male	Age-standardized	Aortic aneurysm	Rate	1990	5.097695393	8.664082903	1.930605188	
Deaths	Equatorial Guinea	Male	Age-standardized	Aortic aneurysm	Rate	2019	4.125507917	6.563449943	2.759342827	-19.07%
Deaths	Equatorial Guinea	Male	All Ages	Aortic aneurysm	Number	1990	3.932850023	6.989861404	1.513136058	
Deaths	Equatorial Guinea	Male	All Ages	Aortic aneurysm	Number	2019	6.907861003	10.96736178	4.476287033	75.65%
DALYs	Eritrea	Male	Age-standardized	Aortic aneurysm	Rate	1990	102.1384644	201.9569824	43.72946407	
DALYs	Eritrea	Male	Age-standardized	Aortic aneurysm	Rate	2019	72.65101742	117.5517102	45.785777	-28.87%
DALYs	Eritrea	Male	All Ages	Aortic aneurysm	Number	1990	524.9222762	1100.559852	228.681188	
DALYs	Eritrea	Male	All Ages	Aortic aneurysm	Number	2019	1023.341853	1758.804963	619.8604565	94.95%
Deaths	Eritrea	Male	Age-standardized	Aortic aneurysm	Rate	1990	4.322404801	8.358884336	1.790444727	
Deaths	Eritrea	Male	Age-standardized	Aortic aneurysm	Rate	2019	3.261394103	5.108927397	2.049499925	-24.55%
Deaths	Eritrea	Male	All Ages	Aortic aneurysm	Number	1990	16.09232504	32.50504639	6.9409414	
Deaths	Eritrea	Male	All Ages	Aortic aneurysm	Number	2019	32.11578242	52.78993719	20.28646446	99.57%

DALYs	Estonia	Male	Age-standardized	Aortic aneurysm	Rate	1990	102.0705156	120.3968203	87.26456518	
DALYs	Estonia	Male	Age-standardized	Aortic aneurysm	Rate	2019	107.5423694	140.9797762	80.87872485	5.36%
DALYs	Estonia	Male	All Ages	Aortic aneurysm	Number	1990	777.3598595	917.7523264	661.3264701	
DALYs	Estonia	Male	All Ages	Aortic aneurysm	Number	2019	1027.706837	1350.886532	770.6636538	32.20%
Deaths	Estonia	Male	Age-standardized	Aortic aneurysm	Rate	1990	4.553093907	5.31224463	3.919253209	
Deaths	Estonia	Male	Age-standardized	Aortic aneurysm	Rate	2019	5.05365133	6.593320486	3.840491178	10.99%
Deaths	Estonia	Male	All Ages	Aortic aneurysm	Number	1990	31.62753754	37.12480037	27.04251887	
Deaths	Estonia	Male	All Ages	Aortic aneurysm	Number	2019	49.17232627	64.28059397	37.09345932	55.47%
DALYs	Eswatini	Male	Age-standardized	Aortic aneurysm	Rate	1990	80.29502609	110.5930662	53.34607582	
DALYs	Eswatini	Male	Age-standardized	Aortic aneurysm	Rate	2019	84.84084436	114.2824399	60.09508115	5.66%
DALYs	Eswatini	Male	All Ages	Aortic aneurysm	Number	1990	101.0184867	141.1454105	65.84701031	
DALYs	Eswatini	Male	All Ages	Aortic aneurysm	Number	2019	205.5273727	286.5245466	135.4618776	103.46%
Deaths	Eswatini	Male	Age-standardized	Aortic aneurysm	Rate	1990	4.177668761	5.695793087	2.837753467	
Deaths	Eswatini	Male	Age-standardized	Aortic aneurysm	Rate	2019	4.349305596	5.722820166	3.21161038	4.11%
Deaths	Eswatini	Male	All Ages	Aortic aneurysm	Number	1990	3.967187543	5.471601168	2.648897622	
Deaths	Eswatini	Male	All Ages	Aortic aneurysm	Number	2019	7.546654377	10.23514279	5.283361666	90.23%
DALYs	Ethiopia	Male	Age-standardized	Aortic aneurysm	Rate	1990	72.39810589	154.8270526	29.81728927	
DALYs	Ethiopia	Male	Age-standardized	Aortic aneurysm	Rate	2019	37.10517501	54.73836039	22.28565437	-48.75%
DALYs	Ethiopia	Male	All Ages	Aortic aneurysm	Number	1990	8231.697156	17903.23314	3201.152283	
DALYs	Ethiopia	Male	All Ages	Aortic aneurysm	Number	2019	8410.935114	12297.89215	5076.925324	2.18%
Deaths	Ethiopia	Male	Age-standardized	Aortic aneurysm	Rate	1990	3.201066396	6.657182952	1.376256392	
Deaths	Ethiopia	Male	Age-standardized	Aortic aneurysm	Rate	2019	1.877476239	2.729375976	1.120398701	-41.35%
Deaths	Ethiopia	Male	All Ages	Aortic aneurysm	Number	1990	295.8849477	632.3852107	120.3542621	
Deaths	Ethiopia	Male	All Ages	Aortic aneurysm	Number	2019	349.5556219	512.8111702	208.4302473	18.14%
DALYs	Fiji	Male	Age-standardized	Aortic aneurysm	Rate	1990	137.4229447	179.294069	103.8612933	
DALYs	Fiji	Male	Age-standardized	Aortic aneurysm	Rate	2019	136.4727358	171.8916009	104.6021841	-0.69%
DALYs	Fiji	Male	All Ages	Aortic aneurysm	Number	1990	274.0730539	359.5024868	203.9072798	
DALYs	Fiji	Male	All Ages	Aortic aneurysm	Number	2019	480.8090915	623.2083728	361.8507477	75.43%
Deaths	Fiji	Male	Age-standardized	Aortic aneurysm	Rate	1990	6.509178735	8.415447683	5.032815592	
Deaths	Fiji	Male	Age-standardized	Aortic aneurysm	Rate	2019	6.902489598	8.434201433	5.453064166	6.04%
Deaths	Fiji	Male	All Ages	Aortic aneurysm	Number	1990	9.483384097	12.35726591	7.166763969	
Deaths	Fiji	Male	All Ages	Aortic aneurysm	Number	2019	18.24244824	23.2977199	13.85621905	92.36%
DALYs	Finland	Male	Age-standardized	Aortic aneurysm	Rate	1990	262.2834219	300.2926703	219.3344736	
DALYs	Finland	Male	Age-standardized	Aortic aneurysm	Rate	2019	133.7915147	149.3038562	120.593192	-48.99%
DALYs	Finland	Male	All Ages	Aortic aneurysm	Number	1990	7363.713536	8450.299383	6110.666701	
DALYs	Finland	Male	All Ages	Aortic aneurysm	Number	2019	7128.722404	7992.033006	6379.855398	-3.19%
Deaths	Finland	Male	Age-standardized	Aortic aneurysm	Rate	1990	13.55766377	15.47895105	11.4379684	
Deaths	Finland	Male	Age-standardized	Aortic aneurysm	Rate	2019	7.455574836	8.323791574	6.648191008	-45.01%
Deaths	Finland	Male	All Ages	Aortic aneurysm	Number	1990	356.8985448	408.0616288	300.0365843	
Deaths	Finland	Male	All Ages	Aortic aneurysm	Number	2019	418.8322056	469.5284399	370.5972607	17.35%
DALYs	France	Male	Age-standardized	Aortic aneurysm	Rate	1990	114.9132193	120.6348133	108.6309806	
DALYs	France	Male	Age-standardized	Aortic aneurysm	Rate	2019	64.04680294	70.7444461	57.80052678	-44.27%
DALYs	France	Male	All Ages	Aortic aneurysm	Number	1990	38974.12716	41069.19995	36851.75325	
DALYs	France	Male	All Ages	Aortic aneurysm	Number	2019	36576.11199	40672.64383	32884.11432	-6.15%
Deaths	France	Male	Age-standardized	Aortic aneurysm	Rate	1990	6.197201966	6.565093412	5.806048027	
Deaths	France	Male	Age-standardized	Aortic aneurysm	Rate	2019	3.585416641	3.983489986	3.201550278	-42.14%
Deaths	France	Male	All Ages	Aortic aneurysm	Number	1990	2041.465422	2166.850755	1913.30452	
Deaths	France	Male	All Ages	Aortic aneurysm	Number	2019	2257.261646	2514.821016	2009.174451	10.57%
DALYs	Gabon	Male	Age-standardized	Aortic aneurysm	Rate	1990	140.028492	207.1081457	92.43356335	
DALYs	Gabon	Male	Age-standardized	Aortic aneurysm	Rate	2019	115.5605553	155.9405152	83.21019221	-17.47%
DALYs	Gabon	Male	All Ages	Aortic aneurysm	Number	1990	364.9001081	543.6506155	236.2828292	
DALYs	Gabon	Male	All Ages	Aortic aneurysm	Number	2019	580.4899084	791.9230166	408.2504639	59.08%
Deaths	Gabon	Male	Age-standardized	Aortic aneurysm	Rate	1990	6.501516845	9.503661257	4.387159616	
Deaths	Gabon	Male	Age-standardized	Aortic aneurysm	Rate	2019	5.626119866	7.504877365	4.196690389	-13.46%
Deaths	Gabon	Male	All Ages	Aortic aneurysm	Number	1990	14.36706075	21.2871768	9.494165212	
Deaths	Gabon	Male	All Ages	Aortic aneurysm	Number	2019	22.73031871	30.55907636	16.39042811	58.21%
DALYs	Gambia	Male	Age-standardized	Aortic aneurysm	Rate	1990	41.3123835	63.92033686	22.38045015	
DALYs	Gambia	Male	Age-standardized	Aortic aneurysm	Rate	2019	51.24069277	68.52393923	36.84448304	24.03%
DALYs	Gambia	Male	All Ages	Aortic aneurysm	Number	1990	79.83916638	125.9206446	42.89501696	
DALYs	Gambia	Male	All Ages	Aortic aneurysm	Number	2019	249.513625	344.3532802	175.6112307	212.52%
Deaths	Gambia	Male	Age-standardized	Aortic aneurysm	Rate	1990	2.06956612	3.149657303	1.134593287	
Deaths	Gambia	Male	Age-standardized	Aortic aneurysm	Rate	2019	2.603173022	3.402050877	1.921185708	25.78%
Deaths	Gambia	Male	All Ages	Aortic aneurysm	Number	1990	3.13247069	4.856388048	1.684507112	
Deaths	Gambia	Male	All Ages	Aortic aneurysm	Number	2019	10.46743801	14.02576575	7.594796428	234.16%
DALYs	Georgia	Male	Age-standardized	Aortic aneurysm	Rate	1990	28.29339295	35.0787879	18.85420784	
DALYs	Georgia	Male	Age-standardized	Aortic aneurysm	Rate	2019	100.6945491	120.3769569	80.85151658	255.89%
DALYs	Georgia	Male	All Ages	Aortic aneurysm	Number	1990	669.5433266	835.125082	443.291928	
DALYs	Georgia	Male	All Ages	Aortic aneurysm	Number	2019	2360.402598	2856.229531	1892.893678	252.54%
Deaths	Georgia	Male	Age-standardized	Aortic aneurysm	Rate	1990	1.449892921	1.790492153	0.959789872	
Deaths	Georgia	Male	Age-standardized	Aortic aneurysm	Rate	2019	4.317791276	5.116244646	3.45630546	197.80%
Deaths	Georgia	Male	All Ages	Aortic aneurysm	Number	1990	28.06289754	35.08351853	18.70056688	
Deaths	Georgia	Male	All Ages	Aortic aneurysm	Number	2019	101.8176419	120.4944168	81.56147972	262.82%
DALYs	Germany	Male	Age-standardized	Aortic aneurysm	Rate	1990	110.6395007	122.6256827	97.84286216	
DALYs	Germany	Male	Age-standardized	Aortic aneurysm	Rate	2019	65.08473188	70.72720975	59.53722238	-41.17%
DALYs	Germany	Male	All Ages	Aortic aneurysm	Number	1990	52614.34355	58483.2531	46511.53211	
DALYs	Germany	Male	All Ages	Aortic aneurysm	Number	2019	51110.98595	55862.58353	46501.58462	-2.86%
Deaths	Germany	Male	Age-standardized	Aortic aneurysm	Rate	1990	5.978690288	6.647646027	5.279492911	
Deaths	Germany	Male	Age-standardized	Aortic aneurysm	Rate	2019	3.337806909	3.649224354	3.020408619	-44.17%
Deaths	Germany	Male	All Ages	Aortic aneurysm	Number	1990	2720.759093	3046.491729	2382.665945	
Deaths	Germany	Male	All Ages	Aortic aneurysm	Number	2019	2890.956811	3197.714415	2601.629087	6.26%
DALYs	Ghana	Male	Age-standardized	Aortic aneurysm	Rate	1990	38.82692019	49.63671937	29.59142211	
DALYs	Ghana	Male	Age-standardized	Aortic aneurysm	Rate	2019	35.93711766	47.99120056	26.22342429	-7.44%
DALYs	Ghana	Male	All Ages	Aortic aneurysm	Number	1990	1221.955521	1581.632478	921.9790372	
DALYs	Ghana	Male	All Ages	Aortic aneurysm	Number	2019	2611.412773	3533.73294	1838.676883	113.71%
Deaths	Ghana	Male	Age-standardized	Aortic aneurysm	Rate	1990	1.896018937	2.390414429	1.475545134	
Deaths	Ghana	Male	Age-standardized	Aortic aneurysm	Rate	2019	1.871646817	2.482782435	1.393316511	-1.29%
Deaths	Ghana	Male	All Ages	Aortic aneurysm	Number	1990	47.40464076	60.70680001	36.22640724	
Deaths	Ghana	Male	All Ages	Aortic aneurysm	Number	2019	105.7378693	141.428547	77.16037087	123.05%
DALYs	Greece	Male	Age-standardized	Aortic aneurysm	Rate	1990	120.1481445	126.8312118	113.5414459	
DALYs	Greece	Male	Age-standardized	Aortic aneurysm	Rate	2019	154.5818583	172.8525556	138.462302	28.66%
DALYs	Greece	Male	All Ages	Aortic aneurysm	Number	1990	8127.691636	8591.338993	7686.526654	
DALYs	Greece	Male	All Ages	Aortic aneurysm	Number	2019	14519.48648	16191.57122	12970.72758	78.64%
Deaths	Greece	Male	Age-standardized	Aortic aneurysm	Rate	1990	5.744672537	6.1054952	5.398036404	
Deaths	Greece	Male	Age-standardized	Aortic aneurysm	Rate	2019	7.207884873	8.016051242	6.410850916	25.47%
Deaths	Greece	Male	All Ages	Aortic aneurysm	Number	1990	386.8542667	411.479829	364.0585933	
Deaths	Greece	Male	All Ages	Aortic aneurysm	Number	2019	801.1500667	892.0438186	706.5509157	107.09%
DALYs	Greenland	Male	Age-standardized	Aortic aneurysm	Rate	1990	78.79308933	95.47028734	63.68510233	
DALYs	Greenland	Male	Age-standardized	Aortic aneurysm	Rate	2019	46.93887361	57.14673011	36.9560257	-40.43%
DALYs	Greenland	Male	All Ages	Aortic aneurysm	Number	1990	13.63414042	16.86728446	11.0213319	
DALYs	Greenland	Male	All Ages	Aortic aneurysm	Number	2019	17.09588589	20.9773175	13.2114045	25.39%
Deaths	Greenland	Male	Age-standardized	Aortic aneurysm	Rate	1990	3.923864842	4.745031139	3.174295578	

Deaths	Greenland	Male	Age-standardized	Aortic aneurysm	Rate	2019	2.447709413	2.95637809	1.949990082	-37.62%
Deaths	Greenland	Male	All Ages	Aortic aneurysm	Number	1990	0.517018351	0.629469133	0.417674431	
Deaths	Greenland	Male	All Ages	Aortic aneurysm	Number	2019	0.767031376	0.929811797	0.604251438	48.36%
DALYs	Grenada	Male	Age-standardized	Aortic aneurysm	Rate	1990	147.8379842	183.3719024	121.2569507	
DALYs	Grenada	Male	Age-standardized	Aortic aneurysm	Rate	2019	145.9590074	165.6123796	127.2955805	-1.27%
DALYs	Grenada	Male	All Ages	Aortic aneurysm	Number	1990	45.44062448	56.98046594	37.07434547	
DALYs	Grenada	Male	All Ages	Aortic aneurysm	Number	2019	75.4430372	85.82690454	65.49981823	66.03%
Deaths	Grenada	Male	Age-standardized	Aortic aneurysm	Rate	1990	7.841395369	10.30669241	6.237152907	
Deaths	Grenada	Male	Age-standardized	Aortic aneurysm	Rate	2019	7.841116155	8.918891737	6.825388004	0.00%
Deaths	Grenada	Male	All Ages	Aortic aneurysm	Number	1990	2.461305286	3.26490839	1.951385317	
Deaths	Grenada	Male	All Ages	Aortic aneurysm	Number	2019	3.443895577	3.933971437	2.989992296	39.92%
DALYs	Guam	Male	Age-standardized	Aortic aneurysm	Rate	1990	235.9980138	279.3502758	198.7917099	
DALYs	Guam	Male	Age-standardized	Aortic aneurysm	Rate	2019	87.4699441	105.7384087	71.0936575	-62.94%
DALYs	Guam	Male	All Ages	Aortic aneurysm	Number	1990	96.66711283	116.5705578	81.14515182	
DALYs	Guam	Male	All Ages	Aortic aneurysm	Number	2019	80.2280491	97.59750113	64.75262066	-17.01%
Deaths	Guam	Male	Age-standardized	Aortic aneurysm	Rate	1990	12.00670215	14.19524193	10.02572162	
Deaths	Guam	Male	Age-standardized	Aortic aneurysm	Rate	2019	3.82153183	4.597859834	3.110262691	-68.17%
Deaths	Guam	Male	All Ages	Aortic aneurysm	Number	1990	3.580755066	4.261929878	3.000665051	
Deaths	Guam	Male	All Ages	Aortic aneurysm	Number	2019	3.297023717	3.987731632	2.67916324	-7.92%
DALYs	Guatemala	Male	Age-standardized	Aortic aneurysm	Rate	1990	32.68442427	40.75158464	26.31952878	
DALYs	Guatemala	Male	Age-standardized	Aortic aneurysm	Rate	2019	30.86199548	39.61989288	24.23476821	-5.58%
DALYs	Guatemala	Male	All Ages	Aortic aneurysm	Number	1990	610.1046839	770.0843443	488.5837307	
DALYs	Guatemala	Male	All Ages	Aortic aneurysm	Number	2019	1577.018255	2040.187563	1220.773429	158.48%
Deaths	Guatemala	Male	Age-standardized	Aortic aneurysm	Rate	1990	1.685159259	2.072324621	1.343607235	
Deaths	Guatemala	Male	Age-standardized	Aortic aneurysm	Rate	2019	1.716428553	2.131498407	1.386222293	1.86%
Deaths	Guatemala	Male	All Ages	Aortic aneurysm	Number	1990	24.79578821	31.03866867	20.0230082	
Deaths	Guatemala	Male	All Ages	Aortic aneurysm	Number	2019	73.69873588	93.87897064	57.94156405	197.22%
DALYs	Guinea	Male	Age-standardized	Aortic aneurysm	Rate	1990	39.99975436	64.99309121	21.28609141	
DALYs	Guinea	Male	Age-standardized	Aortic aneurysm	Rate	2019	49.69167787	76.47275816	32.42966915	24.23%
DALYs	Guinea	Male	All Ages	Aortic aneurysm	Number	1990	676.375447	1098.541581	358.5755947	
DALYs	Guinea	Male	All Ages	Aortic aneurysm	Number	2019	1435.540886	2195.178136	926.3112349	112.24%
Deaths	Guinea	Male	Age-standardized	Aortic aneurysm	Rate	1990	1.955540386	3.140868607	1.036615729	
Deaths	Guinea	Male	Age-standardized	Aortic aneurysm	Rate	2019	2.422636352	3.697701794	1.640899761	23.89%
Deaths	Guinea	Male	All Ages	Aortic aneurysm	Number	1990	29.65718799	48.13904401	15.6859297	
Deaths	Guinea	Male	All Ages	Aortic aneurysm	Number	2019	60.58357711	92.15475664	40.25092151	104.28%
DALYs	Guinea-Bissau	Male	Age-standardized	Aortic aneurysm	Rate	1990	65.14512129	116.1668743	30.82743101	
DALYs	Guinea-Bissau	Male	Age-standardized	Aortic aneurysm	Rate	2019	64.43772711	94.79121313	42.84592174	-1.09%
DALYs	Guinea-Bissau	Male	All Ages	Aortic aneurysm	Number	1990	136.8269041	246.1478221	63.33885114	
DALYs	Guinea-Bissau	Male	All Ages	Aortic aneurysm	Number	2019	233.5765635	349.1246419	151.0799796	70.71%
Deaths	Guinea-Bissau	Male	Age-standardized	Aortic aneurysm	Rate	1990	2.962604329	5.113349478	1.455395788	
Deaths	Guinea-Bissau	Male	Age-standardized	Aortic aneurysm	Rate	2019	3.03373934	4.405909361	2.075524917	2.40%
Deaths	Guinea-Bissau	Male	All Ages	Aortic aneurysm	Number	1990	5.293389322	9.388983492	2.494782416	
Deaths	Guinea-Bissau	Male	All Ages	Aortic aneurysm	Number	2019	8.391265407	12.39360983	5.558671583	58.52%
DALYs	Guyana	Male	Age-standardized	Aortic aneurysm	Rate	1990	73.39017423	85.71863778	62.15012085	
DALYs	Guyana	Male	Age-standardized	Aortic aneurysm	Rate	2019	116.2057388	147.9701349	88.88467928	58.34%
DALYs	Guyana	Male	All Ages	Aortic aneurysm	Number	1990	138.7619284	162.1618708	116.8453913	
DALYs	Guyana	Male	All Ages	Aortic aneurysm	Number	2019	347.2808697	446.192061	262.1973433	150.27%
Deaths	Guyana	Male	Age-standardized	Aortic aneurysm	Rate	1990	3.572421515	4.147273882	3.049157615	
Deaths	Guyana	Male	Age-standardized	Aortic aneurysm	Rate	2019	5.583061044	6.94868779	4.32432195	56.28%
Deaths	Guyana	Male	All Ages	Aortic aneurysm	Number	1990	5.751201222	6.692824368	4.884716936	
Deaths	Guyana	Male	All Ages	Aortic aneurysm	Number	2019	14.23380447	18.03000728	10.92589768	147.49%
DALYs	Haiti	Male	Age-standardized	Aortic aneurysm	Rate	1990	98.72201582	162.1947225	53.86537302	
DALYs	Haiti	Male	Age-standardized	Aortic aneurysm	Rate	2019	88.2569929	139.5611887	49.01662128	-10.60%
DALYs	Haiti	Male	All Ages	Aortic aneurysm	Number	1990	1548.877179	2558.411378	818.7978968	
DALYs	Haiti	Male	All Ages	Aortic aneurysm	Number	2019	2869.355331	4557.075943	1555.279879	85.25%
Deaths	Haiti	Male	Age-standardized	Aortic aneurysm	Rate	1990	4.888557178	7.911007881	2.679057313	
Deaths	Haiti	Male	Age-standardized	Aortic aneurysm	Rate	2019	4.544815937	7.103416168	2.573778805	-7.03%
Deaths	Haiti	Male	All Ages	Aortic aneurysm	Number	1990	63.03928069	103.6372924	34.31227017	
Deaths	Haiti	Male	All Ages	Aortic aneurysm	Number	2019	124.03769	195.7879122	69.00670468	96.76%
DALYs	Honduras	Male	Age-standardized	Aortic aneurysm	Rate	1990	27.64461742	43.24673273	15.90776559	
DALYs	Honduras	Male	Age-standardized	Aortic aneurysm	Rate	2019	39.35826295	53.07489662	27.586321	42.37%
DALYs	Honduras	Male	All Ages	Aortic aneurysm	Number	1990	283.3930649	440.7845176	163.824792	
DALYs	Honduras	Male	All Ages	Aortic aneurysm	Number	2019	1090.992602	1484.436233	763.1279547	284.98%
Deaths	Honduras	Male	Age-standardized	Aortic aneurysm	Rate	1990	1.406689277	2.359820966	0.794965497	
Deaths	Honduras	Male	Age-standardized	Aortic aneurysm	Rate	2019	2.132001933	2.825843043	1.499662555	51.56%
Deaths	Honduras	Male	All Ages	Aortic aneurysm	Number	1990	12.52839141	20.21157916	7.197323913	
Deaths	Honduras	Male	All Ages	Aortic aneurysm	Number	2019	53.14291084	70.11070781	37.50418721	324.18%
DALYs	Hungary	Male	Age-standardized	Aortic aneurysm	Rate	1990	92.16265532	98.30368632	86.72136553	
DALYs	Hungary	Male	Age-standardized	Aortic aneurysm	Rate	2019	89.94168978	109.8706593	73.17598959	-2.41%
DALYs	Hungary	Male	All Ages	Aortic aneurysm	Number	1990	5574.75045	5968.717496	5228.804448	
DALYs	Hungary	Male	All Ages	Aortic aneurysm	Number	2019	6715.112089	8275.134822	5451.393022	20.46%
Deaths	Hungary	Male	Age-standardized	Aortic aneurysm	Rate	1990	4.03197965	4.293338551	3.771325039	
Deaths	Hungary	Male	Age-standardized	Aortic aneurysm	Rate	2019	4.238427772	5.084375426	3.492824669	5.12%
Deaths	Hungary	Male	All Ages	Aortic aneurysm	Number	1990	231.3353792	246.9220105	216.5532274	
Deaths	Hungary	Male	All Ages	Aortic aneurysm	Number	2019	318.4778693	384.9429798	260.9632224	37.67%
DALYs	Iceland	Male	Age-standardized	Aortic aneurysm	Rate	1990	116.8816017	130.2673657	105.3966203	
DALYs	Iceland	Male	Age-standardized	Aortic aneurysm	Rate	2019	79.49665642	89.49743913	69.23823926	-31.99%
DALYs	Iceland	Male	All Ages	Aortic aneurysm	Number	1990	153.1911192	171.086515	137.6370196	
DALYs	Iceland	Male	All Ages	Aortic aneurysm	Number	2019	204.0720643	230.4888408	177.4805342	33.21%
Deaths	Iceland	Male	Age-standardized	Aortic aneurysm	Rate	1990	6.141725591	6.863200432	5.500418984	
Deaths	Iceland	Male	Age-standardized	Aortic aneurysm	Rate	2019	4.409534412	5.021702716	3.829609983	-28.20%
Deaths	Iceland	Male	All Ages	Aortic aneurysm	Number	1990	7.96815325	8.927525694	7.12780246	
Deaths	Iceland	Male	All Ages	Aortic aneurysm	Number	2019	11.75775758	13.42232232	10.19739164	47.56%
DALYs	India	Male	Age-standardized	Aortic aneurysm	Rate	1990	45.30623156	78.52262852	23.84607075	
DALYs	India	Male	Age-standardized	Aortic aneurysm	Rate	2019	46.27792757	62.36601931	29.73412988	2.14%
DALYs	India	Male	All Ages	Aortic aneurysm	Number	1990	106379.8396	185310.4903	55020.9145	
DALYs	India	Male	All Ages	Aortic aneurysm	Number	2019	259618.0447	351707.6736	164174.4851	144.05%
Deaths	India	Male	Age-standardized	Aortic aneurysm	Rate	1990	2.238143843	3.838632287	1.198791809	
Deaths	India	Male	Age-standardized	Aortic aneurysm	Rate	2019	2.300916532	3.050503169	1.527143616	2.80%
Deaths	India	Male	All Ages	Aortic aneurysm	Number	1990	4097.965964	7120.830343	2141.272745	
Deaths	India	Male	All Ages	Aortic aneurysm	Number	2019	11169.95009	14964.98892	7236.68077	172.57%
DALYs	Indonesia	Male	Age-standardized	Aortic aneurysm	Rate	1990	28.39900708	40.17242131	20.71272393	
DALYs	Indonesia	Male	Age-standardized	Aortic aneurysm	Rate	2019	42.84142996	62.21401864	26.67828817	50.86%
DALYs	Indonesia	Male	All Ages	Aortic aneurysm	Number	1990	13465.32903	19058.30691	9607.577392	
DALYs	Indonesia	Male	All Ages	Aortic aneurysm	Number	2019	41265.07627	61027.80687	25171.12752	206.45%
Deaths	Indonesia	Male	Age-standardized	Aortic aneurysm	Rate	1990	1.478824863	2.091899644	1.078920605	
Deaths	Indonesia	Male	Age-standardized	Aortic aneurysm	Rate	2019	2.382517987	3.431986591	1.516772141	61.11%
Deaths	Indonesia	Male	All Ages	Aortic aneurysm	Number	1990	539.5825133	762.6290052	395.1360224	
Deaths	Indonesia	Male	All Ages	Aortic aneurysm	Number	2019	1806.970441	2625.981284	1126.794731	234.88%
DALYs	Iran (Islamic Republic of)	Male	Age-standardized	Aortic aneurysm	Rate	1990	31.9022985	41.03839392	22.66524968	
DALYs	Iran (Islamic Republic of)	Male	Age-standardized	Aortic aneurysm	Rate	2019	29.80257402	33.44214773	26.07778279	-6.58%

DALYs	Iran (Islamic Republic of)	Male	All Ages	Aortic aneurysm	Number	1990	4611.719065	5995.585333	3297.387365	
DALYs	Iran (Islamic Republic of)	Male	All Ages	Aortic aneurysm	Number	2019	11041.86488	12437.52626	9693.222023	139.43%
Deaths	Iran (Islamic Republic of)	Male	Age-standardized	Aortic aneurysm	Rate	1990	1.567106066	1.990311838	1.109313177	
Deaths	Iran (Islamic Republic of)	Male	Age-standardized	Aortic aneurysm	Rate	2019	1.505537112	1.681963067	1.306847177	-3.93%
Deaths	Iran (Islamic Republic of)	Male	All Ages	Aortic aneurysm	Number	1990	171.3599148	219.1404707	121.7522842	
Deaths	Iran (Islamic Republic of)	Male	All Ages	Aortic aneurysm	Number	2019	501.3177671	559.5234308	436.5153814	192.55%
DALYs	Iraq	Male	Age-standardized	Aortic aneurysm	Rate	1990	24.74476957	34.80700899	16.78956644	
DALYs	Iraq	Male	Age-standardized	Aortic aneurysm	Rate	2019	26.53135883	33.30662809	20.1470581	7.22%
DALYs	Iraq	Male	All Ages	Aortic aneurysm	Number	1990	1067.819055	1508.811075	714.2168569	
DALYs	Iraq	Male	All Ages	Aortic aneurysm	Number	2019	3150.966953	4098.622917	2312.113299	195.08%
Deaths	Iraq	Male	Age-standardized	Aortic aneurysm	Rate	1990	1.118911021	1.547064759	0.765325887	
Deaths	Iraq	Male	Age-standardized	Aortic aneurysm	Rate	2019	1.349747319	1.649479383	1.083709771	20.63%
Deaths	Iraq	Male	All Ages	Aortic aneurysm	Number	1990	39.94489224	56.01042531	27.28694067	
Deaths	Iraq	Male	All Ages	Aortic aneurysm	Number	2019	125.0689914	156.0655633	95.74588738	213.10%
DALYs	Ireland	Male	Age-standardized	Aortic aneurysm	Rate	1990	160.4362662	171.328917	149.4745001	
DALYs	Ireland	Male	Age-standardized	Aortic aneurysm	Rate	2019	95.28075344	108.1129819	83.70431773	-40.61%
DALYs	Ireland	Male	All Ages	Aortic aneurysm	Number	1990	3030.593034	3248.690033	2821.418862	
DALYs	Ireland	Male	All Ages	Aortic aneurysm	Number	2019	3330.61205	3793.135204	2920.741495	9.90%
Deaths	Ireland	Male	Age-standardized	Aortic aneurysm	Rate	1990	8.636964937	9.258716867	8.043097694	
Deaths	Ireland	Male	Age-standardized	Aortic aneurysm	Rate	2019	5.657788226	6.39474782	4.893173459	-34.49%
Deaths	Ireland	Male	All Ages	Aortic aneurysm	Number	1990	156.0967496	167.5928439	145.0064007	
Deaths	Ireland	Male	All Ages	Aortic aneurysm	Number	2019	196.8968693	223.97131	170.5590109	26.14%
DALYs	Israel	Male	Age-standardized	Aortic aneurysm	Rate	1990	60.23419988	68.69910749	54.05093223	
DALYs	Israel	Male	Age-standardized	Aortic aneurysm	Rate	2019	45.59527061	50.59325129	40.34949294	-24.30%
DALYs	Israel	Male	All Ages	Aortic aneurysm	Number	1990	1344.023667	1539.901448	1204.033205	
DALYs	Israel	Male	All Ages	Aortic aneurysm	Number	2019	2365.067478	2627.317853	2091.388437	75.97%
Deaths	Israel	Male	Age-standardized	Aortic aneurysm	Rate	1990	3.226493023	3.707630683	2.865587866	
Deaths	Israel	Male	Age-standardized	Aortic aneurysm	Rate	2019	2.513260139	2.808827879	2.209925681	-22.11%
Deaths	Israel	Male	All Ages	Aortic aneurysm	Number	1990	70.26225187	81.20725089	62.17819729	
Deaths	Israel	Male	All Ages	Aortic aneurysm	Number	2019	133.407349	148.9378217	117.0531494	89.87%
DALYs	Italy	Male	Age-standardized	Aortic aneurysm	Rate	1990	105.3987721	109.3409916	102.1793505	
DALYs	Italy	Male	Age-standardized	Aortic aneurysm	Rate	2019	75.06164767	81.72002769	69.15197024	-28.78%
DALYs	Italy	Male	All Ages	Aortic aneurysm	Number	1990	40005.3111	41461.94927	38812.40193	
DALYs	Italy	Male	All Ages	Aortic aneurysm	Number	2019	44700.23164	48624.53893	41116.31581	11.74%
Deaths	Italy	Male	Age-standardized	Aortic aneurysm	Rate	1990	5.233134015	5.512275908	5.007798859	
Deaths	Italy	Male	Age-standardized	Aortic aneurysm	Rate	2019	4.09122046	4.440392412	3.720968289	-21.82%
Deaths	Italy	Male	All Ages	Aortic aneurysm	Number	1990	1946.242625	2034.808331	1873.148332	
Deaths	Italy	Male	All Ages	Aortic aneurysm	Number	2019	2712.864351	2949.733028	2447.402895	39.39%
DALYs	Jamaica	Male	Age-standardized	Aortic aneurysm	Rate	1990	33.90273383	39.78696425	29.26688314	
DALYs	Jamaica	Male	Age-standardized	Aortic aneurysm	Rate	2019	65.01268749	82.97150955	50.52252105	91.76%
DALYs	Jamaica	Male	All Ages	Aortic aneurysm	Number	1990	284.7563426	336.1238774	245.2134058	
DALYs	Jamaica	Male	All Ages	Aortic aneurysm	Number	2019	928.6190067	1192.064842	720.0453971	226.11%
Deaths	Jamaica	Male	Age-standardized	Aortic aneurysm	Rate	1990	1.643577592	1.891668626	1.414261278	
Deaths	Jamaica	Male	Age-standardized	Aortic aneurysm	Rate	2019	3.072036606	3.839467465	2.408418589	86.91%
Deaths	Jamaica	Male	All Ages	Aortic aneurysm	Number	1990	13.58882206	15.74232092	11.68897615	
Deaths	Jamaica	Male	All Ages	Aortic aneurysm	Number	2019	43.09439112	54.02645665	33.87199217	217.13%
DALYs	Japan	Male	Age-standardized	Aortic aneurysm	Rate	1990	80.04691995	82.53939583	77.11524719	
DALYs	Japan	Male	Age-standardized	Aortic aneurysm	Rate	2019	110.2547909	119.7146149	101.791571	37.74%
DALYs	Japan	Male	All Ages	Aortic aneurysm	Number	1990	57312.36874	59124.25045	55382.72323	
DALYs	Japan	Male	All Ages	Aortic aneurysm	Number	2019	155624.4182	168632.6393	141361.1961	171.54%
Deaths	Japan	Male	Age-standardized	Aortic aneurysm	Rate	1990	4.572483622	4.742435583	4.330253569	
Deaths	Japan	Male	Age-standardized	Aortic aneurysm	Rate	2019	5.949296573	6.478797157	5.284689571	30.11%
Deaths	Japan	Male	All Ages	Aortic aneurysm	Number	1990	2978.743037	3081.865412	2848.626929	
Deaths	Japan	Male	All Ages	Aortic aneurysm	Number	2019	10089.96385	10992.20903	8807.113578	238.73%
DALYs	Jordan	Male	Age-standardized	Aortic aneurysm	Rate	1990	56.96599379	76.75284091	42.42786608	
DALYs	Jordan	Male	Age-standardized	Aortic aneurysm	Rate	2019	40.00385301	53.01843028	30.15569255	-29.78%
DALYs	Jordan	Male	All Ages	Aortic aneurysm	Number	1990	483.4240201	651.201955	356.0412916	
DALYs	Jordan	Male	All Ages	Aortic aneurysm	Number	2019	1625.586655	2153.445385	1212.227344	236.27%
Deaths	Jordan	Male	Age-standardized	Aortic aneurysm	Rate	1990	2.520811577	3.381327833	1.881484993	
Deaths	Jordan	Male	Age-standardized	Aortic aneurysm	Rate	2019	1.841419223	2.410558775	1.386620916	-26.95%
Deaths	Jordan	Male	All Ages	Aortic aneurysm	Number	1990	15.93742651	21.38019516	11.85434437	
Deaths	Jordan	Male	All Ages	Aortic aneurysm	Number	2019	57.61275924	75.89615046	43.30297286	261.49%
DALYs	Kazakhstan	Male	Age-standardized	Aortic aneurysm	Rate	1990	63.56025086	89.5225435	52.86704166	
DALYs	Kazakhstan	Male	Age-standardized	Aortic aneurysm	Rate	2019	94.9325116	112.8007639	79.56390749	49.36%
DALYs	Kazakhstan	Male	All Ages	Aortic aneurysm	Number	1990	3502.966964	4876.328615	2925.340788	
DALYs	Kazakhstan	Male	All Ages	Aortic aneurysm	Number	2019	7493.12079	9010.078653	6204.265395	113.91%
Deaths	Kazakhstan	Male	Age-standardized	Aortic aneurysm	Rate	1990	2.547607178	3.639686539	2.095020821	
Deaths	Kazakhstan	Male	Age-standardized	Aortic aneurysm	Rate	2019	3.94345851	4.583551866	3.345483322	54.79%
Deaths	Kazakhstan	Male	All Ages	Aortic aneurysm	Number	1990	117.8576417	166.8572231	97.64454123	
Deaths	Kazakhstan	Male	All Ages	Aortic aneurysm	Number	2019	264.8116237	313.3540525	222.3143014	124.69%
DALYs	Kenya	Male	Age-standardized	Aortic aneurysm	Rate	1990	51.18784768	74.79221819	32.35689947	
DALYs	Kenya	Male	Age-standardized	Aortic aneurysm	Rate	2019	55.50021329	74.09799194	42.68311018	8.42%
DALYs	Kenya	Male	All Ages	Aortic aneurysm	Number	1990	2234.694929	3255.082236	1413.790306	
DALYs	Kenya	Male	All Ages	Aortic aneurysm	Number	2019	6288.859346	8218.311752	4819.626651	181.42%
Deaths	Kenya	Male	Age-standardized	Aortic aneurysm	Rate	1990	2.524256053	3.673549019	1.588137942	
Deaths	Kenya	Male	Age-standardized	Aortic aneurysm	Rate	2019	2.740286324	3.727880076	2.12336404	8.56%
Deaths	Kenya	Male	All Ages	Aortic aneurysm	Number	1990	89.9214305	131.3383735	56.79426328	
Deaths	Kenya	Male	All Ages	Aortic aneurysm	Number	2019	233.0574597	311.0290166	179.250947	159.18%
DALYs	Kiribati	Male	Age-standardized	Aortic aneurysm	Rate	1990	58.15597404	70.87119529	46.81894442	
DALYs	Kiribati	Male	Age-standardized	Aortic aneurysm	Rate	2019	49.2427087	61.61282735	39.11518933	-15.33%
DALYs	Kiribati	Male	All Ages	Aortic aneurysm	Number	1990	11.23291644	13.91646418	8.816253266	
DALYs	Kiribati	Male	All Ages	Aortic aneurysm	Number	2019	17.08348163	22.53593362	12.90503139	52.08%
Deaths	Kiribati	Male	Age-standardized	Aortic aneurysm	Rate	1990	2.463116837	2.945550827	2.021907052	
Deaths	Kiribati	Male	Age-standardized	Aortic aneurysm	Rate	2019	2.190941075	2.647938765	1.792735999	-11.05%
Deaths	Kiribati	Male	All Ages	Aortic aneurysm	Number	1990	0.359743811	0.442056426	0.287303044	
Deaths	Kiribati	Male	All Ages	Aortic aneurysm	Number	2019	0.531875606	0.670136739	0.413344043	47.85%
DALYs	Kuwait	Male	Age-standardized	Aortic aneurysm	Rate	1990	32.53057576	38.32092386	26.10093038	
DALYs	Kuwait	Male	Age-standardized	Aortic aneurysm	Rate	2019	36.28442289	45.68561417	28.14402099	11.54%
DALYs	Kuwait	Male	All Ages	Aortic aneurysm	Number	1990	154.4487816	177.4753007	130.2332799	
DALYs	Kuwait	Male	All Ages	Aortic aneurysm	Number	2019	574.9567012	719.3485888	456.6419501	272.26%
Deaths	Kuwait	Male	Age-standardized	Aortic aneurysm	Rate	1990	1.571126729	1.879119513	1.187668897	
Deaths	Kuwait	Male	Age-standardized	Aortic aneurysm	Rate	2019	1.932282786	2.44328871	1.479317835	22.99%
Deaths	Kuwait	Male	All Ages	Aortic aneurysm	Number	1990	5.018973024	5.829148853	4.149549768	
Deaths	Kuwait	Male	All Ages	Aortic aneurysm	Number	2019	24.04814573	30.12758449	18.79384489	379.14%
DALYs	Kyrgyzstan	Male	Age-standardized	Aortic aneurysm	Rate	1990	27.19981333	32.68162153	23.199274	
DALYs	Kyrgyzstan	Male	Age-standardized	Aortic aneurysm	Rate	2019	28.22954589	32.97798266	23.9756949	3.79%
DALYs	Kyrgyzstan	Male	All Ages	Aortic aneurysm	Number	1990	353.1249558	423.3230259	302.5513608	
DALYs	Kyrgyzstan	Male	All Ages	Aortic aneurysm	Number	2019	582.6030045	687.1707406	494.4175436	64.98%
Deaths	Kyrgyzstan	Male	Age-standardized	Aortic aneurysm	Rate	1990	1.210646264	1.439915302	1.028170285	
Deaths	Kyrgyzstan	Male	Age-standardized	Aortic aneurysm	Rate	2019	1.407899037	1.637184002	1.201584414	16.29%
Deaths	Kyrgyzstan	Male	All Ages	Aortic aneurysm	Number	1990	13.27956167	15.94805604	11.33275017	

Deaths	Kyrgyzstan	Male	All Ages	Aortic aneurysm	Number	2019	23.2261164	27.03728744	19.81043336	74.90%
DALYs	Lao People's Democratic Republic	Male	Age-standardized	Aortic aneurysm	Rate	1990	35.74167823	58.22548172	19.22649929	
DALYs	Lao People's Democratic Republic	Male	Age-standardized	Aortic aneurysm	Rate	2019	41.07284968	53.52345459	29.08166841	14.92%
DALYs	Lao People's Democratic Republic	Male	All Ages	Aortic aneurysm	Number	1990	351.5066569	584.6104649	190.4573688	
DALYs	Lao People's Democratic Republic	Male	All Ages	Aortic aneurysm	Number	2019	864.9301334	1156.067335	604.248594	146.06%
Deaths	Lao People's Democratic Republic	Male	Age-standardized	Aortic aneurysm	Rate	1990	1.814347901	2.878791368	0.946071421	
Deaths	Lao People's Democratic Republic	Male	Age-standardized	Aortic aneurysm	Rate	2019	2.171178634	2.769009913	1.589215	19.67%
Deaths	Lao People's Democratic Republic	Male	All Ages	Aortic aneurysm	Number	1990	14.04804029	22.9119729	7.527755785	
Deaths	Lao People's Democratic Republic	Male	All Ages	Aortic aneurysm	Number	2019	35.83107518	46.43400153	25.5737632	155.06%
DALYs	Latvia	Male	Age-standardized	Aortic aneurysm	Rate	1990	87.46109423	100.6791376	75.34657109	
DALYs	Latvia	Male	Age-standardized	Aortic aneurysm	Rate	2019	83.57985677	109.4135832	63.54416769	-4.44%
DALYs	Latvia	Male	All Ages	Aortic aneurysm	Number	1990	1129.540125	1310.892053	970.9822379	
DALYs	Latvia	Male	All Ages	Aortic aneurysm	Number	2019	1164.875528	1530.903691	884.7910409	3.13%
Deaths	Latvia	Male	Age-standardized	Aortic aneurysm	Rate	1990	4.036729617	4.650396737	3.475565929	
Deaths	Latvia	Male	Age-standardized	Aortic aneurysm	Rate	2019	3.94176371	5.044761748	3.052750796	-2.35%
Deaths	Latvia	Male	All Ages	Aortic aneurysm	Number	1990	48.42345113	55.76202845	41.75943177	
Deaths	Latvia	Male	All Ages	Aortic aneurysm	Number	2019	56.08351532	71.86227673	43.08290053	15.82%
DALYs	Lebanon	Male	Age-standardized	Aortic aneurysm	Rate	1990	47.71030352	66.16102282	32.54932089	
DALYs	Lebanon	Male	Age-standardized	Aortic aneurysm	Rate	2019	47.74903723	62.11157727	33.78023344	0.08%
DALYs	Lebanon	Male	All Ages	Aortic aneurysm	Number	1990	545.2295605	768.6147413	371.7071741	
DALYs	Lebanon	Male	All Ages	Aortic aneurysm	Number	2019	1144.726975	1488.364445	808.6299457	109.95%
Deaths	Lebanon	Male	Age-standardized	Aortic aneurysm	Rate	1990	2.292642447	3.146222382	1.577905522	
Deaths	Lebanon	Male	Age-standardized	Aortic aneurysm	Rate	2019	2.360080789	3.025059301	1.695076683	2.94%
Deaths	Lebanon	Male	All Ages	Aortic aneurysm	Number	1990	22.10078137	30.57787815	15.07536786	
Deaths	Lebanon	Male	All Ages	Aortic aneurysm	Number	2019	54.91654075	70.21046816	39.3174557	148.48%
DALYs	Lesotho	Male	Age-standardized	Aortic aneurysm	Rate	1990	67.24053955	105.3805395	37.40067585	
DALYs	Lesotho	Male	Age-standardized	Aortic aneurysm	Rate	2019	83.43891149	115.6766884	51.69094879	24.09%
DALYs	Lesotho	Male	All Ages	Aortic aneurysm	Number	1990	274.9879708	433.1515544	155.1549323	
DALYs	Lesotho	Male	All Ages	Aortic aneurysm	Number	2019	440.9898474	635.4729912	253.1417434	60.37%
Deaths	Lesotho	Male	Age-standardized	Aortic aneurysm	Rate	1990	3.524936238	5.524907517	1.943379313	
Deaths	Lesotho	Male	Age-standardized	Aortic aneurysm	Rate	2019	4.197526316	5.741610819	2.747212441	19.08%
Deaths	Lesotho	Male	All Ages	Aortic aneurysm	Number	1990	11.39805895	17.98910553	6.325475948	
Deaths	Lesotho	Male	All Ages	Aortic aneurysm	Number	2019	16.64302869	23.18157567	10.22520625	46.02%
DALYs	Liberia	Male	Age-standardized	Aortic aneurysm	Rate	1990	42.63057202	64.87285229	22.36822407	
DALYs	Liberia	Male	Age-standardized	Aortic aneurysm	Rate	2019	39.16838373	58.53912585	24.42738475	-8.12%
DALYs	Liberia	Male	All Ages	Aortic aneurysm	Number	1990	255.7669997	390.5865942	134.1618751	
DALYs	Liberia	Male	All Ages	Aortic aneurysm	Number	2019	423.8927338	659.7490599	255.3581947	65.73%
Deaths	Liberia	Male	Age-standardized	Aortic aneurysm	Rate	1990	2.128977516	3.218763684	1.121219334	
Deaths	Liberia	Male	Age-standardized	Aortic aneurysm	Rate	2019	2.031837579	2.970541277	1.304512219	-4.56%
Deaths	Liberia	Male	All Ages	Aortic aneurysm	Number	1990	11.4150481	17.15089607	5.988940036	
Deaths	Liberia	Male	All Ages	Aortic aneurysm	Number	2019	17.37872733	26.05786222	10.98085594	52.24%
DALYs	Libya	Male	Age-standardized	Aortic aneurysm	Rate	1990	22.41537344	34.78358467	14.45318904	
DALYs	Libya	Male	Age-standardized	Aortic aneurysm	Rate	2019	27.5285383	45.55737754	15.78569017	22.81%
DALYs	Libya	Male	All Ages	Aortic aneurysm	Number	1990	255.0330636	401.8110037	166.6709233	
DALYs	Libya	Male	All Ages	Aortic aneurysm	Number	2019	774.2533465	1286.1366	440.1049331	203.59%
Deaths	Libya	Male	Age-standardized	Aortic aneurysm	Rate	1990	1.080198096	1.65850016	0.68884742	
Deaths	Libya	Male	Age-standardized	Aortic aneurysm	Rate	2019	1.321326494	2.204418617	0.772598107	22.32%
Deaths	Libya	Male	All Ages	Aortic aneurysm	Number	1990	9.806418269	15.15129083	6.308911149	
Deaths	Libya	Male	All Ages	Aortic aneurysm	Number	2019	31.14224902	51.35735601	18.07997677	217.57%
DALYs	Lithuania	Male	Age-standardized	Aortic aneurysm	Rate	1990	78.39873137	94.26681778	66.92146967	
DALYs	Lithuania	Male	Age-standardized	Aortic aneurysm	Rate	2019	111.2443116	136.5523798	88.01755837	41.90%
DALYs	Lithuania	Male	All Ages	Aortic aneurysm	Number	1990	1412.43051	1708.35839	1197.708741	
DALYs	Lithuania	Male	All Ages	Aortic aneurysm	Number	2019	2231.863963	2740.10043	1764.32837	58.02%
Deaths	Lithuania	Male	Age-standardized	Aortic aneurysm	Rate	1990	3.219417697	3.818908379	2.766922625	
Deaths	Lithuania	Male	Age-standardized	Aortic aneurysm	Rate	2019	4.775567641	5.774715338	3.847086157	48.34%
Deaths	Lithuania	Male	All Ages	Aortic aneurysm	Number	1990	55.2448662	66.0862221	47.40435887	
Deaths	Lithuania	Male	All Ages	Aortic aneurysm	Number	2019	99.36417215	120.6870029	79.6022298	79.86%
DALYs	Luxembourg	Male	Age-standardized	Aortic aneurysm	Rate	1990	144.2849097	159.5386458	130.9557555	
DALYs	Luxembourg	Male	Age-standardized	Aortic aneurysm	Rate	2019	74.17297463	87.65438897	63.02658027	-48.59%
DALYs	Luxembourg	Male	All Ages	Aortic aneurysm	Number	1990	320.9727988	355.7817437	290.3002834	
DALYs	Luxembourg	Male	All Ages	Aortic aneurysm	Number	2019	334.4044977	395.8126226	284.0794212	4.18%
Deaths	Luxembourg	Male	Age-standardized	Aortic aneurysm	Rate	1990	7.586520406	8.386286477	6.83160921	
Deaths	Luxembourg	Male	Age-standardized	Aortic aneurysm	Rate	2019	4.243308615	5.014281953	3.596250065	-44.07%
Deaths	Luxembourg	Male	All Ages	Aortic aneurysm	Number	1990	15.8350488	17.69008711	14.26140406	
Deaths	Luxembourg	Male	All Ages	Aortic aneurysm	Number	2019	19.08197337	22.59463432	16.15386085	20.50%
DALYs	Madagascar	Male	Age-standardized	Aortic aneurysm	Rate	1990	115.6850333	174.2883876	68.72322667	
DALYs	Madagascar	Male	Age-standardized	Aortic aneurysm	Rate	2019	86.94128902	131.6756485	53.04823368	-24.85%
DALYs	Madagascar	Male	All Ages	Aortic aneurysm	Number	1990	3498.980602	5396.477768	2072.415043	
DALYs	Madagascar	Male	All Ages	Aortic aneurysm	Number	2019	5607.153499	8467.324874	3477.772132	60.25%
Deaths	Madagascar	Male	Age-standardized	Aortic aneurysm	Rate	1990	4.937566619	7.328428226	2.881505299	
Deaths	Madagascar	Male	Age-standardized	Aortic aneurysm	Rate	2019	3.800442142	5.654067822	2.311732821	-23.03%
Deaths	Madagascar	Male	All Ages	Aortic aneurysm	Number	1990	123.581645	184.6434747	73.13667947	
Deaths	Madagascar	Male	All Ages	Aortic aneurysm	Number	2019	181.616524	275.2918007	111.1614797	46.96%
DALYs	Malawi	Male	Age-standardized	Aortic aneurysm	Rate	1990	74.25230601	118.0895632	35.20892181	
DALYs	Malawi	Male	Age-standardized	Aortic aneurysm	Rate	2019	68.48595608	98.62449504	39.17223643	-7.77%
DALYs	Malawi	Male	All Ages	Aortic aneurysm	Number	1990	1432.429781	2297.825144	684.2544257	
DALYs	Malawi	Male	All Ages	Aortic aneurysm	Number	2019	2475.729194	3652.253961	1389.264948	72.83%
Deaths	Malawi	Male	Age-standardized	Aortic aneurysm	Rate	1990	3.559145835	5.591017647	1.650911064	
Deaths	Malawi	Male	Age-standardized	Aortic aneurysm	Rate	2019	3.287388916	4.672276596	1.893946065	-7.64%
Deaths	Malawi	Male	All Ages	Aortic aneurysm	Number	1990	54.56037294	86.81403173	25.79720602	
Deaths	Malawi	Male	All Ages	Aortic aneurysm	Number	2019	90.32206901	129.7224474	51.73273713	65.55%
DALYs	Malaysia	Male	Age-standardized	Aortic aneurysm	Rate	1990	114.6571925	142.039672	92.76935343	
DALYs	Malaysia	Male	Age-standardized	Aortic aneurysm	Rate	2019	114.7767957	146.8169423	88.97545248	0.10%
DALYs	Malaysia	Male	All Ages	Aortic aneurysm	Number	1990	4923.963125	6071.195124	4030.624403	
DALYs	Malaysia	Male	All Ages	Aortic aneurysm	Number	2019	14534.75749	18752.04399	11138.02951	195.18%
Deaths	Malaysia	Male	Age-standardized	Aortic aneurysm	Rate	1990	6.060505432	7.47697377	4.874060494	
Deaths	Malaysia	Male	Age-standardized	Aortic aneurysm	Rate	2019	6.609869793	8.337556554	5.134310845	9.06%
Deaths	Malaysia	Male	All Ages	Aortic aneurysm	Number	1990	227.7588385	281.0214866	184.6060915	
Deaths	Malaysia	Male	All Ages	Aortic aneurysm	Number	2019	729.7561664	922.0897219	567.2982885	220.41%
DALYs	Maldives	Male	Age-standardized	Aortic aneurysm	Rate	1990	60.08030336	87.91113535	37.86199183	
DALYs	Maldives	Male	Age-standardized	Aortic aneurysm	Rate	2019	46.3590981	57.00226635	37.42435009	-22.84%
DALYs	Maldives	Male	All Ages	Aortic aneurysm	Number	1990	28.43726932	41.99885555	17.08802579	
DALYs	Maldives	Male	All Ages	Aortic aneurysm	Number	2019	74.05598927	90.15632972	60.43194019	160.42%
Deaths	Maldives	Male	Age-standardized	Aortic aneurysm	Rate	1990	3.493358049	5.086458501	2.289946628	
Deaths	Maldives	Male	Age-standardized	Aortic aneurysm	Rate	2019	3.03287328	3.738770169	2.445874188	-13.18%
Deaths	Maldives	Male	All Ages	Aortic aneurysm	Number	1990	1.239955663	1.82285063	0.781554485	
Deaths	Maldives	Male	All Ages	Aortic aneurysm	Number	2019	3.964891804	4.857797011	3.202085763	219.76%
DALYs	Mali	Male	Age-standardized	Aortic aneurysm	Rate	1990	36.05358815	57.40354877	18.06338429	
DALYs	Mali	Male	Age-standardized	Aortic aneurysm	Rate	2019	36.2678948	53.95575118	22.03093232	0.59%
DALYs	Mali	Male	All Ages	Aortic aneurysm	Number	1990	727.7942845	1172.953554	361.0381412	
DALYs	Mali	Male	All Ages	Aortic aneurysm	Number	2019	1588.883223	2405.516901	943.9189221	118.31%

Deaths	Mali	Male	Age-standardized	Aortic aneurysm	Rate	1990	1.868848941	2.987992819	0.948801728	
Deaths	Mali	Male	Age-standardized	Aortic aneurysm	Rate	2019	1.971045554	2.924423539	1.221578733	5.47%
Deaths	Mali	Male	All Ages	Aortic aneurysm	Number	1990	31.13154391	49.85724758	15.65840571	
Deaths	Mali	Male	All Ages	Aortic aneurysm	Number	2019	71.22934283	105.5285856	43.5047577	128.80%
DALYs	Malta	Male	Age-standardized	Aortic aneurysm	Rate	1990	83.58185386	92.890742	74.44783904	
DALYs	Malta	Male	Age-standardized	Aortic aneurysm	Rate	2019	58.61947412	69.19418354	49.30959118	-29.87%
DALYs	Malta	Male	All Ages	Aortic aneurysm	Number	1990	156.8295939	174.8605923	139.0378377	
DALYs	Malta	Male	All Ages	Aortic aneurysm	Number	2019	241.2937546	284.8817028	200.7689479	53.86%
Deaths	Malta	Male	Age-standardized	Aortic aneurysm	Rate	1990	4.560459472	5.100253156	4.073282729	
Deaths	Malta	Male	Age-standardized	Aortic aneurysm	Rate	2019	3.2308089	3.800455707	2.717354686	-29.16%
Deaths	Malta	Male	All Ages	Aortic aneurysm	Number	1990	7.960893482	8.924293216	7.072316997	
Deaths	Malta	Male	All Ages	Aortic aneurysm	Number	2019	13.71677396	16.20401606	11.45266385	72.30%
DALYs	Marshall Islands	Male	Age-standardized	Aortic aneurysm	Rate	1990	136.0669858	226.2789439	77.97341491	
DALYs	Marshall Islands	Male	Age-standardized	Aortic aneurysm	Rate	2019	116.5800059	171.7857663	73.16054588	-14.32%
DALYs	Marshall Islands	Male	All Ages	Aortic aneurysm	Number	1990	12.48716297	20.96717448	6.753692897	
DALYs	Marshall Islands	Male	All Ages	Aortic aneurysm	Number	2019	23.56405078	35.275144	14.17250494	88.71%
Deaths	Marshall Islands	Male	Age-standardized	Aortic aneurysm	Rate	1990	6.167982597	10.09435615	3.710861999	
Deaths	Marshall Islands	Male	Age-standardized	Aortic aneurysm	Rate	2019	5.082653333	7.347190903	3.383793823	-17.60%
Deaths	Marshall Islands	Male	All Ages	Aortic aneurysm	Number	1990	0.437342317	0.724413028	0.249052967	
Deaths	Marshall Islands	Male	All Ages	Aortic aneurysm	Number	2019	0.775623235	1.145469972	0.484522886	77.35%
DALYs	Mauritania	Male	Age-standardized	Aortic aneurysm	Rate	1990	53.22637161	72.78115017	37.01800409	
DALYs	Mauritania	Male	Age-standardized	Aortic aneurysm	Rate	2019	36.94547385	53.47045731	25.76940999	-30.59%
DALYs	Mauritania	Male	All Ages	Aortic aneurysm	Number	1990	261.6804093	358.4070619	182.5034474	
DALYs	Mauritania	Male	All Ages	Aortic aneurysm	Number	2019	387.1354881	574.7569874	265.2562812	47.94%
Deaths	Mauritania	Male	Age-standardized	Aortic aneurysm	Rate	1990	2.576990699	3.479506266	1.796364269	
Deaths	Mauritania	Male	Age-standardized	Aortic aneurysm	Rate	2019	1.985831406	2.72174246	1.485482549	-22.94%
Deaths	Mauritania	Male	All Ages	Aortic aneurysm	Number	1990	10.91117012	14.88073928	7.600886897	
Deaths	Mauritania	Male	All Ages	Aortic aneurysm	Number	2019	18.39526607	25.60341752	13.40545193	68.59%
DALYs	Mauritius	Male	Age-standardized	Aortic aneurysm	Rate	1990	35.9653305	39.6800464	32.51196097	
DALYs	Mauritius	Male	Age-standardized	Aortic aneurysm	Rate	2019	33.65093557	41.49480093	26.87149503	-6.44%
DALYs	Mauritius	Male	All Ages	Aortic aneurysm	Number	1990	129.0828982	142.2811154	116.0129366	
DALYs	Mauritius	Male	All Ages	Aortic aneurysm	Number	2019	255.8674582	319.5038528	203.3790296	98.22%
Deaths	Mauritius	Male	Age-standardized	Aortic aneurysm	Rate	1990	1.730636232	1.923003172	1.556618313	
Deaths	Mauritius	Male	Age-standardized	Aortic aneurysm	Rate	2019	1.692655244	2.050962503	1.349348921	-2.19%
Deaths	Mauritius	Male	All Ages	Aortic aneurysm	Number	1990	4.836305575	5.36378169	4.353593357	
Deaths	Mauritius	Male	All Ages	Aortic aneurysm	Number	2019	11.55730952	14.18657784	9.208720583	138.97%
DALYs	Mexico	Male	Age-standardized	Aortic aneurysm	Rate	1990	32.01108902	33.22981165	30.79195406	
DALYs	Mexico	Male	Age-standardized	Aortic aneurysm	Rate	2019	31.33431814	38.72668248	24.80700832	-2.11%
DALYs	Mexico	Male	All Ages	Aortic aneurysm	Number	1990	6963.375706	7214.415534	6737.837113	
DALYs	Mexico	Male	All Ages	Aortic aneurysm	Number	2019	17071.34727	21143.4348	13459.98101	145.16%
Deaths	Mexico	Male	Age-standardized	Aortic aneurysm	Rate	1990	1.584453151	1.655046723	1.493290296	
Deaths	Mexico	Male	Age-standardized	Aortic aneurysm	Rate	2019	1.605937366	1.958495145	1.289943209	1.36%
Deaths	Mexico	Male	All Ages	Aortic aneurysm	Number	1990	294.3058007	306.0662587	280.5139403	
Deaths	Mexico	Male	All Ages	Aortic aneurysm	Number	2019	812.9617793	996.9333266	648.6859914	176.23%
DALYs	Micronesia (Federated States of)	Male	Age-standardized	Aortic aneurysm	Rate	1990	138.0917803	228.8902736	77.17128131	
DALYs	Micronesia (Federated States of)	Male	Age-standardized	Aortic aneurysm	Rate	2019	129.4574772	190.868263	68.05756063	-6.25%
DALYs	Micronesia (Federated States of)	Male	All Ages	Aortic aneurysm	Number	1990	36.76299389	62.41788022	18.97863712	
DALYs	Micronesia (Federated States of)	Male	All Ages	Aortic aneurysm	Number	2019	48.09138523	73.3871108	23.37871679	30.81%
Deaths	Micronesia (Federated States of)	Male	Age-standardized	Aortic aneurysm	Rate	1990	5.935386483	9.61754114	3.650094125	
Deaths	Micronesia (Federated States of)	Male	Age-standardized	Aortic aneurysm	Rate	2019	5.66399703	8.029617845	3.289094074	-4.57%
Deaths	Micronesia (Federated States of)	Male	All Ages	Aortic aneurysm	Number	1990	1.236022851	2.044013189	0.69605377	
Deaths	Micronesia (Federated States of)	Male	All Ages	Aortic aneurysm	Number	2019	1.599501602	2.365072975	0.82946354	29.41%
DALYs	Monaco	Male	Age-standardized	Aortic aneurysm	Rate	1990	110.9573956	136.8142266	86.94523484	
DALYs	Monaco	Male	Age-standardized	Aortic aneurysm	Rate	2019	87.13921824	105.6964645	65.23075409	-21.47%
DALYs	Monaco	Male	All Ages	Aortic aneurysm	Number	1990	31.7278316	38.96530393	24.91053584	
DALYs	Monaco	Male	All Ages	Aortic aneurysm	Number	2019	35.86868134	43.20450962	27.4618473	13.05%
Deaths	Monaco	Male	Age-standardized	Aortic aneurysm	Rate	1990	5.895591913	7.162058925	4.668296911	
Deaths	Monaco	Male	Age-standardized	Aortic aneurysm	Rate	2019	4.763636574	5.664656305	3.713860244	-19.20%
Deaths	Monaco	Male	All Ages	Aortic aneurysm	Number	1990	1.805933305	2.18742237	1.429853253	
Deaths	Monaco	Male	All Ages	Aortic aneurysm	Number	2019	2.206886619	2.623786027	1.734311065	22.20%
DALYs	Mongolia	Male	Age-standardized	Aortic aneurysm	Rate	1990	29.52201657	37.70224312	22.11073283	
DALYs	Mongolia	Male	Age-standardized	Aortic aneurysm	Rate	2019	33.51573327	42.97498209	26.50573741	13.53%
DALYs	Mongolia	Male	All Ages	Aortic aneurysm	Number	1990	145.0004713	187.9127169	106.2868107	
DALYs	Mongolia	Male	All Ages	Aortic aneurysm	Number	2019	363.6639941	481.3950494	278.0403351	150.80%
Deaths	Mongolia	Male	Age-standardized	Aortic aneurysm	Rate	1990	1.485519992	1.879730292	1.141406162	
Deaths	Mongolia	Male	Age-standardized	Aortic aneurysm	Rate	2019	1.675965597	2.063989175	1.366698073	12.82%
Deaths	Mongolia	Male	All Ages	Aortic aneurysm	Number	1990	5.768577543	7.37393299	4.337528507	
Deaths	Mongolia	Male	All Ages	Aortic aneurysm	Number	2019	13.02927543	16.91245698	10.11281792	125.87%
DALYs	Montenegro	Male	Age-standardized	Aortic aneurysm	Rate	1990	246.9800644	298.4212684	211.5754396	
DALYs	Montenegro	Male	Age-standardized	Aortic aneurysm	Rate	2019	278.9204925	355.2130969	216.6789325	12.93%
DALYs	Montenegro	Male	All Ages	Aortic aneurysm	Number	1990	721.3862615	869.2866797	616.7175264	
DALYs	Montenegro	Male	All Ages	Aortic aneurysm	Number	2019	1204.66744	1535.455151	933.662054	66.99%
Deaths	Montenegro	Male	Age-standardized	Aortic aneurysm	Rate	1990	10.38863223	12.63064222	8.859593469	
Deaths	Montenegro	Male	Age-standardized	Aortic aneurysm	Rate	2019	13.22446763	16.73071936	10.36818536	27.30%
Deaths	Montenegro	Male	All Ages	Aortic aneurysm	Number	1990	27.22066885	33.26509649	23.30070306	
Deaths	Montenegro	Male	All Ages	Aortic aneurysm	Number	2019	55.1799353	70.23775337	43.01120822	102.71%
DALYs	Morocco	Male	Age-standardized	Aortic aneurysm	Rate	1990	30.84619547	46.00285715	17.13293598	
DALYs	Morocco	Male	Age-standardized	Aortic aneurysm	Rate	2019	36.4868132	49.25500775	24.26412386	18.29%
DALYs	Morocco	Male	All Ages	Aortic aneurysm	Number	1990	2172.699227	3298.063932	1215.539642	
DALYs	Morocco	Male	All Ages	Aortic aneurysm	Number	2019	5503.581578	7503.66539	3612.868589	153.31%
Deaths	Morocco	Male	Age-standardized	Aortic aneurysm	Rate	1990	1.517646565	2.240325656	0.841262631	
Deaths	Morocco	Male	Age-standardized	Aortic aneurysm	Rate	2019	1.918690224	2.562789216	1.293427576	26.43%
Deaths	Morocco	Male	All Ages	Aortic aneurysm	Number	1990	87.85713834	130.2916749	48.89812792	
Deaths	Morocco	Male	All Ages	Aortic aneurysm	Number	2019	249.4948294	335.0569234	167.556957	183.98%
DALYs	Mozambique	Male	Age-standardized	Aortic aneurysm	Rate	1990	77.07931199	129.0296967	32.38408623	
DALYs	Mozambique	Male	Age-standardized	Aortic aneurysm	Rate	2019	94.30728456	148.4074143	53.65552609	22.35%
DALYs	Mozambique	Male	All Ages	Aortic aneurysm	Number	1990	2235.604766	3744.389461	934.1789173	
DALYs	Mozambique	Male	All Ages	Aortic aneurysm	Number	2019	5129.721449	8391.649057	2876.588967	129.46%
Deaths	Mozambique	Male	Age-standardized	Aortic aneurysm	Rate	1990	3.720135605	6.189302274	1.527015311	
Deaths	Mozambique	Male	Age-standardized	Aortic aneurysm	Rate	2019	4.334212999	6.684989571	2.499551991	16.51%
Deaths	Mozambique	Male	All Ages	Aortic aneurysm	Number	1990	88.53815618	148.8430434	37.09809748	
Deaths	Mozambique	Male	All Ages	Aortic aneurysm	Number	2019	181.8566207	286.0875355	103.6338858	105.40%
DALYs	Myanmar	Male	Age-standardized	Aortic aneurysm	Rate	1990	38.59858847	61.1147577	21.82879414	
DALYs	Myanmar	Male	Age-standardized	Aortic aneurysm	Rate	2019	41.89683574	53.6309679	29.78296591	8.54%
DALYs	Myanmar	Male	All Ages	Aortic aneurysm	Number	1990	4372.947125	7096.784498	2396.65753	
DALYs	Myanmar	Male	All Ages	Aortic aneurysm	Number	2019	8326.16053	10834.6628	5818.666759	90.40%
Deaths	Myanmar	Male	Age-standardized	Aortic aneurysm	Rate	1990	1.863397788	2.881856848	1.077535715	
Deaths	Myanmar	Male	Age-standardized	Aortic aneurysm	Rate	2019	2.167385049	2.723033536	1.565218127	16.31%
Deaths	Myanmar	Male	All Ages	Aortic aneurysm	Number	1990	166.8229643	262.6161362	95.16375931	
Deaths	Myanmar	Male	All Ages	Aortic aneurysm	Number	2019	357.9338461	454.0708659	256.2947633	114.56%
DALYs	Namibia	Male	Age-standardized	Aortic aneurysm	Rate	1990	86.51629668	113.261596	65.73282607	

DALYs	Namibia	Male	Age-standardized	Aortic aneurysm	Rate	2019	85.06974486	116.0648542	60.25878293	-1.67%
DALYs	Namibia	Male	All Ages	Aortic aneurysm	Number	1990	282.1706865	370.9932831	211.9085681	
DALYs	Namibia	Male	All Ages	Aortic aneurysm	Number	2019	496.2427683	689.1371158	352.901398	75.87%
Deaths	Namibia	Male	Age-standardized	Aortic aneurysm	Rate	1990	4.531113924	5.927935696	3.497328608	
Deaths	Namibia	Male	Age-standardized	Aortic aneurysm	Rate	2019	4.668955693	6.267179725	3.289769505	3.04%
Deaths	Namibia	Male	All Ages	Aortic aneurysm	Number	1990	12.29950392	16.15399906	9.347671766	
Deaths	Namibia	Male	All Ages	Aortic aneurysm	Number	2019	22.26715167	30.04554358	15.73862338	81.04%
DALYs	Nauru	Male	Age-standardized	Aortic aneurysm	Rate	1990	164.1001391	245.5588473	89.53597795	
DALYs	Nauru	Male	Age-standardized	Aortic aneurysm	Rate	2019	150.9877114	217.6060313	85.17444362	-7.99%
DALYs	Nauru	Male	All Ages	Aortic aneurysm	Number	1990	4.138930327	6.424389376	1.998650461	
DALYs	Nauru	Male	All Ages	Aortic aneurysm	Number	2019	3.977010299	5.963871681	2.005554945	-3.91%
Deaths	Nauru	Male	Age-standardized	Aortic aneurysm	Rate	1990	7.037629148	10.22368381	4.1486623	
Deaths	Nauru	Male	Age-standardized	Aortic aneurysm	Rate	2019	6.449893341	9.016618322	3.942831873	-8.35%
Deaths	Nauru	Male	All Ages	Aortic aneurysm	Number	1990	0.123231696	0.186143881	0.064638271	
Deaths	Nauru	Male	All Ages	Aortic aneurysm	Number	2019	0.109742789	0.162123851	0.058162656	-10.95%
DALYs	Nepal	Male	Age-standardized	Aortic aneurysm	Rate	1990	32.66111261	64.15223084	12.612786	
DALYs	Nepal	Male	Age-standardized	Aortic aneurysm	Rate	2019	44.74259163	67.57274715	25.95252872	36.99%
DALYs	Nepal	Male	All Ages	Aortic aneurysm	Number	1990	1582.054684	3122.216352	610.6154613	
DALYs	Nepal	Male	All Ages	Aortic aneurysm	Number	2019	4661.185079	7127.026417	2659.58149	194.63%
Deaths	Nepal	Male	Age-standardized	Aortic aneurysm	Rate	1990	1.603252948	3.13688152	0.610506801	
Deaths	Nepal	Male	Age-standardized	Aortic aneurysm	Rate	2019	2.368192284	3.520996027	1.373086968	47.71%
Deaths	Nepal	Male	All Ages	Aortic aneurysm	Number	1990	62.98524088	122.9384772	24.13146818	
Deaths	Nepal	Male	All Ages	Aortic aneurysm	Number	2019	216.7317816	325.7881758	125.5245578	244.10%
DALYs	Netherlands	Male	Age-standardized	Aortic aneurysm	Rate	1990	235.193808	249.8874013	221.3097451	
DALYs	Netherlands	Male	Age-standardized	Aortic aneurysm	Rate	2019	101.8343926	111.9033065	91.08124965	-56.70%
DALYs	Netherlands	Male	All Ages	Aortic aneurysm	Number	1990	19912.81112	21164.14848	18714.48791	
DALYs	Netherlands	Male	All Ages	Aortic aneurysm	Number	2019	16100.77511	17805.81146	14338.24118	-19.14%
Deaths	Netherlands	Male	Age-standardized	Aortic aneurysm	Rate	1990	13.26646057	14.13747365	12.39867015	
Deaths	Netherlands	Male	Age-standardized	Aortic aneurysm	Rate	2019	6.462898627	7.165007496	5.654544948	-51.28%
Deaths	Netherlands	Male	All Ages	Aortic aneurysm	Number	1990	1070.127371	1141.875653	1002.550614	
Deaths	Netherlands	Male	All Ages	Aortic aneurysm	Number	2019	1019.61296	1132.231706	890.9827181	-4.72%
DALYs	New Zealand	Male	Age-standardized	Aortic aneurysm	Rate	1990	260.5954624	276.3027799	244.7048736	
DALYs	New Zealand	Male	Age-standardized	Aortic aneurysm	Rate	2019	112.306351	123.9718584	100.7967081	-56.90%
DALYs	New Zealand	Male	All Ages	Aortic aneurysm	Number	1990	4558.640032	4843.076194	4287.649464	
DALYs	New Zealand	Male	All Ages	Aortic aneurysm	Number	2019	4057.500092	4498.020509	3610.425879	-10.99%
Deaths	New Zealand	Male	Age-standardized	Aortic aneurysm	Rate	1990	14.83521915	15.84226859	13.79513158	
Deaths	New Zealand	Male	Age-standardized	Aortic aneurysm	Rate	2019	6.652928261	7.438431975	5.88822094	-55.15%
Deaths	New Zealand	Male	All Ages	Aortic aneurysm	Number	1990	243.8741543	259.780433	227.6530377	
Deaths	New Zealand	Male	All Ages	Aortic aneurysm	Number	2019	249.0634679	279.4937934	220.0485898	2.13%
DALYs	Nicaragua	Male	Age-standardized	Aortic aneurysm	Rate	1990	26.64322989	31.98544469	21.64476543	
DALYs	Nicaragua	Male	Age-standardized	Aortic aneurysm	Rate	2019	30.86269185	38.50403099	24.09680782	15.84%
DALYs	Nicaragua	Male	All Ages	Aortic aneurysm	Number	1990	202.0341648	241.2742682	166.1919346	
DALYs	Nicaragua	Male	All Ages	Aortic aneurysm	Number	2019	602.311846	758.5299115	467.1928901	198.12%
Deaths	Nicaragua	Male	Age-standardized	Aortic aneurysm	Rate	1990	1.331626218	1.6105153	1.063825999	
Deaths	Nicaragua	Male	Age-standardized	Aortic aneurysm	Rate	2019	1.728871702	2.106990909	1.375282723	29.83%
Deaths	Nicaragua	Male	All Ages	Aortic aneurysm	Number	1990	8.138035068	9.777298987	6.540005566	
Deaths	Nicaragua	Male	All Ages	Aortic aneurysm	Number	2019	28.15146559	34.89881827	22.10680791	245.92%
DALYs	Niger	Male	Age-standardized	Aortic aneurysm	Rate	1990	38.99837675	67.90074395	16.67012329	
DALYs	Niger	Male	Age-standardized	Aortic aneurysm	Rate	2019	34.20031888	57.34168712	17.24269174	-12.30%
DALYs	Niger	Male	All Ages	Aortic aneurysm	Number	1990	594.6569074	1056.703174	255.3142973	
DALYs	Niger	Male	All Ages	Aortic aneurysm	Number	2019	1347.207158	2309.686825	671.0500244	126.55%
Deaths	Niger	Male	Age-standardized	Aortic aneurysm	Rate	1990	1.880989515	3.254009616	0.798403297	
Deaths	Niger	Male	Age-standardized	Aortic aneurysm	Rate	2019	1.726866871	2.832426408	0.867707103	-8.19%
Deaths	Niger	Male	All Ages	Aortic aneurysm	Number	1990	22.86915376	40.14264395	9.724781239	
Deaths	Niger	Male	All Ages	Aortic aneurysm	Number	2019	53.86650492	90.69972096	27.14836475	135.54%
DALYs	Nigeria	Male	Age-standardized	Aortic aneurysm	Rate	1990	50.53923867	81.35992957	27.29720382	
DALYs	Nigeria	Male	Age-standardized	Aortic aneurysm	Rate	2019	44.66708543	64.07951916	31.32029688	-11.62%
DALYs	Nigeria	Male	All Ages	Aortic aneurysm	Number	1990	11716.14588	19012.28586	6134.25029	
DALYs	Nigeria	Male	All Ages	Aortic aneurysm	Number	2019	18736.59855	27540.77574	12937.84953	59.92%
Deaths	Nigeria	Male	Age-standardized	Aortic aneurysm	Rate	1990	2.481701851	3.924571244	1.385735509	
Deaths	Nigeria	Male	Age-standardized	Aortic aneurysm	Rate	2019	2.312388091	3.182383782	1.6660002	-6.82%
Deaths	Nigeria	Male	All Ages	Aortic aneurysm	Number	1990	469.8931471	754.3336742	253.3690746	
Deaths	Nigeria	Male	All Ages	Aortic aneurysm	Number	2019	803.4329351	1134.771478	570.0365041	70.98%
DALYs	Niue	Male	Age-standardized	Aortic aneurysm	Rate	1990	123.7617759	168.5112391	82.65129267	
DALYs	Niue	Male	Age-standardized	Aortic aneurysm	Rate	2019	101.6849702	135.6182688	74.1099918	-17.84%
DALYs	Niue	Male	All Ages	Aortic aneurysm	Number	1990	1.153964508	1.574313375	0.766121503	
DALYs	Niue	Male	All Ages	Aortic aneurysm	Number	2019	0.979924804	1.307954629	0.707074367	-15.08%
Deaths	Niue	Male	Age-standardized	Aortic aneurysm	Rate	1990	5.656820291	7.465049534	3.924643731	
Deaths	Niue	Male	Age-standardized	Aortic aneurysm	Rate	2019	4.813161625	6.322940183	3.549207751	-14.91%
Deaths	Niue	Male	All Ages	Aortic aneurysm	Number	1990	0.050196427	0.066616946	0.034691724	
Deaths	Niue	Male	All Ages	Aortic aneurysm	Number	2019	0.043669879	0.057278539	0.032269402	-13.00%
DALYs	North Macedonia	Male	Age-standardized	Aortic aneurysm	Rate	1990	46.62524964	53.62841409	40.80421182	
DALYs	North Macedonia	Male	Age-standardized	Aortic aneurysm	Rate	2019	75.8028239	97.26193739	57.59902437	62.58%
DALYs	North Macedonia	Male	All Ages	Aortic aneurysm	Number	1990	422.4480619	485.7941517	368.3165484	
DALYs	North Macedonia	Male	All Ages	Aortic aneurysm	Number	2019	1144.308518	1479.708987	853.0565395	170.88%
Deaths	North Macedonia	Male	Age-standardized	Aortic aneurysm	Rate	1990	2.22975835	2.593077298	1.947478437	
Deaths	North Macedonia	Male	Age-standardized	Aortic aneurysm	Rate	2019	3.744948567	4.723043718	2.922319474	67.95%
Deaths	North Macedonia	Male	All Ages	Aortic aneurysm	Number	1990	17.7011869	20.39430061	15.43114214	
Deaths	North Macedonia	Male	All Ages	Aortic aneurysm	Number	2019	50.98804599	65.10148372	38.93103935	188.05%
DALYs	Northern Mariana Islands	Male	Age-standardized	Aortic aneurysm	Rate	1990	129.7065251	164.7654717	103.2567129	
DALYs	Northern Mariana Islands	Male	Age-standardized	Aortic aneurysm	Rate	2019	59.90834611	69.81900862	50.72596571	-53.81%
DALYs	Northern Mariana Islands	Male	All Ages	Aortic aneurysm	Number	1990	18.29373374	24.46896436	13.43599856	
DALYs	Northern Mariana Islands	Male	All Ages	Aortic aneurysm	Number	2019	15.96404013	19.32411785	13.03957944	-12.73%
Deaths	Northern Mariana Islands	Male	Age-standardized	Aortic aneurysm	Rate	1990	5.886206514	7.322969514	4.783947292	
Deaths	Northern Mariana Islands	Male	Age-standardized	Aortic aneurysm	Rate	2019	2.9130405	3.343120218	2.488909295	-50.51%
Deaths	Northern Mariana Islands	Male	All Ages	Aortic aneurysm	Number	1990	0.538866517	0.698702071	0.417320619	
Deaths	Northern Mariana Islands	Male	All Ages	Aortic aneurysm	Number	2019	0.623888288	0.731470914	0.523614698	15.78%
DALYs	Norway	Male	Age-standardized	Aortic aneurysm	Rate	1990	226.6119388	235.4352853	217.2992578	
DALYs	Norway	Male	Age-standardized	Aortic aneurysm	Rate	2019	119.4989221	132.5417566	109.9503702	-47.27%
DALYs	Norway	Male	All Ages	Aortic aneurysm	Number	1990	6786.553182	7065.715784	6496.96048	
DALYs	Norway	Male	All Ages	Aortic aneurysm	Number	2019	5348.225628	5944.318669	4911.328232	-21.19%
Deaths	Norway	Male	Age-standardized	Aortic aneurysm	Rate	1990	12.23798314	12.7648746	11.64154624	
Deaths	Norway	Male	Age-standardized	Aortic aneurysm	Rate	2019	7.388368829	8.159359627	6.69697095	-39.63%
Deaths	Norway	Male	All Ages	Aortic aneurysm	Number	1990	370.9624787	387.1494171	353.1186185	
Deaths	Norway	Male	All Ages	Aortic aneurysm	Number	2019	336.048973	371.013145	305.0534009	-9.41%
DALYs	Oman	Male	Age-standardized	Aortic aneurysm	Rate	1990	64.08098808	100.0617468	36.12107341	
DALYs	Oman	Male	Age-standardized	Aortic aneurysm	Rate	2019	63.39368695	87.24598893	42.40492167	-1.07%
DALYs	Oman	Male	All Ages	Aortic aneurysm	Number	1990	267.2029437	426.3680888	153.4720847	
DALYs	Oman	Male	All Ages	Aortic aneurysm	Number	2019	701.2965121	1112.67254	433.7814715	162.46%
Deaths	Oman	Male	Age-standardized	Aortic aneurysm	Rate	1990	3.212355199	4.886866481	1.88354997	
Deaths	Oman	Male	Age-standardized	Aortic aneurysm	Rate	2019	3.699611954	5.104430712	2.390560798	15.17%

Deaths	Oman	Male	All Ages	Aortic aneurysm	Number	1990	9.034026665	14.15678112	5.07869587	
Deaths	Oman	Male	All Ages	Aortic aneurysm	Number	2019	23.98488671	34.51282926	15.79876004	165.49%
DALYs	Pakistan	Male	Age-standardized	Aortic aneurysm	Rate	1990	39.24853708	62.68439933	19.3469757	
DALYs	Pakistan	Male	Age-standardized	Aortic aneurysm	Rate	2019	55.42952592	81.03447913	35.60677807	41.23%
DALYs	Pakistan	Male	All Ages	Aortic aneurysm	Number	1990	12541.83603	20028.19165	6204.108237	
DALYs	Pakistan	Male	All Ages	Aortic aneurysm	Number	2019	33513.85444	50076.07672	21010.18237	167.22%
Deaths	Pakistan	Male	Age-standardized	Aortic aneurysm	Rate	1990	1.920195646	3.073684469	0.918299212	
Deaths	Pakistan	Male	Age-standardized	Aortic aneurysm	Rate	2019	2.659592204	3.79039088	1.752504607	38.51%
Deaths	Pakistan	Male	All Ages	Aortic aneurysm	Number	1990	555.0968679	885.7187339	267.612012	
Deaths	Pakistan	Male	All Ages	Aortic aneurysm	Number	2019	1285.931453	1867.642959	831.3680906	131.66%
DALYs	Palau	Male	Age-standardized	Aortic aneurysm	Rate	1990	119.9244772	165.7788042	69.49871824	
DALYs	Palau	Male	Age-standardized	Aortic aneurysm	Rate	2019	104.2328312	144.7813879	70.74460318	-13.08%
DALYs	Palau	Male	All Ages	Aortic aneurysm	Number	1990	6.484029348	9.072224009	3.569722004	
DALYs	Palau	Male	All Ages	Aortic aneurysm	Number	2019	11.73427241	16.54229031	7.608676785	80.97%
Deaths	Palau	Male	Age-standardized	Aortic aneurysm	Rate	1990	5.248363076	6.932465624	3.23358733	
Deaths	Palau	Male	Age-standardized	Aortic aneurysm	Rate	2019	4.547441038	6.097675677	3.265872452	-13.36%
Deaths	Palau	Male	All Ages	Aortic aneurysm	Number	1990	0.232959172	0.317897138	0.138340729	
Deaths	Palau	Male	All Ages	Aortic aneurysm	Number	2019	0.416269686	0.581037188	0.281313561	78.69%
DALYs	Palestine	Male	Age-standardized	Aortic aneurysm	Rate	1990	31.66839723	48.48705275	18.80189197	
DALYs	Palestine	Male	Age-standardized	Aortic aneurysm	Rate	2019	31.59237927	37.85723823	26.26027032	-0.24%
DALYs	Palestine	Male	All Ages	Aortic aneurysm	Number	1990	137.665727	215.3146178	81.01740532	
DALYs	Palestine	Male	All Ages	Aortic aneurysm	Number	2019	391.7490579	475.7586383	323.5368796	184.57%
Deaths	Palestine	Male	Age-standardized	Aortic aneurysm	Rate	1990	1.507021409	2.239926709	0.897715521	
Deaths	Palestine	Male	Age-standardized	Aortic aneurysm	Rate	2019	1.607826492	1.918883515	1.350347445	6.69%
Deaths	Palestine	Male	All Ages	Aortic aneurysm	Number	1990	5.478306336	8.251865366	3.261864469	
Deaths	Palestine	Male	All Ages	Aortic aneurysm	Number	2019	14.62450871	17.57554543	12.16359254	166.95%
DALYs	Panama	Male	Age-standardized	Aortic aneurysm	Rate	1990	68.15571487	76.51995694	60.67162961	
DALYs	Panama	Male	Age-standardized	Aortic aneurysm	Rate	2019	63.27974057	83.1924845	47.5509718	-7.15%
DALYs	Panama	Male	All Ages	Aortic aneurysm	Number	1990	542.970448	606.6828715	485.187281	
DALYs	Panama	Male	All Ages	Aortic aneurysm	Number	2019	1288.462757	1696.644438	965.7352589	137.30%
Deaths	Panama	Male	Age-standardized	Aortic aneurysm	Rate	1990	3.173455365	3.602999558	2.773577405	
Deaths	Panama	Male	Age-standardized	Aortic aneurysm	Rate	2019	2.901417284	3.75337432	2.194114819	-8.57%
Deaths	Panama	Male	All Ages	Aortic aneurysm	Number	1990	23.03400179	26.19203639	20.23067564	
Deaths	Panama	Male	All Ages	Aortic aneurysm	Number	2019	57.58307405	74.61075625	43.6852836	149.99%
DALYs	Papua New Guinea	Male	Age-standardized	Aortic aneurysm	Rate	1990	70.3465497	145.3523998	30.17076141	
DALYs	Papua New Guinea	Male	Age-standardized	Aortic aneurysm	Rate	2019	68.47224546	122.4019301	35.09979206	-2.66%
DALYs	Papua New Guinea	Male	All Ages	Aortic aneurysm	Number	1990	733.9740334	1531.828195	305.7588679	
DALYs	Papua New Guinea	Male	All Ages	Aortic aneurysm	Number	2019	1900.661632	3401.773406	928.4650064	158.95%
Deaths	Papua New Guinea	Male	Age-standardized	Aortic aneurysm	Rate	1990	3.259001184	6.698700992	1.450898103	
Deaths	Papua New Guinea	Male	Age-standardized	Aortic aneurysm	Rate	2019	3.154385553	5.511012327	1.703386652	-3.21%
Deaths	Papua New Guinea	Male	All Ages	Aortic aneurysm	Number	1990	25.72712869	53.16087332	11.03284729	
Deaths	Papua New Guinea	Male	All Ages	Aortic aneurysm	Number	2019	65.69431007	117.6211171	33.44780107	155.35%
DALYs	Paraguay	Male	Age-standardized	Aortic aneurysm	Rate	1990	60.53833674	72.46682537	48.88222089	
DALYs	Paraguay	Male	Age-standardized	Aortic aneurysm	Rate	2019	87.29540894	117.5429845	63.81694345	44.20%
DALYs	Paraguay	Male	All Ages	Aortic aneurysm	Number	1990	676.2615022	815.4633903	544.0428038	
DALYs	Paraguay	Male	All Ages	Aortic aneurysm	Number	2019	2420.063351	3271.644478	1762.016887	257.86%
Deaths	Paraguay	Male	Age-standardized	Aortic aneurysm	Rate	1990	2.747271734	3.305532268	2.226134879	
Deaths	Paraguay	Male	Age-standardized	Aortic aneurysm	Rate	2019	3.944524853	5.255826205	2.944752162	43.58%
Deaths	Paraguay	Male	All Ages	Aortic aneurysm	Number	1990	27.55911626	33.10050881	22.3147152	
Deaths	Paraguay	Male	All Ages	Aortic aneurysm	Number	2019	99.69773554	133.3734186	73.48764302	261.76%
DALYs	Peru	Male	Age-standardized	Aortic aneurysm	Rate	1990	36.6486361	45.73550006	27.08406476	
DALYs	Peru	Male	Age-standardized	Aortic aneurysm	Rate	2019	26.852454	36.40364907	18.80021967	-26.73%
DALYs	Peru	Male	All Ages	Aortic aneurysm	Number	1990	2287.307808	2877.628318	1656.59264	
DALYs	Peru	Male	All Ages	Aortic aneurysm	Number	2019	4201.304893	5690.473235	2939.269362	83.68%
Deaths	Peru	Male	Age-standardized	Aortic aneurysm	Rate	1990	1.760339505	2.15332987	1.358685013	
Deaths	Peru	Male	Age-standardized	Aortic aneurysm	Rate	2019	1.372007236	1.8264928	0.981572986	-22.06%
Deaths	Peru	Male	All Ages	Aortic aneurysm	Number	1990	95.94717841	117.8772903	73.70300782	
Deaths	Peru	Male	All Ages	Aortic aneurysm	Number	2019	209.7388745	279.5097307	149.820853	118.60%
DALYs	Philippines	Male	Age-standardized	Aortic aneurysm	Rate	1990	23.30280108	28.18633585	19.75105753	
DALYs	Philippines	Male	Age-standardized	Aortic aneurysm	Rate	2019	47.72455575	61.43171274	35.28883144	104.80%
DALYs	Philippines	Male	All Ages	Aortic aneurysm	Number	1990	3214.507638	4151.320071	2703.834539	
DALYs	Philippines	Male	All Ages	Aortic aneurysm	Number	2019	17720.42599	22992.87268	12929.90438	451.26%
Deaths	Philippines	Male	Age-standardized	Aortic aneurysm	Rate	1990	1.328693043	1.548645127	1.134912025	
Deaths	Philippines	Male	Age-standardized	Aortic aneurysm	Rate	2019	2.329336073	2.964452332	1.760725621	75.31%
Deaths	Philippines	Male	All Ages	Aortic aneurysm	Number	1990	144.7377007	173.9243148	122.789023	
Deaths	Philippines	Male	All Ages	Aortic aneurysm	Number	2019	696.5180816	896.1314123	515.8128458	381.23%
DALYs	Poland	Male	Age-standardized	Aortic aneurysm	Rate	1990	83.57948222	86.89336643	80.49098831	
DALYs	Poland	Male	Age-standardized	Aortic aneurysm	Rate	2019	121.344912	151.3035399	95.712944	45.19%
DALYs	Poland	Male	All Ages	Aortic aneurysm	Number	1990	15668.10376	16285.76949	15105.78662	
DALYs	Poland	Male	All Ages	Aortic aneurysm	Number	2019	34725.20205	43400.6477	27342.40172	121.63%
Deaths	Poland	Male	Age-standardized	Aortic aneurysm	Rate	1990	3.552097729	3.69134282	3.391830801	
Deaths	Poland	Male	Age-standardized	Aortic aneurysm	Rate	2019	5.661659182	6.98635953	4.50368799	59.39%
Deaths	Poland	Male	All Ages	Aortic aneurysm	Number	1990	616.0104692	641.6262142	591.7061836	
Deaths	Poland	Male	All Ages	Aortic aneurysm	Number	2019	1610.469783	1993.198494	1272.678462	161.44%
DALYs	Portugal	Male	Age-standardized	Aortic aneurysm	Rate	1990	57.34805278	60.67453747	54.06643436	
DALYs	Portugal	Male	Age-standardized	Aortic aneurysm	Rate	2019	61.99392653	69.32564206	55.35453463	8.10%
DALYs	Portugal	Male	All Ages	Aortic aneurysm	Number	1990	3393.762568	3607.055656	3187.060572	
DALYs	Portugal	Male	All Ages	Aortic aneurysm	Number	2019	5749.307187	6444.877023	5128.888047	69.41%
Deaths	Portugal	Male	Age-standardized	Aortic aneurysm	Rate	1990	2.547571315	2.710477388	2.393835973	
Deaths	Portugal	Male	Age-standardized	Aortic aneurysm	Rate	2019	2.883272463	3.217139744	2.574368062	13.18%
Deaths	Portugal	Male	All Ages	Aortic aneurysm	Number	1990	145.9602643	155.9479856	136.7776622	
Deaths	Portugal	Male	All Ages	Aortic aneurysm	Number	2019	298.195154	334.854663	265.1081001	104.30%
DALYs	Puerto Rico	Male	Age-standardized	Aortic aneurysm	Rate	1990	54.45846655	60.24840107	49.4043838	
DALYs	Puerto Rico	Male	Age-standardized	Aortic aneurysm	Rate	2019	32.50560901	42.00356975	24.86422069	-40.31%
DALYs	Puerto Rico	Male	All Ages	Aortic aneurysm	Number	1990	912.7543715	1011.490458	825.666228	
DALYs	Puerto Rico	Male	All Ages	Aortic aneurysm	Number	2019	974.9363041	1254.439664	747.5776829	6.81%
Deaths	Puerto Rico	Male	Age-standardized	Aortic aneurysm	Rate	1990	2.943762731	3.259921159	2.614854482	
Deaths	Puerto Rico	Male	Age-standardized	Aortic aneurysm	Rate	2019	1.724653851	2.181462498	1.345656165	-41.41%
Deaths	Puerto Rico	Male	All Ages	Aortic aneurysm	Number	1990	47.37758693	52.92305553	42.08945273	
Deaths	Puerto Rico	Male	All Ages	Aortic aneurysm	Number	2019	57.19507714	71.8380512	44.69099061	20.72%
DALYs	Qatar	Male	Age-standardized	Aortic aneurysm	Rate	1990	51.50405787	71.6083402	35.53487218	
DALYs	Qatar	Male	Age-standardized	Aortic aneurysm	Rate	2019	38.43389207	54.59781693	25.43126297	-25.38%
DALYs	Qatar	Male	All Ages	Aortic aneurysm	Number	1990	48.77760621	68.41857017	32.26558453	
DALYs	Qatar	Male	All Ages	Aortic aneurysm	Number	2019	290.1382655	431.9244788	183.7769624	494.82%
Deaths	Qatar	Male	Age-standardized	Aortic aneurysm	Rate	1990	2.812680351	3.838982207	1.961302606	
Deaths	Qatar	Male	Age-standardized	Aortic aneurysm	Rate	2019	2.542604239	3.565510628	1.702215232	-9.60%
Deaths	Qatar	Male	All Ages	Aortic aneurysm	Number	1990	1.437484422	2.013222419	0.967386744	
Deaths	Qatar	Male	All Ages	Aortic aneurysm	Number	2019	8.789165284	12.84275841	5.554450986	511.43%
DALYs	Republic of Korea	Male	Age-standardized	Aortic aneurysm	Rate	1990	49.00300569	62.97733048	35.95169387	
DALYs	Republic of Korea	Male	Age-standardized	Aortic aneurysm	Rate	2019	36.6563979	42.96836122	30.64764077	-25.20%
DALYs	Republic of Korea	Male	All Ages	Aortic aneurysm	Number	1990	6293.56342	8154.993652	4508.871678	

DALYs	Republic of Korea	Male	All Ages	Aortic aneurysm	Number	2019	13917.41771	16547.37607	11611.81632	121.14%
Deaths	Republic of Korea	Male	Age-standardized	Aortic aneurysm	Rate	1990	2.585463319	3.316466906	1.935540259	
Deaths	Republic of Korea	Male	Age-standardized	Aortic aneurysm	Rate	2019	2.159338255	2.583167316	1.785649913	-16.48%
Deaths	Republic of Korea	Male	All Ages	Aortic aneurysm	Number	1990	237.6201139	306.3100436	173.4397212	
Deaths	Republic of Korea	Male	All Ages	Aortic aneurysm	Number	2019	730.637511	868.9572967	604.4733435	207.48%
DALYs	Republic of Moldova	Male	Age-standardized	Aortic aneurysm	Rate	1990	37.93025355	43.19041756	32.53721537	
DALYs	Republic of Moldova	Male	Age-standardized	Aortic aneurysm	Rate	2019	57.26923212	68.95067892	47.69416772	50.99%
DALYs	Republic of Moldova	Male	All Ages	Aortic aneurysm	Number	1990	717.0735026	822.8700152	611.3649619	
DALYs	Republic of Moldova	Male	All Ages	Aortic aneurysm	Number	2019	1344.23156	1624.830695	1120.996477	87.46%
Deaths	Republic of Moldova	Male	Age-standardized	Aortic aneurysm	Rate	1990	1.575455366	1.802139467	1.346824102	
Deaths	Republic of Moldova	Male	Age-standardized	Aortic aneurysm	Rate	2019	2.253491066	2.667660612	1.903723797	43.04%
Deaths	Republic of Moldova	Male	All Ages	Aortic aneurysm	Number	1990	25.23746908	28.9419848	21.43825638	
Deaths	Republic of Moldova	Male	All Ages	Aortic aneurysm	Number	2019	51.54286501	61.53744032	43.36241664	104.23%
DALYs	Romania	Male	Age-standardized	Aortic aneurysm	Rate	1990	51.55534651	57.21755976	46.31249716	
DALYs	Romania	Male	Age-standardized	Aortic aneurysm	Rate	2019	75.36727513	91.7297939	61.22892741	46.19%
DALYs	Romania	Male	All Ages	Aortic aneurysm	Number	1990	6466.180891	7231.847374	5788.965176	
DALYs	Romania	Male	All Ages	Aortic aneurysm	Number	2019	10889.44866	13311.65768	8828.776117	68.41%
Deaths	Romania	Male	Age-standardized	Aortic aneurysm	Rate	1990	2.100438968	2.360109363	1.884606798	
Deaths	Romania	Male	Age-standardized	Aortic aneurysm	Rate	2019	3.184285516	3.829834535	2.612999048	51.60%
Deaths	Romania	Male	All Ages	Aortic aneurysm	Number	1990	244.0206784	273.5884649	218.5647997	
Deaths	Romania	Male	All Ages	Aortic aneurysm	Number	2019	483.8653831	583.9164473	395.4746174	98.29%
DALYs	Russian Federation	Male	Age-standardized	Aortic aneurysm	Rate	1990	107.5080821	119.9412209	96.59473626	
DALYs	Russian Federation	Male	Age-standardized	Aortic aneurysm	Rate	2019	153.1481208	186.0384346	123.188393	42.45%
DALYs	Russian Federation	Male	All Ages	Aortic aneurysm	Number	1990	71223.84779	80431.30686	62964.09117	
DALYs	Russian Federation	Male	All Ages	Aortic aneurysm	Number	2019	136804.5375	167013.719	109470.054	92.08%
Deaths	Russian Federation	Male	Age-standardized	Aortic aneurysm	Rate	1990	4.727162761	5.164155414	4.393442306	
Deaths	Russian Federation	Male	Age-standardized	Aortic aneurysm	Rate	2019	6.631740613	7.948686338	5.452897118	40.29%
Deaths	Russian Federation	Male	All Ages	Aortic aneurysm	Number	1990	2654.363718	2937.782466	2416.737513	
Deaths	Russian Federation	Male	All Ages	Aortic aneurysm	Number	2019	5596.862884	6771.342935	4546.876658	110.86%
DALYs	Rwanda	Male	Age-standardized	Aortic aneurysm	Rate	1990	114.646192	192.4470447	51.30869331	
DALYs	Rwanda	Male	Age-standardized	Aortic aneurysm	Rate	2019	56.74738441	83.62919471	31.92122365	-50.50%
DALYs	Rwanda	Male	All Ages	Aortic aneurysm	Number	1990	1669.546028	2827.592497	737.9521949	
DALYs	Rwanda	Male	All Ages	Aortic aneurysm	Number	2019	1595.136422	2380.269154	907.7617649	-4.46%
Deaths	Rwanda	Male	Age-standardized	Aortic aneurysm	Rate	1990	5.004295267	8.100917678	2.221705268	
Deaths	Rwanda	Male	Age-standardized	Aortic aneurysm	Rate	2019	2.751050751	3.994943324	1.531780201	-45.03%
Deaths	Rwanda	Male	All Ages	Aortic aneurysm	Number	1990	59.94182485	99.53316551	26.63487827	
Deaths	Rwanda	Male	All Ages	Aortic aneurysm	Number	2019	58.14635131	85.79865291	32.64843031	-3.00%
DALYs	Saint Kitts and Nevis	Male	Age-standardized	Aortic aneurysm	Rate	1990	96.16830256	111.9795192	78.57620468	
DALYs	Saint Kitts and Nevis	Male	Age-standardized	Aortic aneurysm	Rate	2019	85.50259089	100.9829118	71.05220627	-11.09%
DALYs	Saint Kitts and Nevis	Male	All Ages	Aortic aneurysm	Number	1990	16.14485942	19.0081961	13.0746291	
DALYs	Saint Kitts and Nevis	Male	All Ages	Aortic aneurysm	Number	2019	26.193212	31.45178654	20.98963692	62.24%
Deaths	Saint Kitts and Nevis	Male	Age-standardized	Aortic aneurysm	Rate	1990	5.174678696	5.956049737	4.375225277	
Deaths	Saint Kitts and Nevis	Male	Age-standardized	Aortic aneurysm	Rate	2019	4.608281633	5.32583086	3.94563293	-10.95%
Deaths	Saint Kitts and Nevis	Male	All Ages	Aortic aneurysm	Number	1990	0.825316148	0.96979917	0.67587452	
Deaths	Saint Kitts and Nevis	Male	All Ages	Aortic aneurysm	Number	2019	1.166274599	1.374392789	0.978968233	41.31%
DALYs	Saint Lucia	Male	Age-standardized	Aortic aneurysm	Rate	1990	139.0320472	158.7497367	120.8631814	
DALYs	Saint Lucia	Male	Age-standardized	Aortic aneurysm	Rate	2019	125.8502855	151.8005562	100.0181177	-9.48%
DALYs	Saint Lucia	Male	All Ages	Aortic aneurysm	Number	1990	52.77758438	60.66575796	45.77932292	
DALYs	Saint Lucia	Male	All Ages	Aortic aneurysm	Number	2019	124.690174	151.2888169	99.49831491	136.26%
Deaths	Saint Lucia	Male	Age-standardized	Aortic aneurysm	Rate	1990	7.809839877	8.897054286	6.814879152	
Deaths	Saint Lucia	Male	Age-standardized	Aortic aneurysm	Rate	2019	6.9678233	8.387263531	5.540742265	-10.78%
Deaths	Saint Lucia	Male	All Ages	Aortic aneurysm	Number	1990	2.582334436	2.983566096	2.230037248	
Deaths	Saint Lucia	Male	All Ages	Aortic aneurysm	Number	2019	6.320059218	7.659193453	5.025944048	144.74%
DALYs	Saint Vincent and the Grenadines	Male	Age-standardized	Aortic aneurysm	Rate	1990	74.80071814	86.09698746	65.55591511	
DALYs	Saint Vincent and the Grenadines	Male	Age-standardized	Aortic aneurysm	Rate	2019	77.76952636	91.32362368	64.3021205	3.97%
DALYs	Saint Vincent and the Grenadines	Male	All Ages	Aortic aneurysm	Number	1990	24.1868616	27.76028215	21.20537958	
DALYs	Saint Vincent and the Grenadines	Male	All Ages	Aortic aneurysm	Number	2019	52.91197336	62.32370347	43.77220728	118.76%
Deaths	Saint Vincent and the Grenadines	Male	Age-standardized	Aortic aneurysm	Rate	1990	4.0977889	4.712033024	3.555818942	
Deaths	Saint Vincent and the Grenadines	Male	Age-standardized	Aortic aneurysm	Rate	2019	4.218316165	4.967842281	3.524764657	2.94%
Deaths	Saint Vincent and the Grenadines	Male	All Ages	Aortic aneurysm	Number	1990	1.189749931	1.377611374	1.029646508	
Deaths	Saint Vincent and the Grenadines	Male	All Ages	Aortic aneurysm	Number	2019	2.661284973	3.145855033	2.206516545	123.68%
DALYs	Samoa	Male	Age-standardized	Aortic aneurysm	Rate	1990	103.5513424	145.450613	71.33266872	
DALYs	Samoa	Male	Age-standardized	Aortic aneurysm	Rate	2019	74.81443464	101.1656048	50.885693	-27.75%
DALYs	Samoa	Male	All Ages	Aortic aneurysm	Number	1990	46.15230349	65.58721485	31.15867635	
DALYs	Samoa	Male	All Ages	Aortic aneurysm	Number	2019	55.8283814	77.49797286	36.45536258	20.97%
Deaths	Samoa	Male	Age-standardized	Aortic aneurysm	Rate	1990	4.906982245	6.6441584	3.475346238	
Deaths	Samoa	Male	Age-standardized	Aortic aneurysm	Rate	2019	3.503138528	4.601321807	2.465690982	-28.61%
Deaths	Samoa	Male	All Ages	Aortic aneurysm	Number	1990	1.796113848	2.485540922	1.252067288	
Deaths	Samoa	Male	All Ages	Aortic aneurysm	Number	2019	2.219712896	2.951706935	1.538440512	23.58%
DALYs	San Marino	Male	Age-standardized	Aortic aneurysm	Rate	1990	107.997483	130.0135969	88.67833396	
DALYs	San Marino	Male	Age-standardized	Aortic aneurysm	Rate	2019	90.77173614	128.8635089	58.63159451	-15.95%
DALYs	San Marino	Male	All Ages	Aortic aneurysm	Number	1990	16.11710173	19.49292136	13.12724153	
DALYs	San Marino	Male	All Ages	Aortic aneurysm	Number	2019	26.67028947	37.22615472	17.4195494	65.48%
Deaths	San Marino	Male	Age-standardized	Aortic aneurysm	Rate	1990	6.22540698	7.368743774	5.129073852	
Deaths	San Marino	Male	Age-standardized	Aortic aneurysm	Rate	2019	5.316568483	7.331195781	3.529883913	-14.60%
Deaths	San Marino	Male	All Ages	Aortic aneurysm	Number	1990	0.88492558	1.051824231	0.72012041	
Deaths	San Marino	Male	All Ages	Aortic aneurysm	Number	2019	1.69986679	2.343049996	1.129698643	92.09%
DALYs	Sao Tome and Principe	Male	Age-standardized	Aortic aneurysm	Rate	1990	35.04752176	51.6964932	20.31954417	
DALYs	Sao Tome and Principe	Male	Age-standardized	Aortic aneurysm	Rate	2019	49.75058561	65.78122393	37.18942457	41.95%
DALYs	Sao Tome and Principe	Male	All Ages	Aortic aneurysm	Number	1990	10.76493028	16.14095123	6.223011214	
DALYs	Sao Tome and Principe	Male	All Ages	Aortic aneurysm	Number	2019	25.85219979	35.26830926	18.89787499	140.15%
Deaths	Sao Tome and Principe	Male	Age-standardized	Aortic aneurysm	Rate	1990	1.811567499	2.636460897	1.071978064	
Deaths	Sao Tome and Principe	Male	Age-standardized	Aortic aneurysm	Rate	2019	2.538659505	3.288066154	1.947351099	40.14%
Deaths	Sao Tome and Principe	Male	All Ages	Aortic aneurysm	Number	1990	0.473433555	0.698050803	0.275253132	
Deaths	Sao Tome and Principe	Male	All Ages	Aortic aneurysm	Number	2019	1.078544652	1.419373593	0.810151768	127.81%
DALYs	Saudi Arabia	Male	Age-standardized	Aortic aneurysm	Rate	1990	22.56405083	36.09481694	12.33948069	
DALYs	Saudi Arabia	Male	Age-standardized	Aortic aneurysm	Rate	2019	26.95532324	35.2946155	19.85019561	19.46%
DALYs	Saudi Arabia	Male	All Ages	Aortic aneurysm	Number	1990	872.9494764	1433.378592	469.5549857	
DALYs	Saudi Arabia	Male	All Ages	Aortic aneurysm	Number	2019	3470.15281	4809.817562	2411.502837	297.52%
Deaths	Saudi Arabia	Male	Age-standardized	Aortic aneurysm	Rate	1990	1.120061413	1.745580596	0.626536331	
Deaths	Saudi Arabia	Male	Age-standardized	Aortic aneurysm	Rate	2019	1.376496891	1.756853211	1.050696626	22.89%
Deaths	Saudi Arabia	Male	All Ages	Aortic aneurysm	Number	1990	32.16920232	51.62410289	17.63462266	
Deaths	Saudi Arabia	Male	All Ages	Aortic aneurysm	Number	2019	115.0314169	153.8531895	83.73651375	257.58%
DALYs	Senegal	Male	Age-standardized	Aortic aneurysm	Rate	1990	39.26501952	61.25024773	21.1593373	
DALYs	Senegal	Male	Age-standardized	Aortic aneurysm	Rate	2019	40.81741209	56.78586803	27.80187977	3.95%
DALYs	Senegal	Male	All Ages	Aortic aneurysm	Number	1990	665.2983094	1045.962288	357.9560405	
DALYs	Senegal	Male	All Ages	Aortic aneurysm	Number	2019	1526.046401	2122.583408	1016.448611	129.38%
Deaths	Senegal	Male	Age-standardized	Aortic aneurysm	Rate	1990	1.930629871	3.002346426	1.051027991	
Deaths	Senegal	Male	Age-standardized	Aortic aneurysm	Rate	2019	2.06156751	2.813962459	1.449775861	6.78%
Deaths	Senegal	Male	All Ages	Aortic aneurysm	Number	1990	27.9827553	43.78357942	15.09634791	
Deaths	Senegal	Male	All Ages	Aortic aneurysm	Number	2019	64.64407368	89.51069878	44.51090274	131.01%

DALYs	Serbia	Male	Age-standardized	Aortic aneurysm	Rate	1990	112.9159863	133.714484	96.26163865	
DALYs	Serbia	Male	Age-standardized	Aortic aneurysm	Rate	2019	136.8661588	174.4368677	103.3424694	21.21%
DALYs	Serbia	Male	All Ages	Aortic aneurysm	Number	1990	6167.701657	7342.782664	5236.065774	
DALYs	Serbia	Male	All Ages	Aortic aneurysm	Number	2019	9597.106943	12252.1659	7257.69333	55.60%
Deaths	Serbia	Male	Age-standardized	Aortic aneurysm	Rate	1990	4.784890693	5.66517156	4.056815342	
Deaths	Serbia	Male	Age-standardized	Aortic aneurysm	Rate	2019	6.494515292	8.107938222	5.063920467	35.73%
Deaths	Serbia	Male	All Ages	Aortic aneurysm	Number	1990	241.7619393	289.456573	205.6465451	
Deaths	Serbia	Male	All Ages	Aortic aneurysm	Number	2019	460.2503777	577.3104266	355.0623046	90.37%
DALYs	Seychelles	Male	Age-standardized	Aortic aneurysm	Rate	1990	63.10195403	75.71234143	46.73424013	
DALYs	Seychelles	Male	Age-standardized	Aortic aneurysm	Rate	2019	59.7049099	84.02786557	42.04900492	-5.38%
DALYs	Seychelles	Male	All Ages	Aortic aneurysm	Number	1990	15.93059606	19.12324647	11.92570599	
DALYs	Seychelles	Male	All Ages	Aortic aneurysm	Number	2019	31.08658255	44.06918873	21.95383988	95.14%
Deaths	Seychelles	Male	Age-standardized	Aortic aneurysm	Rate	1990	3.161061727	3.8017032	2.326956029	
Deaths	Seychelles	Male	Age-standardized	Aortic aneurysm	Rate	2019	3.001581843	4.20803424	2.132947257	-5.05%
Deaths	Seychelles	Male	All Ages	Aortic aneurysm	Number	1990	0.714268173	0.8545944	0.530960356	
Deaths	Seychelles	Male	All Ages	Aortic aneurysm	Number	2019	1.295449669	1.818653898	0.918323005	81.37%
DALYs	Sierra Leone	Male	Age-standardized	Aortic aneurysm	Rate	1990	40.52119671	66.31316763	19.56356281	
DALYs	Sierra Leone	Male	Age-standardized	Aortic aneurysm	Rate	2019	40.12881168	61.14353334	24.36968748	-0.97%
DALYs	Sierra Leone	Male	All Ages	Aortic aneurysm	Number	1990	410.0472186	671.7920615	197.7239642	
DALYs	Sierra Leone	Male	All Ages	Aortic aneurysm	Number	2019	764.7867959	1181.194672	454.2350447	86.51%
Deaths	Sierra Leone	Male	Age-standardized	Aortic aneurysm	Rate	1990	1.99806921	3.231696396	0.971723149	
Deaths	Sierra Leone	Male	Age-standardized	Aortic aneurysm	Rate	2019	2.012434038	2.991059262	1.241023764	0.72%
Deaths	Sierra Leone	Male	All Ages	Aortic aneurysm	Number	1990	18.35790526	29.78442571	8.951001947	
Deaths	Sierra Leone	Male	All Ages	Aortic aneurysm	Number	2019	31.12094201	47.10418005	19.11134371	69.52%
DALYs	Singapore	Male	Age-standardized	Aortic aneurysm	Rate	1990	101.8870483	110.4501831	93.98153896	
DALYs	Singapore	Male	Age-standardized	Aortic aneurysm	Rate	2019	48.61966691	54.61184897	42.9667558	-52.28%
DALYs	Singapore	Male	All Ages	Aortic aneurysm	Number	1990	1072.292569	1168.20599	989.9479839	
DALYs	Singapore	Male	All Ages	Aortic aneurysm	Number	2019	1773.422774	1992.542749	1561.465852	65.39%
Deaths	Singapore	Male	Age-standardized	Aortic aneurysm	Rate	1990	5.229637646	5.689378761	4.767037893	
Deaths	Singapore	Male	Age-standardized	Aortic aneurysm	Rate	2019	2.767674162	3.157147044	2.403044071	-47.08%
Deaths	Singapore	Male	All Ages	Aortic aneurysm	Number	1990	44.37380456	48.23310502	40.74700989	
Deaths	Singapore	Male	All Ages	Aortic aneurysm	Number	2019	90.10359388	102.18511	79.01958741	103.06%
DALYs	Slovakia	Male	Age-standardized	Aortic aneurysm	Rate	1990	62.84132274	72.9504073	53.7008228	
DALYs	Slovakia	Male	Age-standardized	Aortic aneurysm	Rate	2019	76.44336678	99.28263862	57.09540601	21.65%
DALYs	Slovakia	Male	All Ages	Aortic aneurysm	Number	1990	1635.219016	1911.842808	1393.989819	
DALYs	Slovakia	Male	All Ages	Aortic aneurysm	Number	2019	2933.286654	3820.050408	2179.775965	79.38%
Deaths	Slovakia	Male	Age-standardized	Aortic aneurysm	Rate	1990	2.558705866	2.961367739	2.203000753	
Deaths	Slovakia	Male	Age-standardized	Aortic aneurysm	Rate	2019	3.314368094	4.193201672	2.519005357	29.53%
Deaths	Slovakia	Male	All Ages	Aortic aneurysm	Number	1990	62.56776342	72.99092975	53.65628727	
Deaths	Slovakia	Male	All Ages	Aortic aneurysm	Number	2019	123.0586786	156.2197744	92.80753184	96.68%
DALYs	Slovenia	Male	Age-standardized	Aortic aneurysm	Rate	1990	84.78769146	110.3526295	63.87049683	
DALYs	Slovenia	Male	Age-standardized	Aortic aneurysm	Rate	2019	86.43708935	115.4907197	65.42446676	1.95%
DALYs	Slovenia	Male	All Ages	Aortic aneurysm	Number	1990	849.7693334	1108.761912	638.8891011	
DALYs	Slovenia	Male	All Ages	Aortic aneurysm	Number	2019	1564.328488	2093.680205	1188.11602	84.09%
Deaths	Slovenia	Male	Age-standardized	Aortic aneurysm	Rate	1990	3.756551212	4.872874218	2.86781888	
Deaths	Slovenia	Male	Age-standardized	Aortic aneurysm	Rate	2019	4.272298252	5.5910931	3.318022945	13.73%
Deaths	Slovenia	Male	All Ages	Aortic aneurysm	Number	1990	34.89049805	45.38999531	26.49273559	
Deaths	Slovenia	Male	All Ages	Aortic aneurysm	Number	2019	78.94216058	103.6786744	61.29142232	126.26%
DALYs	Solomon Islands	Male	Age-standardized	Aortic aneurysm	Rate	1990	100.2632511	194.9762045	37.92128545	
DALYs	Solomon Islands	Male	Age-standardized	Aortic aneurysm	Rate	2019	95.40609133	168.0377776	39.61289086	-4.84%
DALYs	Solomon Islands	Male	All Ages	Aortic aneurysm	Number	1990	87.28325246	174.1265704	29.62541573	
DALYs	Solomon Islands	Male	All Ages	Aortic aneurysm	Number	2019	180.0458026	329.4381097	66.7512364	106.28%
Deaths	Solomon Islands	Male	Age-standardized	Aortic aneurysm	Rate	1990	4.048039516	7.574595604	1.739325168	
Deaths	Solomon Islands	Male	Age-standardized	Aortic aneurysm	Rate	2019	3.90343091	6.634864343	1.78121463	-3.57%
Deaths	Solomon Islands	Male	All Ages	Aortic aneurysm	Number	1990	2.825801388	5.487531193	1.082428088	
Deaths	Solomon Islands	Male	All Ages	Aortic aneurysm	Number	2019	5.646270113	9.981377791	2.319047196	99.81%
DALYs	Somalia	Male	Age-standardized	Aortic aneurysm	Rate	1990	89.85566806	176.0380313	32.3319195	
DALYs	Somalia	Male	Age-standardized	Aortic aneurysm	Rate	2019	54.36667571	103.4459643	18.41209034	-39.50%
DALYs	Somalia	Male	All Ages	Aortic aneurysm	Number	1990	1219.613593	2484.920251	440.1475321	
DALYs	Somalia	Male	All Ages	Aortic aneurysm	Number	2019	1891.944915	3725.364877	647.8743808	55.13%
Deaths	Somalia	Male	Age-standardized	Aortic aneurysm	Rate	1990	3.95316551	7.35774701	1.420224375	
Deaths	Somalia	Male	Age-standardized	Aortic aneurysm	Rate	2019	2.4451899	4.526370773	0.822837321	-38.15%
Deaths	Somalia	Male	All Ages	Aortic aneurysm	Number	1990	41.20001387	81.3270401	14.75484057	
Deaths	Somalia	Male	All Ages	Aortic aneurysm	Number	2019	60.72118121	117.0748683	20.60600852	47.38%
DALYs	South Africa	Male	Age-standardized	Aortic aneurysm	Rate	1990	93.20424941	108.9909318	81.93958846	
DALYs	South Africa	Male	Age-standardized	Aortic aneurysm	Rate	2019	64.38521248	72.99607055	55.74565283	-30.92%
DALYs	South Africa	Male	All Ages	Aortic aneurysm	Number	1990	8847.523577	10259.52493	7754.868248	
DALYs	South Africa	Male	All Ages	Aortic aneurysm	Number	2019	12041.10865	13814.2006	10394.15678	36.10%
Deaths	South Africa	Male	Age-standardized	Aortic aneurysm	Rate	1990	4.810873117	5.633808421	4.213078285	
Deaths	South Africa	Male	Age-standardized	Aortic aneurysm	Rate	2019	3.522031756	3.974125355	3.072645256	-26.79%
Deaths	South Africa	Male	All Ages	Aortic aneurysm	Number	1990	366.8588193	428.5916958	322.2575622	
Deaths	South Africa	Male	All Ages	Aortic aneurysm	Number	2019	521.8024566	591.8449575	453.0603562	42.24%
DALYs	South Sudan	Male	Age-standardized	Aortic aneurysm	Rate	1990	72.18663664	121.3516923	38.4793815	
DALYs	South Sudan	Male	Age-standardized	Aortic aneurysm	Rate	2019	42.07863674	70.00351912	22.72449084	-41.71%
DALYs	South Sudan	Male	All Ages	Aortic aneurysm	Number	1990	1017.548016	1733.088086	544.0484751	
DALYs	South Sudan	Male	All Ages	Aortic aneurysm	Number	2019	879.6270173	1466.398637	469.6643377	-13.55%
Deaths	South Sudan	Male	Age-standardized	Aortic aneurysm	Rate	1990	3.401586697	5.604811251	1.814044434	
Deaths	South Sudan	Male	Age-standardized	Aortic aneurysm	Rate	2019	2.070732771	3.412002319	1.134604517	-39.12%
Deaths	South Sudan	Male	All Ages	Aortic aneurysm	Number	1990	40.09567559	67.01800892	21.47128807	
Deaths	South Sudan	Male	All Ages	Aortic aneurysm	Number	2019	35.32005234	58.27917681	19.18377988	-11.91%
DALYs	Spain	Male	Age-standardized	Aortic aneurysm	Rate	1990	85.41154483	90.36822568	80.61206136	
DALYs	Spain	Male	Age-standardized	Aortic aneurysm	Rate	2019	77.88442579	86.40994496	69.7031577	-8.81%
DALYs	Spain	Male	All Ages	Aortic aneurysm	Number	1990	20089.91357	21303.57827	18933.72272	
DALYs	Spain	Male	All Ages	Aortic aneurysm	Number	2019	31397.5371	34914.82875	28159.2142	56.29%
Deaths	Spain	Male	Age-standardized	Aortic aneurysm	Rate	1990	4.12275536	4.386264736	3.861045091	
Deaths	Spain	Male	Age-standardized	Aortic aneurysm	Rate	2019	4.14486185	4.639207679	3.673136487	0.54%
Deaths	Spain	Male	All Ages	Aortic aneurysm	Number	1990	943.5617677	1005.49029	884.5306284	
Deaths	Spain	Male	All Ages	Aortic aneurysm	Number	2019	1820.067077	2045.130335	1601.712563	92.89%
DALYs	Sri Lanka	Male	Age-standardized	Aortic aneurysm	Rate	1990	44.40890247	54.00886268	35.81123782	
DALYs	Sri Lanka	Male	Age-standardized	Aortic aneurysm	Rate	2019	42.18073562	57.33695774	30.29562632	-5.02%
DALYs	Sri Lanka	Male	All Ages	Aortic aneurysm	Number	1990	2390.004788	2945.615364	1915.297049	
DALYs	Sri Lanka	Male	All Ages	Aortic aneurysm	Number	2019	4489.563644	6140.429105	3169.652225	87.85%
Deaths	Sri Lanka	Male	Age-standardized	Aortic aneurysm	Rate	1990	2.446148739	2.956856099	1.971978226	
Deaths	Sri Lanka	Male	Age-standardized	Aortic aneurysm	Rate	2019	2.462551663	3.293948745	1.797823155	0.67%
Deaths	Sri Lanka	Male	All Ages	Aortic aneurysm	Number	1990	106.2671266	128.0939483	85.54095771	
Deaths	Sri Lanka	Male	All Ages	Aortic aneurysm	Number	2019	225.7435357	306.481018	162.2752694	112.43%
DALYs	Sudan	Male	Age-standardized	Aortic aneurysm	Rate	1990	33.34950445	61.61205432	13.95298214	
DALYs	Sudan	Male	Age-standardized	Aortic aneurysm	Rate	2019	32.94296136	58.53173066	18.20481899	-1.22%
DALYs	Sudan	Male	All Ages	Aortic aneurysm	Number	1990	1734.426647	3248.998804	720.8109591	
DALYs	Sudan	Male	All Ages	Aortic aneurysm	Number	2019	3583.137673	6458.608312	1928.943178	106.59%
Deaths	Sudan	Male	Age-standardized	Aortic aneurysm	Rate	1990	1.526814472	2.729019741	0.643842499	

Deaths	Sudan	Male	Age-standardized	Aortic aneurysm	Rate	2019	1.603436619	2.806129211	0.921007046	5.02%
Deaths	Sudan	Male	All Ages	Aortic aneurysm	Number	1990	67.29729226	122.3452109	28.3013938	
Deaths	Sudan	Male	All Ages	Aortic aneurysm	Number	2019	143.9822188	255.6797283	81.47932789	113.95%
DALYs	Suriname	Male	Age-standardized	Aortic aneurysm	Rate	1990	75.57711667	85.93288935	65.23882801	
DALYs	Suriname	Male	Age-standardized	Aortic aneurysm	Rate	2019	75.50268191	92.9463548	61.35052157	-0.10%
DALYs	Suriname	Male	All Ages	Aortic aneurysm	Number	1990	93.82702797	107.2944482	80.66481665	
DALYs	Suriname	Male	All Ages	Aortic aneurysm	Number	2019	204.9968994	252.9197908	165.3573427	118.48%
Deaths	Suriname	Male	Age-standardized	Aortic aneurysm	Rate	1990	3.895249015	4.40824316	3.347063516	
Deaths	Suriname	Male	Age-standardized	Aortic aneurysm	Rate	2019	3.859751	4.729791335	3.158718578	-0.91%
Deaths	Suriname	Male	All Ages	Aortic aneurysm	Number	1990	4.263764835	4.818878858	3.694750465	
Deaths	Suriname	Male	All Ages	Aortic aneurysm	Number	2019	9.529397929	11.7268908	7.802182886	123.50%
DALYs	Sweden	Male	Age-standardized	Aortic aneurysm	Rate	1990	207.4806063	217.54388	197.2826687	
DALYs	Sweden	Male	Age-standardized	Aortic aneurysm	Rate	2019	111.2652648	120.5064217	102.4944692	-46.37%
DALYs	Sweden	Male	All Ages	Aortic aneurysm	Number	1990	13658.31694	14379.72316	12965.32053	
DALYs	Sweden	Male	All Ages	Aortic aneurysm	Number	2019	10751.37049	11710.74121	9861.930346	-21.28%
Deaths	Sweden	Male	Age-standardized	Aortic aneurysm	Rate	1990	11.07672667	11.67540731	10.44376181	
Deaths	Sweden	Male	Age-standardized	Aortic aneurysm	Rate	2019	6.562356125	7.150002712	5.938739575	-40.76%
Deaths	Sweden	Male	All Ages	Aortic aneurysm	Number	1990	747.4629747	791.212271	705.4141177	
Deaths	Sweden	Male	All Ages	Aortic aneurysm	Number	2019	686.5344114	751.1953551	616.5460221	-8.15%
DALYs	Switzerland	Male	Age-standardized	Aortic aneurysm	Rate	1990	155.8131746	184.3769449	127.7626272	
DALYs	Switzerland	Male	Age-standardized	Aortic aneurysm	Rate	2019	75.69455662	85.00062809	67.22259339	-51.42%
DALYs	Switzerland	Male	All Ages	Aortic aneurysm	Number	1990	6760.663787	8009.571537	5530.991035	
DALYs	Switzerland	Male	All Ages	Aortic aneurysm	Number	2019	5891.619315	6631.645258	5197.354881	-12.85%
Deaths	Switzerland	Male	Age-standardized	Aortic aneurysm	Rate	1990	8.331878252	9.899785281	6.89554966	
Deaths	Switzerland	Male	Age-standardized	Aortic aneurysm	Rate	2019	4.471925585	5.030941965	3.925896822	-46.33%
Deaths	Switzerland	Male	All Ages	Aortic aneurysm	Number	1990	358.3394409	426.1745566	294.9843832	
Deaths	Switzerland	Male	All Ages	Aortic aneurysm	Number	2019	369.4477013	416.5225908	323.2420534	3.10%
DALYs	Syrian Arab Republic	Male	Age-standardized	Aortic aneurysm	Rate	1990	30.88956537	45.43807467	19.61058923	
DALYs	Syrian Arab Republic	Male	Age-standardized	Aortic aneurysm	Rate	2019	31.36771256	42.12619878	23.55064013	1.55%
DALYs	Syrian Arab Republic	Male	All Ages	Aortic aneurysm	Number	1990	960.4424594	1442.095057	599.7808548	
DALYs	Syrian Arab Republic	Male	All Ages	Aortic aneurysm	Number	2019	1942.191149	2675.156191	1435.414283	102.22%
Deaths	Syrian Arab Republic	Male	Age-standardized	Aortic aneurysm	Rate	1990	1.426235416	2.01900204	0.908743042	
Deaths	Syrian Arab Republic	Male	Age-standardized	Aortic aneurysm	Rate	2019	1.582119512	2.083363389	1.216943527	10.93%
Deaths	Syrian Arab Republic	Male	All Ages	Aortic aneurysm	Number	1990	36.22934231	52.63255986	23.17390952	
Deaths	Syrian Arab Republic	Male	All Ages	Aortic aneurysm	Number	2019	83.40044434	111.7546815	62.60322489	130.20%
DALYs	Taiwan (Province of China)	Male	Age-standardized	Aortic aneurysm	Rate	1990	38.54925806	41.16665143	35.99687621	
DALYs	Taiwan (Province of China)	Male	Age-standardized	Aortic aneurysm	Rate	2019	91.60053388	118.2941331	70.19645567	137.62%
DALYs	Taiwan (Province of China)	Male	All Ages	Aortic aneurysm	Number	1990	3371.740627	3603.099172	3137.165129	
DALYs	Taiwan (Province of China)	Male	All Ages	Aortic aneurysm	Number	2019	16293.14677	21052.48684	12441.46304	383.23%
Deaths	Taiwan (Province of China)	Male	Age-standardized	Aortic aneurysm	Rate	1990	1.784390188	1.926786186	1.653418079	
Deaths	Taiwan (Province of China)	Male	Age-standardized	Aortic aneurysm	Rate	2019	4.093049584	5.190750534	3.184228281	129.38%
Deaths	Taiwan (Province of China)	Male	All Ages	Aortic aneurysm	Number	1990	131.3343	140.7819704	122.3157499	
Deaths	Taiwan (Province of China)	Male	All Ages	Aortic aneurysm	Number	2019	738.2387695	938.9562047	575.5878026	462.11%
DALYs	Tajikistan	Male	Age-standardized	Aortic aneurysm	Rate	1990	40.27394854	55.50766242	30.65759065	
DALYs	Tajikistan	Male	Age-standardized	Aortic aneurysm	Rate	2019	61.91177668	76.23088014	49.80945405	53.73%
DALYs	Tajikistan	Male	All Ages	Aortic aneurysm	Number	1990	482.9574646	640.0411213	372.8132598	
DALYs	Tajikistan	Male	All Ages	Aortic aneurysm	Number	2019	1196.936657	1516.25811	938.3880026	147.83%
Deaths	Tajikistan	Male	Age-standardized	Aortic aneurysm	Rate	1990	2.140692935	3.250429321	1.573138507	
Deaths	Tajikistan	Male	Age-standardized	Aortic aneurysm	Rate	2019	3.819821513	4.636074151	3.134096116	78.44%
Deaths	Tajikistan	Male	All Ages	Aortic aneurysm	Number	1990	23.34190218	34.51529079	17.27372845	
Deaths	Tajikistan	Male	All Ages	Aortic aneurysm	Number	2019	52.60110388	65.1651767	42.2051814	125.35%
DALYs	Thailand	Male	Age-standardized	Aortic aneurysm	Rate	1990	50.43553598	65.07961609	38.48632156	
DALYs	Thailand	Male	Age-standardized	Aortic aneurysm	Rate	2019	48.53101864	63.58417826	35.57595445	-3.78%
DALYs	Thailand	Male	All Ages	Aortic aneurysm	Number	1990	7860.500271	10271.92746	6040.916771	
DALYs	Thailand	Male	All Ages	Aortic aneurysm	Number	2019	21308.56085	28175.39255	15483.41586	171.08%
Deaths	Thailand	Male	Age-standardized	Aortic aneurysm	Rate	1990	2.974578145	3.855049634	2.250945226	
Deaths	Thailand	Male	Age-standardized	Aortic aneurysm	Rate	2019	2.758314127	3.62270665	2.034180837	-7.27%
Deaths	Thailand	Male	All Ages	Aortic aneurysm	Number	1990	350.783421	453.7534555	266.8753949	
Deaths	Thailand	Male	All Ages	Aortic aneurysm	Number	2019	1151.177998	1507.662479	848.0075575	228.17%
DALYs	Timor-Leste	Male	Age-standardized	Aortic aneurysm	Rate	1990	18.88589494	31.3261796	9.114319283	
DALYs	Timor-Leste	Male	Age-standardized	Aortic aneurysm	Rate	2019	33.32699825	52.27021665	19.8281335	76.47%
DALYs	Timor-Leste	Male	All Ages	Aortic aneurysm	Number	1990	26.16404649	45.20871511	12.4159335	
DALYs	Timor-Leste	Male	All Ages	Aortic aneurysm	Number	2019	131.8774344	209.6832602	75.63063417	404.04%
Deaths	Timor-Leste	Male	Age-standardized	Aortic aneurysm	Rate	1990	1.045336295	1.735386631	0.499084732	
Deaths	Timor-Leste	Male	Age-standardized	Aortic aneurysm	Rate	2019	1.851243232	2.854766954	1.151315117	77.10%
Deaths	Timor-Leste	Male	All Ages	Aortic aneurysm	Number	1990	1.035140935	1.7306534	0.499162915	
Deaths	Timor-Leste	Male	All Ages	Aortic aneurysm	Number	2019	6.122303225	9.468348598	3.74152233	491.45%
DALYs	Togo	Male	Age-standardized	Aortic aneurysm	Rate	1990	48.59890598	68.77537673	31.05759521	
DALYs	Togo	Male	Age-standardized	Aortic aneurysm	Rate	2019	57.1438447	79.35878231	42.81086356	17.58%
DALYs	Togo	Male	All Ages	Aortic aneurysm	Number	1990	306.699984	435.7832099	195.629463	
DALYs	Togo	Male	All Ages	Aortic aneurysm	Number	2019	950.5085939	1318.885019	679.4882946	209.91%
Deaths	Togo	Male	Age-standardized	Aortic aneurysm	Rate	1990	2.363436455	3.30179904	1.507587759	
Deaths	Togo	Male	Age-standardized	Aortic aneurysm	Rate	2019	2.809289654	3.797361547	2.147628253	18.86%
Deaths	Togo	Male	All Ages	Aortic aneurysm	Number	1990	11.9034427	16.87155595	7.575798474	
Deaths	Togo	Male	All Ages	Aortic aneurysm	Number	2019	34.95060028	48.6108564	26.05360023	193.62%
DALYs	Tokelau	Male	Age-standardized	Aortic aneurysm	Rate	1990	77.822623	114.4949843	53.60992096	
DALYs	Tokelau	Male	Age-standardized	Aortic aneurysm	Rate	2019	65.94723896	84.63187453	51.49947378	-15.26%
DALYs	Tokelau	Male	All Ages	Aortic aneurysm	Number	1990	0.46991902	0.689147854	0.32393807	
DALYs	Tokelau	Male	All Ages	Aortic aneurysm	Number	2019	0.436441757	0.562452647	0.337314425	-7.12%
Deaths	Tokelau	Male	Age-standardized	Aortic aneurysm	Rate	1990	3.67150122	5.299214088	2.581682165	
Deaths	Tokelau	Male	Age-standardized	Aortic aneurysm	Rate	2019	3.121209148	3.825224035	2.553670789	-14.99%
Deaths	Tokelau	Male	All Ages	Aortic aneurysm	Number	1990	0.021860459	0.031704913	0.015220904	
Deaths	Tokelau	Male	All Ages	Aortic aneurysm	Number	2019	0.019563193	0.024047357	0.015981109	-10.51%
DALYs	Tonga	Male	Age-standardized	Aortic aneurysm	Rate	1990	75.54471136	95.83768588	57.61851567	
DALYs	Tonga	Male	Age-standardized	Aortic aneurysm	Rate	2019	86.68593525	110.3819459	67.82713091	14.75%
DALYs	Tonga	Male	All Ages	Aortic aneurysm	Number	1990	20.64683425	26.3859627	15.60118768	
DALYs	Tonga	Male	All Ages	Aortic aneurysm	Number	2019	32.01587381	41.19329967	24.78264743	55.06%
Deaths	Tonga	Male	Age-standardized	Aortic aneurysm	Rate	1990	3.653468578	4.572024573	2.866070798	
Deaths	Tonga	Male	Age-standardized	Aortic aneurysm	Rate	2019	4.158952383	5.246114725	3.322271722	13.84%
Deaths	Tonga	Male	All Ages	Aortic aneurysm	Number	1990	0.852835851	1.082763201	0.659315392	
Deaths	Tonga	Male	All Ages	Aortic aneurysm	Number	2019	1.405041995	1.7760636	1.115781709	64.75%
DALYs	Trinidad and Tobago	Male	Age-standardized	Aortic aneurysm	Rate	1990	134.3871189	154.4665899	117.6891891	
DALYs	Trinidad and Tobago	Male	Age-standardized	Aortic aneurysm	Rate	2019	125.8262315	167.5704489	91.59775353	-6.37%
DALYs	Trinidad and Tobago	Male	All Ages	Aortic aneurysm	Number	1990	546.6145067	627.1324476	478.7271032	
DALYs	Trinidad and Tobago	Male	All Ages	Aortic aneurysm	Number	2019	1107.751062	1482.505921	802.464823	102.66%
Deaths	Trinidad and Tobago	Male	Age-standardized	Aortic aneurysm	Rate	1990	6.779507221	7.902005371	5.868495053	
Deaths	Trinidad and Tobago	Male	Age-standardized	Aortic aneurysm	Rate	2019	6.240448814	8.137704569	4.578982712	-7.95%
Deaths	Trinidad and Tobago	Male	All Ages	Aortic aneurysm	Number	1990	24.31905468	28.22616495	21.05810332	
Deaths	Trinidad and Tobago	Male	All Ages	Aortic aneurysm	Number	2019	51.38614968	68.04919498	37.57425199	111.30%
DALYs	Tunisia	Male	Age-standardized	Aortic aneurysm	Rate	1990	28.17530285	38.31209092	19.45287544	
DALYs	Tunisia	Male	Age-standardized	Aortic aneurysm	Rate	2019	32.22931842	44.85332475	22.34151784	14.39%

DALYs	Tunisia	Male	All Ages	Aortic aneurysm	Number	1990	734.6082308	1001.099481	505.2378299	
DALYs	Tunisia	Male	All Ages	Aortic aneurysm	Number	2019	1930.20395	2709.755848	1320.458919	162.75%
Deaths	Tunisia	Male	Age-standardized	Aortic aneurysm	Rate	1990	1.481474356	1.990217114	1.032860272	
Deaths	Tunisia	Male	Age-standardized	Aortic aneurysm	Rate	2019	1.717114924	2.29798325	1.234967521	15.91%
Deaths	Tunisia	Male	All Ages	Aortic aneurysm	Number	1990	32.0966508	43.2907836	22.20355984	
Deaths	Tunisia	Male	All Ages	Aortic aneurysm	Number	2019	93.10571725	125.4577874	65.86194492	190.08%
DALYs	Turkey	Male	Age-standardized	Aortic aneurysm	Rate	1990	119.7599491	172.8379378	83.3289577	
DALYs	Turkey	Male	Age-standardized	Aortic aneurysm	Rate	2019	74.50482761	95.13026815	57.53809967	-37.79%
DALYs	Turkey	Male	All Ages	Aortic aneurysm	Number	1990	23623.88532	34604.9456	16191.35792	
DALYs	Turkey	Male	All Ages	Aortic aneurysm	Number	2019	32642.57639	41779.33411	25059.59376	38.18%
Deaths	Turkey	Male	Age-standardized	Aortic aneurysm	Rate	1990	4.659663486	6.491868576	3.228235018	
Deaths	Turkey	Male	Age-standardized	Aortic aneurysm	Rate	2019	3.142551844	3.950796714	2.455596059	-32.56%
Deaths	Turkey	Male	All Ages	Aortic aneurysm	Number	1990	803.3523431	1140.208338	560.2188356	
Deaths	Turkey	Male	All Ages	Aortic aneurysm	Number	2019	1276.855839	1619.047558	994.5863998	58.94%
DALYs	Turkmenistan	Male	Age-standardized	Aortic aneurysm	Rate	1990	34.51218407	43.71677926	29.2543473	
DALYs	Turkmenistan	Male	Age-standardized	Aortic aneurysm	Rate	2019	82.80926846	105.5953541	64.88704603	139.94%
DALYs	Turkmenistan	Male	All Ages	Aortic aneurysm	Number	1990	287.794381	363.7608032	242.7962972	
DALYs	Turkmenistan	Male	All Ages	Aortic aneurysm	Number	2019	1555.036867	1988.779111	1203.367929	440.33%
Deaths	Turkmenistan	Male	Age-standardized	Aortic aneurysm	Rate	1990	1.660053331	2.095335326	1.408372428	
Deaths	Turkmenistan	Male	Age-standardized	Aortic aneurysm	Rate	2019	3.755588694	4.673675938	2.979386082	126.23%
Deaths	Turkmenistan	Male	All Ages	Aortic aneurysm	Number	1990	11.06734411	13.90676435	9.396677822	
Deaths	Turkmenistan	Male	All Ages	Aortic aneurysm	Number	2019	56.96560312	72.1598466	44.557222	414.72%
DALYs	Tuvalu	Male	Age-standardized	Aortic aneurysm	Rate	1990	124.5599214	195.3388211	72.11227292	
DALYs	Tuvalu	Male	Age-standardized	Aortic aneurysm	Rate	2019	99.79024807	135.4547619	70.55810563	-19.89%
DALYs	Tuvalu	Male	All Ages	Aortic aneurysm	Number	1990	3.879969098	6.206193054	2.156931738	
DALYs	Tuvalu	Male	All Ages	Aortic aneurysm	Number	2019	5.022516523	6.873819697	3.465939584	29.45%
Deaths	Tuvalu	Male	Age-standardized	Aortic aneurysm	Rate	1990	5.534698619	8.463937695	3.360395693	
Deaths	Tuvalu	Male	Age-standardized	Aortic aneurysm	Rate	2019	4.431132094	5.941596429	3.264552112	-19.94%
Deaths	Tuvalu	Male	All Ages	Aortic aneurysm	Number	1990	0.141915792	0.224773905	0.082438584	
Deaths	Tuvalu	Male	All Ages	Aortic aneurysm	Number	2019	0.190836719	0.25630442	0.138069394	34.47%
DALYs	Uganda	Male	Age-standardized	Aortic aneurysm	Rate	1990	69.06325124	118.8986262	27.23600165	
DALYs	Uganda	Male	Age-standardized	Aortic aneurysm	Rate	2019	57.49111139	86.71983965	34.7500235	-16.76%
DALYs	Uganda	Male	All Ages	Aortic aneurysm	Number	1990	2338.340903	4103.731497	917.5030482	
DALYs	Uganda	Male	All Ages	Aortic aneurysm	Number	2019	4124.748844	6333.277102	2427.08102	76.40%
Deaths	Uganda	Male	Age-standardized	Aortic aneurysm	Rate	1990	3.213212515	5.422239983	1.232974746	
Deaths	Uganda	Male	Age-standardized	Aortic aneurysm	Rate	2019	2.713651599	3.971725286	1.629289163	-15.55%
Deaths	Uganda	Male	All Ages	Aortic aneurysm	Number	1990	90.73471059	154.7727741	35.66055218	
Deaths	Uganda	Male	All Ages	Aortic aneurysm	Number	2019	146.4206876	220.6125108	88.14388318	61.37%
DALYs	Ukraine	Male	Age-standardized	Aortic aneurysm	Rate	1990	80.57847739	96.53197764	67.11405437	
DALYs	Ukraine	Male	Age-standardized	Aortic aneurysm	Rate	2019	134.6195416	166.3812342	107.6484083	67.07%
DALYs	Ukraine	Male	All Ages	Aortic aneurysm	Number	1990	21565.89872	25999.66792	17738.58661	
DALYs	Ukraine	Male	All Ages	Aortic aneurysm	Number	2019	38265.48837	47372.10826	30420.16852	77.44%
Deaths	Ukraine	Male	Age-standardized	Aortic aneurysm	Rate	1990	3.503302264	4.104569392	2.943821046	
Deaths	Ukraine	Male	Age-standardized	Aortic aneurysm	Rate	2019	5.167583572	6.306110652	4.183826176	47.51%
Deaths	Ukraine	Male	All Ages	Aortic aneurysm	Number	1990	854.2496331	1009.669025	713.5185503	
Deaths	Ukraine	Male	All Ages	Aortic aneurysm	Number	2019	1452.214853	1779.390938	1164.800669	70.00%
DALYs	United Arab Emirates	Male	Age-standardized	Aortic aneurysm	Rate	1990	60.44162143	87.63975177	37.44656914	
DALYs	United Arab Emirates	Male	Age-standardized	Aortic aneurysm	Rate	2019	56.86164352	95.68719396	28.72767008	-5.92%
DALYs	United Arab Emirates	Male	All Ages	Aortic aneurysm	Number	1990	273.6576235	408.8415136	180.1676258	
DALYs	United Arab Emirates	Male	All Ages	Aortic aneurysm	Number	2019	2759.721778	5060.62115	1264.288932	908.46%
Deaths	United Arab Emirates	Male	Age-standardized	Aortic aneurysm	Rate	1990	2.898249811	4.124647065	1.74543254	
Deaths	United Arab Emirates	Male	Age-standardized	Aortic aneurysm	Rate	2019	2.680906589	4.512692595	1.382849015	-7.50%
Deaths	United Arab Emirates	Male	All Ages	Aortic aneurysm	Number	1990	7.358222199	10.81561976	4.884829086	
Deaths	United Arab Emirates	Male	All Ages	Aortic aneurysm	Number	2019	71.99578426	127.9446801	34.06140682	878.44%
DALYs	United Kingdom	Male	Age-standardized	Aortic aneurysm	Rate	1990	273.1530225	279.2947191	265.5163915	
DALYs	United Kingdom	Male	Age-standardized	Aortic aneurysm	Rate	2019	129.9038881	135.5584116	121.9473395	-52.44%
DALYs	United Kingdom	Male	All Ages	Aortic aneurysm	Number	1990	105728.0304	108030.0475	102900.0219	
DALYs	United Kingdom	Male	All Ages	Aortic aneurysm	Number	2019	75632.43059	79035.15895	70789.726	-28.47%
Deaths	United Kingdom	Male	Age-standardized	Aortic aneurysm	Rate	1990	15.78756851	16.21956227	15.14318792	
Deaths	United Kingdom	Male	Age-standardized	Aortic aneurysm	Rate	2019	8.223177955	8.636698448	7.535058396	-47.91%
Deaths	United Kingdom	Male	All Ages	Aortic aneurysm	Number	1990	5851.007481	5990.014684	5650.470457	
Deaths	United Kingdom	Male	All Ages	Aortic aneurysm	Number	2019	4992.755286	5242.974148	4570.871439	-14.67%
DALYs	United Republic of Tanzania	Male	Age-standardized	Aortic aneurysm	Rate	1990	90.43031084	135.9783918	48.65683263	
DALYs	United Republic of Tanzania	Male	Age-standardized	Aortic aneurysm	Rate	2019	66.49037204	116.4239508	36.70587294	-26.47%
DALYs	United Republic of Tanzania	Male	All Ages	Aortic aneurysm	Number	1990	5105.567939	7732.880592	2773.242021	
DALYs	United Republic of Tanzania	Male	All Ages	Aortic aneurysm	Number	2019	8314.918374	14452.65483	4542.795805	62.86%
Deaths	United Republic of Tanzania	Male	Age-standardized	Aortic aneurysm	Rate	1990	4.263245136	6.341542309	2.284719937	
Deaths	United Republic of Tanzania	Male	Age-standardized	Aortic aneurysm	Rate	2019	3.248027735	5.778200886	1.797246378	-23.81%
Deaths	United Republic of Tanzania	Male	All Ages	Aortic aneurysm	Number	1990	199.7811323	299.4922636	107.9088118	
Deaths	United Republic of Tanzania	Male	All Ages	Aortic aneurysm	Number	2019	329.4992953	579.5981708	182.2743764	64.93%
DALYs	United States of America	Male	Age-standardized	Aortic aneurysm	Rate	1990	158.2610857	165.0762916	151.7354273	
DALYs	United States of America	Male	Age-standardized	Aortic aneurysm	Rate	2019	66.24784966	69.87634003	61.33059423	-58.14%
DALYs	United States of America	Male	All Ages	Aortic aneurysm	Number	1990	216141.242	225435.9357	207521.3538	
DALYs	United States of America	Male	All Ages	Aortic aneurysm	Number	2019	156904.9572	165576.298	145516.0398	-27.41%
Deaths	United States of America	Male	Age-standardized	Aortic aneurysm	Rate	1990	8.312513348	8.698550092	7.889778591	
Deaths	United States of America	Male	Age-standardized	Aortic aneurysm	Rate	2019	3.225944983	3.43396866	2.961874294	-61.19%
Deaths	United States of America	Male	All Ages	Aortic aneurysm	Number	1990	11059.27932	11555.88809	10541.01492	
Deaths	United States of America	Male	All Ages	Aortic aneurysm	Number	2019	8003.342368	8526.039876	7330.05297	-27.63%
DALYs	United States Virgin Islands	Male	Age-standardized	Aortic aneurysm	Rate	1990	91.02117662	110.373199	73.02063186	
DALYs	United States Virgin Islands	Male	Age-standardized	Aortic aneurysm	Rate	2019	109.2882027	132.0680033	90.08768821	20.07%
DALYs	United States Virgin Islands	Male	All Ages	Aortic aneurysm	Number	1990	35.22988414	42.75531214	28.10582042	
DALYs	United States Virgin Islands	Male	All Ages	Aortic aneurysm	Number	2019	90.99676407	109.8227181	74.65964051	158.29%
Deaths	United States Virgin Islands	Male	Age-standardized	Aortic aneurysm	Rate	1990	4.74539486	5.741327896	3.821546263	
Deaths	United States Virgin Islands	Male	Age-standardized	Aortic aneurysm	Rate	2019	5.730198828	6.822708577	4.777733459	20.75%
Deaths	United States Virgin Islands	Male	All Ages	Aortic aneurysm	Number	1990	1.537466856	1.86989766	1.227870265	
Deaths	United States Virgin Islands	Male	All Ages	Aortic aneurysm	Number	2019	4.513870953	5.432653523	3.753641272	193.59%
DALYs	Uruguay	Male	Age-standardized	Aortic aneurysm	Rate	1990	166.5084245	183.5839733	149.6920995	
DALYs	Uruguay	Male	Age-standardized	Aortic aneurysm	Rate	2019	156.8148623	172.3158383	142.8180025	-5.82%
DALYs	Uruguay	Male	All Ages	Aortic aneurysm	Number	1990	2857.756806	3161.838811	2564.803314	
DALYs	Uruguay	Male	All Ages	Aortic aneurysm	Number	2019	3437.225237	3796.939996	3125.765123	20.28%
Deaths	Uruguay	Male	Age-standardized	Aortic aneurysm	Rate	1990	7.681865696	8.515059282	6.901399815	
Deaths	Uruguay	Male	Age-standardized	Aortic aneurysm	Rate	2019	7.519297414	8.281471396	6.773360393	-2.12%
Deaths	Uruguay	Male	All Ages	Aortic aneurysm	Number	1990	129.1895634	143.7101271	115.7090067	
Deaths	Uruguay	Male	All Ages	Aortic aneurysm	Number	2019	170.9369559	188.571129	153.9699184	32.31%
DALYs	Uzbekistan	Male	Age-standardized	Aortic aneurysm	Rate	1990	16.38730478	26.54408918	12.03151056	
DALYs	Uzbekistan	Male	Age-standardized	Aortic aneurysm	Rate	2019	47.69448188	56.51674637	39.81088673	191.05%
DALYs	Uzbekistan	Male	All Ages	Aortic aneurysm	Number	1990	779.6289601	1207.269153	591.6190184	
DALYs	Uzbekistan	Male	All Ages	Aortic aneurysm	Number	2019	4277.837465	5143.894028	3484.31503	448.70%
Deaths	Uzbekistan	Male	Age-standardized	Aortic aneurysm	Rate	1990	0.86057791	1.490427594	0.598224878	
Deaths	Uzbekistan	Male	Age-standardized	Aortic aneurysm	Rate	2019	2.541757525	2.963534241	2.148723932	195.35%
Deaths	Uzbekistan	Male	All Ages	Aortic aneurysm	Number	1990	34.59433237	57.63898555	24.79048759	

Deaths	Uzbekistan	Male	All Ages	Aortic aneurysm	Number	2019	153.6531134	185.2919134	126.0530129	344.16%
DALYs	Vanuatu	Male	Age-standardized	Aortic aneurysm	Rate	1990	99.58385279	164.8247681	52.79179394	
DALYs	Vanuatu	Male	Age-standardized	Aortic aneurysm	Rate	2019	103.5802235	158.1094242	62.43639739	4.01%
DALYs	Vanuatu	Male	All Ages	Aortic aneurysm	Number	1990	39.90297999	67.22787048	20.59185246	
DALYs	Vanuatu	Male	All Ages	Aortic aneurysm	Number	2019	102.1164394	157.8445924	59.25079409	155.91%
Deaths	Vanuatu	Male	Age-standardized	Aortic aneurysm	Rate	1990	4.439998592	7.116439926	2.439693289	
Deaths	Vanuatu	Male	Age-standardized	Aortic aneurysm	Rate	2019	4.481860108	6.640699432	2.844544446	0.94%
Deaths	Vanuatu	Male	All Ages	Aortic aneurysm	Number	1990	1.420430543	2.33515355	0.758829033	
Deaths	Vanuatu	Male	All Ages	Aortic aneurysm	Number	2019	3.653503543	5.51405443	2.257608284	157.21%
DALYs	Venezuela (Bolivarian Republic of)	Male	Age-standardized	Aortic aneurysm	Rate	1990	64.12892863	72.02882481	57.32837886	
DALYs	Venezuela (Bolivarian Republic of)	Male	Age-standardized	Aortic aneurysm	Rate	2019	69.3190834	91.48272179	51.55021482	8.09%
DALYs	Venezuela (Bolivarian Republic of)	Male	All Ages	Aortic aneurysm	Number	1990	2998.039631	3348.022502	2693.638315	
DALYs	Venezuela (Bolivarian Republic of)	Male	All Ages	Aortic aneurysm	Number	2019	9291.974318	12346.77319	6854.909193	209.94%
Deaths	Venezuela (Bolivarian Republic of)	Male	Age-standardized	Aortic aneurysm	Rate	1990	3.135714868	3.53617718	2.763525035	
Deaths	Venezuela (Bolivarian Republic of)	Male	Age-standardized	Aortic aneurysm	Rate	2019	3.566460347	4.645917269	2.668311574	13.74%
Deaths	Venezuela (Bolivarian Republic of)	Male	All Ages	Aortic aneurysm	Number	1990	128.9226107	144.8086856	114.6574196	
Deaths	Venezuela (Bolivarian Republic of)	Male	All Ages	Aortic aneurysm	Number	2019	439.7713216	571.3666293	328.0102899	241.11%
DALYs	Viet Nam	Male	Age-standardized	Aortic aneurysm	Rate	1990	42.30688631	59.10552304	29.61719137	
DALYs	Viet Nam	Male	Age-standardized	Aortic aneurysm	Rate	2019	54.42343583	75.30211062	36.02006349	28.64%
DALYs	Viet Nam	Male	All Ages	Aortic aneurysm	Number	1990	7149.541185	10167.57539	4974.16438	
DALYs	Viet Nam	Male	All Ages	Aortic aneurysm	Number	2019	20600.28511	29204.96409	13558.94901	188.13%
Deaths	Viet Nam	Male	Age-standardized	Aortic aneurysm	Rate	1990	2.231308382	3.116262807	1.574845878	
Deaths	Viet Nam	Male	Age-standardized	Aortic aneurysm	Rate	2019	3.000163861	4.181270176	1.97321626	34.46%
Deaths	Viet Nam	Male	All Ages	Aortic aneurysm	Number	1990	311.9413001	436.0488677	218.2618306	
Deaths	Viet Nam	Male	All Ages	Aortic aneurysm	Number	2019	922.3831771	1280.509235	613.6044415	195.69%
DALYs	Yemen	Male	Age-standardized	Aortic aneurysm	Rate	1990	34.44660272	63.92426235	14.39169522	
DALYs	Yemen	Male	Age-standardized	Aortic aneurysm	Rate	2019	35.59666543	54.73467476	21.44705297	3.34%
DALYs	Yemen	Male	All Ages	Aortic aneurysm	Number	1990	883.5103201	1699.439048	358.7480186	
DALYs	Yemen	Male	All Ages	Aortic aneurysm	Number	2019	2575.216159	4062.811592	1514.224195	191.48%
Deaths	Yemen	Male	Age-standardized	Aortic aneurysm	Rate	1990	1.608476968	2.898073575	0.691740148	
Deaths	Yemen	Male	Age-standardized	Aortic aneurysm	Rate	2019	1.713824103	2.555678351	1.063762191	6.55%
Deaths	Yemen	Male	All Ages	Aortic aneurysm	Number	1990	31.35624238	58.30939025	13.07872648	
Deaths	Yemen	Male	All Ages	Aortic aneurysm	Number	2019	97.82794302	150.0905352	59.45703581	211.99%
DALYs	Zambia	Male	Age-standardized	Aortic aneurysm	Rate	1990	69.71956224	108.609097	40.48627662	
DALYs	Zambia	Male	Age-standardized	Aortic aneurysm	Rate	2019	92.00677396	127.3082421	65.84743865	31.97%
DALYs	Zambia	Male	All Ages	Aortic aneurysm	Number	1990	1091.73182	1709.73827	632.1413862	
DALYs	Zambia	Male	All Ages	Aortic aneurysm	Number	2019	3231.242987	4574.5158	2245.167393	195.97%
Deaths	Zambia	Male	Age-standardized	Aortic aneurysm	Rate	1990	3.381431115	5.22892363	1.952590858	
Deaths	Zambia	Male	Age-standardized	Aortic aneurysm	Rate	2019	4.380629187	5.922007574	3.216647107	29.55%
Deaths	Zambia	Male	All Ages	Aortic aneurysm	Number	1990	43.47308385	67.43506073	25.08775662	
Deaths	Zambia	Male	All Ages	Aortic aneurysm	Number	2019	119.3634531	165.0129728	85.66659469	174.57%
DALYs	Zimbabwe	Male	Age-standardized	Aortic aneurysm	Rate	1990	66.0077865	80.54766542	55.07433968	
DALYs	Zimbabwe	Male	Age-standardized	Aortic aneurysm	Rate	2019	88.03722493	112.872048	68.13540046	33.37%
DALYs	Zimbabwe	Male	All Ages	Aortic aneurysm	Number	1990	1384.39151	1711.930526	1136.243063	
DALYs	Zimbabwe	Male	All Ages	Aortic aneurysm	Number	2019	2988.287861	3930.383097	2252.217209	115.86%
Deaths	Zimbabwe	Male	Age-standardized	Aortic aneurysm	Rate	1990	3.096699259	3.741711663	2.600990871	
Deaths	Zimbabwe	Male	Age-standardized	Aortic aneurysm	Rate	2019	3.889117097	4.859410448	3.079014832	25.59%
Deaths	Zimbabwe	Male	All Ages	Aortic aneurysm	Number	1990	51.79462209	63.05916581	42.94536395	
Deaths	Zimbabwe	Male	All Ages	Aortic aneurysm	Number	2019	103.2543227	132.3757028	80.0088621	99.35%
