# Supplementary material for: Burden of Aortic Aneurysm and Its Attributable Risk Factors from 1990 to 2019: An Analysis of the Global Burden of Disease Study 2019
Source: Front Cardiovasc Med. 2022 May 31;9:901225. doi: 10.3389/fcvm.2022.901225 (PMC9197430; doi:10.3389/fcvm.2022.901225)
Supplement: Supplementary Table 5 — Global burden of aortic aneurysm in different age groups in 2019. DALY, disability-adjusted life year rate. SDI, socio-demographic index. [file Data_Sheet_5.PDF]

measure	location	sex	age	cause	metri	year	val	upper	lower	multiple to 65-69
Deaths	High SDI	Both	15 to 19	Aortic aneurysm	Rate	2019	0.031070831	0.034425	0.028301	0.003335215
Deaths	High SDI	Both	20 to 24	Aortic aneurysm	Rate	2019	0.063477619	0.069512	0.058238	0.006813835
Deaths	High SDI	Both	25 to 29	Aortic aneurysm	Rate	2019	0.12220025	0.133974	0.111946	0.013117258
Deaths	High SDI	Both	30 to 34	Aortic aneurysm	Rate	2019	0.219483858	0.240142	0.199694	0.023559906
Deaths	High SDI	Both	35 to 39	Aortic aneurysm	Rate	2019	0.393011496	0.425529	0.365346	0.042186764
Deaths	High SDI	Both	40 to 44	Aortic aneurysm	Rate	2019	0.686407653	0.7327	0.643342	0.073680588
Deaths	High SDI	Both	45 to 49	Aortic aneurysm	Rate	2019	1.127095805	1.190521	1.062777	0.120985075
Deaths	High SDI	Both	50 to 54	Aortic aneurysm	Rate	2019	1.806334872	1.900755	1.70978	0.19389617
Deaths	High SDI	Both	55 to 59	Aortic aneurysm	Rate	2019	3.048253314	3.201	2.885785	0.327206572
Deaths	High SDI	Both	60 to 64	Aortic aneurysm	Rate	2019	5.29918687	5.550619	5.03232	0.568826995
Deaths	High SDI	Both	65 to 69	Aortic aneurysm	Rate	2019	9.31599048	9.747574	8.838899	1
Deaths	High SDI	Both	70 to 74	Aortic aneurysm	Rate	2019	16.93051737	17.78256	15.92633	1.817360956
Deaths	High SDI	Both	75 to 79	Aortic aneurysm	Rate	2019	28.72986003	30.27984	26.46186	3.083929733
Deaths	High SDI	Both	80 to 84	Aortic aneurysm	Rate	2019	49.06344105	53.137	41.79458	5.266583425
Deaths	High SDI	Both	85 to 89	Aortic aneurysm	Rate	2019	78.89075692	87.16395	63.46057	8.46831661
Deaths	High SDI	Both	90 to 94	Aortic aneurysm	Rate	2019	107.3594737	122.3393	80.03139	11.52421462
Deaths	High SDI	Both	95 plus	Aortic aneurysm	Rate	2019	137.8726202	161.2381	99.34967	14.79956645
Deaths	High-middle SDI	Both	15 to 19	Aortic aneurysm	Rate	2019	0.049493218	0.05591	0.044194	0.005497051
Deaths	High-middle SDI	Both	20 to 24	Aortic aneurysm	Rate	2019	0.085590836	0.097469	0.07628	0.009506296
Deaths	High-middle SDI	Both	25 to 29	Aortic aneurysm	Rate	2019	0.148406123	0.169033	0.132893	0.016482986
Deaths	High-middle SDI	Both	30 to 34	Aortic aneurysm	Rate	2019	0.248690186	0.283237	0.223722	0.027621212
Deaths	High-middle SDI	Both	35 to 39	Aortic aneurysm	Rate	2019	0.462263463	0.518282	0.420319	0.051342102
Deaths	High-middle SDI	Both	40 to 44	Aortic aneurysm	Rate	2019	0.777091101	0.855054	0.706998	0.086308986
Deaths	High-middle SDI	Both	45 to 49	Aortic aneurysm	Rate	2019	1.192277815	1.305112	1.090547	0.132422426
Deaths	High-middle SDI	Both	50 to 54	Aortic aneurysm	Rate	2019	1.871763673	2.03305	1.724901	0.207890715
Deaths	High-middle SDI	Both	55 to 59	Aortic aneurysm	Rate	2019	3.314954066	3.585272	3.072089	0.368181187
Deaths	High-middle SDI	Both	60 to 64	Aortic aneurysm	Rate	2019	5.599840596	6.028731	5.196843	0.621956116
Deaths	High-middle SDI	Both	65 to 69	Aortic aneurysm	Rate	2019	9.003594388	9.652142	8.401876	1
Deaths	High-middle SDI	Both	70 to 74	Aortic aneurysm	Rate	2019	13.29625103	14.18716	12.41833	1.476771438
Deaths	High-middle SDI	Both	75 to 79	Aortic aneurysm	Rate	2019	20.01393539	21.3599	18.43077	2.222882832
Deaths	High-middle SDI	Both	80 to 84	Aortic aneurysm	Rate	2019	28.22170353	30.43722	24.97778	3.134492994
Deaths	High-middle SDI	Both	85 to 89	Aortic aneurysm	Rate	2019	38.6903473	42.07038	33.20577	4.297211273
Deaths	High-middle SDI	Both	90 to 94	Aortic aneurysm	Rate	2019	48.71084433	54.01484	39.17749	5.410155349
Deaths	High-middle SDI	Both	95 plus	Aortic aneurysm	Rate	2019	58.07418216	65.99044	45.18895	6.450110884
Deaths	Global	Both	15 to 19	Aortic aneurysm	Rate	2019	0.037063009	0.042297	0.032498	0.005016114
Deaths	Global	Both	20 to 24	Aortic aneurysm	Rate	2019	0.062797667	0.070703	0.056738	0.008499047
Deaths	Global	Both	25 to 29	Aortic aneurysm	Rate	2019	0.113442368	0.127595	0.103057	0.01535331
Deaths	Global	Both	30 to 34	Aortic aneurysm	Rate	2019	0.187691965	0.209526	0.171933	0.025402263
Deaths	Global	Both	35 to 39	Aortic aneurysm	Rate	2019	0.341703452	0.37841	0.311549	0.046246204
Deaths	Global	Both	40 to 44	Aortic aneurysm	Rate	2019	0.549272085	0.600726	0.507096	0.074338579
Deaths	Global	Both	45 to 49	Aortic aneurysm	Rate	2019	0.869089632	0.94422	0.806732	0.117622741
Deaths	Global	Both	50 to 54	Aortic aneurysm	Rate	2019	1.398198444	1.506352	1.307704	0.189232419
Deaths	Global	Both	55 to 59	Aortic aneurysm	Rate	2019	2.537843832	2.713862	2.37605	0.343472222
Deaths	Global	Both	60 to 64	Aortic aneurysm	Rate	2019	4.363445532	4.651909	4.082078	0.590549471
Deaths	Global	Both	65 to 69	Aortic aneurysm	Rate	2019	7.388789166	7.830132	6.937228	1
Deaths	Global	Both	70 to 74	Aortic aneurysm	Rate	2019	12.15626241	12.81284	11.42242	1.645230651
Deaths	Global	Both	75 to 79	Aortic aneurysm	Rate	2019	19.8838722	20.91282	18.43723	2.691086693
Deaths	Global	Both	80 to 84	Aortic aneurysm	Rate	2019	31.81199005	34.01279	27.90369	4.30544022
Deaths	Global	Both	85 to 89	Aortic aneurysm	Rate	2019	49.50559229	54.05282	41.02228	6.700095399
Deaths	Global	Both	90 to 94	Aortic aneurysm	Rate	2019	71.14802666	79.90073	55.22546	9.629186199
Deaths	Global	Both	95 plus	Aortic aneurysm	Rate	2019	98.02247661	112.8553	72.26316	13.26637889
Deaths	Low SDI	Both	15 to 19	Aortic aneurysm	Rate	2019	0.033310829	0.046615	0.022401	0.004348714
Deaths	Low SDI	Both	20 to 24	Aortic aneurysm	Rate	2019	0.056833453	0.07745	0.038451	0.007419583
Deaths	Low SDI	Both	25 to 29	Aortic aneurysm	Rate	2019	0.112872707	0.15393	0.079317	0.014735483
Deaths	Low SDI	Both	30 to 34	Aortic aneurysm	Rate	2019	0.154270754	0.208658	0.108303	0.020139981
Deaths	Low SDI	Both	35 to 39	Aortic aneurysm	Rate	2019	0.301467944	0.409633	0.213311	0.039356511
Deaths	Low SDI	Both	40 to 44	Aortic aneurysm	Rate	2019	0.454720422	0.609496	0.321723	0.059363556
Deaths	Low SDI	Both	45 to 49	Aortic aneurysm	Rate	2019	0.794765472	1.050261	0.570691	0.103756292
Deaths	Low SDI	Both	50 to 54	Aortic aneurysm	Rate	2019	1.308648028	1.714353	0.947963	0.170843439
Deaths	Low SDI	Both	55 to 59	Aortic aneurysm	Rate	2019	2.599336493	3.335816	1.87703	0.339342265
Deaths	Low SDI	Both	60 to 64	Aortic aneurysm	Rate	2019	4.459140008	5.748899	3.240338	0.582138817
Deaths	Low SDI	Both	65 to 69	Aortic aneurysm	Rate	2019	7.659925569	9.790513	5.617863	1
Deaths	Low SDI	Both	70 to 74	Aortic aneurysm	Rate	2019	11.64154786	14.71994	8.585758	1.519799084
Deaths	Low SDI	Both	75 to 79	Aortic aneurysm	Rate	2019	18.95115054	23.4279	14.14903	2.474064581
Deaths	Low SDI	Both	80 to 84	Aortic aneurysm	Rate	2019	25.87567712	31.92761	19.22096	3.378058558
Deaths	Low SDI	Both	85 to 89	Aortic aneurysm	Rate	2019	31.76138313	39.09599	23.35777	4.146434955
Deaths	Low SDI	Both	90 to 94	Aortic aneurysm	Rate	2019	38.41164059	47.5741	27.7081	5.014623216

Deaths	Low SDI	Both	95 plus	Aortic aneurysm	Rate	2019	38.69729831	48.70585	27.605	5.051915709
Deaths	Low-middle SDI	Both	15 to 19	Aortic aneurysm	Rate	2019	0.031035243	0.03924	0.02499	0.004683109
Deaths	Low-middle SDI	Both	20 to 24	Aortic aneurysm	Rate	2019	0.053267687	0.0677	0.04397	0.008037907
Deaths	Low-middle SDI	Both	25 to 29	Aortic aneurysm	Rate	2019	0.100596033	0.123947	0.083026	0.015179587
Deaths	Low-middle SDI	Both	30 to 34	Aortic aneurysm	Rate	2019	0.169908867	0.212473	0.132865	0.02563865
Deaths	Low-middle SDI	Both	35 to 39	Aortic aneurysm	Rate	2019	0.311836592	0.389947	0.241512	0.047055045
Deaths	Low-middle SDI	Both	40 to 44	Aortic aneurysm	Rate	2019	0.477721838	0.58725	0.381298	0.072086544
Deaths	Low-middle SDI	Both	45 to 49	Aortic aneurysm	Rate	2019	0.790394399	0.962644	0.63976	0.119267734
Deaths	Low-middle SDI	Both	50 to 54	Aortic aneurysm	Rate	2019	1.300230832	1.577467	1.057578	0.196200258
Deaths	Low-middle SDI	Both	55 to 59	Aortic aneurysm	Rate	2019	2.392867642	2.923476	1.925998	0.361075308
Deaths	Low-middle SDI	Both	60 to 64	Aortic aneurysm	Rate	2019	3.959193392	4.787939	3.217053	0.597428352
Deaths	Low-middle SDI	Both	65 to 69	Aortic aneurysm	Rate	2019	6.627059757	8.090179	5.380766	1
Deaths	Low-middle SDI	Both	70 to 74	Aortic aneurysm	Rate	2019	10.14930309	12.05044	8.488386	1.531494126
Deaths	Low-middle SDI	Both	75 to 79	Aortic aneurysm	Rate	2019	15.76729255	18.58811	13.28355	2.379228969
Deaths	Low-middle SDI	Both	80 to 84	Aortic aneurysm	Rate	2019	23.91978344	27.96608	19.71712	3.609411159
Deaths	Low-middle SDI	Both	85 to 89	Aortic aneurysm	Rate	2019	30.79047898	35.57527	25.66848	4.646174942
Deaths	Low-middle SDI	Both	90 to 94	Aortic aneurysm	Rate	2019	39.76887232	46.69896	31.33292	6.000982906
Deaths	Low-middle SDI	Both	95 plus	Aortic aneurysm	Rate	2019	47.61682411	55.82547	36.16946	7.185211218
Deaths	Middle SDI	Both	15 to 19	Aortic aneurysm	Rate	2019	0.041507544	0.049738	0.034646	0.008137736
Deaths	Middle SDI	Both	20 to 24	Aortic aneurysm	Rate	2019	0.063142968	0.073265	0.054443	0.012379456
Deaths	Middle SDI	Both	25 to 29	Aortic aneurysm	Rate	2019	0.10083889	0.115871	0.088365	0.019769908
Deaths	Middle SDI	Both	30 to 34	Aortic aneurysm	Rate	2019	0.162520157	0.184082	0.144335	0.031862791
Deaths	Middle SDI	Both	35 to 39	Aortic aneurysm	Rate	2019	0.278771135	0.313347	0.248182	0.054654306
Deaths	Middle SDI	Both	40 to 44	Aortic aneurysm	Rate	2019	0.418988139	0.471681	0.371469	0.082144467
Deaths	Middle SDI	Both	45 to 49	Aortic aneurysm	Rate	2019	0.611974345	0.692724	0.541053	0.119980261
Deaths	Middle SDI	Both	50 to 54	Aortic aneurysm	Rate	2019	0.970508106	1.079916	0.86526	0.190272381
Deaths	Middle SDI	Both	55 to 59	Aortic aneurysm	Rate	2019	1.751095712	1.953289	1.564881	0.343310012
Deaths	Middle SDI	Both	60 to 64	Aortic aneurysm	Rate	2019	3.000767147	3.347278	2.694325	0.588313591
Deaths	Middle SDI	Both	65 to 69	Aortic aneurysm	Rate	2019	5.100625233	5.631875	4.576771	1
Deaths	Middle SDI	Both	70 to 74	Aortic aneurysm	Rate	2019	8.407544301	9.303591	7.536737	1.648336021
Deaths	Middle SDI	Both	75 to 79	Aortic aneurysm	Rate	2019	13.73580355	15.2643	12.30573	2.692964671
Deaths	Middle SDI	Both	80 to 84	Aortic aneurysm	Rate	2019	21.41274609	23.84393	18.75084	4.198063004
Deaths	Middle SDI	Both	85 to 89	Aortic aneurysm	Rate	2019	30.33374097	34.21606	25.88253	5.947063267
Deaths	Middle SDI	Both	90 to 94	Aortic aneurysm	Rate	2019	40.43309798	46.36067	32.07983	7.927086608
Deaths	Middle SDI	Both	95 plus	Aortic aneurysm	Rate	2019	52.58295158	62.17887	39.39579	10.30911882
DALYs (Di	Global	Both	15 to 19	Aortic aneurysm	Rate	2019	2.645770647	3.019593	2.319712	0.015323439
DALYs (Di	Global	Both	20 to 24	Aortic aneurysm	Rate	2019	4.17830275	4.704217	3.775078	0.024199365
DALYs (Di	Global	Both	25 to 29	Aortic aneurysm	Rate	2019	6.988757604	7.860249	6.348901	0.040476601
DALYs (Di	Global	Both	30 to 34	Aortic aneurysm	Rate	2019	10.62686509	11.86284	9.735066	0.061547332
DALYs (Di	Global	Both	35 to 39	Aortic aneurysm	Rate	2019	17.65424029	19.55098	16.09614	0.102247593
DALYs (Di	Global	Both	40 to 44	Aortic aneurysm	Rate	2019	25.67969885	28.08601	23.70776	0.148728428
DALYs (Di	Global	Both	45 to 49	Aortic aneurysm	Rate	2019	36.40099618	39.54978	33.78832	0.21082268
DALYs (Di	Global	Both	50 to 54	Aortic aneurysm	Rate	2019	51.91280486	55.92862	48.55401	0.300662009
DALYs (Di	Global	Both	55 to 59	Aortic aneurysm	Rate	2019	82.33910672	88.05301	77.08928	0.476881211
DALYs (Di	Global	Both	60 to 64	Aortic aneurysm	Rate	2019	121.5151583	129.5561	113.6793	0.703776106
DALYs (Di	Global	Both	65 to 69	Aortic aneurysm	Rate	2019	172.6616707	182.998	162.1034	1
DALYs (Di	Global	Both	70 to 74	Aortic aneurysm	Rate	2019	232.1201028	244.675	218.0937	1.344363818
DALYs (Di	Global	Both	75 to 79	Aortic aneurysm	Rate	2019	299.9178907	315.4693	278.0749	1.73702646
DALYs (Di	Global	Both	80 to 84	Aortic aneurysm	Rate	2019	368.0441386	393.5774	322.8854	2.131591436
DALYs (Di	Global	Both	85 to 89	Aortic aneurysm	Rate	2019	436.4178078	476.4367	361.7361	2.527589394
DALYs (Di	Global	Both	90 to 94	Aortic aneurysm	Rate	2019	485.3773475	545.0771	376.9162	2.811147057
DALYs (Di	Global	Both	95 plus	Aortic aneurysm	Rate	2019	498.7405317	573.8989	368.3141	2.888542255
DALYs (Di	High-middle SDI	Both	70 to 74	Aortic aneurysm	Rate	2019	253.8887992	270.9295	237.1205	1.206379938
DALYs (Di	High-middle SDI	Both	75 to 79	Aortic aneurysm	Rate	2019	302.0044871	322.3237	278.0962	1.435006804
DALYs (Di	High-middle SDI	Both	80 to 84	Aortic aneurysm	Rate	2019	326.7586578	352.3456	289.2729	1.552628908
DALYs (Di	High-middle SDI	Both	85 to 89	Aortic aneurysm	Rate	2019	341.5847501	371.4018	293.1701	1.623076681
DALYs (Di	High-middle SDI	Both	90 to 94	Aortic aneurysm	Rate	2019	333.3235791	369.6248	268.1597	1.583822838
DALYs (Di	High-middle SDI	Both	95 plus	Aortic aneurysm	Rate	2019	301.4860708	342.5307	234.6631	1.432543493
DALYs (Di	High-middle SDI	Both	15 to 19	Aortic aneurysm	Rate	2019	3.531392722	3.989345	3.15317	0.016779792
DALYs (Di	High-middle SDI	Both	20 to 24	Aortic aneurysm	Rate	2019	5.693996465	6.483715	5.074919	0.027055637
DALYs (Di	High-middle SDI	Both	25 to 29	Aortic aneurysm	Rate	2019	9.140213459	10.41	8.184443	0.043430707
DALYs (Di	High-middle SDI	Both	30 to 34	Aortic aneurysm	Rate	2019	14.07574927	16.03099	12.66255	0.066882437
DALYs (Di	High-middle SDI	Both	35 to 39	Aortic aneurysm	Rate	2019	23.88075918	26.77447	21.71362	0.113471996
DALYs (Di	High-middle SDI	Both	40 to 44	Aortic aneurysm	Rate	2019	36.33085024	39.9771	33.05269	0.172629943
DALYs (Di	High-middle SDI	Both	45 to 49	Aortic aneurysm	Rate	2019	49.93998612	54.65964	45.67735	0.237295216
DALYs (Di	High-middle SDI	Both	50 to 54	Aortic aneurysm	Rate	2019	69.50126663	75.49593	64.04579	0.330242744
DALYs (Di	High-middle SDI	Both	55 to 59	Aortic aneurysm	Rate	2019	107.5598425	116.3347	99.68137	0.511082161
DALYs (Di	High-middle SDI	Both	60 to 64	Aortic aneurysm	Rate	2019	155.9632782	167.9184	144.736	0.741076292

DALYs (Di	High-middle SDI	Both	65 to 69	Aortic aneurysm	Rate	2019	210.4550907	225.6169	196.3654	1
DALYs (Di	High SDI	Both	95 plus	Aortic aneurysm	Rate	2019	698.7360512	816.5854	504.4246	3.211943396
DALYs (Di	High SDI	Both	15 to 19	Aortic aneurysm	Rate	2019	2.214947808	2.453881	2.017747	0.010181652
DALYs (Di	High SDI	Both	20 to 24	Aortic aneurysm	Rate	2019	4.22245976	4.6239	3.873983	0.019409764
DALYs (Di	High SDI	Both	25 to 29	Aortic aneurysm	Rate	2019	7.528892767	8.254218	6.897227	0.034608744
DALYs (Di	High SDI	Both	30 to 34	Aortic aneurysm	Rate	2019	12.43073793	13.601	11.30998	0.057141501
DALYs (Di	High SDI	Both	35 to 39	Aortic aneurysm	Rate	2019	20.30380662	21.98416	18.87437	0.09333235
DALYs (Di	High SDI	Both	40 to 44	Aortic aneurysm	Rate	2019	32.07194328	34.23475	30.05971	0.147428011
DALYs (Di	High SDI	Both	45 to 49	Aortic aneurysm	Rate	2019	47.18560797	49.84159	44.49281	0.216902365
DALYs (Di	High SDI	Both	50 to 54	Aortic aneurysm	Rate	2019	67.04351642	70.54813	63.45953	0.308185014
DALYs (Di	High SDI	Both	55 to 59	Aortic aneurysm	Rate	2019	98.86130771	103.8167	93.59253	0.454444742
DALYs (Di	High SDI	Both	60 to 64	Aortic aneurysm	Rate	2019	147.518426	154.5214	140.0876	0.678111332
DALYs (Di	High SDI	Both	65 to 69	Aortic aneurysm	Rate	2019	217.5430775	227.6272	206.4038	1
DALYs (Di	High SDI	Both	70 to 74	Aortic aneurysm	Rate	2019	322.8609408	339.1063	303.7175	1.484124177
DALYs (Di	High SDI	Both	75 to 79	Aortic aneurysm	Rate	2019	432.3142885	455.627	398.2161	1.98725831
DALYs (Di	High SDI	Both	80 to 84	Aortic aneurysm	Rate	2019	565.7029275	612.6077	482.01	2.600417968
DALYs (Di	High SDI	Both	85 to 89	Aortic aneurysm	Rate	2019	693.2799469	765.9435	557.8174	3.186862827
DALYs (Di	High SDI	Both	90 to 94	Aortic aneurysm	Rate	2019	730.8499495	832.6441	545.0679	3.359564266
DALYs (Di	Low-middle SDI	Both	15 to 19	Aortic aneurysm	Rate	2019	2.216007651	2.802164	1.784292	0.014297532
DALYs (Di	Low-middle SDI	Both	20 to 24	Aortic aneurysm	Rate	2019	3.54443214	4.504984	2.925601	0.022868438
DALYs (Di	Low-middle SDI	Both	25 to 29	Aortic aneurysm	Rate	2019	6.197586817	7.636342	5.115778	0.039986413
DALYs (Di	Low-middle SDI	Both	30 to 34	Aortic aneurysm	Rate	2019	9.620759326	12.02966	7.523694	0.062072492
DALYs (Di	Low-middle SDI	Both	35 to 39	Aortic aneurysm	Rate	2019	16.11352595	20.14965	12.47786	0.103963386
DALYs (Di	Low-middle SDI	Both	40 to 44	Aortic aneurysm	Rate	2019	22.34006286	27.46231	17.8318	0.144136584
DALYs (Di	Low-middle SDI	Both	45 to 49	Aortic aneurysm	Rate	2019	33.10623264	40.32066	26.79533	0.213599187
DALYs (Di	Low-middle SDI	Both	50 to 54	Aortic aneurysm	Rate	2019	48.28323527	58.57929	39.27542	0.311520188
DALYs (Di	Low-middle SDI	Both	55 to 59	Aortic aneurysm	Rate	2019	77.66982864	94.89689	62.51286	0.501120512
DALYs (Di	Low-middle SDI	Both	60 to 64	Aortic aneurysm	Rate	2019	110.3098987	133.404	89.60944	0.71171205
DALYs (Di	Low-middle SDI	Both	65 to 69	Aortic aneurysm	Rate	2019	154.9923157	189.232	125.8276	1
DALYs (Di	Low-middle SDI	Both	70 to 74	Aortic aneurysm	Rate	2019	194.1879462	230.577	162.3826	1.252887573
DALYs (Di	Low-middle SDI	Both	75 to 79	Aortic aneurysm	Rate	2019	238.467612	281.1132	200.878	1.538577
DALYs (Di	Low-middle SDI	Both	80 to 84	Aortic aneurysm	Rate	2019	278.2838248	325.4439	229.3596	1.795468528
DALYs (Di	Low-middle SDI	Both	85 to 89	Aortic aneurysm	Rate	2019	273.5404037	316.0695	227.9676	1.764864294
DALYs (Di	Low-middle SDI	Both	90 to 94	Aortic aneurysm	Rate	2019	272.7026443	320.202	214.9001	1.759459126
DALYs (Di	Low-middle SDI	Both	95 plus	Aortic aneurysm	Rate	2019	243.8587681	286.0522	185.6393	1.573360377
DALYs (Di	Low SDI	Both	15 to 19	Aortic aneurysm	Rate	2019	2.379423658	3.329642	1.600101	0.013276775
DALYs (Di	Low SDI	Both	20 to 24	Aortic aneurysm	Rate	2019	3.781820714	5.153985	2.55863	0.021101909
DALYs (Di	Low SDI	Both	25 to 29	Aortic aneurysm	Rate	2019	6.954162682	9.483948	4.88675	0.038803031
DALYs (Di	Low SDI	Both	30 to 34	Aortic aneurysm	Rate	2019	8.737748559	11.81822	6.134238	0.048755133
DALYs (Di	Low SDI	Both	35 to 39	Aortic aneurysm	Rate	2019	15.57667707	21.16526	11.0213	0.086915177
DALYs (Di	Low SDI	Both	40 to 44	Aortic aneurysm	Rate	2019	21.26597757	28.50432	15.0464	0.118660495
DALYs (Di	Low SDI	Both	45 to 49	Aortic aneurysm	Rate	2019	33.30094314	44.00792	23.91144	0.185813532
DALYs (Di	Low SDI	Both	50 to 54	Aortic aneurysm	Rate	2019	48.62063265	63.69992	35.21718	0.271294763
DALYs (Di	Low SDI	Both	55 to 59	Aortic aneurysm	Rate	2019	84.36967271	108.2761	60.92132	0.470768254
DALYs (Di	Low SDI	Both	60 to 64	Aortic aneurysm	Rate	2019	124.2753378	160.2238	90.29778	0.693434997
DALYs (Di	Low SDI	Both	65 to 69	Aortic aneurysm	Rate	2019	179.2169969	229.0928	131.4107	1
DALYs (Di	Low SDI	Both	70 to 74	Aortic aneurysm	Rate	2019	222.8352059	281.7964	164.3097	1.243382099
DALYs (Di	Low SDI	Both	75 to 79	Aortic aneurysm	Rate	2019	287.2037949	355.0543	214.378	1.602547748
DALYs (Di	Low SDI	Both	80 to 84	Aortic aneurysm	Rate	2019	301.6446898	372.2319	224.0485	1.683125457
DALYs (Di	Low SDI	Both	85 to 89	Aortic aneurysm	Rate	2019	282.5222718	347.7661	207.7397	1.576425656
DALYs (Di	Low SDI	Both	90 to 94	Aortic aneurysm	Rate	2019	263.8722167	326.8335	190.3162	1.472361557
DALYs (Di	Low SDI	Both	95 plus	Aortic aneurysm	Rate	2019	201.7538895	253.9543	144.0382	1.125751982
DALYs (Di	Middle SDI	Both	15 to 19	Aortic aneurysm	Rate	2019	2.963267372	3.550968	2.47329	0.024872892
DALYs (Di	Middle SDI	Both	20 to 24	Aortic aneurysm	Rate	2019	4.201683763	4.875289	3.622722	0.035267836
DALYs (Di	Middle SDI	Both	25 to 29	Aortic aneurysm	Rate	2019	6.213104958	7.139008	5.44433	0.05215118
DALYs (Di	Middle SDI	Both	30 to 34	Aortic aneurysm	Rate	2019	9.201578485	10.42198	8.172367	0.077235646
DALYs (Di	Middle SDI	Both	35 to 39	Aortic aneurysm	Rate	2019	14.40273616	16.18885	12.8235	0.120892805
DALYs (Di	Middle SDI	Both	40 to 44	Aortic aneurysm	Rate	2019	19.59095828	22.05476	17.37069	0.164441386
DALYs (Di	Middle SDI	Both	45 to 49	Aortic aneurysm	Rate	2019	25.63561336	29.01659	22.66427	0.215178641
DALYs (Di	Middle SDI	Both	50 to 54	Aortic aneurysm	Rate	2019	36.02873697	40.09032	32.12179	0.302415806
DALYs (Di	Middle SDI	Both	55 to 59	Aortic aneurysm	Rate	2019	56.80324644	63.36134	50.76277	0.476791611
DALYs (Di	Middle SDI	Both	60 to 64	Aortic aneurysm	Rate	2019	83.54125921	93.18605	75.01215	0.701223505
DALYs (Di	Middle SDI	Both	65 to 69	Aortic aneurysm	Rate	2019	119.1364217	131.5583	106.8961	1
DALYs (Di	Middle SDI	Both	70 to 74	Aortic aneurysm	Rate	2019	160.570027	177.6714	143.9368	1.347782859
DALYs (Di	Middle SDI	Both	75 to 79	Aortic aneurysm	Rate	2019	207.4530891	230.5579	185.8696	1.741307035
DALYs (Di	Middle SDI	Both	80 to 84	Aortic aneurysm	Rate	2019	248.4395069	276.6316	217.5282	2.085336318
DALYs (Di	Middle SDI	Both	85 to 89	Aortic aneurysm	Rate	2019	268.6547069	303.0581	229.2634	2.255017425
DALYs (Di	Middle SDI	Both	90 to 94	Aortic aneurysm	Rate	2019	277.0119398	317.6598	219.965	2.325165855

DALYs (DiMiddle SDI	Both	95 plus	Aortic aneurysm	Rate	2019	267.0255365	314.9856	200.5591	2.241342595
