# Supplementary material for: Burden of Aortic Aneurysm and Its Attributable Risk Factors from 1990 to 2019: An Analysis of the Global Burden of Disease Study 2019
Source: Front Cardiovasc Med. 2022 May 31;9:901225. doi: 10.3389/fcvm.2022.901225 (PMC9197430; doi:10.3389/fcvm.2022.901225)
Supplement: Supplementary Table 6 — Estimated annual percentage changes of aortic aneurism-related age-standardized deaths and DALYs in 31 GBD regions from 1990 to 2019. DALY, disability-adjusted life year rate. SDI, socio-demographic index; EAPC, estimated annual percentage changes; GBD, Global Burden of Disease. [file Data_Sheet_6.PDF]

measure	location_id	EAPC0.025	EAPC0.5	EAPC0.975	p	gender
DALYs	Andean Latin America	-0.24	-0.12	0	0.043354198	Both
DALYs	Andean Latin America	-0.29	-0.14	0	0.049127779	Male
DALYs	Andean Latin America	-0.16	-0.05	0.06	0.374709178	Female
Deaths	Andean Latin America	-0.05	0.06	0.18	0.285225724	Both
Deaths	Andean Latin America	0	0.14	0.29	0.045316639	Male
Deaths	Andean Latin America	-0.13	-0.02	0.09	0.771480766	Female
DALYs	Australasia	-4.23	-3.95	-3.68	2.45E-22	Both
DALYs	Australasia	-4.78	-4.47	-4.16	1.84E-22	Male
DALYs	Australasia	-3.48	-3.23	-2.98	4.29E-21	Female
Deaths	Australasia	-3.94	-3.67	-3.41	5.92E-22	Both
Deaths	Australasia	-4.62	-4.32	-4.02	1.90E-22	Male
Deaths	Australasia	-3.16	-2.92	-2.68	2.27E-20	Female
DALYs	Caribbean	-0.7	-0.57	-0.43	2.07E-09	Both
DALYs	Caribbean	-0.76	-0.6	-0.45	1.30E-08	Male
DALYs	Caribbean	-0.53	-0.42	-0.3	3.00E-08	Female
Deaths	Caribbean	-0.75	-0.62	-0.48	4.10E-10	Both
Deaths	Caribbean	-0.82	-0.66	-0.5	4.56E-09	Male
Deaths	Caribbean	-0.52	-0.41	-0.31	1.42E-08	Female
DALYs	Central Asia	1.71	1.84	1.97	2.25E-22	Both
DALYs	Central Asia	1.67	1.84	2.01	1.59E-19	Male
DALYs	Central Asia	1.72	1.77	1.82	1.78E-33	Female
Deaths	Central Asia	2.13	2.23	2.33	8.33E-28	Both
Deaths	Central Asia	2.13	2.27	2.41	3.15E-24	Male
Deaths	Central Asia	1.98	2.06	2.13	1.89E-30	Female
DALYs	Central Europe	0.52	0.8	1.08	2.10E-06	Both
DALYs	Central Europe	0.41	0.74	1.07	8.58E-05	Male
DALYs	Central Europe	0.68	0.86	1.04	1.77E-10	Female
Deaths	Central Europe	0.84	1.12	1.41	6.49E-09	Both
Deaths	Central Europe	0.72	1.07	1.42	8.40E-07	Male
Deaths	Central Europe	1.03	1.21	1.4	8.00E-14	Female
DALYs	Central Latin America	-1.03	-0.81	-0.59	4.43E-08	Both
DALYs	Central Latin America	-0.98	-0.77	-0.55	7.77E-08	Male
DALYs	Central Latin America	-0.93	-0.68	-0.42	8.70E-06	Female
Deaths	Central Latin America	-0.8	-0.57	-0.34	2.48E-05	Both
Deaths	Central Latin America	-0.76	-0.54	-0.31	3.80E-05	Male
Deaths	Central Latin America	-0.64	-0.38	-0.11	0.00777486	Female
DALYs	Central Sub-Saharan Africa	-1.41	-1.21	-1.01	1.01E-12	Both
DALYs	Central Sub-Saharan Africa	-1.41	-1.22	-1.02	4.01E-13	Male
DALYs	Central Sub-Saharan Africa	-1.25	-1.07	-0.9	4.87E-13	Female
Deaths	Central Sub-Saharan Africa	-1.41	-1.21	-1.01	1.02E-12	Both
Deaths	Central Sub-Saharan Africa	-1.33	-1.14	-0.95	8.08E-13	Male
Deaths	Central Sub-Saharan Africa	-1.26	-1.08	-0.9	9.30E-13	Female
DALYs	East Asia	-0.19	-0.15	-0.1	1.44E-07	Both
DALYs	East Asia	0.08	0.14	0.2	8.55E-05	Male
DALYs	East Asia	-0.86	-0.82	-0.78	7.10E-27	Female
Deaths	East Asia	-0.2	-0.16	-0.12	2.39E-08	Both
Deaths	East Asia	0.11	0.17	0.23	2.35E-06	Male
Deaths	East Asia	-0.81	-0.77	-0.72	1.38E-25	Female
DALYs	Eastern Europe	0.83	1.06	1.29	3.95E-10	Both
DALYs	Eastern Europe	0.78	1.05	1.32	7.66E-09	Male
DALYs	Eastern Europe	0.48	0.69	0.89	1.29E-07	Female
Deaths	Eastern Europe	0.92	1.15	1.37	3.49E-11	Both
Deaths	Eastern Europe	0.76	1	1.25	3.89E-09	Male
Deaths	Eastern Europe	0.62	0.88	1.15	2.39E-07	Female
DALYs	Eastern Sub-Saharan Africa	-1.55	-1.38	-1.21	5.91E-16	Both
DALYs	Eastern Sub-Saharan Africa	-1.51	-1.36	-1.22	1.81E-17	Male
DALYs	Eastern Sub-Saharan Africa	-1.5	-1.29	-1.08	5.89E-13	Female
Deaths	Eastern Sub-Saharan Africa	-1.44	-1.28	-1.11	3.01E-15	Both
Deaths	Eastern Sub-Saharan Africa	-1.38	-1.25	-1.11	4.75E-17	Male
Deaths	Eastern Sub-Saharan Africa	-1.42	-1.21	-1	2.09E-12	Female
DALYs	Global	-1.17	-1.06	-0.95	2.17E-18	Both
DALYs	Global	-1.4	-1.29	-1.18	2.66E-20	Male
DALYs	Global	-0.86	-0.76	-0.65	4.90E-15	Female
Deaths	Global	-1.12	-1	-0.89	7.21E-17	Both
Deaths	Global	-1.46	-1.34	-1.22	2.34E-19	Male
Deaths	Global	-0.71	-0.61	-0.5	2.85E-12	Female
DALYs	High SDI	-2.09	-1.94	-1.79	1.77E-21	Both

DALYs	High SDI	-2.69	-2.53	-2.37	1.35E-23	Male
DALYs	High SDI	-1.34	-1.19	-1.05	4.94E-16	Female
Deaths	High SDI	-1.94	-1.79	-1.63	4.00E-20	Both
Deaths	High SDI	-2.72	-2.55	-2.37	1.38E-22	Male
Deaths	High SDI	-1.04	-0.9	-0.76	1.38E-13	Female
DALYs	High-income Asia Pacific	1.08	1.23	1.39	7.30E-16	Both
DALYs	High-income Asia Pacific	0.54	0.7	0.86	9.77E-10	Male
DALYs	High-income Asia Pacific	1.61	1.76	1.91	2.72E-20	Female
Deaths	High-income Asia Pacific	1.26	1.42	1.59	7.17E-17	Both
Deaths	High-income Asia Pacific	0.46	0.64	0.81	2.80E-08	Male
Deaths	High-income Asia Pacific	2.03	2.21	2.4	9.88E-21	Female
DALYs	High-income North America	-3.42	-3.16	-2.91	1.68E-20	Both
DALYs	High-income North America	-3.99	-3.7	-3.42	2.81E-21	Male
DALYs	High-income North America	-2.74	-2.47	-2.21	2.15E-17	Female
Deaths	High-income North America	-3.53	-3.26	-2.99	2.54E-20	Both
Deaths	High-income North America	-4.34	-4.03	-3.72	3.88E-21	Male
Deaths	High-income North America	-2.62	-2.36	-2.09	5.45E-17	Female
DALYs	High-middle SDI	-0.57	-0.39	-0.2	0.000167177	Both
DALYs	High-middle SDI	-0.81	-0.59	-0.38	4.13E-06	Male
DALYs	High-middle SDI	-0.28	-0.16	-0.05	0.007805094	Female
Deaths	High-middle SDI	-0.38	-0.18	0.01	0.063579914	Both
Deaths	High-middle SDI	-0.69	-0.45	-0.22	0.00047529	Male
Deaths	High-middle SDI	-0.03	0.1	0.23	0.124519466	Female
DALYs	Low SDI	-0.9	-0.77	-0.64	1.46E-12	Both
DALYs	Low SDI	-0.7	-0.6	-0.51	3.40E-13	Male
DALYs	Low SDI	-1.14	-0.95	-0.75	9.15E-11	Female
Deaths	Low SDI	-0.8	-0.68	-0.57	1.71E-12	Both
Deaths	Low SDI	-0.56	-0.48	-0.4	1.19E-12	Male
Deaths	Low SDI	-1.06	-0.89	-0.71	4.30E-11	Female
DALYs	Low-middle SDI	0.19	0.25	0.32	3.87E-09	Both
DALYs	Low-middle SDI	0.31	0.38	0.44	1.04E-12	Male
DALYs	Low-middle SDI	0.14	0.2	0.27	5.02E-07	Female
Deaths	Low-middle SDI	0.2	0.26	0.33	9.10E-09	Both
Deaths	Low-middle SDI	0.33	0.4	0.47	3.17E-12	Male
Deaths	Low-middle SDI	0.14	0.2	0.27	7.87E-07	Female
DALYs	Middle SDI	-0.14	-0.06	0.01	0.104840959	Both
DALYs	Middle SDI	-0.03	0.04	0.11	0.233992923	Male
DALYs	Middle SDI	-0.33	-0.25	-0.17	4.71E-07	Female
Deaths	Middle SDI	-0.1	-0.02	0.07	0.70157999	Both
Deaths	Middle SDI	0.01	0.09	0.17	0.038131787	Male
Deaths	Middle SDI	-0.27	-0.19	-0.12	2.12E-05	Female
DALYs	North Africa and Middle East	-0.93	-0.82	-0.7	1.01E-14	Both
DALYs	North Africa and Middle East	-1.06	-0.92	-0.78	7.28E-14	Male
DALYs	North Africa and Middle East	-0.58	-0.52	-0.47	2.39E-18	Female
Deaths	North Africa and Middle East	-0.57	-0.48	-0.4	1.03E-12	Both
Deaths	North Africa and Middle East	-0.67	-0.55	-0.44	1.76E-10	Male
Deaths	North Africa and Middle East	-0.47	-0.38	-0.29	1.60E-09	Female
DALYs	Oceania	-0.66	-0.61	-0.56	2.48E-21	Both
DALYs	Oceania	-0.61	-0.56	-0.51	1.06E-19	Male
DALYs	Oceania	-0.78	-0.7	-0.63	3.02E-17	Female
Deaths	Oceania	-0.76	-0.7	-0.65	3.37E-21	Both
Deaths	Oceania	-0.65	-0.59	-0.53	2.65E-18	Male
Deaths	Oceania	-0.91	-0.84	-0.76	2.23E-19	Female
DALYs	South Asia	-0.01	0.07	0.14	0.074465444	Both
DALYs	South Asia	0.07	0.14	0.22	0.000790738	Male
DALYs	South Asia	0.12	0.2	0.28	1.91E-05	Female
Deaths	South Asia	-0.03	0.06	0.15	0.18428423	Both
Deaths	South Asia	0.07	0.16	0.26	0.001004526	Male
Deaths	South Asia	0.05	0.15	0.25	0.004313994	Female
DALYs	Southeast Asia	0.58	0.65	0.72	1.01E-17	Both
DALYs	Southeast Asia	0.82	0.88	0.93	1.73E-23	Male
DALYs	Southeast Asia	0.21	0.32	0.42	8.03E-07	Female
Deaths	Southeast Asia	0.56	0.62	0.69	4.46E-18	Both
Deaths	Southeast Asia	0.88	0.93	0.99	2.76E-25	Male
Deaths	Southeast Asia	0.16	0.25	0.35	7.94E-06	Female
DALYs	Southern Latin America	-1.55	-1.25	-0.95	3.08E-09	Both
DALYs	Southern Latin America	-1.91	-1.55	-1.2	1.03E-09	Male
DALYs	Southern Latin America	-0.65	-0.47	-0.28	1.40E-05	Female

Deaths	Southern Latin America	-1.46	-1.16	-0.85	2.64E-08	Both
Deaths	Southern Latin America	-1.85	-1.48	-1.12	5.46E-09	Male
Deaths	Southern Latin America	-0.55	-0.35	-0.15	0.001367354	Female
DALYs	Southern Sub-Saharan Africa	-2.42	-1.94	-1.47	4.70E-09	Both
DALYs	Southern Sub-Saharan Africa	-2.17	-1.59	-1.01	5.72E-06	Male
DALYs	Southern Sub-Saharan Africa	-2.75	-2.4	-2.06	3.24E-14	Female
Deaths	Southern Sub-Saharan Africa	-2.45	-1.98	-1.51	2.60E-09	Both
Deaths	Southern Sub-Saharan Africa	-2.1	-1.53	-0.97	6.29E-06	Male
Deaths	Southern Sub-Saharan Africa	-2.81	-2.44	-2.06	1.67E-13	Female
DALYs	Tropical Latin America	0.2	0.42	0.63	0.000542903	Both
DALYs	Tropical Latin America	-0.09	0.14	0.37	0.232910002	Male
DALYs	Tropical Latin America	0.77	0.99	1.2	3.51E-10	Female
Deaths	Tropical Latin America	0.54	0.76	0.99	1.03E-07	Both
Deaths	Tropical Latin America	0.18	0.41	0.64	0.001208057	Male
Deaths	Tropical Latin America	1.21	1.43	1.66	2.17E-13	Female
DALYs	Western Europe	-2.39	-2.14	-1.89	1.12E-16	Both
DALYs	Western Europe	-2.91	-2.64	-2.38	4.84E-18	Male
DALYs	Western Europe	-1.59	-1.39	-1.19	2.12E-14	Female
Deaths	Western Europe	-2.23	-1.98	-1.72	2.19E-15	Both
Deaths	Western Europe	-2.79	-2.51	-2.22	7.87E-17	Male
Deaths	Western Europe	-1.54	-1.33	-1.12	2.43E-13	Female
DALYs	Western Sub-Saharan Africa	-1.62	-1.42	-1.22	1.91E-14	Both
DALYs	Western Sub-Saharan Africa	-0.4	-0.3	-0.2	1.19E-06	Male
DALYs	Western Sub-Saharan Africa	-3.66	-3.22	-2.79	7.70E-15	Female
Deaths	Western Sub-Saharan Africa	-1.52	-1.33	-1.14	1.92E-14	Both
Deaths	Western Sub-Saharan Africa	-0.2	-0.12	-0.04	0.003434308	Male
Deaths	Western Sub-Saharan Africa	-3.58	-3.17	-2.76	2.19E-15	Female
DALYs	World Bank High Income	-1.85	-1.69	-1.53	5.94E-19	Both
DALYs	World Bank High Income	-2.38	-2.21	-2.04	3.17E-21	Male
DALYs	World Bank High Income	-1.14	-0.99	-0.85	7.54E-14	Female
Deaths	World Bank High Income	-1.72	-1.55	-1.39	1.26E-17	Both
Deaths	World Bank High Income	-2.41	-2.22	-2.03	3.10E-20	Male
Deaths	World Bank High Income	-0.9	-0.75	-0.61	2.64E-11	Female
DALYs	World Bank Low Income	-1.24	-1.09	-0.93	1.81E-14	Both
DALYs	World Bank Low Income	-1.04	-0.92	-0.79	1.18E-14	Male
DALYs	World Bank Low Income	-1.47	-1.28	-1.09	6.83E-14	Female
Deaths	World Bank Low Income	-1.16	-1.02	-0.88	9.62E-15	Both
Deaths	World Bank Low Income	-0.9	-0.79	-0.68	1.96E-14	Male
Deaths	World Bank Low Income	-1.4	-1.23	-1.06	1.83E-14	Female
DALYs	World Bank Lower Middle Income	0.13	0.18	0.23	1.05E-07	Both
DALYs	World Bank Lower Middle Income	0.22	0.27	0.32	2.02E-11	Male
DALYs	World Bank Lower Middle Income	0.04	0.1	0.16	0.001563185	Female
Deaths	World Bank Lower Middle Income	0.16	0.22	0.28	1.93E-08	Both
Deaths	World Bank Lower Middle Income	0.27	0.33	0.39	3.07E-12	Male
Deaths	World Bank Lower Middle Income	0.05	0.12	0.18	0.000934142	Female
DALYs	World Bank Upper Middle Income	-0.45	-0.35	-0.24	2.79E-07	Both
DALYs	World Bank Upper Middle Income	-0.48	-0.37	-0.26	2.10E-07	Male
DALYs	World Bank Upper Middle Income	-0.47	-0.38	-0.29	2.74E-09	Female
Deaths	World Bank Upper Middle Income	-0.3	-0.2	-0.09	0.000714068	Both
Deaths	World Bank Upper Middle Income	-0.38	-0.27	-0.16	2.58E-05	Male
Deaths	World Bank Upper Middle Income	-0.27	-0.17	-0.08	0.000868048	Female
