# Supplementary material for: Burden of Aortic Aneurysm and Its Attributable Risk Factors from 1990 to 2019: An Analysis of the Global Burden of Disease Study 2019
Source: Front Cardiovasc Med. 2022 May 31;9:901225. doi: 10.3389/fcvm.2022.901225 (PMC9197430; doi:10.3389/fcvm.2022.901225)
Supplement: Supplementary Table 7 — Estimated annual percentage changes of aortic aneurism-related age-standardized deaths and DALYs in 204 countries and territories from 1990 to 2019. DALY, disability-adjusted life year rate. SDI, socio-demographic index; EAPC, estimated annual percentage changes. [file Data_Sheet_7.PDF]

measure	location_id	EAPC0.025	EAPC0.5	EAPC0.975	p	gender
DALYs	Afghanistan	-0.43	-0.37	-0.3	6.29E-12	Both
DALYs	Afghanistan	0.24	0.29	0.34	2.53E-12	Female
DALYs	Afghanistan	-0.54	-0.45	-0.36	4.92E-11	Male
Deaths	Afghanistan	-0.3	-0.25	-0.2	1.49E-11	Both
Deaths	Afghanistan	0.37	0.41	0.44	4.48E-20	Female
Deaths	Afghanistan	-0.37	-0.31	-0.25	9.03E-12	Male
DALYs	Albania	-0.28	-0.02	0.24	0.88789332	Both
DALYs	Albania	-0.05	0.15	0.35	0.128827961	Female
DALYs	Albania	-0.44	-0.13	0.17	0.374448103	Male
Deaths	Albania	-0.14	0.11	0.37	0.364319084	Both
Deaths	Albania	0.1	0.3	0.49	0.003903562	Female
Deaths	Albania	-0.43	-0.13	0.16	0.359485921	Male
DALYs	Algeria	-0.71	-0.67	-0.63	1.78E-24	Both
DALYs	Algeria	-0.26	-0.22	-0.18	2.73E-11	Female
DALYs	Algeria	-0.95	-0.9	-0.85	5.20E-25	Male
Deaths	Algeria	-0.63	-0.57	-0.52	5.18E-19	Both
Deaths	Algeria	-0.11	-0.06	-0.01	0.025058735	Female
Deaths	Algeria	-0.85	-0.8	-0.75	5.48E-23	Male
DALYs	American Samoa	-2.68	-2.42	-2.15	3.05E-17	Both
DALYs	American Samoa	-3.95	-3.59	-3.22	4.58E-18	Female
DALYs	American Samoa	-1.66	-1.44	-1.23	5.64E-14	Male
Deaths	American Samoa	-2.77	-2.51	-2.24	1.18E-17	Both
Deaths	American Samoa	-3.99	-3.64	-3.29	1.71E-18	Female
Deaths	American Samoa	-1.49	-1.29	-1.09	2.74E-13	Male
DALYs	Andorra	-1.04	-0.94	-0.83	5.30E-17	Both
DALYs	Andorra	0.04	0.18	0.32	0.014018772	Female
DALYs	Andorra	-1.42	-1.33	-1.24	1.01E-22	Male
Deaths	Andorra	-0.87	-0.76	-0.64	5.40E-14	Both
Deaths	Andorra	0.15	0.3	0.45	0.000388956	Female
Deaths	Andorra	-1.27	-1.18	-1.09	1.52E-21	Male
DALYs	Angola	-1.06	-0.92	-0.77	3.10E-13	Both
DALYs	Angola	-0.76	-0.64	-0.52	1.54E-11	Female
DALYs	Angola	-0.99	-0.83	-0.68	1.62E-11	Male
Deaths	Angola	-0.9	-0.76	-0.63	5.95E-12	Both
Deaths	Angola	-0.7	-0.57	-0.44	1.04E-09	Female
Deaths	Angola	-0.8	-0.66	-0.51	3.03E-10	Male
DALYs	Antigua and Barbuda	-1.18	-0.95	-0.71	6.62E-09	Both
DALYs	Antigua and Barbuda	-0.44	-0.27	-0.11	0.001728802	Female
DALYs	Antigua and Barbuda	-1.77	-1.45	-1.12	8.70E-10	Male
Deaths	Antigua and Barbuda	-0.88	-0.64	-0.4	7.37E-06	Both
Deaths	Antigua and Barbuda	-0.23	-0.07	0.1	0.412387711	Female
Deaths	Antigua and Barbuda	-1.5	-1.16	-0.81	1.68E-07	Male
DALYs	Argentina	-1.86	-1.52	-1.17	9.97E-10	Both
DALYs	Argentina	-0.93	-0.69	-0.45	2.19E-06	Female
DALYs	Argentina	-2.15	-1.77	-1.38	4.93E-10	Male
Deaths	Argentina	-1.75	-1.41	-1.06	4.51E-09	Both
Deaths	Argentina	-0.88	-0.63	-0.38	2.07E-05	Female
Deaths	Argentina	-2.02	-1.64	-1.25	2.24E-09	Male
DALYs	Armenia	1.88	2.16	2.43	1.00E-15	Both
DALYs	Armenia	1.96	2.13	2.31	1.10E-20	Female
DALYs	Armenia	1.84	2.17	2.5	5.61E-14	Male
Deaths	Armenia	2.2	2.46	2.73	9.14E-18	Both
Deaths	Armenia	2.26	2.45	2.64	1.27E-21	Female
Deaths	Armenia	2.1	2.41	2.73	1.61E-15	Male
DALYs	Australia	-4.44	-4.17	-3.9	3.76E-23	Both
DALYs	Australia	-3.74	-3.49	-3.23	5.87E-22	Female
DALYs	Australia	-4.95	-4.65	-4.34	5.10E-23	Male
Deaths	Australia	-4.14	-3.88	-3.62	1.24E-22	Both
Deaths	Australia	-3.4	-3.15	-2.9	6.85E-21	Female
Deaths	Australia	-4.79	-4.5	-4.2	5.45E-23	Male
DALYs	Austria	-2.02	-1.79	-1.55	4.38E-15	Both
DALYs	Austria	-1.32	-1.11	-0.9	1.73E-11	Female
DALYs	Austria	-2.72	-2.45	-2.19	2.33E-17	Male
Deaths	Austria	-1.81	-1.59	-1.37	6.94E-15	Both
Deaths	Austria	-1.11	-0.93	-0.75	2.27E-11	Female
Deaths	Austria	-2.62	-2.38	-2.13	4.92E-18	Male
DALYs	Azerbaijan	0.68	0.86	1.04	2.30E-10	Both

DALYs	Azerbaijan	0.71	0.87	1.04	7.63E-12	Female
DALYs	Azerbaijan	0.56	0.76	0.95	1.39E-08	Male
Deaths	Azerbaijan	1.23	1.42	1.61	2.73E-15	Both
Deaths	Azerbaijan	0.91	1.09	1.27	5.23E-13	Female
Deaths	Azerbaijan	1.25	1.44	1.63	2.09E-15	Male
DALYs	Bahamas	-0.21	-0.04	0.13	0.607427731	Both
DALYs	Bahamas	0.07	0.23	0.4	0.005979251	Female
DALYs	Bahamas	-0.52	-0.33	-0.14	0.001169875	Male
Deaths	Bahamas	-0.2	-0.01	0.19	0.944561319	Both
Deaths	Bahamas	0.13	0.31	0.5	0.002012181	Female
Deaths	Bahamas	-0.65	-0.42	-0.18	0.001078457	Male
DALYs	Bahrain	-1.1	-0.84	-0.59	2.97E-07	Both
DALYs	Bahrain	0.5	0.69	0.87	1.98E-08	Female
DALYs	Bahrain	-1.79	-1.45	-1.11	2.15E-09	Male
Deaths	Bahrain	-0.54	-0.22	0.1	0.167549209	Both
Deaths	Bahrain	0.99	1.26	1.53	2.51E-10	Female
Deaths	Bahrain	-1.32	-0.92	-0.52	6.16E-05	Male
DALYs	Bangladesh	-0.1	0.09	0.27	0.348543146	Both
DALYs	Bangladesh	0.52	0.64	0.76	1.77E-11	Female
DALYs	Bangladesh	-0.33	-0.1	0.14	0.418761389	Male
Deaths	Bangladesh	0.12	0.36	0.61	0.005150588	Both
Deaths	Bangladesh	0.61	0.77	0.94	2.90E-10	Female
Deaths	Bangladesh	-0.1	0.22	0.53	0.16928083	Male
DALYs	Barbados	-0.7	-0.47	-0.24	0.000258091	Both
DALYs	Barbados	0.36	0.54	0.72	1.50E-06	Female
DALYs	Barbados	-1.43	-1.11	-0.8	6.61E-08	Male
Deaths	Barbados	-0.58	-0.34	-0.1	0.006910649	Both
Deaths	Barbados	0.43	0.63	0.83	4.91E-07	Female
Deaths	Barbados	-1.39	-1.06	-0.73	3.91E-07	Male
DALYs	Belarus	1.24	1.5	1.77	3.44E-12	Both
DALYs	Belarus	1.07	1.17	1.28	6.43E-20	Female
DALYs	Belarus	1.29	1.63	1.96	8.49E-11	Male
Deaths	Belarus	1.4	1.59	1.79	4.02E-16	Both
Deaths	Belarus	1.22	1.29	1.37	4.52E-25	Female
Deaths	Belarus	1.46	1.72	1.98	7.05E-14	Male
DALYs	Belgium	-2.82	-2.57	-2.32	1.10E-18	Both
DALYs	Belgium	-1.62	-1.45	-1.29	5.56E-17	Female
DALYs	Belgium	-3.41	-3.12	-2.84	2.71E-19	Male
Deaths	Belgium	-2.54	-2.28	-2.03	4.33E-17	Both
Deaths	Belgium	-1.66	-1.46	-1.25	1.21E-14	Female
Deaths	Belgium	-3.12	-2.83	-2.54	5.40E-18	Male
DALYs	Belize	-0.71	-0.19	0.33	0.459814378	Both
DALYs	Belize	-0.62	-0.32	-0.02	0.03990895	Female
DALYs	Belize	-0.78	-0.08	0.63	0.827698368	Male
Deaths	Belize	-0.66	-0.15	0.37	0.553941529	Both
Deaths	Belize	-0.51	-0.2	0.12	0.208972296	Female
Deaths	Belize	-0.81	-0.1	0.61	0.76714372	Male
DALYs	Benin	-1.34	-1.19	-1.04	1.41E-15	Both
DALYs	Benin	-3.26	-2.94	-2.61	3.81E-17	Female
DALYs	Benin	-0.24	-0.14	-0.04	0.006206129	Male
Deaths	Benin	-1.26	-1.12	-0.98	8.53E-16	Both
Deaths	Benin	-3.1	-2.81	-2.51	1.27E-17	Female
Deaths	Benin	-0.02	0.08	0.19	0.123447939	Male
DALYs	Bermuda	-2.26	-2.13	-2	8.76E-24	Both
DALYs	Bermuda	-3.24	-2.91	-2.57	1.09E-16	Female
DALYs	Bermuda	-2.08	-1.98	-1.87	1.27E-25	Male
Deaths	Bermuda	-2.02	-1.88	-1.74	1.24E-21	Both
Deaths	Bermuda	-2.86	-2.55	-2.23	5.84E-16	Female
Deaths	Bermuda	-1.83	-1.7	-1.58	4.11E-22	Male
DALYs	Bhutan	1.03	1.07	1.11	2.63E-30	Both
DALYs	Bhutan	1.27	1.33	1.39	2.03E-28	Female
DALYs	Bhutan	0.78	0.83	0.87	6.94E-26	Male
Deaths	Bhutan	1.4	1.46	1.51	2.10E-29	Both
Deaths	Bhutan	1.61	1.68	1.76	9.70E-28	Female
Deaths	Bhutan	1.07	1.11	1.16	1.92E-28	Male
DALYs	Bolivia (Plurinational State o	0.07	0.12	0.17	1.88E-05	Both
DALYs	Bolivia (Plurinational State o	0.42	0.55	0.69	3.27E-09	Female
DALYs	Bolivia (Plurinational State o	-0.26	-0.16	-0.05	0.00395644	Male

Deaths	Bolivia (Plurinational State o:	0.42	0.46	0.51	1.39E-18	Both
Deaths	Bolivia (Plurinational State o:	0.72	0.86	1.01	1.02E-12	Female
Deaths	Bolivia (Plurinational State o:	0.06	0.16	0.25	0.00237621	Male
DALYs	Bosnia and Herzegovina	0.58	0.78	0.97	5.26E-09	Both
DALYs	Bosnia and Herzegovina	0.54	0.75	0.95	3.26E-08	Female
DALYs	Bosnia and Herzegovina	0.55	0.76	0.97	4.39E-08	Male
Deaths	Bosnia and Herzegovina	0.83	1.12	1.41	1.11E-08	Both
Deaths	Bosnia and Herzegovina	0.5	0.87	1.25	5.56E-05	Female
Deaths	Bosnia and Herzegovina	1	1.27	1.54	2.25E-10	Male
DALYs	Botswana	-1.99	-1.55	-1.12	6.14E-08	Both
DALYs	Botswana	-2.53	-2.29	-2.04	1.64E-17	Female
DALYs	Botswana	-1.56	-1	-0.43	0.001142969	Male
Deaths	Botswana	-1.85	-1.48	-1.11	7.58E-09	Both
Deaths	Botswana	-2.56	-2.3	-2.04	6.44E-17	Female
Deaths	Botswana	-1.15	-0.69	-0.23	0.004712237	Male
DALYs	Brazil	0.19	0.4	0.62	0.000724221	Both
DALYs	Brazil	0.77	0.99	1.21	3.77E-10	Female
DALYs	Brazil	-0.11	0.12	0.35	0.298432429	Male
Deaths	Brazil	0.53	0.75	0.98	1.35E-07	Both
Deaths	Brazil	1.21	1.43	1.66	2.51E-13	Female
Deaths	Brazil	0.16	0.39	0.62	0.00185019	Male
DALYs	Brunei Darussalam	-0.14	-0.02	0.1	0.751392607	Both
DALYs	Brunei Darussalam	0.11	0.3	0.49	0.003456055	Female
DALYs	Brunei Darussalam	-0.38	-0.01	0.36	0.955596804	Male
Deaths	Brunei Darussalam	0.24	0.36	0.47	4.56E-07	Both
Deaths	Brunei Darussalam	0.21	0.49	0.78	0.001429884	Female
Deaths	Brunei Darussalam	0.07	0.53	0.99	0.0260809	Male
DALYs	Bulgaria	0.9	1.18	1.45	1.08E-09	Both
DALYs	Bulgaria	0.54	0.88	1.22	1.16E-05	Female
DALYs	Bulgaria	1.06	1.33	1.6	5.57E-11	Male
Deaths	Bulgaria	1.03	1.28	1.54	3.48E-11	Both
Deaths	Bulgaria	0.58	0.88	1.19	2.14E-06	Female
Deaths	Bulgaria	1.3	1.54	1.79	2.37E-13	Male
DALYs	Burkina Faso	-0.18	-0.07	0.05	0.23123408	Both
DALYs	Burkina Faso	-2.07	-1.78	-1.49	7.84E-13	Female
DALYs	Burkina Faso	0.99	1.08	1.17	1.48E-20	Male
Deaths	Burkina Faso	-0.02	0.05	0.13	0.1470499	Both
Deaths	Burkina Faso	-1.74	-1.53	-1.32	7.00E-15	Female
Deaths	Burkina Faso	1.07	1.2	1.32	3.82E-18	Male
DALYs	Burundi	-2.98	-2.7	-2.41	1.08E-17	Both
DALYs	Burundi	-2.67	-2.38	-2.1	2.71E-16	Female
DALYs	Burundi	-3.39	-3.1	-2.81	5.09E-19	Male
Deaths	Burundi	-2.77	-2.51	-2.25	6.13E-18	Both
Deaths	Burundi	-2.6	-2.32	-2.04	3.99E-16	Female
Deaths	Burundi	-3.06	-2.82	-2.57	9.74E-20	Male
DALYs	C?te d'Ivoire	-3.22	-2.83	-2.43	1.49E-14	Female
DALYs	Cabo Verde	0.7	1.11	1.51	4.97E-06	Both
DALYs	Cabo Verde	0.15	0.26	0.36	2.99E-05	Female
DALYs	Cabo Verde	0.84	1.38	1.93	1.51E-05	Male
Deaths	Cabo Verde	0.66	1.15	1.64	4.30E-05	Both
Deaths	Cabo Verde	0.43	0.57	0.71	5.64E-09	Female
Deaths	Cabo Verde	0.86	1.5	2.16	5.20E-05	Male
DALYs	Cambodia	0.49	0.58	0.68	2.50E-13	Both
DALYs	Cambodia	0.72	0.75	0.77	1.81E-30	Female
DALYs	Cambodia	0.46	0.59	0.73	1.30E-09	Male
Deaths	Cambodia	0.64	0.73	0.82	3.77E-16	Both
Deaths	Cambodia	0.76	0.79	0.82	6.26E-30	Female
Deaths	Cambodia	0.69	0.83	0.97	5.65E-13	Male
DALYs	Cameroon	-0.88	-0.75	-0.62	3.13E-12	Both
DALYs	Cameroon	-3.37	-2.99	-2.61	1.94E-15	Female
DALYs	Cameroon	0.36	0.5	0.65	6.22E-08	Male
Deaths	Cameroon	-0.93	-0.8	-0.67	3.34E-13	Both
Deaths	Cameroon	-3.3	-2.94	-2.58	6.19E-16	Female
Deaths	Cameroon	0.4	0.54	0.67	6.09E-09	Male
DALYs	Canada	-4.37	-4.02	-3.66	1.71E-19	Both
DALYs	Canada	-3.92	-3.56	-3.2	4.81E-18	Female
DALYs	Canada	-4.75	-4.38	-4.02	3.29E-20	Male
Deaths	Canada	-4.21	-3.85	-3.49	5.15E-19	Both

Deaths	Canada	-3.65	-3.31	-2.96	1.20E-17	Female
Deaths	Canada	-4.71	-4.33	-3.96	8.54E-20	Male
DALYs	Central African Republic	-1.47	-1.35	-1.24	4.09E-20	Both
DALYs	Central African Republic	-1.55	-1.42	-1.28	1.33E-18	Female
DALYs	Central African Republic	-1.39	-1.27	-1.15	7.40E-19	Male
Deaths	Central African Republic	-1.49	-1.38	-1.27	5.69E-21	Both
Deaths	Central African Republic	-1.53	-1.39	-1.26	1.50E-18	Female
Deaths	Central African Republic	-1.38	-1.26	-1.14	6.81E-19	Male
DALYs	Chad	-0.75	-0.65	-0.54	2.64E-13	Both
DALYs	Chad	-2.85	-2.56	-2.27	7.10E-17	Female
DALYs	Chad	0.1	0.25	0.41	0.002057134	Male
Deaths	Chad	-0.77	-0.67	-0.56	1.83E-13	Both
Deaths	Chad	-2.85	-2.57	-2.29	3.28E-17	Female
Deaths	Chad	0.16	0.31	0.45	0.00019513	Male
DALYs	Chile	-0.09	0.19	0.47	0.183492814	Both
DALYs	Chile	0.06	0.26	0.46	0.013546209	Female
DALYs	Chile	-0.23	0.09	0.42	0.563585722	Male
Deaths	Chile	-0.03	0.28	0.58	0.074278759	Both
Deaths	Chile	0.25	0.48	0.71	0.000240654	Female
Deaths	Chile	-0.31	0.06	0.42	0.754675531	Male
DALYs	China	-0.32	-0.27	-0.23	1.43E-12	Both
DALYs	China	-1	-0.96	-0.91	1.24E-27	Female
DALYs	China	-0.06	0.01	0.07	0.782341053	Male
Deaths	China	-0.35	-0.31	-0.28	3.37E-17	Both
Deaths	China	-0.99	-0.94	-0.89	4.91E-26	Female
Deaths	China	-0.05	0	0.06	0.910481428	Male
DALYs	Colombia	-2.01	-1.66	-1.32	1.55E-10	Both
DALYs	Colombia	-1.94	-1.57	-1.19	2.86E-09	Female
DALYs	Colombia	-1.91	-1.56	-1.22	5.53E-10	Male
Deaths	Colombia	-1.77	-1.41	-1.04	1.62E-08	Both
Deaths	Colombia	-1.64	-1.25	-0.85	5.81E-07	Female
Deaths	Colombia	-1.69	-1.32	-0.96	4.48E-08	Male
DALYs	Comoros	-2.19	-1.87	-1.54	3.09E-12	Both
DALYs	Comoros	-1.94	-1.67	-1.41	3.17E-13	Female
DALYs	Comoros	-2.28	-1.92	-1.55	1.99E-11	Male
Deaths	Comoros	-1.98	-1.71	-1.45	1.47E-13	Both
Deaths	Comoros	-1.8	-1.58	-1.36	8.63E-15	Female
Deaths	Comoros	-2.01	-1.72	-1.42	2.05E-12	Male
DALYs	Congo	-2.03	-1.74	-1.46	6.06E-13	Both
DALYs	Congo	-1.74	-1.54	-1.34	2.51E-15	Female
DALYs	Congo	-2.35	-2.01	-1.67	1.32E-12	Male
Deaths	Congo	-1.83	-1.56	-1.3	2.00E-12	Both
Deaths	Congo	-1.72	-1.52	-1.32	3.87E-15	Female
Deaths	Congo	-2.03	-1.72	-1.4	1.04E-11	Male
DALYs	Cook Islands	-1.41	-1.24	-1.06	2.46E-14	Both
DALYs	Cook Islands	-1.31	-1.15	-1	7.29E-15	Female
DALYs	Cook Islands	-1.25	-1.06	-0.87	4.68E-12	Male
Deaths	Cook Islands	-1.32	-1.18	-1.03	4.05E-16	Both
Deaths	Cook Islands	-1.08	-0.93	-0.78	5.51E-13	Female
Deaths	Cook Islands	-1.28	-1.14	-1	7.66E-16	Male
DALYs	Costa Rica	0.76	0.95	1.15	8.53E-11	Both
DALYs	Costa Rica	1.06	1.3	1.54	6.05E-12	Female
DALYs	Costa Rica	0.74	0.96	1.17	6.36E-10	Male
Deaths	Costa Rica	0.74	0.93	1.13	1.59E-10	Both
Deaths	Costa Rica	1.15	1.39	1.64	2.92E-12	Female
Deaths	Costa Rica	0.68	0.9	1.12	3.46E-09	Male
DALYs	Croatia	1.36	1.74	2.13	3.88E-10	Both
DALYs	Croatia	1.2	1.52	1.85	2.13E-10	Female
DALYs	Croatia	1.28	1.71	2.13	5.09E-09	Male
Deaths	Croatia	1.92	2.29	2.68	5.48E-13	Both
Deaths	Croatia	1.84	2.14	2.45	1.20E-14	Female
Deaths	Croatia	1.79	2.24	2.69	5.21E-11	Male
DALYs	Cuba	-1.15	-0.92	-0.69	6.92E-09	Both
DALYs	Cuba	-0.68	-0.5	-0.33	2.12E-06	Female
DALYs	Cuba	-1.26	-1	-0.73	2.49E-08	Male
Deaths	Cuba	-1.07	-0.85	-0.63	9.29E-09	Both
Deaths	Cuba	-0.54	-0.38	-0.22	5.55E-05	Female
Deaths	Cuba	-1.2	-0.94	-0.68	5.50E-08	Male

DALYs	Cyprus	-2.68	-2.4	-2.11	3.81E-16	Both
DALYs	Cyprus	-3.08	-2.81	-2.54	1.42E-18	Female
DALYs	Cyprus	-2.54	-2.24	-1.94	4.59E-15	Male
Deaths	Cyprus	-2.76	-2.49	-2.22	2.19E-17	Both
Deaths	Cyprus	-2.97	-2.73	-2.48	1.81E-19	Female
Deaths	Cyprus	-2.48	-2.19	-1.9	4.50E-15	Male
DALYs	Czechia	0.52	0.77	1.02	7.59E-07	Both
DALYs	Czechia	1.55	1.81	2.06	1.24E-14	Female
DALYs	Czechia	-0.12	0.16	0.45	0.240516062	Male
Deaths	Czechia	0.93	1.22	1.52	2.03E-09	Both
Deaths	Czechia	1.97	2.27	2.56	1.40E-15	Female
Deaths	Czechia	0.19	0.52	0.86	0.002995994	Male
DALYs	C么te d'Ivoire	-1.4	-1.25	-1.1	5.94E-16	Both
DALYs	C么te d'Ivoire	-0.76	-0.58	-0.4	3.59E-07	Male
Deaths	C么te d'Ivoire	-1.32	-1.18	-1.05	5.08E-17	Both
Deaths	C么te d'Ivoire	-3.13	-2.78	-2.42	1.53E-15	Female
Deaths	C么te d'Ivoire	-0.55	-0.39	-0.24	2.12E-05	Male
DALYs	Democratic People's Republic o	0.17	0.2	0.22	5.13E-15	Both
DALYs	Democratic People's Republic o	-0.25	-0.21	-0.18	7.20E-13	Female
DALYs	Democratic People's Republic o	0.14	0.17	0.21	2.32E-11	Male
Deaths	Democratic People's Republic o	-0.05	-0.02	0.01	0.231319902	Both
Deaths	Democratic People's Republic o	-0.51	-0.45	-0.38	3.09E-14	Female
Deaths	Democratic People's Republic o	0.04	0.08	0.12	0.000319348	Male
DALYs	Democratic Republic of the Con	-1.47	-1.25	-1.02	5.24E-12	Both
DALYs	Democratic Republic of the Con	-1.29	-1.09	-0.88	1.14E-11	Female
DALYs	Democratic Republic of the Con	-1.5	-1.29	-1.09	2.87E-13	Male
Deaths	Democratic Republic of the Con	-1.52	-1.3	-1.08	1.88E-12	Both
Deaths	Democratic Republic of the Con	-1.29	-1.09	-0.89	1.15E-11	Female
Deaths	Democratic Republic of the Con	-1.48	-1.29	-1.09	1.34E-13	Male
DALYs	Denmark	-2.19	-1.88	-1.58	5.36E-13	Both
DALYs	Denmark	-1.16	-0.86	-0.56	2.44E-06	Female
DALYs	Denmark	-2.71	-2.38	-2.06	8.36E-15	Male
Deaths	Denmark	-1.43	-1.17	-0.91	8.01E-10	Both
Deaths	Denmark	-0.55	-0.3	-0.04	0.023967386	Female
Deaths	Denmark	-1.94	-1.64	-1.35	5.16E-12	Male
DALYs	Djibouti	-1.5	-1.35	-1.2	5.09E-17	Both
DALYs	Djibouti	-1.93	-1.74	-1.55	1.58E-17	Female
DALYs	Djibouti	-1.34	-1.2	-1.06	8.81E-17	Male
Deaths	Djibouti	-1.39	-1.24	-1.1	2.40E-16	Both
Deaths	Djibouti	-1.78	-1.6	-1.41	8.12E-17	Female
Deaths	Djibouti	-1.19	-1.06	-0.94	2.22E-16	Male
DALYs	Dominica	-0.12	-0.08	-0.04	0.000664978	Both
DALYs	Dominica	-0.11	-0.03	0.05	0.414650815	Female
DALYs	Dominica	-0.43	-0.35	-0.28	2.86E-10	Male
Deaths	Dominica	-0.02	0.01	0.05	0.412638043	Both
Deaths	Dominica	0.07	0.15	0.23	0.001020292	Female
Deaths	Dominica	-0.4	-0.34	-0.27	2.49E-11	Male
DALYs	Dominican Republic	1.39	1.57	1.75	6.41E-17	Both
DALYs	Dominican Republic	0.89	1.06	1.23	3.69E-13	Female
DALYs	Dominican Republic	1.56	1.78	2	3.82E-16	Male
Deaths	Dominican Republic	1.48	1.65	1.82	3.98E-18	Both
Deaths	Dominican Republic	1.17	1.41	1.65	1.30E-12	Female
Deaths	Dominican Republic	1.62	1.83	2.03	2.91E-17	Male
DALYs	Ecuador	0.3	0.65	1.02	0.000849428	Both
DALYs	Ecuador	0.16	0.5	0.85	0.00595912	Female
DALYs	Ecuador	0.42	0.8	1.17	0.000159675	Male
Deaths	Ecuador	0.61	0.99	1.37	9.21E-06	Both
Deaths	Ecuador	0.42	0.77	1.13	0.000100735	Female
Deaths	Ecuador	0.82	1.21	1.61	8.34E-07	Male
DALYs	Egypt	0.49	0.59	0.69	1.32E-12	Both
DALYs	Egypt	0.13	0.2	0.27	2.70E-06	Female
DALYs	Egypt	0.36	0.48	0.59	3.30E-09	Male
Deaths	Egypt	0.54	0.68	0.82	1.38E-10	Both
Deaths	Egypt	0.3	0.4	0.49	4.15E-09	Female
Deaths	Egypt	0.26	0.43	0.59	9.70E-06	Male
DALYs	El Salvador	-0.27	-0.11	0.06	0.192253721	Both
DALYs	El Salvador	-0.3	-0.22	-0.15	8.10E-07	Female
DALYs	El Salvador	-0.06	0.15	0.36	0.156510059	Male

Deaths	El Salvador	-0.21	-0.05	0.11	0.535078314	Both
Deaths	El Salvador	-0.16	-0.07	0.02	0.107484931	Female
Deaths	El Salvador	-0.04	0.17	0.37	0.106833764	Male
DALYs	Equatorial Guinea	-1.44	-1.14	-0.85	1.25E-08	Both
DALYs	Equatorial Guinea	-0.29	-0.21	-0.13	7.68E-06	Female
DALYs	Equatorial Guinea	-2.12	-1.66	-1.2	5.11E-08	Male
Deaths	Equatorial Guinea	-0.91	-0.67	-0.44	3.01E-06	Both
Deaths	Equatorial Guinea	-0.04	0.04	0.12	0.271315198	Female
Deaths	Equatorial Guinea	-1.52	-1.13	-0.74	2.42E-06	Male
DALYs	Eritrea	-1.61	-1.42	-1.23	4.31E-15	Both
DALYs	Eritrea	-1.31	-1.13	-0.96	1.73E-13	Female
DALYs	Eritrea	-1.84	-1.64	-1.44	6.17E-16	Male
Deaths	Eritrea	-1.42	-1.23	-1.03	3.83E-13	Both
Deaths	Eritrea	-1.18	-0.98	-0.79	6.20E-11	Female
Deaths	Eritrea	-1.64	-1.44	-1.24	1.20E-14	Male
DALYs	Estonia	-0.89	-0.41	0.06	0.085965035	Both
DALYs	Estonia	-1.03	-0.64	-0.24	0.002742454	Female
DALYs	Estonia	-0.96	-0.45	0.06	0.083777845	Male
Deaths	Estonia	-0.4	0.02	0.44	0.931603962	Both
Deaths	Estonia	-0.5	-0.15	0.2	0.39722963	Female
Deaths	Estonia	-0.53	-0.07	0.39	0.759377228	Male
DALYs	Eswatini	-0.82	-0.6	-0.39	3.06E-06	Both
DALYs	Eswatini	-2.01	-1.88	-1.74	2.53E-22	Female
DALYs	Eswatini	0	0.24	0.48	0.052827536	Male
Deaths	Eswatini	-0.92	-0.74	-0.56	4.85E-09	Both
Deaths	Eswatini	-1.94	-1.75	-1.55	3.65E-17	Female
Deaths	Eswatini	0	0.14	0.28	0.050117946	Male
DALYs	Ethiopia	-2.73	-2.45	-2.16	1.76E-16	Both
DALYs	Ethiopia	-2.26	-1.97	-1.67	8.45E-14	Female
DALYs	Ethiopia	-2.96	-2.67	-2.38	2.38E-17	Male
Deaths	Ethiopia	-2.32	-2.05	-1.77	5.74E-15	Both
Deaths	Ethiopia	-2.05	-1.74	-1.44	3.11E-12	Female
Deaths	Ethiopia	-2.42	-2.16	-1.9	3.36E-16	Male
DALYs	Fiji	-0.47	-0.39	-0.31	2.51E-10	Both
DALYs	Fiji	-0.52	-0.4	-0.28	1.87E-07	Female
DALYs	Fiji	-0.47	-0.31	-0.15	0.000395988	Male
Deaths	Fiji	-0.42	-0.33	-0.24	9.12E-08	Both
Deaths	Fiji	-0.47	-0.36	-0.25	2.66E-07	Female
Deaths	Fiji	-0.36	-0.17	0.02	0.077201435	Male
DALYs	Finland	-2.16	-1.93	-1.71	1.84E-16	Both
DALYs	Finland	-1.11	-0.88	-0.65	1.26E-08	Female
DALYs	Finland	-2.89	-2.66	-2.44	3.17E-20	Male
Deaths	Finland	-1.75	-1.54	-1.32	1.30E-14	Both
Deaths	Finland	-0.83	-0.6	-0.37	1.12E-05	Female
Deaths	Finland	-2.6	-2.39	-2.18	1.26E-19	Male
DALYs	France	-2.71	-2.4	-2.09	2.10E-15	Both
DALYs	France	-1.78	-1.56	-1.34	1.54E-14	Female
DALYs	France	-3.15	-2.8	-2.46	6.79E-16	Male
Deaths	France	-2.66	-2.33	-2	1.86E-14	Both
Deaths	France	-2.02	-1.78	-1.54	4.34E-15	Female
Deaths	France	-3.07	-2.71	-2.34	8.43E-15	Male
DALYs	Gabon	-1.26	-1.12	-0.98	8.51E-16	Both
DALYs	Gabon	-1.75	-1.59	-1.42	5.27E-18	Female
DALYs	Gabon	-1.14	-1	-0.86	1.71E-14	Male
Deaths	Gabon	-1.22	-1.07	-0.92	2.20E-14	Both
Deaths	Gabon	-1.69	-1.52	-1.35	4.43E-17	Female
Deaths	Gabon	-0.99	-0.84	-0.7	2.05E-12	Male
DALYs	Gambia	-0.67	-0.47	-0.27	4.41E-05	Both
DALYs	Gambia	-2.64	-2.3	-1.95	1.14E-13	Female
DALYs	Gambia	0.49	0.63	0.78	9.69E-10	Male
Deaths	Gambia	-0.66	-0.49	-0.32	2.77E-06	Both
Deaths	Gambia	-2.54	-2.23	-1.92	1.34E-14	Female
Deaths	Gambia	0.6	0.71	0.81	4.63E-14	Male
DALYs	Georgia	4.88	5.72	6.56	2.04E-14	Both
DALYs	Georgia	3.2	3.63	4.07	1.27E-16	Female
DALYs	Georgia	5.11	6.09	7.09	2.27E-13	Male
Deaths	Georgia	4.44	5.2	5.97	2.46E-14	Both
Deaths	Georgia	3.14	3.59	4.04	4.88E-16	Female

Deaths	Georgia	4.44	5.36	6.29	1.02E-12	Male
DALYs	Germany	-1.58	-1.39	-1.2	1.07E-14	Both
DALYs	Germany	-0.74	-0.6	-0.46	1.49E-09	Female
DALYs	Germany	-2.49	-2.23	-1.98	1.26E-16	Male
Deaths	Germany	-1.68	-1.47	-1.26	3.07E-14	Both
Deaths	Germany	-0.74	-0.59	-0.45	3.38E-09	Female
Deaths	Germany	-2.78	-2.49	-2.21	6.53E-17	Male
DALYs	Ghana	-3.06	-2.46	-1.85	6.52E-09	Both
DALYs	Ghana	-4.77	-3.91	-3.04	7.62E-10	Female
DALYs	Ghana	-1.21	-0.84	-0.46	0.000105975	Male
Deaths	Ghana	-3.02	-2.43	-1.83	4.87E-09	Both
Deaths	Ghana	-4.89	-4.02	-3.14	5.41E-10	Female
Deaths	Ghana	-0.83	-0.52	-0.21	0.001776919	Male
DALYs	Greece	0.1	0.4	0.69	0.009730092	Both
DALYs	Greece	0.14	0.4	0.67	0.004177893	Female
DALYs	Greece	0.07	0.37	0.67	0.018596713	Male
Deaths	Greece	0.1	0.42	0.73	0.01107214	Both
Deaths	Greece	0.28	0.57	0.86	0.000440522	Female
Deaths	Greece	0.01	0.32	0.63	0.042331393	Male
DALYs	Greenland	-2.5	-2.24	-1.99	1.01E-16	Both
DALYs	Greenland	-3.32	-2.77	-2.21	8.46E-11	Female
DALYs	Greenland	-2.07	-1.97	-1.87	1.32E-26	Male
Deaths	Greenland	-2.5	-2.19	-1.89	1.20E-14	Both
Deaths	Greenland	-3.28	-2.7	-2.12	3.38E-10	Female
Deaths	Greenland	-1.91	-1.81	-1.71	6.05E-25	Male
DALYs	Grenada	-0.76	-0.34	0.09	0.113902404	Both
DALYs	Grenada	-0.77	-0.35	0.08	0.103635588	Female
DALYs	Grenada	-1.11	-0.47	0.17	0.144333805	Male
Deaths	Grenada	-0.91	-0.41	0.1	0.111960924	Both
Deaths	Grenada	-0.63	-0.19	0.25	0.383294012	Female
Deaths	Grenada	-1.25	-0.44	0.37	0.272369387	Male
DALYs	Guam	-4.09	-3.8	-3.51	2.66E-21	Both
DALYs	Guam	-4.46	-3.45	-2.43	2.07E-07	Female
DALYs	Guam	-4.1	-3.79	-3.48	2.43E-20	Male
Deaths	Guam	-4.69	-4.37	-4.05	7.81E-22	Both
Deaths	Guam	-5.08	-4.11	-3.14	2.99E-09	Female
Deaths	Guam	-4.68	-4.32	-3.95	4.67E-20	Male
DALYs	Guatemala	-0.89	-0.7	-0.52	2.58E-08	Both
DALYs	Guatemala	0	0.12	0.25	0.055613809	Female
DALYs	Guatemala	-0.87	-0.63	-0.39	1.14E-05	Male
Deaths	Guatemala	-0.99	-0.77	-0.54	1.78E-07	Both
Deaths	Guatemala	0.07	0.2	0.34	0.005069162	Female
Deaths	Guatemala	-0.69	-0.4	-0.11	0.009332556	Male
DALYs	Guinea	-0.54	-0.44	-0.35	3.15E-10	Both
DALYs	Guinea	-3.15	-2.86	-2.57	5.07E-18	Female
DALYs	Guinea	0.85	0.97	1.09	3.12E-16	Male
Deaths	Guinea	-0.59	-0.5	-0.42	2.46E-12	Both
Deaths	Guinea	-3.1	-2.83	-2.56	1.37E-18	Female
Deaths	Guinea	0.82	0.95	1.08	3.21E-15	Male
DALYs	Guinea-Bissau	-0.94	-0.84	-0.75	7.54E-17	Both
DALYs	Guinea-Bissau	-2.7	-2.43	-2.16	3.66E-17	Female
DALYs	Guinea-Bissau	0	0.05	0.1	0.04323384	Male
Deaths	Guinea-Bissau	-0.98	-0.89	-0.79	4.82E-17	Both
Deaths	Guinea-Bissau	-2.64	-2.37	-2.1	6.46E-17	Female
Deaths	Guinea-Bissau	0.11	0.16	0.2	3.24E-07	Male
DALYs	Guyana	0.75	1.09	1.44	4.57E-07	Both
DALYs	Guyana	0.74	1.02	1.3	3.96E-08	Female
DALYs	Guyana	0.77	1.17	1.57	1.67E-06	Male
Deaths	Guyana	0.65	1.01	1.37	2.95E-06	Both
Deaths	Guyana	0.7	1.01	1.32	3.28E-07	Female
Deaths	Guyana	0.64	1.05	1.47	1.52E-05	Male
DALYs	Haiti	-0.49	-0.39	-0.29	8.74E-09	Both
DALYs	Haiti	-0.61	-0.53	-0.46	8.67E-15	Female
DALYs	Haiti	-0.44	-0.27	-0.11	0.001839563	Male
Deaths	Haiti	-0.43	-0.37	-0.31	5.24E-13	Both
Deaths	Haiti	-0.81	-0.71	-0.61	1.05E-14	Female
Deaths	Haiti	-0.28	-0.16	-0.04	0.013060853	Male
DALYs	Honduras	1.22	1.39	1.56	3.37E-16	Both

DALYs	Honduras	1.02	1.25	1.48	6.79E-12	Female
DALYs	Honduras	1.27	1.5	1.74	1.71E-13	Male
Deaths	Honduras	1.57	1.77	1.97	2.93E-17	Both
Deaths	Honduras	1.41	1.77	2.14	7.38E-11	Female
Deaths	Honduras	1.51	1.78	2.05	1.03E-13	Male
DALYs	Hungary	-0.43	-0.28	-0.14	0.000481884	Both
DALYs	Hungary	-0.38	-0.24	-0.09	0.002420694	Female
DALYs	Hungary	-0.48	-0.3	-0.11	0.002602592	Male
Deaths	Hungary	-0.12	0.02	0.16	0.751702896	Both
Deaths	Hungary	-0.07	0.06	0.18	0.362214957	Female
Deaths	Hungary	-0.15	0.04	0.23	0.670996483	Male
DALYs	Iceland	-2.06	-1.86	-1.66	1.99E-17	Both
DALYs	Iceland	-2.38	-2.12	-1.87	4.83E-16	Female
DALYs	Iceland	-2.04	-1.84	-1.65	1.60E-17	Male
Deaths	Iceland	-1.85	-1.63	-1.4	8.08E-15	Both
Deaths	Iceland	-2.08	-1.82	-1.56	2.82E-14	Female
Deaths	Iceland	-1.87	-1.63	-1.39	3.96E-14	Male
DALYs	India	-0.11	-0.02	0.07	0.602064396	Both
DALYs	India	0.05	0.14	0.23	0.00351167	Female
DALYs	India	-0.06	0.04	0.14	0.420381085	Male
Deaths	India	-0.16	-0.06	0.04	0.221375235	Both
Deaths	India	-0.03	0.07	0.17	0.178351957	Female
Deaths	India	-0.08	0.03	0.13	0.633852615	Male
DALYs	Indonesia	1.06	1.1	1.14	2.34E-30	Both
DALYs	Indonesia	0.41	0.48	0.56	2.11E-13	Female
DALYs	Indonesia	1.45	1.49	1.53	2.66E-34	Male
Deaths	Indonesia	1.21	1.26	1.3	3.30E-31	Both
Deaths	Indonesia	0.61	0.68	0.75	5.30E-18	Female
Deaths	Indonesia	1.67	1.7	1.74	1.38E-36	Male
DALYs	Iran (Islamic Republic of)	-0.49	-0.38	-0.26	2.33E-07	Both
DALYs	Iran (Islamic Republic of)	-0.64	-0.4	-0.17	0.001473732	Female
DALYs	Iran (Islamic Republic of)	-0.4	-0.28	-0.15	9.02E-05	Male
Deaths	Iran (Islamic Republic of)	-0.42	-0.31	-0.21	1.95E-06	Both
Deaths	Iran (Islamic Republic of)	-0.93	-0.69	-0.45	2.90E-06	Female
Deaths	Iran (Islamic Republic of)	-0.31	-0.19	-0.07	0.002809412	Male
DALYs	Iraq	-0.5	-0.37	-0.24	3.52E-06	Both
DALYs	Iraq	-0.8	-0.68	-0.57	6.80E-13	Female
DALYs	Iraq	-0.34	-0.21	-0.07	0.005632359	Male
Deaths	Iraq	-0.06	0.02	0.11	0.592881814	Both
Deaths	Iraq	-0.5	-0.42	-0.34	1.60E-11	Female
Deaths	Iraq	0.19	0.29	0.38	1.55E-06	Male
DALYs	Ireland	-2.64	-2.26	-1.87	1.68E-12	Both
DALYs	Ireland	-1.77	-1.49	-1.2	2.75E-11	Female
DALYs	Ireland	-3.18	-2.74	-2.29	7.39E-13	Male
Deaths	Ireland	-2.11	-1.76	-1.4	8.62E-11	Both
Deaths	Ireland	-1.25	-0.98	-0.72	3.53E-08	Female
Deaths	Ireland	-2.77	-2.34	-1.91	1.07E-11	Male
DALYs	Israel	-1.83	-1.61	-1.38	1.02E-14	Both
DALYs	Israel	-2.32	-2.04	-1.77	6.63E-15	Female
DALYs	Israel	-1.68	-1.47	-1.27	1.00E-14	Male
Deaths	Israel	-1.73	-1.52	-1.31	1.11E-14	Both
Deaths	Israel	-2.11	-1.84	-1.58	2.33E-14	Female
Deaths	Israel	-1.53	-1.34	-1.15	1.65E-14	Male
DALYs	Italy	-1.89	-1.54	-1.2	8.69E-10	Both
DALYs	Italy	-0.81	-0.51	-0.21	0.001846252	Female
DALYs	Italy	-2.35	-1.97	-1.59	2.99E-11	Male
Deaths	Italy	-1.61	-1.23	-0.85	4.42E-07	Both
Deaths	Italy	-0.6	-0.27	0.07	0.117417598	Female
Deaths	Italy	-2.09	-1.66	-1.23	1.37E-08	Male
DALYs	Jamaica	1.11	1.45	1.79	1.89E-09	Both
DALYs	Jamaica	-0.55	-0.22	0.11	0.182290511	Female
DALYs	Jamaica	1.94	2.42	2.9	3.46E-11	Male
Deaths	Jamaica	1.01	1.32	1.63	1.64E-09	Both
Deaths	Jamaica	-0.61	-0.27	0.06	0.101570511	Female
Deaths	Jamaica	1.93	2.39	2.85	1.62E-11	Male
DALYs	Japan	1.66	1.81	1.97	2.49E-20	Both
DALYs	Japan	2.17	2.33	2.49	3.98E-23	Female
DALYs	Japan	1.14	1.29	1.44	8.51E-17	Male

Deaths	Japan	1.65	1.8	1.96	4.11E-20	Both
Deaths	Japan	2.44	2.62	2.81	1.04E-22	Female
Deaths	Japan	0.85	1.01	1.16	1.33E-13	Male
DALYs	Jordan	-2.01	-1.76	-1.51	1.51E-14	Both
DALYs	Jordan	-1.85	-1.65	-1.45	2.84E-16	Female
DALYs	Jordan	-2.16	-1.88	-1.59	8.33E-14	Male
Deaths	Jordan	-1.75	-1.54	-1.33	9.45E-15	Both
Deaths	Jordan	-1.71	-1.52	-1.33	7.73E-16	Female
Deaths	Jordan	-1.92	-1.66	-1.4	1.94E-13	Male
DALYs	Kazakhstan	0.41	0.81	1.2	0.00024043	Both
DALYs	Kazakhstan	0.73	0.9	1.07	1.65E-11	Female
DALYs	Kazakhstan	0.24	0.71	1.18	0.004117656	Male
Deaths	Kazakhstan	0.67	0.97	1.28	4.12E-07	Both
Deaths	Kazakhstan	0.78	0.95	1.11	2.95E-12	Female
Deaths	Kazakhstan	0.5	0.87	1.24	4.00E-05	Male
DALYs	Kenya	-0.16	-0.08	0	0.060523894	Both
DALYs	Kenya	-0.49	-0.33	-0.17	0.000305906	Female
DALYs	Kenya	-0.11	0.11	0.33	0.319483559	Male
Deaths	Kenya	-0.16	-0.09	-0.03	0.003193188	Both
Deaths	Kenya	-0.43	-0.27	-0.1	0.002500315	Female
Deaths	Kenya	-0.1	0.08	0.26	0.361543315	Male
DALYs	Kiribati	-0.85	-0.78	-0.7	7.15E-19	Both
DALYs	Kiribati	-0.54	-0.41	-0.27	8.72E-07	Female
DALYs	Kiribati	-0.95	-0.83	-0.71	2.19E-14	Male
Deaths	Kiribati	-0.66	-0.6	-0.53	5.70E-18	Both
Deaths	Kiribati	-0.38	-0.23	-0.09	0.002116356	Female
Deaths	Kiribati	-0.75	-0.64	-0.53	2.28E-12	Male
DALYs	Kuwait	0.02	0.36	0.69	0.03995952	Both
DALYs	Kuwait	-0.31	-0.01	0.3	0.963578696	Female
DALYs	Kuwait	0.14	0.49	0.85	0.008547979	Male
Deaths	Kuwait	0.52	0.86	1.2	1.39E-05	Both
Deaths	Kuwait	0.25	0.51	0.76	0.000317683	Female
Deaths	Kuwait	0.48	0.85	1.22	5.74E-05	Male
DALYs	Kyrgyzstan	-0.58	-0.18	0.21	0.351768361	Both
DALYs	Kyrgyzstan	-0.56	-0.13	0.3	0.547224054	Female
DALYs	Kyrgyzstan	-0.69	-0.33	0.03	0.07205378	Male
Deaths	Kyrgyzstan	0.17	0.56	0.94	0.006143774	Both
Deaths	Kyrgyzstan	0.31	0.77	1.24	0.001998907	Female
Deaths	Kyrgyzstan	-0.16	0.18	0.52	0.28653327	Male
DALYs	Lao People's Democratic Republ	0.57	0.61	0.65	4.71E-23	Both
DALYs	Lao People's Democratic Republ	1.01	1.08	1.15	5.35E-24	Female
DALYs	Lao People's Democratic Republ	0.32	0.36	0.4	1.87E-17	Male
Deaths	Lao People's Democratic Republ	0.6	0.65	0.69	6.36E-22	Both
Deaths	Lao People's Democratic Republ	0.85	0.91	0.97	1.29E-23	Female
Deaths	Lao People's Democratic Republ	0.42	0.47	0.51	2.60E-18	Male
DALYs	Latvia	-1.2	-0.7	-0.2	0.008110964	Both
DALYs	Latvia	-0.94	-0.56	-0.18	0.005397384	Female
DALYs	Latvia	-1.46	-0.91	-0.36	0.002226639	Male
Deaths	Latvia	-0.85	-0.43	0	0.047863939	Both
Deaths	Latvia	-0.49	-0.18	0.12	0.231041251	Female
Deaths	Latvia	-1.18	-0.7	-0.21	0.006830899	Male
DALYs	Lebanon	-0.32	-0.16	-0.01	0.039344785	Both
DALYs	Lebanon	-1.55	-1.38	-1.21	7.03E-16	Female
DALYs	Lebanon	0.09	0.38	0.67	0.011421421	Male
Deaths	Lebanon	-0.19	-0.09	0.02	0.093209479	Both
Deaths	Lebanon	-1.59	-1.39	-1.19	2.91E-14	Female
Deaths	Lebanon	0.23	0.47	0.71	0.000395368	Male
DALYs	Lesotho	0.22	0.28	0.35	5.65E-10	Both
DALYs	Lesotho	-0.68	-0.48	-0.27	5.60E-05	Female
DALYs	Lesotho	0.6	0.77	0.93	1.73E-10	Male
Deaths	Lesotho	0.07	0.12	0.17	6.22E-05	Both
Deaths	Lesotho	-0.51	-0.35	-0.2	7.27E-05	Female
Deaths	Lesotho	0.4	0.52	0.65	3.84E-09	Male
DALYs	Liberia	-1.32	-1.1	-0.88	6.95E-11	Both
DALYs	Liberia	-3.07	-2.76	-2.44	1.30E-16	Female
DALYs	Liberia	-0.36	-0.18	0	0.056064236	Male
Deaths	Liberia	-1.18	-1.01	-0.83	3.71E-12	Both
Deaths	Liberia	-2.95	-2.66	-2.37	2.39E-17	Female

Deaths	Liberia	-0.17	-0.03	0.12	0.705167012	Male
DALYs	Libya	0.32	0.43	0.55	2.79E-08	Both
DALYs	Libya	-0.04	0.01	0.06	0.760444691	Female
DALYs	Libya	0.52	0.67	0.82	7.21E-10	Male
Deaths	Libya	0.39	0.51	0.62	9.13E-10	Both
Deaths	Libya	0	0.04	0.09	0.052149852	Female
Deaths	Libya	0.58	0.73	0.88	9.11E-11	Male
DALYs	Lithuania	0.57	0.97	1.37	2.93E-05	Both
DALYs	Lithuania	0.02	0.44	0.87	0.042144261	Female
DALYs	Lithuania	0.72	1.16	1.59	7.32E-06	Male
Deaths	Lithuania	0.81	1.17	1.53	3.36E-07	Both
Deaths	Lithuania	0.27	0.72	1.18	0.002994304	Female
Deaths	Lithuania	1.02	1.39	1.77	2.46E-08	Male
DALYs	Luxembourg	-2.66	-2.4	-2.15	1.57E-17	Both
DALYs	Luxembourg	-1.55	-1.36	-1.17	1.15E-14	Female
DALYs	Luxembourg	-3.33	-3.04	-2.75	8.89E-19	Male
Deaths	Luxembourg	-2.24	-2	-1.76	4.40E-16	Both
Deaths	Luxembourg	-1.26	-1.07	-0.88	3.75E-12	Female
Deaths	Luxembourg	-2.96	-2.68	-2.4	1.14E-17	Male
DALYs	Madagascar	-1.6	-1.45	-1.29	1.40E-17	Both
DALYs	Madagascar	-1.75	-1.58	-1.41	2.58E-17	Female
DALYs	Madagascar	-1.47	-1.32	-1.17	6.50E-17	Male
Deaths	Madagascar	-1.67	-1.49	-1.31	3.71E-16	Both
Deaths	Madagascar	-1.81	-1.63	-1.45	5.10E-17	Female
Deaths	Madagascar	-1.5	-1.31	-1.12	2.31E-14	Male
DALYs	Malawi	-0.94	-0.79	-0.65	7.51E-12	Both
DALYs	Malawi	-1.24	-1.02	-0.8	5.11E-10	Female
DALYs	Malawi	-0.76	-0.55	-0.33	1.57E-05	Male
Deaths	Malawi	-0.94	-0.81	-0.68	3.39E-13	Both
Deaths	Malawi	-1.18	-0.95	-0.73	2.19E-09	Female
Deaths	Malawi	-0.69	-0.52	-0.34	1.56E-06	Male
DALYs	Malaysia	-0.64	-0.44	-0.24	9.09E-05	Both
DALYs	Malaysia	-1.16	-0.92	-0.68	1.42E-08	Female
DALYs	Malaysia	-0.59	-0.38	-0.16	0.001315956	Male
Deaths	Malaysia	-0.57	-0.31	-0.05	0.020368845	Both
Deaths	Malaysia	-1.04	-0.74	-0.43	3.21E-05	Female
Deaths	Malaysia	-0.54	-0.25	0.05	0.099176838	Male
DALYs	Maldives	-1.49	-1.34	-1.18	7.09E-17	Both
DALYs	Maldives	-0.51	-0.32	-0.12	0.002891733	Female
DALYs	Maldives	-1.59	-1.42	-1.25	2.29E-16	Male
Deaths	Maldives	-1.09	-0.94	-0.8	1.27E-13	Both
Deaths	Maldives	-0.21	0.03	0.27	0.790439411	Female
Deaths	Maldives	-1.08	-0.94	-0.79	1.77E-13	Male
DALYs	Mali	-1.28	-1.1	-0.91	2.11E-12	Both
DALYs	Mali	-3.04	-2.75	-2.45	2.67E-17	Female
DALYs	Mali	-0.07	0.07	0.2	0.313760774	Male
Deaths	Mali	-1.06	-0.91	-0.76	6.04E-13	Both
Deaths	Mali	-2.96	-2.68	-2.41	6.48E-18	Female
Deaths	Mali	0.16	0.24	0.33	4.12E-06	Male
DALYs	Malta	-1.71	-1.49	-1.26	6.23E-14	Both
DALYs	Malta	-1.33	-1.19	-1.06	5.65E-17	Female
DALYs	Malta	-1.98	-1.73	-1.47	7.17E-14	Male
Deaths	Malta	-1.7	-1.47	-1.25	1.33E-13	Both
Deaths	Malta	-1.34	-1.18	-1.01	1.06E-14	Female
Deaths	Malta	-1.93	-1.67	-1.42	1.43E-13	Male
DALYs	Marshall Islands	-0.55	-0.47	-0.4	1.95E-13	Both
DALYs	Marshall Islands	-0.87	-0.61	-0.35	4.64E-05	Female
DALYs	Marshall Islands	-0.59	-0.44	-0.3	1.27E-06	Male
Deaths	Marshall Islands	-0.66	-0.58	-0.5	7.05E-15	Both
Deaths	Marshall Islands	-0.92	-0.66	-0.4	1.63E-05	Female
Deaths	Marshall Islands	-0.73	-0.55	-0.36	1.78E-06	Male
DALYs	Mauritania	-2.12	-1.88	-1.64	1.18E-15	Both
DALYs	Mauritania	-3.35	-3.11	-2.86	8.33E-21	Female
DALYs	Mauritania	-1.45	-1.22	-0.98	3.27E-11	Male
Deaths	Mauritania	-1.88	-1.65	-1.42	1.04E-14	Both
Deaths	Mauritania	-3.29	-3.03	-2.78	2.43E-20	Female
Deaths	Mauritania	-1.11	-0.91	-0.71	6.73E-10	Male
DALYs	Mauritius	-0.79	-0.6	-0.41	7.15E-07	Both

DALYs	Mauritius	-1.3	-1.02	-0.73	6.73E-08	Female
DALYs	Mauritius	-0.66	-0.46	-0.27	3.37E-05	Male
Deaths	Mauritius	-0.67	-0.48	-0.29	1.78E-05	Both
Deaths	Mauritius	-1.1	-0.83	-0.57	6.60E-07	Female
Deaths	Mauritius	-0.57	-0.38	-0.18	0.000444359	Male
DALYs	Mexico	-0.49	-0.37	-0.25	7.03E-07	Both
DALYs	Mexico	-0.47	-0.36	-0.25	3.19E-07	Female
DALYs	Mexico	-0.43	-0.29	-0.14	0.000305026	Male
Deaths	Mexico	-0.38	-0.28	-0.17	1.06E-05	Both
Deaths	Mexico	-0.35	-0.24	-0.13	0.000145343	Female
Deaths	Mexico	-0.27	-0.15	-0.03	0.019132656	Male
DALYs	Micronesia (Federated States o	-0.72	-0.61	-0.51	2.37E-12	Both
DALYs	Micronesia (Federated States o	-1.05	-0.9	-0.75	8.98E-13	Female
DALYs	Micronesia (Federated States o	-0.45	-0.38	-0.31	5.14E-12	Male
Deaths	Micronesia (Federated States o	-0.79	-0.67	-0.55	1.11E-11	Both
Deaths	Micronesia (Federated States o	-1.11	-0.95	-0.79	1.19E-12	Female
Deaths	Micronesia (Federated States o	-0.43	-0.34	-0.26	3.50E-09	Male
DALYs	Monaco	-0.68	-0.64	-0.61	1.66E-25	Both
DALYs	Monaco	-0.59	-0.53	-0.47	7.01E-17	Female
DALYs	Monaco	-0.83	-0.8	-0.77	2.49E-29	Male
Deaths	Monaco	-0.51	-0.48	-0.45	7.35E-23	Both
Deaths	Monaco	-0.34	-0.31	-0.28	1.31E-17	Female
Deaths	Monaco	-0.74	-0.7	-0.67	2.06E-26	Male
DALYs	Mongolia	-0.63	-0.38	-0.12	0.005774078	Both
DALYs	Mongolia	-0.82	-0.65	-0.49	1.11E-08	Female
DALYs	Mongolia	-0.51	-0.15	0.2	0.387028913	Male
Deaths	Mongolia	-0.54	-0.3	-0.05	0.018358775	Both
Deaths	Mongolia	-0.54	-0.38	-0.23	1.82E-05	Female
Deaths	Mongolia	-0.57	-0.2	0.18	0.293785371	Male
DALYs	Montenegro	0.54	0.72	0.89	5.81E-09	Both
DALYs	Montenegro	0.73	0.92	1.12	1.60E-10	Female
DALYs	Montenegro	0.46	0.65	0.84	1.01E-07	Male
Deaths	Montenegro	1.02	1.22	1.42	4.89E-13	Both
Deaths	Montenegro	0.95	1.19	1.42	5.97E-11	Female
Deaths	Montenegro	1.01	1.22	1.42	1.07E-12	Male
DALYs	Morocco	0.3	0.46	0.63	4.20E-06	Both
DALYs	Morocco	0.48	0.58	0.68	1.49E-12	Female
DALYs	Morocco	0.16	0.4	0.65	0.002279121	Male
Deaths	Morocco	0.46	0.7	0.93	1.41E-06	Both
Deaths	Morocco	0.68	0.82	0.96	9.80E-13	Female
Deaths	Morocco	0.2	0.58	0.97	0.004126649	Male
DALYs	Mozambique	0.24	0.37	0.5	2.36E-06	Both
DALYs	Mozambique	-0.83	-0.64	-0.45	1.88E-07	Female
DALYs	Mozambique	0.84	0.97	1.1	4.39E-15	Male
Deaths	Mozambique	-0.01	0.11	0.23	0.070172733	Both
Deaths	Mozambique	-0.79	-0.6	-0.41	5.45E-07	Female
Deaths	Mozambique	0.61	0.72	0.82	2.66E-14	Male
DALYs	Myanmar	0.19	0.24	0.3	2.36E-09	Both
DALYs	Myanmar	0.42	0.53	0.65	5.04E-10	Female
DALYs	Myanmar	0.23	0.27	0.3	5.26E-16	Male
Deaths	Myanmar	0.43	0.47	0.51	4.18E-20	Both
Deaths	Myanmar	0.57	0.67	0.76	1.15E-14	Female
Deaths	Myanmar	0.51	0.52	0.54	4.00E-31	Male
DALYs	Namibia	-1.31	-1.09	-0.87	9.37E-11	Both
DALYs	Namibia	-2.31	-2.04	-1.77	3.90E-15	Female
DALYs	Namibia	-0.66	-0.43	-0.19	0.001019304	Male
Deaths	Namibia	-1.16	-0.96	-0.75	2.91E-10	Both
Deaths	Namibia	-2.17	-1.89	-1.61	4.13E-14	Female
Deaths	Namibia	-0.39	-0.2	-0.02	0.032469321	Male
DALYs	Nauru	-1.16	-0.89	-0.61	5.32E-07	Both
DALYs	Nauru	-1.56	-1.21	-0.86	9.95E-08	Female
DALYs	Nauru	-0.65	-0.48	-0.31	4.54E-06	Male
Deaths	Nauru	-1.13	-0.87	-0.61	1.96E-07	Both
Deaths	Nauru	-1.64	-1.29	-0.94	4.08E-08	Female
Deaths	Nauru	-0.63	-0.49	-0.35	5.39E-08	Male
DALYs	Nepal	0.89	1.1	1.3	8.71E-12	Both
DALYs	Nepal	1.01	1.15	1.3	7.68E-16	Female
DALYs	Nepal	0.95	1.2	1.45	1.25E-10	Male

Deaths	Nepal	1.23	1.4	1.58	3.17E-16	Both
Deaths	Nepal	1.35	1.47	1.59	1.15E-20	Female
Deaths	Nepal	1.25	1.47	1.68	2.57E-14	Male
DALYs	Netherlands	-3.34	-2.92	-2.49	3.83E-14	Both
DALYs	Netherlands	-1.35	-0.99	-0.62	7.25E-06	Female
DALYs	Netherlands	-4.34	-3.87	-3.39	8.93E-16	Male
Deaths	Netherlands	-2.83	-2.43	-2.04	5.30E-13	Both
Deaths	Netherlands	-1.11	-0.78	-0.46	2.92E-05	Female
Deaths	Netherlands	-3.83	-3.37	-2.91	1.18E-14	Male
DALYs	New Zealand	-3.49	-3.19	-2.89	6.98E-19	Both
DALYs	New Zealand	-2.73	-2.45	-2.17	8.48E-17	Female
DALYs	New Zealand	-4.12	-3.79	-3.46	1.00E-19	Male
Deaths	New Zealand	-3.19	-2.91	-2.63	1.17E-18	Both
Deaths	New Zealand	-2.39	-2.13	-1.88	2.80E-16	Female
Deaths	New Zealand	-3.96	-3.64	-3.33	8.67E-20	Male
DALYs	Nicaragua	-0.01	0.2	0.4	0.057834696	Both
DALYs	Nicaragua	0.13	0.64	1.15	0.015852123	Female
DALYs	Nicaragua	-0.01	0.12	0.26	0.065436642	Male
Deaths	Nicaragua	0.39	0.71	1.03	8.26E-05	Both
Deaths	Nicaragua	0.34	1.09	1.84	0.005666275	Female
Deaths	Nicaragua	0.49	0.6	0.71	1.11E-11	Male
DALYs	Niger	-1.49	-1.34	-1.19	3.35E-17	Both
DALYs	Niger	-2.99	-2.7	-2.41	1.82E-17	Female
DALYs	Niger	-0.46	-0.36	-0.26	2.78E-08	Male
Deaths	Niger	-1.31	-1.2	-1.08	7.54E-19	Both
Deaths	Niger	-2.84	-2.58	-2.32	4.11E-18	Female
Deaths	Niger	-0.24	-0.17	-0.11	1.30E-05	Male
DALYs	Nigeria	-1.94	-1.72	-1.5	1.79E-15	Both
DALYs	Nigeria	-3.99	-3.52	-3.04	6.98E-15	Female
DALYs	Nigeria	-0.77	-0.63	-0.48	1.21E-09	Male
Deaths	Nigeria	-1.78	-1.57	-1.36	6.74E-15	Both
Deaths	Nigeria	-3.86	-3.42	-2.98	2.06E-15	Female
Deaths	Nigeria	-0.56	-0.44	-0.32	4.37E-08	Male
DALYs	Niue	-1.19	-1.09	-0.98	2.32E-18	Both
DALYs	Niue	-1.45	-1.36	-1.28	5.19E-24	Female
DALYs	Niue	-1.02	-0.92	-0.82	2.45E-17	Male
Deaths	Niue	-1.14	-1.04	-0.93	2.05E-18	Both
Deaths	Niue	-1.54	-1.46	-1.37	3.19E-24	Female
Deaths	Niue	-0.83	-0.75	-0.66	7.48E-17	Male
DALYs	North Macedonia	1.63	1.97	2.3	1.09E-12	Both
DALYs	North Macedonia	1.7	1.87	2.05	3.48E-19	Female
DALYs	North Macedonia	1.57	1.99	2.41	1.54E-10	Male
Deaths	North Macedonia	1.73	2.04	2.35	7.08E-14	Both
Deaths	North Macedonia	1.78	1.95	2.12	4.69E-20	Female
Deaths	North Macedonia	1.72	2.13	2.55	2.25E-11	Male
DALYs	Northern Mariana Islands	-5.54	-5.06	-4.58	9.95E-19	Both
DALYs	Northern Mariana Islands	-8.02	-7.37	-6.73	1.76E-19	Female
DALYs	Northern Mariana Islands	-3.75	-3.32	-2.89	2.75E-15	Male
Deaths	Northern Mariana Islands	-5.7	-5.21	-4.73	5.99E-19	Both
Deaths	Northern Mariana Islands	-8.1	-7.45	-6.78	2.40E-19	Female
Deaths	Northern Mariana Islands	-3.46	-3.06	-2.66	3.22E-15	Male
DALYs	Norway	-2.56	-2.27	-1.98	1.87E-15	Both
DALYs	Norway	-1.36	-1.1	-0.85	1.54E-09	Female
DALYs	Norway	-3.23	-2.9	-2.56	1.63E-16	Male
Deaths	Norway	-2.06	-1.76	-1.47	1.41E-12	Both
Deaths	Norway	-0.98	-0.72	-0.46	4.35E-06	Female
Deaths	Norway	-2.78	-2.42	-2.06	5.93E-14	Male
DALYs	Oman	-0.19	0.12	0.43	0.419188871	Both
DALYs	Oman	-0.49	-0.36	-0.23	5.40E-06	Female
DALYs	Oman	-0.14	0.15	0.44	0.304666825	Male
Deaths	Oman	0.51	0.72	0.93	1.01E-07	Both
Deaths	Oman	-0.32	-0.25	-0.17	2.98E-07	Female
Deaths	Oman	0.54	0.73	0.92	1.47E-08	Male
DALYs	Pakistan	0.51	0.72	0.93	1.38E-07	Both
DALYs	Pakistan	0.24	0.37	0.5	3.35E-06	Female
DALYs	Pakistan	0.74	0.99	1.25	7.73E-09	Male
Deaths	Pakistan	0.5	0.69	0.89	3.98E-08	Both
Deaths	Pakistan	0.3	0.44	0.57	5.22E-07	Female

Deaths	Pakistan	0.74	0.96	1.18	1.09E-09	Male
DALYs	Palau	-0.58	-0.49	-0.41	5.97E-13	Both
DALYs	Palau	-0.6	-0.49	-0.38	1.20E-09	Female
DALYs	Palau	-0.62	-0.55	-0.48	3.37E-15	Male
Deaths	Palau	-0.65	-0.57	-0.5	2.26E-15	Both
Deaths	Palau	-0.6	-0.48	-0.36	4.54E-09	Female
Deaths	Palau	-0.67	-0.59	-0.51	5.95E-15	Male
DALYs	Palestine	-0.23	-0.09	0.05	0.177587267	Both
DALYs	Palestine	-0.06	0.08	0.23	0.251995264	Female
DALYs	Palestine	-0.39	-0.23	-0.06	0.009193194	Male
Deaths	Palestine	-0.13	0	0.13	0.999601162	Both
Deaths	Palestine	-0.04	0.11	0.25	0.14195261	Female
Deaths	Palestine	-0.13	0.03	0.19	0.696545738	Male
DALYs	Panama	-1.41	-1.12	-0.82	2.49E-08	Both
DALYs	Panama	-1.71	-1.31	-0.91	2.65E-07	Female
DALYs	Panama	-1.31	-0.99	-0.67	7.19E-07	Male
Deaths	Panama	-1.6	-1.26	-0.92	2.70E-08	Both
Deaths	Panama	-1.72	-1.3	-0.89	6.43E-07	Female
Deaths	Panama	-1.53	-1.17	-0.8	5.03E-07	Male
DALYs	Papua New Guinea	-0.24	-0.2	-0.17	5.32E-12	Both
DALYs	Papua New Guinea	-0.44	-0.36	-0.29	1.73E-10	Female
DALYs	Papua New Guinea	-0.18	-0.15	-0.12	7.36E-11	Male
Deaths	Papua New Guinea	-0.36	-0.32	-0.27	4.93E-15	Both
Deaths	Papua New Guinea	-0.6	-0.52	-0.44	7.47E-14	Female
Deaths	Papua New Guinea	-0.22	-0.19	-0.15	7.27E-12	Male
DALYs	Paraguay	0.8	1.08	1.35	8.18E-09	Both
DALYs	Paraguay	0.34	0.52	0.7	1.62E-06	Female
DALYs	Paraguay	0.98	1.3	1.62	4.84E-09	Male
Deaths	Paraguay	0.88	1.15	1.42	1.54E-09	Both
Deaths	Paraguay	0.56	0.73	0.91	2.52E-09	Female
Deaths	Paraguay	1.03	1.36	1.68	2.36E-09	Male
DALYs	Peru	-0.95	-0.82	-0.69	2.65E-13	Both
DALYs	Peru	-0.88	-0.76	-0.63	9.99E-13	Female
DALYs	Peru	-1.01	-0.84	-0.67	1.15E-10	Male
Deaths	Peru	-0.83	-0.7	-0.58	5.18E-12	Both
Deaths	Peru	-1.01	-0.88	-0.74	1.69E-13	Female
Deaths	Peru	-0.72	-0.56	-0.41	3.43E-08	Male
DALYs	Philippines	2.41	2.92	3.44	2.55E-12	Both
DALYs	Philippines	2.48	2.98	3.48	7.66E-13	Female
DALYs	Philippines	2.47	3	3.53	2.16E-12	Male
Deaths	Philippines	1.79	2.18	2.57	4.05E-12	Both
Deaths	Philippines	1.75	2.1	2.45	7.03E-13	Female
Deaths	Philippines	1.97	2.39	2.81	1.92E-12	Male
DALYs	Poland	0.27	0.81	1.35	0.004849833	Both
DALYs	Poland	0.38	0.77	1.16	0.00033494	Female
DALYs	Poland	0.11	0.72	1.34	0.023070875	Male
Deaths	Poland	0.56	1.13	1.71	0.000374227	Both
Deaths	Poland	0.77	1.19	1.61	3.13E-06	Female
Deaths	Poland	0.37	1.03	1.7	0.00342977	Male
DALYs	Portugal	-0.1	0.05	0.21	0.501373804	Both
DALYs	Portugal	0.05	0.27	0.48	0.017060964	Female
DALYs	Portugal	-0.21	-0.05	0.1	0.502085945	Male
Deaths	Portugal	0.24	0.36	0.48	1.42E-06	Both
Deaths	Portugal	0.37	0.55	0.73	7.90E-07	Female
Deaths	Portugal	0.09	0.22	0.34	0.001212168	Male
DALYs	Puerto Rico	-2.87	-2.56	-2.24	6.92E-16	Both
DALYs	Puerto Rico	-2.31	-2.06	-1.81	4.91E-16	Female
DALYs	Puerto Rico	-3.06	-2.71	-2.36	2.98E-15	Male
Deaths	Puerto Rico	-2.91	-2.6	-2.28	4.94E-16	Both
Deaths	Puerto Rico	-2.32	-2.07	-1.81	6.31E-16	Female
Deaths	Puerto Rico	-3.12	-2.76	-2.4	2.43E-15	Male
DALYs	Qatar	-0.54	-0.32	-0.1	0.006500852	Both
DALYs	Qatar	0.74	0.9	1.05	2.15E-12	Female
DALYs	Qatar	-1.32	-1.04	-0.76	3.49E-08	Male
Deaths	Qatar	0.36	0.68	1	0.000170037	Both
Deaths	Qatar	1.25	1.46	1.67	2.50E-14	Female
Deaths	Qatar	-0.66	-0.24	0.18	0.243234376	Male
DALYs	Republic of Korea	-1.17	-1.05	-0.93	3.24E-17	Both

DALYs	Republic of Korea	-1.36	-1.18	-1.01	4.02E-14	Female
DALYs	Republic of Korea	-1.24	-1.14	-1.03	3.05E-19	Male
Deaths	Republic of Korea	-0.66	-0.52	-0.39	1.06E-08	Both
Deaths	Republic of Korea	-0.69	-0.54	-0.38	1.55E-07	Female
Deaths	Republic of Korea	-0.88	-0.74	-0.6	1.53E-11	Male
DALYs	Republic of Moldova	1.48	1.84	2.2	3.19E-11	Both
DALYs	Republic of Moldova	1.41	1.87	2.35	5.71E-09	Female
DALYs	Republic of Moldova	1.39	1.72	2.05	2.02E-11	Male
Deaths	Republic of Moldova	1.36	1.65	1.95	4.03E-12	Both
Deaths	Republic of Moldova	1.36	1.76	2.16	6.24E-10	Female
Deaths	Republic of Moldova	1.16	1.42	1.69	1.13E-11	Male
DALYs	Romania	0.93	1.06	1.18	1.05E-16	Both
DALYs	Romania	1.03	1.09	1.15	1.13E-25	Female
DALYs	Romania	0.87	1.05	1.22	1.09E-12	Male
Deaths	Romania	1.19	1.29	1.39	2.38E-21	Both
Deaths	Romania	1.26	1.34	1.41	5.89E-25	Female
Deaths	Romania	1.12	1.27	1.42	1.04E-16	Male
DALYs	Russian Federation	0.64	0.91	1.19	1.79E-07	Both
DALYs	Russian Federation	0.22	0.48	0.75	0.000764946	Female
DALYs	Russian Federation	0.62	0.93	1.24	1.24E-06	Male
Deaths	Russian Federation	0.8	1.09	1.38	1.83E-08	Both
Deaths	Russian Federation	0.43	0.78	1.14	8.55E-05	Female
Deaths	Russian Federation	0.61	0.92	1.23	1.43E-06	Male
DALYs	Rwanda	-3.39	-2.95	-2.52	6.00E-14	Both
DALYs	Rwanda	-2.46	-2.09	-1.71	6.51E-12	Female
DALYs	Rwanda	-3.91	-3.44	-2.98	7.60E-15	Male
Deaths	Rwanda	-2.95	-2.56	-2.17	1.55E-13	Both
Deaths	Rwanda	-2.31	-1.94	-1.57	2.36E-11	Female
Deaths	Rwanda	-3.33	-2.93	-2.53	8.80E-15	Male
DALYs	Saint Kitts and Nevis	-1.24	-0.91	-0.59	3.28E-06	Both
DALYs	Saint Kitts and Nevis	-0.56	-0.23	0.1	0.165559436	Female
DALYs	Saint Kitts and Nevis	-1.74	-1.4	-1.05	6.63E-09	Male
Deaths	Saint Kitts and Nevis	-0.8	-0.53	-0.26	0.00044515	Both
Deaths	Saint Kitts and Nevis	-0.15	0.2	0.54	0.25736884	Female
Deaths	Saint Kitts and Nevis	-1.28	-1.03	-0.78	5.10E-09	Male
DALYs	Saint Lucia	-1.4	-1.08	-0.76	1.81E-07	Both
DALYs	Saint Lucia	-1.62	-1.26	-0.89	1.09E-07	Female
DALYs	Saint Lucia	-1.32	-1.02	-0.73	1.21E-07	Male
Deaths	Saint Lucia	-1.51	-1.16	-0.8	2.88E-07	Both
Deaths	Saint Lucia	-1.62	-1.24	-0.86	3.25E-07	Female
Deaths	Saint Lucia	-1.52	-1.18	-0.83	1.31E-07	Male
DALYs	Saint Vincent and the Grenadin	-0.14	0.01	0.16	0.879529071	Both
DALYs	Saint Vincent and the Grenadin	0.08	0.17	0.25	0.000287614	Female
DALYs	Saint Vincent and the Grenadin	-0.56	-0.35	-0.14	0.001965504	Male
Deaths	Saint Vincent and the Grenadin	0	0.13	0.25	0.047054732	Both
Deaths	Saint Vincent and the Grenadin	0.27	0.36	0.45	9.85E-09	Female
Deaths	Saint Vincent and the Grenadin	-0.49	-0.3	-0.12	0.00223948	Male
DALYs	Samoa	-1.23	-1.12	-1	5.93E-18	Both
DALYs	Samoa	-0.94	-0.8	-0.67	5.93E-13	Female
DALYs	Samoa	-1.38	-1.27	-1.17	5.33E-21	Male
Deaths	Samoa	-1.36	-1.21	-1.06	3.07E-16	Both
Deaths	Samoa	-1.17	-1	-0.83	1.31E-12	Female
Deaths	Samoa	-1.42	-1.3	-1.19	1.05E-19	Male
DALYs	San Marino	-0.54	-0.37	-0.2	0.000144983	Both
DALYs	San Marino	0.4	0.57	0.74	1.72E-07	Female
DALYs	San Marino	-0.81	-0.63	-0.45	7.81E-08	Male
Deaths	San Marino	-0.36	-0.22	-0.08	0.00278755	Both
Deaths	San Marino	0.45	0.63	0.81	6.18E-08	Female
Deaths	San Marino	-0.69	-0.55	-0.41	6.43E-09	Male
DALYs	Sao Tome and Principe	-0.22	-0.04	0.14	0.651110597	Both
DALYs	Sao Tome and Principe	-2.48	-2.16	-1.83	9.40E-14	Female
DALYs	Sao Tome and Principe	1.23	1.32	1.41	1.69E-23	Male
Deaths	Sao Tome and Principe	-0.19	0	0.2	0.963442199	Both
Deaths	Sao Tome and Principe	-2.4	-2.08	-1.76	1.78E-13	Female
Deaths	Sao Tome and Principe	1.24	1.37	1.49	2.28E-19	Male
DALYs	Saudi Arabia	-0.04	0.11	0.26	0.130615601	Both
DALYs	Saudi Arabia	-1.62	-1.35	-1.07	8.20E-11	Female
DALYs	Saudi Arabia	0.24	0.45	0.66	0.000163939	Male

Deaths	Saudi Arabia	0.03	0.19	0.36	0.022311713	Both
Deaths	Saudi Arabia	-1.49	-1.2	-0.91	3.89E-09	Female
Deaths	Saudi Arabia	0.32	0.54	0.77	2.77E-05	Male
DALYs	Senegal	-0.94	-0.79	-0.63	4.34E-11	Both
DALYs	Senegal	-2.7	-2.41	-2.12	4.75E-16	Female
DALYs	Senegal	0.08	0.21	0.33	0.002911462	Male
Deaths	Senegal	-0.91	-0.8	-0.69	1.84E-14	Both
Deaths	Senegal	-2.67	-2.4	-2.13	7.25E-17	Female
Deaths	Senegal	0.17	0.25	0.34	2.00E-06	Male
DALYs	Serbia	0.88	1.02	1.17	9.99E-15	Both
DALYs	Serbia	1.58	1.79	2	1.04E-16	Female
DALYs	Serbia	0.59	0.77	0.95	1.30E-09	Male
Deaths	Serbia	1.42	1.58	1.73	1.10E-18	Both
Deaths	Serbia	2.11	2.36	2.61	7.01E-18	Female
Deaths	Serbia	1.11	1.29	1.47	6.86E-15	Male
DALYs	Seychelles	-0.33	-0.23	-0.12	0.00018082	Both
DALYs	Seychelles	0.04	0.1	0.16	0.002715371	Female
DALYs	Seychelles	-0.73	-0.56	-0.39	1.98E-07	Male
Deaths	Seychelles	-0.27	-0.17	-0.06	0.003711064	Both
Deaths	Seychelles	0.09	0.15	0.2	6.63E-06	Female
Deaths	Seychelles	-0.67	-0.49	-0.32	3.71E-06	Male
DALYs	Sierra Leone	-0.91	-0.75	-0.59	2.01E-10	Both
DALYs	Sierra Leone	-2.63	-2.31	-1.98	1.97E-14	Female
DALYs	Sierra Leone	0.02	0.11	0.21	0.019515474	Male
Deaths	Sierra Leone	-0.92	-0.81	-0.68	6.27E-14	Both
Deaths	Sierra Leone	-2.67	-2.38	-2.09	4.93E-16	Female
Deaths	Sierra Leone	0.09	0.16	0.23	7.35E-05	Male
DALYs	Singapore	-2.6	-2.46	-2.32	3.93E-25	Both
DALYs	Singapore	-1.84	-1.59	-1.34	2.27E-13	Female
DALYs	Singapore	-3.08	-2.93	-2.79	1.37E-26	Male
Deaths	Singapore	-2.11	-1.98	-1.86	6.38E-24	Both
Deaths	Singapore	-1.38	-1.14	-0.9	1.66E-10	Female
Deaths	Singapore	-2.66	-2.51	-2.37	1.24E-24	Male
DALYs	Slovakia	0.32	0.75	1.18	0.001222896	Both
DALYs	Slovakia	-0.42	0.02	0.47	0.913031316	Female
DALYs	Slovakia	0.6	1.05	1.51	5.66E-05	Male
Deaths	Slovakia	0.45	0.87	1.29	0.000202884	Both
Deaths	Slovakia	-0.25	0.21	0.68	0.362894152	Female
Deaths	Slovakia	0.85	1.27	1.7	1.31E-06	Male
DALYs	Slovenia	-0.66	-0.17	0.32	0.486239143	Both
DALYs	Slovenia	-0.23	0.04	0.3	0.773473889	Female
DALYs	Slovenia	-1.14	-0.51	0.12	0.109310108	Male
Deaths	Slovenia	-0.21	0.26	0.72	0.268824278	Both
Deaths	Slovenia	0.23	0.48	0.72	0.000407004	Female
Deaths	Slovenia	-0.71	-0.07	0.57	0.820715583	Male
DALYs	Solomon Islands	-0.55	-0.48	-0.4	3.63E-13	Both
DALYs	Solomon Islands	-0.85	-0.73	-0.61	3.94E-13	Female
DALYs	Solomon Islands	-0.31	-0.25	-0.19	2.32E-09	Male
Deaths	Solomon Islands	-0.51	-0.43	-0.35	7.25E-12	Both
Deaths	Solomon Islands	-0.72	-0.61	-0.5	1.06E-11	Female
Deaths	Solomon Islands	-0.29	-0.22	-0.16	1.10E-07	Male
DALYs	Somalia	-2.15	-1.97	-1.78	9.10E-19	Both
DALYs	Somalia	-1.99	-1.8	-1.6	1.93E-17	Female
DALYs	Somalia	-2.09	-1.93	-1.76	9.21E-20	Male
Deaths	Somalia	-2.05	-1.87	-1.69	8.39E-19	Both
Deaths	Somalia	-1.89	-1.7	-1.51	4.45E-17	Female
Deaths	Somalia	-1.98	-1.82	-1.67	2.27E-20	Male
DALYs	South Africa	-3.02	-2.47	-1.92	7.80E-10	Both
DALYs	South Africa	-3.44	-2.98	-2.52	1.88E-13	Female
DALYs	South Africa	-2.75	-2.11	-1.46	3.49E-07	Male
Deaths	South Africa	-2.94	-2.4	-1.85	1.09E-09	Both
Deaths	South Africa	-3.35	-2.89	-2.42	6.59E-13	Female
Deaths	South Africa	-2.56	-1.93	-1.29	1.14E-06	Male
DALYs	South Sudan	-2.41	-2.18	-1.95	1.15E-17	Both
DALYs	South Sudan	-2.27	-2.03	-1.78	4.94E-16	Female
DALYs	South Sudan	-2.37	-2.13	-1.9	3.17E-17	Male
Deaths	South Sudan	-2.2	-2	-1.8	3.08E-18	Both
Deaths	South Sudan	-2.15	-1.92	-1.7	1.87E-16	Female

Deaths	South Sudan	-2.16	-1.96	-1.76	4.55E-18	Male
DALYs	Spain	-1.45	-0.95	-0.44	0.000649441	Both
DALYs	Spain	0.01	0.34	0.66	0.041183228	Female
DALYs	Spain	-1.86	-1.32	-0.77	3.12E-05	Male
Deaths	Spain	-1.1	-0.59	-0.08	0.024120387	Both
Deaths	Spain	0.17	0.52	0.86	0.00442882	Female
Deaths	Spain	-1.5	-0.95	-0.4	0.001410258	Male
DALYs	Sri Lanka	-0.21	-0.11	-0.01	0.039056498	Both
DALYs	Sri Lanka	0	0.14	0.28	0.048179074	Female
DALYs	Sri Lanka	-0.08	0.08	0.23	0.315164912	Male
Deaths	Sri Lanka	0.07	0.2	0.33	0.004383112	Both
Deaths	Sri Lanka	0.18	0.36	0.53	0.000228979	Female
Deaths	Sri Lanka	0.33	0.49	0.65	6.88E-07	Male
DALYs	Sudan	-0.06	0.02	0.09	0.676726633	Both
DALYs	Sudan	0.12	0.23	0.33	0.000137721	Female
DALYs	Sudan	-0.22	-0.14	-0.06	0.000985107	Male
Deaths	Sudan	0.15	0.24	0.33	4.53E-06	Both
Deaths	Sudan	0.27	0.37	0.48	5.14E-08	Female
Deaths	Sudan	-0.03	0.06	0.15	0.161465251	Male
DALYs	Suriname	-0.82	-0.56	-0.3	0.00013599	Both
DALYs	Suriname	-0.78	-0.68	-0.57	1.80E-13	Female
DALYs	Suriname	-0.71	-0.4	-0.09	0.013614447	Male
Deaths	Suriname	-0.78	-0.51	-0.24	0.000560216	Both
Deaths	Suriname	-0.67	-0.55	-0.43	4.28E-10	Female
Deaths	Suriname	-0.74	-0.41	-0.08	0.01747267	Male
DALYs	Sweden	-2.67	-2.34	-2	3.17E-14	Both
DALYs	Sweden	-1.37	-1.11	-0.85	1.99E-09	Female
DALYs	Sweden	-3.38	-2.99	-2.61	2.09E-15	Male
Deaths	Sweden	-2.23	-1.9	-1.57	2.02E-12	Both
Deaths	Sweden	-1.02	-0.78	-0.54	3.73E-07	Female
Deaths	Sweden	-2.99	-2.6	-2.21	7.44E-14	Male
DALYs	Switzerland	-2.41	-2.27	-2.12	3.50E-23	Both
DALYs	Switzerland	-0.95	-0.79	-0.62	1.73E-10	Female
DALYs	Switzerland	-3.13	-2.97	-2.81	1.06E-25	Male
Deaths	Switzerland	-1.88	-1.75	-1.62	1.17E-21	Both
Deaths	Switzerland	-0.56	-0.4	-0.25	1.11E-05	Female
Deaths	Switzerland	-2.68	-2.54	-2.4	5.45E-25	Male
DALYs	Syrian Arab Republic	-0.43	-0.28	-0.14	0.000433628	Both
DALYs	Syrian Arab Republic	-0.37	-0.29	-0.2	3.35E-07	Female
DALYs	Syrian Arab Republic	-0.43	-0.24	-0.05	0.016983265	Male
Deaths	Syrian Arab Republic	-0.07	0.06	0.18	0.376497649	Both
Deaths	Syrian Arab Republic	-0.31	-0.21	-0.11	0.000251867	Female
Deaths	Syrian Arab Republic	-0.05	0.11	0.28	0.167331738	Male
DALYs	Taiwan (Province of China)	2.16	2.58	3.01	5.24E-13	Both
DALYs	Taiwan (Province of China)	1.44	1.85	2.26	4.67E-10	Female
DALYs	Taiwan (Province of China)	2.63	3.07	3.52	1.79E-14	Male
Deaths	Taiwan (Province of China)	2.03	2.5	2.97	9.15E-12	Both
Deaths	Taiwan (Province of China)	1.58	2.02	2.45	2.85E-10	Female
Deaths	Taiwan (Province of China)	2.45	2.92	3.39	2.53E-13	Male
DALYs	Tajikistan	0.92	1.07	1.21	5.08E-15	Both
DALYs	Tajikistan	0.5	0.62	0.74	4.81E-11	Female
DALYs	Tajikistan	1.4	1.67	1.94	2.78E-13	Male
Deaths	Tajikistan	0.98	1.17	1.36	3.52E-13	Both
Deaths	Tajikistan	0.2	0.47	0.75	0.001527991	Female
Deaths	Tajikistan	2.04	2.24	2.44	8.12E-20	Male
DALYs	Thailand	-1.02	-0.82	-0.62	3.71E-09	Both
DALYs	Thailand	-1.25	-1.1	-0.94	1.57E-14	Female
DALYs	Thailand	-0.92	-0.69	-0.45	2.35E-06	Male
Deaths	Thailand	-1.01	-0.85	-0.69	2.70E-11	Both
Deaths	Thailand	-1.22	-1.08	-0.94	1.84E-15	Female
Deaths	Thailand	-0.91	-0.71	-0.5	1.02E-07	Male
DALYs	Timor-Leste	1.87	2.03	2.19	3.19E-21	Both
DALYs	Timor-Leste	1.58	1.66	1.74	4.24E-27	Female
DALYs	Timor-Leste	2.06	2.29	2.53	3.35E-18	Male
Deaths	Timor-Leste	1.88	2.01	2.15	7.54E-23	Both
Deaths	Timor-Leste	1.51	1.58	1.64	7.18E-29	Female
Deaths	Timor-Leste	2.09	2.31	2.52	3.92E-19	Male
DALYs	Togo	-1.07	-0.94	-0.8	4.59E-14	Both

DALYs	Togo	-3.22	-2.84	-2.47	4.41E-15	Female
DALYs	Togo	0.34	0.47	0.6	5.80E-08	Male
Deaths	Togo	-1.16	-1.03	-0.89	1.21E-15	Both
Deaths	Togo	-3.13	-2.78	-2.42	1.53E-15	Female
Deaths	Togo	0.39	0.51	0.64	4.87E-09	Male
DALYs	Tokelau	-0.72	-0.68	-0.64	9.78E-25	Both
DALYs	Tokelau	-0.84	-0.78	-0.72	4.09E-21	Female
DALYs	Tokelau	-0.71	-0.67	-0.63	2.08E-24	Male
Deaths	Tokelau	-0.69	-0.66	-0.62	8.25E-25	Both
Deaths	Tokelau	-0.82	-0.75	-0.68	1.45E-19	Female
Deaths	Tokelau	-0.69	-0.65	-0.61	4.08E-24	Male
DALYs	Tonga	0.04	0.24	0.44	0.023029338	Both
DALYs	Tonga	-0.24	-0.15	-0.06	0.001979971	Female
DALYs	Tonga	0.18	0.4	0.63	0.001076859	Male
Deaths	Tonga	-0.11	0.11	0.33	0.314996205	Both
Deaths	Tonga	-0.23	-0.15	-0.07	0.000612579	Female
Deaths	Tonga	0.08	0.33	0.58	0.012419358	Male
DALYs	Trinidad and Tobago	-1.09	-0.74	-0.38	0.000250977	Both
DALYs	Trinidad and Tobago	-1.01	-0.63	-0.25	0.002169004	Female
DALYs	Trinidad and Tobago	-1.17	-0.82	-0.47	4.71E-05	Male
Deaths	Trinidad and Tobago	-1.04	-0.69	-0.35	0.000284408	Both
Deaths	Trinidad and Tobago	-0.85	-0.48	-0.12	0.011760832	Female
Deaths	Trinidad and Tobago	-1.19	-0.86	-0.53	1.15E-05	Male
DALYs	Tunisia	0.01	0.1	0.2	0.03556944	Both
DALYs	Tunisia	-0.16	-0.1	-0.04	0.001854565	Female
DALYs	Tunisia	0.2	0.32	0.44	8.53E-06	Male
Deaths	Tunisia	0.06	0.16	0.26	0.00383383	Both
Deaths	Tunisia	-0.16	-0.08	-0.01	0.021433334	Female
Deaths	Tunisia	0.3	0.42	0.54	4.34E-08	Male
DALYs	Turkey	-2.25	-1.96	-1.66	6.62E-14	Both
DALYs	Turkey	-1.3	-1.13	-0.97	2.79E-14	Female
DALYs	Turkey	-2.47	-2.14	-1.82	1.33E-13	Male
Deaths	Turkey	-1.77	-1.54	-1.32	3.03E-14	Both
Deaths	Turkey	-1.14	-0.97	-0.79	8.46E-12	Female
Deaths	Turkey	-1.95	-1.68	-1.41	4.33E-13	Male
DALYs	Turkmenistan	2.84	3.17	3.51	5.67E-18	Both
DALYs	Turkmenistan	2.59	2.83	3.08	2.73E-20	Female
DALYs	Turkmenistan	2.73	3.1	3.48	1.55E-16	Male
Deaths	Turkmenistan	2.74	3.01	3.27	5.29E-20	Both
Deaths	Turkmenistan	2.6	2.79	2.98	3.72E-23	Female
Deaths	Turkmenistan	2.54	2.86	3.17	2.05E-17	Male
DALYs	Tuvalu	-1.03	-0.91	-0.8	4.48E-16	Both
DALYs	Tuvalu	-1.37	-1.22	-1.06	1.53E-15	Female
DALYs	Tuvalu	-1	-0.9	-0.8	3.49E-17	Male
Deaths	Tuvalu	-1.2	-1.06	-0.92	1.65E-15	Both
Deaths	Tuvalu	-1.44	-1.26	-1.08	2.83E-14	Female
Deaths	Tuvalu	-1.08	-0.96	-0.85	1.36E-16	Male
DALYs	Uganda	-1.37	-1.19	-1.01	9.84E-14	Both
DALYs	Uganda	-1.1	-0.92	-0.74	4.93E-11	Female
DALYs	Uganda	-1.39	-1.17	-0.94	2.82E-11	Male
Deaths	Uganda	-1.25	-1.1	-0.94	8.40E-15	Both
Deaths	Uganda	-1.07	-0.89	-0.72	3.43E-11	Female
Deaths	Uganda	-1.22	-1.04	-0.85	5.50E-12	Male
DALYs	Ukraine	1.23	1.52	1.81	1.31E-11	Both
DALYs	Ukraine	1.24	1.38	1.53	5.93E-18	Female
DALYs	Ukraine	0.99	1.32	1.66	6.51E-09	Male
Deaths	Ukraine	0.97	1.18	1.39	4.94E-12	Both
Deaths	Ukraine	0.96	1.06	1.16	2.96E-19	Female
Deaths	Ukraine	0.65	0.91	1.17	6.46E-08	Male
DALYs	United Arab Emirates	-0.55	-0.24	0.08	0.130981684	Both
DALYs	United Arab Emirates	-1.38	-0.85	-0.31	0.003140239	Female
DALYs	United Arab Emirates	-0.99	-0.65	-0.31	0.000579712	Male
Deaths	United Arab Emirates	-0.62	-0.23	0.16	0.22928347	Both
Deaths	United Arab Emirates	-1.81	-1.04	-0.26	0.010545432	Female
Deaths	United Arab Emirates	-1.16	-0.75	-0.34	0.000891215	Male
DALYs	United Kingdom	-3.22	-2.91	-2.59	2.57E-17	Both
DALYs	United Kingdom	-2.43	-2.13	-1.82	2.73E-14	Female
DALYs	United Kingdom	-3.82	-3.49	-3.16	6.96E-19	Male

Deaths	United Kingdom	-2.77	-2.46	-2.15	1.13E-15	Both
Deaths	United Kingdom	-2.05	-1.74	-1.44	2.63E-12	Female
Deaths	United Kingdom	-3.47	-3.14	-2.82	7.73E-18	Male
DALYs	United Republic of Tanzania	-1.62	-1.42	-1.23	7.82E-15	Both
DALYs	United Republic of Tanzania	-1.31	-1.11	-0.9	1.15E-11	Female
DALYs	United Republic of Tanzania	-1.79	-1.58	-1.38	2.71E-15	Male
Deaths	United Republic of Tanzania	-1.49	-1.32	-1.15	2.03E-15	Both
Deaths	United Republic of Tanzania	-1.35	-1.15	-0.96	1.07E-12	Female
Deaths	United Republic of Tanzania	-1.56	-1.39	-1.22	6.10E-16	Male
DALYs	United States of America	-3.33	-3.08	-2.83	1.27E-20	Both
DALYs	United States of America	-2.62	-2.36	-2.11	2.96E-17	Female
DALYs	United States of America	-3.91	-3.64	-3.36	2.28E-21	Male
Deaths	United States of America	-3.47	-3.21	-2.95	1.52E-20	Both
Deaths	United States of America	-2.52	-2.26	-2.01	7.40E-17	Female
Deaths	United States of America	-4.32	-4.01	-3.71	2.52E-21	Male
DALYs	United States Virgin Islands	0.1	0.36	0.61	0.008120593	Both
DALYs	United States Virgin Islands	-1.01	-0.94	-0.86	5.19E-21	Female
DALYs	United States Virgin Islands	0.63	0.97	1.32	3.30E-06	Male
Deaths	United States Virgin Islands	0.21	0.45	0.69	0.000746862	Both
Deaths	United States Virgin Islands	-0.68	-0.59	-0.5	8.12E-14	Female
Deaths	United States Virgin Islands	0.69	1.02	1.36	7.48E-07	Male
DALYs	Uruguay	-0.94	-0.71	-0.47	1.49E-06	Both
DALYs	Uruguay	-0.57	-0.44	-0.31	1.36E-07	Female
DALYs	Uruguay	-1.11	-0.78	-0.45	4.46E-05	Male
Deaths	Uruguay	-0.86	-0.6	-0.35	4.01E-05	Both
Deaths	Uruguay	-0.49	-0.36	-0.22	9.33E-06	Female
Deaths	Uruguay	-0.99	-0.64	-0.29	0.000896268	Male
DALYs	Uzbekistan	4.06	4.22	4.39	6.66E-30	Both
DALYs	Uzbekistan	4.06	4.36	4.67	7.62E-23	Female
DALYs	Uzbekistan	3.72	4.01	4.3	2.35E-22	Male
Deaths	Uzbekistan	4.23	4.4	4.57	8.68E-30	Both
Deaths	Uzbekistan	4.3	4.63	4.96	9.94E-23	Female
Deaths	Uzbekistan	3.65	4.03	4.41	2.79E-19	Male
DALYs	Vanuatu	-0.5	-0.35	-0.2	4.93E-05	Both
DALYs	Vanuatu	-0.89	-0.7	-0.5	4.65E-08	Female
DALYs	Vanuatu	-0.24	-0.12	0.01	0.071803292	Male
Deaths	Vanuatu	-0.59	-0.46	-0.33	5.31E-08	Both
Deaths	Vanuatu	-1	-0.85	-0.69	8.17E-12	Female
Deaths	Vanuatu	-0.3	-0.18	-0.07	0.002700039	Male
DALYs	Venezuela (Bolivarian Republic	-0.74	-0.43	-0.12	0.008909582	Both
DALYs	Venezuela (Bolivarian Republic	-0.26	0.02	0.31	0.874080066	Female
DALYs	Venezuela (Bolivarian Republic	-0.93	-0.58	-0.23	0.00205887	Male
Deaths	Venezuela (Bolivarian Republic	-0.57	-0.24	0.08	0.133557032	Both
Deaths	Venezuela (Bolivarian Republic	-0.08	0.22	0.52	0.143854934	Female
Deaths	Venezuela (Bolivarian Republic	-0.76	-0.39	-0.02	0.038035831	Male
DALYs	Viet Nam	0.49	0.54	0.6	8.93E-18	Both
DALYs	Viet Nam	-0.22	-0.08	0.06	0.260329441	Female
DALYs	Viet Nam	0.86	0.89	0.93	6.75E-29	Male
Deaths	Viet Nam	0.43	0.52	0.6	5.66E-13	Both
Deaths	Viet Nam	-0.32	-0.15	0.02	0.08750918	Female
Deaths	Viet Nam	0.98	1.03	1.07	1.82E-28	Male
DALYs	Yemen	0.26	0.32	0.39	5.36E-11	Both
DALYs	Yemen	0.29	0.36	0.44	3.30E-11	Female
DALYs	Yemen	0.13	0.19	0.26	1.81E-06	Male
Deaths	Yemen	0.51	0.57	0.64	9.59E-17	Both
Deaths	Yemen	0.37	0.44	0.51	4.04E-13	Female
Deaths	Yemen	0.26	0.32	0.38	8.64E-12	Male
DALYs	Zambia	0.01	0.22	0.43	0.037152685	Both
DALYs	Zambia	-0.65	-0.36	-0.06	0.020845523	Female
DALYs	Zambia	0.52	0.71	0.9	1.89E-08	Male
Deaths	Zambia	-0.06	0.16	0.39	0.152854608	Both
Deaths	Zambia	-0.67	-0.35	-0.03	0.031617079	Female
Deaths	Zambia	0.52	0.71	0.89	1.26E-08	Male
DALYs	Zimbabwe	0.18	0.41	0.64	0.001000468	Both
DALYs	Zimbabwe	0.37	0.65	0.94	6.54E-05	Female
DALYs	Zimbabwe	0.22	0.63	1.05	0.004203675	Male
Deaths	Zimbabwe	0.03	0.24	0.44	0.023063844	Both
Deaths	Zimbabwe	0.26	0.48	0.69	8.66E-05	Female

Deaths	Zimbabwe	0.05	0.4	0.76	0.026691483	Male
