# Supplementary material for: Burden of Aortic Aneurysm and Its Attributable Risk Factors from 1990 to 2019: An Analysis of the Global Burden of Disease Study 2019
Source: Front Cardiovasc Med. 2022 May 31;9:901225. doi: 10.3389/fcvm.2022.901225 (PMC9197430; doi:10.3389/fcvm.2022.901225)
Supplement: Supplementary Table 8 — Estimated annual percentage changes of aortic aneurism-related attributable risks in 31 GBD regions from 1990 to 2019. DALY, disability-adjusted life year rate. SDI, socio-demographic index; EAPC, estimated annual percentage changes; GBD, Global Burden of Disease. [file Data_Sheet_8.PDF]

measure	location_id	rei	EAPC0.025	EAPC0.5	EAPC0.975	p	Gender
DALYs	Oceania	Diet high in sodium	-0.89	-0.72	-0.55	1.98E-09	Female
DALYs	Southeast Asia	Diet high in sodium	-1.01	-0.89	-0.78	1.96E-15	Female
DALYs	Global	Diet high in sodium	-0.98	-0.89	-0.81	6.90E-19	Female
DALYs	Central Asia	Diet high in sodium	-0.53	-0.36	-0.19	0.000167487	Female
DALYs	East Asia	Diet high in sodium	-1.14	-1.06	-0.99	2.72E-22	Female
DALYs	Australasia	Diet high in sodium	-3.49	-3.24	-2.98	7.88E-21	Female
DALYs	Southern Latin America	Diet high in sodium	-0.9	-0.7	-0.5	8.58E-08	Female
DALYs	High-middle SDI	Diet high in sodium	-0.68	-0.6	-0.52	2.17E-15	Female
DALYs	Low SDI	Diet high in sodium	-1.64	-1.41	-1.17	1.05E-12	Female
DALYs	Eastern Europe	Diet high in sodium	0.33	0.55	0.77	2.47E-05	Female
DALYs	Central Europe	Diet high in sodium	-0.59	-0.52	-0.46	1.04E-15	Female
DALYs	High-income Asia Pacific	Diet high in sodium	-1.18	-1.01	-0.85	4.69E-13	Female
DALYs	Middle SDI	Diet high in sodium	-0.93	-0.89	-0.85	1.61E-27	Female
DALYs	North Africa and Middle East	Diet high in sodium	-0.68	-0.62	-0.56	2.88E-19	Female
DALYs	Low-middle SDI	Diet high in sodium	-0.05	0.01	0.07	0.843113558	Female
DALYs	Central Latin America	Diet high in sodium	-1.13	-0.85	-0.56	1.29E-06	Female
DALYs	Andean Latin America	Diet high in sodium	-0.55	-0.47	-0.38	6.63E-12	Female
DALYs	Eastern Sub-Saharan Africa	Diet high in sodium	-2.08	-1.83	-1.58	8.93E-15	Female
DALYs	High SDI	Diet high in sodium	-1.52	-1.34	-1.15	1.44E-14	Female
DALYs	World Bank Low Income	Diet high in sodium	-2.03	-1.8	-1.57	1.08E-15	Female
DALYs	South Asia	Diet high in sodium	0.33	0.42	0.51	1.27E-10	Female
DALYs	Western Europe	Diet high in sodium	-1.57	-1.39	-1.22	1.22E-15	Female
DALYs	World Bank Lower Middle Income	Diet high in sodium	-0.21	-0.17	-0.12	2.62E-08	Female
DALYs	World Bank High Income	Diet high in sodium	-1.33	-1.16	-0.99	7.46E-14	Female
DALYs	High-income North America	Diet high in sodium	-2.83	-2.54	-2.26	6.26E-17	Female
DALYs	Western Sub-Saharan Africa	Diet high in sodium	-3.18	-2.77	-2.35	8.48E-14	Female
DALYs	Tropical Latin America	Diet high in sodium	0.18	0.43	0.69	0.001864929	Female
DALYs	Central Sub-Saharan Africa	Diet high in sodium	-1.07	-0.91	-0.76	2.16E-12	Female
DALYs	Caribbean	Diet high in sodium	-0.93	-0.79	-0.66	8.31E-13	Female
DALYs	World Bank Upper Middle Income	Diet high in sodium	-0.89	-0.83	-0.77	6.03E-22	Female
DALYs	Southern Sub-Saharan Africa	Diet high in sodium	-3.1	-2.82	-2.53	4.42E-18	Female
DALYs	Oceania	Lead exposure	-0.89	-0.78	-0.66	5.30E-14	Female
DALYs	Southeast Asia	Lead exposure	-0.47	-0.24	-0.01	0.042195619	Female
DALYs	Eastern Europe	Lead exposure	0.55	0.72	0.89	1.44E-09	Female
DALYs	Global	Lead exposure	-0.9	-0.77	-0.64	1.12E-12	Female
DALYs	Central Asia	Lead exposure	1.63	1.73	1.83	1.20E-24	Female
DALYs	North Africa and Middle East	Lead exposure	-1.36	-1.24	-1.13	2.99E-19	Female
DALYs	Australasia	Lead exposure	-4.38	-4.07	-3.77	2.08E-21	Female
DALYs	High-income North America	Lead exposure	-4.81	-4.51	-4.21	7.62E-23	Female
DALYs	High-income Asia Pacific	Lead exposure	-0.51	-0.29	-0.07	0.010656119	Female
DALYs	Southern Latin America	Lead exposure	-1.2	-0.92	-0.64	2.65E-07	Female
DALYs	High-middle SDI	Lead exposure	-1	-0.74	-0.48	3.00E-06	Female
DALYs	Low SDI	Lead exposure	-0.27	-0.17	-0.07	0.00121796	Female
DALYs	Central Europe	Lead exposure	0.13	0.49	0.86	0.009631179	Female
DALYs	Western Europe	Lead exposure	-2.78	-2.42	-2.07	5.55E-14	Female
DALYs	Western Sub-Saharan Africa	Lead exposure	-3.45	-3.14	-2.83	2.24E-18	Female
DALYs	Central Sub-Saharan Africa	Lead exposure	-0.69	-0.6	-0.5	5.97E-13	Female
DALYs	Middle SDI	Lead exposure	-0.93	-0.77	-0.61	1.70E-10	Female
DALYs	East Asia	Lead exposure	-1.65	-1.54	-1.42	6.83E-22	Female
DALYs	Low-middle SDI	Lead exposure	0.03	0.14	0.26	0.019203332	Female
DALYs	Central Latin America	Lead exposure	-1.63	-1.32	-1	2.98E-09	Female
DALYs	Eastern Sub-Saharan Africa	Lead exposure	-1.74	-1.58	-1.43	7.89E-19	Female
DALYs	World Bank Low Income	Lead exposure	-1.21	-1.09	-0.96	9.42E-17	Female
DALYs	High SDI	Lead exposure	-3.36	-3.11	-2.87	4.15E-21	Female
DALYs	South Asia	Lead exposure	-0.05	0.08	0.22	0.205303539	Female
DALYs	Andean Latin America	Lead exposure	-0.56	-0.37	-0.17	0.000720742	Female
DALYs	World Bank High Income	Lead exposure	-3.02	-2.77	-2.51	4.27E-19	Female
DALYs	World Bank Lower Middle Income	Lead exposure	0.2	0.31	0.42	4.72E-06	Female
DALYs	Caribbean	Lead exposure	-0.7	-0.52	-0.33	2.99E-06	Female
DALYs	Southern Sub-Saharan Africa	Lead exposure	-1.78	-1.46	-1.14	4.19E-10	Female
DALYs	Tropical Latin America	Lead exposure	-0.5	-0.14	0.22	0.441721699	Female
DALYs	World Bank Upper Middle Income	Lead exposure	-1.06	-0.87	-0.69	2.17E-10	Female
DALYs	Oceania	High systolic blood pressure	-0.23	-0.04	0.15	0.650593694	Female
DALYs	Southeast Asia	High systolic blood pressure	0.25	0.37	0.48	6.84E-07	Female
DALYs	Global	High systolic blood pressure	-1.36	-1.21	-1.07	1.50E-16	Female
DALYs	High-income North America	High systolic blood pressure	-4.28	-3.79	-3.29	3.41E-15	Female
DALYs	Central Asia	High systolic blood pressure	1.44	1.52	1.59	3.94E-27	Female
DALYs	East Asia	High systolic blood pressure	-0.16	-0.1	-0.05	0.00076796	Female
DALYs	North Africa and Middle East	High systolic blood pressure	-0.77	-0.63	-0.5	2.67E-10	Female
DALYs	Australasia	High systolic blood pressure	-4.44	-4.12	-3.8	5.26E-21	Female
DALYs	Southern Latin America	High systolic blood pressure	0.17	0.48	0.79	0.003377183	Female
DALYs	High-middle SDI	High systolic blood pressure	-0.5	-0.38	-0.26	7.14E-07	Female
DALYs	Low SDI	High systolic blood pressure	-0.85	-0.72	-0.59	7.52E-12	Female
DALYs	Eastern Europe	High systolic blood pressure	0.28	0.49	0.7	6.34E-05	Female
DALYs	Central Europe	High systolic blood pressure	0.05	0.24	0.43	0.015226655	Female
DALYs	High-income Asia Pacific	High systolic blood pressure	0.23	0.37	0.5	4.08E-06	Female
DALYs	Middle SDI	High systolic blood pressure	-0.06	0.04	0.15	0.395689295	Female
DALYs	Low-middle SDI	High systolic blood pressure	0.32	0.38	0.44	1.14E-13	Female
DALYs	Central Latin America	High systolic blood pressure	-0.72	-0.5	-0.28	6.36E-05	Female
DALYs	Eastern Sub-Saharan Africa	High systolic blood pressure	-0.81	-0.64	-0.47	2.83E-08	Female
DALYs	Andean Latin America	High systolic blood pressure	1.17	1.35	1.52	2.30E-15	Female
DALYs	World Bank Low Income	High systolic blood pressure	-1.2	-1.07	-0.94	3.99E-16	Female
DALYs	High SDI	High systolic blood pressure	-2.67	-2.39	-2.11	1.58E-16	Female
DALYs	South Asia	High systolic blood pressure	0.24	0.31	0.37	1.32E-10	Female
DALYs	Western Europe	High systolic blood pressure	-2.75	-2.47	-2.2	4.83E-17	Female
DALYs	Western Sub-Saharan Africa	High systolic blood pressure	-2.77	-2.41	-2.05	8.12E-14	Female
DALYs	World Bank High Income	High systolic blood pressure	-2.41	-2.15	-1.89	3.92E-16	Female
DALYs	World Bank Lower Middle Income	High systolic blood pressure	0.17	0.21	0.24	7.16E-13	Female
DALYs	Southern Sub-Saharan Africa	High systolic blood pressure	-2.53	-2.2	-1.87	8.41E-14	Female
DALYs	Tropical Latin America	High systolic blood pressure	0.55	0.84	1.13	2.19E-06	Female
DALYs	Central Sub-Saharan Africa	High systolic blood pressure	-1.43	-1.32	-1.21	1.96E-20	Female
DALYs	Caribbean	High systolic blood pressure	-0.43	-0.31	-0.2	7.68E-06	Female
DALYs	World Bank Upper Middle Income	High systolic blood pressure	-0.38	-0.28	-0.19	2.39E-06	Female
DALYs	Oceania	Smoking	-1.02	-0.96	-0.9	4.80E-24	Female
DALYs	Southeast Asia	Smoking	-0.4	-0.15	0.1	0.216911603	Female
DALYs	Eastern Europe	Smoking	1.33	1.72	2.11	9.36E-10	Female
DALYs	Global	Smoking	-2.08	-1.94	-1.79	1.64E-21	Female
DALYs	Central Asia	Smoking	0.83	1.06	1.29	2.85E-10	Female
DALYs	North Africa and Middle East	Smoking	-1.42	-1.31	-1.21	6.16E-21	Female
DALYs	Australasia	Smoking	-4.64	-4.35	-4.06	7.44E-23	Female
DALYs	High-income North America	Smoking	-3.41	-3.14	-2.86	9.28E-20	Female

DALYs	Southern Latin America	Smoking	-0.83	-0.68	-0.53	5.57E-10	Female
DALYs	High-middle SDI	Smoking	-0.35	-0.24	-0.12	0.000211834	Female
DALYs	Low SDI	Smoking	-0.63	-0.52	-0.4	7.67E-10	Female
DALYs	Western Europe	Smoking	-2.19	-1.98	-1.76	2.54E-17	Female
DALYs	Central Europe	Smoking	0.32	0.49	0.66	2.02E-06	Female
DALYs	High-income Asia Pacific	Smoking	0.6	0.71	0.82	1.78E-13	Female
DALYs	Central Sub-Saharan Africa	Smoking	-1.92	-1.73	-1.53	4.37E-17	Female
DALYs	Middle SDI	Smoking	-1.18	-0.92	-0.65	1.30E-07	Female
DALYs	East Asia	Smoking	-0.7	-0.37	-0.04	0.027938842	Female
DALYs	Low-middle SDI	Smoking	-0.17	-0.08	0.01	0.071005708	Female
DALYs	Central Latin America	Smoking	-2.73	-2.51	-2.28	1.25E-19	Female
DALYs	Eastern Sub-Saharan Africa	Smoking	-1.61	-1.45	-1.29	2.91E-17	Female
DALYs	World Bank Low Income	Smoking	-1.24	-1.11	-0.97	2.06E-16	Female
DALYs	High SDI	Smoking	-2.57	-2.38	-2.2	2.58E-21	Female
DALYs	South Asia	Smoking	-0.38	-0.3	-0.23	1.02E-08	Female
DALYs	Andean Latin America	Smoking	-1.27	-1.09	-0.91	9.45E-13	Female
DALYs	Western Sub-Saharan Africa	Smoking	-4.12	-3.68	-3.23	4.88E-16	Female
DALYs	World Bank High Income	Smoking	-2.23	-2.04	-1.86	1.31E-19	Female
DALYs	World Bank Lower Middle Income	Smoking	-0.23	-0.19	-0.15	1.84E-10	Female
DALYs	Caribbean	Smoking	-0.99	-0.83	-0.67	4.66E-11	Female
DALYs	Southern Sub-Saharan Africa	Smoking	-4.82	-4.4	-3.97	1.90E-18	Female
DALYs	Tropical Latin America	Smoking	-0.64	-0.34	-0.05	0.022822453	Female
DALYs	World Bank Upper Middle Income	Smoking	-0.65	-0.51	-0.38	2.18E-08	Female
Deaths	Oceania	High systolic blood pressure	-0.47	-0.34	-0.2	2.90E-05	Female
Deaths	Tropical Latin America	High systolic blood pressure	1	1.3	1.6	8.63E-10	Female
Deaths	Global	High systolic blood pressure	-1.39	-1.23	-1.07	1.88E-15	Female
Deaths	North Africa and Middle East	High systolic blood pressure	-0.65	-0.51	-0.37	4.58E-08	Female
Deaths	Western Europe	High systolic blood pressure	-2.75	-2.47	-2.18	1.74E-16	Female
Deaths	South Asia	High systolic blood pressure	0.14	0.23	0.31	5.49E-06	Female
Deaths	Southern Latin America	High systolic blood pressure	0.19	0.53	0.86	0.00304402	Female
Deaths	High-income North America	High systolic blood pressure	-4.09	-3.63	-3.17	1.38E-15	Female
Deaths	Southeast Asia	High systolic blood pressure	0.3	0.42	0.54	9.06E-08	Female
Deaths	Middle SDI	High systolic blood pressure	0.02	0.12	0.21	0.022927315	Female
Deaths	High-middle SDI	High systolic blood pressure	-0.31	-0.18	-0.04	0.011966435	Female
Deaths	Eastern Europe	High systolic blood pressure	0.41	0.68	0.95	2.09E-05	Female
Deaths	Australasia	High systolic blood pressure	-4.18	-3.88	-3.58	3.70E-21	Female
Deaths	East Asia	High systolic blood pressure	-0.17	-0.14	-0.1	1.50E-08	Female
Deaths	Low-middle SDI	High systolic blood pressure	0.31	0.36	0.42	2.87E-13	Female
Deaths	High SDI	High systolic blood pressure	-2.44	-2.17	-1.9	6.80E-16	Female
Deaths	Andean Latin America	High systolic blood pressure	1.39	1.58	1.77	3.19E-16	Female
Deaths	Low SDI	High systolic blood pressure	-0.84	-0.74	-0.63	2.30E-14	Female
Deaths	Caribbean	High systolic blood pressure	-0.51	-0.4	-0.28	1.79E-07	Female
Deaths	High-income Asia Pacific	High systolic blood pressure	0.61	0.77	0.94	2.24E-10	Female
Deaths	Central Asia	High systolic blood pressure	1.84	1.91	1.99	5.49E-29	Female
Deaths	Southern Sub-Saharan Africa	High systolic blood pressure	-2.65	-2.28	-1.9	6.58E-13	Female
Deaths	World Bank Lower Middle Income	High systolic blood pressure	0.17	0.21	0.26	2.76E-10	Female
Deaths	World Bank High Income	High systolic blood pressure	-2.24	-1.98	-1.72	2.49E-15	Female
Deaths	World Bank Upper Middle Income	High systolic blood pressure	-0.15	-0.06	0.04	0.216449788	Female
Deaths	Central Sub-Saharan Africa	High systolic blood pressure	-1.42	-1.31	-1.2	5.99E-20	Female
Deaths	Central Latin America	High systolic blood pressure	-0.46	-0.21	0.04	0.094227319	Female
Deaths	Central Europe	High systolic blood pressure	0.38	0.57	0.76	1.08E-06	Female
Deaths	World Bank Low Income	High systolic blood pressure	-1.18	-1.06	-0.95	1.31E-17	Female
Deaths	Eastern Sub-Saharan Africa	High systolic blood pressure	-0.83	-0.67	-0.51	3.98E-09	Female
Deaths	Western Sub-Saharan Africa	High systolic blood pressure	-2.7	-2.37	-2.04	2.01E-14	Female
Deaths	Oceania	Diet high in sodium	-1.15	-1.02	-0.88	6.13E-15	Female
Deaths	Tropical Latin America	Diet high in sodium	0.57	0.83	1.09	4.37E-07	Female
Deaths	Global	Diet high in sodium	-0.73	-0.65	-0.56	2.10E-15	Female
Deaths	North Africa and Middle East	Diet high in sodium	-0.58	-0.52	-0.45	9.78E-16	Female
Deaths	South Asia	Diet high in sodium	0.16	0.23	0.3	6.75E-07	Female
Deaths	Southern Latin America	Diet high in sodium	-0.83	-0.61	-0.39	4.06E-06	Female
Deaths	High-income North America	Diet high in sodium	-2.85	-2.56	-2.27	6.67E-17	Female
Deaths	Southeast Asia	Diet high in sodium	-0.83	-0.74	-0.64	2.74E-15	Female
Deaths	Middle SDI	Diet high in sodium	-0.8	-0.76	-0.72	6.92E-27	Female
Deaths	High-middle SDI	Diet high in sodium	-0.42	-0.35	-0.27	2.62E-10	Female
Deaths	Australasia	Diet high in sodium	-3.3	-3.05	-2.79	1.98E-20	Female
Deaths	East Asia	Diet high in sodium	-1.1	-1.02	-0.93	1.19E-20	Female
Deaths	Low-middle SDI	Diet high in sodium	-0.1	-0.04	0.02	0.149388953	Female
Deaths	High SDI	Diet high in sodium	-1.04	-0.87	-0.7	5.67E-11	Female
Deaths	Western Europe	Diet high in sodium	-1.74	-1.55	-1.37	2.95E-16	Female
Deaths	Andean Latin America	Diet high in sodium	-0.47	-0.39	-0.31	1.20E-10	Female
Deaths	Low SDI	Diet high in sodium	-1.58	-1.38	-1.17	7.05E-14	Female
Deaths	Eastern Europe	Diet high in sodium	0.44	0.72	1	1.40E-05	Female
Deaths	Caribbean	Diet high in sodium	-1.1	-0.97	-0.83	1.59E-14	Female
Deaths	Central Asia	Diet high in sodium	-0.06	0.1	0.27	0.203194859	Female
Deaths	High-income Asia Pacific	Diet high in sodium	-0.64	-0.49	-0.34	4.04E-07	Female
Deaths	Southern Sub-Saharan Africa	Diet high in sodium	-3.07	-2.75	-2.42	2.08E-16	Female
Deaths	World Bank Lower Middle Income	Diet high in sodium	-0.29	-0.24	-0.19	2.60E-10	Female
Deaths	World Bank High Income	Diet high in sodium	-0.92	-0.75	-0.59	3.66E-10	Female
Deaths	World Bank Upper Middle Income	Diet high in sodium	-0.68	-0.62	-0.56	2.05E-18	Female
Deaths	Central Latin America	Diet high in sodium	-0.85	-0.56	-0.26	0.000673652	Female
Deaths	Central Europe	Diet high in sodium	-0.11	-0.05	0.01	0.08847418	Female
Deaths	Central Sub-Saharan Africa	Diet high in sodium	-1.17	-1	-0.84	8.60E-13	Female
Deaths	World Bank Low Income	Diet high in sodium	-1.89	-1.68	-1.47	1.31E-15	Female
Deaths	Eastern Sub-Saharan Africa	Diet high in sodium	-1.94	-1.71	-1.47	1.02E-14	Female
Deaths	Western Sub-Saharan Africa	Diet high in sodium	-3.08	-2.69	-2.3	3.47E-14	Female
Deaths	Oceania	Lead exposure	-0.88	-0.77	-0.67	5.55E-15	Female
Deaths	Tropical Latin America	Lead exposure	0.33	0.68	1.04	0.000496517	Female
Deaths	Global	Lead exposure	-0.59	-0.46	-0.32	1.48E-07	Female
Deaths	North Africa and Middle East	Lead exposure	-0.85	-0.74	-0.62	2.58E-13	Female
Deaths	Western Europe	Lead exposure	-2.34	-1.98	-1.63	5.38E-12	Female
Deaths	South Asia	Lead exposure	0.27	0.42	0.57	3.25E-06	Female
Deaths	Southern Latin America	Lead exposure	-0.68	-0.4	-0.12	0.007284563	Female
Deaths	High-income North America	Lead exposure	-4.24	-3.95	-3.66	9.91E-22	Female
Deaths	Southeast Asia	Lead exposure	-0.04	0.17	0.38	0.108468294	Female
Deaths	Andean Latin America	Lead exposure	-0.15	0.04	0.24	0.646941988	Female
Deaths	Central Asia	Lead exposure	1.91	2.03	2.16	5.13E-24	Female
Deaths	Middle SDI	Lead exposure	-0.35	-0.19	-0.03	0.018609371	Female
Deaths	High-middle SDI	Lead exposure	-0.31	-0.04	0.23	0.745405439	Female
Deaths	Australasia	Lead exposure	-3.78	-3.47	-3.15	2.85E-19	Female
Deaths	East Asia	Lead exposure	-1.03	-0.93	-0.83	1.34E-17	Female
Deaths	High-income Asia Pacific	Lead exposure	0.42	0.66	0.91	4.98E-06	Female
Deaths	Low-middle SDI	Lead exposure	0.47	0.58	0.7	2.02E-11	Female

Deaths	High SDI	Lead exposure	-2.76	-2.53	-2.3	3.21E-19	Female
Deaths	Low SDI	Lead exposure	0.03	0.13	0.24	0.013124246	Female
Deaths	Eastern Europe	Lead exposure	0.87	1.07	1.27	6.62E-12	Female
Deaths	Caribbean	Lead exposure	-0.42	-0.24	-0.06	0.010861104	Female
Deaths	Central Europe	Lead exposure	0.83	1.18	1.53	1.51E-07	Female
Deaths	Central Latin America	Lead exposure	-1.04	-0.71	-0.39	0.000125276	Female
Deaths	World Bank Upper Middle Income	Lead exposure	-0.36	-0.18	0	0.050052677	Female
Deaths	Southern Sub-Saharan Africa	Lead exposure	-1.73	-1.36	-0.99	4.11E-08	Female
Deaths	World Bank Lower Middle Income	Lead exposure	0.68	0.79	0.9	1.24E-14	Female
Deaths	World Bank High Income	Lead exposure	-2.47	-2.23	-1.98	5.10E-17	Female
Deaths	Central Sub-Saharan Africa	Lead exposure	-0.49	-0.38	-0.27	9.12E-08	Female
Deaths	Western Sub-Saharan Africa	Lead exposure	-3.07	-2.78	-2.48	1.47E-17	Female
Deaths	World Bank Low Income	Lead exposure	-0.96	-0.84	-0.71	2.97E-14	Female
Deaths	Eastern Sub-Saharan Africa	Lead exposure	-1.41	-1.25	-1.1	4.71E-16	Female
Deaths	Oceania	Smoking	-0.98	-0.92	-0.87	4.55E-25	Female
Deaths	Tropical Latin America	Smoking	-0.42	-0.12	0.19	0.450595926	Female
Deaths	Global	Smoking	-2.3	-2.12	-1.94	2.85E-20	Female
Deaths	North Africa and Middle East	Smoking	-1.33	-1.22	-1.12	3.93E-20	Female
Deaths	Western Europe	Smoking	-2.42	-2.17	-1.92	1.18E-16	Female
Deaths	South Asia	Smoking	-0.25	-0.18	-0.1	6.24E-05	Female
Deaths	Southern Latin America	Smoking	-0.79	-0.63	-0.47	8.65E-09	Female
Deaths	High-income North America	Smoking	-3.55	-3.26	-2.96	2.42E-19	Female
Deaths	Southeast Asia	Smoking	-0.57	-0.31	-0.06	0.017433406	Female
Deaths	Andean Latin America	Smoking	-1.23	-1.04	-0.85	7.11E-12	Female
Deaths	Middle SDI	Smoking	-1.11	-0.83	-0.55	1.83E-06	Female
Deaths	High-middle SDI	Smoking	-0.2	-0.07	0.07	0.335498819	Female
Deaths	Australasia	Smoking	-4.71	-4.43	-4.14	4.04E-23	Female
Deaths	East Asia	Smoking	-0.8	-0.44	-0.09	0.016880859	Female
Deaths	Low-middle SDI	Smoking	-0.14	-0.04	0.05	0.336818078	Female
Deaths	High SDI	Smoking	-2.76	-2.54	-2.33	2.42E-20	Female
Deaths	Low SDI	Smoking	-0.54	-0.42	-0.31	3.55E-08	Female
Deaths	Eastern Europe	Smoking	1.46	1.88	2.3	5.33E-10	Female
Deaths	Caribbean	Smoking	-0.91	-0.76	-0.61	6.68E-11	Female
Deaths	High-income Asia Pacific	Smoking	0.41	0.49	0.57	1.66E-13	Female
Deaths	Central Europe	Smoking	0.61	0.76	0.91	5.46E-11	Female
Deaths	World Bank Upper Middle Income	Smoking	-0.51	-0.35	-0.18	0.000163983	Female
Deaths	Central Asia	Smoking	1.01	1.23	1.45	4.36E-12	Female
Deaths	Southern Sub-Saharan Africa	Smoking	-4.88	-4.42	-3.95	1.32E-17	Female
Deaths	World Bank Lower Middle Income	Smoking	-0.09	-0.05	0	0.033267317	Female
Deaths	World Bank High Income	Smoking	-2.45	-2.24	-2.03	5.38E-19	Female
Deaths	Central Sub-Saharan Africa	Smoking	-1.99	-1.79	-1.59	5.77E-17	Female
Deaths	Central Latin America	Smoking	-2.58	-2.36	-2.13	8.10E-19	Female
Deaths	Western Sub-Saharan Africa	Smoking	-4.26	-3.83	-3.4	5.74E-17	Female
Deaths	World Bank Low Income	Smoking	-1.22	-1.1	-0.98	2.46E-17	Female
Deaths	Eastern Sub-Saharan Africa	Smoking	-1.62	-1.47	-1.31	1.12E-17	Female
DALYs	Oceania	High systolic blood pressure	-0.16	-0.03	0.11	0.676948875	Both
DALYs	Southeast Asia	High systolic blood pressure	0.84	0.91	0.98	1.58E-21	Both
DALYs	Global	High systolic blood pressure	-1.43	-1.31	-1.19	2.44E-19	Both
DALYs	High-income North America	High systolic blood pressure	-4.69	-4.25	-3.82	6.81E-18	Both
DALYs	Central Asia	High systolic blood pressure	1.61	1.73	1.84	1.85E-23	Both
DALYs	East Asia	High systolic blood pressure	0.61	0.69	0.78	5.79E-16	Both
DALYs	North Africa and Middle East	High systolic blood pressure	-0.85	-0.73	-0.61	6.97E-13	Both
DALYs	Australasia	High systolic blood pressure	-5.18	-4.82	-4.45	2.11E-21	Both
DALYs	Southern Latin America	High systolic blood pressure	-0.56	-0.22	0.12	0.192744666	Both
DALYs	High-middle SDI	High systolic blood pressure	-0.59	-0.39	-0.2	0.00028434	Both
DALYs	Low SDI	High systolic blood pressure	-0.35	-0.27	-0.18	2.78E-07	Both
DALYs	Eastern Europe	High systolic blood pressure	0.84	1.07	1.31	4.28E-10	Both
DALYs	Central Europe	High systolic blood pressure	0.28	0.56	0.84	0.000278526	Both
DALYs	High-income Asia Pacific	High systolic blood pressure	0.15	0.25	0.36	4.60E-05	Both
DALYs	Middle SDI	High systolic blood pressure	0.26	0.35	0.45	5.49E-08	Both
DALYs	Low-middle SDI	High systolic blood pressure	0.57	0.63	0.69	1.19E-19	Both
DALYs	Central Latin America	High systolic blood pressure	-0.74	-0.53	-0.32	1.70E-05	Both
DALYs	Eastern Sub-Saharan Africa	High systolic blood pressure	-0.55	-0.42	-0.29	2.26E-07	Both
DALYs	Andean Latin America	High systolic blood pressure	0.89	1.11	1.34	4.50E-11	Both
DALYs	World Bank Low Income	High systolic blood pressure	-0.73	-0.63	-0.53	5.13E-13	Both
DALYs	High SDI	High systolic blood pressure	-3.06	-2.83	-2.59	3.02E-20	Both
DALYs	South Asia	High systolic blood pressure	0.33	0.41	0.49	1.82E-11	Both
DALYs	Western Europe	High systolic blood pressure	-3.25	-2.96	-2.67	2.24E-18	Both
DALYs	Western Sub-Saharan Africa	High systolic blood pressure	-0.55	-0.42	-0.29	5.38E-07	Both
DALYs	World Bank High Income	High systolic blood pressure	-2.72	-2.5	-2.29	5.69E-20	Both
DALYs	World Bank Lower Middle Income	High systolic blood pressure	0.44	0.48	0.53	2.49E-19	Both
DALYs	Southern Sub-Saharan Africa	High systolic blood pressure	-2.45	-1.95	-1.44	1.56E-08	Both
DALYs	Tropical Latin America	High systolic blood pressure	0.12	0.4	0.69	0.006365611	Both
DALYs	Central Sub-Saharan Africa	High systolic blood pressure	-1.47	-1.32	-1.18	3.93E-17	Both
DALYs	Caribbean	High systolic blood pressure	-0.55	-0.41	-0.26	4.33E-06	Both
DALYs	World Bank Upper Middle Income	High systolic blood pressure	-0.19	-0.06	0.06	0.308003356	Both
DALYs	Oceania	Diet high in sodium	-0.78	-0.64	-0.5	4.66E-10	Both
DALYs	Southeast Asia	Diet high in sodium	-0.56	-0.47	-0.37	8.37E-11	Both
DALYs	Global	Diet high in sodium	-0.85	-0.77	-0.7	7.36E-19	Both
DALYs	Central Asia	Diet high in sodium	-0.02	0.12	0.26	0.096484269	Both
DALYs	East Asia	Diet high in sodium	-0.42	-0.34	-0.25	8.33E-09	Both
DALYs	Australasia	Diet high in sodium	-4.19	-3.91	-3.62	1.11E-21	Both
DALYs	Southern Latin America	Diet high in sodium	-1.56	-1.26	-0.95	3.74E-09	Both
DALYs	High-middle SDI	Diet high in sodium	-0.44	-0.34	-0.24	2.70E-07	Both
DALYs	Low SDI	Diet high in sodium	-1.57	-1.39	-1.22	1.32E-15	Both
DALYs	Eastern Europe	Diet high in sodium	0.83	1.12	1.41	9.49E-09	Both
DALYs	Central Europe	Diet high in sodium	-0.02	0.08	0.19	0.105225827	Both
DALYs	High-income Asia Pacific	Diet high in sodium	-1.43	-1.28	-1.13	7.05E-17	Both
DALYs	Middle SDI	Diet high in sodium	-0.42	-0.39	-0.36	1.15E-21	Both
DALYs	North Africa and Middle East	Diet high in sodium	-0.85	-0.74	-0.62	1.93E-13	Both
DALYs	Low-middle SDI	Diet high in sodium	0.29	0.35	0.4	7.75E-14	Both
DALYs	Central Latin America	Diet high in sodium	-1.34	-1.05	-0.76	4.52E-08	Both
DALYs	Andean Latin America	Diet high in sodium	-0.43	-0.33	-0.22	4.73E-07	Both
DALYs	Eastern Sub-Saharan Africa	Diet high in sodium	-2.8	-2.58	-2.36	5.32E-20	Both
DALYs	High SDI	Diet high in sodium	-1.74	-1.57	-1.39	3.41E-17	Both
DALYs	World Bank Low Income	Diet high in sodium	-2.17	-2	-1.84	1.49E-20	Both
DALYs	South Asia	Diet high in sodium	0.6	0.69	0.79	4.16E-15	Both
DALYs	Western Europe	Diet high in sodium	-2.19	-1.99	-1.79	3.03E-18	Both
DALYs	World Bank Lower Middle Income	Diet high in sodium	0.18	0.22	0.25	2.38E-12	Both
DALYs	World Bank High Income	Diet high in sodium	-1.47	-1.3	-1.13	1.66E-15	Both
DALYs	High-income North America	Diet high in sodium	-1.83	-1.56	-1.29	2.80E-12	Both
DALYs	Western Sub-Saharan Africa	Diet high in sodium	-1.14	-0.96	-0.78	1.82E-11	Both

DALYs	Tropical Latin America	Diet high in sodium	-0.27	-0.01	0.26	0.956151905	Both
DALYs	Central Sub-Saharan Africa	Diet high in sodium	-1.23	-1.04	-0.86	3.71E-12	Both
DALYs	Caribbean	Diet high in sodium	-0.94	-0.78	-0.62	1.34E-10	Both
DALYs	World Bank Upper Middle Income	Diet high in sodium	-0.52	-0.46	-0.4	6.39E-16	Both
DALYs	Southern Sub-Saharan Africa	Diet high in sodium	-3.14	-2.71	-2.29	2.97E-13	Both
DALYs	Oceania	Lead exposure	-0.91	-0.81	-0.71	3.46E-16	Both
DALYs	Southeast Asia	Lead exposure	0.12	0.31	0.5	0.002697427	Both
DALYs	Eastern Europe	Lead exposure	0.72	1.06	1.4	6.52E-07	Both
DALYs	Global	Lead exposure	-1.41	-1.26	-1.11	1.32E-16	Both
DALYs	Central Asia	Lead exposure	1.42	1.65	1.88	8.24E-15	Both
DALYs	North Africa and Middle East	Lead exposure	-1.4	-1.27	-1.14	1.81E-18	Both
DALYs	Australasia	Lead exposure	-5.18	-4.9	-4.63	7.36E-25	Both
DALYs	High-income North America	Lead exposure	-5.91	-5.63	-5.34	4.97E-26	Both
DALYs	High-income Asia Pacific	Lead exposure	-1.27	-1.04	-0.81	4.07E-10	Both
DALYs	Southern Latin America	Lead exposure	-2.3	-1.84	-1.38	7.21E-09	Both
DALYs	High-middle SDI	Lead exposure	-1.42	-1.08	-0.74	4.40E-07	Both
DALYs	Low SDI	Lead exposure	-0.68	-0.61	-0.55	2.07E-17	Both
DALYs	Central Europe	Lead exposure	-0.29	0.22	0.73	0.381429371	Both
DALYs	Western Europe	Lead exposure	-3.77	-3.34	-2.91	2.29E-15	Both
DALYs	Western Sub-Saharan Africa	Lead exposure	-1.17	-1.05	-0.92	2.25E-16	Both
DALYs	Central Sub-Saharan Africa	Lead exposure	-1.15	-1.02	-0.9	5.50E-16	Both
DALYs	Middle SDI	Lead exposure	-0.9	-0.72	-0.54	7.11E-09	Both
DALYs	East Asia	Lead exposure	-1.15	-0.98	-0.8	4.05E-12	Both
DALYs	Low-middle SDI	Lead exposure	-0.24	-0.12	0	0.041929614	Both
DALYs	Central Latin America	Lead exposure	-2.1	-1.81	-1.52	3.25E-13	Both
DALYs	Eastern Sub-Saharan Africa	Lead exposure	-2.44	-2.3	-2.15	1.34E-23	Both
DALYs	World Bank Low Income	Lead exposure	-1.42	-1.32	-1.21	4.87E-21	Both
DALYs	High SDI	Lead exposure	-4.33	-4.09	-3.85	2.15E-24	Both
DALYs	South Asia	Lead exposure	-0.4	-0.26	-0.13	0.000384161	Both
DALYs	Andean Latin America	Lead exposure	-0.71	-0.57	-0.43	5.06E-09	Both
DALYs	World Bank High Income	Lead exposure	-3.86	-3.57	-3.28	1.29E-20	Both
DALYs	World Bank Lower Middle Income	Lead exposure	-0.12	0	0.13	0.959349945	Both
DALYs	Caribbean	Lead exposure	-1.04	-0.85	-0.66	5.27E-10	Both
DALYs	Southern Sub-Saharan Africa	Lead exposure	-2.47	-1.91	-1.35	1.65E-07	Both
DALYs	Tropical Latin America	Lead exposure	-1.26	-0.91	-0.55	1.49E-05	Both
DALYs	World Bank Upper Middle Income	Lead exposure	-1.13	-0.92	-0.71	1.27E-09	Both
DALYs	Oceania	Smoking	-0.89	-0.86	-0.83	9.94E-31	Both
DALYs	Southeast Asia	Smoking	0.36	0.44	0.52	7.97E-12	Both
DALYs	Eastern Europe	Smoking	1.1	1.38	1.66	6.46E-11	Both
DALYs	Global	Smoking	-1.81	-1.69	-1.57	1.80E-22	Both
DALYs	Central Asia	Smoking	1.31	1.47	1.63	1.06E-17	Both
DALYs	North Africa and Middle East	Smoking	-1.56	-1.4	-1.23	1.32E-16	Both
DALYs	Australasia	Smoking	-5.33	-5.02	-4.71	1.01E-23	Both
DALYs	High-income North America	Smoking	-3.99	-3.74	-3.48	1.11E-22	Both
DALYs	Southern Latin America	Smoking	-1.93	-1.64	-1.36	2.73E-12	Both
DALYs	High-middle SDI	Smoking	-0.89	-0.69	-0.48	2.08E-07	Both
DALYs	Low SDI	Smoking	-1.03	-0.93	-0.83	1.86E-17	Both
DALYs	Western Europe	Smoking	-2.98	-2.72	-2.47	4.41E-19	Both
DALYs	Central Europe	Smoking	0.1	0.41	0.72	0.010450962	Both
DALYs	High-income Asia Pacific	Smoking	0.39	0.56	0.74	2.79E-07	Both
DALYs	Central Sub-Saharan Africa	Smoking	-2.07	-1.81	-1.56	2.31E-14	Both
DALYs	Middle SDI	Smoking	-0.57	-0.45	-0.33	2.63E-08	Both
DALYs	East Asia	Smoking	-0.08	0	0.08	0.995426142	Both
DALYs	Low-middle SDI	Smoking	-0.22	-0.16	-0.1	9.50E-06	Both
DALYs	Central Latin America	Smoking	-2.36	-2.17	-1.98	1.40E-19	Both
DALYs	Eastern Sub-Saharan Africa	Smoking	-2.1	-1.95	-1.79	7.55E-21	Both
DALYs	World Bank Low Income	Smoking	-1.42	-1.29	-1.16	1.50E-18	Both
DALYs	High SDI	Smoking	-2.79	-2.65	-2.5	3.00E-25	Both
DALYs	South Asia	Smoking	-0.6	-0.52	-0.43	8.72E-13	Both
DALYs	Andean Latin America	Smoking	-1.26	-1.11	-0.96	4.98E-15	Both
DALYs	Western Sub-Saharan Africa	Smoking	-0.95	-0.85	-0.75	4.18E-16	Both
DALYs	World Bank High Income	Smoking	-2.51	-2.35	-2.18	2.10E-22	Both
DALYs	World Bank Lower Middle Income	Smoking	-0.22	-0.15	-0.09	2.42E-05	Both
DALYs	Caribbean	Smoking	-1.14	-0.98	-0.81	1.04E-12	Both
DALYs	Southern Sub-Saharan Africa	Smoking	-3.75	-3.2	-2.64	2.86E-12	Both
DALYs	Tropical Latin America	Smoking	-1.07	-0.78	-0.48	8.67E-06	Both
DALYs	World Bank Upper Middle Income	Smoking	-0.77	-0.64	-0.5	2.05E-10	Both
Deaths	Oceania	High systolic blood pressure	-0.36	-0.26	-0.17	3.49E-06	Both
Deaths	Tropical Latin America	High systolic blood pressure	0.44	0.73	1.02	1.77E-05	Both
Deaths	Global	High systolic blood pressure	-1.57	-1.43	-1.3	1.19E-18	Both
Deaths	North Africa and Middle East	High systolic blood pressure	-0.58	-0.47	-0.36	2.39E-09	Both
Deaths	Western Europe	High systolic blood pressure	-3.27	-2.96	-2.65	1.18E-17	Both
Deaths	South Asia	High systolic blood pressure	0.28	0.37	0.45	1.49E-09	Both
Deaths	Southern Latin America	High systolic blood pressure	-0.51	-0.15	0.23	0.428199435	Both
Deaths	High-income North America	High systolic blood pressure	-4.83	-4.42	-4.01	5.20E-19	Both
Deaths	Southeast Asia	High systolic blood pressure	0.85	0.92	0.99	6.71E-22	Both
Deaths	Middle SDI	High systolic blood pressure	0.28	0.38	0.47	1.41E-08	Both
Deaths	High-middle SDI	High systolic blood pressure	-0.51	-0.31	-0.11	0.004282747	Both
Deaths	Eastern Europe	High systolic blood pressure	0.91	1.13	1.36	4.05E-11	Both
Deaths	Australasia	High systolic blood pressure	-4.97	-4.63	-4.29	1.25E-21	Both
Deaths	East Asia	High systolic blood pressure	0.52	0.59	0.66	3.87E-16	Both
Deaths	Low-middle SDI	High systolic blood pressure	0.55	0.6	0.66	9.52E-19	Both
Deaths	High SDI	High systolic blood pressure	-3.05	-2.81	-2.57	6.40E-20	Both
Deaths	Andean Latin America	High systolic blood pressure	1.16	1.39	1.61	3.26E-13	Both
Deaths	Low SDI	High systolic blood pressure	-0.32	-0.26	-0.19	6.70E-09	Both
Deaths	Caribbean	High systolic blood pressure	-0.66	-0.52	-0.37	8.79E-08	Both
Deaths	High-income Asia Pacific	High systolic blood pressure	0.18	0.31	0.44	2.99E-05	Both
Deaths	Central Asia	High systolic blood pressure	2.06	2.16	2.26	1.42E-27	Both
Deaths	Southern Sub-Saharan Africa	High systolic blood pressure	-2.47	-1.97	-1.46	1.42E-08	Both
Deaths	World Bank Lower Middle Income	High systolic blood pressure	0.44	0.49	0.54	7.69E-18	Both
Deaths	World Bank High Income	High systolic blood pressure	-2.75	-2.52	-2.29	2.80E-19	Both
Deaths	World Bank Upper Middle Income	High systolic blood pressure	-0.05	0.07	0.19	0.223976812	Both
Deaths	Central Sub-Saharan Africa	High systolic blood pressure	-1.46	-1.31	-1.17	2.19E-17	Both
Deaths	Central Latin America	High systolic blood pressure	-0.57	-0.34	-0.11	0.00511264	Both
Deaths	Central Europe	High systolic blood pressure	0.5	0.78	1.07	5.20E-06	Both
Deaths	World Bank Low Income	High systolic blood pressure	-0.69	-0.6	-0.51	1.47E-13	Both
Deaths	Eastern Sub-Saharan Africa	High systolic blood pressure	-0.52	-0.39	-0.27	4.09E-07	Both
Deaths	Western Sub-Saharan Africa	High systolic blood pressure	-0.51	-0.39	-0.26	7.93E-07	Both
Deaths	Oceania	Diet high in sodium	-0.98	-0.87	-0.75	2.78E-15	Both
Deaths	Tropical Latin America	Diet high in sodium	-0.01	0.26	0.53	0.055417049	Both
Deaths	Global	Diet high in sodium	-0.8	-0.72	-0.63	2.28E-16	Both
Deaths	North Africa and Middle East	Diet high in sodium	-0.6	-0.5	-0.4	3.11E-11	Both

Deaths	South Asia	Diet high in sodium	0.45	0.53	0.6	2.53E-14	Both
Deaths	Southern Latin America	Diet high in sodium	-1.54	-1.22	-0.9	1.69E-08	Both
Deaths	High-income North America	Diet high in sodium	-2.35	-2.06	-1.77	2.15E-14	Both
Deaths	Southeast Asia	Diet high in sodium	-0.39	-0.31	-0.23	8.09E-09	Both
Deaths	Middle SDI	Diet high in sodium	-0.35	-0.31	-0.28	6.93E-18	Both
Deaths	High-middle SDI	Diet high in sodium	-0.32	-0.22	-0.12	0.000118236	Both
Deaths	Australasia	Diet high in sodium	-4.14	-3.85	-3.57	9.90E-22	Both
Deaths	East Asia	Diet high in sodium	-0.44	-0.38	-0.31	2.15E-12	Both
Deaths	Low-middle SDI	Diet high in sodium	0.26	0.3	0.35	1.97E-14	Both
Deaths	High SDI	Diet high in sodium	-1.63	-1.45	-1.28	4.35E-16	Both
Deaths	Western Europe	Diet high in sodium	-2.28	-2.07	-1.85	9.90E-18	Both
Deaths	Andean Latin America	Diet high in sodium	-0.25	-0.15	-0.05	0.003760953	Both
Deaths	Low SDI	Diet high in sodium	-1.46	-1.31	-1.15	1.78E-16	Both
Deaths	Eastern Europe	Diet high in sodium	0.96	1.22	1.48	2.42E-10	Both
Deaths	Caribbean	Diet high in sodium	-1.14	-0.97	-0.8	2.90E-12	Both
Deaths	Central Asia	Diet high in sodium	0.46	0.59	0.73	1.22E-09	Both
Deaths	High-income Asia Pacific	Diet high in sodium	-1.22	-1.09	-0.95	7.29E-16	Both
Deaths	Southern Sub-Saharan Africa	Diet high in sodium	-2.99	-2.55	-2.11	2.25E-12	Both
Deaths	World Bank Lower Middle Income	Diet high in sodium	0.12	0.16	0.2	1.68E-08	Both
Deaths	World Bank High Income	Diet high in sodium	-1.39	-1.22	-1.05	1.68E-14	Both
Deaths	World Bank Upper Middle Income	Diet high in sodium	-0.42	-0.36	-0.31	8.77E-14	Both
Deaths	Central Latin America	Diet high in sodium	-1.1	-0.8	-0.51	6.59E-06	Both
Deaths	Central Europe	Diet high in sodium	0.26	0.38	0.5	2.67E-07	Both
Deaths	Central Sub-Saharan Africa	Diet high in sodium	-1.22	-1.04	-0.85	4.66E-12	Both
Deaths	World Bank Low Income	Diet high in sodium	-1.94	-1.78	-1.62	1.09E-19	Both
Deaths	Eastern Sub-Saharan Africa	Diet high in sodium	-2.5	-2.3	-2.1	1.21E-19	Both
Deaths	Western Sub-Saharan Africa	Diet high in sodium	-1	-0.84	-0.68	2.68E-11	Both
Deaths	Oceania	Lead exposure	-0.78	-0.69	-0.6	1.60E-15	Both
Deaths	Tropical Latin America	Lead exposure	-0.54	-0.2	0.15	0.248896864	Both
Deaths	Global	Lead exposure	-1.1	-0.95	-0.8	2.46E-13	Both
Deaths	North Africa and Middle East	Lead exposure	-0.77	-0.65	-0.53	1.20E-11	Both
Deaths	Western Europe	Lead exposure	-3.2	-2.77	-2.35	1.28E-13	Both
Deaths	South Asia	Lead exposure	-0.04	0.1	0.25	0.162094615	Both
Deaths	Southern Latin America	Lead exposure	-1.81	-1.35	-0.9	1.74E-06	Both
Deaths	High-income North America	Lead exposure	-5.44	-5.15	-4.86	7.00E-25	Both
Deaths	Southeast Asia	Lead exposure	0.5	0.68	0.86	1.48E-08	Both
Deaths	Andean Latin America	Lead exposure	-0.14	0	0.15	0.944966289	Both
Deaths	Central Asia	Lead exposure	2	2.2	2.4	1.21E-19	Both
Deaths	Middle SDI	Lead exposure	-0.36	-0.17	0.01	0.05869488	Both
Deaths	High-middle SDI	Lead exposure	-0.82	-0.47	-0.13	0.009345692	Both
Deaths	Australasia	Lead exposure	-4.61	-4.32	-4.03	7.85E-23	Both
Deaths	East Asia	Lead exposure	-0.57	-0.4	-0.24	2.85E-05	Both
Deaths	High-income Asia Pacific	Lead exposure	-0.52	-0.3	-0.07	0.010854266	Both
Deaths	Low-middle SDI	Lead exposure	0.19	0.3	0.41	8.46E-06	Both
Deaths	High SDI	Lead exposure	-3.81	-3.58	-3.34	6.05E-23	Both
Deaths	Low SDI	Lead exposure	-0.31	-0.24	-0.18	1.58E-08	Both
Deaths	Eastern Europe	Lead exposure	1.1	1.38	1.65	3.65E-11	Both
Deaths	Caribbean	Lead exposure	-0.79	-0.59	-0.4	7.69E-07	Both
Deaths	Central Europe	Lead exposure	0.36	0.86	1.36	0.001512242	Both
Deaths	Central Latin America	Lead exposure	-1.5	-1.21	-0.92	3.99E-09	Both
Deaths	World Bank Upper Middle Income	Lead exposure	-0.51	-0.3	-0.09	0.007036606	Both
Deaths	Southern Sub-Saharan Africa	Lead exposure	-2.23	-1.67	-1.11	1.65E-06	Both
Deaths	World Bank Lower Middle Income	Lead exposure	0.35	0.47	0.59	1.21E-08	Both
Deaths	World Bank High Income	Lead exposure	-3.36	-3.08	-2.81	2.40E-19	Both
Deaths	Central Sub-Saharan Africa	Lead exposure	-0.93	-0.8	-0.67	6.47E-13	Both
Deaths	Western Sub-Saharan Africa	Lead exposure	-0.81	-0.69	-0.57	1.77E-12	Both
Deaths	World Bank Low Income	Lead exposure	-1.11	-1.01	-0.91	2.52E-18	Both
Deaths	Eastern Sub-Saharan Africa	Lead exposure	-2.03	-1.89	-1.75	8.85E-22	Both
Deaths	Oceania	Smoking	-0.94	-0.9	-0.86	1.57E-28	Both
Deaths	Tropical Latin America	Smoking	-0.95	-0.64	-0.33	0.000225011	Both
Deaths	Global	Smoking	-2.03	-1.89	-1.75	7.72E-22	Both
Deaths	North Africa and Middle East	Smoking	-1.25	-1.11	-0.97	9.98E-16	Both
Deaths	Western Europe	Smoking	-3.14	-2.86	-2.57	2.48E-18	Both
Deaths	South Asia	Smoking	-0.72	-0.62	-0.53	3.99E-14	Both
Deaths	Southern Latin America	Smoking	-2	-1.7	-1.4	3.34E-12	Both
Deaths	High-income North America	Smoking	-4.41	-4.12	-3.84	1.71E-22	Both
Deaths	Southeast Asia	Smoking	0.29	0.37	0.45	1.09E-10	Both
Deaths	Andean Latin America	Smoking	-1.2	-1.05	-0.9	2.26E-14	Both
Deaths	Middle SDI	Smoking	-0.58	-0.45	-0.31	3.67E-07	Both
Deaths	High-middle SDI	Smoking	-0.85	-0.63	-0.4	4.29E-06	Both
Deaths	Australasia	Smoking	-5.56	-5.26	-4.95	2.53E-24	Both
Deaths	East Asia	Smoking	-0.18	-0.06	0.05	0.280844321	Both
Deaths	Low-middle SDI	Smoking	-0.29	-0.22	-0.16	1.10E-07	Both
Deaths	High SDI	Smoking	-3.09	-2.92	-2.74	3.11E-24	Both
Deaths	Low SDI	Smoking	-1	-0.91	-0.81	1.54E-17	Both
Deaths	Eastern Europe	Smoking	1.24	1.5	1.75	1.55E-12	Both
Deaths	Caribbean	Smoking	-1.22	-1.07	-0.91	6.73E-14	Both
Deaths	High-income Asia Pacific	Smoking	0.02	0.18	0.34	0.027122726	Both
Deaths	Central Europe	Smoking	0.29	0.61	0.93	0.000526224	Both
Deaths	World Bank Upper Middle Income	Smoking	-0.7	-0.55	-0.41	1.25E-08	Both
Deaths	Central Asia	Smoking	1.76	1.9	2.04	6.63E-22	Both
Deaths	Southern Sub-Saharan Africa	Smoking	-3.93	-3.36	-2.79	1.99E-12	Both
Deaths	World Bank Lower Middle Income	Smoking	-0.22	-0.16	-0.09	4.03E-05	Both
Deaths	World Bank High Income	Smoking	-2.79	-2.6	-2.4	9.07E-22	Both
Deaths	Central Sub-Saharan Africa	Smoking	-2.2	-1.93	-1.66	1.89E-14	Both
Deaths	Central Latin America	Smoking	-2.27	-2.08	-1.89	2.65E-19	Both
Deaths	Western Sub-Saharan Africa	Smoking	-0.84	-0.75	-0.66	2.89E-16	Both
Deaths	World Bank Low Income	Smoking	-1.41	-1.29	-1.16	1.29E-18	Both
Deaths	Eastern Sub-Saharan Africa	Smoking	-2.13	-1.97	-1.81	7.85E-21	Both
DALYs	Oceania	Diet high in sodium	-0.78	-0.65	-0.51	1.35E-10	Male
DALYs	Southeast Asia	Diet high in sodium	-0.34	-0.25	-0.17	2.52E-06	Male
DALYs	Global	Diet high in sodium	-0.88	-0.81	-0.74	1.18E-19	Male
DALYs	Central Asia	Diet high in sodium	0.06	0.21	0.37	0.008573795	Male
DALYs	East Asia	Diet high in sodium	-0.22	-0.12	-0.02	0.02322061	Male
DALYs	Australasia	Diet high in sodium	-4.54	-4.22	-3.91	1.55E-21	Male
DALYs	Southern Latin America	Diet high in sodium	-1.78	-1.44	-1.1	2.76E-09	Male
DALYs	High-middle SDI	Diet high in sodium	-0.52	-0.4	-0.29	5.63E-08	Male
DALYs	Low SDI	Diet high in sodium	-1.45	-1.31	-1.16	3.45E-17	Male
DALYs	Eastern Europe	Diet high in sodium	0.82	1.12	1.43	2.44E-08	Male
DALYs	Central Europe	Diet high in sodium	0.05	0.18	0.31	0.009862576	Male
DALYs	High-income Asia Pacific	Diet high in sodium	-1.77	-1.62	-1.47	2.00E-19	Male
DALYs	Middle SDI	Diet high in sodium	-0.23	-0.2	-0.17	3.02E-13	Male

DALYs	North Africa and Middle East	Diet high in sodium	-0.89	-0.77	-0.64	4.34E-13	Male
DALYs	Low-middle SDI	Diet high in sodium	0.52	0.58	0.64	1.73E-18	Male
DALYs	Central Latin America	Diet high in sodium	-1.25	-0.96	-0.67	2.90E-07	Male
DALYs	Andean Latin America	Diet high in sodium	-0.38	-0.25	-0.12	0.000513566	Male
DALYs	Eastern Sub-Saharan Africa	Diet high in sodium	-3.21	-3.01	-2.81	6.95E-23	Male
DALYs	High SDI	Diet high in sodium	-2.05	-1.88	-1.72	1.49E-19	Male
DALYs	World Bank Low Income	Diet high in sodium	-2.17	-2.04	-1.9	2.81E-23	Male
DALYs	South Asia	Diet high in sodium	0.82	0.93	1.04	9.57E-17	Male
DALYs	Western Europe	Diet high in sodium	-2.57	-2.36	-2.15	1.26E-19	Male
DALYs	World Bank Lower Middle Income	Diet high in sodium	0.37	0.41	0.46	6.90E-18	Male
DALYs	World Bank High Income	Diet high in sodium	-1.74	-1.57	-1.41	1.25E-17	Male
DALYs	High-income North America	Diet high in sodium	-1.77	-1.48	-1.2	3.51E-11	Male
DALYs	Western Sub-Saharan Africa	Diet high in sodium	0.01	0.1	0.19	0.025776499	Male
DALYs	Tropical Latin America	Diet high in sodium	-0.37	-0.09	0.19	0.497374036	Male
DALYs	Central Sub-Saharan Africa	Diet high in sodium	-1.19	-1.02	-0.84	2.78E-12	Male
DALYs	Caribbean	Diet high in sodium	-0.91	-0.73	-0.55	3.55E-09	Male
DALYs	World Bank Upper Middle Income	Diet high in sodium	-0.45	-0.39	-0.33	1.29E-13	Male
DALYs	Southern Sub-Saharan Africa	Diet high in sodium	-3.08	-2.56	-2.03	1.33E-10	Male
DALYs	Oceania	Lead exposure	-0.93	-0.83	-0.73	2.88E-16	Male
DALYs	Southeast Asia	Lead exposure	0.39	0.57	0.75	5.86E-07	Male
DALYs	Eastern Europe	Lead exposure	0.67	1.01	1.36	1.54E-06	Male
DALYs	Global	Lead exposure	-1.59	-1.44	-1.29	6.88E-18	Male
DALYs	Central Asia	Lead exposure	1.36	1.61	1.85	8.66E-14	Male
DALYs	North Africa and Middle East	Lead exposure	-1.4	-1.27	-1.14	6.72E-18	Male
DALYs	Australasia	Lead exposure	-5.6	-5.32	-5.03	1.70E-25	Male
DALYs	High-income North America	Lead exposure	-6.41	-6.11	-5.82	1.52E-26	Male
DALYs	High-income Asia Pacific	Lead exposure	-1.71	-1.47	-1.23	6.01E-13	Male
DALYs	Southern Latin America	Lead exposure	-2.48	-1.98	-1.48	1.06E-08	Male
DALYs	High-middle SDI	Lead exposure	-1.57	-1.22	-0.86	1.40E-07	Male
DALYs	Low SDI	Lead exposure	-0.72	-0.65	-0.58	2.05E-17	Male
DALYs	Central Europe	Lead exposure	-0.36	0.19	0.74	0.480504654	Male
DALYs	Western Europe	Lead exposure	-4.16	-3.72	-3.27	3.72E-16	Male
DALYs	Western Sub-Saharan Africa	Lead exposure	-0.19	-0.08	0.04	0.200744919	Male
DALYs	Central Sub-Saharan Africa	Lead exposure	-1.2	-1.07	-0.94	5.67E-16	Male
DALYs	Middle SDI	Lead exposure	-0.82	-0.64	-0.46	5.78E-08	Male
DALYs	East Asia	Lead exposure	-0.94	-0.76	-0.58	2.22E-09	Male
DALYs	Low-middle SDI	Lead exposure	-0.19	-0.07	0.04	0.210689251	Male
DALYs	Central Latin America	Lead exposure	-2.06	-1.78	-1.49	4.07E-13	Male
DALYs	Eastern Sub-Saharan Africa	Lead exposure	-2.5	-2.35	-2.21	1.17E-23	Male
DALYs	World Bank Low Income	Lead exposure	-1.37	-1.27	-1.17	5.16E-21	Male
DALYs	High SDI	Lead exposure	-4.8	-4.56	-4.31	2.16E-25	Male
DALYs	South Asia	Lead exposure	-0.37	-0.22	-0.08	0.004012943	Male
DALYs	Andean Latin America	Lead exposure	-0.74	-0.61	-0.48	2.76E-10	Male
DALYs	World Bank High Income	Lead exposure	-4.24	-3.95	-3.65	2.22E-21	Male
DALYs	World Bank Lower Middle Income	Lead exposure	-0.14	-0.01	0.12	0.904430402	Male
DALYs	Caribbean	Lead exposure	-1.07	-0.87	-0.68	7.29E-10	Male
DALYs	Southern Sub-Saharan Africa	Lead exposure	-2.47	-1.82	-1.16	4.73E-06	Male
DALYs	Tropical Latin America	Lead exposure	-1.48	-1.11	-0.75	1.01E-06	Male
DALYs	World Bank Upper Middle Income	Lead exposure	-1.13	-0.92	-0.71	1.29E-09	Male
DALYs	Oceania	Smoking	-0.89	-0.85	-0.81	1.34E-26	Male
DALYs	Southeast Asia	Smoking	0.47	0.54	0.61	1.49E-15	Male
DALYs	Eastern Europe	Smoking	0.88	1.17	1.45	3.25E-09	Male
DALYs	Global	Smoking	-1.85	-1.74	-1.63	1.73E-23	Male
DALYs	Central Asia	Smoking	1.29	1.45	1.6	1.22E-17	Male
DALYs	North Africa and Middle East	Smoking	-1.58	-1.42	-1.25	1.04E-16	Male
DALYs	Australasia	Smoking	-5.73	-5.4	-5.06	1.38E-23	Male
DALYs	High-income North America	Smoking	-4.43	-4.16	-3.88	3.75E-23	Male
DALYs	Southern Latin America	Smoking	-2.27	-1.94	-1.61	1.74E-12	Male
DALYs	High-middle SDI	Smoking	-1.13	-0.91	-0.69	4.20E-09	Male
DALYs	Low SDI	Smoking	-0.97	-0.88	-0.79	1.23E-17	Male
DALYs	Western Europe	Smoking	-3.44	-3.17	-2.91	3.22E-20	Male
DALYs	Central Europe	Smoking	-0.04	0.33	0.69	0.07538002	Male
DALYs	High-income Asia Pacific	Smoking	0.02	0.21	0.39	0.02886277	Male
DALYs	Central Sub-Saharan Africa	Smoking	-2.01	-1.78	-1.55	2.11E-15	Male
DALYs	Middle SDI	Smoking	-0.46	-0.36	-0.26	2.09E-08	Male
DALYs	East Asia	Smoking	-0.03	0.03	0.09	0.324524413	Male
DALYs	Low-middle SDI	Smoking	-0.09	-0.02	0.04	0.473301371	Male
DALYs	Central Latin America	Smoking	-2.19	-2	-1.81	8.44E-19	Male
DALYs	Eastern Sub-Saharan Africa	Smoking	-2.08	-1.92	-1.77	6.57E-21	Male
DALYs	World Bank Low Income	Smoking	-1.37	-1.24	-1.12	1.17E-18	Male
DALYs	High SDI	Smoking	-3.13	-2.99	-2.85	5.70E-27	Male
DALYs	South Asia	Smoking	-0.42	-0.33	-0.25	1.60E-08	Male
DALYs	Andean Latin America	Smoking	-1.24	-1.09	-0.94	9.58E-15	Male
DALYs	Western Sub-Saharan Africa	Smoking	-0.39	-0.29	-0.2	9.02E-07	Male
DALYs	World Bank High Income	Smoking	-2.84	-2.68	-2.51	5.82E-24	Male
DALYs	World Bank Lower Middle Income	Smoking	-0.13	-0.06	0	0.04196413	Male
DALYs	Caribbean	Smoking	-1.15	-0.98	-0.8	6.10E-12	Male
DALYs	Southern Sub-Saharan Africa	Smoking	-3.49	-2.86	-2.23	5.72E-10	Male
DALYs	Tropical Latin America	Smoking	-1.2	-0.9	-0.59	1.67E-06	Male
DALYs	World Bank Upper Middle Income	Smoking	-0.83	-0.7	-0.57	9.43E-12	Male
DALYs	Oceania	High systolic blood pressure	-0.12	-0.02	0.09	0.744948066	Male
DALYs	Southeast Asia	High systolic blood pressure	1.18	1.24	1.29	6.17E-28	Male
DALYs	Global	High systolic blood pressure	-1.56	-1.45	-1.34	3.17E-21	Male
DALYs	High-income North America	High systolic blood pressure	-5.13	-4.67	-4.22	2.20E-18	Male
DALYs	Central Asia	High systolic blood pressure	1.65	1.8	1.95	1.06E-20	Male
DALYs	East Asia	High systolic blood pressure	0.92	1.03	1.13	3.51E-18	Male
DALYs	North Africa and Middle East	High systolic blood pressure	-0.9	-0.77	-0.64	1.56E-12	Male
DALYs	Australasia	High systolic blood pressure	-5.66	-5.26	-4.86	2.70E-21	Male
DALYs	Southern Latin America	High systolic blood pressure	-0.86	-0.49	-0.12	0.011775269	Male
DALYs	High-middle SDI	High systolic blood pressure	-0.75	-0.53	-0.31	4.39E-05	Male
DALYs	Low SDI	High systolic blood pressure	-0.01	0.04	0.1	0.122699648	Male
DALYs	Eastern Europe	High systolic blood pressure	0.9	1.17	1.45	1.37E-09	Male
DALYs	Central Europe	High systolic blood pressure	0.29	0.62	0.95	0.000586218	Male
DALYs	High-income Asia Pacific	High systolic blood pressure	-0.17	-0.06	0.05	0.282218195	Male
DALYs	Middle SDI	High systolic blood pressure	0.43	0.52	0.61	4.24E-12	Male
DALYs	Low-middle SDI	High systolic blood pressure	0.79	0.85	0.91	5.78E-22	Male
DALYs	Central Latin America	High systolic blood pressure	-0.67	-0.45	-0.24	0.000176596	Male
DALYs	Eastern Sub-Saharan Africa	High systolic blood pressure	-0.34	-0.23	-0.12	0.000203008	Male
DALYs	Andean Latin America	High systolic blood pressure	0.75	1.01	1.28	1.10E-08	Male
DALYs	World Bank Low Income	High systolic blood pressure	-0.42	-0.33	-0.25	1.23E-08	Male
DALYs	High SDI	High systolic blood pressure	-3.49	-3.26	-3.02	4.86E-22	Male
DALYs	South Asia	High systolic blood pressure	0.48	0.57	0.67	1.19E-12	Male

DALYs	Western Europe	High systolic blood pressure	-3.67	-3.37	-3.06	2.39E-19	Male
DALYs	Western Sub-Saharan Africa	High systolic blood pressure	0.79	0.88	0.96	4.67E-19	Male
DALYs	World Bank High Income	High systolic blood pressure	-3.08	-2.87	-2.67	4.04E-22	Male
DALYs	World Bank Lower Middle Income	High systolic blood pressure	0.61	0.67	0.73	1.70E-20	Male
DALYs	Southern Sub-Saharan Africa	High systolic blood pressure	-2.34	-1.71	-1.08	6.40E-06	Male
DALYs	Tropical Latin America	High systolic blood pressure	-0.07	0.22	0.51	0.126340966	Male
DALYs	Central Sub-Saharan Africa	High systolic blood pressure	-1.45	-1.29	-1.13	5.63E-16	Male
DALYs	Caribbean	High systolic blood pressure	-0.58	-0.41	-0.25	2.38E-05	Male
DALYs	World Bank Upper Middle Income	High systolic blood pressure	-0.12	0.01	0.14	0.902450601	Male
Deaths	Oceania	High systolic blood pressure	-0.27	-0.2	-0.13	3.74E-06	Male
Deaths	Tropical Latin America	High systolic blood pressure	0.15	0.45	0.75	0.00445244	Male
Deaths	Global	High systolic blood pressure	-1.79	-1.66	-1.52	5.86E-21	Male
Deaths	North Africa and Middle East	High systolic blood pressure	-0.59	-0.46	-0.34	3.72E-08	Male
Deaths	Western Europe	High systolic blood pressure	-3.74	-3.42	-3.09	1.05E-18	Male
Deaths	South Asia	High systolic blood pressure	0.47	0.57	0.66	1.78E-12	Male
Deaths	Southern Latin America	High systolic blood pressure	-0.83	-0.43	-0.03	0.038050213	Male
Deaths	High-income North America	High systolic blood pressure	-5.56	-5.12	-4.68	7.80E-20	Male
Deaths	Southeast Asia	High systolic blood pressure	1.25	1.3	1.35	1.78E-30	Male
Deaths	Middle SDI	High systolic blood pressure	0.44	0.53	0.62	2.29E-12	Male
Deaths	High-middle SDI	High systolic blood pressure	-0.77	-0.53	-0.28	0.000128497	Male
Deaths	Eastern Europe	High systolic blood pressure	0.89	1.13	1.38	3.23E-10	Male
Deaths	Australasia	High systolic blood pressure	-5.59	-5.21	-4.83	1.23E-21	Male
Deaths	East Asia	High systolic blood pressure	0.88	0.97	1.07	1.54E-18	Male
Deaths	Low-middle SDI	High systolic blood pressure	0.78	0.84	0.9	1.35E-21	Male
Deaths	High SDI	High systolic blood pressure	-3.64	-3.4	-3.16	4.65E-22	Male
Deaths	Andean Latin America	High systolic blood pressure	1.04	1.3	1.56	4.94E-11	Male
Deaths	Low SDI	High systolic blood pressure	0.07	0.11	0.16	4.68E-06	Male
Deaths	Caribbean	High systolic blood pressure	-0.7	-0.52	-0.35	1.16E-06	Male
Deaths	High-income Asia Pacific	High systolic blood pressure	-0.35	-0.22	-0.09	0.001836081	Male
Deaths	Central Asia	High systolic blood pressure	2.11	2.25	2.38	2.01E-24	Male
Deaths	Southern Sub-Saharan Africa	High systolic blood pressure	-2.26	-1.63	-1.01	1.12E-05	Male
Deaths	World Bank Lower Middle Income	High systolic blood pressure	0.64	0.7	0.76	1.20E-20	Male
Deaths	World Bank High Income	High systolic blood pressure	-3.25	-3.03	-2.8	1.24E-21	Male
Deaths	World Bank Upper Middle Income	High systolic blood pressure	-0.03	0.09	0.21	0.150056019	Male
Deaths	Central Sub-Saharan Africa	High systolic blood pressure	-1.38	-1.23	-1.07	6.87E-16	Male
Deaths	Central Latin America	High systolic blood pressure	-0.51	-0.28	-0.05	0.018007453	Male
Deaths	Central Europe	High systolic blood pressure	0.48	0.83	1.18	3.59E-05	Male
Deaths	World Bank Low Income	High systolic blood pressure	-0.29	-0.21	-0.14	3.44E-06	Male
Deaths	Eastern Sub-Saharan Africa	High systolic blood pressure	-0.23	-0.13	-0.02	0.018195011	Male
Deaths	Western Sub-Saharan Africa	High systolic blood pressure	0.91	1	1.09	5.79E-20	Male
Deaths	Oceania	Diet high in sodium	-0.93	-0.82	-0.71	5.76E-15	Male
Deaths	Tropical Latin America	Diet high in sodium	-0.16	0.13	0.41	0.366298621	Male
Deaths	Global	Diet high in sodium	-0.93	-0.85	-0.76	4.30E-18	Male
Deaths	North Africa and Middle East	Diet high in sodium	-0.62	-0.51	-0.4	3.30E-10	Male
Deaths	South Asia	Diet high in sodium	0.71	0.8	0.89	1.03E-16	Male
Deaths	Southern Latin America	Diet high in sodium	-1.78	-1.42	-1.06	1.01E-08	Male
Deaths	High-income North America	Diet high in sodium	-2.48	-2.17	-1.87	1.57E-14	Male
Deaths	Southeast Asia	Diet high in sodium	-0.12	-0.05	0.03	0.194902318	Male
Deaths	Middle SDI	Diet high in sodium	-0.18	-0.14	-0.11	3.55E-09	Male
Deaths	High-middle SDI	Diet high in sodium	-0.46	-0.34	-0.23	1.23E-06	Male
Deaths	Australasia	Diet high in sodium	-4.65	-4.34	-4.03	6.27E-22	Male
Deaths	East Asia	Diet high in sodium	-0.26	-0.18	-0.1	7.83E-05	Male
Deaths	Low-middle SDI	Diet high in sodium	0.52	0.57	0.62	7.87E-21	Male
Deaths	High SDI	Diet high in sodium	-2.13	-1.95	-1.77	4.31E-19	Male
Deaths	Western Europe	Diet high in sodium	-2.7	-2.47	-2.24	4.08E-19	Male
Deaths	Andean Latin America	Diet high in sodium	-0.15	-0.02	0.11	0.763299638	Male
Deaths	Low SDI	Diet high in sodium	-1.3	-1.18	-1.06	4.97E-18	Male
Deaths	Eastern Europe	Diet high in sodium	0.86	1.14	1.41	2.93E-09	Male
Deaths	Caribbean	Diet high in sodium	-1.09	-0.91	-0.72	1.58E-10	Male
Deaths	Central Asia	Diet high in sodium	0.56	0.71	0.86	1.12E-10	Male
Deaths	High-income Asia Pacific	Diet high in sodium	-1.74	-1.6	-1.45	1.31E-19	Male
Deaths	Southern Sub-Saharan Africa	Diet high in sodium	-2.8	-2.28	-1.74	1.92E-09	Male
Deaths	World Bank Lower Middle Income	Diet high in sodium	0.33	0.38	0.42	6.95E-17	Male
Deaths	World Bank High Income	Diet high in sodium	-1.82	-1.64	-1.46	2.54E-17	Male
Deaths	World Bank Upper Middle Income	Diet high in sodium	-0.4	-0.34	-0.29	8.10E-13	Male
Deaths	Central Latin America	Diet high in sodium	-1.01	-0.71	-0.41	4.82E-05	Male
Deaths	Central Europe	Diet high in sodium	0.33	0.49	0.65	9.52E-07	Male
Deaths	Central Sub-Saharan Africa	Diet high in sodium	-1.03	-0.86	-0.69	5.04E-11	Male
Deaths	World Bank Low Income	Diet high in sodium	-1.86	-1.74	-1.62	1.98E-22	Male
Deaths	Eastern Sub-Saharan Africa	Diet high in sodium	-2.85	-2.67	-2.49	8.03E-23	Male
Deaths	Western Sub-Saharan Africa	Diet high in sodium	0.19	0.26	0.33	1.29E-08	Male
Deaths	Oceania	Lead exposure	-0.74	-0.65	-0.55	5.66E-14	Male
Deaths	Tropical Latin America	Lead exposure	-0.8	-0.46	-0.11	0.012466476	Male
Deaths	Global	Lead exposure	-1.35	-1.19	-1.03	2.82E-15	Male
Deaths	North Africa and Middle East	Lead exposure	-0.77	-0.64	-0.51	1.04E-10	Male
Deaths	Western Europe	Lead exposure	-3.61	-3.17	-2.72	1.85E-14	Male
Deaths	South Asia	Lead exposure	0.01	0.16	0.31	0.040939567	Male
Deaths	Southern Latin America	Lead exposure	-1.99	-1.48	-0.98	1.97E-06	Male
Deaths	High-income North America	Lead exposure	-6.05	-5.75	-5.43	2.83E-25	Male
Deaths	Southeast Asia	Lead exposure	0.82	0.98	1.15	7.52E-13	Male
Deaths	Andean Latin America	Lead exposure	-0.09	0.04	0.18	0.5252992	Male
Deaths	Central Asia	Lead exposure	1.97	2.2	2.43	3.36E-18	Male
Deaths	Middle SDI	Lead exposure	-0.29	-0.11	0.07	0.202718778	Male
Deaths	High-middle SDI	Lead exposure	-1.02	-0.65	-0.28	0.001282097	Male
Deaths	Australasia	Lead exposure	-5.14	-4.84	-4.54	1.12E-23	Male
Deaths	East Asia	Lead exposure	-0.33	-0.16	0.01	0.065223856	Male
Deaths	High-income Asia Pacific	Lead exposure	-1.11	-0.86	-0.62	9.52E-08	Male
Deaths	Low-middle SDI	Lead exposure	0.23	0.34	0.46	1.45E-06	Male
Deaths	High SDI	Lead exposure	-4.38	-4.13	-3.88	5.75E-24	Male
Deaths	Low SDI	Lead exposure	-0.32	-0.26	-0.19	1.82E-08	Male
Deaths	Eastern Europe	Lead exposure	0.94	1.22	1.49	6.74E-10	Male
Deaths	Caribbean	Lead exposure	-0.81	-0.6	-0.4	2.04E-06	Male
Deaths	Central Europe	Lead exposure	0.29	0.84	1.39	0.003988311	Male
Deaths	Central Latin America	Lead exposure	-1.46	-1.17	-0.88	6.50E-09	Male
Deaths	World Bank Upper Middle Income	Lead exposure	-0.55	-0.34	-0.13	0.002591981	Male
Deaths	Southern Sub-Saharan Africa	Lead exposure	-2.13	-1.49	-0.84	6.75E-05	Male
Deaths	World Bank Lower Middle Income	Lead exposure	0.31	0.44	0.56	1.04E-07	Male
Deaths	World Bank High Income	Lead exposure	-3.82	-3.53	-3.23	3.20E-20	Male
Deaths	Central Sub-Saharan Africa	Lead exposure	-0.89	-0.76	-0.63	1.39E-12	Male
Deaths	Western Sub-Saharan Africa	Lead exposure	0.17	0.3	0.43	4.47E-05	Male
Deaths	World Bank Low Income	Lead exposure	-1	-0.9	-0.81	1.40E-17	Male
Deaths	Eastern Sub-Saharan Africa	Lead exposure	-2.06	-1.92	-1.78	9.68E-22	Male

Deaths	Oceania	Smoking	-0.98	-0.92	-0.87	5.80E-25	Male
Deaths	Tropical Latin America	Smoking	-1.1	-0.78	-0.46	3.04E-05	Male
Deaths	Global	Smoking	-2.11	-1.98	-1.85	4.23E-23	Male
Deaths	North Africa and Middle East	Smoking	-1.26	-1.12	-0.98	7.32E-16	Male
Deaths	Western Europe	Smoking	-3.66	-3.36	-3.06	1.76E-19	Male
Deaths	South Asia	Smoking	-0.52	-0.43	-0.34	2.43E-10	Male
Deaths	Southern Latin America	Smoking	-2.37	-2.02	-1.68	1.88E-12	Male
Deaths	High-income North America	Smoking	-5.03	-4.72	-4.41	4.11E-23	Male
Deaths	Southeast Asia	Smoking	0.47	0.53	0.59	1.11E-16	Male
Deaths	Andean Latin America	Smoking	-1.18	-1.03	-0.88	3.99E-14	Male
Deaths	Middle SDI	Smoking	-0.52	-0.41	-0.3	3.89E-08	Male
Deaths	High-middle SDI	Smoking	-1.16	-0.92	-0.68	2.09E-08	Male
Deaths	Australasia	Smoking	-6.13	-5.8	-5.46	1.84E-24	Male
Deaths	East Asia	Smoking	-0.16	-0.07	0.02	0.126276775	Male
Deaths	Low-middle SDI	Smoking	-0.15	-0.08	-0.01	0.01913069	Male
Deaths	High SDI	Smoking	-3.51	-3.34	-3.17	4.76E-26	Male
Deaths	Low SDI	Smoking	-0.95	-0.86	-0.77	4.06E-18	Male
Deaths	Eastern Europe	Smoking	0.9	1.16	1.42	5.55E-10	Male
Deaths	Caribbean	Smoking	-1.26	-1.08	-0.91	5.66E-13	Male
Deaths	High-income Asia Pacific	Smoking	-0.44	-0.24	-0.05	0.017510913	Male
Deaths	Central Europe	Smoking	0.14	0.52	0.91	0.009924325	Male
Deaths	World Bank Upper Middle Income	Smoking	-0.81	-0.68	-0.55	2.33E-11	Male
Deaths	Central Asia	Smoking	1.68	1.83	1.97	2.64E-21	Male
Deaths	Southern Sub-Saharan Africa	Smoking	-3.66	-3.02	-2.37	3.43E-10	Male
Deaths	World Bank Lower Middle Income	Smoking	-0.16	-0.09	-0.03	0.006100997	Male
Deaths	World Bank High Income	Smoking	-3.18	-2.98	-2.79	2.97E-23	Male
Deaths	Central Sub-Saharan Africa	Smoking	-2	-1.77	-1.54	2.23E-15	Male
Deaths	Central Latin America	Smoking	-2.09	-1.9	-1.72	1.48E-18	Male
Deaths	Western Sub-Saharan Africa	Smoking	-0.34	-0.26	-0.18	4.45E-07	Male
Deaths	World Bank Low Income	Smoking	-1.32	-1.2	-1.08	1.52E-18	Male
Deaths	Eastern Sub-Saharan Africa	Smoking	-2.08	-1.93	-1.78	6.69E-21	Male
