# Supplementary material for: Burden of Aortic Aneurysm and Its Attributable Risk Factors from 1990 to 2019: An Analysis of the Global Burden of Disease Study 2019
Source: Front Cardiovasc Med. 2022 May 31;9:901225. doi: 10.3389/fcvm.2022.901225 (PMC9197430; doi:10.3389/fcvm.2022.901225)
Supplement: Supplementary Table 9 — Population attributable fractions of aortic aneurism-related attributable risks in 31 GBD regions in 2019. DALY, disability-adjusted life year rate. SDI, socio-demographic index; PAF, population attributable fractions; GBD, Global Burden of Disease. [file Data_Sheet_9.PDF]

measure	location	sex	age	cause	rei	metric	year	val	PAF(2019)
DALYs	Andean Latin America	Both	Age-standardized	Aortic aneurysm	Diet high in sodium	Rate	2019	1.188319475	4.26%
DALYs	Andean Latin America	Both	Age-standardized	Aortic aneurysm	High systolic blood pressure	Rate	2019	7.749601555	27.81%
DALYs	Andean Latin America	Both	Age-standardized	Aortic aneurysm	Lead exposure	Rate	2019	0.576861038	2.07%
DALYs	Andean Latin America	Both	Age-standardized	Aortic aneurysm	Smoking	Rate	2019	5.955171987	21.37%
Deaths	Andean Latin America	Both	Age-standardized	Aortic aneurysm	Diet high in sodium	Rate	2019	0.05861157	4.07%
Deaths	Andean Latin America	Both	Age-standardized	Aortic aneurysm	High systolic blood pressure	Rate	2019	0.388807112	26.98%
Deaths	Andean Latin America	Both	Age-standardized	Aortic aneurysm	Lead exposure	Rate	2019	0.030362702	2.11%
Deaths	Andean Latin America	Both	Age-standardized	Aortic aneurysm	Smoking	Rate	2019	0.248321167	17.23%
DALYs	Australasia	Both	Age-standardized	Aortic aneurysm	Diet high in sodium	Rate	2019	1.130116141	2.11%
DALYs	Australasia	Both	Age-standardized	Aortic aneurysm	High systolic blood pressure	Rate	2019	18.34965387	34.30%
DALYs	Australasia	Both	Age-standardized	Aortic aneurysm	Lead exposure	Rate	2019	0.896368933	1.68%
DALYs	Australasia	Both	Age-standardized	Aortic aneurysm	Smoking	Rate	2019	18.92098692	35.37%
Deaths	Australasia	Both	Age-standardized	Aortic aneurysm	Diet high in sodium	Rate	2019	0.056298967	1.70%
Deaths	Australasia	Both	Age-standardized	Aortic aneurysm	High systolic blood pressure	Rate	2019	1.040938471	31.42%
Deaths	Australasia	Both	Age-standardized	Aortic aneurysm	Lead exposure	Rate	2019	0.056164986	1.70%
Deaths	Australasia	Both	Age-standardized	Aortic aneurysm	Smoking	Rate	2019	0.849817551	25.65%
DALYs	Caribbean	Both	Age-standardized	Aortic aneurysm	Diet high in sodium	Rate	2019	1.664000086	2.90%
DALYs	Caribbean	Both	Age-standardized	Aortic aneurysm	High systolic blood pressure	Rate	2019	20.21236127	35.28%
DALYs	Caribbean	Both	Age-standardized	Aortic aneurysm	Lead exposure	Rate	2019	1.836106597	3.21%
DALYs	Caribbean	Both	Age-standardized	Aortic aneurysm	Smoking	Rate	2019	22.58583215	39.43%
Deaths	Caribbean	Both	Age-standardized	Aortic aneurysm	Diet high in sodium	Rate	2019	0.086215987	2.83%
Deaths	Caribbean	Both	Age-standardized	Aortic aneurysm	High systolic blood pressure	Rate	2019	0.986922889	32.42%
Deaths	Caribbean	Both	Age-standardized	Aortic aneurysm	Lead exposure	Rate	2019	0.096259428	3.16%
Deaths	Caribbean	Both	Age-standardized	Aortic aneurysm	Smoking	Rate	2019	1.01650201	33.39%
DALYs	Central Asia	Both	Age-standardized	Aortic aneurysm	Diet high in sodium	Rate	2019	2.11173514	4.53%
DALYs	Central Asia	Both	Age-standardized	Aortic aneurysm	High systolic blood pressure	Rate	2019	19.87999446	42.67%
DALYs	Central Asia	Both	Age-standardized	Aortic aneurysm	Lead exposure	Rate	2019	0.691398094	1.48%
DALYs	Central Asia	Both	Age-standardized	Aortic aneurysm	Smoking	Rate	2019	19.62455562	42.12%
Deaths	Central Asia	Both	Age-standardized	Aortic aneurysm	Diet high in sodium	Rate	2019	0.103292158	4.44%
Deaths	Central Asia	Both	Age-standardized	Aortic aneurysm	High systolic blood pressure	Rate	2019	0.91735118	39.42%
Deaths	Central Asia	Both	Age-standardized	Aortic aneurysm	Lead exposure	Rate	2019	0.036689046	1.58%
Deaths	Central Asia	Both	Age-standardized	Aortic aneurysm	Smoking	Rate	2019	0.788015722	33.86%
DALYs	Central Europe	Both	Age-standardized	Aortic aneurysm	Diet high in sodium	Rate	2019	7.169423194	10.93%
DALYs	Central Europe	Both	Age-standardized	Aortic aneurysm	High systolic blood pressure	Rate	2019	28.19368504	43.00%
DALYs	Central Europe	Both	Age-standardized	Aortic aneurysm	Lead exposure	Rate	2019	0.833613716	1.27%
DALYs	Central Europe	Both	Age-standardized	Aortic aneurysm	Smoking	Rate	2019	36.2381514	55.27%
Deaths	Central Europe	Both	Age-standardized	Aortic aneurysm	Diet high in sodium	Rate	2019	0.325489376	10.82%
Deaths	Central Europe	Both	Age-standardized	Aortic aneurysm	High systolic blood pressure	Rate	2019	1.212506965	40.31%
Deaths	Central Europe	Both	Age-standardized	Aortic aneurysm	Lead exposure	Rate	2019	0.03995854	1.33%
Deaths	Central Europe	Both	Age-standardized	Aortic aneurysm	Smoking	Rate	2019	1.399086746	46.51%
DALYs	Central Latin America	Both	Age-standardized	Aortic aneurysm	Diet high in sodium	Rate	2019	2.210632061	6.87%
DALYs	Central Latin America	Both	Age-standardized	Aortic aneurysm	High systolic blood pressure	Rate	2019	11.69759057	36.34%
DALYs	Central Latin America	Both	Age-standardized	Aortic aneurysm	Lead exposure	Rate	2019	0.9606951	2.98%
DALYs	Central Latin America	Both	Age-standardized	Aortic aneurysm	Smoking	Rate	2019	9.505432204	29.53%
Deaths	Central Latin America	Both	Age-standardized	Aortic aneurysm	Diet high in sodium	Rate	2019	0.106439735	6.45%
Deaths	Central Latin America	Both	Age-standardized	Aortic aneurysm	High systolic blood pressure	Rate	2019	0.563927203	34.19%
Deaths	Central Latin America	Both	Age-standardized	Aortic aneurysm	Lead exposure	Rate	2019	0.052462873	3.18%
Deaths	Central Latin America	Both	Age-standardized	Aortic aneurysm	Smoking	Rate	2019	0.399292104	24.21%
DALYs	Central Sub-Saharan Africa	Both	Age-standardized	Aortic aneurysm	Diet high in sodium	Rate	2019	1.154076472	2.07%
DALYs	Central Sub-Saharan Africa	Both	Age-standardized	Aortic aneurysm	High systolic blood pressure	Rate	2019	22.38606857	40.17%
DALYs	Central Sub-Saharan Africa	Both	Age-standardized	Aortic aneurysm	Lead exposure	Rate	2019	1.423241059	2.55%
DALYs	Central Sub-Saharan Africa	Both	Age-standardized	Aortic aneurysm	Smoking	Rate	2019	11.85991899	21.28%
Deaths	Central Sub-Saharan Africa	Both	Age-standardized	Aortic aneurysm	Diet high in sodium	Rate	2019	0.054310641	1.99%
Deaths	Central Sub-Saharan Africa	Both	Age-standardized	Aortic aneurysm	High systolic blood pressure	Rate	2019	1.035216002	37.98%
Deaths	Central Sub-Saharan Africa	Both	Age-standardized	Aortic aneurysm	Lead exposure	Rate	2019	0.06829907	2.51%
Deaths	Central Sub-Saharan Africa	Both	Age-standardized	Aortic aneurysm	Smoking	Rate	2019	0.451684493	16.57%
DALYs	East Asia	Both	Age-standardized	Aortic aneurysm	Diet high in sodium	Rate	2019	2.915563911	14.29%
DALYs	East Asia	Both	Age-standardized	Aortic aneurysm	High systolic blood pressure	Rate	2019	7.315343355	35.86%
DALYs	East Asia	Both	Age-standardized	Aortic aneurysm	Lead exposure	Rate	2019	0.656019349	3.22%
DALYs	East Asia	Both	Age-standardized	Aortic aneurysm	Smoking	Rate	2019	9.888407959	48.48%
Deaths	East Asia	Both	Age-standardized	Aortic aneurysm	Diet high in sodium	Rate	2019	0.115726764	11.84%
Deaths	East Asia	Both	Age-standardized	Aortic aneurysm	High systolic blood pressure	Rate	2019	0.331392244	33.89%
Deaths	East Asia	Both	Age-standardized	Aortic aneurysm	Lead exposure	Rate	2019	0.032657641	3.34%
Deaths	East Asia	Both	Age-standardized	Aortic aneurysm	Smoking	Rate	2019	0.399157151	40.82%
DALYs	Eastern Europe	Both	Age-standardized	Aortic aneurysm	Diet high in sodium	Rate	2019	4.37579519	5.28%
DALYs	Eastern Europe	Both	Age-standardized	Aortic aneurysm	High systolic blood pressure	Rate	2019	37.20978927	44.92%
DALYs	Eastern Europe	Both	Age-standardized	Aortic aneurysm	Lead exposure	Rate	2019	0.430672535	0.52%
DALYs	Eastern Europe	Both	Age-standardized	Aortic aneurysm	Smoking	Rate	2019	46.77966984	56.47%
Deaths	Eastern Europe	Both	Age-standardized	Aortic aneurysm	Diet high in sodium	Rate	2019	0.16177141	4.63%
Deaths	Eastern Europe	Both	Age-standardized	Aortic aneurysm	High systolic blood pressure	Rate	2019	1.482290381	42.40%
Deaths	Eastern Europe	Both	Age-standardized	Aortic aneurysm	Lead exposure	Rate	2019	0.018337707	0.52%
Deaths	Eastern Europe	Both	Age-standardized	Aortic aneurysm	Smoking	Rate	2019	1.65932346	47.46%
DALYs	Eastern Sub-Saharan Africa	Both	Age-standardized	Aortic aneurysm	Diet high in sodium	Rate	2019	2.63927629	5.83%
DALYs	Eastern Sub-Saharan Africa	Both	Age-standardized	Aortic aneurysm	High systolic blood pressure	Rate	2019	17.43597401	38.52%
DALYs	Eastern Sub-Saharan Africa	Both	Age-standardized	Aortic aneurysm	Lead exposure	Rate	2019	1.224235721	2.70%
DALYs	Eastern Sub-Saharan Africa	Both	Age-standardized	Aortic aneurysm	Smoking	Rate	2019	9.987196828	22.06%
Deaths	Eastern Sub-Saharan Africa	Both	Age-standardized	Aortic aneurysm	Diet high in sodium	Rate	2019	0.133877428	5.89%
Deaths	Eastern Sub-Saharan Africa	Both	Age-standardized	Aortic aneurysm	High systolic blood pressure	Rate	2019	0.812480595	35.74%
Deaths	Eastern Sub-Saharan Africa	Both	Age-standardized	Aortic aneurysm	Lead exposure	Rate	2019	0.06414136	2.82%
Deaths	Eastern Sub-Saharan Africa	Both	Age-standardized	Aortic aneurysm	Smoking	Rate	2019	0.404965801	17.81%
DALYs	Global	Both	Age-standardized	Aortic aneurysm	Diet high in sodium	Rate	2019	2.466228562	6.02%
DALYs	Global	Both	Age-standardized	Aortic aneurysm	High systolic blood pressure	Rate	2019	15.11957249	36.93%
DALYs	Global	Both	Age-standardized	Aortic aneurysm	Lead exposure	Rate	2019	0.85238563	2.08%
DALYs	Global	Both	Age-standardized	Aortic aneurysm	Smoking	Rate	2019	16.91938138	41.33%
Deaths	Global	Both	Age-standardized	Aortic aneurysm	Diet high in sodium	Rate	2019	0.113787487	5.14%
Deaths	Global	Both	Age-standardized	Aortic aneurysm	High systolic blood pressure	Rate	2019	0.755247694	34.11%
Deaths	Global	Both	Age-standardized	Aortic aneurysm	Lead exposure	Rate	2019	0.043596207	1.97%
Deaths	Global	Both	Age-standardized	Aortic aneurysm	Smoking	Rate	2019	0.731045968	33.02%
DALYs	High SDI	Both	Age-standardized	Aortic aneurysm	Diet high in sodium	Rate	2019	2.522266748	4.60%
DALYs	High SDI	Both	Age-standardized	Aortic aneurysm	High systolic blood pressure	Rate	2019	18.95719972	34.61%
DALYs	High SDI	Both	Age-standardized	Aortic aneurysm	Lead exposure	Rate	2019	0.436482875	0.80%
DALYs	High SDI	Both	Age-standardized	Aortic aneurysm	Smoking	Rate	2019	24.73515586	45.16%
Deaths	High SDI	Both	Age-standardized	Aortic aneurysm	Diet high in sodium	Rate	2019	0.12720153	4.07%
Deaths	High SDI	Both	Age-standardized	Aortic aneurysm	High systolic blood pressure	Rate	2019	1.006291375	32.19%
Deaths	High SDI	Both	Age-standardized	Aortic aneurysm	Lead exposure	Rate	2019	0.026406575	0.84%
Deaths	High SDI	Both	Age-standardized	Aortic aneurysm	Smoking	Rate	2019	1.089688118	34.85%
DALYs	High-income Asia Pacific	Both	Age-standardized	Aortic aneurysm	Diet high in sodium	Rate	2019	4.296385567	6.56%
DALYs	High-income Asia Pacific	Both	Age-standardized	Aortic aneurysm	High systolic blood pressure	Rate	2019	23.10559247	35.26%
DALYs	High-income Asia Pacific	Both	Age-standardized	Aortic aneurysm	Lead exposure	Rate	2019	0.326546953	0.50%
DALYs	High-income Asia Pacific	Both	Age-standardized	Aortic aneurysm	Smoking	Rate	2019	27.50378486	41.97%
Deaths	High-income Asia Pacific	Both	Age-standardized	Aortic aneurysm	Diet high in sodium	Rate	2019	0.229677661	5.83%

Deaths	High-income Asia Pacific	Both	Age-standardized	Aortic aneurysm	High systolic blood pressure	Rate	2019	1.266360205	32.13%
Deaths	High-income Asia Pacific	Both	Age-standardized	Aortic aneurysm	Lead exposure	Rate	2019	0.021791966	0.55%
Deaths	High-income Asia Pacific	Both	Age-standardized	Aortic aneurysm	Smoking	Rate	2019	1.203047158	30.52%
DALYs	High-income North America	Both	Age-standardized	Aortic aneurysm	Diet high in sodium	Rate	2019	1.863477613	3.96%
DALYs	High-income North America	Both	Age-standardized	Aortic aneurysm	High systolic blood pressure	Rate	2019	14.29046308	30.35%
DALYs	High-income North America	Both	Age-standardized	Aortic aneurysm	Lead exposure	Rate	2019	0.413581663	0.88%
DALYs	High-income North America	Both	Age-standardized	Aortic aneurysm	Smoking	Rate	2019	23.0123968	48.87%
Deaths	High-income North America	Both	Age-standardized	Aortic aneurysm	Diet high in sodium	Rate	2019	0.081366751	3.33%
Deaths	High-income North America	Both	Age-standardized	Aortic aneurysm	High systolic blood pressure	Rate	2019	0.709685814	29.06%
Deaths	High-income North America	Both	Age-standardized	Aortic aneurysm	Lead exposure	Rate	2019	0.024239783	0.99%
Deaths	High-income North America	Both	Age-standardized	Aortic aneurysm	Smoking	Rate	2019	0.955007562	39.11%
DALYs	High-middle SDI	Both	Age-standardized	Aortic aneurysm	Diet high in sodium	Rate	2019	3.295040904	7.06%
DALYs	High-middle SDI	Both	Age-standardized	Aortic aneurysm	High systolic blood pressure	Rate	2019	18.79365798	40.27%
DALYs	High-middle SDI	Both	Age-standardized	Aortic aneurysm	Lead exposure	Rate	2019	0.659896053	1.41%
DALYs	High-middle SDI	Both	Age-standardized	Aortic aneurysm	Smoking	Rate	2019	23.40932815	50.16%
Deaths	High-middle SDI	Both	Age-standardized	Aortic aneurysm	Diet high in sodium	Rate	2019	0.138604843	6.17%
Deaths	High-middle SDI	Both	Age-standardized	Aortic aneurysm	High systolic blood pressure	Rate	2019	0.84383302	37.58%
Deaths	High-middle SDI	Both	Age-standardized	Aortic aneurysm	Lead exposure	Rate	2019	0.033627524	1.50%
Deaths	High-middle SDI	Both	Age-standardized	Aortic aneurysm	Smoking	Rate	2019	0.928538849	41.35%
DALYs	Low SDI	Both	Age-standardized	Aortic aneurysm	Diet high in sodium	Rate	2019	1.594922079	4.26%
DALYs	Low SDI	Both	Age-standardized	Aortic aneurysm	High systolic blood pressure	Rate	2019	13.97932423	37.31%
DALYs	Low SDI	Both	Age-standardized	Aortic aneurysm	Lead exposure	Rate	2019	1.596052927	4.26%
DALYs	Low SDI	Both	Age-standardized	Aortic aneurysm	Smoking	Rate	2019	9.761962478	26.05%
Deaths	Low SDI	Both	Age-standardized	Aortic aneurysm	Diet high in sodium	Rate	2019	0.07612433	4.06%
Deaths	Low SDI	Both	Age-standardized	Aortic aneurysm	High systolic blood pressure	Rate	2019	0.649237456	34.67%
Deaths	Low SDI	Both	Age-standardized	Aortic aneurysm	Lead exposure	Rate	2019	0.080398588	4.29%
Deaths	Low SDI	Both	Age-standardized	Aortic aneurysm	Smoking	Rate	2019	0.401853311	21.46%
DALYs	Low-middle SDI	Both	Age-standardized	Aortic aneurysm	Diet high in sodium	Rate	2019	1.796369934	5.24%
DALYs	Low-middle SDI	Both	Age-standardized	Aortic aneurysm	High systolic blood pressure	Rate	2019	12.57870374	36.67%
DALYs	Low-middle SDI	Both	Age-standardized	Aortic aneurysm	Lead exposure	Rate	2019	1.511242974	4.41%
DALYs	Low-middle SDI	Both	Age-standardized	Aortic aneurysm	Smoking	Rate	2019	11.91270522	34.73%
Deaths	Low-middle SDI	Both	Age-standardized	Aortic aneurysm	Diet high in sodium	Rate	2019	0.078370784	4.57%
Deaths	Low-middle SDI	Both	Age-standardized	Aortic aneurysm	High systolic blood pressure	Rate	2019	0.591242617	34.50%
Deaths	Low-middle SDI	Both	Age-standardized	Aortic aneurysm	Lead exposure	Rate	2019	0.07637155	4.46%
Deaths	Low-middle SDI	Both	Age-standardized	Aortic aneurysm	Smoking	Rate	2019	0.496927036	28.99%
DALYs	Middle SDI	Both	Age-standardized	Aortic aneurysm	Diet high in sodium	Rate	2019	2.25621096	7.91%
DALYs	Middle SDI	Both	Age-standardized	Aortic aneurysm	High systolic blood pressure	Rate	2019	10.51506996	36.86%
DALYs	Middle SDI	Both	Age-standardized	Aortic aneurysm	Lead exposure	Rate	2019	0.787064551	2.76%
DALYs	Middle SDI	Both	Age-standardized	Aortic aneurysm	Smoking	Rate	2019	10.94573354	38.37%
Deaths	Middle SDI	Both	Age-standardized	Aortic aneurysm	Diet high in sodium	Rate	2019	0.100077275	6.77%
Deaths	Middle SDI	Both	Age-standardized	Aortic aneurysm	High systolic blood pressure	Rate	2019	0.511826226	34.65%
Deaths	Middle SDI	Both	Age-standardized	Aortic aneurysm	Lead exposure	Rate	2019	0.041270056	2.79%
Deaths	Middle SDI	Both	Age-standardized	Aortic aneurysm	Smoking	Rate	2019	0.465331689	31.50%
DALYs	North Africa and Middle East	Both	Age-standardized	Aortic aneurysm	Diet high in sodium	Rate	2019	0.404083523	1.44%
DALYs	North Africa and Middle East	Both	Age-standardized	Aortic aneurysm	High systolic blood pressure	Rate	2019	10.48020458	37.40%
DALYs	North Africa and Middle East	Both	Age-standardized	Aortic aneurysm	Lead exposure	Rate	2019	0.8122633	2.90%
DALYs	North Africa and Middle East	Both	Age-standardized	Aortic aneurysm	Smoking	Rate	2019	12.843387	45.84%
Deaths	North Africa and Middle East	Both	Age-standardized	Aortic aneurysm	Diet high in sodium	Rate	2019	0.017255055	1.27%
Deaths	North Africa and Middle East	Both	Age-standardized	Aortic aneurysm	High systolic blood pressure	Rate	2019	0.476745288	35.21%
Deaths	North Africa and Middle East	Both	Age-standardized	Aortic aneurysm	Lead exposure	Rate	2019	0.040904672	3.02%
Deaths	North Africa and Middle East	Both	Age-standardized	Aortic aneurysm	Smoking	Rate	2019	0.523111079	38.64%
DALYs	Oceania	Both	Age-standardized	Aortic aneurysm	Diet high in sodium	Rate	2019	3.353673593	5.55%
DALYs	Oceania	Both	Age-standardized	Aortic aneurysm	High systolic blood pressure	Rate	2019	18.39836119	30.44%
DALYs	Oceania	Both	Age-standardized	Aortic aneurysm	Lead exposure	Rate	2019	0.367270492	0.61%
DALYs	Oceania	Both	Age-standardized	Aortic aneurysm	Smoking	Rate	2019	23.20256581	38.38%
Deaths	Oceania	Both	Age-standardized	Aortic aneurysm	Diet high in sodium	Rate	2019	0.170042536	5.72%
Deaths	Oceania	Both	Age-standardized	Aortic aneurysm	High systolic blood pressure	Rate	2019	0.819293859	27.54%
Deaths	Oceania	Both	Age-standardized	Aortic aneurysm	Lead exposure	Rate	2019	0.019870099	0.67%
Deaths	Oceania	Both	Age-standardized	Aortic aneurysm	Smoking	Rate	2019	0.88537911	29.76%
DALYs	South Asia	Both	Age-standardized	Aortic aneurysm	Diet high in sodium	Rate	2019	1.426331116	4.30%
DALYs	South Asia	Both	Age-standardized	Aortic aneurysm	High systolic blood pressure	Rate	2019	11.85286029	35.71%
DALYs	South Asia	Both	Age-standardized	Aortic aneurysm	Lead exposure	Rate	2019	1.720103862	5.18%
DALYs	South Asia	Both	Age-standardized	Aortic aneurysm	Smoking	Rate	2019	10.41159207	31.37%
Deaths	South Asia	Both	Age-standardized	Aortic aneurysm	Diet high in sodium	Rate	2019	0.061936954	3.64%
Deaths	South Asia	Both	Age-standardized	Aortic aneurysm	High systolic blood pressure	Rate	2019	0.570245349	33.52%
Deaths	South Asia	Both	Age-standardized	Aortic aneurysm	Lead exposure	Rate	2019	0.088070968	5.18%
Deaths	South Asia	Both	Age-standardized	Aortic aneurysm	Smoking	Rate	2019	0.442248946	26.00%
DALYs	Southeast Asia	Both	Age-standardized	Aortic aneurysm	Diet high in sodium	Rate	2019	2.636066986	7.91%
DALYs	Southeast Asia	Both	Age-standardized	Aortic aneurysm	High systolic blood pressure	Rate	2019	12.86480131	38.62%
DALYs	Southeast Asia	Both	Age-standardized	Aortic aneurysm	Lead exposure	Rate	2019	0.539449578	1.62%
DALYs	Southeast Asia	Both	Age-standardized	Aortic aneurysm	Smoking	Rate	2019	12.35883539	37.10%
Deaths	Southeast Asia	Both	Age-standardized	Aortic aneurysm	Diet high in sodium	Rate	2019	0.134103279	7.17%
Deaths	Southeast Asia	Both	Age-standardized	Aortic aneurysm	High systolic blood pressure	Rate	2019	0.68311742	36.50%
Deaths	Southeast Asia	Both	Age-standardized	Aortic aneurysm	Lead exposure	Rate	2019	0.029256761	1.56%
Deaths	Southeast Asia	Both	Age-standardized	Aortic aneurysm	Smoking	Rate	2019	0.568135252	30.36%
DALYs	Southern Latin America	Both	Age-standardized	Aortic aneurysm	Diet high in sodium	Rate	2019	3.126353343	4.69%
DALYs	Southern Latin America	Both	Age-standardized	Aortic aneurysm	High systolic blood pressure	Rate	2019	23.90414296	35.83%
DALYs	Southern Latin America	Both	Age-standardized	Aortic aneurysm	Lead exposure	Rate	2019	0.633558494	0.95%
DALYs	Southern Latin America	Both	Age-standardized	Aortic aneurysm	Smoking	Rate	2019	32.5449411	48.79%
Deaths	Southern Latin America	Both	Age-standardized	Aortic aneurysm	Diet high in sodium	Rate	2019	0.145612454	4.39%
Deaths	Southern Latin America	Both	Age-standardized	Aortic aneurysm	High systolic blood pressure	Rate	2019	1.142535833	34.42%
Deaths	Southern Latin America	Both	Age-standardized	Aortic aneurysm	Lead exposure	Rate	2019	0.031469804	0.95%
Deaths	Southern Latin America	Both	Age-standardized	Aortic aneurysm	Smoking	Rate	2019	1.315077902	39.62%
DALYs	Southern Sub-Saharan Africa	Both	Age-standardized	Aortic aneurysm	Diet high in sodium	Rate	2019	1.113976849	2.63%
DALYs	Southern Sub-Saharan Africa	Both	Age-standardized	Aortic aneurysm	High systolic blood pressure	Rate	2019	18.01035126	42.51%
DALYs	Southern Sub-Saharan Africa	Both	Age-standardized	Aortic aneurysm	Lead exposure	Rate	2019	0.81620921	1.93%
DALYs	Southern Sub-Saharan Africa	Both	Age-standardized	Aortic aneurysm	Smoking	Rate	2019	13.43450523	31.71%
Deaths	Southern Sub-Saharan Africa	Both	Age-standardized	Aortic aneurysm	Diet high in sodium	Rate	2019	0.047386786	2.18%
Deaths	Southern Sub-Saharan Africa	Both	Age-standardized	Aortic aneurysm	High systolic blood pressure	Rate	2019	0.860659599	39.67%
Deaths	Southern Sub-Saharan Africa	Both	Age-standardized	Aortic aneurysm	Lead exposure	Rate	2019	0.040712647	1.88%
Deaths	Southern Sub-Saharan Africa	Both	Age-standardized	Aortic aneurysm	Smoking	Rate	2019	0.543741234	25.06%
DALYs	Tropical Latin America	Both	Age-standardized	Aortic aneurysm	Diet high in sodium	Rate	2019	4.542105031	4.73%
DALYs	Tropical Latin America	Both	Age-standardized	Aortic aneurysm	High systolic blood pressure	Rate	2019	37.60662134	39.13%
DALYs	Tropical Latin America	Both	Age-standardized	Aortic aneurysm	Lead exposure	Rate	2019	1.562105073	1.63%
DALYs	Tropical Latin America	Both	Age-standardized	Aortic aneurysm	Smoking	Rate	2019	41.85561367	43.55%
Deaths	Tropical Latin America	Both	Age-standardized	Aortic aneurysm	Diet high in sodium	Rate	2019	0.203995896	4.50%
Deaths	Tropical Latin America	Both	Age-standardized	Aortic aneurysm	High systolic blood pressure	Rate	2019	1.681425217	37.09%
Deaths	Tropical Latin America	Both	Age-standardized	Aortic aneurysm	Lead exposure	Rate	2019	0.078757	1.74%
Deaths	Tropical Latin America	Both	Age-standardized	Aortic aneurysm	Smoking	Rate	2019	1.662802463	36.68%
DALYs	Western Europe	Both	Age-standardized	Aortic aneurysm	Diet high in sodium	Rate	2019	1.876652649	3.44%
DALYs	Western Europe	Both	Age-standardized	Aortic aneurysm	High systolic blood pressure	Rate	2019	20.50660735	37.56%
DALYs	Western Europe	Both	Age-standardized	Aortic aneurysm	Lead exposure	Rate	2019	0.540277291	0.99%

DALYs	Western Europe	Both	Age-standardized	Aortic aneurysm	Smoking	Rate	2019	26.22509908	48.03%
Deaths	Western Europe	Both	Age-standardized	Aortic aneurysm	Diet high in sodium	Rate	2019	0.088739119	2.89%
Deaths	Western Europe	Both	Age-standardized	Aortic aneurysm	High systolic blood pressure	Rate	2019	1.062105274	34.64%
Deaths	Western Europe	Both	Age-standardized	Aortic aneurysm	Lead exposure	Rate	2019	0.032203972	1.05%
Deaths	Western Europe	Both	Age-standardized	Aortic aneurysm	Smoking	Rate	2019	1.184878591	38.64%
DALYs	Western Sub-Saharan Africa	Both	Age-standardized	Aortic aneurysm	Diet high in sodium	Rate	2019	1.061397127	3.46%
DALYs	Western Sub-Saharan Africa	Both	Age-standardized	Aortic aneurysm	High systolic blood pressure	Rate	2019	13.17432242	42.92%
DALYs	Western Sub-Saharan Africa	Both	Age-standardized	Aortic aneurysm	Lead exposure	Rate	2019	0.730154475	2.38%
DALYs	Western Sub-Saharan Africa	Both	Age-standardized	Aortic aneurysm	Smoking	Rate	2019	6.004548463	19.56%
Deaths	Western Sub-Saharan Africa	Both	Age-standardized	Aortic aneurysm	Diet high in sodium	Rate	2019	0.050353557	3.20%
Deaths	Western Sub-Saharan Africa	Both	Age-standardized	Aortic aneurysm	High systolic blood pressure	Rate	2019	0.635284443	40.37%
Deaths	Western Sub-Saharan Africa	Both	Age-standardized	Aortic aneurysm	Lead exposure	Rate	2019	0.036660137	2.33%
Deaths	Western Sub-Saharan Africa	Both	Age-standardized	Aortic aneurysm	Smoking	Rate	2019	0.24538962	15.59%
DALYs	World Bank High Income	Both	Age-standardized	Aortic aneurysm	Diet high in sodium	Rate	2019	2.595716961	4.70%
DALYs	World Bank High Income	Both	Age-standardized	Aortic aneurysm	High systolic blood pressure	Rate	2019	19.507	35.30%
DALYs	World Bank High Income	Both	Age-standardized	Aortic aneurysm	Lead exposure	Rate	2019	0.491543679	0.89%
DALYs	World Bank High Income	Both	Age-standardized	Aortic aneurysm	Smoking	Rate	2019	25.66421011	46.44%
Deaths	World Bank High Income	Both	Age-standardized	Aortic aneurysm	Diet high in sodium	Rate	2019	0.127514167	4.14%
Deaths	World Bank High Income	Both	Age-standardized	Aortic aneurysm	High systolic blood pressure	Rate	2019	1.008982452	32.75%
Deaths	World Bank High Income	Both	Age-standardized	Aortic aneurysm	Lead exposure	Rate	2019	0.028891635	0.94%
Deaths	World Bank High Income	Both	Age-standardized	Aortic aneurysm	Smoking	Rate	2019	1.115120462	36.20%
DALYs	World Bank Low Income	Both	Age-standardized	Aortic aneurysm	Diet high in sodium	Rate	2019	1.996631047	5.02%
DALYs	World Bank Low Income	Both	Age-standardized	Aortic aneurysm	High systolic blood pressure	Rate	2019	15.08609627	37.90%
DALYs	World Bank Low Income	Both	Age-standardized	Aortic aneurysm	Lead exposure	Rate	2019	1.277123238	3.21%
DALYs	World Bank Low Income	Both	Age-standardized	Aortic aneurysm	Smoking	Rate	2019	10.00452376	25.13%
Deaths	World Bank Low Income	Both	Age-standardized	Aortic aneurysm	Diet high in sodium	Rate	2019	0.095785019	4.92%
Deaths	World Bank Low Income	Both	Age-standardized	Aortic aneurysm	High systolic blood pressure	Rate	2019	0.688292911	35.36%
Deaths	World Bank Low Income	Both	Age-standardized	Aortic aneurysm	Lead exposure	Rate	2019	0.063312376	3.25%
Deaths	World Bank Low Income	Both	Age-standardized	Aortic aneurysm	Smoking	Rate	2019	0.399484161	20.52%
DALYs	World Bank Lower Middle Income	Both	Age-standardized	Aortic aneurysm	Diet high in sodium	Rate	2019	1.575479616	4.70%
DALYs	World Bank Lower Middle Income	Both	Age-standardized	Aortic aneurysm	High systolic blood pressure	Rate	2019	12.69353277	37.89%
DALYs	World Bank Lower Middle Income	Both	Age-standardized	Aortic aneurysm	Lead exposure	Rate	2019	1.298744017	3.88%
DALYs	World Bank Lower Middle Income	Both	Age-standardized	Aortic aneurysm	Smoking	Rate	2019	11.41702028	34.08%
Deaths	World Bank Lower Middle Income	Both	Age-standardized	Aortic aneurysm	Diet high in sodium	Rate	2019	0.071800387	4.21%
Deaths	World Bank Lower Middle Income	Both	Age-standardized	Aortic aneurysm	High systolic blood pressure	Rate	2019	0.609167957	35.69%
Deaths	World Bank Lower Middle Income	Both	Age-standardized	Aortic aneurysm	Lead exposure	Rate	2019	0.066721595	3.91%
Deaths	World Bank Lower Middle Income	Both	Age-standardized	Aortic aneurysm	Smoking	Rate	2019	0.481715952	28.23%
DALYs	World Bank Upper Middle Income	Both	Age-standardized	Aortic aneurysm	Diet high in sodium	Rate	2019	3.022790834	8.54%
DALYs	World Bank Upper Middle Income	Both	Age-standardized	Aortic aneurysm	High systolic blood pressure	Rate	2019	13.62244648	38.50%
DALYs	World Bank Upper Middle Income	Both	Age-standardized	Aortic aneurysm	Lead exposure	Rate	2019	0.72210435	2.04%
DALYs	World Bank Upper Middle Income	Both	Age-standardized	Aortic aneurysm	Smoking	Rate	2019	16.34023039	46.19%
Deaths	World Bank Upper Middle Income	Both	Age-standardized	Aortic aneurysm	Diet high in sodium	Rate	2019	0.127470145	7.38%
Deaths	World Bank Upper Middle Income	Both	Age-standardized	Aortic aneurysm	High systolic blood pressure	Rate	2019	0.623929194	36.13%
Deaths	World Bank Upper Middle Income	Both	Age-standardized	Aortic aneurysm	Lead exposure	Rate	2019	0.036620514	2.12%
Deaths	World Bank Upper Middle Income	Both	Age-standardized	Aortic aneurysm	Smoking	Rate	2019	0.655179342	37.94%
DALYs	Andean Latin America	Female	Age-standardized	Aortic aneurysm	Diet high in sodium	Rate	2019	0.593783178	3.02%
DALYs	Andean Latin America	Female	Age-standardized	Aortic aneurysm	High systolic blood pressure	Rate	2019	5.198661176	26.46%
DALYs	Andean Latin America	Female	Age-standardized	Aortic aneurysm	Lead exposure	Rate	2019	0.305843196	1.56%
DALYs	Andean Latin America	Female	Age-standardized	Aortic aneurysm	Smoking	Rate	2019	1.626354024	8.28%
Deaths	Andean Latin America	Female	Age-standardized	Aortic aneurysm	Diet high in sodium	Rate	2019	0.031285933	3.02%
Deaths	Andean Latin America	Female	Age-standardized	Aortic aneurysm	High systolic blood pressure	Rate	2019	0.280075448	27.07%
Deaths	Andean Latin America	Female	Age-standardized	Aortic aneurysm	Lead exposure	Rate	2019	0.016735276	1.62%
Deaths	Andean Latin America	Female	Age-standardized	Aortic aneurysm	Smoking	Rate	2019	0.062050136	6.00%
DALYs	Australasia	Female	Age-standardized	Aortic aneurysm	Diet high in sodium	Rate	2019	0.527428653	1.47%
DALYs	Australasia	Female	Age-standardized	Aortic aneurysm	High systolic blood pressure	Rate	2019	11.87068644	33.07%
DALYs	Australasia	Female	Age-standardized	Aortic aneurysm	Lead exposure	Rate	2019	0.479820074	1.34%
DALYs	Australasia	Female	Age-standardized	Aortic aneurysm	Smoking	Rate	2019	10.69154106	29.79%
Deaths	Australasia	Female	Age-standardized	Aortic aneurysm	Diet high in sodium	Rate	2019	0.030060019	1.26%
Deaths	Australasia	Female	Age-standardized	Aortic aneurysm	High systolic blood pressure	Rate	2019	0.744801676	31.13%
Deaths	Australasia	Female	Age-standardized	Aortic aneurysm	Lead exposure	Rate	2019	0.032635662	1.36%
Deaths	Australasia	Female	Age-standardized	Aortic aneurysm	Smoking	Rate	2019	0.516885658	21.60%
DALYs	Caribbean	Female	Age-standardized	Aortic aneurysm	Diet high in sodium	Rate	2019	0.642266757	1.94%
DALYs	Caribbean	Female	Age-standardized	Aortic aneurysm	High systolic blood pressure	Rate	2019	11.29781167	34.18%
DALYs	Caribbean	Female	Age-standardized	Aortic aneurysm	Lead exposure	Rate	2019	0.73417874	2.22%
DALYs	Caribbean	Female	Age-standardized	Aortic aneurysm	Smoking	Rate	2019	8.039244946	24.32%
Deaths	Caribbean	Female	Age-standardized	Aortic aneurysm	Diet high in sodium	Rate	2019	0.035828509	1.95%
Deaths	Caribbean	Female	Age-standardized	Aortic aneurysm	High systolic blood pressure	Rate	2019	0.573826208	31.24%
Deaths	Caribbean	Female	Age-standardized	Aortic aneurysm	Lead exposure	Rate	2019	0.040298949	2.19%
Deaths	Caribbean	Female	Age-standardized	Aortic aneurysm	Smoking	Rate	2019	0.356057345	19.38%
DALYs	Central Asia	Female	Age-standardized	Aortic aneurysm	Diet high in sodium	Rate	2019	0.751473155	2.77%
DALYs	Central Asia	Female	Age-standardized	Aortic aneurysm	High systolic blood pressure	Rate	2019	11.03920901	40.66%
DALYs	Central Asia	Female	Age-standardized	Aortic aneurysm	Lead exposure	Rate	2019	0.306118333	1.13%
DALYs	Central Asia	Female	Age-standardized	Aortic aneurysm	Smoking	Rate	2019	2.821286168	10.39%
Deaths	Central Asia	Female	Age-standardized	Aortic aneurysm	Diet high in sodium	Rate	2019	0.04449466	3.00%
Deaths	Central Asia	Female	Age-standardized	Aortic aneurysm	High systolic blood pressure	Rate	2019	0.563269981	37.99%
Deaths	Central Asia	Female	Age-standardized	Aortic aneurysm	Lead exposure	Rate	2019	0.017602956	1.19%
Deaths	Central Asia	Female	Age-standardized	Aortic aneurysm	Smoking	Rate	2019	0.101333827	6.83%
DALYs	Central Europe	Female	Age-standardized	Aortic aneurysm	Diet high in sodium	Rate	2019	2.29739126	6.77%
DALYs	Central Europe	Female	Age-standardized	Aortic aneurysm	High systolic blood pressure	Rate	2019	13.14846181	38.73%
DALYs	Central Europe	Female	Age-standardized	Aortic aneurysm	Lead exposure	Rate	2019	0.243270544	0.72%
DALYs	Central Europe	Female	Age-standardized	Aortic aneurysm	Smoking	Rate	2019	14.10666398	41.55%
Deaths	Central Europe	Female	Age-standardized	Aortic aneurysm	Diet high in sodium	Rate	2019	0.12304678	7.15%
Deaths	Central Europe	Female	Age-standardized	Aortic aneurysm	High systolic blood pressure	Rate	2019	0.638540655	37.11%
Deaths	Central Europe	Female	Age-standardized	Aortic aneurysm	Lead exposure	Rate	2019	0.013298137	0.77%
Deaths	Central Europe	Female	Age-standardized	Aortic aneurysm	Smoking	Rate	2019	0.541030005	31.44%
DALYs	Central Latin America	Female	Age-standardized	Aortic aneurysm	Diet high in sodium	Rate	2019	0.643495476	3.82%
DALYs	Central Latin America	Female	Age-standardized	Aortic aneurysm	High systolic blood pressure	Rate	2019	5.636684727	33.49%
DALYs	Central Latin America	Female	Age-standardized	Aortic aneurysm	Lead exposure	Rate	2019	0.335065858	1.99%
DALYs	Central Latin America	Female	Age-standardized	Aortic aneurysm	Smoking	Rate	2019	2.685889311	15.96%
Deaths	Central Latin America	Female	Age-standardized	Aortic aneurysm	Diet high in sodium	Rate	2019	0.032627619	3.60%
Deaths	Central Latin America	Female	Age-standardized	Aortic aneurysm	High systolic blood pressure	Rate	2019	0.292884964	32.27%
Deaths	Central Latin America	Female	Age-standardized	Aortic aneurysm	Lead exposure	Rate	2019	0.019543861	2.15%
Deaths	Central Latin America	Female	Age-standardized	Aortic aneurysm	Smoking	Rate	2019	0.112792702	12.43%
DALYs	Central Sub-Saharan Africa	Female	Age-standardized	Aortic aneurysm	Diet high in sodium	Rate	2019	0.700330172	1.89%
DALYs	Central Sub-Saharan Africa	Female	Age-standardized	Aortic aneurysm	High systolic blood pressure	Rate	2019	14.75082762	39.83%
DALYs	Central Sub-Saharan Africa	Female	Age-standardized	Aortic aneurysm	Lead exposure	Rate	2019	0.737606568	1.99%
DALYs	Central Sub-Saharan Africa	Female	Age-standardized	Aortic aneurysm	Smoking	Rate	2019	1.702960228	4.60%
Deaths	Central Sub-Saharan Africa	Female	Age-standardized	Aortic aneurysm	Diet high in sodium	Rate	2019	0.035416214	1.80%
Deaths	Central Sub-Saharan Africa	Female	Age-standardized	Aortic aneurysm	High systolic blood pressure	Rate	2019	0.744067213	37.91%
Deaths	Central Sub-Saharan Africa	Female	Age-standardized	Aortic aneurysm	Lead exposure	Rate	2019	0.038645762	1.97%
Deaths	Central Sub-Saharan Africa	Female	Age-standardized	Aortic aneurysm	Smoking	Rate	2019	0.06693545	3.41%
DALYs	East Asia	Female	Age-standardized	Aortic aneurysm	Diet high in sodium	Rate	2019	1.136967904	10.98%

DALYs	East Asia	Female	Age-standardized	Aortic aneurysm	High systolic blood pressure	Rate	2019	3.629913645	35.06%
DALYs	East Asia	Female	Age-standardized	Aortic aneurysm	Lead exposure	Rate	2019	0.239340524	2.31%
DALYs	East Asia	Female	Age-standardized	Aortic aneurysm	Smoking	Rate	2019	1.15385812	11.14%
Deaths	East Asia	Female	Age-standardized	Aortic aneurysm	Diet high in sodium	Rate	2019	0.047805512	8.87%
Deaths	East Asia	Female	Age-standardized	Aortic aneurysm	High systolic blood pressure	Rate	2019	0.180750628	33.53%
Deaths	East Asia	Female	Age-standardized	Aortic aneurysm	Lead exposure	Rate	2019	0.012976338	2.41%
Deaths	East Asia	Female	Age-standardized	Aortic aneurysm	Smoking	Rate	2019	0.051429894	9.54%
DALYs	Eastern Europe	Female	Age-standardized	Aortic aneurysm	Diet high in sodium	Rate	2019	1.258931564	3.08%
DALYs	Eastern Europe	Female	Age-standardized	Aortic aneurysm	High systolic blood pressure	Rate	2019	17.70389421	43.29%
DALYs	Eastern Europe	Female	Age-standardized	Aortic aneurysm	Lead exposure	Rate	2019	0.118769663	0.29%
DALYs	Eastern Europe	Female	Age-standardized	Aortic aneurysm	Smoking	Rate	2019	11.73822161	28.70%
Deaths	Eastern Europe	Female	Age-standardized	Aortic aneurysm	Diet high in sodium	Rate	2019	0.052059511	2.67%
Deaths	Eastern Europe	Female	Age-standardized	Aortic aneurysm	High systolic blood pressure	Rate	2019	0.795771718	40.84%
Deaths	Eastern Europe	Female	Age-standardized	Aortic aneurysm	Lead exposure	Rate	2019	0.005726712	0.29%
Deaths	Eastern Europe	Female	Age-standardized	Aortic aneurysm	Smoking	Rate	2019	0.382190757	19.62%
DALYs	Eastern Sub-Saharan Africa	Female	Age-standardized	Aortic aneurysm	Diet high in sodium	Rate	2019	2.276200339	6.95%
DALYs	Eastern Sub-Saharan Africa	Female	Age-standardized	Aortic aneurysm	High systolic blood pressure	Rate	2019	12.00817444	36.65%
DALYs	Eastern Sub-Saharan Africa	Female	Age-standardized	Aortic aneurysm	Lead exposure	Rate	2019	0.583204236	1.78%
DALYs	Eastern Sub-Saharan Africa	Female	Age-standardized	Aortic aneurysm	Smoking	Rate	2019	2.812388365	8.58%
Deaths	Eastern Sub-Saharan Africa	Female	Age-standardized	Aortic aneurysm	Diet high in sodium	Rate	2019	0.118944961	6.60%
Deaths	Eastern Sub-Saharan Africa	Female	Age-standardized	Aortic aneurysm	High systolic blood pressure	Rate	2019	0.614819668	34.13%
Deaths	Eastern Sub-Saharan Africa	Female	Age-standardized	Aortic aneurysm	Lead exposure	Rate	2019	0.033662056	1.87%
Deaths	Eastern Sub-Saharan Africa	Female	Age-standardized	Aortic aneurysm	Smoking	Rate	2019	0.129469786	7.19%
DALYs	Global	Female	Age-standardized	Aortic aneurysm	Diet high in sodium	Rate	2019	1.055165127	4.29%
DALYs	Global	Female	Age-standardized	Aortic aneurysm	High systolic blood pressure	Rate	2019	8.725008541	35.48%
DALYs	Global	Female	Age-standardized	Aortic aneurysm	Lead exposure	Rate	2019	0.377725966	1.54%
DALYs	Global	Female	Age-standardized	Aortic aneurysm	Smoking	Rate	2019	5.232461899	21.28%
Deaths	Global	Female	Age-standardized	Aortic aneurysm	Diet high in sodium	Rate	2019	0.054589884	3.73%
Deaths	Global	Female	Age-standardized	Aortic aneurysm	High systolic blood pressure	Rate	2019	0.485059826	33.18%
Deaths	Global	Female	Age-standardized	Aortic aneurysm	Lead exposure	Rate	2019	0.020714931	1.42%
Deaths	Global	Female	Age-standardized	Aortic aneurysm	Smoking	Rate	2019	0.239874957	16.41%
DALYs	High SDI	Female	Age-standardized	Aortic aneurysm	Diet high in sodium	Rate	2019	1.105618519	3.28%
DALYs	High SDI	Female	Age-standardized	Aortic aneurysm	High systolic blood pressure	Rate	2019	10.81815857	32.07%
DALYs	High SDI	Female	Age-standardized	Aortic aneurysm	Lead exposure	Rate	2019	0.190411722	0.56%
DALYs	High SDI	Female	Age-standardized	Aortic aneurysm	Smoking	Rate	2019	10.60698927	31.45%
Deaths	High SDI	Female	Age-standardized	Aortic aneurysm	Diet high in sodium	Rate	2019	0.065903336	3.05%
Deaths	High SDI	Female	Age-standardized	Aortic aneurysm	High systolic blood pressure	Rate	2019	0.662006947	30.67%
Deaths	High SDI	Female	Age-standardized	Aortic aneurysm	Lead exposure	Rate	2019	0.013026004	0.60%
Deaths	High SDI	Female	Age-standardized	Aortic aneurysm	Smoking	Rate	2019	0.494293695	22.90%
DALYs	High-income Asia Pacific	Female	Age-standardized	Aortic aneurysm	Diet high in sodium	Rate	2019	2.209684425	5.16%
DALYs	High-income Asia Pacific	Female	Age-standardized	Aortic aneurysm	High systolic blood pressure	Rate	2019	13.38777149	31.29%
DALYs	High-income Asia Pacific	Female	Age-standardized	Aortic aneurysm	Lead exposure	Rate	2019	0.167270851	0.39%
DALYs	High-income Asia Pacific	Female	Age-standardized	Aortic aneurysm	Smoking	Rate	2019	8.28841377	19.37%
Deaths	High-income Asia Pacific	Female	Age-standardized	Aortic aneurysm	Diet high in sodium	Rate	2019	0.138675589	4.68%
Deaths	High-income Asia Pacific	Female	Age-standardized	Aortic aneurysm	High systolic blood pressure	Rate	2019	0.868723023	29.33%
Deaths	High-income Asia Pacific	Female	Age-standardized	Aortic aneurysm	Lead exposure	Rate	2019	0.012870127	0.43%
Deaths	High-income Asia Pacific	Female	Age-standardized	Aortic aneurysm	Smoking	Rate	2019	0.39014867	13.17%
DALYs	High-income North America	Female	Age-standardized	Aortic aneurysm	Diet high in sodium	Rate	2019	0.755853025	2.54%
DALYs	High-income North America	Female	Age-standardized	Aortic aneurysm	High systolic blood pressure	Rate	2019	8.88888295	29.85%
DALYs	High-income North America	Female	Age-standardized	Aortic aneurysm	Lead exposure	Rate	2019	0.193085919	0.65%
DALYs	High-income North America	Female	Age-standardized	Aortic aneurysm	Smoking	Rate	2019	12.12057486	40.70%
Deaths	High-income North America	Female	Age-standardized	Aortic aneurysm	Diet high in sodium	Rate	2019	0.038445322	2.22%
Deaths	High-income North America	Female	Age-standardized	Aortic aneurysm	High systolic blood pressure	Rate	2019	0.514824384	29.72%
Deaths	High-income North America	Female	Age-standardized	Aortic aneurysm	Lead exposure	Rate	2019	0.012605652	0.73%
Deaths	High-income North America	Female	Age-standardized	Aortic aneurysm	Smoking	Rate	2019	0.548496464	31.66%
DALYs	High-middle SDI	Female	Age-standardized	Aortic aneurysm	Diet high in sodium	Rate	2019	1.145536233	4.61%
DALYs	High-middle SDI	Female	Age-standardized	Aortic aneurysm	High systolic blood pressure	Rate	2019	9.589776044	38.62%
DALYs	High-middle SDI	Female	Age-standardized	Aortic aneurysm	Lead exposure	Rate	2019	0.227873808	0.92%
DALYs	High-middle SDI	Female	Age-standardized	Aortic aneurysm	Smoking	Rate	2019	6.975406151	28.09%
Deaths	High-middle SDI	Female	Age-standardized	Aortic aneurysm	Diet high in sodium	Rate	2019	0.053344986	4.10%
Deaths	High-middle SDI	Female	Age-standardized	Aortic aneurysm	High systolic blood pressure	Rate	2019	0.476651335	36.64%
Deaths	High-middle SDI	Female	Age-standardized	Aortic aneurysm	Lead exposure	Rate	2019	0.012700748	0.98%
Deaths	High-middle SDI	Female	Age-standardized	Aortic aneurysm	Smoking	Rate	2019	0.270264998	20.78%
DALYs	Low SDI	Female	Age-standardized	Aortic aneurysm	Diet high in sodium	Rate	2019	1.074797214	4.29%
DALYs	Low SDI	Female	Age-standardized	Aortic aneurysm	High systolic blood pressure	Rate	2019	9.102987272	36.32%
DALYs	Low SDI	Female	Age-standardized	Aortic aneurysm	Lead exposure	Rate	2019	0.854616826	3.41%
DALYs	Low SDI	Female	Age-standardized	Aortic aneurysm	Smoking	Rate	2019	2.618367023	10.45%
Deaths	Low SDI	Female	Age-standardized	Aortic aneurysm	Diet high in sodium	Rate	2019	0.054132354	4.01%
Deaths	Low SDI	Female	Age-standardized	Aortic aneurysm	High systolic blood pressure	Rate	2019	0.455193707	33.76%
Deaths	Low SDI	Female	Age-standardized	Aortic aneurysm	Lead exposure	Rate	2019	0.046022327	3.41%
Deaths	Low SDI	Female	Age-standardized	Aortic aneurysm	Smoking	Rate	2019	0.118509655	8.79%
DALYs	Low-middle SDI	Female	Age-standardized	Aortic aneurysm	Diet high in sodium	Rate	2019	0.890924522	4.15%
DALYs	Low-middle SDI	Female	Age-standardized	Aortic aneurysm	High systolic blood pressure	Rate	2019	7.949446057	37.07%
DALYs	Low-middle SDI	Female	Age-standardized	Aortic aneurysm	Lead exposure	Rate	2019	0.784412591	3.66%
DALYs	Low-middle SDI	Female	Age-standardized	Aortic aneurysm	Smoking	Rate	2019	2.579710871	12.03%
Deaths	Low-middle SDI	Female	Age-standardized	Aortic aneurysm	Diet high in sodium	Rate	2019	0.041780249	3.65%
Deaths	Low-middle SDI	Female	Age-standardized	Aortic aneurysm	High systolic blood pressure	Rate	2019	0.40259762	35.21%
Deaths	Low-middle SDI	Female	Age-standardized	Aortic aneurysm	Lead exposure	Rate	2019	0.042367953	3.71%
Deaths	Low-middle SDI	Female	Age-standardized	Aortic aneurysm	Smoking	Rate	2019	0.112851098	9.87%
DALYs	Middle SDI	Female	Age-standardized	Aortic aneurysm	Diet high in sodium	Rate	2019	0.946117711	5.65%
DALYs	Middle SDI	Female	Age-standardized	Aortic aneurysm	High systolic blood pressure	Rate	2019	6.025331523	36.01%
DALYs	Middle SDI	Female	Age-standardized	Aortic aneurysm	Lead exposure	Rate	2019	0.326078348	1.95%
DALYs	Middle SDI	Female	Age-standardized	Aortic aneurysm	Smoking	Rate	2019	2.14820585	12.84%
Deaths	Middle SDI	Female	Age-standardized	Aortic aneurysm	Diet high in sodium	Rate	2019	0.045029199	4.87%
Deaths	Middle SDI	Female	Age-standardized	Aortic aneurysm	High systolic blood pressure	Rate	2019	0.318720353	34.46%
Deaths	Middle SDI	Female	Age-standardized	Aortic aneurysm	Lead exposure	Rate	2019	0.018356803	1.98%
Deaths	Middle SDI	Female	Age-standardized	Aortic aneurysm	Smoking	Rate	2019	0.093766446	10.14%
DALYs	North Africa and Middle East	Female	Age-standardized	Aortic aneurysm	Diet high in sodium	Rate	2019	0.12040977	0.86%
DALYs	North Africa and Middle East	Female	Age-standardized	Aortic aneurysm	High systolic blood pressure	Rate	2019	5.324362446	37.85%
DALYs	North Africa and Middle East	Female	Age-standardized	Aortic aneurysm	Lead exposure	Rate	2019	0.28856166	2.05%
DALYs	North Africa and Middle East	Female	Age-standardized	Aortic aneurysm	Smoking	Rate	2019	2.250981459	16.00%
Deaths	North Africa and Middle East	Female	Age-standardized	Aortic aneurysm	Diet high in sodium	Rate	2019	0.005636919	0.78%
Deaths	North Africa and Middle East	Female	Age-standardized	Aortic aneurysm	High systolic blood pressure	Rate	2019	0.263769128	36.28%
Deaths	North Africa and Middle East	Female	Age-standardized	Aortic aneurysm	Lead exposure	Rate	2019	0.015517912	2.13%
Deaths	North Africa and Middle East	Female	Age-standardized	Aortic aneurysm	Smoking	Rate	2019	0.086112321	11.84%
DALYs	Oceania	Female	Age-standardized	Aortic aneurysm	Diet high in sodium	Rate	2019	1.875591181	4.60%
DALYs	Oceania	Female	Age-standardized	Aortic aneurysm	High systolic blood pressure	Rate	2019	13.10322555	32.15%
DALYs	Oceania	Female	Age-standardized	Aortic aneurysm	Lead exposure	Rate	2019	0.167529341	0.41%
DALYs	Oceania	Female	Age-standardized	Aortic aneurysm	Smoking	Rate	2019	10.2716299	25.20%
Deaths	Oceania	Female	Age-standardized	Aortic aneurysm	Diet high in sodium	Rate	2019	0.102925863	4.58%
Deaths	Oceania	Female	Age-standardized	Aortic aneurysm	High systolic blood pressure	Rate	2019	0.64431761	28.68%
Deaths	Oceania	Female	Age-standardized	Aortic aneurysm	Lead exposure	Rate	2019	0.010486044	0.47%

Deaths	Oceania	Female	Age-standardized	Aortic aneurysm	Smoking	Rate	2019	0.408560321	18.19%
DALYs	South Asia	Female	Age-standardized	Aortic aneurysm	Diet high in sodium	Rate	2019	0.691376342	3.35%
DALYs	South Asia	Female	Age-standardized	Aortic aneurysm	High systolic blood pressure	Rate	2019	7.518841211	36.44%
DALYs	South Asia	Female	Age-standardized	Aortic aneurysm	Lead exposure	Rate	2019	0.948256888	4.60%
DALYs	South Asia	Female	Age-standardized	Aortic aneurysm	Smoking	Rate	2019	1.758044665	8.52%
Deaths	South Asia	Female	Age-standardized	Aortic aneurysm	Diet high in sodium	Rate	2019	0.032389893	2.84%
Deaths	South Asia	Female	Age-standardized	Aortic aneurysm	High systolic blood pressure	Rate	2019	0.393161984	34.44%
Deaths	South Asia	Female	Age-standardized	Aortic aneurysm	Lead exposure	Rate	2019	0.052199292	4.57%
Deaths	South Asia	Female	Age-standardized	Aortic aneurysm	Smoking	Rate	2019	0.083241126	7.29%
DALYs	Southeast Asia	Female	Age-standardized	Aortic aneurysm	Diet high in sodium	Rate	2019	1.183694439	5.92%
DALYs	Southeast Asia	Female	Age-standardized	Aortic aneurysm	High systolic blood pressure	Rate	2019	7.511547957	37.57%
DALYs	Southeast Asia	Female	Age-standardized	Aortic aneurysm	Lead exposure	Rate	2019	0.21357436	1.07%
DALYs	Southeast Asia	Female	Age-standardized	Aortic aneurysm	Smoking	Rate	2019	1.779812871	8.90%
Deaths	Southeast Asia	Female	Age-standardized	Aortic aneurysm	Diet high in sodium	Rate	2019	0.065141427	5.39%
Deaths	Southeast Asia	Female	Age-standardized	Aortic aneurysm	High systolic blood pressure	Rate	2019	0.436459935	36.14%
Deaths	Southeast Asia	Female	Age-standardized	Aortic aneurysm	Lead exposure	Rate	2019	0.012630975	1.05%
Deaths	Southeast Asia	Female	Age-standardized	Aortic aneurysm	Smoking	Rate	2019	0.086637058	7.17%
DALYs	Southern Latin America	Female	Age-standardized	Aortic aneurysm	Diet high in sodium	Rate	2019	1.195541252	3.52%
DALYs	Southern Latin America	Female	Age-standardized	Aortic aneurysm	High systolic blood pressure	Rate	2019	11.79563697	34.76%
DALYs	Southern Latin America	Female	Age-standardized	Aortic aneurysm	Lead exposure	Rate	2019	0.195220225	0.58%
DALYs	Southern Latin America	Female	Age-standardized	Aortic aneurysm	Smoking	Rate	2019	13.61005523	40.11%
Deaths	Southern Latin America	Female	Age-standardized	Aortic aneurysm	Diet high in sodium	Rate	2019	0.061631918	3.39%
Deaths	Southern Latin America	Female	Age-standardized	Aortic aneurysm	High systolic blood pressure	Rate	2019	0.620794079	34.11%
Deaths	Southern Latin America	Female	Age-standardized	Aortic aneurysm	Lead exposure	Rate	2019	0.010496141	0.58%
Deaths	Southern Latin America	Female	Age-standardized	Aortic aneurysm	Smoking	Rate	2019	0.557555247	30.64%
DALYs	Southern Sub-Saharan Africa	Female	Age-standardized	Aortic aneurysm	Diet high in sodium	Rate	2019	0.618174944	2.59%
DALYs	Southern Sub-Saharan Africa	Female	Age-standardized	Aortic aneurysm	High systolic blood pressure	Rate	2019	10.26880988	42.95%
DALYs	Southern Sub-Saharan Africa	Female	Age-standardized	Aortic aneurysm	Lead exposure	Rate	2019	0.283213915	1.18%
DALYs	Southern Sub-Saharan Africa	Female	Age-standardized	Aortic aneurysm	Smoking	Rate	2019	3.585942699	15.00%
Deaths	Southern Sub-Saharan Africa	Female	Age-standardized	Aortic aneurysm	Diet high in sodium	Rate	2019	0.027810278	2.17%
Deaths	Southern Sub-Saharan Africa	Female	Age-standardized	Aortic aneurysm	High systolic blood pressure	Rate	2019	0.518688608	40.48%
Deaths	Southern Sub-Saharan Africa	Female	Age-standardized	Aortic aneurysm	Lead exposure	Rate	2019	0.014588667	1.14%
Deaths	Southern Sub-Saharan Africa	Female	Age-standardized	Aortic aneurysm	Smoking	Rate	2019	0.152266234	11.88%
DALYs	Tropical Latin America	Female	Age-standardized	Aortic aneurysm	Diet high in sodium	Rate	2019	2.279120331	3.32%
DALYs	Tropical Latin America	Female	Age-standardized	Aortic aneurysm	High systolic blood pressure	Rate	2019	25.12124972	36.62%
DALYs	Tropical Latin America	Female	Age-standardized	Aortic aneurysm	Lead exposure	Rate	2019	0.896936731	1.31%
DALYs	Tropical Latin America	Female	Age-standardized	Aortic aneurysm	Smoking	Rate	2019	24.13388039	35.18%
Deaths	Tropical Latin America	Female	Age-standardized	Aortic aneurysm	Diet high in sodium	Rate	2019	0.111617537	3.27%
Deaths	Tropical Latin America	Female	Age-standardized	Aortic aneurysm	High systolic blood pressure	Rate	2019	1.206543094	35.35%
Deaths	Tropical Latin America	Female	Age-standardized	Aortic aneurysm	Lead exposure	Rate	2019	0.047738972	1.40%
Deaths	Tropical Latin America	Female	Age-standardized	Aortic aneurysm	Smoking	Rate	2019	0.952481773	27.90%
DALYs	Western Europe	Female	Age-standardized	Aortic aneurysm	Diet high in sodium	Rate	2019	0.578614816	1.99%
DALYs	Western Europe	Female	Age-standardized	Aortic aneurysm	High systolic blood pressure	Rate	2019	10.07705896	34.71%
DALYs	Western Europe	Female	Age-standardized	Aortic aneurysm	Lead exposure	Rate	2019	0.183559667	0.63%
DALYs	Western Europe	Female	Age-standardized	Aortic aneurysm	Smoking	Rate	2019	10.83694053	37.33%
Deaths	Western Europe	Female	Age-standardized	Aortic aneurysm	Diet high in sodium	Rate	2019	0.031471746	1.78%
Deaths	Western Europe	Female	Age-standardized	Aortic aneurysm	High systolic blood pressure	Rate	2019	0.586806751	33.12%
Deaths	Western Europe	Female	Age-standardized	Aortic aneurysm	Lead exposure	Rate	2019	0.012272126	0.69%
Deaths	Western Europe	Female	Age-standardized	Aortic aneurysm	Smoking	Rate	2019	0.495579798	27.97%
DALYs	Western Sub-Saharan Africa	Female	Age-standardized	Aortic aneurysm	Diet high in sodium	Rate	2019	0.598614735	3.31%
DALYs	Western Sub-Saharan Africa	Female	Age-standardized	Aortic aneurysm	High systolic blood pressure	Rate	2019	7.771519428	43.02%
DALYs	Western Sub-Saharan Africa	Female	Age-standardized	Aortic aneurysm	Lead exposure	Rate	2019	0.323922092	1.79%
DALYs	Western Sub-Saharan Africa	Female	Age-standardized	Aortic aneurysm	Smoking	Rate	2019	0.833512139	4.61%
Deaths	Western Sub-Saharan Africa	Female	Age-standardized	Aortic aneurysm	Diet high in sodium	Rate	2019	0.028511291	3.03%
Deaths	Western Sub-Saharan Africa	Female	Age-standardized	Aortic aneurysm	High systolic blood pressure	Rate	2019	0.386252317	41.05%
Deaths	Western Sub-Saharan Africa	Female	Age-standardized	Aortic aneurysm	Lead exposure	Rate	2019	0.016842832	1.79%
Deaths	Western Sub-Saharan Africa	Female	Age-standardized	Aortic aneurysm	Smoking	Rate	2019	0.032672591	3.47%
DALYs	World Bank High Income	Female	Age-standardized	Aortic aneurysm	Diet high in sodium	Rate	2019	1.058014634	3.26%
DALYs	World Bank High Income	Female	Age-standardized	Aortic aneurysm	High systolic blood pressure	Rate	2019	10.53041084	32.42%
DALYs	World Bank High Income	Female	Age-standardized	Aortic aneurysm	Lead exposure	Rate	2019	0.192385892	0.59%
DALYs	World Bank High Income	Female	Age-standardized	Aortic aneurysm	Smoking	Rate	2019	10.62278205	32.70%
Deaths	World Bank High Income	Female	Age-standardized	Aortic aneurysm	Diet high in sodium	Rate	2019	0.061746009	3.04%
Deaths	World Bank High Income	Female	Age-standardized	Aortic aneurysm	High systolic blood pressure	Rate	2019	0.628879754	30.99%
Deaths	World Bank High Income	Female	Age-standardized	Aortic aneurysm	Lead exposure	Rate	2019	0.012897429	0.64%
Deaths	World Bank High Income	Female	Age-standardized	Aortic aneurysm	Smoking	Rate	2019	0.480183659	23.67%
DALYs	World Bank Low Income	Female	Age-standardized	Aortic aneurysm	Diet high in sodium	Rate	2019	1.365517604	5.16%
DALYs	World Bank Low Income	Female	Age-standardized	Aortic aneurysm	High systolic blood pressure	Rate	2019	9.604954523	36.33%
DALYs	World Bank Low Income	Female	Age-standardized	Aortic aneurysm	Lead exposure	Rate	2019	0.597041581	2.26%
DALYs	World Bank Low Income	Female	Age-standardized	Aortic aneurysm	Smoking	Rate	2019	2.351222464	8.89%
Deaths	World Bank Low Income	Female	Age-standardized	Aortic aneurysm	Diet high in sodium	Rate	2019	0.068560808	4.93%
Deaths	World Bank Low Income	Female	Age-standardized	Aortic aneurysm	High systolic blood pressure	Rate	2019	0.471165443	33.90%
Deaths	World Bank Low Income	Female	Age-standardized	Aortic aneurysm	Lead exposure	Rate	2019	0.031770254	2.29%
Deaths	World Bank Low Income	Female	Age-standardized	Aortic aneurysm	Smoking	Rate	2019	0.103339144	7.44%
DALYs	World Bank Lower Middle Income	Female	Age-standardized	Aortic aneurysm	Diet high in sodium	Rate	2019	0.752591527	3.73%
DALYs	World Bank Lower Middle Income	Female	Age-standardized	Aortic aneurysm	High systolic blood pressure	Rate	2019	7.667776487	37.99%
DALYs	World Bank Lower Middle Income	Female	Age-standardized	Aortic aneurysm	Lead exposure	Rate	2019	0.659424833	3.27%
DALYs	World Bank Lower Middle Income	Female	Age-standardized	Aortic aneurysm	Smoking	Rate	2019	1.84568833	9.14%
Deaths	World Bank Lower Middle Income	Female	Age-standardized	Aortic aneurysm	Diet high in sodium	Rate	2019	0.037155927	3.36%
Deaths	World Bank Lower Middle Income	Female	Age-standardized	Aortic aneurysm	High systolic blood pressure	Rate	2019	0.401056817	36.26%
Deaths	World Bank Lower Middle Income	Female	Age-standardized	Aortic aneurysm	Lead exposure	Rate	2019	0.036221019	3.27%
Deaths	World Bank Lower Middle Income	Female	Age-standardized	Aortic aneurysm	Smoking	Rate	2019	0.082281227	7.44%
DALYs	World Bank Upper Middle Income	Female	Age-standardized	Aortic aneurysm	Diet high in sodium	Rate	2019	1.171351826	5.74%
DALYs	World Bank Upper Middle Income	Female	Age-standardized	Aortic aneurysm	High systolic blood pressure	Rate	2019	7.608019582	37.29%
DALYs	World Bank Upper Middle Income	Female	Age-standardized	Aortic aneurysm	Lead exposure	Rate	2019	0.283632168	1.39%
DALYs	World Bank Upper Middle Income	Female	Age-standardized	Aortic aneurysm	Smoking	Rate	2019	4.625282773	22.67%
Deaths	World Bank Upper Middle Income	Female	Age-standardized	Aortic aneurysm	Diet high in sodium	Rate	2019	0.053582931	4.97%
Deaths	World Bank Upper Middle Income	Female	Age-standardized	Aortic aneurysm	High systolic blood pressure	Rate	2019	0.383363646	35.54%
Deaths	World Bank Upper Middle Income	Female	Age-standardized	Aortic aneurysm	Lead exposure	Rate	2019	0.015493905	1.44%
Deaths	World Bank Upper Middle Income	Female	Age-standardized	Aortic aneurysm	Smoking	Rate	2019	0.183878462	17.05%
DALYs	Andean Latin America	Male	Age-standardized	Aortic aneurysm	Diet high in sodium	Rate	2019	1.828725621	4.97%
DALYs	Andean Latin America	Male	Age-standardized	Aortic aneurysm	High systolic blood pressure	Rate	2019	10.48127257	28.50%
DALYs	Andean Latin America	Male	Age-standardized	Aortic aneurysm	Lead exposure	Rate	2019	0.870678798	2.37%
DALYs	Andean Latin America	Male	Age-standardized	Aortic aneurysm	Smoking	Rate	2019	10.59811906	28.82%
Deaths	Andean Latin America	Male	Age-standardized	Aortic aneurysm	Diet high in sodium	Rate	2019	0.088715569	4.68%
Deaths	Andean Latin America	Male	Age-standardized	Aortic aneurysm	High systolic blood pressure	Rate	2019	0.507687669	26.81%
Deaths	Andean Latin America	Male	Age-standardized	Aortic aneurysm	Lead exposure	Rate	2019	0.045500698	2.40%
Deaths	Andean Latin America	Male	Age-standardized	Aortic aneurysm	Smoking	Rate	2019	0.451274201	23.83%
DALYs	Australasia	Male	Age-standardized	Aortic aneurysm	Diet high in sodium	Rate	2019	1.789216873	2.44%
DALYs	Australasia	Male	Age-standardized	Aortic aneurysm	High systolic blood pressure	Rate	2019	25.45447655	34.73%
DALYs	Australasia	Male	Age-standardized	Aortic aneurysm	Lead exposure	Rate	2019	1.368971615	1.87%
DALYs	Australasia	Male	Age-standardized	Aortic aneurysm	Smoking	Rate	2019	27.7630991	37.88%
Deaths	Australasia	Male	Age-standardized	Aortic aneurysm	Diet high in sodium	Rate	2019	0.08666541	1.96%

Deaths	Australasia	Male	Age-standardized	Aortic aneurysm	High systolic blood pressure	Rate	2019	1.382859177	31.25%
Deaths	Australasia	Male	Age-standardized	Aortic aneurysm	Lead exposure	Rate	2019	0.084757849	1.92%
Deaths	Australasia	Male	Age-standardized	Aortic aneurysm	Smoking	Rate	2019	1.214955303	27.45%
DALYs	Caribbean	Male	Age-standardized	Aortic aneurysm	Diet high in sodium	Rate	2019	2.818959911	3.33%
DALYs	Caribbean	Male	Age-standardized	Aortic aneurysm	High systolic blood pressure	Rate	2019	30.19602947	35.64%
DALYs	Caribbean	Male	Age-standardized	Aortic aneurysm	Lead exposure	Rate	2019	3.087175636	3.64%
DALYs	Caribbean	Male	Age-standardized	Aortic aneurysm	Smoking	Rate	2019	38.81593646	45.81%
Deaths	Caribbean	Male	Age-standardized	Aortic aneurysm	Diet high in sodium	Rate	2019	0.145579769	3.25%
Deaths	Caribbean	Male	Age-standardized	Aortic aneurysm	High systolic blood pressure	Rate	2019	1.467123947	32.79%
Deaths	Caribbean	Male	Age-standardized	Aortic aneurysm	Lead exposure	Rate	2019	0.162391117	3.63%
Deaths	Caribbean	Male	Age-standardized	Aortic aneurysm	Smoking	Rate	2019	1.776776307	39.71%
DALYs	Central Asia	Male	Age-standardized	Aortic aneurysm	Diet high in sodium	Rate	2019	3.92076935	5.40%
DALYs	Central Asia	Male	Age-standardized	Aortic aneurysm	High systolic blood pressure	Rate	2019	31.26618349	43.09%
DALYs	Central Asia	Male	Age-standardized	Aortic aneurysm	Lead exposure	Rate	2019	1.238463835	1.71%
DALYs	Central Asia	Male	Age-standardized	Aortic aneurysm	Smoking	Rate	2019	41.41133182	57.07%
Deaths	Central Asia	Male	Age-standardized	Aortic aneurysm	Diet high in sodium	Rate	2019	0.192839882	5.36%
Deaths	Central Asia	Male	Age-standardized	Aortic aneurysm	High systolic blood pressure	Rate	2019	1.420512971	39.46%
Deaths	Central Asia	Male	Age-standardized	Aortic aneurysm	Lead exposure	Rate	2019	0.066807021	1.86%
Deaths	Central Asia	Male	Age-standardized	Aortic aneurysm	Smoking	Rate	2019	1.767809054	49.10%
DALYs	Central Europe	Male	Age-standardized	Aortic aneurysm	Diet high in sodium	Rate	2019	13.04969379	12.61%
DALYs	Central Europe	Male	Age-standardized	Aortic aneurysm	High systolic blood pressure	Rate	2019	45.70341579	44.16%
DALYs	Central Europe	Male	Age-standardized	Aortic aneurysm	Lead exposure	Rate	2019	1.572356679	1.52%
DALYs	Central Europe	Male	Age-standardized	Aortic aneurysm	Smoking	Rate	2019	62.12433861	60.03%
Deaths	Central Europe	Male	Age-standardized	Aortic aneurysm	Diet high in sodium	Rate	2019	0.597302673	12.61%
Deaths	Central Europe	Male	Age-standardized	Aortic aneurysm	High systolic blood pressure	Rate	2019	1.944269887	41.05%
Deaths	Central Europe	Male	Age-standardized	Aortic aneurysm	Lead exposure	Rate	2019	0.076886885	1.62%
Deaths	Central Europe	Male	Age-standardized	Aortic aneurysm	Smoking	Rate	2019	2.497294072	52.73%
DALYs	Central Latin America	Male	Age-standardized	Aortic aneurysm	Diet high in sodium	Rate	2019	4.034739286	8.05%
DALYs	Central Latin America	Male	Age-standardized	Aortic aneurysm	High systolic blood pressure	Rate	2019	18.71291297	37.35%
DALYs	Central Latin America	Male	Age-standardized	Aortic aneurysm	Lead exposure	Rate	2019	1.697943686	3.39%
DALYs	Central Latin America	Male	Age-standardized	Aortic aneurysm	Smoking	Rate	2019	17.36145908	34.65%
Deaths	Central Latin America	Male	Age-standardized	Aortic aneurysm	Diet high in sodium	Rate	2019	0.194549871	7.65%
Deaths	Central Latin America	Male	Age-standardized	Aortic aneurysm	High systolic blood pressure	Rate	2019	0.885966978	34.85%
Deaths	Central Latin America	Male	Age-standardized	Aortic aneurysm	Lead exposure	Rate	2019	0.092318558	3.63%
Deaths	Central Latin America	Male	Age-standardized	Aortic aneurysm	Smoking	Rate	2019	0.736168567	28.96%
DALYs	Central Sub-Saharan Africa	Male	Age-standardized	Aortic aneurysm	Diet high in sodium	Rate	2019	1.714192434	2.18%
DALYs	Central Sub-Saharan Africa	Male	Age-standardized	Aortic aneurysm	High systolic blood pressure	Rate	2019	31.46070448	40.01%
DALYs	Central Sub-Saharan Africa	Male	Age-standardized	Aortic aneurysm	Lead exposure	Rate	2019	2.280086413	2.90%
DALYs	Central Sub-Saharan Africa	Male	Age-standardized	Aortic aneurysm	Smoking	Rate	2019	23.84138029	30.32%
Deaths	Central Sub-Saharan Africa	Male	Age-standardized	Aortic aneurysm	Diet high in sodium	Rate	2019	0.079998538	2.13%
Deaths	Central Sub-Saharan Africa	Male	Age-standardized	Aortic aneurysm	High systolic blood pressure	Rate	2019	1.405561448	37.50%
Deaths	Central Sub-Saharan Africa	Male	Age-standardized	Aortic aneurysm	Lead exposure	Rate	2019	0.108642138	2.90%
Deaths	Central Sub-Saharan Africa	Male	Age-standardized	Aortic aneurysm	Smoking	Rate	2019	0.938805832	25.05%
DALYs	East Asia	Male	Age-standardized	Aortic aneurysm	Diet high in sodium	Rate	2019	4.773175484	15.13%
DALYs	East Asia	Male	Age-standardized	Aortic aneurysm	High systolic blood pressure	Rate	2019	11.28272887	35.76%
DALYs	East Asia	Male	Age-standardized	Aortic aneurysm	Lead exposure	Rate	2019	1.119457367	3.55%
DALYs	East Asia	Male	Age-standardized	Aortic aneurysm	Smoking	Rate	2019	19.09146187	60.50%
Deaths	East Asia	Male	Age-standardized	Aortic aneurysm	Diet high in sodium	Rate	2019	0.193123683	12.43%
Deaths	East Asia	Male	Age-standardized	Aortic aneurysm	High systolic blood pressure	Rate	2019	0.515983782	33.20%
Deaths	East Asia	Male	Age-standardized	Aortic aneurysm	Lead exposure	Rate	2019	0.057515691	3.70%
Deaths	East Asia	Male	Age-standardized	Aortic aneurysm	Smoking	Rate	2019	0.799676155	51.46%
DALYs	Eastern Europe	Male	Age-standardized	Aortic aneurysm	Diet high in sodium	Rate	2019	8.654825054	6.08%
DALYs	Eastern Europe	Male	Age-standardized	Aortic aneurysm	High systolic blood pressure	Rate	2019	64.10871061	45.00%
DALYs	Eastern Europe	Male	Age-standardized	Aortic aneurysm	Lead exposure	Rate	2019	0.899051687	0.63%
DALYs	Eastern Europe	Male	Age-standardized	Aortic aneurysm	Smoking	Rate	2019	96.04621758	67.42%
Deaths	Eastern Europe	Male	Age-standardized	Aortic aneurysm	Diet high in sodium	Rate	2019	0.329265883	5.47%
Deaths	Eastern Europe	Male	Age-standardized	Aortic aneurysm	High systolic blood pressure	Rate	2019	2.540821202	42.24%
Deaths	Eastern Europe	Male	Age-standardized	Aortic aneurysm	Lead exposure	Rate	2019	0.039726614	0.66%
Deaths	Eastern Europe	Male	Age-standardized	Aortic aneurysm	Smoking	Rate	2019	3.677185598	61.13%
DALYs	Eastern Sub-Saharan Africa	Male	Age-standardized	Aortic aneurysm	Diet high in sodium	Rate	2019	3.030380074	5.16%
DALYs	Eastern Sub-Saharan Africa	Male	Age-standardized	Aortic aneurysm	High systolic blood pressure	Rate	2019	23.23516729	39.58%
DALYs	Eastern Sub-Saharan Africa	Male	Age-standardized	Aortic aneurysm	Lead exposure	Rate	2019	1.939973403	3.30%
DALYs	Eastern Sub-Saharan Africa	Male	Age-standardized	Aortic aneurysm	Smoking	Rate	2019	17.69573301	30.14%
Deaths	Eastern Sub-Saharan Africa	Male	Age-standardized	Aortic aneurysm	Diet high in sodium	Rate	2019	0.149888692	5.37%
Deaths	Eastern Sub-Saharan Africa	Male	Age-standardized	Aortic aneurysm	High systolic blood pressure	Rate	2019	1.026383575	36.77%
Deaths	Eastern Sub-Saharan Africa	Male	Age-standardized	Aortic aneurysm	Lead exposure	Rate	2019	0.100051144	3.58%
Deaths	Eastern Sub-Saharan Africa	Male	Age-standardized	Aortic aneurysm	Smoking	Rate	2019	0.710452463	25.45%
DALYs	Global	Male	Age-standardized	Aortic aneurysm	Diet high in sodium	Rate	2019	4.01681027	6.76%
DALYs	Global	Male	Age-standardized	Aortic aneurysm	High systolic blood pressure	Rate	2019	22.17581372	37.30%
DALYs	Global	Male	Age-standardized	Aortic aneurysm	Lead exposure	Rate	2019	1.393707052	2.34%
DALYs	Global	Male	Age-standardized	Aortic aneurysm	Smoking	Rate	2019	29.76283826	50.06%
Deaths	Global	Male	Age-standardized	Aortic aneurysm	Diet high in sodium	Rate	2019	0.18360547	5.82%
Deaths	Global	Male	Age-standardized	Aortic aneurysm	High systolic blood pressure	Rate	2019	1.077586466	34.16%
Deaths	Global	Male	Age-standardized	Aortic aneurysm	Lead exposure	Rate	2019	0.072054174	2.28%
Deaths	Global	Male	Age-standardized	Aortic aneurysm	Smoking	Rate	2019	1.309546037	41.51%
DALYs	High SDI	Male	Age-standardized	Aortic aneurysm	Diet high in sodium	Rate	2019	4.074483579	5.20%
DALYs	High SDI	Male	Age-standardized	Aortic aneurysm	High systolic blood pressure	Rate	2019	27.83225799	35.52%
DALYs	High SDI	Male	Age-standardized	Aortic aneurysm	Lead exposure	Rate	2019	0.727196011	0.93%
DALYs	High SDI	Male	Age-standardized	Aortic aneurysm	Smoking	Rate	2019	40.08102337	51.16%
Deaths	High SDI	Male	Age-standardized	Aortic aneurysm	Diet high in sodium	Rate	2019	0.20054456	4.62%
Deaths	High SDI	Male	Age-standardized	Aortic aneurysm	High systolic blood pressure	Rate	2019	1.41789743	32.68%
Deaths	High SDI	Male	Age-standardized	Aortic aneurysm	Lead exposure	Rate	2019	0.044075104	1.02%
Deaths	High SDI	Male	Age-standardized	Aortic aneurysm	Smoking	Rate	2019	1.791733561	41.30%
DALYs	High-income Asia Pacific	Male	Age-standardized	Aortic aneurysm	Diet high in sodium	Rate	2019	6.589647972	7.27%
DALYs	High-income Asia Pacific	Male	Age-standardized	Aortic aneurysm	High systolic blood pressure	Rate	2019	33.61159186	37.07%
DALYs	High-income Asia Pacific	Male	Age-standardized	Aortic aneurysm	Lead exposure	Rate	2019	0.519852339	0.57%
DALYs	High-income Asia Pacific	Male	Age-standardized	Aortic aneurysm	Smoking	Rate	2019	48.45932888	53.45%
Deaths	High-income Asia Pacific	Male	Age-standardized	Aortic aneurysm	Diet high in sodium	Rate	2019	0.340511655	6.60%
Deaths	High-income Asia Pacific	Male	Age-standardized	Aortic aneurysm	High systolic blood pressure	Rate	2019	1.742032588	33.75%
Deaths	High-income Asia Pacific	Male	Age-standardized	Aortic aneurysm	Lead exposure	Rate	2019	0.034220007	0.66%
Deaths	High-income Asia Pacific	Male	Age-standardized	Aortic aneurysm	Smoking	Rate	2019	2.18613587	42.35%
DALYs	High-income North America	Male	Age-standardized	Aortic aneurysm	Diet high in sodium	Rate	2019	3.080071785	4.64%
DALYs	High-income North America	Male	Age-standardized	Aortic aneurysm	High systolic blood pressure	Rate	2019	20.15886471	30.37%
DALYs	High-income North America	Male	Age-standardized	Aortic aneurysm	Lead exposure	Rate	2019	0.673884796	1.02%
DALYs	High-income North America	Male	Age-standardized	Aortic aneurysm	Smoking	Rate	2019	34.88691752	52.55%
Deaths	High-income North America	Male	Age-standardized	Aortic aneurysm	Diet high in sodium	Rate	2019	0.131364538	3.98%
Deaths	High-income North America	Male	Age-standardized	Aortic aneurysm	High systolic blood pressure	Rate	2019	0.934735953	28.30%
Deaths	High-income North America	Male	Age-standardized	Aortic aneurysm	Lead exposure	Rate	2019	0.039276712	1.19%
Deaths	High-income North America	Male	Age-standardized	Aortic aneurysm	Smoking	Rate	2019	1.420913344	43.03%
DALYs	High-middle SDI	Male	Age-standardized	Aortic aneurysm	Diet high in sodium	Rate	2019	5.726204317	7.93%
DALYs	High-middle SDI	Male	Age-standardized	Aortic aneurysm	High systolic blood pressure	Rate	2019	29.23970989	40.47%
DALYs	High-middle SDI	Male	Age-standardized	Aortic aneurysm	Lead exposure	Rate	2019	1.185715485	1.64%

DALYs	High-middle SDI	Male	Age-standardized	Aortic aneurysm	Smoking	Rate	2019	42.03667036	58.18%
Deaths	High-middle SDI	Male	Age-standardized	Aortic aneurysm	Diet high in sodium	Rate	2019	0.243628212	6.96%
Deaths	High-middle SDI	Male	Age-standardized	Aortic aneurysm	High systolic blood pressure	Rate	2019	1.302768003	37.22%
Deaths	High-middle SDI	Male	Age-standardized	Aortic aneurysm	Lead exposure	Rate	2019	0.062090163	1.77%
Deaths	High-middle SDI	Male	Age-standardized	Aortic aneurysm	Smoking	Rate	2019	1.738769891	49.68%
DALYs	Low SDI	Male	Age-standardized	Aortic aneurysm	Diet high in sodium	Rate	2019	2.132081651	4.24%
DALYs	Low SDI	Male	Age-standardized	Aortic aneurysm	High systolic blood pressure	Rate	2019	18.99842324	37.74%
DALYs	Low SDI	Male	Age-standardized	Aortic aneurysm	Lead exposure	Rate	2019	2.369987269	4.71%
DALYs	Low SDI	Male	Age-standardized	Aortic aneurysm	Smoking	Rate	2019	17.09350105	33.96%
Deaths	Low SDI	Male	Age-standardized	Aortic aneurysm	Diet high in sodium	Rate	2019	0.099330152	4.08%
Deaths	Low SDI	Male	Age-standardized	Aortic aneurysm	High systolic blood pressure	Rate	2019	0.852900424	35.06%
Deaths	Low SDI	Male	Age-standardized	Aortic aneurysm	Lead exposure	Rate	2019	0.117528485	4.83%
Deaths	Low SDI	Male	Age-standardized	Aortic aneurysm	Smoking	Rate	2019	0.699254927	28.75%
DALYs	Low-middle SDI	Male	Age-standardized	Aortic aneurysm	Diet high in sodium	Rate	2019	2.764016032	5.73%
DALYs	Low-middle SDI	Male	Age-standardized	Aortic aneurysm	High systolic blood pressure	Rate	2019	17.53766334	36.33%
DALYs	Low-middle SDI	Male	Age-standardized	Aortic aneurysm	Lead exposure	Rate	2019	2.306077094	4.78%
DALYs	Low-middle SDI	Male	Age-standardized	Aortic aneurysm	Smoking	Rate	2019	21.91744289	45.41%
Deaths	Low-middle SDI	Male	Age-standardized	Aortic aneurysm	Diet high in sodium	Rate	2019	0.118924772	5.03%
Deaths	Low-middle SDI	Male	Age-standardized	Aortic aneurysm	High systolic blood pressure	Rate	2019	0.800878655	33.86%
Deaths	Low-middle SDI	Male	Age-standardized	Aortic aneurysm	Lead exposure	Rate	2019	0.115264921	4.87%
Deaths	Low-middle SDI	Male	Age-standardized	Aortic aneurysm	Smoking	Rate	2019	0.924661983	39.10%
DALYs	Middle SDI	Male	Age-standardized	Aortic aneurysm	Diet high in sodium	Rate	2019	3.670177808	8.81%
DALYs	Middle SDI	Male	Age-standardized	Aortic aneurysm	High systolic blood pressure	Rate	2019	15.40036927	36.98%
DALYs	Middle SDI	Male	Age-standardized	Aortic aneurysm	Lead exposure	Rate	2019	1.303480325	3.13%
DALYs	Middle SDI	Male	Age-standardized	Aortic aneurysm	Smoking	Rate	2019	20.46180567	49.14%
Deaths	Middle SDI	Male	Age-standardized	Aortic aneurysm	Diet high in sodium	Rate	2019	0.162860716	7.59%
Deaths	Middle SDI	Male	Age-standardized	Aortic aneurysm	High systolic blood pressure	Rate	2019	0.736319712	34.31%
Deaths	Middle SDI	Male	Age-standardized	Aortic aneurysm	Lead exposure	Rate	2019	0.068896135	3.21%
Deaths	Middle SDI	Male	Age-standardized	Aortic aneurysm	Smoking	Rate	2019	0.890556469	41.49%
DALYs	North Africa and Middle East	Male	Age-standardized	Aortic aneurysm	Diet high in sodium	Rate	2019	0.674338341	1.63%
DALYs	North Africa and Middle East	Male	Age-standardized	Aortic aneurysm	High systolic blood pressure	Rate	2019	15.39911143	37.19%
DALYs	North Africa and Middle East	Male	Age-standardized	Aortic aneurysm	Lead exposure	Rate	2019	1.319518591	3.19%
DALYs	North Africa and Middle East	Male	Age-standardized	Aortic aneurysm	Smoking	Rate	2019	22.92523202	55.37%
Deaths	North Africa and Middle East	Male	Age-standardized	Aortic aneurysm	Diet high in sodium	Rate	2019	0.028555783	1.45%
Deaths	North Africa and Middle East	Male	Age-standardized	Aortic aneurysm	High systolic blood pressure	Rate	2019	0.68415358	34.75%
Deaths	North Africa and Middle East	Male	Age-standardized	Aortic aneurysm	Lead exposure	Rate	2019	0.065942454	3.35%
Deaths	North Africa and Middle East	Male	Age-standardized	Aortic aneurysm	Smoking	Rate	2019	0.94761231	48.13%
DALYs	Oceania	Male	Age-standardized	Aortic aneurysm	Diet high in sodium	Rate	2019	4.723787228	5.98%
DALYs	Oceania	Male	Age-standardized	Aortic aneurysm	High systolic blood pressure	Rate	2019	23.31595381	29.52%
DALYs	Oceania	Male	Age-standardized	Aortic aneurysm	Lead exposure	Rate	2019	0.558204121	0.71%
DALYs	Oceania	Male	Age-standardized	Aortic aneurysm	Smoking	Rate	2019	35.35266897	44.75%
Deaths	Oceania	Male	Age-standardized	Aortic aneurysm	Diet high in sodium	Rate	2019	0.234169247	6.38%
Deaths	Oceania	Male	Age-standardized	Aortic aneurysm	High systolic blood pressure	Rate	2019	0.97983389	26.69%
Deaths	Oceania	Male	Age-standardized	Aortic aneurysm	Lead exposure	Rate	2019	0.029366551	0.80%
Deaths	Oceania	Male	Age-standardized	Aortic aneurysm	Smoking	Rate	2019	1.343412795	36.59%
DALYs	South Asia	Male	Age-standardized	Aortic aneurysm	Diet high in sodium	Rate	2019	2.175122863	4.71%
DALYs	South Asia	Male	Age-standardized	Aortic aneurysm	High systolic blood pressure	Rate	2019	16.2798795	35.26%
DALYs	South Asia	Male	Age-standardized	Aortic aneurysm	Lead exposure	Rate	2019	2.520169376	5.46%
DALYs	South Asia	Male	Age-standardized	Aortic aneurysm	Smoking	Rate	2019	19.26463014	41.73%
Deaths	South Asia	Male	Age-standardized	Aortic aneurysm	Diet high in sodium	Rate	2019	0.092946676	4.04%
Deaths	South Asia	Male	Age-standardized	Aortic aneurysm	High systolic blood pressure	Rate	2019	0.756240829	32.87%
Deaths	South Asia	Male	Age-standardized	Aortic aneurysm	Lead exposure	Rate	2019	0.126465935	5.50%
Deaths	South Asia	Male	Age-standardized	Aortic aneurysm	Smoking	Rate	2019	0.820839447	35.68%
DALYs	Southeast Asia	Male	Age-standardized	Aortic aneurysm	Diet high in sodium	Rate	2019	4.382590966	8.83%
DALYs	Southeast Asia	Male	Age-standardized	Aortic aneurysm	High systolic blood pressure	Rate	2019	19.252208	38.81%
DALYs	Southeast Asia	Male	Age-standardized	Aortic aneurysm	Lead exposure	Rate	2019	0.940801343	1.90%
DALYs	Southeast Asia	Male	Age-standardized	Aortic aneurysm	Smoking	Rate	2019	24.92294768	50.24%
Deaths	Southeast Asia	Male	Age-standardized	Aortic aneurysm	Diet high in sodium	Rate	2019	0.222320153	8.05%
Deaths	Southeast Asia	Male	Age-standardized	Aortic aneurysm	High systolic blood pressure	Rate	2019	1.000395685	36.23%
Deaths	Southeast Asia	Male	Age-standardized	Aortic aneurysm	Lead exposure	Rate	2019	0.051383934	1.86%
Deaths	Southeast Asia	Male	Age-standardized	Aortic aneurysm	Smoking	Rate	2019	1.186926687	42.99%
DALYs	Southern Latin America	Male	Age-standardized	Aortic aneurysm	Diet high in sodium	Rate	2019	5.444054309	5.10%
DALYs	Southern Latin America	Male	Age-standardized	Aortic aneurysm	High systolic blood pressure	Rate	2019	38.41129644	35.97%
DALYs	Southern Latin America	Male	Age-standardized	Aortic aneurysm	Lead exposure	Rate	2019	1.175451809	1.10%
DALYs	Southern Latin America	Male	Age-standardized	Aortic aneurysm	Smoking	Rate	2019	54.66743724	51.19%
Deaths	Southern Latin America	Male	Age-standardized	Aortic aneurysm	Diet high in sodium	Rate	2019	0.253528877	4.77%
Deaths	Southern Latin America	Male	Age-standardized	Aortic aneurysm	High systolic blood pressure	Rate	2019	1.811182933	34.11%
Deaths	Southern Latin America	Male	Age-standardized	Aortic aneurysm	Lead exposure	Rate	2019	0.059326244	1.12%
Deaths	Southern Latin America	Male	Age-standardized	Aortic aneurysm	Smoking	Rate	2019	2.242177596	42.23%
DALYs	Southern Sub-Saharan Africa	Male	Age-standardized	Aortic aneurysm	Diet high in sodium	Rate	2019	1.771434472	2.56%
DALYs	Southern Sub-Saharan Africa	Male	Age-standardized	Aortic aneurysm	High systolic blood pressure	Rate	2019	28.79925344	41.64%
DALYs	Southern Sub-Saharan Africa	Male	Age-standardized	Aortic aneurysm	Lead exposure	Rate	2019	1.596682537	2.31%
DALYs	Southern Sub-Saharan Africa	Male	Age-standardized	Aortic aneurysm	Smoking	Rate	2019	26.5408072	38.37%
Deaths	Southern Sub-Saharan Africa	Male	Age-standardized	Aortic aneurysm	Diet high in sodium	Rate	2019	0.076726825	2.10%
Deaths	Southern Sub-Saharan Africa	Male	Age-standardized	Aortic aneurysm	High systolic blood pressure	Rate	2019	1.399214881	38.24%
Deaths	Southern Sub-Saharan Africa	Male	Age-standardized	Aortic aneurysm	Lead exposure	Rate	2019	0.083856496	2.29%
Deaths	Southern Sub-Saharan Africa	Male	Age-standardized	Aortic aneurysm	Smoking	Rate	2019	1.120345453	30.62%
DALYs	Tropical Latin America	Male	Age-standardized	Aortic aneurysm	Diet high in sodium	Rate	2019	7.200573739	5.61%
DALYs	Tropical Latin America	Male	Age-standardized	Aortic aneurysm	High systolic blood pressure	Rate	2019	52.06347878	40.54%
DALYs	Tropical Latin America	Male	Age-standardized	Aortic aneurysm	Lead exposure	Rate	2019	2.367413299	1.84%
DALYs	Tropical Latin America	Male	Age-standardized	Aortic aneurysm	Smoking	Rate	2019	62.59348086	48.74%
Deaths	Tropical Latin America	Male	Age-standardized	Aortic aneurysm	Diet high in sodium	Rate	2019	0.317411762	5.36%
Deaths	Tropical Latin America	Male	Age-standardized	Aortic aneurysm	High systolic blood pressure	Rate	2019	2.253315934	38.08%
Deaths	Tropical Latin America	Male	Age-standardized	Aortic aneurysm	Lead exposure	Rate	2019	0.118241336	2.00%
Deaths	Tropical Latin America	Male	Age-standardized	Aortic aneurysm	Smoking	Rate	2019	2.530985464	42.77%
DALYs	Western Europe	Male	Age-standardized	Aortic aneurysm	Diet high in sodium	Rate	2019	3.328958694	3.94%
DALYs	Western Europe	Male	Age-standardized	Aortic aneurysm	High systolic blood pressure	Rate	2019	32.27509343	38.20%
DALYs	Western Europe	Male	Age-standardized	Aortic aneurysm	Lead exposure	Rate	2019	0.968867276	1.15%
DALYs	Western Europe	Male	Age-standardized	Aortic aneurysm	Smoking	Rate	2019	43.42772082	51.40%
Deaths	Western Europe	Male	Age-standardized	Aortic aneurysm	Diet high in sodium	Rate	2019	0.158884632	3.34%
Deaths	Western Europe	Male	Age-standardized	Aortic aneurysm	High systolic blood pressure	Rate	2019	1.649572772	34.68%
Deaths	Western Europe	Male	Age-standardized	Aortic aneurysm	Lead exposure	Rate	2019	0.058807005	1.24%
Deaths	Western Europe	Male	Age-standardized	Aortic aneurysm	Smoking	Rate	2019	2.013416239	42.33%
DALYs	Western Sub-Saharan Africa	Male	Age-standardized	Aortic aneurysm	Diet high in sodium	Rate	2019	1.568726967	3.52%
DALYs	Western Sub-Saharan Africa	Male	Age-standardized	Aortic aneurysm	High systolic blood pressure	Rate	2019	19.09856197	42.84%
DALYs	Western Sub-Saharan Africa	Male	Age-standardized	Aortic aneurysm	Lead exposure	Rate	2019	1.175272551	2.64%
DALYs	Western Sub-Saharan Africa	Male	Age-standardized	Aortic aneurysm	Smoking	Rate	2019	11.65027595	26.13%
Deaths	Western Sub-Saharan Africa	Male	Age-standardized	Aortic aneurysm	Diet high in sodium	Rate	2019	0.074535179	3.27%
Deaths	Western Sub-Saharan Africa	Male	Age-standardized	Aortic aneurysm	High systolic blood pressure	Rate	2019	0.910627234	39.96%
Deaths	Western Sub-Saharan Africa	Male	Age-standardized	Aortic aneurysm	Lead exposure	Rate	2019	0.05859955	2.57%
Deaths	Western Sub-Saharan Africa	Male	Age-standardized	Aortic aneurysm	Smoking	Rate	2019	0.477656294	20.96%
DALYs	World Bank High Income	Male	Age-standardized	Aortic aneurysm	Diet high in sodium	Rate	2019	4.292335256	5.29%

DALYs	World Bank High Income	Male	Age-standardized	Aortic aneurysm	High systolic blood pressure	Rate	2019	29.39245117	36.25%
DALYs	World Bank High Income	Male	Age-standardized	Aortic aneurysm	Lead exposure	Rate	2019	0.84683591	1.04%
DALYs	World Bank High Income	Male	Age-standardized	Aortic aneurysm	Smoking	Rate	2019	42.17419189	52.02%
Deaths	World Bank High Income	Male	Age-standardized	Aortic aneurysm	Diet high in sodium	Rate	2019	0.20660875	4.68%
Deaths	World Bank High Income	Male	Age-standardized	Aortic aneurysm	High systolic blood pressure	Rate	2019	1.467216067	33.26%
Deaths	World Bank High Income	Male	Age-standardized	Aortic aneurysm	Lead exposure	Rate	2019	0.050015261	1.13%
Deaths	World Bank High Income	Male	Age-standardized	Aortic aneurysm	Smoking	Rate	2019	1.870715831	42.40%
DALYs	World Bank Low Income	Male	Age-standardized	Aortic aneurysm	Diet high in sodium	Rate	2019	2.715077043	4.94%
DALYs	World Bank Low Income	Male	Age-standardized	Aortic aneurysm	High systolic blood pressure	Rate	2019	21.23694024	38.61%
DALYs	World Bank Low Income	Male	Age-standardized	Aortic aneurysm	Lead exposure	Rate	2019	2.067257146	3.76%
DALYs	World Bank Low Income	Male	Age-standardized	Aortic aneurysm	Smoking	Rate	2019	18.51382978	33.66%
Deaths	World Bank Low Income	Male	Age-standardized	Aortic aneurysm	Diet high in sodium	Rate	2019	0.128085382	4.89%
Deaths	World Bank Low Income	Male	Age-standardized	Aortic aneurysm	High systolic blood pressure	Rate	2019	0.943232603	36.03%
Deaths	World Bank Low Income	Male	Age-standardized	Aortic aneurysm	Lead exposure	Rate	2019	0.102415091	3.91%
Deaths	World Bank Low Income	Male	Age-standardized	Aortic aneurysm	Smoking	Rate	2019	0.743653057	28.41%
DALYs	World Bank Lower Middle Income	Male	Age-standardized	Aortic aneurysm	Diet high in sodium	Rate	2019	2.456386014	5.12%
DALYs	World Bank Lower Middle Income	Male	Age-standardized	Aortic aneurysm	High systolic blood pressure	Rate	2019	18.06561885	37.67%
DALYs	World Bank Lower Middle Income	Male	Age-standardized	Aortic aneurysm	Lead exposure	Rate	2019	1.998534709	4.17%
DALYs	World Bank Lower Middle Income	Male	Age-standardized	Aortic aneurysm	Smoking	Rate	2019	21.65703949	45.16%
Deaths	World Bank Lower Middle Income	Male	Age-standardized	Aortic aneurysm	Diet high in sodium	Rate	2019	0.110369529	4.61%
Deaths	World Bank Lower Middle Income	Male	Age-standardized	Aortic aneurysm	High systolic blood pressure	Rate	2019	0.840551688	35.13%
Deaths	World Bank Lower Middle Income	Male	Age-standardized	Aortic aneurysm	Lead exposure	Rate	2019	0.101693155	4.25%
Deaths	World Bank Lower Middle Income	Male	Age-standardized	Aortic aneurysm	Smoking	Rate	2019	0.927206201	38.75%
DALYs	World Bank Upper Middle Income	Male	Age-standardized	Aortic aneurysm	Diet high in sodium	Rate	2019	5.051517236	9.66%
DALYs	World Bank Upper Middle Income	Male	Age-standardized	Aortic aneurysm	High systolic blood pressure	Rate	2019	20.23737963	38.72%
DALYs	World Bank Upper Middle Income	Male	Age-standardized	Aortic aneurysm	Lead exposure	Rate	2019	1.22864812	2.35%
DALYs	World Bank Upper Middle Income	Male	Age-standardized	Aortic aneurysm	Smoking	Rate	2019	29.19260067	55.85%
Deaths	World Bank Upper Middle Income	Male	Age-standardized	Aortic aneurysm	Diet high in sodium	Rate	2019	0.214388334	8.43%
Deaths	World Bank Upper Middle Income	Male	Age-standardized	Aortic aneurysm	High systolic blood pressure	Rate	2019	0.910999315	35.82%
Deaths	World Bank Upper Middle Income	Male	Age-standardized	Aortic aneurysm	Lead exposure	Rate	2019	0.063504561	2.50%
Deaths	World Bank Upper Middle Income	Male	Age-standardized	Aortic aneurysm	Smoking	Rate	2019	1.20855901	47.53%
