# Supplementary material for: Burden of Aortic Aneurysm and Its Attributable Risk Factors from 1990 to 2019: An Analysis of the Global Burden of Disease Study 2019
Source: Front Cardiovasc Med. 2022 May 31;9:901225. doi: 10.3389/fcvm.2022.901225 (PMC9197430; doi:10.3389/fcvm.2022.901225)
Supplement: Supplementary Table 10 — Correlation analysis of aortic aneurism-related age-standardized deaths and DALYs in 2019, by gender. DALY, disability-adjusted life year rate. [file Data_Sheet_10.PDF]

row	column	Gender	correlation coefficients	p
Adult hiv death rate	Deaths	Male	0.796001598	0
Adult hiv death rate	Deaths	Female	-0.502977551	0
Adult hiv death rate	DALYs	Male	0.757350646	0
Adult hiv death rate	DALYs	Female	-0.444854125	0
Adult underweight proportion	Deaths	Male	0.687756011	0
Adult underweight proportion	Deaths	Female	-0.483644102	0
Adult underweight proportion	DALYs	Male	0.656197712	0
Adult underweight proportion	DALYs	Female	-0.42320561	0
Alcohol abstainer proportion	DALYs	Female	-0.682149307	0
Alcohol abstainer proportion	DALYs	Male	-0.683846248	0
Alcohol abstainer proportion	Deaths	Female	-0.726714528	0
Alcohol abstainer proportion	Deaths	Male	-0.710556084	0
Alcohol binge drinker proportion	DALYs	Female	0.688189577	0
Alcohol binge drinker proportion	DALYs	Male	0.737030005	0
Alcohol binge drinker proportion	Deaths	Female	0.723956636	0
Alcohol binge drinker proportion	Deaths	Male	0.764051585	0
Alcohol drinker proportion	DALYs	Female	0.683944105	0
Alcohol drinker proportion	DALYs	Male	0.654450293	0
Alcohol drinker proportion	Deaths	Female	0.729258306	0
Alcohol drinker proportion	Deaths	Male	0.686036248	0
Alcohol consumption in grams per day	DALYs	Female	0.388360226	0
Alcohol consumption in grams per day	DALYs	Male	0.447414176	0
Alcohol consumption in grams per day	Deaths	Female	0.420973254	0
Alcohol consumption in grams per day	Deaths	Male	0.460323932	0
Birth prevalence of congenital chromosomal anomalies	Deaths	Male	0.558260241	0
Birth prevalence of congenital chromosomal anomalies	Deaths	Female	0.560543836	0
Birth prevalence of congenital chromosomal anomalies	DALYs	Male	0.492710632	0
Birth prevalence of congenital chromosomal anomalies	DALYs	Female	0.487722481	0
Birth prevalence of congenital heart disease	Deaths	Male	0.544581189	0
Birth prevalence of congenital heart disease	Deaths	Female	0.452096334	0
Birth prevalence of congenital heart disease	DALYs	Male	0.503645296	0
Birth prevalence of congenital heart disease	DALYs	Female	0.399304679	0
BMI	Deaths	Male	0.299469821	1.67E-13
BMI	Deaths	Female	-0.01304094	0.753768
BMI	DALYs	Male	0.299469821	1.67E-13
BMI	DALYs	Female	-0.016869305	0.684912
Bone mineral density	Deaths	Male	0.776692122	0
Bone mineral density	Deaths	Female	-0.493638033	0
Bone mineral density	DALYs	Male	0.741094733	0
Bone mineral density	DALYs	Female	-0.455762862	0
Prevalence of cannabis dependence in women of reproductive age	DALYs	Female	0.540578011	0
Prevalence of cannabis dependence in women of reproductive age	Deaths	Female	0.612979911	0
Prevalence of cocaine dependence in women of reproductive-age	DALYs	Female	0.441138685	0
Prevalence of cocaine dependence in women of reproductive-age	Deaths	Female	0.486995954	0
Death rate of diabetes under 15 years old (per 100,000)	Deaths	Male	-0.484141737	0
Death rate of diabetes under 15 years old (per 100,000)	Deaths	Female	0.692164973	0
Death rate of diabetes under 15 years old (per 100,000)	DALYs	Male	-0.438419607	0
Death rate of diabetes under 15 years old (per 100,000)	DALYs	Female	0.668099058	0
Diabetes Fasting Plasma Glucose (mmol/L)	Deaths	Male	0.106700878	0.010061
Diabetes Fasting Plasma Glucose (mmol/L)	Deaths	Female	-0.039281294	0.344577
Diabetes Fasting Plasma Glucose (mmol/L)	DALYs	Male	0.110952591	0.007431
Diabetes Fasting Plasma Glucose (mmol/L)	DALYs	Female	-0.051832254	0.212211
Diabetes Prevalence	Deaths	Male	-0.597873042	0
Diabetes Prevalence	Deaths	Female	-0.589425418	0
Diabetes Prevalence	DALYs	Male	-0.553519711	0
Diabetes Prevalence	DALYs	Female	-0.546954177	0
Diet high in trans fatty acids	DALYs	Female	0.160630106	0.000101
Diet high in trans fatty acids	DALYs	Male	0.175935036	2.00E-05
Diet high in trans fatty acids	Deaths	Female	0.202872441	8.19E-07
Diet high in trans fatty acids	Deaths	Male	0.209807058	3.34E-07
Education (years per capita)	DALYs	Female	0.479913924	0
Education (years per capita)	DALYs	Male	0.552863374	0
Education (years per capita)	Deaths	Female	0.540858929	0
Education (years per capita)	Deaths	Male	0.608410952	0
Education Absolute Inequality	Deaths	Male	-0.199960171	7.11E-07
Education Absolute Inequality	Deaths	Female	-0.113568329	0.005163
Education Absolute Inequality	DALYs	Male	-0.185860187	4.18E-06

Education Absolute Inequality	DALYs	Female	-0.069953955	0.08558
Education Relative Inequality	Deaths	Male	-0.464737151	0
Education Relative Inequality	Deaths	Female	-0.441301728	0
Education Relative Inequality	DALYs	Male	-0.431875412	0
Education Relative Inequality	DALYs	Female	-0.388229906	0
Female HIV death rate	Deaths	Male	/	
Female HIV death rate	Deaths	Female	-0.480566687	0
Female HIV death rate	DALYs	Male	/	
Female HIV death rate	DALYs	Female	-0.412069561	0
Mean Hemoglobin	Deaths	Male	0.304880892	5.80E-14
Mean Hemoglobin	Deaths	Female	0.138822152	0.000793
Mean Hemoglobin	DALYs	Male	0.238769899	5.63E-09
Mean Hemoglobin	DALYs	Female	0.089143043	0.031686
Hepatitis A Seroprevalence	DALYs	Female	-0.588460816	0
Hepatitis A Seroprevalence	Deaths	Male	-0.719459566	0
Hepatitis A Seroprevalence	Deaths	Female	-0.639601971	0
Hepatitis A Seroprevalence	DALYs	Male	-0.673546107	0
Hepatitis B Seroprevalence	DALYs	Female	-0.428816847	0
Hepatitis B Seroprevalence	Deaths	Male	-0.449296939	0
Hepatitis B Seroprevalence	Deaths	Female	-0.468464646	0
Hepatitis B Seroprevalence	DALYs	Male	-0.395812165	0
Hepatitis C Seroprevalence	DALYs	Female	-0.275638494	1.37E-11
Hepatitis C Seroprevalence	Deaths	Male	-0.449572522	0
Hepatitis C Seroprevalence	Deaths	Female	-0.321251957	2.00E-15
Hepatitis C Seroprevalence	DALYs	Male	-0.393861608	0
Hepatitis E Seroprevalence	DALYs	Female	-0.40401688	0
Hepatitis E Seroprevalence	Deaths	Male	-0.629323204	0
Hepatitis E Seroprevalence	Deaths	Female	-0.418730225	0
Hepatitis E Seroprevalence	DALYs	Male	-0.59532504	0
HIV mortality, females, 10-54	Deaths	Male	/	
HIV mortality, females, 10-54	Deaths	Female	-0.472132974	0
HIV mortality, females, 10-54	DALYs	Male	/	
HIV mortality, females, 10-54	DALYs	Female	-0.404121454	0
HIV Prevalence	DALYs	Female	-0.078084632	0.059976
HIV Prevalence	Deaths	Male	-0.209464785	3.49E-07
HIV Prevalence	Deaths	Female	-0.119477312	0.003926
HIV Prevalence	DALYs	Male	-0.183967845	8.08E-06
Homicide rate	Deaths	Male	-0.620003407	0
Homicide rate	Deaths	Female	-0.560440728	0
Homicide rate	DALYs	Male	-0.540593181	0
Homicide rate	DALYs	Female	-0.474554431	0
Intravenous drug use	Deaths	Male	0.3740042	0
Intravenous drug use	Deaths	Female	0.217119983	1.25E-07
Intravenous drug use	DALYs	Male	0.382765103	0
Intravenous drug use	DALYs	Female	0.23120896	1.72E-08
Mean HIV crude death rate	Deaths	Male	-0.539397316	0
Mean HIV crude death rate	Deaths	Female	-0.471733525	0
Mean HIV crude death rate	DALYs	Male	-0.487105608	0
Mean HIV crude death rate	DALYs	Female	-0.403907967	0
Mean Age of Initiation Smoking	Deaths	Female	-0.499515739	8.88E-15
Mean Age of Initiation Smoking	Deaths	Male	-0.325291281	1.29E-06
Mean Age of Initiation Smoking	DALYs	Female	-0.482470248	9.28E-14
Mean Age of Initiation Smoking	DALYs	Male	-0.281741779	3.15E-05
Mean birthweight	Deaths	Male	-0.521951954	0
Mean birthweight	Deaths	Female	-0.456062765	0
Mean birthweight	DALYs	Male	-0.496766179	0
Mean birthweight	DALYs	Female	-0.420465641	0
Mean cholesterol	Deaths	Male	0.621810682	0
Mean cholesterol	Deaths	Female	0.390968754	0
Mean cholesterol	DALYs	Male	0.602767574	0
Mean cholesterol	DALYs	Female	0.403427549	0
Melanoma incidence	Deaths	Male	0.683392177	0
Melanoma incidence	Deaths	Female	0.694741536	0
Melanoma incidence	DALYs	Male	0.636539268	0
Melanoma incidence	DALYs	Female	0.634798879	0
NAFLD/NASH prevalence	Deaths	Male	-0.048045948	0.237996
NAFLD/NASH prevalence	Deaths	Female	-0.312920383	3.33E-15
NAFLD/NASH prevalence	DALYs	Male	-0.071071183	0.080687

NAFLD/NASH prevalence	DALYs	Female	-0.28274412	1.39E-12
Neonatal Mortality Rate	Deaths	Male	-0.643954468	0
Neonatal Mortality Rate	Deaths	Female	-0.560170448	0
Neonatal Mortality Rate	DALYs	Male	-0.596145924	0
Neonatal Mortality Rate	DALYs	Female	-0.497998018	0
Prevalence of opioid dependence in women of reproductive age	DALYs	Female	0.311727148	1.47E-14
Prevalence of opioid dependence in women of reproductive age	Deaths	Female	0.361679392	0
Pelvic inflammatory disease	Deaths	Male	0.124681334	0.002607
Pelvic inflammatory disease	Deaths	Female	-0.197129209	1.68E-06
Pelvic inflammatory disease	DALYs	Male	0.125882054	0.002367
Pelvic inflammatory disease	DALYs	Female	-0.18527115	6.95E-06
Pelvic inflammatory disease age-standardized prevalence	Deaths	Male	0.120899226	0.003516
Pelvic inflammatory disease age-standardized prevalence	Deaths	Female	-0.196456098	1.83E-06
Pelvic inflammatory disease age-standardized prevalence	DALYs	Male	0.121727688	0.003296
Pelvic inflammatory disease age-standardized prevalence	DALYs	Female	-0.184799343	7.34E-06
Percent of all fevers effectively treated	Deaths	Male	-0.651147436	0
Percent of all fevers effectively treated	Deaths	Female	-0.654589318	0
Percent of all fevers effectively treated	DALYs	Male	-0.626664912	0
Percent of all fevers effectively treated	DALYs	Female	-0.623725943	0
Total Physical Activity (MET-min/week)	DALYs	Female	-0.541503641	0
Total Physical Activity (MET-min/week)	DALYs	Male	-0.478762299	0
Total Physical Activity (MET-min/week)	Deaths	Female	-0.545783622	0
Total Physical Activity (MET-min/week)	Deaths	Male	-0.504812179	0
Prevalence of obesity	Deaths	Male	0.237848005	6.47E-09
Prevalence of obesity	Deaths	Female	-0.038962329	0.348512
Prevalence of obesity	DALYs	Male	0.213835138	1.96E-07
Prevalence of obesity	DALYs	Female	-0.030823206	0.458368
Prevalence of Severe Anemia	Deaths	Male	-0.587117899	0
Prevalence of Severe Anemia	Deaths	Female	-0.395988566	0
Prevalence of Severe Anemia	DALYs	Male	-0.527541357	0
Prevalence of Severe Anemia	DALYs	Female	-0.331040785	2.22E-16
Proportion of cirrhosis due to alcohol	Deaths	Male	0.704135092	0
Proportion of cirrhosis due to alcohol	Deaths	Female	0.57164823	0
Proportion of cirrhosis due to alcohol	DALYs	Male	0.672848032	0
Proportion of cirrhosis due to alcohol	DALYs	Female	0.537726856	0
Proportion of cirrhosis due to hepatitis B	Deaths	Male	-0.169994365	3.81E-05
Proportion of cirrhosis due to hepatitis B	Deaths	Female	-0.116321627	0.004996
Proportion of cirrhosis due to hepatitis B	DALYs	Male	-0.129109562	0.001818
Proportion of cirrhosis due to hepatitis B	DALYs	Female	-0.083824731	0.043413
Proportion of cirrhosis due to hepatitis C	Deaths	Male	-0.355424807	0
Proportion of cirrhosis due to hepatitis C	Deaths	Female	-0.376812414	0
Proportion of cirrhosis due to hepatitis C	DALYs	Male	-0.329231751	4.44E-16
Proportion of cirrhosis due to hepatitis C	DALYs	Female	-0.342326841	0
Proportion of cirrhosis due to NASH	Deaths	Male	0.224202666	4.70E-08
Proportion of cirrhosis due to NASH	Deaths	Female	0.131248084	0.001522
Proportion of cirrhosis due to NASH	DALYs	Male	0.188061759	5.02E-06
Proportion of cirrhosis due to NASH	DALYs	Female	0.137147666	0.000919
Proportion of cirrhosis due to other causes	Deaths	Male	0.166680454	5.41E-05
Proportion of cirrhosis due to other causes	Deaths	Female	-0.105424532	0.010998
Proportion of cirrhosis due to other causes	DALYs	Male	0.121032298	0.00348
Proportion of cirrhosis due to other causes	DALYs	Female	-0.067518645	0.103989
Proportion of liver cancer due to alcohol	Deaths	Male	0.525899438	0
Proportion of liver cancer due to alcohol	Deaths	Female	0.202701308	4.96E-07
Proportion of liver cancer due to alcohol	DALYs	Male	0.496521383	0
Proportion of liver cancer due to alcohol	DALYs	Female	0.224307591	2.45E-08
Proportion of liver cancer due to hepatitis B	Deaths	Male	-0.276606296	4.36E-12
Proportion of liver cancer due to hepatitis B	Deaths	Female	-0.233619476	6.06E-09
Proportion of liver cancer due to hepatitis B	DALYs	Male	-0.216777716	7.24E-08
Proportion of liver cancer due to hepatitis B	DALYs	Female	-0.218493509	5.67E-08
Proportion of liver cancer due to hepatitis C	Deaths	Male	0.31685338	1.33E-15
Proportion of liver cancer due to hepatitis C	Deaths	Female	0.149053754	0.000234
Proportion of liver cancer due to hepatitis C	DALYs	Male	0.286159218	7.25E-13
Proportion of liver cancer due to hepatitis C	DALYs	Female	0.128347161	0.001559
Proportion of liver cancer due to NASH	Deaths	Male	-0.048045948	0.237996
Proportion of liver cancer due to NASH	Deaths	Female	-0.312920383	3.33E-15
Proportion of liver cancer due to NASH	DALYs	Male	-0.071071183	0.080687
Proportion of liver cancer due to NASH	DALYs	Female	-0.28274412	1.39E-12
Proportion of liver cancer due to other causes	Deaths	Male	-0.346068307	0

Proportion of liver cancer due to other causes	Deaths	Female	-0.357475063	0
Proportion of liver cancer due to other causes	DALYs	Male	-0.307127627	1.11E-14
Proportion of liver cancer due to other causes	DALYs	Female	-0.307734464	9.77E-15
Proportion of population involved in agricultural activities	DALYs	Male	-0.641286674	0
Proportion of population involved in agricultural activities	Deaths	Male	-0.70382653	0
Proportion of population involved in agricultural activities	Deaths	Female	-0.562748072	0
Proportion of population involved in agricultural activities	DALYs	Female	-0.508548276	0
Proportion of the population aged 15 to 30	Deaths	Male	-0.04646848	0.263454
Proportion of the population aged 15 to 30	Deaths	Female	-0.049242728	0.235975
Proportion of the population aged 15 to 30	DALYs	Male	-0.027423084	0.509443
Proportion of the population aged 15 to 30	DALYs	Female	-0.027912452	0.501914
Proportion of the population over age 65	Deaths	Male	0.620061211	0
Proportion of the population over age 65	Deaths	Female	0.530992081	0
Proportion of the population over age 65	DALYs	Male	0.585430075	0
Proportion of the population over age 65	DALYs	Female	0.490403787	0
Proportion of the population with 0 years of education	Deaths	Male	-0.402084893	0
Proportion of the population with 0 years of education	Deaths	Female	-0.390554479	0
Proportion of the population with 0 years of education	DALYs	Male	-0.383881722	0
Proportion of the population with 0 years of education	DALYs	Female	-0.351298036	0
Proportion of the population with at least 12 years of education	Deaths	Male	0.504014078	0
Proportion of the population with at least 12 years of education	Deaths	Female	0.45892387	0
Proportion of the population with at least 12 years of education	DALYs	Male	0.450683879	0
Proportion of the population with at least 12 years of education	DALYs	Female	0.392572225	0
Proportion of the population with at least 15 years of education	Deaths	Male	0.631336192	0
Proportion of the population with at least 15 years of education	Deaths	Female	0.560789952	0
Proportion of the population with at least 15 years of education	DALYs	Male	0.573505653	0
Proportion of the population with at least 15 years of education	DALYs	Female	0.500504838	0
Proportion of the population with at least 6 years of education	Deaths	Male	0.485717594	0
Proportion of the population with at least 6 years of education	Deaths	Female	0.446744157	0
Proportion of the population with at least 6 years of education	DALYs	Male	0.450665618	0
Proportion of the population with at least 6 years of education	DALYs	Female	0.389951782	0
Syphilis prevalence (proportion)	Deaths	Male	0.050599256	0.213942
Syphilis prevalence (proportion)	Deaths	Female	-0.22859024	1.30E-08
Syphilis prevalence (proportion)	DALYs	Male	0.081733267	0.044476
Syphilis prevalence (proportion)	DALYs	Female	-0.168567296	3.08E-05
Tuberculosis infection risk-weighted prevalence	Deaths	Male	0.625580153	0
Tuberculosis infection risk-weighted prevalence	Deaths	Female	0.391211201	0
Tuberculosis infection risk-weighted prevalence	DALYs	Male	0.602216167	0
Tuberculosis infection risk-weighted prevalence	DALYs	Female	0.398165451	0
Underweight women of reproductive age	Deaths	Male	/	
Underweight women of reproductive age	Deaths	Female	-0.366376192	0
Underweight women of reproductive age	DALYs	Male	/	
Underweight women of reproductive age	DALYs	Female	-0.344560091	0
Vitamin A Deficiency Prevalence	Deaths	Male	-0.574856245	0
Vitamin A Deficiency Prevalence	Deaths	Female	-0.518888691	0
Vitamin A Deficiency Prevalence	DALYs	Male	-0.52240717	0
Vitamin A Deficiency Prevalence	DALYs	Female	-0.454054622	0
Vitamin A supplementation coverage rate	Deaths	Male	-0.445852623	0
Vitamin A supplementation coverage rate	Deaths	Female	-0.428743814	0
Vitamin A supplementation coverage rate	DALYs	Male	-0.478587449	0
Vitamin A supplementation coverage rate	DALYs	Female	-0.471004205	0
Zinc deficiency	Deaths	Male	-0.407503423	0
Zinc deficiency	Deaths	Female	-0.299651181	5.11E-14
Zinc deficiency	DALYs	Male	-0.370931807	0
Zinc deficiency	DALYs	Female	-0.269459529	1.59E-11
