# Supplementary material for: Burden of Aortic Aneurysm and Its Attributable Risk Factors from 1990 to 2019: An Analysis of the Global Burden of Disease Study 2019
Source: Front Cardiovasc Med. 2022 May 31;9:901225. doi: 10.3389/fcvm.2022.901225 (PMC9197430; doi:10.3389/fcvm.2022.901225)
Supplement: Supplementary Table 11 — Correlation analysis of aortic aneurism-related age-standardized deaths and DALYs in 2019. DALY, disability-adjusted life year rate. [file Data_Sheet_11.PDF]

row	column	correlation coefficients	p
10-year lag distributed energy per capita	Death	0.235818004	4.32E-09
10-year lag distributed energy per capita	DALYs	0.216759424	7.26E-08
Legality of Abortion	Death	0.304236768	6.57E-14
Legality of Abortion	DALYs	0.293938109	4.83E-13
Adolescent Birth Rates (10 - 19 years of age)	Death	-0.539834112	0
Adolescent Birth Rates (10 - 19 years of age)	DALYs	-0.483332548	0
adult_hiv_death_rate_both_sexes	Death	-0.546100168	0
adult_hiv_death_rate_both_sexes	DALYs	-0.498010326	0
Antenatal Care (1 visit) Coverage	Death	0.487842284	0
Antenatal Care (1 visit) Coverage	DALYs	0.451960807	0
Antenatal Care (4 visit) Coverage	Death	0.57041551	0
Antenatal Care (4 visit) Coverage	DALYs	0.524286858	0
Areas that are endemic for Japanese encephalitis	Death	-0.144384673	0.000367
Areas that are endemic for Japanese encephalitis	DALYs	-0.155547144	0.0001223
Asbestos consumption (metric tons per year per capita)	DALYs	0.337224511	0
Asbestos consumption (metric tons per year per capita)	Death	0.305380892	4.75E-14
Bacille Calmette-Guerin (BCG) vaccine coverage	Death	-0.613056665	0
Bacille Calmette-Guerin (BCG) vaccine coverage	DALYs	-0.559878374	0
Calcium g/p/d avail	DALYs	0.537990004	0
Calcium g/p/d avail	Death	0.584579888	0
Coastal Population within 10km(proportion)	Death	0.035465378	0.3838618
Coastal Population within 11km(proportion)	DALYs	0.057633571	0.1568213
Demand for contraception satisfied with modern methods	DALYs	0.494096976	0
Demand for contraception satisfied with modern methods	Death	0.548490537	0
Demand for contraception satisfied with modern methods	DALYs	0.494096976	0
Demand for contraception satisfied with modern methods	Death	0.548490537	0
Contraception (Modern) Prevalence	DALYs	0.534565538	0
Contraception (Modern) Prevalence	Death	0.578728305	0
Contraception (Modern) Prevalence	DALYs	0.534565538	0
Contraception (Modern) Prevalence	Death	0.578728305	0
Dentists per capita	Death	0.291982087	6.98E-13
Dentists per capita	DALYs	0.278825933	7.78E-12
DTP3 Coverage	Death	0.43127574	0
DTP3 Coverage	DALYs	0.44043747	0
echinococcosis endemicity	Death	-0.34730391	0
echinococcosis endemicity	DALYs	-0.315058507	2.22E-15
Proportion of the population with 0 years of education	Death	-0.140802709	0.0005138
Proportion of the population with 0 years of education	DALYs	-0.124606234	0.0021367
Proportion of the population with at least 12 years of education	Death	0.485706722	0
Proportion of the population with at least 12 years of education	DALYs	0.437660991	0
Proportion of the population with at least 15 years of education	Death	0.61688571	0
Proportion of the population with at least 15 years of education	DALYs	0.569141219	0
Proportion of the population with at least 6 years of education	Death	0.453483067	0
Proportion of the population with at least 6 years of education	DALYs	0.419856278	0
Education (years per capita)	DALYs	0.528096472	0
Education (years per capita)	Death	0.58416533	0
Education Absolute Inequality (AAID), maternal	Death	-0.276773939	4.22E-12
Education Absolute Inequality (AAID), maternal	DALYs	-0.253405359	2.56E-10
Education Relative Inequality (Gini),maternal	DALYs	-0.427495923	0
Education Relative Inequality (Gini),maternal	Death	-0.456328728	0
Education Relative Inequality (Gini),maternal	DALYs	-0.427495923	0
Education Relative Inequality (Gini),maternal	Death	-0.456328728	0
Energy kcal/p/day avail	DALYs	0.22105357	6.25E-08
Energy kcal/p/day avail	Death	0.244850199	1.84E-09
Enhanced Vegetation Index long term average 2000-2012 (mean)	Death	-0.017952407	0.6594352
Enhanced Vegetation Index long term average 2000-2012 (mean)	DALYs	-0.014267511	0.7261688
Fiber g/p/d avail	Death	-0.212852321	1.25E-07
Fiber g/p/d avail	DALYs	-0.198536988	8.55E-07
Folic Acid ug/p/day energy	Death	0.509674152	0
Folic Acid ug/p/day energy	DALYs	0.47957809	0
Composite fortification standard and folic acid inclusion	Death	-0.086884348	0.0326228
Composite fortification standard and folic acid inclusion	DALYs	-0.084431967	0.0378787
Composite fortification standard and iron inclusion	Death	0.310076704	6.00E-15
Composite fortification standard and iron inclusion	DALYs	0.254821256	2.02E-10
Fortification standard	Death	-0.086884348	0.0326228
Fortification standard	DALYs	-0.084431967	0.0378787
Fortification standard including folic acid	Death	-0.086884348	0.0326228

Fortification standard including folic acid	DALYs	-0.084431967	0.0378787
Fortification standard including iron	Death	0.33010798	0
Fortification standard including iron	DALYs	0.273864132	7.20E-12
Fraction of out-of-pocket health expenditure out of total health expenditure	Death	-0.445703088	0
Fraction of out-of-pocket health expenditure out of total health expenditure	DALYs	-0.401082226	0
Fruits g/p/d avail	DALYs	-0.0318915	0.4405771
Fruits g/p/d avail	Death	-0.047113583	0.2544231
Gallup: Negative Experience Index	Death	-0.146547105	0.0003942
Gallup: Negative Experience Index	DALYs	-0.129607986	0.0017448
GDP per capita	Death	0.490890646	0
GDP per capita	DALYs	0.433961084	0
Mean GDP PPP	Death	0.350300243	3.00E-07
Mean GDP PPP	DALYs	0.29506012	1.92E-05
Gold production	Death	-0.382665202	0
Gold production	DALYs	-0.354765148	0
No access to handwashing facility	Death	-0.542085521	0
No access to handwashing facility	DALYs	-0.487318593	0
Healthcare access and quality index	DALYs	0.584443658	0
Healthcare access and quality index	Death	0.648103494	0
Health expenditure (per capita)	Death	0.552291057	0
Health expenditure (per capita)	DALYs	0.500573001	0
Health worker density	DALYs	0.640715085	0
Health worker density	Death	0.694980322	0
Hemoglobin C	Death	-0.345855231	0
Hemoglobin C	DALYs	-0.33878063	0
Hemoglobin S	Death	-0.018778597	0.6448145
Hemoglobin S	DALYs	-0.022971903	0.572795
Hepatitis B vaccine coverage	Death	0.015624535	0.7013184
Hepatitis B vaccine coverage	DALYs	0.054987684	0.1767777
Hib3 Vaccine Coverage	Death	0.450752854	0
Hib3 Vaccine Coverage	DALYs	0.456372377	0
Hospital Beds (per 1000)	DALYs	0.522485643	0
Hospital Beds (per 1000)	Death	0.515307858	0
Health Industry Workers	DALYs	0.717116663	0
Health Industry Workers	Death	0.757036684	0
Iron mg/p/day energy	DALYs	0.412970292	0
Iron mg/p/day energy	Death	0.438058221	0
Lag distributed income per capita	Death	0.495125912	0
Lag distributed income per capita	DALYs	0.435533343	0
BCG lagged five year coverage (proportion)	Death	-0.492341857	0
BCG lagged five year coverage (proportion)	DALYs	-0.436361466	0
DTP3 lagged five year coverage (proportion)	Death	0.547809194	0
DTP3 lagged five year coverage (proportion)	DALYs	0.535996447	0
HepB3 lagged five year coverage (proportion)	Death	-0.44557167	0
HepB3 lagged five year coverage (proportion)	DALYs	-0.389024405	0
Hib3 lagged five year coverage (proportion)	Death	0.559470719	0
Hib3 lagged five year coverage (proportion)	DALYs	0.540655252	0
MCV1 lagged five year coverage (proportion)	Death	0.427510132	0
MCV1 lagged five year coverage (proportion)	DALYs	0.437590165	0
MCV2 lagged five year coverage (proportion)	Death	0.391057197	0
MCV2 lagged five year coverage (proportion)	DALYs	0.373547438	0
PCV3 lagged five year coverage (proportion)	Death	0.54234275	0
PCV3 lagged five year coverage (proportion)	DALYs	0.517484019	0
Polio3 lagged five year coverage (proportion)	Death	0.548219193	0
Polio3 lagged five year coverage (proportion)	DALYs	0.524051207	0
RCV1 lagged five year coverage (proportion)	Death	0.481578524	0
RCV1 lagged five year coverage (proportion)	DALYs	0.488923064	0
RotaC lagged five year coverage (proportion)	Death	0.478779057	0
RotaC lagged five year coverage (proportion)	DALYs	0.449404599	0
Average latitude	DALYs	0.634815399	0
Average latitude	Death	0.675507227	0
Liters of alcohol consumed per capita	DALYs	0.587916501	0
Liters of alcohol consumed per capita	Death	0.620595477	0
Live Births	Death	-0.601823211	0
Live Births	DALYs	-0.559236164	0
Log-transformed coal production (per capita)	Death	0.697350807	0

Log-transformed coal production (per capita)	DALYs	0.640103357	0
Malaria Lysenko PFPR 1 (Holoendemic)	Death	-0.264485188	3.84E-11
Malaria Lysenko PFPR 1 (Holoendemic)	DALYs	-0.261160091	6.85E-11
Maternal care and immunization	Death	0.597293065	0
Maternal care and immunization	DALYs	0.582707813	0
Mean number of venomous snake species	Death	-0.492514404	0
Mean number of venomous snake species	DALYs	-0.467271917	0
Mean war mortality rate in the previous ten years	Death	-0.113888692	0.0050375
Mean war mortality rate in the previous ten years	DALYs	-0.143032321	0.0004171
Population-weighted mean temperature	DALYs	-0.574593717	0
Population-weighted mean temperature	Death	-0.619981043	0
Measles Vaccine Coverage (proportion)	Death	0.376298884	0
Measles Vaccine Coverage (proportion)	DALYs	0.393803709	0
Measles Vaccine Coverage 2 doses (proportion)	Death	0.219815597	4.69E-08
Measles Vaccine Coverage 2 doses (proportion)	DALYs	0.209987747	1.86E-07
meningitis belt (proportion)	Death	-0.214325961	1.02E-07
meningitis belt (proportion)	DALYs	-0.200760499	6.40E-07
Milk g/p/d avail	DALYs	0.502728607	0
Milk g/p/d avail	Death	0.529884573	0
Mortality Rate Due to Death Shocks in Last 10 Years	Death	-0.523066745	0
Mortality Rate Due to Death Shocks in Last 10 Years	DALYs	-0.482710065	0
Nurses and Midwives per capita	Death	0.725526954	0
Nurses and Midwives per capita	DALYs	0.6618849	0
Nuts & Seeds g/p/d avail	DALYs	-0.064349831	0.119384
Nuts & Seeds g/p/d avail	Death	-0.0622672	0.1318461
Opioids per million population per day	Death	0.584169515	0
Opioids per million population per day	DALYs	0.535084954	0
PAHO's estimates of Chagas prevalence	Death	-0.264425335	3.88E-11
PAHO's estimates of Chagas prevalence	DALYs	-0.208198773	2.38E-07
PCV3 Coverage	Death	0.529701313	0
PCV3 Coverage	DALYs	0.518456328	0
Percent coverage of children (0-5 years) with diarrhea within the past 2 weeks that received oral rehydration solution	Death	0.442198452	0
Percent coverage of children (0-5 years) with diarrhea within the past 2 weeks that received oral rehydration solution	DALYs	0.356166863	0
Percent coverage of children (0-5 years) with lower respiratory infection	Death	0.58135822	0
Percent coverage of children (0-5 years) with lower respiratory infection	DALYs	0.5180115	0
Percent of women giving birth in a health facility	Death	0.424181129	0
Percent of women giving birth in a health facility	DALYs	0.421629232	0
pharmacists_pc	DALYs	0.532962185	0
pharmacists_pc	Death	0.581341294	0
physicians_pc	DALYs	0.481932056	0
physicians_pc	Death	0.517167011	0
Pigs (per capita)	Death	0.009936158	0.8111128
Pigs (per capita)	DALYs	0.044285941	0.2865639
Pigs raised in extensive agricultural systems per capita	Death	-0.510568031	0
Pigs raised in extensive agricultural systems per capita	DALYs	-0.446945437	0
Pigs raised in intensive/industrial agricultural systems per capita	Death	0.212514165	2.33E-07
Pigs raised in intensive/industrial agricultural systems per capita	DALYs	0.221880811	6.51E-08
Pigs raised in semi-extensive agricultural systems per capita	Death	-0.281449612	4.86E-12
Pigs raised in semi-extensive agricultural systems per capita	DALYs	-0.283124026	3.59E-12
Polio 3-dose coverage (proportion)	Death	0.462466738	0
Polio 3-dose coverage (proportion)	DALYs	0.460018246	0
Indoor Air Pollution (All Cooking Fuels)	Death	-0.475189509	0
Indoor Air Pollution (All Cooking Fuels)	DALYs	-0.426957579	0
Outdoor Air Pollution (PM2.5)	Death	-0.506280283	0
Outdoor Air Pollution (PM2.5)	DALYs	-0.481012486	0
Elevation 100 to 500m (proportion)	DALYs	-0.151601021	0.0002389
Elevation 100 to 500m (proportion)	Death	-0.183642129	8.10E-06
Elevation Over 1500m (proportion)	DALYs	-0.531619234	0
Elevation Over 1500m (proportion)	Death	-0.549808038	0
Elevation 500 to 1500m (proportion)	DALYs	-0.514062298	0
Elevation 500 to 1500m (proportion)	Death	-0.554703693	0
Population Density (150-300 ppl/sqkm, proportion)	DALYs	-0.28411051	2.75E-12
Population Density (150-300 ppl/sqkm, proportion)	Death	-0.295583983	3.22E-13
Population Density (300-500 ppl/sqkm, proportion)	DALYs	-0.225434153	3.74E-08
Population Density (300-500 ppl/sqkm, proportion)	Death	-0.233543225	1.16E-08
Population Density (500-1000 ppl/sqkm, proportion)	DALYs	-0.119768637	0.0037785

Population Density (500-1000 ppl/sqkm, proportion)	Death	-0.117922996	0.0043563
Population Density (over 1000 ppl/sqkm, proportion)	DALYs	0.460252485	0
Population Density (over 1000 ppl/sqkm, proportion)	Death	0.493882966	0
Population Density (under 150 ppl/sqkm, proportion)	DALYs	-0.419070644	0
Population Density (under 150 ppl/sqkm, proportion)	Death	-0.461266732	0
Population weighted probability of dengue transmission	Death	-0.613515101	0
Population weighted probability of dengue transmission	DALYs	-0.553330819	0
Poultry g/p/day energy	DALYs	0.305826159	3.55E-14
Poultry g/p/day energy	Death	0.3264609	4.44E-16
Urbanicity	DALYs	0.405851198	0
Urbanicity	Death	0.456852622	0
Proportion of households using iodized salt	Death	0.488688291	0
Proportion of households using iodized salt	DALYs	0.456219156	0
Proportion of live births by mothers age 35 and older	DALYs	0.46620925	0
Proportion of live births by mothers age 35 and older	Death	0.515704639	0
Proportion of live births by mothers age 40 and older	DALYs	0.095017638	0.0217614
Proportion of live births by mothers age 40 and older	Death	0.113575623	0.006045
Proportion of population vulnerable to venomous snakebites	Death	-0.522263289	0
Proportion of population vulnerable to venomous snakebites	DALYs	-0.487894255	0
Proportion of the population living in low income counties and within the classic monsoon region	Death	-0.47003337	0
Proportion of the population living in low income counties and within the classic monsoon region	DALYs	-0.474598652	0
Proportion of the population living in the classic monsoon region	Death	-0.383208277	0
Proportion of the population living in the classic monsoon region	DALYs	-0.386246766	0
Proportion of the population living in the Indian Ocean monsoon belt	Death	-0.329041762	4.44E-16
Proportion of the population living in the Indian Ocean monsoon belt	DALYs	-0.339691902	0
Proportion of the population that are males aged 15 to 30	Death	-0.593880392	0
Proportion of the population that are males aged 15 to 30	DALYs	-0.565382876	0
Proportion of total population covered by menafrivac initiative	Death	-0.20194629	5.48E-07
Proportion of total population covered by menafrivac initiative	DALYs	-0.190832016	2.27E-06
Percent of total calories available as pufa	DALYs	0.318018954	2.89E-15
Percent of total calories available as pufa	Death	0.327920576	4.44E-16
Pulses & Legumes g/p/d avail	Death	-0.234981427	9.91E-09
Pulses & Legumes g/p/d avail	DALYs	-0.225288417	4.03E-08
Rainfall, population-weighted (mm/yr)	DALYs	-0.144638868	0.0004595
Rainfall, population-weighted (mm/yr)	Death	-0.173558393	2.51E-05
Percent of the population living in the 2nd, 3rd, 4th or 5th world quintile of annual rainfall (in millimeters) ("5=most rain, 1=least rain")	Death	0.448411409	0
Percent of the population living in the 2nd, 3rd, 4th or 5th world quintile of annual rainfall (in millimeters) ("5=most rain, 1=least rain")	DALYs	0.43352993	0
Percent of the population living in the 3rd, 4th or 5th world quintile of annual rainfall (in millimeters) ("5=most rain, 1=least rain")	Death	0.493510911	0
Percent of the population living in the 3rd, 4th or 5th world quintile of annual rainfall (in millimeters) ("5=most rain, 1=least rain")	DALYs	0.472553563	0
Percent of the population living in the 4th or 5th world quintile of annual rainfall, inclusive (in millimeters) ("5=most rain, 1=least rain")	Death	0.385340191	0
Percent of the population living in the 4th or 5th world quintile of annual rainfall, inclusive (in millimeters) ("5=most rain, 1=least rain")	DALYs	0.358747764	0
Percent of the population living in the bottom world quintile of annual rainfall (in millimeters) ("5=most rain, 1=least rain")	Death	-0.329600816	4.44E-16
Percent of the population living in the bottom world quintile of annual rainfall (in millimeters) ("5=most rain, 1=least rain")	DALYs	-0.323445254	1.33E-15
Percent of the population living in the 2nd world quintile of annual rainfall (in millimeters) ("5=most rain, 1=least rain")	Death	-0.353603989	0
Percent of the population living in the 2nd world quintile of annual rainfall (in millimeters) ("5=most rain, 1=least rain")	DALYs	-0.33601197	0
Percent of the population living in the 3rd world quintile of annual rainfall (in millimeters) ("5=most rain, 1=least rain")	Death	-0.196845634	1.74E-06
Percent of the population living in the 3rd world quintile of annual rainfall (in millimeters) ("5=most rain, 1=least rain")	DALYs	-0.174146009	2.43E-05
Percent of the population living in the 4th world quintile of annual rainfall (in millimeters) ("5=most rain, 1=least rain")	Death	0.477582851	0

Percent of the population living in the 4th world quintile of annual rainfall (in millimeters) ("5=most rain, 1=least rain")	DALYs	0.437014477	0
Percent of the population living in the top world quintile of annual rainfall (in millimeters) ("5=most rain, 1=least rain")	Death	-0.25544151	4.16E-10
Percent of the population living in the top world quintile of annual rainfall (in millimeters) ("5=most rain, 1=least rain")	DALYs	-0.221193557	7.16E-08
Red Meats g/p/d avail	DALYs	0.465121158	0
Red Meats g/p/d avail	Death	0.479183237	0
Muslim Religion (proportion of population)	Death	-0.123995684	0.0027543
Muslim Religion (proportion of population)	DALYs	-0.15696313	0.0001454
Residential radon	Death	0.123719906	0.0028155
Residential radon	DALYs	0.114862823	0.0055736
Rotavirus coverage (proportion)	Death	0.453327459	0
Rotavirus coverage (proportion)	DALYs	0.430243273	0
Rotavirus coverage (proportion)	Death	0.323854996	1.33E-15
Rotavirus coverage (proportion)	DALYs	0.346696688	0
Sanitation (proportion with access)	DALYs	0.526836547	0
Sanitation (proportion with access)	Death	0.556992561	0
Schisto cumulative treatments	Death	-0.435272093	0
Schisto cumulative treatments	DALYs	-0.413521198	0
Skilled Birth Attendance (proportion)	Death	0.427833277	0
Skilled Birth Attendance (proportion)	DALYs	0.416230153	0
Stillbirth to live birth ratio	Death	-0.479651336	0
Stillbirth to live birth ratio	DALYs	-0.446074257	0
Sugar g/p/d avail	DALYs	0.140366327	0.0006485
Sugar g/p/d avail	Death	0.159924481	9.96E-05
TB strain prevalence-weighted transmission RR	Death	0.255479413	1.80E-10
TB strain prevalence-weighted transmission RR	DALYs	0.22110768	3.90E-08
Tetanus Toxoid Coverage	Death	0.614627465	0
Tetanus Toxoid Coverage	DALYs	0.555046058	0
the strictness of blood alcohol content law for professional drivers	Death	-0.026039958	0.5310394
the strictness of blood alcohol content law for professional drivers	DALYs	-0.079824387	0.054479
the strictness of blood alcohol content law for the general population	Death	-0.065705626	0.113635
the strictness of blood alcohol content law for the general population	DALYs	-0.118071595	0.0043742
the strictness of blood alcohol content law for youth drivers	Death	-0.033844221	0.4154986
the strictness of blood alcohol content law for youth drivers	DALYs	-0.086479242	0.0371687
Total Fertility Rate	Death	-0.405175816	0
Total Fertility Rate	DALYs	-0.393113959	0
Total Vitamin A Retinol Activity Equivalents ug/p/day	DALYs	0.362717306	0
Total Vitamin A Retinol Activity Equivalents ug/p/day	Death	0.407382375	0
Total Vitamin A Retinol ug/p/day	DALYs	0.487118453	0
Total Vitamin A Retinol ug/p/day	Death	0.522477861	0
UHC.effective.coverage.index	Death	0.206999836	0.0030442
UHC.effective.coverage.index	DALYs	0.151183041	0.0313095
Universal health coverage	DALYs	0.590253906	0
Universal health coverage	Death	0.654730437	0
Vegetables g/p/d avail	DALYs	0.265084053	6.76E-11
Vegetables g/p/d avail	Death	0.301700587	8.08E-14
Improved Water Source (proportion with access)	DALYs	0.233602569	1.15E-08
Improved Water Source (proportion with access)	Death	0.222023314	6.06E-08
Zinc mg/p/day	DALYs	0.307858302	2.35E-14
Zinc mg/p/day	Death	0.343870153	0
Zinc treatment for diarrhea	DALYs	0.440884781	0
Zinc treatment for diarrhea	Death	0.487912929	0
Number of 2 and 4 wheeled vehicles per capita	Death	0.477142753	0
Number of 2 and 4 wheeled vehicles per capita	DALYs	0.424339984	0
Number of 2 wheeled vehicles per capita	Death	0.20095428	6.24E-07
Number of 2 wheeled vehicles per capita	DALYs	0.197793614	9.41E-07
Number of 4 wheeled vehicles per capita	Death	0.535896822	0
Number of 4 wheeled vehicles per capita	DALYs	0.488435635	0
Number of 2 wheeled vehicles divided by the number of 2 and 4 wheeled vehicles in the country	Death	-0.325688005	2.22E-16
Number of 2 wheeled vehicles divided by the number of 2 and 4 wheeled vehicles in the country	DALYs	-0.291919945	2.38E-13
